# Regiodivergent (3 + 2) annulation reactions of oxyallyl cations†

**DOI:** 10.1039/d2sc06999g

**Published:** 2023-04-24

**Authors:** Zachary Protich, Leah L. Lowder, Russell P. Hughes, Jimmy Wu

**Affiliations:** a Department of Chemistry, Dartmouth College Hanover New Hampshire 03755 USA jimmy.wu@dartmouth.edu russell.p.hughes@dartmouth.edu

## Abstract

We report a new method for the regiodivergent dearomative (3 + 2) reaction between 3-substituted indoles and oxyallyl cations. Access to both regioisomeric products is possible and is contingent on the presence or absence of a bromine atom on the substituted oxyallyl cation. In this way, we are able to prepare molecules that contain highly-hindered, stereodefined, vicinal, quaternary centers. Detailed computational studies employing energy decomposition analysis (EDA) at the DFT level establishes that regiochemical control arises from either reactant distortion energy or orbital mixing and dispersive forces, depending on the oxyallyl cation. Examination of the Natural Orbitals for Chemical Valence (NOCV) confirms that indole acts as the nucleophilic partner in the annulation reaction.

## Abstract 2

We report a new method for the regiodivergent dearomative (3 + 2) reaction between 3-substituted indoles and oxyallyl cations.

## Graphical abstract

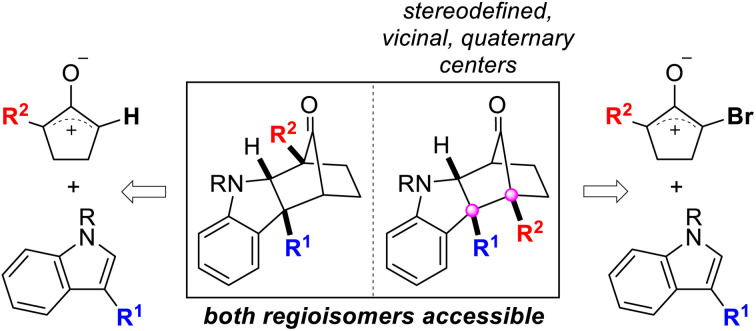

## Introduction

**Scheme 1:**
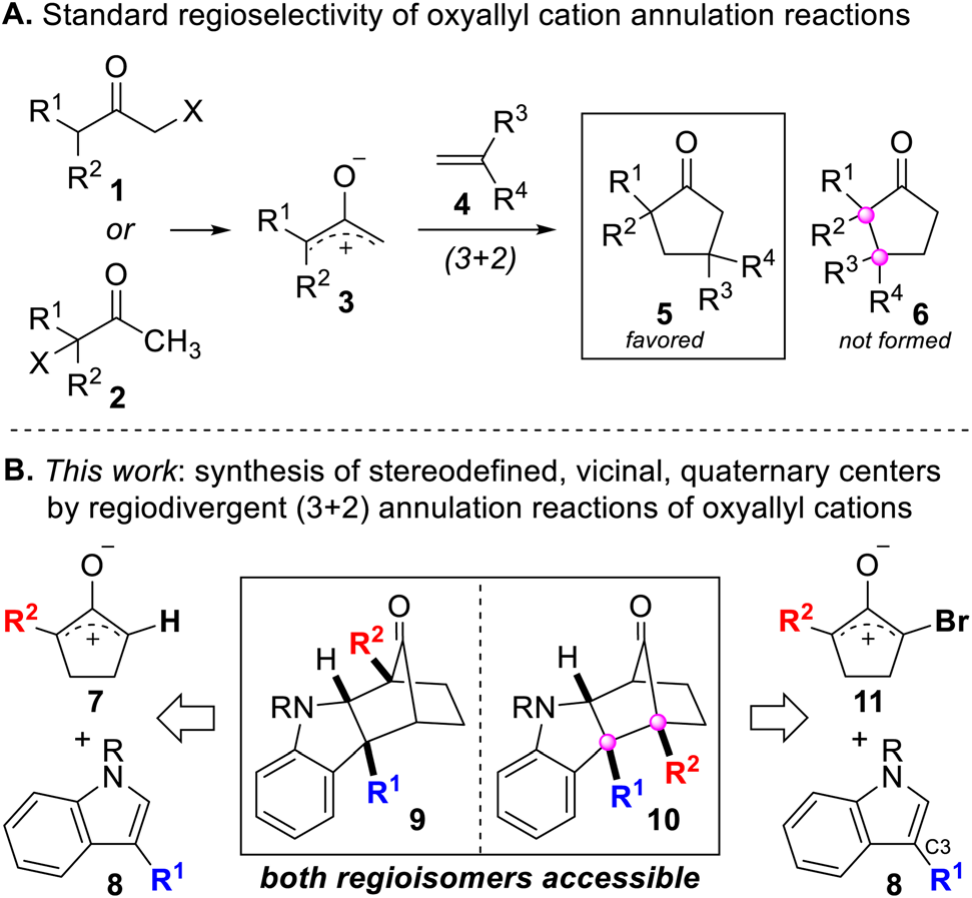
(A) Standard regioselectivity for annulation reactions of oxyallyl cations. (B) Regiodivergent synthesis of stereodefined, vicinal, quaternary centers by (3 + 2) annulation reactions of oxyallyl cations.

Stereodefined, vicinal, quaternary centers are featured in numerous natural products and molecules of biomedical relevance, and they define challenging structural motifs for stereoselective synthesis.^1,2^ Many of the prevailing approaches for establishing stereodefined, vicinal, quaternary centers rely on substrates containing a pre-existing quaternary center and employ a single carbon–carbon bond-forming step to make the second quaternary center.^3–12^ However, few processes are capable of *simultaneously* generating both quaternary centers of the vicinally-substituted system by construction of the bond between the two fully-substituted carbon atoms, and even fewer can do so in an intermolecular and convergent fashion. Cycloaddition reactions have been appreciated as a class of reactions that can be leveraged to achieve the synthesis of stereodefined, vicinal, quaternary centers yet can be extremely challenging to control. The standard regioselectivity for (3 + 2) annulation reactions of oxyallyl cations typically leads to the formation of products such as 5 (Scheme 1A) that possess non-adjacent quaternary centers. Herein, we describe our latest studies of (3 + 2) annulation reactions between oxyallyl cations and indoles in which we have discovered that the inherent regioselectivity of this reaction can be *reversed* to alter the course of bond formation to furnish products 6 and 10 that contain stereodefined, *vicinal*, quaternary centers. The ability to reverse the regiochemical course of a ring-forming reaction is uncommon^13–24^ and generally not feasible. Thus, the lack of methods to “dial-in” the desired regioselective outcome of intermolecular ring-forming reactions underscores the need for additional research in this area. To the best of our knowledge, there is no reported way to reverse the intrinsic regioselectivity of annulation reactions involving oxyallyl cations, which makes this work an attractive and novel approach for synthesizing stereodefined, vicinal, quaternary centers.

Our group recently disclosed the dearomative annulation reaction between oxyallyl cations and 3-substituted indoles.^25,26^ In this manuscript, we report a highly regioselective variant that utilizes unsymmetrical cyclopentyl–oxyallyl cations (Scheme 1B). A key finding of this study is that the presence of a single bromine atom on the oxyallyl cation (11) leads to a surprising reversal of regioselectivity in the annulation reaction. DFT and NOCV studies that shed light on the origins of the observed selectivity are also reported. Notably, compound 10 in Scheme 1B features a pair of stereodefined vicinal quaternary centers, a structural element that is prevalent in complex natural products but is exceedingly hard to make due to the highly congested steric environment.

## Results and discussion

### Regiodivergent annulation reactions

We began our investigation by examining the reaction between indole 8a (R^1^ = Me) and monobrominated α-bromocyclopentanone 12a (R^2^ = Me) in the presence of Na_2_CO_3_ and TFE as solvent at 40 °C. 12 is the precursor to oxyallyl cation 7 (Scheme 1B), which is generated by enolization followed by loss of bromide. After a brief optimization study, we were delighted to find that the desired annulation product 9a could be obtained in 80% yield (formed in 8 : 1 dr along with the minor diastereomer 13a). Notably, 9 and 13 are chromatographically separable stereoisomers of the same regioisomeric pairing, and none of the regioisomeric compound 10 was detected. The gross structures and relative stereochemical assignments of 9a and 13a were confirmed by single-crystal X-ray crystallography for both compounds. We were pleased that the annulation reaction was also amenable to the use of tryptamine- and tryptophol-derived indoles 8b (R^1^ = –(CH_2_)_2_NPhth) and 8c (R^1^ = –(CH_2_)_2_OAc) and variation of the substituent on the bromocyclopentanone (12a–e; R^2^ = Me, Et, i-Pr, Bn, and –CH_2_CO_2_Et) (Table 1 caption). The yields were good in all cases with diastereoselectivities ranging from 4 : 1 to as high as 10 : 1. The structures for every compound 9a–k and 13a–k were verified by 1D- and 2D-NMR spectroscopic analysis.

**Table 1:** (3 + 2) annulation with monobrominated cyclopentanones^a^^,^^b^^,^^c^^,^^d^

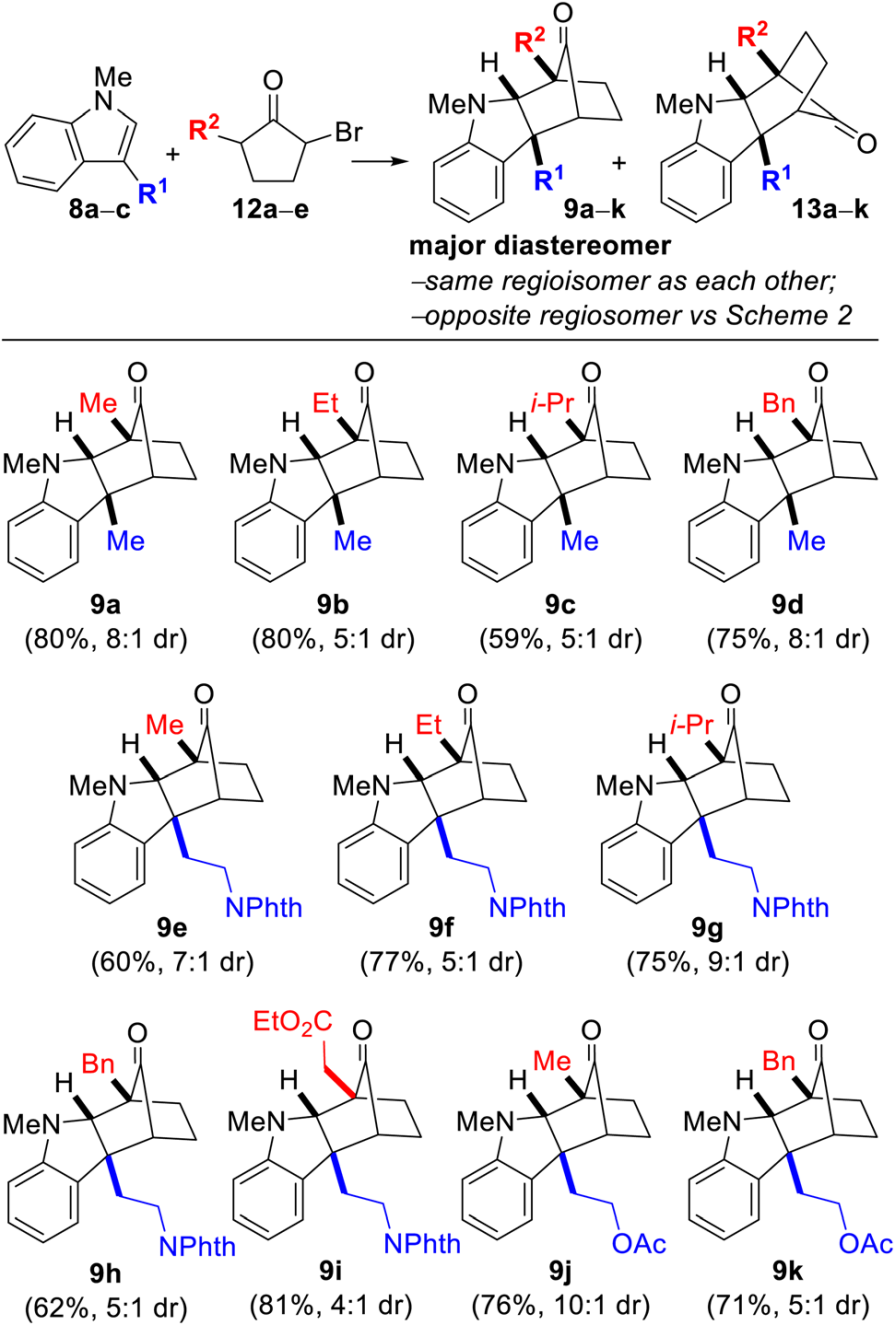

a Reaction conditions: 8 (1.0 equiv.), 12 (1.3 equiv.), Na_2_CO_3_ (3.0 equiv.), TFE [0.2 M], 40 °C.

b Yields are after purification and only of the major diastereomer depicted.

c Diastereoselectivities were determined by ^1^H NMR spectroscopy after workup but prior to column chromatography.

d 8a; R^1^ = Me, 8b; R^1^ = –(CH_2_)_2_NPhth, 8c; R^1^ = –(CH_2_)_2_OAc, 12a; R^2^ = Me; 12b; R^2^ = Et; 12c; R^2^ = i-Pr; 12d; R^2^ = Bn; 12e; R^2^ = –CH_2_CO_2_Et.

Next, we explored the reaction between dibrominated cyclopentanone 14f (R^2^ = H), the precursor to monobrominated oxyallyl cation 11 (Scheme 1B) (Table 2). As expected, the only regioisomer we observed and isolated was compound 15a. This result is consistent with a sterically-controlled, empirical model of regioselectivity in which the first C–C bond-forming event takes place between the C3 position of indole and the less hindered carbon of bromooxyallyl cation 11 (*i.e.*, carbon not bearing the bromine when R^2^ = H).

**Table 2:** (3 + 2) annulation to give stereodefined, vicinal, quaternary centers^b^^,^^c^^,^^d^^,^^e^

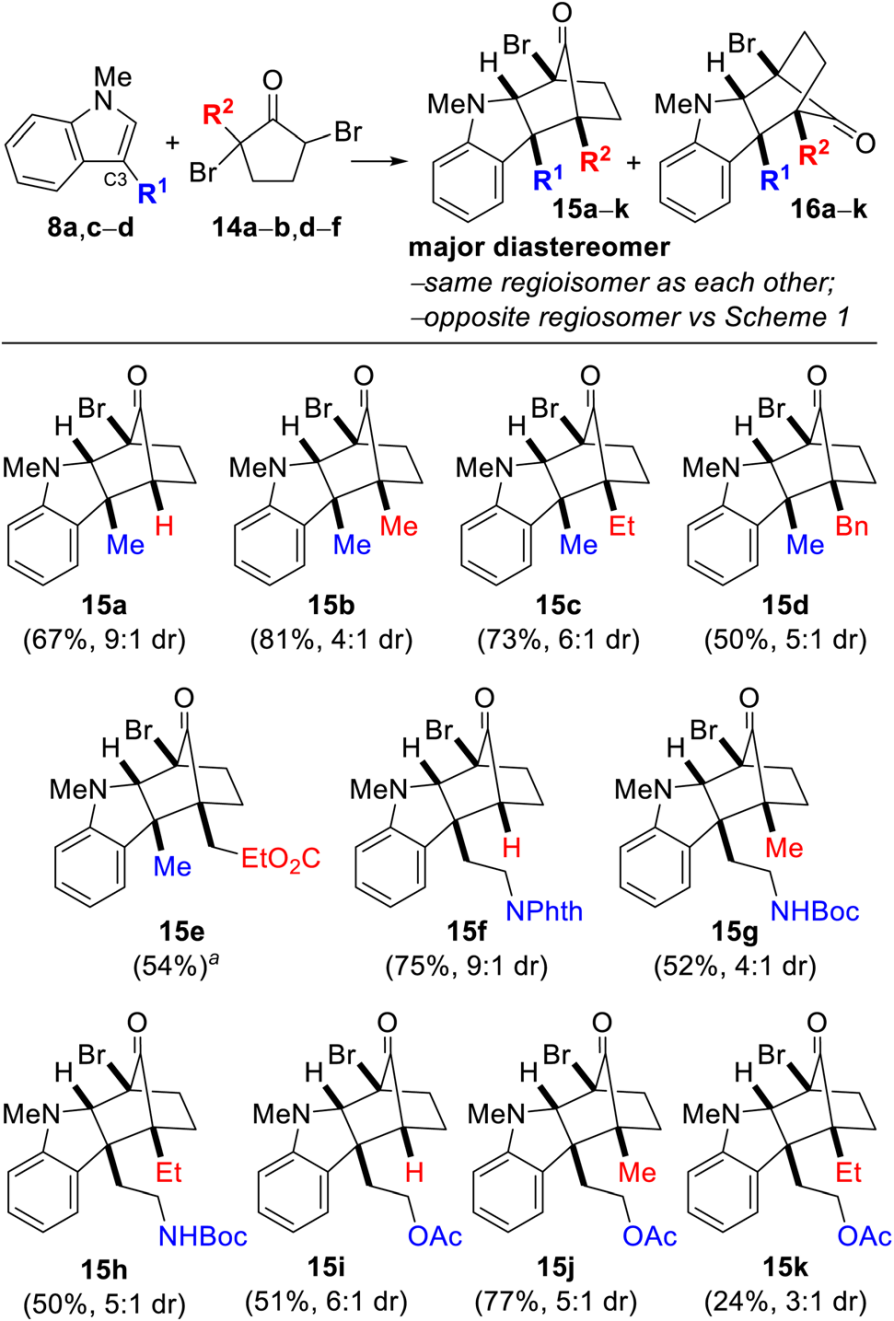

a dr not determined because an analytically pure sample of 16e could not be obtained.

b Reaction conditions: 8 (1.0 equiv.), 14 (2.0 equiv.), Na_2_CO_3_ (3.0 equiv.), TFE [0.2 M], 40 °C.

c Yields are after purification and only of the major diastereomer depicted.

d Diastereoselectivities were determined by ^1^H NMR spectroscopy after workup but prior to column chromatography.

e 8d; R^1^ = –(CH_2_)_2_NHBoc, 14a; R^2^ = Me; 14b; R^2^ = Et; 14c; R^2^ = i-Pr; 14d; R^2^ = Bn; 14e; R^2^ = –CH_2_CO_2_Et; 14f; R^2^ = H.

However, we were surprised when the use of dibromomethylcyclopentanone 14b (R^2^ = Me) lead to the formation of 15b, along with a small amount of the minor diastereomer 16b. As before, 15b and 16b represent the same regioisomeric pairing as each other but are *opposite* to that of the compounds depicted in Table 1. Once again, none of the other possible regioisomer was detected. This unexpected result indicated that the initial C–C bond-forming event had occurred at the carbon bearing the methyl group of oxyallyl cation 11 (R^2^ = Me), despite being the more sterically-hindered position. The gross structures and relative stereochemical assignments of 15b and 16b were confirmed by single-crystal X-ray crystallography for both compounds. We found that the reversal in regioselectivity, presumably due to the presence of a bromine atom on the oxyallyl cation, was a general phenomenon. Thus, the use of tryptamine and tryptophol derivatives 8c (R^1^ = –(CH_2_)_2_OAc) and 8d (R^1^ = –(CH_2_)_2_NHBoc) and dibromo-cyclopentanones 14a–b, d–f all lead to the formation of 15a–k, with varying amounts of diastereomer 16a–k. Most of the molecules depicted in Table 2 contain contiguous quaternary stereocenters. Vicinal quaternary centers have previously been reported to be prepared in either one or two steps, but typically at least one of these steps is intramolecular to compensate for the highly unfavorable steric interactions. In this case, it is remarkable that both quaternary centers are created in a single intermolecular chemical operation in a stereo- and regioselective manner, and under mild reaction conditions. The structural and relative stereochemical assignments of the majority of the compounds in Table 2 were confirmed by 1D- and 2D-NMR spectroscopic analysis with the remaining assigned by analogy.

Having identified a means to ostensibly reverse the regioselectivity of the dearomative annulation reaction between 3-substituted indoles and oxyallyl cations, we then attempted to convert pseudo-regioisomer (due to the presence of Br) 15b to 10b, the true regioisomer of 9a. The desired dehalogenation was accomplished by subjecting 15b to Bu_3_SnH and catalytic AIBN to give 10b (eqn (1)), whose structure was confirmed by 2D-NMR analyses and single-crystal X-ray crystallography.1
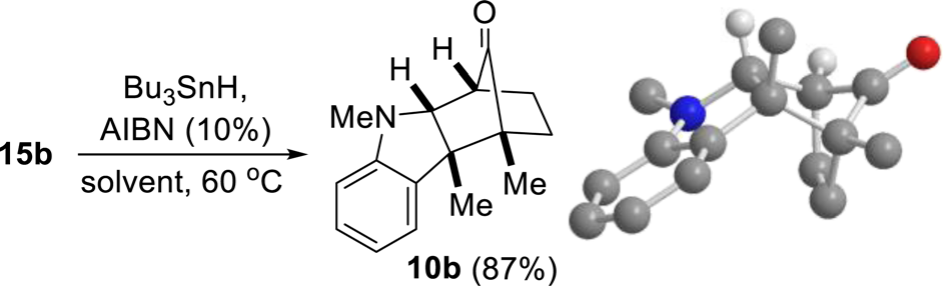


### DFT studies

Since no intermediates were observed experimentally in these reactions, free energy landscapes were explored using Density Functional Theory (DFT)^27,28^ with a view to gaining insight into their complete regioselectivity and preferred stereochemical outcome. Initial calculations were carried out using the B3LYP-D3 functional,^29–33^ combined with the Grimme D3 dispersion correction,^34,35^ and the 6-311G** basis set,^36–39^ to obtain optimized structures. For molecules containing bromine, the Los Alamos core potential was used.^40–43^ Final free energies were obtained by single point calculations using B3LYP-D3 and the def2-tzvp basis,^44,45^ with an implicit Poisson–Boltzmann^7^ solvent model for trifluoroethanol (TFE). Three systems were studied in detail. Formation of 9a and 15a were selected to compare the effect of H *versus* Me and Br substituents on the oxyallyl reactant, while 15b was chosen to probe the notable change in regiochemistry *vis-à-vis*9a.

**Fig. 1:**
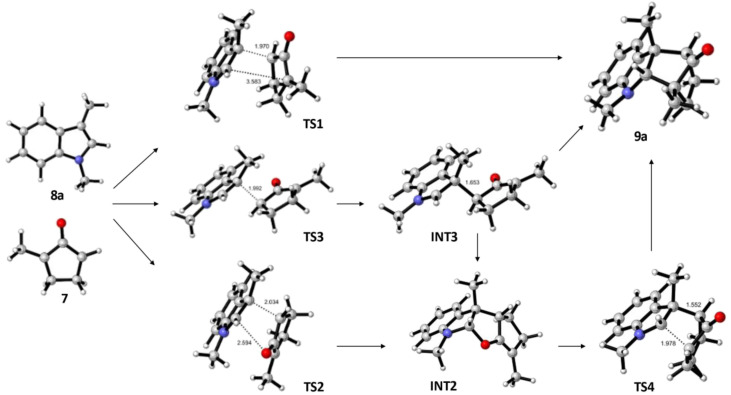
DFT located transition structures (TS) and intermediates (INT) leading to formation of the major isomer 9a. Relative free energies are shown in Fig. 3.

**Fig. 2:**
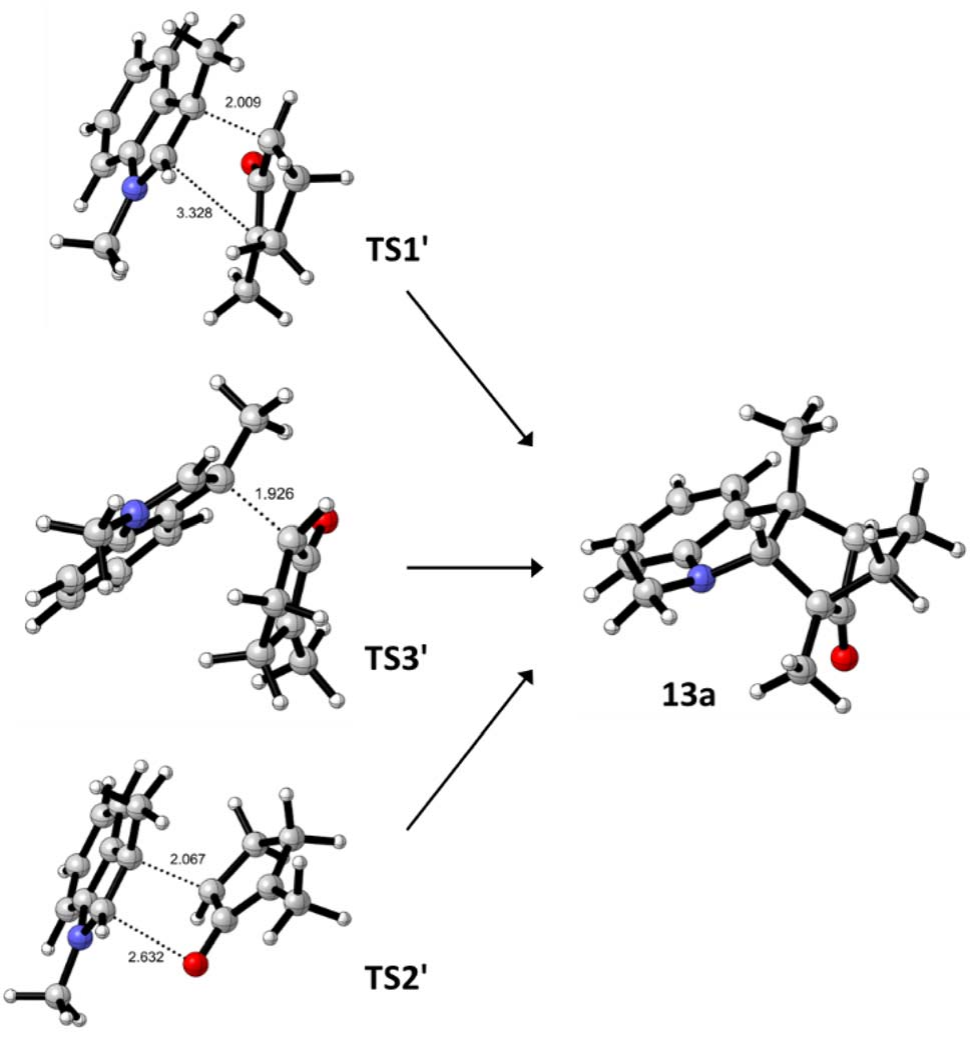
DFT located transition structures (TS′) leading to formation of the minor isomer 13a. Relative free energies are shown in Fig. 3.

**Fig. 3:**
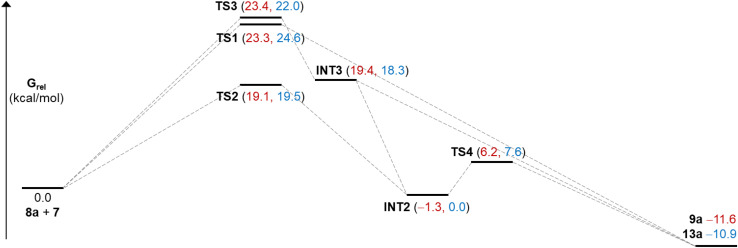
Relative free energies (DFT/B3LYP-D3/def2-tzvp/TFE) for species illustrated in Fig. 1 and 2. Numbers in red are for the major stereoisomer 9a and in blue for minor regioisomer 13a.

An investigation of the formation of major diastereomer 9a and its minor isomer 13a from 8a and oxyallyl cation 7 (R^2^ = Me) derived from 12a located three different transition structures in each reaction manifold, each of which can eventually lead to the final product. These transition structures (TS) and associated intermediates (INT) leading to 9a are illustrated in Fig. 1 while the corresponding three diastereomeric transition structures leading to 13a are depicted in Fig. 2. Relative free energy profiles are shown in Fig. 3. Overall stereochemistry and regiochemistry is determined by which face of the oxyallyl reagent is presented to the indole, and the orientation of that face with respect to the carbonyl group.

For formation of major diastereomer 9a, two initial reaction conformations were examined in detail (Fig. 1). In the first, approach of the two reactants was allowed to occur in a staggered conformation with initial formation of a single C–C bond, *via*TS3, to afford intermediate INT3. Low energy rotation about the new C–C single bond and formation of the second C–C bond affords product 9a; no transition structure for formation of this second C–C bond could be located (see below). Alternatively, similar rotation in INT3 can result in formation of a new C–O bond to give the cyclic ether intermediate INT2, which is more stable than the starting materials. Formation of final product 9a can occur from this intermediate by dissociation of the C–O bond and formation of the second C–C bond *via*TS4. Approach of the reactants in an eclipsed conformation results in two located transition structures. Transition structure (TS1) involves highly asynchronous formation of the two C–C bonds (1.97 and 3.58 Å) and is essentially isoenergetic with TS3 which suggests that there is no additional stabilization from incipient (3.58 Å) formation of the second C–C bond. While Fig. 1 shows TS1 evolving directly to product, it may also generate INT3. Finally, transition structure TS2, while still asynchronous, illustrates a much closer interaction of the O atom (2.59 Å) and leads directly to INT2. Structure TS2 is the lowest energy transition structure (Fig. 3) and represents the rate limiting barrier in the lowest energy pathway to the final product. The regio- and stereochemistry of this reaction is set by the formation of the first C–C bond. The pathway defined in Fig. 1 is reminiscent of that found previously for reactions of acyclic oxyallyl species with dimethylindole (8a);^25^ unlike that system, however, the intermediate cyclic ether could not be isolated or observed in these reactions. The barrier for its conversion to the final product is low (Fig. 3), or as discussed previously, it is possible that traces of protic acid catalyze the ring opening of the cyclic ether and provide an even more facile route to the final product.

Corresponding reaction pathways for formation of the observed minor stereoisomer 13a were also examined and the analogous transition structures are shown in Fig. 2. Final product 13a is slightly less stable than 9a. Once again, the lowest energy pathway is *via*TS2′, which is slightly less favored than the corresponding TS2; this is consistent with the diastereomeric ratio observed experimentally.

The analogous pathways for formation of the major stereoisomer of the experimentally absent regioisomer 10a were also examined. Table 3 presents key free energies for the lowest energy pathways *via* the cyclic ether intermediate for these and reactions of other differently substituted oxyallyl cations. The lowest transition structure (TS2) for formation of 10a lies at 23.8 kcal mol^−1^ (entry 3), sufficiently high to preclude its formation relative to 9a and 13a. Also, the distribution of products does not reflect their relative thermodynamic stabilities but is consistent with kinetic control of regiochemistry and stereochemistry *via*TS2.

**Table 3:** Relative free energies (kcal mol^−1^) of transition structures (TS2) and intermediates (INT2) on the lowest energy pathways (Fig. 3) for reactions of 8a with differently substituted oxyallyl cations

Oxyallyl cation	TS2^a^	INT2	Product	Product number
7 (R^2^ = Me)	19.1	−1.3	−11.6	9a (major)
	19.5	0.0	−10.9	13a (minor)
	23.8	3.4	−10.8	10a (not observed)
11 (R^2^ = H)	14.8	−3.4	−14.1	15a (major)
	15.4	−1.8	−12.8	16a (minor)
	21.9	−5.9	−13.3	Regioisomer (not observed)
11 (R^2^ = Me)	22.9	7.4	−7.1	15b (major)
	23.5	8.6	−5.5	16b (minor)
	25.7	−1.1	−6.9	Regioisomer (not observed)

a All energies are relative to *G*_(__8a__+oxyallyl)_ = 0.0.

Analogous calculations were performed for the corresponding bromooxyallyl cation 11 (R^2^ = H) with very similar results. Once again, calculations are consistent with kinetic control of product regio- and stereochemistry *via*TS2; product stabilities are not reflective of product distribution. In this case TS2 lies significantly lower than that for oxyallyl cation 7 (R^2^ = Me). In unsymmetrically substituted oxyallyl cations, there is a strong preference for the unsubstituted oxyallyl carbon to form the first C–C bond with C3 of the indole. At first blush this would seem to be a steric effect, with preferential formation of the first C–C bond at the less substituted carbon. However, when both CH_3_ and Br substituents are present on the bromooxyallyl cation 11 (R^2^ = Me) the reaction exhibits regiochemistry in which the CH_3_ substituted carbon forms the first C–C bond with C3 of the methylated indole. While calculated activation free energies are also consistent with this regiochemistry and the preferred stereochemical outcome (Table 3), the underlying origins of this regiochemical preference are less apparent and cannot be readily attributed to a steric preference for formation of the first C–C bond.

To further understand the underlying reasons for these substituent effects the transition structures were subjected to an Energy Decomposition Analysis (EDA),^46–48^ including examination of the Natural Orbitals for Chemical Valence (NOCV).^49–52^ In EDA analyses, reactant wavefunctions in the transition state are evaluated independently *in their transition state geometries*, which differ from those in the starting materials. The energy difference due to structural and electronic reorganization from the ground state to the transition structure is *E*_prep_, which is always positive. At this stage, the total energy of the transition structure consists of an overall repulsive interaction *E*_steric_ (for neutral fragments), which is a combination of Pauli repulsion (*E*_Pauli_) attenuated by attractive electrostatic interactions (*E*_estat_) between the total charge distributions in the two reacting molecules. The EDA method partitions these components computationally, but they are sometimes left combined as a net repulsive *E*_steric_ to represent an overall “steric wall” that limits the distance (bond length) between reactants. Finally, relaxation of the wavefunction by allowing orbital mixing between reactant fragments gives a net stabilization (*E*_orb_) due to electron sharing from this overlap, together with polarization of electrons in the resultant molecular orbitals. A crucial attractive interaction due to dispersive forces (*E*_disp_)^28,53–55^ completes the partition components for *E*_int_, so that:*E*_int_ = (*E*_Pauli_ + *E*_estat_) + *E*_orb_ + *E*_disp_ = *E*_steric_ + *E*_orb_ + *E*_disp_

The mathematical details of EDA and its applications,^47,48,56,57^ together with its strengths and weaknesses^46,58,59^ have been addressed at length in the literature. Notably, both the reactant distortion energies (*E*_prep_) and the overall attractive interactions (*E*_int_) between the two reacting molecules must be considered where *E*_tot_ = *E*_int_ + *E*_prep_. This “distortion-interaction” or “activation-strain” model is now an essential tool for reaction analysis.^60–63^ Furthermore, the NOCV method separates the components of *E*_orb_ and quantifies them in terms of the number of electrons “transferred” from one fragment to the other in the transition structure. These are expressed as eigenvalues for each bonding/antibonding NOCV pair, and pictorially as electron deformation densities, which illustrate the “electron flow” in the resultant interaction. Stabilization energies associated with each component can also be calculated.

EDA results are compiled in Table S1.† Factors determining the regioisomeric preference for each reaction requires comparison of observed (obs) *versus* non-observed (n/o) product pairs, shown as shaded/unshaded rows in Table S1.† For the monosubstituted oxyallyl cations 7 (R^2^ = Me) and 11 (R^2^ = H) (entries 1 and 2), the value of *E*_int_ is less favorable for the observed regioisomer, due principally to less favorable *E*_orb_, with *E*_steric_ almost the same. However, *E*_prep_ is significantly more favorable for the observed regioisomer, so that *E*_tot_ is favorable for that isomer. Consequently, the regiochemistry preference in these monosubstituted systems is determined not by steric repulsion or by favorable orbital interactions in the transition state, but by the relative magnitudes of distortion energy required to achieve the transition structure geometry for the reactants. A different story emerges for the disubstituted system 11 (R^2^ = Me) (entry 3), for which *E*_prep_ is essentially identical for each regioisomer. The more favorable *E*_int_ (and *E*_tot_) for the observed regioisomer (major diastereomer) is determined almost exclusively by the more favorable value of *E*_orb_.

Comparison of stereoisomeric pairs illustrates that in all cases *E*_prep_ and *E*_steric_ are more costly for the major than for the minor stereoisomer. However, these repulsive terms are outweighed by significantly more attractive *E*_orb_ and *E*_disp_ terms to afford more negative *E*_int_ (and *E*_tot_) for the major stereoisomer in each pair. It is noteworthy that the preferences for regiochemistry and stereochemistry are dominated by different components, illustrating the necessity of examining all factors contributing to transition structure energies.

Finally, the NOCV data shown in Table 4 for TS2 in the reaction between 7 (R^2^ = Me) and 8a provide information about the role of each reactant in the transition structure and the major electron redistribution contributions to *E*_orb_.

**Table 4:** NOCV contributions to *E*_orb_ in TS2 for the reaction of oxyallyl cation 7 (R^2^ = Me) with 8a

Bonding NOCV	Antibonding NOCV	NOCV eigenvalue	Deformation density^a^	Stabilization energy^b^ (kcal mol^−1^)
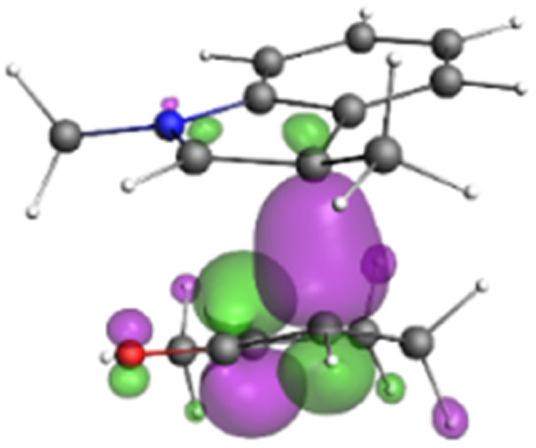	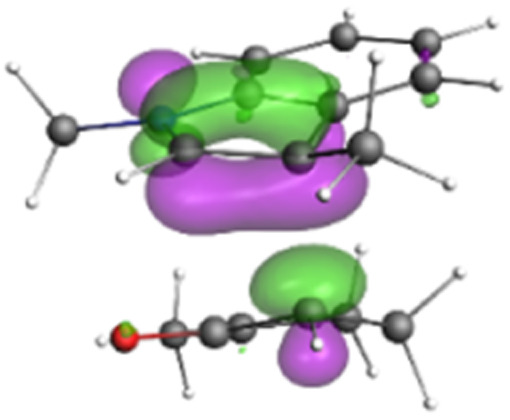	−0.914	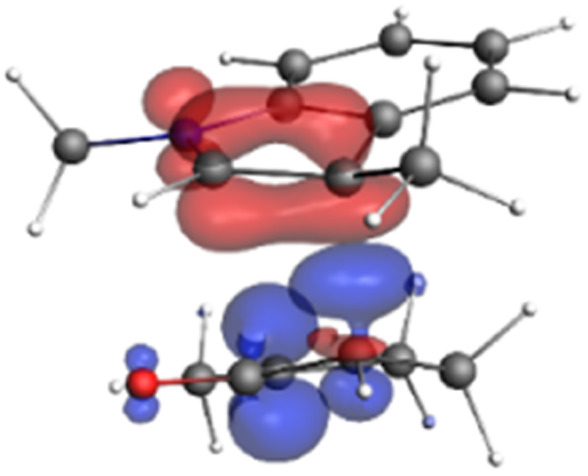	−52
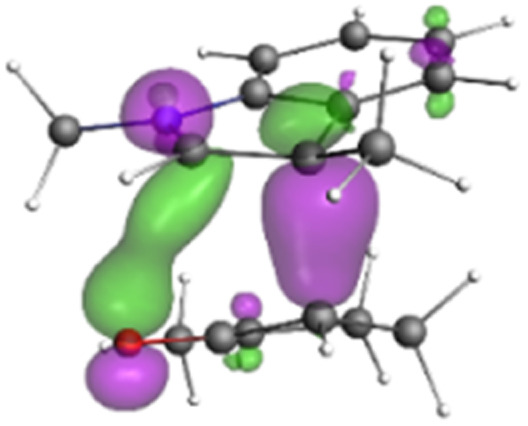	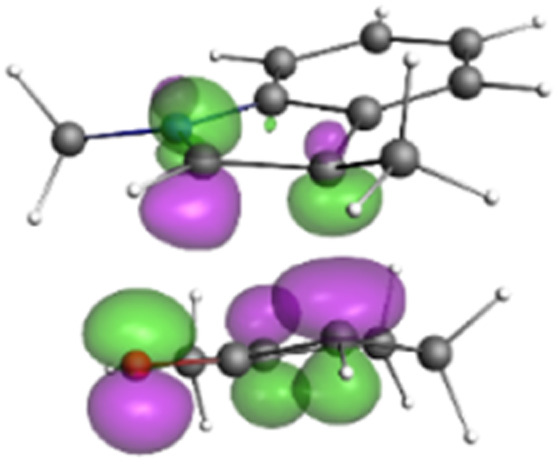	−0.479	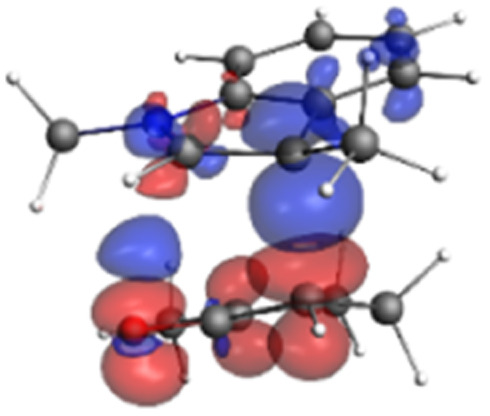	−15

a Deformation density is illustrative of electron “flow” from red regions to blue regions in the transition structure.

b Energy lowering resulting from each NOCV interaction. The sum of stabilization energies = *E*_orb_.

The two principal NOCV orbitals involve donation from the indole N–C–C π-orbital into the oxyallyl LUMO (53 kcal mol^−1^), and a smaller donation from the oxyallyl HOMO to the indole C–C π*-orbital (15 kcal mol^−1^). The former interaction is by far the dominant one, leading to formation of the first C–C σ-bond, and is consistent with the indole acting as the nucleophilic partner in the reaction. The small interaction of the O_lp_ with indole C2 is evident and contributes to TS2 being lower in energy (Fig. 1–3) than transition structures lacking this component.

## Conclusions

In conclusion, we have developed a new method for carrying out (3 + 2) annulation reactions between 3-substituted indoles and unsymmetrical oxyallyl cations in a regiodivergent fashion. This provides a means to access bicyclic indole compounds that contain stereodefined, vicinal quaternary centers. DFT studies establish that the regio- and diastereoselectivity of the reaction is kinetically controlled. Using EDC analysis, we found that the regioselectivity of the reactions is determined by reactant distortion energies (*E*_prep_) for oxyallyl cations 7 (R^2^ = Me) and 11 (R^2^ = H); whereas for 11 (R^2^ = Me), regioselectivity is governed almost exclusively by more favorable orbital mixing (*E*_orb_) and dispersive forces (*E*_disp_). NOCV studies are consistent with the indole acting as the nucleophilic partner in the annulation reaction.

## Data availability

The datasets supporting this article have been uploaded as part of the ESI.†

## Author contributions

Project conceptualization: Z. P., L. L. L., and J. W.; experimental design and investigation: Z. P., L. L. L., and R. P. H.; funding acquisition: J. W.; manuscript writing: J. W. and R. P. H.; manuscript editing: Z. P., L. L. L., J. W., and R. P. H.

## Conflicts of interest

There are no conflicts to declare.

## Supplementary Material

SC-014-D2SC06999G-s001

SC-014-D2SC06999G-s002
