# Supplementary material for: Regiodivergent (3 + 2) annulation reactions of oxyallyl cations
Source: Chem Sci. 2023 Apr 24;14(19):5196–203. doi: 10.1039/d2sc06999g (PMC10189855; doi:10.1039/d2sc06999g)
Supplement: SC-014-D2SC06999G-s001 [file SC-014-D2SC06999G-s001.pdf]

Electronic Supplementary Material (ESI) for Chemical Science.
This journal is © The Royal Society of Chemistry 2023

Table of Contents

I. GENERAL INFORMATION S1
II. EXPERIMENTAL PROCEDURES AND ANALYTICAL DATA S2
Synthesis of Starting Materials S2
Annulation Reactions with Monobrominated Ketones S7
Annulation Reactions with Dibrominated Ketones S12
Debromination of **15b** S17
III. DFT STUDIES S18
IV. X-RAY CRYSTALLOGRAPHIC DATA S70
V. NMR SPECTRA S113

General Information

^1H NMR data were recorded using a Bruker Avance III 500 MHz spectrometer (TBI probe) and a Bruker Avance III 600 MHz spectrometer (BBFO probe) with calibration of spectra to $CDCl_3$ (7.26 ppm). ^{13}C NMR data were recorded at 150 MHz using a Bruker Avance III 600 MHz spectrometer (BBFO probe) at ambient temperature (unless otherwise stated) and are expressed in ppm using solvent as the internal standard ($CDCl_3$ at 77.16 ppm). Two-dimensional NMR spectra, including COSY, HSQC, HMBC, and NOESY were recorded using a Bruker Avance III 600 MHz spectrometer (BBFO probe). Infrared spectra were recorded using a JASCO FT/IRM4100 Fourier transform infrared spectrometer. Electronic paramagnetic resonance spectra were recorded using an Elexsys E500 CW-EPR spectrometer. Chemical shift values (δ) are expressed in ppm downfield relative to internal standard (tetramethylsilane at 0 ppm). Multiplicities are indicated as s (singlet), d (doublet), t (triplet), q (quartet), m (multiplet), and br s (broad singlet). Coupling constants are reported in hertz (Hz). Analytical thin-layer chromatography (TLC) was performed on SiliCycle precoated TLC plates (silica gel 60 F254, 0.25 mm). Visualization was accomplished with UV light and/or with ceric ammonium molybdate (CAM) or $KMnO_4$ staining solutions. Flash column chromatography was performed using a Biotage Isolera system on Biotage SNAP Ultra columns (part nos. FSUL-0442-0010 and FSUL-0442-0025). High-resolution mass spectra were acquired from the Mass Spectrometry Laboratory of University of Illinois (Urbana-Champaign, IL).

Reagents were used as received without purification unless stated otherwise. Tetrahydrofuran, methylene chloride, and dimethylformamide were dried and purified by the solvent system using the glass contour solvent purification system (from Pure Process Technology, LLC) by passing the solvents through two drying columns. The room temperature in the laboratory was measured at 20.0 °C.

A. Synthesis of Substituted Indoles 8a–d:

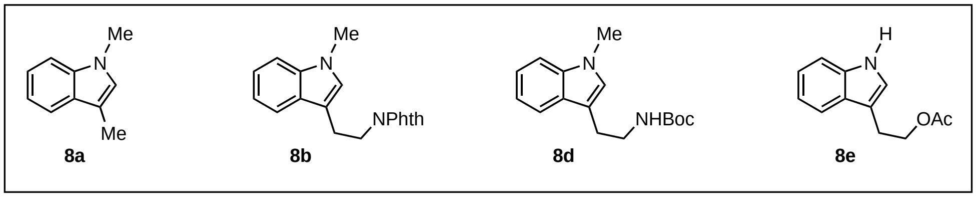

Compounds **8a-b**, **8d**[1] and **8e**[2] were synthesized according to known procedures.

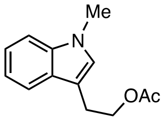

8c

Indole **8c** was prepared using a modified procedure previously reported in the literature.[3] To a flame dried 100 mL round bottom flask indole **8e** (1.24 g, 6.13 mmol, 1.0 equiv) was dissolved in 61.3 mL dry THF (0.1 M) under an atmosphere of nitrogen. The solution was then cooled to 0 °C and NaH (294 mg, 7.36 mmol, 1.2 equiv) was added in 4 portions over 20 minutes. MeI (0.401 mL, 6.44 mmol, 1.05 equiv) was added dropwise to the reaction mixture over 20 minutes and then was allowed to slowly warm to room temperature overnight. When the reaction was determined complete by thin-layer chromatographic analysis the reaction mixture was cooled back down to 0 °C and carefully diluted with water. The aqueous layer was extracted 3x with equal volume EtOAc. The combined organic layers were then extracted 3x 50% brine, 3x sat. brine, dried over anhydrous Na_2SO_4, and concentrated in vacuo. The residue was purified *via* silica gel flash column chromatography (hexanes/EtOAc) to afford the desired indole **8c** (1.05 g, 79%). The ^{1}H NMR spectrum of the product was consistent with what was reported in the literature.

B. Synthesis of Substituted Cyclopentanones 19b–d:

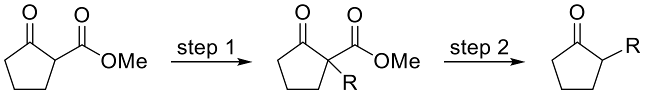

17 **18b–d** **19b–d**

General Procedure A:

Step 1: All alkylated compounds other than **18d** were prepared using a modified procedure previously reported in the literature.[4]

To a stirring mixture of K_2CO_3 (2.4 equiv) in acetone (0.2 M) was added methyl 2-oxocyclopentane carboxylate **17**. The reaction mixture was cooled to 0 °C then alkyl halide (1.2 equiv) dissolved in acetone was added slowly to the mixture at 0 °C. The reaction mixture was then warmed to room temperature and then heated to reflux. When the reaction was determined complete by thin-layer chromatographic analysis the reaction mixture was then cooled back to room temperature, filtered through a celite pad and washed with excess acetone. The filtrate was then concentrated, and the residue was purified *via* silica gel flash column chromatography (hexanes/EtOAc) to afford alkylated product **18**.

Step 2: All cyclopentanones other than **19d** were prepared using a modified procedure previously reported in the literature.[5]

---

[1] Touchette, S. J.; Dunkley, E. M.; Lowder, L. L.; Wu, J. *Chem. Sci.* **2019**, *10*, 7812.
[2] Jia, W.-L.; He, J.; Yang, J.-J.; Gao, X.-W.; Wu, L.-Z.; Liu, Q. *J. Org. Chem.* **2016**, *81*, 7172-7181.
[3] Leeson, P.D.; *J. Chem. Soc. Perkin Trans. 1,* **1984**, 2125-2128.
[4] Abraham et al. *Tetrahedron: Asymmetry*. **2011**, *22*, 69–100.
[5] Kato et al. *Chem. Pharm. Bull.* **1995**, 43, 2152-2158.

To a stirring mixture of **18** (1.0 equiv) in glacial acetic acid (0.9 M) was added 12.5% aqueous sulfuric acid dropwise at room temperature until a reaction concentration of 0.6 M was reached. The mixture was then heated to reflux. When the reaction was determined complete by thin-layer chromatographic analysis the reaction mixture was cooled to 0 °C. The mixture was then carefully neutralized with 1 M NaOH until a pH of ~7 was reached, then a small amount of saturated sodium bicarbonate was added. The neutral mixture was then extracted with EtOAc. The organic layers were combined and then washed with brine, dried over Na_2SO_4, and concentrated in vacuo. The crude oil of **19a**–**c** was used without further purification.

General Procedure B :

Step 1: Methyl 1-benzyl-2-oxocyclopentane-1-carboxylate was prepared using a modified procedure previously reported in the literature (**18d**).[6]

To a THF (0.1 M) solution of methyl 2-oxocyclopentane carboxylate (1.0 equiv, **17**) cooled at 0 °C, NaH (1.3 equiv, 60% dispersion in mineral oil) was slowly added. The reaction mixture was warmed to room temperature and allowed to stir at this temperature for 30 minutes. The reaction mixture was cooled back down to 0 °C and then benzyl chloride (1.2 equiv) was slowly added, and the mixture was then heated to reflux. When the reaction was determined complete by thin-layer chromatographic analysis the reaction mixture was cooled to 0 °C and quenched with distilled water. The organic layer was separated, and the aqueous layer was extracted with EtOAc. The organic layers were combined then washed with brine, dried over Na_2SO_4, and concentrated in vacuo. The residue was purified *via* silica gel flash column chromatography (hexanes/EtOAc) to afford alkylated product **18**.

Step 2:

To a stirring mixture of **18** (1.0 equiv) in glacial acetic acid (0.9 M) was added 12.5% aqueous sulfuric acid dropwise at room temperature until a reaction concentration of 0.6 M was reached. The mixture was then heated to reflux. When the reaction was determined complete by thin-layer chromatographic analysis the reaction mixture was cooled to 0 °C. The mixture was then carefully neutralized with 1 M NaOH until a pH of ~7 was reached then a small amount of saturated sodium bicarbonate was added. The neutral mixture was then extracted with EtOAc. The organic layers were combined and then washed with brine, dried over Na_2SO_4, and concentrated in vacuo. The crude oil of **19d** was used without further purification.

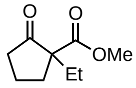

18b

Following General Procedure A, a mixture of **17** (8.52 g, 59.9 mmol, 1.0 equiv), bromoethane (5.23 mL, 70.56 mmol, 1.2 equiv), and K_2CO_3 (19.84 g, 140.8 mmol, 2.4 equiv) in acetone (293 mL, 0.2 M) afforded 1,3-ketoester **18b** as a colorless oil that was found to be homogeneous as judged by ^{1}H NMR spectroscopy (7.61 g, 76%). **^{1}H NMR** (600 MHz, $CDCl_3$) δ 3.70 (s, 3H), 2.56 – 2.47 (m, 1H), 2.45 – 2.35 (m, 1H), 2.30 – 2.19 (m, 1H), 2.05 – 1.84 (m, 4H), 1.64 (m, *J* = 7.4 Hz, 1H), 0.89 (t, *J* = 7.4 Hz, 3H). **^{13}C NMR** (150 MHz, $CDCl_3$) δ 215.35, 171.91, 61.31, 52.80, 38.45, 32.55, 27.25, 19.89, 9.59. **FTIR** (neat, cm^{-1}) 2962.13, 2875.34, 1736.58. **HRMS** (ESI) calcd. for C9H14O3 (m/z M+Na$^+$): 193.0841, found 193.0842.

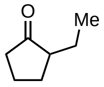

19b

Following General Procedure A, a mixture of **18b** (7.16 g, 44.7 mmol, 1.0 equiv), glacial acetic acid (24.9 mL, 0.4 M), and 12.5% aqueous sulfuric acid (21.7 mL, 0.2 M) afforded cyclopentanone **19b** as a pale yellow oil that was found to be homogeneous as judged by ^{1}H NMR spectroscopy (3.40 g, 68%). **^{1}H NMR** (600 MHz, $CDCl_3$) δ 2.34 – 2.24 (m, 1H), 2.24 – 2.15 (m, 1H), 2.14 – 2.04 (m, 1H), 2.03 – 1.94 (m, 2H), 1.82 – 1.71 (m, 2H), 1.58 – 1.47 (m, 1H), 1.37 – 1.26 (m, 1H), 0.93 (t, *J* = 0.9 Hz, 3H). **^{13}C NMR** (150 MHz, $CDCl_3$) δ 221.86, 50.90, 38.65, 29.33, 22.99, 21.03, 12.22. **FTIR** (neat, cm^{-1}) 2968.87, 2883.06, 1750.08, 1725.98. **HRMS** (EI) calcd. for C7H12O: 112.0888, found 112.0889.

[6] Orito et al. *J. Org. Chem.* **2006**, *71*, 5951–5958.

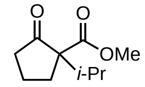

18c

Following General Procedure A, a mixture of **17** (4.26 g, 29.9 mmol, 1.0 equiv), 2-iodopropane (3.58 mL, 35.2 mmol, 1.2 equiv), and K_2CO_3 (19.84 g, 70.4 mmol, 2.4 equiv) in acetone (146 mL, 0.2 M) afforded 1,3-ketoester **18c** as a colorless oil that was found to be homogeneous as judged by ^{1}H NMR spectroscopy (2.22 g, 41%). **^{1}H NMR** (600 MHz, $CDCl_3$) δ 3.70 (s, 3H), 2.57 (hept, *J* = 6.9 Hz, 1H), 2.52 – 2.44 (m, 1H), 2.43 – 2.36 (m, 1H), 2.17 – 2.08 (m, 1H), 1.99 – 1.84 (m, 3H), 0.87 (d, *J* = 6.8 Hz, 3H), 0.83 (d, *J* = 6.9 Hz, 3H). **^{13}C NMR** (150 MHz, $CDCl_3$) δ 215.23, 171.16, 65.95, 52.93, 39.50, 32.54, 27.70, 20.02, 18.88, 18.16. **FTIR** (neat, cm^{-1}) 2965.02, 2879.20, 1749.12, 1720.19. **HRMS** (ESI) calcd. for C10H16O3 (m/z M+Na^{+}): 207.0997 found 207.0999.

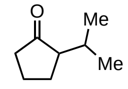

19c

Following General Procedure A, a mixture of **18c** (2.22 g, 12.0 mmol, 1.0 equiv), glacial acetic acid (13.4 mL, 0.4 M), and 12.5% aqueous sulfuric acid (6.7 mL, 0.2 M) afforded cyclopentanone **19c** as a pale yellow oil that was found to be homogeneous as judged by ^{1}H NMR spectroscopy (625 mg, 41%). **^{1}H NMR** (600 MHz, $CDCl_3$) δ 2.33 – 2.23 (m, 1H), 2.18 – 2.09 (m, 1H), 2.08 – 1.94 (m, 4H), 1.78 – 1.61 (m, 2H), 0.98 (d, *J* = 6.9 Hz, 3H), 0.81 (d, *J* = 6.8 Hz, 3H). **^{13}C NMR** (150 MHz, $CDCl_3$) δ 221.53, 55.41, 39.54, 27.70, 24.95, 21.45, 20.94, 18.72. **FTIR** (neat, cm^{-1}) 2960.20, 2874.38, 1734.66. **HRMS** (EI) calcd. for C8H14O: 126.1044, found 126.1043.

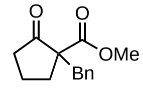

18d

Following General Procedure B, a mixture of **17** (8.52 g, 59.9 mmol, 1.0 equiv), NaH (3.11 g, 77.9 mmol, 1.3 equiv), and benzyl chloride (8.24 mL, 71.9 mmol, 1.2 equiv) in THF (60 mL, 0.1 M) afforded 1,3-ketoester **18d** as a colorless oil that was found to be homogeneous as judged by ^{1}H NMR spectroscopy (8.55 g, 61%). **^{1}H NMR** (600 MHz, $CDCl_3$) δ 7.28 – 7.19 (m, 3H), 7.15 – 7.10 (m, 2H), 3.73 (s, 3H), 3.21 (d, *J* = 13.7 Hz, 1H), 3.12 (d, *J* = 13.8 Hz, 1H), 2.46 – 2.33 (m, 2H), 2.09 – 2.00 (m, 1H), 2.00 – 1.84 (m, 2H), 1.64 – 1.55 (m, 2H). **^{13}C NMR** (150 MHz, $CDCl_3$) δ 215.19, 171.73, 136.86, 130.48, 128.75, 127.23, 61.85, 52.99, 39.50, 38.72, 32.04, 19.78. **FTIR** (neat, cm^{-1}) 3086.51, 3062.41, 3028.66, 2954.41, 2889.81, 1958.36, 1884.11, 1752.01, 1726.94. **HRMS** (ESI) calcd. for C14H16O3 (m/z M+Na^{+}): 255.0997 found 255.0998

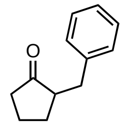

19d

Following General Procedure B, a mixture of **18d** (5.1 g, 21.9 mmol, 1.0 equiv), glacial acetic acid (24.3 mL, 0.4 M), and 12.5% aqueous sulfuric acid (10.6 mL, 0.2 M) afforded cyclopentanone **19d** as a colorless oil that was found to be homogeneous as judged by ^{1}H NMR spectroscopy (3.14 g, 82%). **^{1}H NMR** (600 MHz, $CDCl_3$) δ 7.31 – 7.24 (m, 2H), 7.23 – 7.14 (m, 3H), 3.15 (dd, *J* = 13.9, 4.2 Hz, 1H), 2.60 – 2.48 (m, 1H), 2.41 – 2.28 (m, 2H), 2.18 – 2.03 (m, 2H), 2.01 – 1.90 (m, 1H), 1.80 – 1.66 (m, 1H), 1.65 – 1.49 (m, 1H). **^{13}C NMR** (150 MHz, $CDCl_3$) δ 220.58, 140.35, 129.24, 128.76, 126.50, 51.38, 38.56, 35.94, 29.50, 20.89. **FTIR** (neat, cm^{-1}) 3084, 3061, 3026, 2962, 2876, 1736. **HRMS** (ESI) calcd. for C12H14O (m/z M+H^{+}): 175.1123, found 175.1121.

C. Preparation of Monobrominated Cyclopentanones 12a–e:

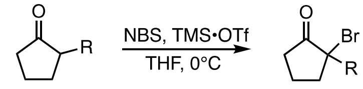

19 **12a–e**

General Procedure C:

The desired monobrominated cyclopentanones were prepared from the corresponding ketones using a procedure previously reported in the literature.[7] To a solution of cyclopentanone **19** (1.0 equiv) in THF (0.2 M) was added NBS (1.1 equiv) and was allowed to completely dissolve. The reaction mixture was then cooled to 0 °C and TMS•OTf (0.1 equiv) was added dropwise and allowed to stir at this temperature. When the reaction was determined complete by thin-layer chromatographic analysis (and by color change of the reaction mixture from a light yellow to a colorless solution) the reaction mixture was quenched with saturated aq. $NaHCO_3$ at 0 °C, filtered, and the residual solid was washed with EtOAc. The filtrate was extracted with EtOAc. The combined organic layers were then washed with brine, dried over Na_2SO_4, and concentrated in

[7] Guha, S.K.; Wu, B.; Kim, B.S.; Baik, W.; Koo, S. *Tetrahedron Lett*. **2006**, 47, 291-293.

vacuo. The residue was purified *via* silica gel flash column chromatography (hexanes/EtOAc) to afford the desired monobrominated product **12**.

Note* These monobrominated compounds readily decompose and were thus carried forward immediately to (3+2) dearomative annulation following purification.

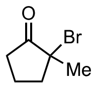

12a

Following General Procedure C, a mixture of commercially-available 2-methylcyclopentan-1-one (2.18 mL, 20.38 mmol, 1.0 equiv), NBS (3.98 g, 22.4 mmol, 1.1 equiv) and TMS•OTf (0.368 mL, 2.04 mmol, 0.10 equiv) in THF (102 mL, 0.2 M) afforded monobrominated cyclopentanone **12a** as a pale yellow oil that was found to be approximately 95% pure as judged by ^{1}H NMR spectroscopy (2.06 g, 57%). **^{1}H NMR** (600 MHz, $CDCl_3$) δ 2.56 – 2.47 (m, 1H), 2.46 – 2.40 (m, 1H), 2.17 – 2.02 (m, 2H), 1.99 – 1.92 (m, 1H), 1.90 – 1.82 (m, 1H), 1.75 (s, 3H).**^{13}C NMR** (150 MHz, $CDCl_3$) δ 210.60, 64.23, 41.27, 34.19, 24.78, 18.62.

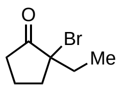

12b

Following General Procedure C, a mixture of 2-ethylcyclopentan-1-one **(19b)** (400 mg, 3.56 mmol, 1.0 equiv), NBS (698 mg, 3.92 mmol, 1.1 equiv) and TMS•OTf (0.064 mL, 0.356 mmol, 0.10 equiv) in THF (17.8 mL, 0.2 M) afforded monobrominated cyclopentanone **12b** as a pale yellow oil that was found to be approximately 95% pure as judged by ^{1}H NMR spectroscopy (196 mg, 29%). **^{1}H NMR** (600 MHz, $CDCl_3$) δ 2.61 – 2.54 (m, 1H), 2.41 – 2.34 (m, 1H), 2.22 – 2.03 (m, 3H), 2.03 – 1.97 (m, 1H), 1.93 – 1.85 (m, 1H), 1.78 – 1.70 (m, 1H), 1.08 (t, *J* = 7.4 Hz, 3H).**^{13}C NMR** (150 MHz, $CDCl_3$) δ 211.08, 70.84, 38.48, 35.38, 30.69, 19.00, 10.46.

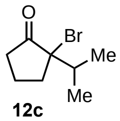

12c

Following General Procedure C, a mixture of 2-isopropylcyclopentan-1-one **(19c)** (352 mg, 2.79 mmol, 1.0 equiv), NBS (546 mg, 3.07 mmol, 1.1 equiv) and TMS•OTf (0.050 mL, 0.279 mmol, 0.10 equiv) in THF (14 mL, 0.2 M) afforded monobrominated cyclopentanone **12c** as a pale yellow oil that was found to be approximately 95% pure as judged by ^{1}H NMR spectroscopy (409 mg, 71%). **^{1}H NMR** (600 MHz, $CDCl_3$) δ 2.61 – 2.52 (m, 1H), 2.37 (hept, *J* = 6.8 Hz, 1H), 2.27 – 2.19 (m, 1H), 2.17 – 2.08 (m, 1H), 2.07 – 1.95 (m, 3H), 1.23 (d, *J* = 6.7 Hz, 3H), 0.91 (d, *J* = 6.8 Hz, 3H). **^{13}C NMR** (150 MHz, $CDCl_3$) δ 211.09, 75.87, 36.47, 34.44, 34.17, 19.40, 18.81, 18.49.

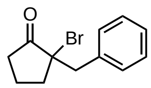

12d

Following General Procedure C, a mixture of 2-benzylcyclopentan-1-one **(19d)** (500 mg, 2.87 mmol, 1.0 equiv), NBS (561 mg, 3.15 mmol, 1.1 equiv) and TMS•OTf (0.052 mL, 0.287 mmol, 0.10 equiv) in THF (14.3 mL, 0.2 M) afforded monobrominated cyclopentanone **12d** as a pale yellow oil that was found to be approximately 95% pure as judged by ^{1}H NMR spectroscopy (440 mg, 60%). **^{1}H NMR** (600 MHz, $CDCl_3$) δ 7.32 – 7.24 (m, 3H), 7.24 – 7.20 (m, 2H), 3.45 – 3.36 (m, 2H), 2.55 (dd, *J* = 19.1, 17.7 Hz, 1H), 2.23 (dd, *J* = 14.5, 2.1 Hz, 1H), 2.16 – 2.07 (m, 1H), 2.05 – 1.86 (m, 3H). **^{13}C NMR** (150 MHz, $CDCl_3$) δ 210.68, 136.56, 130.91, 128.75, 127.48, 68.87, 43.37, 37.79, 35.59, 18.94.

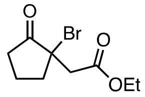

12e

Following General Procedure C, a mixture of commercially-available ethyl 2-(2-oxocyclopentyl)acetate (600 mg, 3.52 mmol, 1.0 equiv), NBS (690 mg, 3.86 mmol, 1.1 equiv) and TMS•OTf (0.069 mL, 0.352 mmol, 0.10 equiv) in THF (16 mL, 0.2 M) afforded monobrominated cyclopentanone **12e** as a pale yellow oil that was found to be approximately 90% pure as judged by ^{1}H NMR spectroscopy (347 mg, 40%). **^{1}H NMR** (600 MHz, $CDCl_3$) δ 4.14 (q, *J* = 6.9 Hz, 2H), 3.23 (s, 2H), 2.55 (dd, *J* = 10.8, 8.3 Hz, 1H), 2.50 – 2.44 (m, 1H), 2.41 – 2.33 (m, 1H), 2.30 – 2.22 (m, 1H), 2.21 – 2.11 (m, 1H), 2.10 – 2.03 (m, 1H), 1.26 (t, *J* = 7.1 Hz, 3H). **^{13}C NMR** (150 MHz, $CDCl_3$) δ 210.10, 169.75, 62.57, 61.40, 42.48, 38.57, 34.94, 19.21, 14.43.

D. Synthesis of Dibrominated Cyclopentanones 14a–b, d–f:

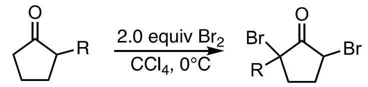

19 **14a–b, d–f**

General Procedure D:

The desired dibrominated cyclopentanones were prepared from the corresponding ketones using a procedure previously reported in the literature.[8-9] Cyclopentanone **19** (1.0 equiv) was dissolved in CCl_4 (1.0 M) and then cooled to 0 °C. Bromine (2.0 equiv) was then added dropwise and allowed to stir at this temperature. When the reaction was determined complete by thin-layer chromatographic analysis (and by color change of the reaction mixture from dark red to a near-colorless solution) the reaction mixture was quenched with saturated aq. $Na_2S_2O_3$ at 0 °C. The mixture was extracted with DCM and the combined organic layers were then washed with brine, dried over Na_2SO_4, and concentrated in vacuo. The crude oil was used without further purification to furnish dibrominated product **14**.

Note* These dibrominated compounds readily decompose and were thus carried forward immediately to the (3+2) dearomative annulation following purification.

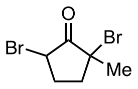

14a

Following General Procedure D, a mixture of 2-methylcyclopentan-1-one (0.200 mL, 2.03 mmol, 1.0 equiv) and bromine (0.208 mL, 4.06 mmol, 2.0 equiv) in CCl_4 (2.0 mL, 1.0 M) afforded dibrominated cyclopentanone **14a** as a pale yellow oil that was found to be approximately 85% pure as judged by ^{1}H NMR spectroscopy (510 mg, 98%). **^{1}H NMR** (600 MHz, $CDCl_3$) δ 4.53 (d, *J* = 6.4 Hz, 1H), 2.77 – 2.67 (m, 1H), 2.52 – 2.45 (m, 1H), 2.42 – 2.34 (m, 1H), 2.28 – 2.22 (m, 1H), 1.95 (s, 3H). **^{13}C NMR** (150 MHz, $CDCl_3$) δ δ 204.21, 62.39, 45.30, 44.21, 39.10, 39.09, 39.07, 31.07, 27.72, 26.94, 26.92.

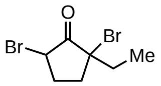

14b

Following General Procedure D, a mixture of 2-ethylcyclopentan-1-one **(19b)** (0.200 mL, 2.03 mmol, 1.0 equiv) and bromine (0.208 mL, 4.06 mmol, 2.0 equiv) in CCl_4 (2.0 mL, 1.0 M) afforded dibrominated cyclopentanone **14b** as a pale yellow oil that was found to be homogeneous as judged by ^{1}H NMR spectroscopy (510 mg, 98%). **^{1}H NMR** (600 MHz, $CDCl_3$) δ 4.51 (d, *J* = 6.6 Hz, 1H), 2.76 – 2.64 (m, 1H), 2.43 – 2.31 (m, 2H), 2.31 – 2.21 (m, 2H), 2.00 – 1.88 (m, 1H), 1.12 (t, *J* = 7.53, 2.4 Hz, 3H). **^{13}C NMR** (151 MHz, $CDCl_3$) δ 204.14, 68.71, 45.79, 35.89, 32.05, 31.04, 10.54.

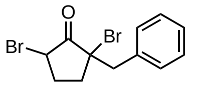

14d

Following General Procedure D, a mixture of 2-benzylcyclopentan-1-one **(19d)** (0.300 mL, 1.72 mmol, 1.0 equiv) and bromine (0.176 mL, 3.44 mmol, 2.0 equiv) in CCl_4 (1.72 mL, 1.0 M) afforded dibrominated cyclopentanone **14d** as a pale yellow oil that was found to be approximately 90% pure as judged by ^{1}H NMR spectroscopy (488 mg, 86%). **^{1}H NMR** (600 MHz, $CDCl_3$) δ 7.35 – 7.27 (m, 4H), 7.25 – 7.22 (m, 1H), 4.49 (d, *J* = 6.3 Hz, 1H), 3.48 (s, 2H), 2.69 (ddd, *J* = 14.6, 12.0, 6.1 Hz, 1H), 2.44 (ddd, *J* = 14.7, 12.0, 6.2 Hz, 1H), 2.22 (dd, *J* = 14.8, 6.2 Hz, 1H), 2.18 – 2.12 (m, 1H). **^{13}C NMR** (150 MHz, $CDCl_3$) δ 203.49, 135.62, 131.06, 128.81, 127.78, 67.29, 45.40, 44.22, 35.34, 31.01.

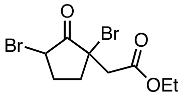

14e

Following General Procedure D, a mixture of ethyl 2-(2-oxocyclopentyl)acetate (400 mg, 2.35 mmol, 1.0 equiv) and bromine (0.240 mL, 4.70 mmol, 2.0 equiv)* in CCl_4 (2.4 mL, 1.0 M) afforded dibrominated cyclopentanone **12e** as a pale yellow oil that was found to be an inseparable 5:3 mixture of what we believe to be diastereomers of the desired product (696 mg, 90% combined). **^{1}H NMR** (600 MHz, $CDCl_3$) see spectrum **^{13}C NMR** (150 MHz, $CDCl_3$) see spectrum.

*2.1 equiv of bromine was used instead of 2.0 equiv as described in general procedure D

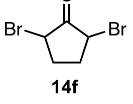

14f

Dibromocyclopentanone **14f** was prepared according to literature procedure[10] (1.2 g, 14%). The ^{1}H NMR spectrum of the product was consistent with what was reported in the literature.

---

[8] Qiao, J.-B.; Zhao, Y.-M.; Gu, P. M. Org. Lett. **2016**, 18, 1984– 1987,
[9] Fry, A. J.; O'Dea, J. J. *J. Org. Chem.* **1975**, *40*, 3625– 3631.

[10] Folkins, P. L.; Harpp, D. N. *J. Org. Chem.***1992**, *57*, 2013.

E. Annulation Reactions with Monobrominated Ketones:

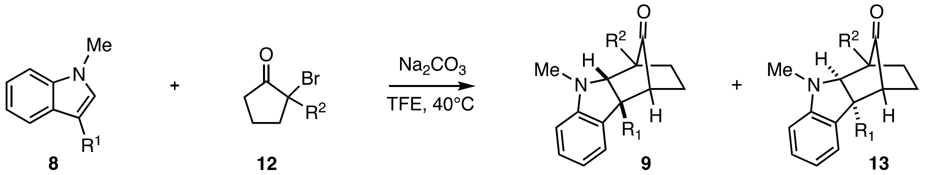

General Procedure E:

To a solution of indole **8** (1.0 equiv) in trifluoroethanol (0.2 M) was sequentially added sodium carbonate (3.0 equiv) and α-bromocyclopentanone **12** (1.3 equiv). The reaction mixture was stirred at 40 °C until the reaction was judged complete as determined by thin-layer chromatographic analysis and then cooled to room temperature. The mixture was filtered through a short pad of silica gel and washed with EtOAc The filtrate was concentrated and the residue was purified via silica gel flash column chromatography (hexanes/EtOAc) to afford ketone **9** as the major product and diastereomer **13** as the minor product.

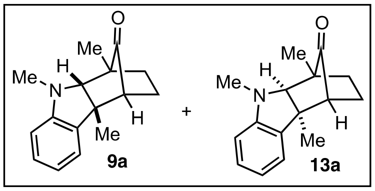

Following General Procedure E, a mixture of indole (100 mg, 0.689 mmol, 1.0 equiv), sodium carbonate (219 mg, 2.06 mmol, 3.0 equiv), and cyclopentanone **12a** (158 mg, 0.895 mmol, 1.3 equiv) in TFE (3.4 mL, 0.2 M) afforded ketone **9a** as a colorless solid that was found to be homogeneous as judged by ^{1}H NMR spectroscopy (133 mg, 80%). **^{1}H NMR** (600 MHz, $CDCl_3$) δ 7.11 (t, *J* = 7.7 Hz, 1H), 6.88 (d, *J* = 7.3 Hz, 1H), 6.62 (t, *J* = 7.3 Hz, 1H), 6.39 (d, *J* = 7.8 Hz, 1H), 3.20 (s, 1H), 2.86 (s, 3H), 2.00 (d, *J* = 4.9 Hz, 1H), 1.91 (td, *J* = 11.7, 4.6 Hz, 1H), 1.68 – 1.59 (m, 1H), 1.36 – 1.25 (m, 5H), 1.17 (s, 3H). **^{13}C NMR** (150 MHz, $CDCl_3$) δ 215.92, 154.05, 132.08, 128.79, 124.43, 117.13, 105.62, 77.86, 50.22, 48.33, 48.03, 35.07, 28.28, 23.62, 20.04, 15.02. **FTIR** (film, cm^{-1}) 3024.80, 2951.52, 2921.63, 2863.77, 1765.51, 1607.38. **HRMS** (ESI) calcd. for C16H19NO (m/z $M+H^+$): 242.1545, found 242.1549.

Ketone **13a** was also isolated as a colorless solid that was found to be homogeneous as judged by ^{1}H NMR spectroscopy (5.20 mg, 3%).**^{1}H NMR** (600 MHz, $CDCl_3$) δ 7.03 (t, *J* = 7.7 Hz, 1H), 6.88 (d, *J* = 7.3 Hz, 1H), 6.61 (t, *J* = 7.3 Hz, 1H), 6.32 (d, *J* = 7.9 Hz, 1H), 3.00 (s, 1H), 2.87 (s, 3H), 2.09 (d, *J* = 3.9 Hz, 1H), 2.01 – 1.93 (m, 1H), 1.82 – 1.74 (m, 1H), 1.70 – 1.61 (m, 2H), 1.45 (s, 3H), 1.08 (s, 3H). **^{13}C NMR** (150 MHz, $CDCl_3$) δ 215.66, 151.80, 135.12, 128.84, 123.52, 118.02, 107.20, 80.61, 50.02, 49.41, 47.99, 37.50, 28.14, 25.25, 18.73, 12.10. **FTIR** (film, cm^{-1}) 3013.23, 2970.80, 2926.45, 2873.42, 1762.62, 1603.52. **HRMS** (ESI) calcd. for C16H19NO (m/z $M+H^+$): 242.1545, found 242.1544.

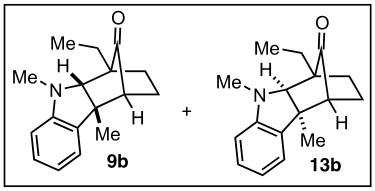

Following General Procedure E, a mixture of indole (100 mg, 0.689 mmol, 1.0 equiv), sodium carbonate (219 mg, 2.06 mmol, 3.0 equiv), and cyclopentanone **12b** (171 mg, 0.895 mmol, 1.3 equiv) in TFE (3.4 mL, 0.2 M) afforded ketone **9b** as a colorless solid that was found to be homogeneous as judged by ^{1}H NMR spectroscopy (141 mg, 80%). **^{1}H NMR** (600 MHz, $CDCl_3$) δ 7.12 (t, *J* = 7.7 Hz, 1H), 6.88 (d, *J* = 7.2 Hz, 1H), 6.64 (t, *J* = 7.3 Hz, 1H), 6.41 (d, *J* = 7.9 Hz, 1H), 3.35 (s, 1H), 2.85 (s, 3H), 1.96 (d, *J* = 4.9 Hz, 1H), 1.84 (td, *J* = 11.2, 4.6 Hz, 1H), 1.71 – 1.58 (m, 3H), 1.43 (td, *J* = 12.2, 2.0 Hz, 1H), 1.33 – 1.25 (m, 4H), 1.03 (t, *J* = 7.6 Hz, 3H). **^{13}C NMR** (150 MHz, $CDCl_3$) δ 215.71, 154.34, 132.23, 128.75, 124.43, 117.37, 106.14, 75.26, 51.82, 50.55, 48.11, 35.69, 28.26, 22.14, 21.37, 19.85, 9.57. **FTIR** (film, cm^{-1}) 3021.91, 2962.13, 2878.24, 2820.38, 1760.69, 1605.45. **HRMS** (ESI) calcd. for C17H21NO (m/z $M+H^+$): 256.1701, found 256.1703.

Ketone **13b** was also isolated as a colorless solid that was found to be homogeneous as judged by ^{1}H NMR spectroscopy (9.90 mg, 6%).**^{1}H NMR** (600 MHz, $CDCl_3$) δ 7.04 (t, *J* = 7.7 Hz, 1H), 6.89 (d, *J* = 7.3 Hz, 1H), 6.62 (t, *J* = 7.3 Hz, 1H), 6.35 (d, *J* = 7.9 Hz, 1H), 3.08 (s, 1H), 2.88 (s, 3H), 2.07 (d, *J* = 3.4 Hz, 1H), 2.00 – 1.95 (m, 1H), 1.78 – 1.73 (m, 2H), 1.68 – 1.52 (m, 3H), 1.45 (s, 3H), 0.96 (t, *J* = 7.6 Hz, 3H).**^{13}C NMR** (150 MHz, $CDCl_3$) δ 215.45, 152.13, 135.20, 128.78, 123.47, 118.20, 107.57, 78.93, 53.14, 50.45, 47.82, 37.86,

25.34, 24.47, 18.62, 18.55, 9.32. **FTIR** (film, cm^{-1}) 3048.91, 2962.13, 2877.27, 2817.49, 1767.44, 1604.48. **HRMS** (ESI) calcd. for C17H21NO (m/z $M+H^+$): 256.1701, found 256.1701.

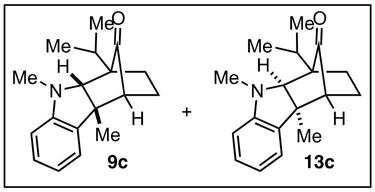

Following General Procedure E, a mixture of indole (100 mg, 0.689 mmol, 1.0 equiv), sodium carbonate (219 mg, 2.06 mmol, 3.0 equiv), and cyclopentanone **12c** (183 mg, 0.895 mmol, 1.3 equiv) in TFE (3.4 mL, 0.2 M) afforded ketone **9c** as a colorless solid that was found to be homogeneous as judged by ^{1}H NMR spectroscopy (110 mg, 59%). **^{1}H NMR** (600 MHz, $CDCl_3$) δ 7.13 (t, *J* = 7.7 Hz, 1H), 6.89 (d, *J* = 7.3 Hz, 1H), 6.68 (t, *J* = 7.3 Hz, 1H), 6.49 (d, *J* = 7.9 Hz, 1H), 3.50 (s, 1H), 2.87 (s, 3H), 2.00 – 1.91 (m, 3H), 1.63 – 1.54 (m, 1H), 1.45 (td, *J* = 12.1, 2.1 Hz, 1H), 1.30 (s, 3H), 1.24 (td, *J* = 11.2, 4.6 Hz, 2H), 1.10 – 1.04 (apparent triplet 6H). **^{13}C NMR** (150 MHz, $CDCl_3$) δ 215.56, 155.19, 132.61, 128.70, 124.33, 118.05, 107.67, 73.66, 55.02, 50.57, 48.43, 37.72, 28.32, 27.28, 21.38, 19.72, 19.20, 19.09. **FTIR** (film, cm^{-1}) 3049.87, 2960.20, 2876.31, 2815.56, 1760.69, 1605.45. **HRMS** (ESI) calcd. for C18H23NO (m/z $M+H^+$): 270.1858, found 270.1856.

Ketone **13c** was also isolated as a colorless solid that was found to be homogeneous as judged by ^{1}H NMR spectroscopy (19.4 mg, 10%).**^{1}H NMR** (600 MHz, $CDCl_3$) δ 7.04 (t, *J* = 7.7 Hz, 1H), 6.89 (d, *J* = 7.3 Hz, 1H), 6.64 (t, *J* = 7.4 Hz, 1H), 6.39 (d, *J* = 7.9 Hz, 1H), 3.10 (s, 1H), 2.90 (s, 3H), 2.23 (hept, *J* = 6.8 Hz, 1H), 2.02 – 1.88 (m, 3H), 1.75 – 1.67 (m, 1H), 1.45 (s, 3H), 1.43 – 1.35 (m, 1H), 1.01 (d, *J* = 6.7 Hz, 3H), 0.94 (d, *J* = 6.9 Hz, 3H). **^{13}C NMR** (150 MHz, $CDCl_3$) δ 214.53, 152.71, 135.29, 128.71, 123.44, 118.52, 108.18, 79.14, 56.30, 51.02, 47.45, 38.82, 25.49, 23.49, 20.38, 19.33, 18.54, 16.94. **FTIR** (film, cm^{-1}) 3049.87, 2959.23, 2927.41, 2873.42, 2817.49, 1765.51, 1604.48. **HRMS** (ESI) calcd. for C18H23NO (m/z $M+H^+$): 270.1858, found 270.1855.

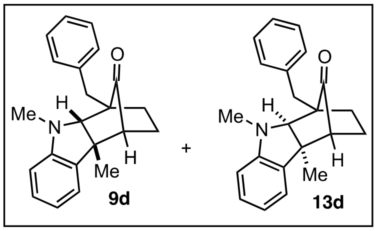

Following General Procedure E, a mixture of indole (100 mg, 0.689 mmol, 1.0 equiv), sodium carbonate (219 mg, 2.06 mmol, 3.0 equiv), and cyclopentanone **12d** (226 mg, 0.895 mmol, 1.3 equiv) in TFE (3.4 mL, 0.2 M) afforded ketone **9d** as a colorless solid that was found to be homogeneous as judged by ^{1}H NMR spectroscopy (164 mg, 75%). **^{1}H NMR** (600 MHz, $CDCl_3$) δ 7.36 – 7.27 (m, 4H), 7.24 – 7.19 (m, 1H), 7.10 (t, *J* = 7.7 Hz, 1H), 6.87 (d, *J* = 7.3 Hz, 1H), 6.63 (t, *J* = 7.3 Hz, 1H), 6.39 (d, *J* = 7.9 Hz, 1H), 3.25 (s, 1H), 3.02 (d, *J* = 14.4 Hz, 1H), 2.94 (d, *J* = 14.4 Hz, 1H), 2.66 (s, 3H), 2.01 (d, *J* = 5.0 Hz, 1H), 1.97 (td, *J* = 11.7, 4.2 Hz, 1H), 1.61 (tt, *J* = 12.6, 4.7 Hz, 1H), 1.41 (td, *J* = 12.1, 1.9 Hz, 1H), 1.29 (td, *J* = 12.1, 5.0 Hz, 1H), 1.26 (s, 3H). **^{13}C NMR** (150 MHz, $CDCl_3$) δ 215.28, 154.44, 138.78, 131.89, 130.66, 128.74, 128.67, 126.63, 124.23, 117.45, 106.32, 75.17, 53.15, 50.36, 48.38, 35.84, 35.68, 27.73, 22.48, 19.81. **FTIR** (film, cm^{-1}) 3056.62, 3026.73, 2951.52, 2872.45, 2360.44, 2340.19, 1763.58, 1605.45, 1558.20, 1540.85. **HRMS** (ESI) calcd. for C22H23NO (m/z $M+H^+$): 318.1858, found 318.1858.

Ketone **13d** was also isolated as a colorless solid that was found to be approximately 95% pure as judged by ^{1}H NMR spectroscopy (21 mg, 9%).**^{1}H NMR** (600 MHz, $CDCl_3$) δ 7.32 – 7.27 (m, 4H), 7.22 – 7.16 (m, 1H), 7.08 (t, *J* = 7.7 Hz, 1H), 6.91 (d, *J* = 7.3 Hz, 1H), 6.67 (t, *J* = 7.4 Hz, 1H), 6.43 (d, *J* = 7.9 Hz, 1H), 3.21 – 3.12 (m, 2H), 2.91 (s, 3H), 2.80 (d, *J* = 14.7 Hz, 1H), 2.10 (d, *J* = 3.6 Hz, 1H), 1.99 – 1.89 (m, 1H), 1.75 – 1.60 (m, 2H), 1.55 – 1.49 (m, 1H), 1.46 (s, 3H). **^{13}C NMR** (150 MHz, $CDCl_3$) δ 214.30, 152.30, 139.01, 135.33, 130.52, 128.90, 128.57, 126.36, 123.52, 118.70, 108.29, 80.90, 53.59, 50.31, 48.25, 38.96, 32.48, 25.39, 24.64, 18.70. **FTIR** (film, cm^{-1}) 3061.44, 2970.80, 2924.52, 2881.13, 1759.73, 1602.56. **HRMS** (ESI) calcd. for C22H23NO (m/z $M+H^+$): 318.1858, found 318.1859.

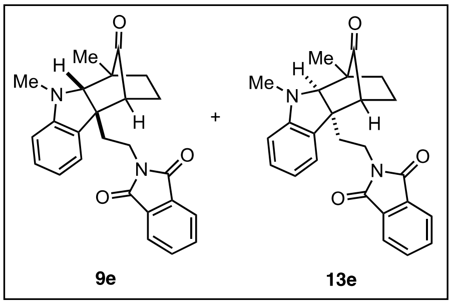

Following General Procedure E, a mixture of indole (100 mg, 0.329 mmol, 1.0 equiv), sodium carbonate (104 mg, 0.986 mmol, 3.0 equiv), and cyclopentanone **12a** (77 mg, 0.427 mmol, 1.3 equiv) in TFE (1.6 mL, 0.2 M) afforded ketone **9e** as a yellow oil that was found to be homogeneous as judged by ^{1}H NMR spectroscopy (79.7 mg, 60%). **^{1}H NMR** (600 MHz, $CDCl_3$) δ 7.70 (dd, *J* = 5.4, 3.0 Hz, 2H), 7.63 (dd, *J* = 5.4, 3.0 Hz, 2H), 6.89 – 6.82 (m, 2H), 6.41 (t, *J* = 7.4 Hz, 1H), 6.24 (d, *J* = 7.8 Hz, 1H), 3.60 (s, 1H), 3.58 – 3.47 (m, 2H), 2.90 (s, 3H), 2.40 (td, *J* = 8.6, 5.3 Hz, 1H), 1.99 (d, *J* = 4.8 Hz, 1H), 1.93 – 1.84 (m, 1H), 1.65 – 1.52 (m, 3H), 1.36 – 1.28 (m, 2H), 1.20 (s, 3H). **^{13}C NMR** (150 MHz, $CDCl_3$) δ 215.58, 168.25, 154.04, 133.97, 132.35, 128.89, 128.39, 124.65, 123.28, 117.52, 105.92, 105.90, 73.78, 51.08, 50.31, 47.93, 36.90, 34.94, 33.83, 23.80, 19.53, 15.04. **FTIR** (film, cm^{-1}) 3054.69, 2943.80, 2870.52, 1765.51, 1709.59, 1605.45, 1492.63. **HRMS** (ESI) calcd. for C25H24N2O3 (m/z M+H$^+$): 401.1865, found 401.1867.

Ketone **13e** was also isolated as a yellow oil that was found to be approximately 95% pure as judged by ^{1}H NMR spectroscopy (21 mg, 9%).**^{1}H NMR** (600 MHz, $CDCl_3$) δ 7.79 (dd, *J* = 5.4, 3.0 Hz, 2H), 7.70 (dd, *J* = 5.4, 5.3 Hz, 3H), 7.02 – 6.96 (m, 2H), 6.60 (t, *J* = 7.4 Hz, 1H), 6.29 (d, *J* = 8.2 Hz, 1H), 3.57 (td, *J* = 10.9, 4.2 Hz, 1H), 3.43 (ddd, *J* = 13.8, 10.2, 6.4 Hz, 1H), 3.29 (s, 1H), 2.90 (s, 3H), 2.26 (ddd, *J* = 13.3, 10.9, 6.4 Hz, 1H), 2.18 (d, *J* = 3.9 Hz, 1H), 2.06 – 1.94 (m, 3H), 1.83 – 1.73 (m, 1H), 1.73 – 1.65 (m, 2H), 1.09 (s, 3H). **^{13}C NMR** (150 MHz, $CDCl_3$) δ 214.68, 168.41, 152.26, 134.25, 132.42, 130.80, 129.32, 124.25, 123.50, 123.47, 123.27, 118.37, 107.36, 78.06, 50.58, 49.85, 48.95, 36.97, 35.12, 34.27, 28.26, 18.26, 11.85. **FTIR** (film, cm^{-1}) 3407.60, 3055.66, 2929.34, 2870.52, 1769.37, 1713.44, 1604.48, 1491.67. **HRMS** (ESI) calcd. for C25H21N2O3 (m/z M+H$^+$): 401.1865, found 401.1863.

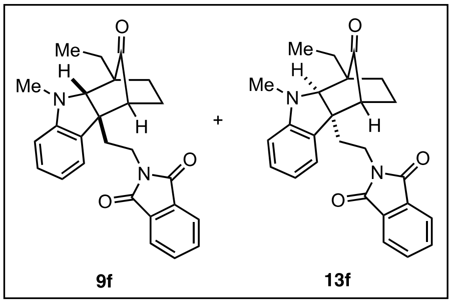

Following General Procedure E, a mixture of indole (100 mg, 0.329 mmol, 1.0 equiv), sodium carbonate (104 mg, 0.986 mmol, 3.0 equiv), and cyclopentanone **12b** (81 mg, 0.427 mmol, 1.3 equiv) in TFE (1.6 mL, 0.2 M) afforded ketone **9f** as a yellow oil that was found to be homogeneous as judged by ^{1}H NMR spectroscopy (105 mg, 77%). **^{1}H NMR** (600 MHz, $CDCl_3$) δ 7.70 (dd, *J* = 5.4, 3.1 Hz, 2H), 7.64 (dd, *J* = 5.5, 3.0 Hz, 2H), 6.91 – 6.82 (m, 2H), 6.43 (t, *J* = 7.4 Hz, 1H), 6.27 (d, *J* = 7.8 Hz, 1H), 3.76 (s, 1H), 3.58 – 3.46 (m, 2H), 2.89 (s, 3H), 2.37 (dt, *J* = 8.6, 5.5 Hz, 1H), 1.95 (d, *J* = 4.8 Hz, 1H), 1.81 (td, *J* = 7.2, 5.0 Hz, 1H), 1.76 – 1.54 (m, 5H), 1.44 (td, *J* = 12.2, 1.9 Hz, 1H), 1.32 (td, *J* = 11.2, 4.5 Hz, 1H), 1.06 (t, *J* = 7.5 Hz, 3H). **^{13}C NMR** (150 MHz, $CDCl_3$) δ 215.58, 168.24, 154.60, 133.97, 132.37, 128.83, 128.36, 124.64, 123.28, 117.47, 106.11, 71.23, 51.67, 50.86, 50.65, 36.86, 35.24, 33.84, 22.19, 21.49, 19.37, 9.53. **FTIR** (film, cm^{-1}) 3054.69, 2963.09, 2926.45, 2877.27, 2796.28, 1763.58, 1711.51, 1605.45. **HRMS** (ESI) calcd. for C26H26N2O3 (m/z M+H$^+$): 415.2022, found 415.2014.

Ketone **13f** was also isolated as a yellow oil that was found to be homogeneous as judged by ^{1}H NMR spectroscopy (15 mg, 11%).**^{1}H NMR** (600 MHz, $CDCl_3$) δ 7.79 (dd, *J* = 5.4, 3.0 Hz, 2H), 7.69 (dd, *J* = 5.5, 3.0 Hz, 2H), 7.02 – 6.96 (m, 2H), 6.60 (t, *J* = 7.4 Hz, 1H), 6.32 (d, *J* = 8.1 Hz, 1H), 3.58 (td, *J* = 10.9, 4.3 Hz, 1H), 3.43 (ddd, *J* = 13.9, 10.3, 6.5 Hz, 1H), 3.36 (s, 1H), 2.92 (s, 3H), 2.27 (ddd, *J* = 13.2, 10.9, 6.4 Hz, 1H), 2.16 (d, *J* = 3.5 Hz, 1H), 2.04 – 1.95 (m, 2H), 1.82 – 1.69 (m, 3H), 1.68 – 1.51 (m, 6H), , 0.98 (t, *J* = 7.6 Hz, 4H). **^{13}C NMR** (150 MHz, $CDCl_3$) δ 214.50, 168.43, 152.59, 134.26, 132.43, 130.87, 129.26, 124.20, 123.47, 118.54, 107.74, 76.48, 52.66, 50.48, 50.33, 37.36, 35.11, 34.31, 24.60, 18.41, 18.09, 9.34. **FTIR** (film, cm^{-1}) 3054.69, 2964.05, 2937.06, 2879.20, 2822.31, 1766.48, 1710.55, 1603.52. **HRMS** (ESI) calcd. for C26H26N2O3 (m/z M+H$^+$): 415.2022, found 415.2016.

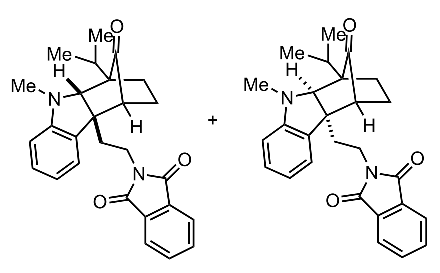

9g **13g**

Following General Procedure E, a mixture of indole (100 mg, 0.329 mmol, 1.0 equiv), sodium carbonate (104 mg, 0.986 mmol, 3.0 equiv), and cyclopentanone **12c** (87 mg, 0.427 mmol, 1.3 equiv) in TFE (1.6 mL, 0.2 M) afforded ketone **9g** as a yellow oil that was found to be homogeneous as judged by ^{1}H NMR spectroscopy (106 mg, 75%). **^{1}H NMR** (600 MHz, $CDCl_3$) δ 7.72 (dd, *J* = 5.4, 3.1 Hz, 2H), 7.64 (dd, *J* = 5.5, 3.0 Hz, 2H), 6.93 (t, *J* = 1.3 Hz, 1H), 6.87 (d, *J* = 1.3 Hz, 1H), 6.49 (t, *J* = 1.0 Hz, 1H), 6.36 (d, *J* = 7.9 Hz, 1H), 3.94 (s, 1H), 3.55 – 3.45 (m, 2H), 2.92 (s, 3H), 2.34 (dt, *J* = 14.0, 5.1 Hz, 1H), 2.02 – 1.90 (m, 3H), 1.62 – 1.52 (m, 2H), 1.46 (td, *J* = 12.2, 2.0 Hz, 1H), 1.32 – 1.23 (m, 1H), 1.15 – 1.07 (apparent triplet, 6H). **^{13}C NMR** (150 MHz, $CDCl_3$) δ 215.48, 168.24, 155.57, 134.02, 132.38, 128.83, 128.63, 124.56, 123.32, 118.09, 107.52, 69.63, 54.83, 51.17, 50.67, 37.11, 36.90, 33.92, 27.38, 21.58, 19.23, 19.14, 19.06. **FTIR** (film, cm^{-1}) 3054.69, 2963.09, 2926.45, 2877.27, 2796.28, 1763.58, 1711.51, 1605.45. **HRMS** (ESI) calcd. for C27H28N2O3 (m/z $M+H^+$): 429.2178, found 429.2176.

Ketone **13g** was also isolated as a yellow oil that was found to be homogeneous as judged by ^{1}H NMR spectroscopy (12 mg, 9%).**^{1}H** NMR (600 MHz, $CDCl_3$) δ 7.79 (dd, *J* = 5.4, 3.0 Hz, 2H), 7.69 (dd, *J* = 5.5, 3.0 Hz, 2H), 7.01 – 6.95 (m, 2H), 6.61 (t, *J* = 7.4, 1.0 Hz, 1H), 6.36 (d, *J* = 7.9 Hz, 1H), 3.60 – 3.53 (m, 1H), 3.47 – 3.40 (m, 1H), 3.39 (s, 1H), 2.94 (s, 3H), 2.32 – 2.19 (m, 2H), 2.08 (d, *J* = 3.6 Hz, 1H), 2.04 – 1.89 (m, 3H), 1.75 – 1.66 (m, 1H), 1.47 – 1.38 (m, 1H), 1.01 (d, *J* = 6.8 Hz, 3H), 0.97 (d, *J* = 6.9 Hz, 3H). **^{13}C** NMR (150 MHz, $CDCl_3$) δ 213.69, 168.41, 153.18, 134.25, 132.41, 130.95, 129.15, 124.12, 123.46, 118.82, 108.33, 76.52, 55.76, 50.98, 50.11, 38.33, 35.07, 34.27, 30.04, 23.36, 20.63, 19.41, 18.08, 16.91. **FTIR** (film, cm^{-1}) 3054.69, 2964.05, 2937.06, 2879.20, 2822.31, 1766.48, 1710.55, 1603.52. **HRMS** (ESI) calcd. for C26H26N2O3 (m/z $M+H^+$): 429.2178, found 429.2176.

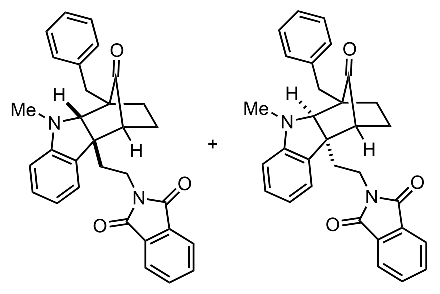

9h **13h**

Following General Procedure E, a mixture of indole (100 mg, 0.329 mmol, 1.0 equiv), sodium carbonate (104 mg, 0.986 mmol, 3.0 equiv), and cyclopentanone **12d** (108 mg, 0.427 mmol, 1.3 equiv) in TFE (1.6 mL, 0.2 M) afforded ketone **9h** as a colorless oil that was found to be approximately 95% pure as judged by ^{1}H NMR spectroscopy (97.2 mg, 62%). **^{1}H NMR** (600 MHz, $CDCl_3$) δ 7.68 (dd, *J* = 8.5, 2.7 Hz, 2H), 7.63 (dd, *J* = 5.3, 3.5 Hz, 2H), 7.34 – 7.28 (m, 4H), 7.23 – 7.19 (m, 1H), 6.88 – 6.80 (m, 2H), 6.41 (t, *J* = 1.0 Hz, 1H), 6.26 (d, *J* = 7.9 Hz, 1H), 3.66 (s, 1H), 3.50 – 3.38 (m, 2H), 3.06 – 2.96 (m, 2H), 2.71 (s, 3H), 2.36 (dt, *J* = 14.2, 8.5 Hz, 1H), 1.99 (d, *J* = 4.9 Hz, 1H), 1.93 (td, *J* = 11.2, 4.5 Hz, 1H), 1.60 (ddd, *J* = 13.8, 6.9, 4.7 Hz, 1H), 1.54 (dt, *J* = 12.4, 4.7 Hz, 1H), 1.40 (td, *J* = 12.2, 1.9 Hz, 1H), 1.28 (td, *J* = 11.3, 4.9 Hz, 1H). **^{13}C NMR** (150 MHz, $CDCl_3$) δ 215.07, 168.16, 154.56, 138.58, 133.93, 132.32, 130.67, 128.81, 128.72, 128.18, 126.70, 124.43, 123.25, 117.71, 106.52, 71.35, 52.99, 51.19, 50.45, 36.34, 35.77, 35.53, 33.75, 22.29, 19.32. **FTIR** (film, cm^{-1}) 2957.30, 2925.48, 2869.56, 1766.48, 1738.51. **HRMS** (ESI) calcd. for C31H28N2O3 (m/z $M+H^+$): 477.2178, found 477.2174.

Ketone **13h** was also isolated as a colorless oil that was found to be approximately 95% pure as judged by ^{1}H NMR spectroscopy (22.3 mg, 14%).**^{1}H NMR** (600 MHz, $CDCl_3$) δ 7.80 (dd, *J* = 5.4, 3.0 Hz, 2H), 7.70 (dd, *J* = 5.5, 3.0 Hz, 2H), 7.33 – 7.25 (m, 4H), 7.22 – 7.17 (m, 1H), 7.06 – 6.99 (m, 2H), 6.66 (d, *J* = 1.0 Hz, 1H), 6.39 (d, *J* = 7.9 Hz, 1H), 3.57 (ddd, *J* = 14.0, 11.0, 4.2 Hz, 1H), 3.47 – 3.39 (m, 2H), 3.20 (d, *J* = 14.7 Hz, 1H), 2.96 (s, 3H), 2.78 (d, *J* = 14.7 Hz, 1H), 2.27 (ddd, *J* = 13.3, 11.0, 6.4 Hz, 1H), 2.19 (d, *J* = 3.6 Hz, 1H), 2.05 – 1.99 (m, 1H), 1.96 (td, *J* = 10.7, 2.4 Hz, 1H), 1.70 (td, *J* = 11.9, 2.4 Hz, 1H), 1.67 – 1.58 (m, 1H), 1.54 (ddd, *J* = 12.1, 10.7, 6.4 Hz, 1H). **^{13}C NMR** (150 MHz, $CDCl_3$) δ 213.41, 168.41, 152.79, 138.79, 134.28, 132.41, 131.00, 130.59, 129.36, 128.61, 126.40, 124.23, 123.48, 118.96, 108.34, 78.47, 53.20, 50.83, 50.21, 38.32, 35.13, 34.26, 32.31, 24.62, 18.25. **FTIR** (film, cm^{-1}) 2957.30, 2926.45, 2870.52, 1770.33, 1734.66. **HRMS** (ESI) calcd. for C31H28N2O3 (m/z $M+H^+$): 477.2178, found 477.2179.

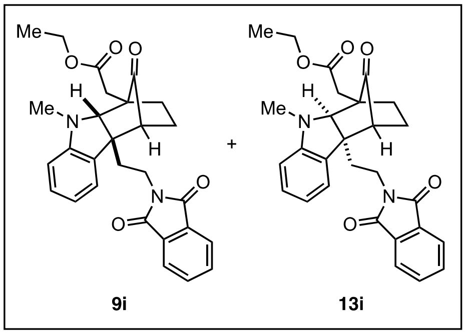

Following General Procedure E, a mixture of indole (100 mg, 0.329 mmol, 1.0 equiv), sodium carbonate (104 mg, 0.986 mmol, 3.0 equiv), and cyclopentanone **12e** (106 mg, 0.427 mmol, 1.3 equiv) in TFE (1.6 mL, 0.2 M) afforded ketone **9i** as a yellow oil that was found to be homogeneous as judged by ^{1}H NMR spectroscopy (126 mg, 81%). **^{1}H NMR** (600 MHz, $CDCl_3$) δ 7.67 (dd, *J* = 5.4, 3.1 Hz, 2H), 7.62 (dd, *J* = 5.5, 3.0 Hz, 2H), 6.85 – 6.76 (m, 2H), 6.37 (t, *J* = 0.9 Hz, 1H), 6.25 (d, *J* = 7.8 Hz, 1H), 4.25 – 4.13 (m, 2H), 3.98 (s, 1H), 3.63 (dt, *J* = 14.1, 8.2 Hz, 1H), 3.52 (ddd, *J* = 14.1, 8.5, 3.9 Hz, 1H), 2.87 (s, 3H), 2.65 (d, *J* = 16.4 Hz, 1H), 2.59 (d, *J* = 16.4 Hz, 1H), 2.43 (dt, *J* = 14.2, 8.6 Hz, 1H), 2.02 (d, *J* = 4.5 Hz, 1H), 1.97 (td, *J* = 11.0, 3.4 Hz, 1H), 1.73 – 1.54 (m, 3H), 1.35 – 1.26 (m, 4H). **^{13}C NMR** (150 MHz, $CDCl_3$) δ 212.97, 171.38, 168.21, 168.20, 154.51, 133.86, 132.34, 128.79, 128.14, 124.44, 123.19, 117.54 106.27, 71.89, 60.91, 51.33, 49.94, 49.54, 36.29, 35.27, 34.22, 33.80, 22.38, 19.32, 14.55. **FTIR** (film, cm^{-1}) 3054.69, 2979.48, 2938.98, 2873.42, 2255.34, 1769.37, 1712.48, 1605.45. **HRMS** (ESI) calcd. for C28H28N2O5 (m/z M+H$^+$): 473.2076, found: 473.2076.

Ketone **13i** was also isolated as a yellow oil that was found to be homogeneous as judged by ^{1}H NMR spectroscopy (17 mg, 11%).**^{1}H NMR** (600 MHz, $CDCl_3$) δ 7.79 (dd, *J* = 5.4, 3.0 Hz, 2H), 7.69 (dd, *J* = 5.5, 3.0 Hz, 2H), 7.03 – 6.96 (m, 2H), 6.63 (t, *J* = 0.9 Hz, 1H), 6.36 (d, *J* = 7.0 Hz, 1H), 4.18 (q, *J* = 7.1, 1.2 Hz, 2H) , 3.67 – 3.56 (m, 2H), 3.45 (ddd, *J* = 13.7, 10.1, 6.2 Hz, 1H), 2.92 (s, 3H), 2.74 (d, *J* = 17.0 Hz, 1H), 2.46 (d, *J* = 16.9 Hz, 1H), 2.27 (ddd, *J* = 13.4, 10.7, 6.2 Hz, 1H), 2.22 (d, *J* = 3.7 Hz, 1H), 2.10 – 2.00 (m, 2H), 1.93 (ddd, *J* = 12.0, 10.6, 6.2 Hz, 1H), 1.88 – 1.75 (m, 2H), 1.30 (t, *J* = 7.1 Hz, 3H). **^{13}C NMR** (150 MHz, $CDCl_3$) δ 212.48, 172.39, 168.35, 152.69, 134.23, 132.40, 130.99, 129.38, 124.27, 123.46, 119.06, 108.61, 76.28, 60.84, 50.99, 49.90, 49.54, 37.63, 35.02, 34.25, 31.38, 25.53, 18.13, 14.61. **FTIR** (film, cm^{-1}) 3055.66, 2980.45, 2938.02, 2822.31, 1770.33, 1711.51, 1604.48.

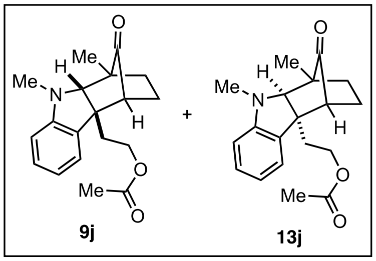

Following General Procedure E, a mixture of indole (100 mg, 0.460 mmol, 1.0 equiv), sodium carbonate (146 mg, 1.38 mmol, 3.0 equiv), and cyclopentanone **12a** (105 mg, 0.598 mmol, 1.3 equiv) in TFE (2.3 mL, 0.2 M) afforded ketone **9j** as a colorless oil that was found to be homogeneous as judged by ^{1}H NMR spectroscopy (110 mg, 76%). **^{1}H NMR** (600 MHz, $CDCl_3$) δ 7.11 (d, *J* = 7.7 Hz, 1H), 6.85 (d, *J* = 7.3 Hz, 1H), 6.63 (t, *J* = 7.4 Hz, 1H), 6.40 (d, *J* = 7.9 Hz, 1H), 3.98 (ddd, *J* = 11.3, 8.6, 5.4 Hz, 1H), 3.85 (ddd, *J* = 11.3, 8.1, 6.5 Hz, 1H), 3.43 (s, 1H), 2.87 (s, 3H), 2.14 – 2.04 (m, 2H), 1.91 (s, 3H), 1.90 – 1.86 (m, 1H), 1.82 – 1.73 (m, 1H), 1.67 – 1.58 (m, 1H), 1.38 – 1.29 (m, 2H), 1.17 (s, 3H).**^{13}C NMR** (150 MHz, $CDCl_3$) δ 215.57, 171.13, 154.29, 129.26, 128.81, 124.87, 117.54, 106.20, 74.80, 61.08, 50.88, 49.92, 47.90, 38.43, 35.12, 23.57, 21.16, 19.85, 15.02. **FTIR** (film, cm^{-1}) 2957.30, 2925.48, 2869.56, 1766.48, 1738.51. **HRMS** (ESI) calcd. for C19H23NO3 (m/z M+H$^+$): 314.1756, found 314.1758.

Ketone **13j** was also isolated as a colorless oil that was found to be homogeneous as judged by ^{1}H NMR spectroscopy (27 mg, 19%).**^{1}H NMR** (600 MHz, $CDCl_3$) δ 7.04 (t, *J* = 7.7 Hz, 1H), 6.87 (d, *J* = 7.4 Hz, 1H), 6.61 (t, *J* = 7.4 Hz, 1H), 6.31 (d, *J* = 7.9 Hz, 1H), 4.03 (ddd, *J* = 11.2, 8.5, 5.7 Hz, 1H), 3.91 (ddd, *J* = 11.2, 8.1, 6.5 Hz, 1H), 3.18 (s, 1H), 2.87 (s, 3H), 2.19 (d, *J* = 4.0 Hz, 1H), 2.17 – 2.04 (m, 2H), 2.04 – 1.96 (m, 4H), 1.85 – 1.77 (m, 1H), 1.71 – 1.61 (m, 2H), 1.06 (s, 3H). **^{13}C NMR** (150 MHz, $CDCl_3$) δ 214.69, 171.22, 152.13, 131.02, 129.44, 124.29, 118.13, 107.40, 78.71, 61.39, 50.32, 49.77, 48.78, 36.96, 35.66, 28.37, 21.31, 18.29, 11.74. **FTIR** (film, cm^{-1}) 2957.30, 2926.45, 2870.52, 1770.33, 1734.66. **HRMS** (ESI) calcd. for C19H23NO3 (m/z M+H$^+$): 314.1756, found 314.1755.

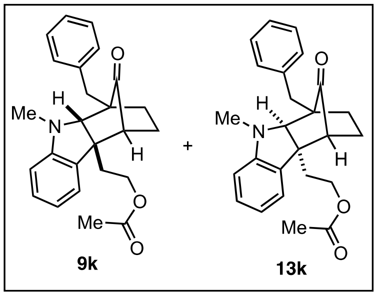

9k **13k**

Following General Procedure E, a mixture of indole (100 mg, 0.460 mmol, 1.0 equiv), sodium carbonate (146 mg, 1.38 mmol, 3.0 equiv), and cyclopentanone **12d** (151 mg, 0.598 mmol, 1.3 equiv) in TFE (2.3 mL, 0.2 M) afforded ketone **9k** as a colorless oil that was found to be homogeneous as judged by ^{1}H NMR spectroscopy (129 mg, 71%). **^{1}H NMR** (600 MHz, $CDCl_3$) δ 7.33 – 7.26 (m, 4H), 7.24 – 7.18 (m, 1H), 7.10 (t, *J* = 7.7 Hz, 1H), 6.83 (d, *J* = 7.3 Hz, 1H), 6.64 (t, *J* = 7.4 Hz, 1H), 6.42 (d, *J* = 7.9 Hz, 1H), 3.84 (t, *J* = 1.4 Hz, 2H), 3.50 (s, 1H), 3.00 (d, *J* = 14.4 Hz, 1H), 2.96 (d, *J* = 14.1 Hz, 1H), 2.68 (s, 3H), 2.08 – 2.00 (m, 2H), 1.95 (td, *J* = 11.1, 4.7 Hz, 1H), 1.83 (s, 3H), 1.75 (dt, *J* = 14.0, 6.9 Hz, 1H), 1.63 – 1.54 (m, 1H), 1.41 (td, *J* = 12.0, 1.9 Hz, 1H), 1.32 (td, *J* = 11.2, 4.7 Hz, 1H). **^{13}C NMR** (150 MHz, $CDCl_3$) δ 215.05, 171.04, 154.74, 138.60, 130.68, 129.22, 128.76, 128.74, 126.74, 124.70, 118.02, 107.13, 72.14, 61.06, 53.03, 51.16, 50.17, 37.89, 36.02, 35.70, 22.32, 21.08, 19.67. **FTIR** (film, cm^{-1}) 3507.88, 3458.71, 3058.55, 2926.45, 2874.38, 2794.35, 1763.58, 1738.51, 1604.48. **HRMS** (ESI) calcd. for C25H27NO3 (m/z M+H$^+$): 390.2069, found 390.2072.

Ketone **13k** was also isolated as a colorless oil that was found to be homogeneous as judged by ^{1}H NMR spectroscopy (12 mg, 6%).**^{1}H NMR** (600 MHz, $CDCl_3$) δ 7.32 – 7.22 (m, 5H), 7.20 – 7.15 (m, 1H), 7.06 (t, *J* = 7.7 Hz, 1H), 6.88 (d, *J* = 7.4 Hz, 1H), 6.65 (t, *J* = 6.6 Hz, 1H), 6.39 (d, *J* = 7.9 Hz, 1H), 4.01 (ddd, *J* = 11.2, 8.6, 5.7 Hz, 1H), 3.89 (ddd, *J* = 11.1, 8.2, 6.4 Hz, 1H), 3.31 (s, 1H), 3.14 (d, *J* = 14.7 Hz, 1H), 2.89 (s, 3H), 2.74 (d, *J* = 14.7 Hz, 1H), 2.17 (d, *J* = 3.6 Hz, 1H), 2.15 – 2.03 (m, 2H), 2.00 – 1.92 (m, 4H), 1.73 – 1.61 (m, 2H), 1.54 – 1.47 (m, 1H). **^{13}C NMR** (150 MHz, $CDCl_3$) δ 213.43, 171.18, 152.70, 138.80, 131.26, 130.54, 129.45, 128.58, 126.41, 124.24, 118.78, 108.51, 79.00, 61.34, 52.94, 50.55, 50.14, 38.44, 35.69, 32.20, 24.84, 21.29, 18.27. **FTIR** (film, cm^{-1}) 3026.73, 2918.73, 1844.58, 1769.37, 1735.62. **HRMS** (ESI) calcd. for C25H27NO3 (m/z M+H$^+$): 390.2069, found 390.2069.

F. Annulation Reactions with Dibrominated Ketones:

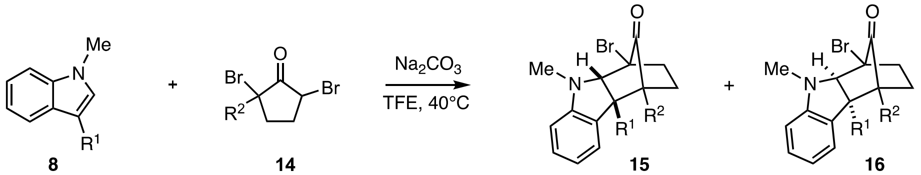

8 **14** **15** **16**

General Procedure F:

To a solution of indole **8** (1.0 equiv) in trifluoroethanol (0.2 M) was sequentially added sodium carbonate (3.0 equiv) and 2,5-dibromoclopentanone **14** (2.0 equiv). The reaction mixture was stirred at 40 °C until the reaction was judged complete as determined by thin-layer chromatographic analysis and then cooled to room temperature. The mixture was filtered through a short pad of silica gel and washed with EtOAc The filtrate was concentrated and the residue was purified via silica gel flash column chromatography (hexanes/EtOAc) to afford **15** as the major product and diastereomer **16** as the minor product.

Modified General Procedure F:

To a solution of indole **8** (1.0 equiv) in hexafluoroisopropanol (0.2 M) was sequentially added sodium carbonate (3.0 equiv) and 2,5-dibromoclopentanone **14** (2.0 equiv). The reaction mixture was stirred at room temperature for 40 hours. Then an additional 2.0 equiv of **14** was added and the reaction continued to stir for an additional 20 hours (Total reaction time 60 hours). When the reaction was judged complete as determined by thin-layer chromatographic analysis the mixture was filtered through a short pad of silica gel and washed with EtOAc The filtrate was concentrated and the residue was purified via silica gel flash column chromatography (hexanes/EtOAc) to afford **15** as the major product and diastereomer **16** as the minor product.

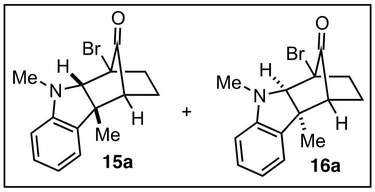

Following General Procedure F, a mixture of indole (100 mg, 0.689 mmol, 1.0 equiv), sodium carbonate (219 mg, 2.06 mmol, 3.0 equiv), and cyclopentanone **14f** (333 mg, 1.37 mmol, 2.0 equiv) in TFE (3.4 mL, 0.2 M) afforded ketone **15a** as a colorless oil that was found to be homogeneous as judged by ^{1}H NMR spectroscopy (142 mg, 67%). **^{1}H NMR** (600 MHz, $CDCl_3$) δ 7.15 (t, *J* = 7.7 Hz, 1H), 6.87 (d, *J* = 7.3 Hz, 1H), 6.66 (t, *J* = 7.4 Hz, 1H), 6.46 (d, *J* = 7.9 Hz, 1H), 3.72 (d, *J* = 2.0 Hz, 1H), 2.99 (s, 3H), 2.40 (td, *J* = 11.2, 4.2 Hz, 1H), 2.07 (d, *J* = 5.0 Hz, 1H), 1.89 (td, *J* = 12.2, 2.0 Hz, 1H), 1.84 – 1.76 (m, 1H), 1.47 (td, *J* = 11.2, 4.8 Hz, 1H), 1.38 (s, 3H). **^{13}C NMR** (150 MHz, $CDCl_3$) δ 206.54, 153.65, 130.84, 129.31, 124.22, 117.89, 106.28, 78.75, 63.72, 50.80, 45.81, 34.93, 28.19, 26.50, 21.77. **FTIR** (film, cm^{-1}) 2983.34, 2957.52, 2886.92, 1774.19, 1603.52, 1488.78. **HRMS** (ESI) calcd. for C15H16BrNO (m/z M+H^{+}): 306.0494; found 306.0488.

Ketone **16a** was also isolated as a colorless oil that was found to be homogeneous as judged by ^{1}H NMR spectroscopy (12 mg, 6%).**^{1}H NMR** (600 MHz, $CDCl_3$) δ 7.07 (t, *J* = 7.7 Hz, 1H), 6.87 (d, *J* = 7.3 Hz, 1H), 6.65 (t, *J* = 7.4 Hz, 1H), 6.38 (d, *J* = 7.9 Hz, 1H), 3.30 (s, 1H), 3.00 (s, 3H), 2.29 (td, *J* = 11.9, 8.8 Hz, 1H), 2.23 – 2.16 (m, 2H), 2.13 (td, *J* = 10.5, 3.0 Hz, 1H), 2.05 – 1.96 (m, 1H), 1.52 (s, 3H). **^{13}C NMR** (150 MHz, $CDCl_3$) δ 205.86, 151.45, 133.68, 129.32, 123.31, 118.68, 107.94, 80.12, 66.49, 50.47, 45.85, 38.13, 31.28, 25.20, 20.76. **FTIR** (film, cm^{-1}) 2965.98, 2922.59, 2888.84, 1778.05, 1601.59, 1491.67 **HRMS** (ESI) calcd. for C15H16BrNO (m/z M+H^{+}): 306.0494; found 306.0489.

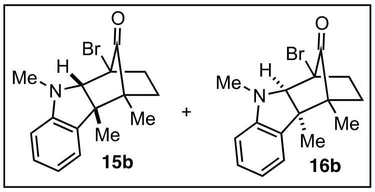

Following General Procedure F, a mixture of indole (200 mg, 1.37 mmol, 1.0 equiv), sodium carbonate (438 mg, 4.11 mmol, 3.0 equiv), and cyclopentanone **14a** (705 mg, 2.75 mmol, 2.0 equiv) in TFE (6.4 mL, 0.2 M) afforded ketone **15b** as a light blue solid that was found to be homogeneous as judged by ^{1}H NMR spectroscopy. Nitrogen gas was blown over the isolated material for ~24 h to volatilize remaining impurities. (356 mg, 81%). **^{1}H NMR** (600 MHz, $CDCl_3$) δ 7.15 (t, *J* = 7.7 Hz, 1H), 6.90 (d, *J* = 7.3 Hz, 1H), 6.65 (t, *J* = 7.4 Hz, 1H), 6.46 (d, *J* = 7.9 Hz, 1H), 3.71 (s, 1H), 2.99 (s, 3H), 2.44 (td, *J* = 11.1, 4.3 Hz, 1H), 1.92 (td, *J* = 12.3, 2.0 Hz, 1H), 1.65 – 1.54 (m, 2H), 1.49 (td, *J* = 12.7, 4.3 Hz, 1H), 1.25 (s, 3H), 1.16 (s, 3H). **^{13}C NMR** (150 MHz, $CDCl_3$) δ 208.39, 153.51, 130.97, 129.26, 123.86, 117.68, 106.12, 79.11, 63.61, 52.33, 45.61, 34.84, 29.69, 27.60, 26.13, 11.97. **FTIR** (film, cm^{-1}) 3025.76, 2961.16, 2869.56, 2823.28, 1785.76, 1605.45. **HRMS** (ESI) calcd. for C16H18BrNO (m/z M+H^{+}): 320.0650, found 320.0647.

Ketone **16b** was also isolated as a light blue solid that was found to be homogeneous as judged by ^{1}H NMR spectroscopy. Nitrogen gas was blown over the isolated material for ~24 h to volatilize remaining impurities. (19 mg, 4%).**^{1}H NMR** (600 MHz, $CDCl_3$) δ 7.06 (t, *J* = 7.7 Hz, 1H), 6.87 (d, *J* = 7.5 Hz, 1H), 6.63 (t, *J* = 7.4 Hz, 1H), 6.38 (d, *J* = 8.0 Hz, 1H), 3.35 (s, 1H), 2.97 (s, 3H), 2.35 – 2.27 (m, 1H), 2.26 – 2.15 (m, 2H), 1.77 – 1.68 (m, 1H), 1.50 (s, 3H), 1.11 (s, 3H). **^{13}C NMR** (150 MHz, $CDCl_3$) δ 207.94, 152.41, 132.02, 129.12, 125.19, 118.11, 108.28, 80.57, 66.37, 52.59, 45.05, 38.99, 31.21, 28.43, 24.03, 12.07. **FTIR** (film, cm^{-1}) 3049.87, 2973.70, 2883.06, 2822.31, 1786.72, 1603.52. **HRMS** (ESI) calcd. for C16H18BrNO (m/z M+H^{+}): 320.0650, found 320.0648.

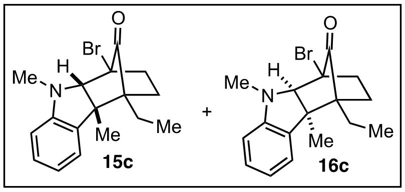

Following General Procedure F, a mixture of indole (100 mg, 0.689 mmol, 1.0 equiv), sodium carbonate (219 mg, 2.06 mmol, 3.0 equiv), and cyclopentanone **14b** (371 mg, 1.37 mmol, 2.0 equiv) in TFE (3.4 mL, 0.2 M) afforded ketone **13-2a** as a white solid that was found to be homogeneous as judged by ^{1}H NMR spectroscopy (168 mg, 73%). **^{1}H NMR** (600 MHz, $CDCl_3$) δ 7.14 (t, *J* = 7.7 Hz, 1H), 6.91 (d, *J* = 7.3 Hz, 1H), 6.63 (t, *J* = 7.4 Hz, 1H), 6.46 (d, *J* = 7.9 Hz, 1H), 3.66 (s, 1H), 2.98 (s, 3H), 2.47 (td, *J* = 11.6, 3.8 Hz, 1H), 1.96 – 1.87 (m, 2H), 1.81 (td, *J* = 12.6, 3.8 Hz, 1H), 1.52 (dq, *J* = 14.9, 7.5 Hz, 1H), 1.42 (td, *J* = 11.2, 5.4 Hz, 1H), 1.26 (s, 3H), 0.96 (t, *J* = 7.5 Hz, 3H). **^{13}C NMR** (150 MHz, $CDCl_3$) δ 207.86, 153.63, 130.96, 129.15, 124.44, 117.57, 106.06, 79.16, 63.80, 53.15, 49.03, 34.85, 27.41, 25.91, 24.16, 18.33, 9.34. **FTIR** (film, cm^{-1}) 3051.80, 2967.91, 2880.17, 2823.28, 1779.01, 1603.52. **HRMS** (ESI) calcd. for C17H20BrNO (m/z M+H^{+}): 334.0807, found 334.0807.

Ketone **16c** was also isolated as a white solid that was found to be approximately 95% pure as judged by ^{1}H NMR spectroscopy (14 mg, 6%).**^{1}H NMR** (600 MHz, $CDCl_3$) δ 7.07 (t, *J* = 7.7 Hz, 1H), 6.90 (d, *J* = 7.4 Hz, 1H), 6.64 (t, *J* = 7.8 Hz, 1H), 6.39 (d, *J* = 7.9 Hz, 1H), 3.29 (s, 1H), 2.96 (s, 3H), 2.31 (td, *J* = 12.6, 3.1 Hz, 1H), 2.24 (ddd, *J* = 13.0, 10.5, 6.9 Hz, 1H), 2.10 (td, *J* = 10.5, 3.2 Hz, 1H), 1.93 (td, *J* = 12.6, 7.0 Hz, 1H), 1.70 – 1.59 (m, 2H), 1.54 (s, 3H), 0.99 (t, *J* = 7.6 Hz, 3H). **^{13}C NMR** (150 MHz, $CDCl_3$) δ 207.56, 152.60, 131.72, 129.12, 125.36, 118.16, 108.38, 80.94, 66.28, 53.42, 48.09, 39.08, 30.89, 25.45, 23.63, 19.53, 9.95. **FTIR** (film, cm^{-1}) 3048.91, 2964.05, 2880.17, 2817.49, 1779.97, 1604.48. **HRMS** (ESI) calcd. for C17H20BrNO (m/z $M+H^+$): 334.0807, found 334.0802.

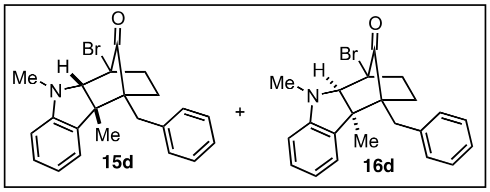

Following General Procedure F, a mixture of indole (100 mg, 0.689 mmol, 1.0 equiv), sodium carbonate (219 mg, 2.06 mmol, 3.0 equiv), and cyclopentanone **14d** (457 mg, 1.37 mmol, 2.0 equiv) in TFE (3.4 mL, 0.2 M) afforded ketone **15d** as a white solid that was found to be homogeneous as judged by ^{1}H NMR spectroscopy (136 mg, 50%). **^{1}H NMR** (600 MHz, $CDCl_3$) δ 7.28 – 7.18 (m, 5H), 7.14 (t, *J* = 7.7 Hz, 1H), 6.64 (d, *J* = 7.3 Hz, 1H), 6.59 (t, *J* = 7.3 Hz, 1H), 6.45 (d, *J* = 7.9 Hz, 1H), 3.69 (s, 1H), 3.34 (d, *J* = 14.1 Hz, 1H), 2.97 (s, 3H), 2.71 (d, *J* = 14.1 Hz, 1H), 2.40 (td, *J* = 10.8, 4.4 Hz, 1H), 1.77 (td, *J* = 12.2, 2.0 Hz, 1H), 1.53 – 1.39 (m, 2H), 1.38 (s, 3H). **^{13}C NMR** (150 MHz, $CDCl_3$) δ 207.50, 153.64, 137.58, 130.92, 130.71, 129.26, 128.76, 126.84, 124.34, 117.74, 106.11, 79.13, 63.53, 53.22, 49.94, 34.84, 32.68, 27.49, 25.92, 25.54 **FTIR** (film, cm^{-1}) 3058.55, 3026.73, 2950.55, 2870.52, 1781.90, 1604.48, 1492.63 **HRMS** (ESI) calcd. for C22H22BrNO (m/z $M+H^+$): 396.0963; found 396.0952.

We were not able to obtain an analytically pure sample of ketone **16d** for characterization.

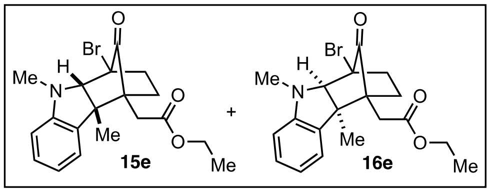

Following General Procedure F, a mixture of indole (100 mg, 0.689 mmol, 1.0 equiv), sodium carbonate (218 mg, 2.06 mmol, 3.0 equiv), and cyclopentanone **14e** (451 mg, 1.37 mmol, 2.0 equiv) in TFE (3.4 mL, 0.2 M) afforded ketone **15e** as a yellow oil that was found to be homogeneous as judged by ^{1}H NMR spectroscopy (146 mg, 54%). **^{1}H NMR** (600 MHz, $CDCl_3$) δ 7.14 (t, *J* = 7.7 Hz, 1H), 6.85 (d, *J* = 7.3 Hz, 1H), 6.63 (t, *J* = 7.4 Hz, 1H), 6.46 (d, *J* = 7.9 Hz, 1H), 4.18 – 4.08 (m, 2H), 3.69 (d, *J* = 1.8 Hz, 1H), 2.98 (s, 3H), 2.72 (d, *J* = 15.7 Hz, 1H), 2.57 (d, *J* = 15.7 Hz, 1H), 2.48 (td, *J* = 11.3, 3.2 Hz, 1H), 2.10 – 1.95 (m, 2H), 1.63 (td, *J* = 11.1, 5.2 Hz, 1H), 1.30 (s, 3H), 1.23 (t, *J* = 7.2 Hz, 3H). **^{13}C NMR** (150 MHz, $CDCl_3$) δ 205.57, 171.06, 153.59, 130.10, 129.42, 124.49, 117.80, 106.27, 79.22, 62.88, 61.05, 52.94, 47.28, 34.97, 31.58, 27.64, 26.53, 25.88, 14.43. **FTIR** (film, cm^{-1}) 3051.80, 2959.23, 2871.49, 2823.28, 1787.69, 1735.621691.27. **HRMS** (ESI) calcd. for C19H22BrNO3 (m/z $M+H^+$): 392.0861, found 392.0843.

Ketone **16e** was not successfully isolated.

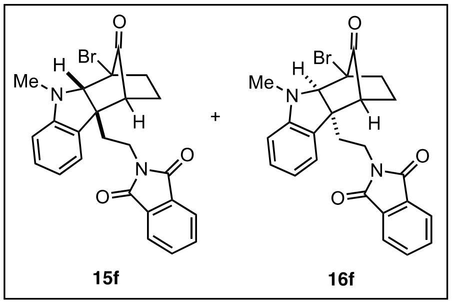

Following General Procedure F, a mixture of indole (100 mg, 0.329 mmol, 1.0 equiv), sodium carbonate (104 mg, 0.986 mmol, 3.0 equiv), and cyclopentanone **14f** (158 mg, 0.657 mmol, 2.0 equiv) in TFE (1.6 mL, 0.2 M) afforded ketone **15f** as a yellow oil that was found to be homogeneous as judged by ^{1}H NMR spectroscopy (114 mg, 75%). **^{1}H NMR** (600 MHz, $CDCl_3$) δ 7.68 (dd, *J* = 3.2, 1.8 Hz, 2H), 7.63 (dd, *J* = 3.0, 2.1 Hz, 2H), 6.84 (t, *J* = 7.7 Hz, 1H), 6.81 (d, *J* = 7.4 Hz, 1H), 6.39 (t, *J* = 0.9 Hz, 1H), 6.29 (d, *J* = 7.9 Hz, 1H), 4.10 (s, 1H), 3.59 (ddd, *J* = 14.2, 8.6, 7.7 Hz, 1H), 3.50 (ddd, *J* = 14.2, 8.5, 3.9 Hz, 1H), 3.03 (s, 3H), 2.46 (dt, *J* = 14.3, 8.6 Hz, 1H), 2.38 (td, *J* = 11.2, 4.6 Hz, 1H), 2.05 (d, *J* = 5.0 Hz, 1H), 1.90 (td, *J* = 12.4, 2.0 Hz, 1H), 1.80 – 1.68 (m, 2H), 1.51 – 1.43 (m, 2H).**^{13}C NMR** (150 MHz, $CDCl_3$) δ 206.43, 168.15, 153.88, 133.99, 132.25, 129.26, 127.09, 124.28, 123.29, 117.84, 106.21, 74.73, 63.42, 53.42, 45.88, 36.57, 34.46, 33.60, 26.75, 21.18. **FTIR** (film, cm^{-1}) 3054.69, 2983.34, 2947.66, 2873.42, 1768.40, 1710.55, 1605.45. **HRMS** (ESI) calcd. for C24H21BrN2O3 (m/z $M+H^+$): 465.0814, found 465.0822.

Ketone **16f** was also isolated as a yellow oil that was found to be approximately 90% pure as judged by ^{1}H NMR spectroscopy (7 mg, 4%).**^{1}H NMR** (600 MHz, $CDCl_3$) δ 7.80 (dd, *J* = 5.4, 3.0 Hz, 2H), 7.70 (dd, *J* = 5.5, 3.0 Hz, 2H), 7.03 (t, *J* = 7.7 Hz, 1H), 6.97 (d, *J* = 7.3 Hz, 1H), 6.64 (t, *J* = 7.4 Hz, 1H), 6.36 (d, *J* = 7.9 Hz, 1H), 3.59 (s, 1H), 3.55 (td, *J* = 10.7, 4.3 Hz, 1H), 3.50 – 3.41 (m, 1H), 3.04 (s, 3H), 2.34 – 2.29 (m, 2H), 2.27 (d, *J* = 4.0 Hz, 1H), 2.23 (ddd, *J* = 12.7, 10.4, 6.5 Hz, 1H), 2.16 (td, *J* = 10.5, 3.2 Hz, 1H), 2.11 – 2.04 (m, 1H), 2.03 – 1.96 (m, 1H). **^{13}C NMR** (150 MHz, $CDCl_3$) δ 205.04, 168.36, 151.95, 134.37, 132.34, 129.82, 129.45, 124.03, 123.55, 119.03, 108.14, 77.78, 65.99, 52.93, 45.76, 37.63, 35.05, 33.90, 31.35, 20.30. **FTIR** (film, cm^{-1}) 3057.58, 2922.59, 2857.02, 1773.23, 1712.48, 1609.31, 1491.67. **HRMS** (ESI) calcd. for C24H21BrN2O3 (m/z M+H^{+}): 465.0814, found 465.0817.

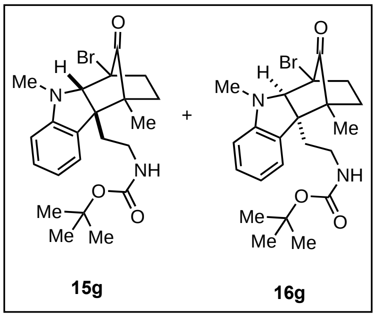

Following General Procedure F, a mixture of indole (100 mg, 0.364 mmol, 1.0 equiv), sodium carbonate (115 mg, 1.09 mmol, 3.0 equiv), and cyclopentanone **14a** (186 mg, 0.729 mmol, 2.0 equiv) in HFIP (1.8 mL, 0.2 M) afforded ketone **15g** as a colorless oil that was found to be homogeneous as judged by ^{1}H NMR spectroscopy (84 mg, 52%). **^{1}H NMR** (600 MHz, $CDCl_3$) δ 7.13 (t, *J* = 7.4 Hz, 1H), 6.87 (d, *J* = 7.2 Hz, 1H), 6.64 (t, *J* = 7.4 Hz, 1H), 6.43 (d, *J* = 7.9 Hz, 1H), 4.27 (broad s, 1H), 3.87 (s, 1H), 3.05 – 2.92 (m, 4H), 2.61 (broad s, 1H), 2.44 – 2.33 (m, 1H), 2.00 – 1.86 (m, 2H), 1.63 (td, *J* = 11.2, 5.0 Hz, 1H), 1.52 – 1.31 (m, 11H), 1.15 (s, 3H). **^{13}C NMR** (150 MHz, $CDCl_3$) δ 208.25, 155.88, 153.78, 129.62, 127.96, 123.82, 117.99, 106.25, 79.56, 75.16, 63.52, 55.05, 45.64, 36.91, 36.76, 34.49, 29.80, 28.71, 27.28, 12.22. **FTIR** (film, cm^{-1}) 3417.24, 3053.73, 2977.55, 2934.16, 2872.45, 1786.72, 1710.55, 1605.45, 1563.02. **HRMS** (ESI) calcd. for C22H29BrN2O3 (m/z M+H^{+}): 449.1440; found 449.1434.

***Note:** This reaction was run at room temperature.

Ketone **16g** was also isolated as a colorless oil that was found to be homogeneous as judged by ^{1}H NMR spectroscopy (21 mg, 13%).**^{1}H NMR** (600 MHz, $CDCl_3$) δ 7.06 (t, *J* = 7.7 Hz, 1H), 6.83 (d, *J* = 7.5 Hz, 1H), 6.62 (t, *J* = 7.4 Hz, 1H), 6.37 (d, *J* = 8.0 Hz, 1H), 4.36 (broad s, 1H), 3.56 (s, 1H), 3.02 – 2.91 (m, 4H), 2.66 (broad s, 1H), 2.36 – 2.19 (m, 3H), 2.05 (broad s, 1H), 1.96 (s, 1H), 1.77 – 1.64 (m, 1H), 1.52 – 1.36 (m, 11H), 1.13 (s, 3H). **^{13}C NMR** (150 MHz, $CDCl_3$) δ 207.71, 156.10, 153.04, 129.58, 127.89, 125.83, 118.17, 108.38, 79.77, 78.09, 66.04, 55.22, 45.44, 38.48, 37.20, 34.57, 31.38, 28.77, 28.05, 12.46. **FTIR** (film, cm^{-1}) 3412.42, 3052.76, 2976.59, 2931.27, 2882.09, 1788.65, 1708.62, 1602.56, 1563.99. **HRMS** (ESI) calcd. for C22H29BrN2O3 (m/z M+H^{+}): 449.1440; found 449.1441.

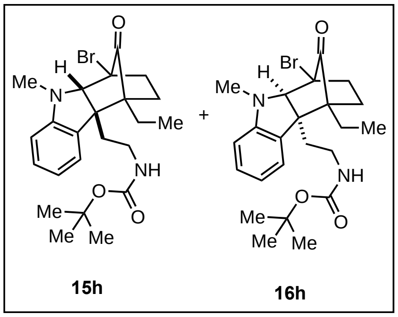

Following General Procedure F, a mixture of indole (100 mg, 0.364 mmol, 1.0 equiv), sodium carbonate (115 mg, 1.09 mmol, 3.0 equiv), and cyclopentanone **14b** (196 mg, 0.729 mmol, 2.0 equiv) in HFIP (1.8 mL, 0.2 M) afforded ketone **15h** as a colorless oil that was found to be homogeneous as judged by ^{1}H NMR spectroscopy (84 mg, 50%). **^{1}H NMR** (600 MHz, $CDCl_3$) δ 7.14 (t, *J* = 7.1 Hz, 1H), 6.88 (d, *J* = 7.4 Hz, 1H), 6.64 (t, *J* = 7.4 Hz, 1H), 6.43 (d, *J* = 7.9 Hz, 1H), 4.24 (broad s, 1H), 3.83 (s, 1H), 3.06 – 2.93 (m, 4H), 2.59 (broad s, 1H), 2.42 (td, *J* = 11.3, 4.0 Hz, 1H), 2.03 – 1.84 (m, 3H), 1.79 (td, *J* = 12.7, 4.3 Hz, 1H), 1.63 – 1.32 (m, 12H), 0.95 (t, *J* = 7.5 Hz, 3H). **^{13}C NMR** (150 MHz, $CDCl_3$) δ 207.93, 155.91, 154.02, 129.59, 127.95, 124.55, 117.93, 106.24, 79.60, 75.18, 63.76, 56.04, 49.14, 36.83, 36.64, 34.52, 28.73, 27.11, 24.31, 18.61, 9.53. **FTIR** (film, cm^{-1}) 3414.35, 2974.66, 2939.95, 2883.06, 1779.01, 1710.55, 1603.52, 1546.63. **HRMS** (ESI) calcd. for C23H31BrN2O3 (m/z M+H^{+}): 463.1596, found 463.1598.

***Note:** This reaction was carried out at room temperature.

Ketone **16h** was also isolated as a colorless oil that was found to be homogeneous as judged by ^{1}H NMR spectroscopy (24 mg, 14%).**^{1}H NMR** (600 MHz, $CDCl_3$) δ 7.06 (t, *J* = 7.6 Hz, 1H), 6.85 (d, *J* = 7.4 Hz, 1H), 6.63 (t, *J* = 7.4 Hz, 1H), 6.36 (d, *J* = 7.9 Hz, 1H), 4.36 (broad s, 1H), 3.54 (s, 1H), 2.99 – 2.92 (m, 4H), 2.60 (broad s, 1H), 2.34 – 2.22 (m, 2H), 2.15 – 2.05 (m, 2H), 2.03 – 1.89 (m, 2H), 1.74 – 1.66 (m, 1H), 1.66 –

1.58 (m, 2H), 1.50 – 1.36 (m, 9H), 0.97 (t, *J* = 7.6 Hz, 3H). **^{13}C NMR** (150 MHz, $CDCl_3$) δ 207.33, 156.07, 153.24, 129.55, 127.59, 125.97, 118.18, 108.44, 79.73, 78.02, 65.98, 56.07, 48.42, 38.54, 37.26, 35.47, 31.01, 28.76, 23.03, 19.57, 9.99. **FTIR** (film, cm^{-1}) 3412.42, 2974.66, 2938.02, 2883.06, 1782.87, 1708.62, 1601.59. **HRMS** (ESI) calcd. for C23H31BrN2O3 (m/z M+H$^+$): 463.1596, found 463.1598.

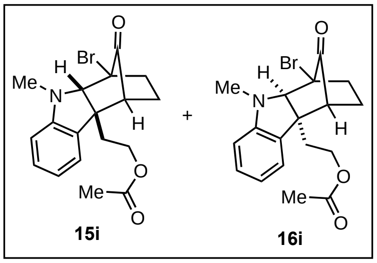

Following General Procedure F, a mixture of indole (100 mg, 0.460 mmol, 1.0 equiv), sodium carbonate (146 mg, 1.38 mmol, 3.0 equiv), and cyclopentanone **12f** (222 mg, 0.921 mmol, 2.0 equiv) in TFE (2.3 mL, 0.2 M) afforded ketone **15i** as a colorless oil that was found to be homogeneous as judged by ^{1}H NMR spectroscopy (88 mg, 51%). **^{1}H NMR** (600 MHz, $CDCl_3$) δ 7.14 (t, *J* = 1.3 Hz, 1H), 6.83 (d, *J* = 1.2 Hz, 1H), 6.66 (t, *J* = 1.0 Hz, 1H), 6.45 (d, *J* = 7.9 Hz, 1H), 3.96 (d, *J* = 2.0 Hz, 1H), 3.95 – 3.86 (m, 2H), 2.99 (s, 3H), 2.37 (td, *J* = 11.2, 4.7 Hz, 1H), 2.17 – 2.07 (m, 2H), 1.94 – 1.83 (m, 5H), 1.79 (tt, *J* = 12.6, 4.8 Hz, 1H), 1.51 (td, *J* = 11.2, 4.5 Hz, 1H). **^{13}C NMR** (150 MHz, $CDCl_3$) δ 206.32, 170.96, 154.05, 129.71, 127.58, 124.58, 118.04, 106.59, 75.83, 63.37, 60.75, 53.32, 45.44, 38.27, 34.80, 26.46, 21.55, 21.07. **FTIR** (film, cm^{-1}) 3544.52, 3460.63, 3052.76, 2953.45, 2925.48, 2829.06, 1781.90, 1738.51, 1605.45, 1492.63. **HRMS** (ESI) calcd. for C18H20BrNO3 (m/z M+H$^+$): 378.0705, 378.0697.

Ketone **16i** was not successfully isolated.

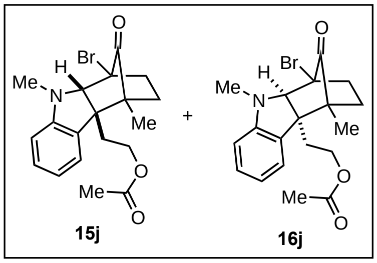

Following General Procedure F, a mixture of indole (100 mg, 0.460 mmol, 1.0 equiv), sodium carbonate (146 mg, 1.38 mmol, 3.0 equiv), and cyclopentanone **14a** (235 mg, 0.921 mmol, 2.0 equiv) in TFE (2.3 mL, 0.2 M) afforded ketone **15j** as a white solid that was found to be homogeneous as judged by ^{1}H NMR spectroscopy (138 mg, 77%). **^{1}H NMR** (600 MHz, $CDCl_3$) δ 7.15 (t, *J* = 1.3 Hz, 1H), 6.87 (d, *J* = 1.3 Hz, 1H), 6.65 (t, *J* = 1.0 Hz, 1H), 6.45 (d, *J* = 7.9 Hz, 1H), 3.96 (d, *J* = 1.9 Hz, 1H), 3.92 – 3.84 (m, 1H), 3.79 – 3.72 (m, 1H), 2.99 (s, 3H), 2.41 (td, *J* = 11.2, 4.6 Hz, 1H), 2.15 (ddd, *J* = 14.0, 8.5, 6.4 Hz, 1H), 1.96 – 1.87 (m, 4H), 1.64 (td, *J* = 11.2, 5.1 Hz, 1H), 1.61 – 1.57 (m, 1H), 1.48 (td, *J* = 12.8, 4.7 Hz, 1H), 1.17 (s, 3H). **^{13}C NMR** (150 MHz, $CDCl_3$) δ 208.25, 171.04, 153.87, 129.70, 127.76, 123.93, 117.97, 106.40, 75.28, 63.44, 61.07, 54.65, 45.73, 35.28, 34.68, 29.80, 27.32, 21.08, 12.21. **FTIR** (film, cm^{-1}) 3051.80, 2960.20, 2902.34, 2826.17, 1787.69, 1739.48, 1605.45. **HRMS** (ESI) calcd. for C19H22BrNO3 (m/z M+H$^+$): 392.0861, 392.0852.

Ketone **16j** was not successfully isolated.

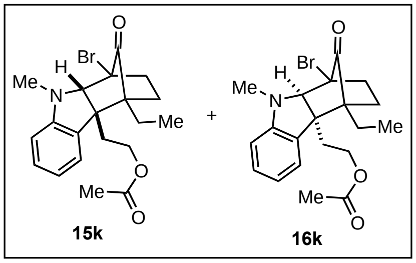

Following modified General Procedure F, a mixture of indole (100 mg, 0.460 mmol, 1.0 equiv), sodium carbonate (146 mg, 1.38 mmol, 3.0 equiv), and cyclopentanone **12a** (248 mg, 0.921 mmol, 2.0 equiv) in HFIP (2.3 mL, 0.2 M) afforded ketone **15k** as a colorless oil that was found to be homogeneous as judged by ^{1}H NMR spectroscopy (46 mg, 24%). **^{1}H NMR** (600 MHz, $CDCl_3$) δ 7.13 (t, *J* = 7.7 Hz, 1H), 6.88 (d, *J* = 7.3 Hz, 1H), 6.63 (t, *J* = 7.4 Hz, 1H), 6.44 (d, *J* = 7.9 Hz, 1H), 3.91 (s, 1H), 3.86 (ddd, *J* = 11.3, 8.7, 5.5 Hz, 1H), 3.72 (ddd, *J* = 11.0, 7.8, 6.0 Hz, 1H), 2.98 (s, 3H), 2.43 (td, *J* = 11.2, 4.2 Hz, 1H), 2.17 (ddd, *J* = 13.8, 8.7, 6.3 Hz, 1H), 1.95 – 1.86 (m, 5H), 1.80 (td, *J* = 12.8, 4.2 Hz, 1H), 1.63 – 1.56 (m, 1H), 1.56 – 1.50 (m, 1H), 1.46 (ddd, *J* = 12.9, 11.2, 5.1 Hz, 1H), 0.96 (t, *J* = 7.5 Hz, 3H). **^{13}C NMR** (150 MHz, $CDCl_3$) δ 207.87, 171.01, 154.05, 129.63, 127.69, 124.63, 117.88, 106.34, 75.25, 63.65, 61.13, 55.57, 49.18, 34.96, 34.67, 27.13, 24.23, 21.07, 18.55, 9.50. **FTIR** (film, cm^{-1}) 3052.76, 2967.91, 2883.06, 2828.10, 1779.97, 1739.48, 1603.52, 1492.63. **HRMS** (ESI) calcd. for C20H24BrNO3 (m/z M+H$^+$): 406.1018; found 406.1017.

Ketone **16k** was also isolated as a dark green oil that was found to be homogeneous as judged by ^{1}H NMR spectroscopy (20 mg, 11%).**^{1}H NMR** (600 MHz, $CDCl_3$) δ 7.07 (t, *J* = 7.7 Hz, 1H), 6.86 (d, *J* = 7.5 Hz, 1H), 6.64 (t, *J* = 7.5 Hz, 1H), 6.38 (d, *J* = 8.0 Hz, 1H), 3.84 (ddd, *J* = 11.2, 8.6, 6.1 Hz, 1H), 3.74 (ddd, *J* = 11.2,

8.3, 5.8 Hz, 1H), 3.58 (s, 1H), 2.95 (s, 3H), 2.35 – 2.20 (m, 3H), 2.15 – 2.07 (m, 2H), 2.00 – 1.92 (m, 4H), 1.71 (p, *J* = 7.5 Hz, 1H), 1.63 (p, *J* = 7.5 Hz, 1H). **^{13}C NMR** (150 MHz, $CDCl_3$) δ 207.01, 171.07, 153.25, 129.69, 127.15, 125.96, 118.31, 108.64, 78.19, 65.87, 61.53, 55.69, 48.44, 38.74, 34.26, 31.00, 30.04, 23.09, 21.29, 19.55, 9.98. **FTIR** (film, cm^{-1}) 3051.80, 2968.87, 2923.56, 1783.83, 1736.58, 1601.59, 1490.70. **HRMS** (ESI) calcd. for C20H24BrNO3 (m/z M+H$^+$): 406.1018; found 406.1012.

G. Debromination:

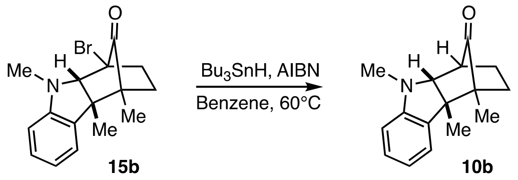

General Procedure G:

To a flame-dried 10 mL round bottom flask, bromo-ketone **15b** (100 mg, 0.312 mmol, 1.0 equiv) was dissolved in benzene (1.3 mL, 0.25 M) under an atmosphere of nitrogen. Bu_3SnH (0.100 mL, 0.375 mmol, 1.2 equiv) and AIBN (0.005 g, 0.031 mmol, 0.10 equiv) were added sequentially and then the mixture was heated to 60 °C for 20 h. When the reaction was judged complete as determined by thin-layer chromatographic analysis the reaction mixture was cooled to room temperature and the solvent was removed in *vacuo*. The residue was purified via silica gel flash column chromatography (hexanes/EtOAc) to afford debrominated ketone **10b** as a white solid that was found to be homogeneous as judged by ^{1}H NMR spectroscopy (66 mg, 87%).

^{1}H NMR (600 MHz, $CDCl_3$) δ 7.11 (t, *J* = 7.6 Hz, 1H), 6.91 (d, *J* = 6.3 Hz, 1H), 6.58 (t, *J* = 7.3 Hz, 1H), 6.36 (d, *J* = 7.8 Hz, 1H), 3.58 (d, *J* = 1.5 Hz, 1H), 2.78 (s, 3H), 2.32 (t, *J* = 4.7 Hz, 1H), 1.78 (td, *J* = 11.0, 4.5 Hz, 1H), 1.67 – 1.59 (m, 1H), 1.45 (td, *J* = 11.1, 5.2 Hz, 1H), 1.38 – 1.33 (m, 1H), 1.21 (s, 3H), 1.07 (s, 3H). **^{13}C NMR** (150 MHz, $CDCl_3$) δ 215.98, 153.57, 131.85, 128.82, 124.15, 116.65, 104.86, 71.65, 49.59, 48.89, 44.28, 33.04, 28.17, 26.05, 17.28, 11.64. **FTIR** (film, cm^{-1}) 2958.27, 2928.38, 2867.63, 1768.40, 1604.48, 1493.60. **HRMS** (ESI) calcd. for C16H19NO (m/z M+H$^+$): 242.1545; found 242.1539.

Computational Studies

Initial DFT calculations were carried out using the B3LYP-D3 functional,[1] combined with the Grimme D3 dispersion correction,[2] and the 6-311G** basis set,[3] to obtain optimized structures; for molecules containing bromine, the Los Alamos core potential was used.[4] Final zero point energy corrected free energies were obtained by single point calculations using B3LYP-D3 and the def2-tzvp basis,[5] with an implicit Poisson-Boltzmann[6] solvent model for trifluoroethanol (TFE), as implemented in the Jaguar suite of programs.[7] Computed structures were confirmed as energy minima or transition structures by calculating the vibrational frequencies using second derivative analytic methods and confirming a single imaginary frequency for first order saddle points, and the absence of imaginary frequencies for minima. SCFE energies and corrected free energies are compiled in Table S1.

Energy Decomposition Analyses (EDA),[8] and Natural Orbitals for Chemical Valence (NOCV).[9] calculations were carried out on transition structures (**TS2**) using B3LYP-D3 and the triple zeta basis TZVP, as implemented in the ADF suite of programs.[10] Results are compiled in Table S2.

XYZ coordinate files of all calculated structures are provided at the end of this section

References

(1) (a) Lee, C.; Yang, W.; Parr, R. G. Development of the Colle-Salvetti correlation-energy formula into a functional of the electron density. *Phys. Rev. B* **1988**, *37* (2), 785-789. From SciFinder CAPLUS. (b) Becke, A. D. Density-functional thermochemistry. III. The role of exact exchange. *J. Chem. Phys.* **1993**, *98* (7), 5648-5652. (c) Becke, A. D. A new mixing of Hartree-Fock and local-density-functional theories. *J. Chem. Phys.* **1993**, *98* (2), 1372-1377. (d) Stephens, P. J.; Devlin, F. J.; Chabalowski, C. F.; Frisch, M. J. Ab Initio Calculation of Vibrational Absorption and Circular Dichroism Spectra Using Density Functional Force Fields. *J. Phys. Chem.* **1994**, *98* (45), 11623-11627. From SciFinder CAPLUS. (e) Andersson, M. P.; Uvdal, P. New Scale Factors for Harmonic Vibrational Frequencies Using the B3LYP Density Functional Method with the Triple-ζ Basis Set 6-311+G(d,p). *J. Phys. Chem. A* **2005**, *109* (12), 2937-2941, doi: 10.1021/jp045733a. DOI: 10.1021/jp045733a.

(2) (a) Goerigk, L.; Grimme, S. A thorough benchmark of density functional methods for general main group thermochemistry, kinetics, and noncovalent interactions. *Phys. Chem. Chem. Phys.* **2011**, *13* (14), 6670-6688, 10.1039/c0cp02984j. DOI: 10.1039/c0cp02984j. (b) Grimme, S.; Antony, J.; Ehrlich, S.; Krieg, H. A consistent and accurate ab initio parametrization of density functional dispersion correction (DFT-D) for the 94 elements H-Pu. *J. Chem. Phys.* **2010**, *132* (15), 154104.

(3) (a) Frisch, M. J.; Pople, J. A.; Binkley, J. S. Self-consistent molecular orbital methods. 25. Supplementary functions for Gaussian basis sets. *J. Chem. Phys.* **1984**, *80* (7), 3265-3269. (b) Clark, T.; Chandrasekhar, J.; Spitznagel, G. W.; Schleyer, P. v. R. Efficient diffuse function-augmented basis sets for anion calculations. III. The 3-21 + G basis set for first-row elements, lithium to fluorine. *J. Comput. Chem.* **1983**, *4*, 294-301, 10.1002/jcc.540040303. DOI: 10.1002/jcc.540040303. (c) McLean, A. D.; Chandler, G. S. Contracted Gaussian basis sets for molecular calculations. I. Second row atoms, Z = 11-18. *J, Chem. Phys.* **1980**, *72*, 5639-5648, 10.1063/1.438980. DOI: 10.1063/1.438980. (d) Krishnan, R.; Binkley, J. S.; Seeger, R.; Pople, J. A. Self-consistent molecular orbital methods. XX. A basis set for correlated wave functions. *J. Chem. Phys.* **1980**, *72* (1), 650-654.

(4) (a) Wadt, W. R.; Hay, P. J. Ab initio effective core potentials for molecular calculations. Potentials for main group elements sodium to bismuth. *J. Chem. Phys.* **1985**, *82* (1), 284-298. (b) Hay, P. J.; Wadt, W. R. Ab initio effective core potentials for molecular calculations. Potentials for potassium to gold including the outermost core orbitals. *J. Chem. Phys.* **1985**, *82* (1), 299-310. (c) Hay, P. J.; Wadt, W. R. Ab initio effective core potentials for molecular calculations. Potentials for the transition metal atoms scandium to mercury. *J. Chem. Phys.* **1985**, *82* (1), 270-283. (d) Dunning, T. H.; Hay, P. J. Modern Theoretical Chemistry, Vol. 4: Applications of Electronic Structure Theory. Schaefer, H. F., III (Editor), Ed.; Plenum, NY: 1977; p 461.

(5) (a) Schaefer, A.; Huber, C.; Ahlrichs, R. Fully optimized contracted Gaussian basis sets of triple zeta valence quality for atoms Li to Kr. *J. Chem. Phys.* **1994**, *100* (8), 5829-5835, 10.1063/1.467146. DOI: 10.1063/1.467146. (b) Schaefer, A.; Horn, H.; Ahlrichs, R. Fully optimized contracted Gaussian basis sets for atoms lithium to krypton. *J. Chem. Phys.* **1992**, *97* (4), 2571-2577, 10.1063/1.463096. DOI: 10.1063/1.463096.

(6) (a) Marten, B.; Kim, K.; Cortis, C.; Friesner, R. A.; Murphy, R. B.; Ringnalda, M. N.; Sitkoff, D.; Honig, B. A New Model For Calculation of Solvation Free Energies: Correction of Self-Consistent Reaction Field Continuum Dielectric Theory for Short Range Hydrogen-Bonding Effects. *J. Phys. Chem.* **1996**, *100* (28), 11775-11788. From SciFinder CAPLUS. (b) Tannor, D. J.; Marten, B.; Murphy, R.; Friesner, R. A.; Sitkoff, D.; Nicholls, A.; Honig, B.; Ringnalda, M.; Goddard, W. A. Accurate First Principles Calculation of Molecular Charge Distributions and Solvation Energies from Ab Initio Quantum Mechanics and Continuum Dielectric Theory. *J. Am. Chem. Soc.* **1994**, *116* (26), 11875-11882. DOI: 10.1021/ja00105a030 (acccessed 2013/11/29).

(7) (a) *Jaguar, versions 7.0-10.2*; Schrödinger, LLC, New York, NY: 2007-2020. (accessed. (b) Bochevarov, A. D.; Harder, E.; Hughes, T. F.; Greenwood, J. R.; Braden, D. A.; Philipp, D. M.; Rinaldo, D.; Halls, M. D.; Zhang, J.; Friesner, R. A. Jaguar: A high-performance quantum chemistry software program with strengths in life and materials sciences. *Int. J. Quantum Chem.* **2013**, *113* (18), 2110-2142. DOI: 10.1002/qua.24481.

(8) (a) Andrés, J.; Ayers, P. W.; Boto, R. A.; Carbó-Dorca, R.; Chermette, H.; Cioslowski, J.; Contreras-García, J.; Cooper, D. L.; Frenking, G.; Gatti, C.; et al. Nine questions on energy decomposition analysis. *J. Comput. Chem.* **2019**, *40* (26), 2248-2283. DOI: 10.1002/jcc.26003. (b) Frenking, G.; Bickelhaupt, F. M. The EDA perspective of chemical bonding. In *The Chemical Bond*, Frenking, G., Shaik, S. Eds.; Vol. 1; Wiley-VCH Verlag GmbH & Co., 2014; pp 121-157. (c) Hopffgarten, M. v.; Frenking, G. Energy decomposition analysis. *Wiley Interdisciplinary Reviews: Computational Molecular Science* **2012**, *2* (1), 43-62. DOI: 10.1002/wcms.71.

(9) (a) Mitoraj, M. P.; Parafiniuk, M.; Srebro, M.; Handzlik, M.; Buczek, A.; Michalak, A. Applications of the ETS-NOCV method in descriptions of chemical reactions. *J Mol Model* **2011**, *17* (9), 2337-2352. (b) Mitoraj, M. P.; Michalak, A.; Ziegler, T. A Combined Charge and Energy Decomposition Scheme for Bond Analysis. *J. Chem. Theory Comput.* **2009**, *5* (4), 962-975, doi: 10.1021/ct800503d. DOI: 10.1021/ct800503d. (c) Mitoraj, M.; Michalak, A. Applications of natural orbitals for chemical valence in a description of bonding in conjugated molecules. *J. Mol. Model.* **2008**, *14* (Copyright (C) 2011 American Chemical Society (ACS). All Rights Reserved.), 681-687, 10.1007/s00894-008-0276-1. DOI: 10.1007/s00894-008-0276-1. (d) Mitoraj, M.; Michalak, A. Natural orbitals for chemical valence as descriptors of chemical bonding in transition metal complexes. *J. Mol. Model.* **2007**, *13* (Copyright (C) 2011 American Chemical Society (ACS). All Rights Reserved.), 347-355, 10.1007/s00894-006-0149-4. DOI: 10.1007/s00894-006-0149-4.

(10) (a) *ADF 2010-2016*; SCM, Theoretical Chemistry, Vrije Universiteit, Amsterdam, The Netherlands, http://www.scm.com: (accessed. (b) Guerra, C. F.; Snijders, J. G.; Velde, G. t.; Baerends, E. J. Towards an order-N DFT method. *Theoretical Chemistry Accounts* **1998**, *99*, 391-403. (c) Bickelhaupt, F. M.; Baerends, E. J. Kohn-Sham density functional theory: predicting and understanding chemistry. *Rev. Comput. Chem.* **2000**, *15*, 1-86. From SciFinder CAPLUS.

Table S1. EDA results [SCF Energies (kcal/mol)] for the rate limiting transition states (**TS2**) in reactions of oxyallyl cations with dimethylindole.

Entry								
	**7 (R^2=CH_3)**							
	**TS2**	**E_{prep}**	**E_{int}**	**E_{steric}**	**E_{orb}**	**E_{disp}**	**E_{tot}[b]**	**ΔE_{tot}[c]**
**1**	obs major	+24.7	-30.9	+59.1	-75.9	-14.0	-6.2	0.0
	obs minor	+20.2	-23.3	+52.4	-65.9	-9.9	-3.1	+3.1
	n/o major[a]	+33.5	-35.3	+58.7	-79.8	-14.2	-1.8	+4.6
	n/o minor[a]	+27.8	-28.0	+51.9	-67.6	-12.3	-0.2	+5.9
	**11 (R^2=H)**							
**2**	obs major	+23.0	-33.3	+53.1	-71.4	-15.0	-10.3	0
	obs minor	+18.0	-24.3	+45.7	-59.9	-10.2	-6.3	+4.0
	n/o major[a]	+31.9	-34.9	+54.0	-74.2	-14.8	-3.0	+7.3
	n/o minor[a]	+27.7	-29.8	+48.2	-64.7	-13.2	-2.0	+8.3
	**11 (R^2=CH_3)**							
**3**	obs major	+34.6	-37.4	+63.7	-84.2	-16.8	-2.7	0.0
	obs minor	+29.6	-29.0	+54.7	-71.0	-12.7	+0.6	+3.3
	n/o major[a]	+34.4	-34.2	+63.1	-81.1	-16.2	+0.2	+2.9
	n/o minor[a]	+30.9	-28.6	+56.4	-71.6	-13.4	+2.3	+5.0

a. n/o = not observed
b. E_{tot} = E_{int}+E_{prep}
c. E_{tot} – E_{tot} (major isomer)

Table S2. DFT Calculated Energies (B3LYP-D3/def2-tzvp/solv//B3LYP-D3/LACV3P**)

	**Total Free Energy G (au) at 298.15K 1.0atm**	**G(kcal/mol)**	**G(relative) kcal/mol)**	**SCFE (au)**
	**REACTANTS**			
**8a**	-442.471681	-277655.2	-	-442.613447
**7 (R^2=CH_3)**	-308.658514	-193686.1	-	-308.724961
**11 (R^2=H)**	-2842.937304	-1783970.2	-	-2842.968493
**11 (R^2=CH_3)**	-2882.256559	-1808643.4	-	-2882.314165
	**REACTIONS**			
**8a+7 (R^2=CH_3)**	-751.130195	-471341.3	**0.0**	-751.338408
	**OBSERVED REGIOISOMER: MAJOR STEREOISOMER**			
**9a**	-751.148635	-471352.9	-11.6	-751.411250
**TS1**	-751.093003	-471318.0	23.3	-751.327864
**TS2**	-751.099806	-471322.3	19.1	-751.348850
**TS3**	-751.092974	-471318.0	23.4	-751.331887
**TS4**	-751.120291	-471335.1	6.2	-751.378747
**INT2**	-751.132247	-471342.6	-1.3	-751.398522
**INT3**	-751.099205	-471321.9	19.4	-751.335640
	**OBSERVED REGIOISOMER: MINOR STEREOISOMER**			
**13a**	-751.147506	-471352.2	-10.9	-751.408910
**TS1'**	-751.091051	-471316.8	24.6	-751.332221
**TS2'**	-751.099061	-471321.8	19.5	-751.344380
**TS3'**	-751.095110	-471319.3	22.0	-751.332934
**INT2'**	-751.130197	-471341.3	0.0	-751.396354
**INT3'**	-751.101074	-471323.1	18.3	-751.335152
	**UNOBSERVED REGIOISOMER: MAJOR STEREOISOMER**			
**10**	-751.147391	-471352.1	-10.8	-751.410343
**TS1**	-751.089962	-471316.1	25.2	-751.322997
**TS2**	-751.092200	-471317.5	23.8	-751.341942
**TS4**	-751.114581	-471331.5	9.8	
**INT2**	-751.124831	-471338.0	3.4	-751.391713
	**UNOBSERVED REGIOISOMER: MINOR STEREOISOMER**			
**TS2**	-751.094007	-471318.6	22.7	-751.339112
**8a+11 (R^2=H)**	-3285.408985	-2061625.3	**0.0**	
	**OBSERVED REGIOISOMER: MAJOR STEREOISOMER**			
**TS2**	-3285.385390	-2061610.5	14.8	-3285.599078
**15a**	-3285.431452	-2061639.4	-14.1	-3285.658635
**INT2**	-3285.414332	-2061628.7	-3.4	-3285.644450
**TS4**	-3285.400378	-2061619.9	5.4	**-3285.622816**
	**OBSERVED REGIOISOMER: MINOR STEREOISOMER**			
**16a**	-3285.429406	-2061638.2	-12.8	-3285.654281
**INT2**	-3285.411867	-2061627.2	-1.8	-3285.641947
**TS2**	-3285.384462	-2061610.0	15.4	-3285.592655

	**UNOBSERVED REGIOISOMER: MAJOR STEREOISOMER**			
**TS2**	-3285.374088	-2061603.5	21.9	-3285.587182
**INT2**	-3285.412974	-2061627.9	-2.5	-3285.641222
	-3285.428812	-2061637.8	-12.4	-3285.653343
	**UNOBSERVED REGIOISOMER: MINOR STEREOISOMER**			
**TS2**	-3285.376075	-2061604.7	20.7	-3285.585150

**8a+11(R^2=CH_3)**	-3324.728240	-2086298.6	**0.0**	
	**OBSERVED REGIOISOMER: MAJOR STEREOISOMER**			
**15b**	-3324.739507	-2086305.6	-7.1	-3324.993588
**TS1**	-3324.681339	-2086269.1	29.4	-3324.911257
**TS2**	-3324.691708	-2086275.6	22.9	-3324.932780
**TS3**	-3324.686997	-2086272.7	25.9	-3324.916154
**INT2**	-3324.716448	-2086291.2	7.4	-3324.973904
**INT3**	-3324.695141	-2086277.8	20.8	-3324.919021
	**OBSERVED REGIOISOMER: MINOR STEREOISOMER**			
**15b(minor stereoisomer)**	-3324.737079	-2086304.1	-5.5	-3324.989303
**TS1**	-3324.680453	-2086268.6	30.0	-3324.915182
**TS2**	-3324.690782	-2086275.1	23.5	-3324.927023
**INT2**	-3324.714534	-2086290.0	8.6	-3324.972144
	**UNOBSERVED REGIOISOMER: MAJOR STEREOISOMER**			
**TS2**	-3324.687311	-2086272.9	25.7	-3324.927662
	-3324.739298	-2086305.5	-6.9	-3324.993008
	-3324.730006	-2086299.7	-1.1	-3324.984799
	**UNOBSERVED REGIOISOMER: MINOR STEREOISOMER**			
**TS2**	-3324.687740	-2086273.1	25.4	-3324.924486
**INT2**	-3324.724499	-2086296.2	2.3	-3324.978455

XYZ coordinate files for DFT calculated structures.

Reactants

Dimethylindole (8a)

```
22
DIMETHYL_INDOLE_DEF2_TZVP_SP_TRIFLUOROETHANOL
C         -1.38820        1.28380        0.40260
C         -2.72440        1.53860        0.74190
C         -3.72270        0.74760        0.19090
C         -3.41180       -0.29940       -0.69680
C         -2.09840       -0.57630       -1.05340
C         -1.09640        0.22440       -0.49960
C         -0.13480        1.88320        0.77540
C          0.83420        1.18550        0.10520
N          0.26970        0.18660       -0.67360
H         -2.97240        2.34260        1.42640
H         -4.75950        0.93500        0.44640
H         -4.21280       -0.90200       -1.10970
H         -1.86320       -1.38560       -1.73520
H          1.90520        1.32400        0.11310
C          0.98590       -0.77980       -1.47800
H          2.03510       -0.49020       -1.53670
H          0.92270       -1.78450       -1.04650
H          0.57980       -0.80970       -2.49310
C          0.05890        3.04040        1.70460
H          1.11770        3.28710        1.81350
H         -0.45680        3.93610        1.34150
H         -0.33740        2.82350        2.70260
```

7 (R^2=CH_3)

```
15
METHYL_OXALLYL_DEF2_TZVP_SP_TRIFLUOROETHANOL
C         -0.47970        4.65530       -1.09560
C          0.98240        3.58000       -2.44040
C         -1.32730        4.08740       -2.16330
H         -2.09000        3.40340       -1.76470
C          0.92140        4.39580       -1.23590
O          1.88880        4.74500       -0.54460
H         -1.88840        4.86220       -2.70540
C         -0.32710        3.35710       -3.07820
H         -0.54890        2.28450       -3.16920
H         -0.34310        3.74300       -4.10780
H         -0.88130        5.23760       -0.27290
C          2.27730        3.08960       -2.93650
H          2.19840        2.20970       -3.57790
```

```
H          2.75880        3.89230       -3.51600
H          2.94130        2.91640       -2.08350
```

11 (R^2=H)

```
12
BROMO_H_OXALLYL
C         -0.48270        4.67540       -1.08690
C          0.97810        3.60700       -2.37960
C         -1.33770        4.10670       -2.14450
H         -2.10050        3.43600       -1.73060
C          0.94900        4.40450       -1.18370
O          1.85730        4.76520       -0.44140
H         -1.88260        4.88920       -2.68690
C         -0.32200        3.36850       -3.03570
H         -0.55160        2.29650       -3.11350
H         -0.33530        3.74630       -4.06780
Br        -1.18850        5.70840        0.32070
H          1.91150        3.21340       -2.76890
```

11 (R^2=CH_3)

```
15
BROMO_METHYL_OXALLYL_DEF2_TZVP_SP_TRIFLUOROETHANOL
C         -0.47660        4.67410       -1.08300
C          0.98690        3.59840       -2.38420
C         -1.33610        4.11240       -2.14630
H         -2.10180        3.44230       -1.73760
C          0.93550        4.41050       -1.18830
O          1.87920        4.75970       -0.47540
H         -1.87400        4.89750       -2.69150
C         -0.32110        3.37120       -3.03640
H         -0.54640        2.29860       -3.11220
H         -0.32620        3.74750       -4.06890
Br        -1.19410        5.70850        0.32880
C          2.27670        3.09250       -2.88710
H          2.18390        2.13820       -3.41190
H          2.69600        3.81910       -3.59970
H          2.98790        3.02920       -2.05960
```

Reaction 8a + 7 ($R^2 = CH_3$): observed regioisomer; major stereoisomer

9a

```
37
REACTION1_A_OBS_MAJOR_DEF2_TZVP_SP_TRIFLUOROETHANOL
C        -1.49510        1.93940       -0.05380
C        -2.71040        2.04080        0.59830
C        -3.63470        0.99260        0.50170
C        -3.31400       -0.14390       -0.23670
C        -2.09000       -0.25890       -0.90170
C        -1.18770        0.80260       -0.81830
C        -0.32070        2.89110       -0.05960
C         0.72970        2.13540       -0.97900
N         0.05160        0.92340       -1.43900
H        -2.94210        2.92220        1.18740
H        -4.59010        1.06170        1.00760
H        -4.02510       -0.96010       -0.30000
H        -1.85780       -1.15070       -1.47080
H         1.63100        1.87450       -0.40880
C         0.82950       -0.24070       -1.80830
H         1.72260        0.07740       -2.34610
H         1.13960       -0.83540       -0.93440
H         0.25600       -0.88630       -2.47760
C         0.21770        3.14410        1.35520
H         1.10090        3.78950        1.33410
H        -0.54260        3.63240        1.97070
H         0.48740        2.20050        1.83550
C        -0.52330        4.24070       -0.81660
C         1.10550        3.16470       -2.09200
C        -1.20880        4.01120       -2.18100
H        -2.10480        3.39850       -2.08100
C         0.92020        4.46020       -1.27850
O         1.69460        5.35570       -1.07710
H        -1.50330        4.96710       -2.61910
C        -0.10300        3.31110       -3.04720
H        -0.42320        2.33800       -3.41870
H         0.17910        3.93040       -3.90210
C         2.46000        2.98940       -2.74730
H         2.48340        2.10530       -3.39040
H         2.70030        3.86060       -3.36190
H         3.24780        2.89080       -1.99530
H        -0.94360        5.03610       -0.20440
```

TS1

```
37
REACTION1_A_OBS_MAJOR_DEF2_TZVP_SP_TRIFLUOROETHANOL
```

```
C          -1.49510        1.93940       -0.05380
C          -2.71040        2.04080        0.59830
C          -3.63470        0.99260        0.50170
C          -3.31400       -0.14390       -0.23670
C          -2.09000       -0.25890       -0.90170
C          -1.18770        0.80260       -0.81830
C          -0.32070        2.89110       -0.05960
C           0.72970        2.13540       -0.97900
N           0.05160        0.92340       -1.43900
H          -2.94210        2.92220        1.18740
H          -4.59010        1.06170        1.00760
H          -4.02510       -0.96010       -0.30000
H          -1.85780       -1.15070       -1.47080
H           1.63100        1.87450       -0.40880
C           0.82950       -0.24070       -1.80830
H           1.72260        0.07740       -2.34610
H           1.13960       -0.83540       -0.93440
H           0.25600       -0.88630       -2.47760
C           0.21770        3.14410        1.35520
H           1.10090        3.78950        1.33410
H          -0.54260        3.63240        1.97070
H           0.48740        2.20050        1.83550
C          -0.52330        4.24070       -0.81660
C           1.10550        3.16470       -2.09200
C          -1.20880        4.01120       -2.18100
H          -2.10480        3.39850       -2.08100
C           0.92020        4.46020       -1.27850
O           1.69460        5.35570       -1.07710
H          -1.50330        4.96710       -2.61910
C          -0.10300        3.31110       -3.04720
H          -0.42320        2.33800       -3.41870
H           0.17910        3.93040       -3.90210
C           2.46000        2.98940       -2.74730
H           2.48340        2.10530       -3.39040
H           2.70030        3.86060       -3.36190
H           3.24780        2.89080       -1.99530
H          -0.94360        5.03610       -0.20440
```

TS2

```
37
REACTION1_A_OBS_MAJOR_O_BOUND_TS_DEF2_TZVP_SP_TRIFLUOROETHANOL(O
BS_MAJOR)
C          -1.05890        3.26380        0.22790
C          -2.34910        2.76890        0.06790
C          -2.52720        1.55650       -0.59890
C          -1.42940        0.85530       -1.11120
```

```
C         -0.12570        1.32050       -0.93680
C          0.03570        2.51880       -0.25120
C         -0.52280        4.51450        0.78840
C          0.89060        4.29080        0.83550
N          1.20690        3.18630        0.14040
H         -3.20170        3.31490        0.45510
H         -3.52510        1.15470       -0.72830
H         -1.59050       -0.07840       -1.63730
H          0.72020        0.75870       -1.31260
H          1.65640        4.94120        1.21950
C          2.56340        2.86550       -0.28720
H          3.12530        2.39360        0.52210
H          2.52000        2.18800       -1.13800
H          3.02700        3.80230       -0.60260
C         -1.26830        5.29360        1.84500
H         -0.70340        6.17510        2.15580
H         -2.23530        5.63230        1.46370
H         -1.45400        4.67570        2.72810
C         -0.51380        5.78370       -0.80090
C          0.39030        4.71380       -2.63150
C         -1.67960        5.50240       -1.71120
H         -2.54750        5.06970       -1.20720
C          0.73870        5.48260       -1.51880
O          1.88680        5.76070       -1.05610
H         -2.01270        6.44770       -2.15450
C         -1.08850        4.57610       -2.80020
H         -1.40400        3.53270       -2.65970
H         -1.42990        4.86660       -3.80300
C          1.37830        4.10930       -3.56140
H          1.25440        3.01730       -3.61180
H          1.25980        4.47930       -4.58830
H          2.39120        4.34190       -3.22580
H         -0.53300        6.67010       -0.17440
```

TS3

```
37
REACTION1_A_OBS_MAJOR_INT_TS_SP_TFE_DEF2_TZVP
C         -1.21920        1.64970        0.29260
C         -2.40200        1.69160        1.02850
C         -3.46630        0.89510        0.61540
C         -3.37610        0.08520       -0.52710
C         -2.20920        0.03350       -1.28160
C         -1.14440        0.81560       -0.83890
C          0.06400        2.37960        0.39590
C          0.86850        1.77860       -0.61240
N          0.14610        0.93160       -1.37210
```

```
H         -2.49770        2.37210        1.86110
H         -4.39370        0.91770        1.17520
H         -4.23080       -0.50880       -0.82950
H         -2.13570       -0.58680       -2.16670
H          1.90740        1.96420       -0.83630
C          0.59870        0.25280       -2.57360
H          1.65210        0.47450       -2.73800
H          0.47320       -0.82690       -2.46200
H          0.02260        0.58780       -3.44000
C          0.65230        2.78570        1.72820
H          0.87990        1.90010        2.32810
H          1.57200        3.35820        1.59000
H         -0.05530        3.40920        2.27440
C         -0.35830        4.10100       -0.51280
C         -0.20360        5.92050        0.91320
C          0.88280        4.86410       -0.92810
H          1.76690        4.23730       -1.07380
C         -1.06440        4.86410        0.55330
O         -2.18790        4.57350        1.02550
H          0.68960        5.35080       -1.89000
C          1.08460        5.93540        0.16750
H          1.94550        5.70260        0.81500
H          1.30630        6.91880       -0.27100
C         -0.60930        6.97430        1.87800
H          0.12470        7.07950        2.68930
H         -0.68610        7.96260        1.40270
H         -1.58020        6.71580        2.30590
H         -0.97210        3.66330       -1.29460
```

TS4

```
37
REACTION1_A_MAJOR_SECOND_TS_BS_HESSREF_REDONE_SP_TFE_DEF2_TZVP
C         -1.59140        2.07310       -0.06780
C         -2.85490        2.25320        0.45740
C         -3.82060        1.25160        0.27250
C         -3.49140        0.08480       -0.41100
C         -2.21390       -0.10870       -0.95590
C         -1.27370        0.89940       -0.77170
C         -0.37360        2.96280       -0.05860
C          0.69460        2.12740       -0.77740
N          0.06990        0.91920       -1.15500
H         -3.09680        3.14800        1.02150
H         -4.80230        1.35470        0.71790
H         -4.23820       -0.69250       -0.52980
H         -1.96940       -1.02110       -1.48560
H          1.68470        2.00190       -0.33530
```

C	0.74610	-0.18060	-1.80140
H	1.82480	-0.01870	-1.76110
H	0.51060	-1.11820	-1.28900
H	0.46130	-0.28070	-2.85580
C	0.04980	3.26610	1.42100
H	0.93860	3.90130	1.43810
H	-0.76550	3.78250	1.93160
H	0.25030	2.32750	1.94080
C	-0.47090	4.28880	-0.85940
C	1.08340	3.32970	-2.29870
C	-1.22070	4.13950	-2.11750
H	-2.19710	3.66700	-1.98940
C	0.98850	4.45560	-1.34220
O	1.86160	5.16070	-0.89080
H	-1.45500	5.12030	-2.54890
C	-0.24990	3.33540	-3.06180
H	-0.58890	2.30250	-3.20200
H	-0.16460	3.79980	-4.04710
H	-0.83990	5.07910	-0.20450
C	2.36700	2.98990	-2.98430
H	2.36360	1.99570	-3.44300
H	2.59390	3.70300	-3.78720
H	3.19790	3.05380	-2.27450

INT2

37
REACTION1_A_OBS_MAJOR_O_BOUND_DEF2_TZVP_SP_TRIFLUOROETHANOL

C	-1.50150	1.95150	-0.07120
C	-2.70790	2.04020	0.60060
C	-3.62710	0.98600	0.51090
C	-3.31860	-0.14080	-0.24610
C	-2.09280	-0.25660	-0.90900
C	-1.18390	0.79230	-0.79550
C	-0.32790	2.91410	-0.12070
C	0.59870	2.20870	-1.18320
N	0.12200	0.86280	-1.27860
H	-2.94460	2.91850	1.19080
H	-4.57850	1.05250	1.02470
H	-4.03790	-0.94860	-0.32350
H	-1.86100	-1.14180	-1.48820
H	1.66690	2.23550	-0.95690
C	0.62290	-0.01850	-2.31330
H	1.69260	0.15580	-2.44150
H	0.48260	-1.06040	-2.01560
H	0.12760	0.14750	-3.27810
C	0.34500	3.03780	1.25080

H	1.24460	3.65800	1.19120
H	-0.34100	3.49890	1.96580
H	0.62310	2.05440	1.63790
C	-0.60000	4.28070	-0.80850
C	-1.20550	4.64300	-3.08970
C	-1.95020	5.02130	-0.81390
H	-2.76260	4.30510	-0.68620
C	-0.43580	3.94530	-2.25610
O	0.44300	2.92620	-2.45530
H	-2.03400	5.78190	-0.03420
C	-2.03490	5.60880	-2.25570
H	-3.07000	5.67370	-2.60900
H	-1.62640	6.62900	-2.29550
C	-1.28050	4.54510	-4.57740
H	-2.30850	4.35590	-4.90870
H	-0.95830	5.47550	-5.06140
H	-0.64630	3.73640	-4.94720
H	0.20230	4.96640	-0.49710

INT3

37
REACTION1_A_OBS_INT_MAJOR_SP_TFE_DEF2_TZVP

C	-1.31130	1.74880	0.18020
C	-2.47290	1.83480	0.93680
C	-3.53680	0.98850	0.61830
C	-3.45800	0.08290	-0.44610
C	-2.29990	-0.02520	-1.21140
C	-1.24560	0.81210	-0.85930
C	-0.00490	2.51280	0.21750
C	0.78910	1.76440	-0.76810
N	0.04860	0.87270	-1.41990
H	-2.56120	2.57940	1.71350
H	-4.45090	1.04820	1.19660
H	-4.30870	-0.54730	-0.67790
H	-2.22840	-0.72760	-2.03260
H	1.81890	1.93250	-1.04220
C	0.47320	0.06220	-2.55080
H	1.51960	0.26700	-2.76980
H	0.35220	-0.99650	-2.31110
H	-0.13460	0.30000	-3.42680
C	0.65010	2.64560	1.59570
H	1.64740	3.08180	1.51060
H	0.04770	3.31330	2.21470
H	0.73310	1.67040	2.08070
C	-0.21620	4.00270	-0.46650
C	-0.20840	5.84440	1.02780

```
C          1.08970        4.78600       -0.68620
H          1.98370        4.15820       -0.75730
C         -1.04990        4.86550        0.50010
O         -2.26180        4.63820        0.72360
H          1.01470        5.32820       -1.63200
C          1.18130        5.80090        0.48210
H          1.92470        5.48760        1.23400
H          1.52460        6.78300        0.12550
C         -0.70280        6.92920        1.92050
H         -0.10950        6.99760        2.84370
H         -0.64810        7.92060        1.44620
H         -1.74420        6.73380        2.18640
H         -0.77260        3.81080       -1.38590
```

Reaction 8a + 7 (R^2 = CH_3): observed regioisomer; minor stereoisomer

13a

```
37
REACTION1_A_OBS_MINOR_SP_TFE_DEF2-TZVP
C         -1.39200        1.54270       -0.10610
C         -2.65840        1.70860        0.42630
C         -3.64000        0.74160        0.18070
C         -3.32670       -0.38040       -0.58370
C         -2.05350       -0.55620       -1.13030
C         -1.09070        0.42740       -0.89940
C         -0.15440        2.40080        0.07920
C          0.90530        1.65550       -0.84130
N          0.21270        0.48320       -1.38700
H         -2.88530        2.57570        1.03830
H         -4.63590        0.86090        0.58980
H         -4.08530       -1.13400       -0.76440
H         -1.83030       -1.43160       -1.72760
H          1.79420        1.34620       -0.27700
C          0.96630       -0.72780       -1.64550
H          1.91450       -0.47250       -2.11860
H          1.17320       -1.29970       -0.72680
H          0.42080       -1.37260       -2.33720
C          0.25490        2.44820        1.55520
H          1.22760        2.92490        1.69740
H         -0.48580        2.99360        2.14690
H          0.31780        1.43190        1.95170
C         -0.30020        3.79420       -0.60370
C          1.28340        2.70790       -1.92360
C         -0.08220        3.40510       -2.07050
C          1.89890        2.18650       -3.20320
H          1.25690        1.42550       -3.65020
H          2.00480        2.99830       -3.92710
```

H	2.88930	1.75540	-3.02620
H	-1.24000	4.29920	-0.39160
O	-0.73770	3.61070	-3.05310
C	0.97620	4.63950	-0.40640
H	0.83190	5.62820	-0.84900
H	1.23150	4.78970	0.64230
C	2.06710	3.83400	-1.19430
H	2.82890	3.41200	-0.53220
H	2.58080	4.46870	-1.91920

TS1'

37
REACTION1_A_OBS_MINOR_TS_BS_SP_TFE_DEF2_TZVP(DIRECT)

C	-1.28190	1.46920	0.13020
C	-2.65050	1.67070	0.26430
C	-3.51200	0.94290	-0.54920
C	-3.01820	0.05090	-1.50950
C	-1.65190	-0.15770	-1.66610
C	-0.80900	0.55030	-0.82000
C	-0.10020	2.11030	0.73940
C	0.99180	1.30190	0.29690
N	0.59270	0.52480	-0.72630
H	-3.03730	2.39150	0.97450
H	-4.58220	1.08530	-0.45880
H	-3.71230	-0.48350	-2.14730
H	-1.26950	-0.83810	-2.41670
H	2.03260	1.36960	0.56980
C	1.44530	-0.31600	-1.54690
H	2.48480	-0.02940	-1.39360
H	1.31430	-1.36950	-1.28560
H	1.19220	-0.17050	-2.59810
C	-0.13180	2.71350	2.12390
H	0.79240	3.25360	2.34290
H	-0.96100	3.41750	2.21880
H	-0.26340	1.93760	2.88270
C	0.08540	3.70840	-0.46410
C	0.88760	3.05050	-2.53220
C	-0.32280	3.32220	-1.84330
O	-1.49550	3.21330	-2.25030
C	1.53330	4.12550	-0.49670
H	1.59100	5.20670	-0.66190
H	2.08340	3.91420	0.42670
C	2.09150	3.37640	-1.72720
H	2.62740	2.46340	-1.39510
H	2.83890	3.96510	-2.27440
H	-0.62430	4.29380	0.11280

C	0.91370	2.57840	-3.93910
H	1.33770	3.32670	-4.62350
H	1.53030	1.67460	-4.06100
H	-0.10890	2.36290	-4.25860

TS2'

```
37
REACTION1_A_OBS_MINOR_TS_O_BOUND_SP_TFE_DEF2_TZVP
C         -1.25700        1.52430        0.06860
C         -2.51100        1.96410        0.48550
C         -3.62510        1.17150        0.21400
C         -3.49890       -0.04260       -0.47470
C         -2.25440       -0.50350       -0.89940
C         -1.14990        0.28940       -0.60730
C          0.08280        2.12240        0.09490
C          0.93780        1.09400       -0.39710
N          0.20510        0.07090       -0.88140
H         -2.61880        2.90340        1.01540
H         -4.60600        1.49680        0.54040
H         -4.38240       -0.63700       -0.67560
H         -2.15500       -1.44450       -1.42690
H          2.00870        1.11180       -0.49280
C          0.73060       -1.05260       -1.63880
H          1.77790       -0.86140       -1.86380
H          0.63270       -1.98220       -1.07170
H          0.18600       -1.14810       -2.58050
C          0.54510        3.12740        1.11570
H          1.47560        3.59710        0.78910
H         -0.19960        3.91330        1.25640
H          0.71240        2.65210        2.08630
C         -0.01170        3.18250       -1.67740
C          1.64040        4.78110       -1.78930
C          1.41190        3.41230       -1.95810
C          2.96990        5.41200       -1.99530
H          3.72480        4.63280       -2.12210
H          2.98680        6.05460       -2.88670
H          3.24600        6.05280       -1.14730
H         -0.51670        2.38720       -2.21700
O          2.22180        2.47770       -2.23140
C         -0.72770        4.48950       -1.48200
H         -1.40910        4.66150       -2.32220
H         -1.35370        4.50980       -0.58440
C          0.40140        5.55030       -1.45980
H          0.48510        6.06160       -0.48970
H          0.19530        6.34370       -2.19250
```

TS3'

```
37
REACTION1_A_OBS_INT_MINOR_TS_SP_TFE_DEF2_TZVP
C         -0.69890       1.40940        0.44080
C         -1.64080       1.48220        1.46120
C         -2.91080       0.95880        1.22300
C         -3.24480       0.38840       -0.01250
C         -2.31450       0.31550       -1.04820
C         -1.04800       0.82560       -0.78820
C          0.65360       1.99220        0.30620
C          1.06270       1.54890       -0.98940
N          0.06590       0.91690       -1.63940
H         -1.39350       1.98350        2.38940
H         -3.66220       1.01010        2.00210
H         -4.24530       0.00320       -0.17050
H         -2.57620      -0.11130       -2.00890
H          2.01490       1.71450       -1.47220
C          0.06120       0.52700       -3.03780
H          1.07330       0.59570       -3.43410
H         -0.28910      -0.50230       -3.13460
H         -0.59670       1.18290       -3.61610
C          1.65170       1.98240        1.45840
H          2.62520       2.34390        1.11560
H          1.30380       2.63470        2.25930
H          1.77620       0.96310        1.83280
C          0.35370       3.88050        0.07120
C         -1.74330       4.65410        0.71000
C         -0.49840       4.27640        1.23960
C         -2.82530       5.23340        1.54780
H         -2.50010       5.26000        2.58990
H         -3.09400       6.25160        1.23300
H         -3.74590       4.63640        1.48050
H          1.40260       4.16060        0.12200
O         -0.14660       4.21050        2.44540
C         -0.39340       4.22630       -1.20200
H          0.00350       5.16740       -1.59570
H         -0.28450       3.48510       -1.99900
C         -1.85850       4.43160       -0.75730
H         -2.46870       3.54100       -0.98370
H         -2.33370       5.26910       -1.28570
```

INT2'

```
37
REACTION1_A_OBS_MINOR_O_BOUND_SP_TFE_DEF2_TZVP
C         -1.39370       1.54720       -0.10630
C         -2.65770       1.71260        0.42540
```

C	-3.63700	0.73820	0.18390
C	-3.32790	-0.37940	-0.58590
C	-2.05260	-0.55720	-1.13460
C	-1.09240	0.42240	-0.89500
C	-0.17500	2.43690	-0.04500
C	0.94170	1.48610	-0.60130
N	0.22110	0.48400	-1.35860
H	-2.88840	2.58290	1.03070
H	-4.62960	0.85110	0.60340
H	-4.08580	-1.13480	-0.76210
H	-1.82540	-1.43600	-1.72560
H	1.57230	1.03420	0.17580
C	0.93460	-0.68120	-1.83610
H	1.89630	-0.35830	-2.23600
H	1.10370	-1.42730	-1.04390
H	0.37870	-1.15570	-2.64720
C	0.16150	2.98070	1.34050
H	1.08830	3.56100	1.31830
H	-0.63590	3.63060	1.70820
H	0.28160	2.16100	2.05260
C	-0.16440	3.52100	-1.17340
C	1.76340	4.64870	-2.02040
C	1.26210	3.48190	-1.61960
C	3.14060	4.94650	-2.51430
H	3.77930	4.06310	-2.44770
H	3.13110	5.27900	-3.55960
H	3.60350	5.75140	-1.93110
H	-0.81500	3.15980	-1.97960
O	1.83800	2.25760	-1.42680
C	-0.38310	5.02740	-0.94640
H	-1.40810	5.34750	-1.14400
H	-0.13960	5.29600	0.08400
C	0.65960	5.69100	-1.89940
H	1.03420	6.64080	-1.50170
H	0.21150	5.91740	-2.87730

INT3'

37
REACTION1_A_OBS_INT_MINOR_SP_TFE_DEF2_TZVP

C	-0.80170	1.45460	0.38730
C	-1.75130	1.49540	1.39880
C	-2.95410	0.81390	1.19860
C	-3.20820	0.11510	0.01360
C	-2.26030	0.06570	-1.00940
C	-1.06870	0.74060	-0.78430
C	0.53150	2.14210	0.25160

C	0.97190	1.63530	-1.05460
N	0.04970	0.87330	-1.64170
H	-1.55810	2.06790	2.29830
H	-3.71230	0.83690	1.97250
H	-4.15550	-0.39470	-0.11590
H	-2.45490	-0.47010	-1.93050
H	1.90230	1.86100	-1.55550
C	0.09520	0.35440	-2.99850
H	1.08300	0.53290	-3.41980
H	-0.10440	-0.71880	-2.99100
H	-0.65730	0.85350	-3.61550
C	1.53810	1.87950	1.39750
H	2.52630	2.24960	1.11180
H	1.21250	2.42240	2.28320
H	1.60970	0.80980	1.60630
C	0.35830	3.78430	0.14070
C	-1.70530	4.74800	0.78570
C	-0.51140	4.25900	1.31670
C	-2.76560	5.37340	1.62210
H	-2.44750	5.37490	2.66720
H	-2.98370	6.40790	1.32010
H	-3.71670	4.82490	1.54890
H	1.38520	4.14620	0.22280
O	-0.17630	4.11260	2.52080
C	-0.35810	4.29170	-1.11890
H	0.09060	5.24400	-1.41230
H	-0.27430	3.61980	-1.98070
C	-1.82220	4.53250	-0.68520
H	-2.44520	3.65030	-0.92100
H	-2.27710	5.37750	-1.21920

Reaction 8a + 7 ($R^2 = CH_3$): unobserved regioisomer; major stereoisomer

10

37
REACTION1_A_NOTOBS_1_DEF2_TZVP_SP_TRIFLUOROETHANOL

C	-1.49490	1.94770	-0.03020
C	-2.70550	2.04870	0.63080
C	-3.64310	1.01330	0.51790
C	-3.33850	-0.11220	-0.24310
C	-2.11700	-0.23080	-0.91280
C	-1.20190	0.81810	-0.81330
C	-0.31030	2.88770	-0.02840
C	0.74290	2.11690	-0.93290
N	0.03600	0.93930	-1.43130
H	-2.92290	2.91790	1.24220
H	-4.59460	1.08270	1.03100

H	-4.05860	-0.91970	-0.31730
H	-1.89520	-1.11750	-1.49380
H	1.61810	1.81810	-0.34190
C	0.78690	-0.19860	-1.90920
H	1.62900	0.15210	-2.50910
H	1.17740	-0.82470	-1.09120
H	0.16350	-0.82250	-2.55340
C	0.22540	3.10890	1.39340
H	1.10510	3.75950	1.39450
H	-0.53450	3.56370	2.03220
H	0.50320	2.14990	1.83710
C	-0.49840	4.24680	-0.80450
C	1.16510	3.14630	-2.01810
C	-1.12730	3.96350	-2.19600
H	-2.00200	3.31840	-2.11040
C	0.96890	4.44460	-1.22530
O	1.72490	5.34710	-0.98900
H	-1.45090	4.90350	-2.64850
C	0.01910	3.30270	-3.03700
H	-0.27790	2.34130	-3.45400
H	0.33340	3.95340	-3.85620
C	-1.13210	5.39100	-0.03890
H	-2.15250	5.14270	0.26580
H	-0.55460	5.64940	0.85000
H	-1.17540	6.28350	-0.66790
H	2.15950	3.00650	-2.43670

TS1

37
REACTION1_A_NOTOBS_1_TS_DEF2_TZVP_SP_TRIFLUOROETHANOL(DIRECT)

C	-1.42900	1.76970	0.21140
C	-2.61140	1.98740	0.91590
C	-3.66900	1.09680	0.74590
C	-3.55950	-0.00560	-0.11290
C	-2.37790	-0.26130	-0.80300
C	-1.32920	0.62940	-0.60420
C	-0.15160	2.51870	0.12900
C	0.66060	1.65270	-0.67880
N	-0.01950	0.59300	-1.11920
H	-2.70660	2.83340	1.58470
H	-4.59430	1.25730	1.28650
H	-4.40220	-0.67550	-0.23410
H	-2.28640	-1.11910	-1.45780
H	1.68130	1.83160	-0.98270
C	0.43650	-0.32910	-2.14730
H	1.52340	-0.29970	-2.19810

H	0.11750	-1.34160	-1.89940
H	0.02350	-0.03940	-3.11790
C	0.43240	3.10250	1.41430
H	1.27250	3.76390	1.20810
H	-0.32380	3.69450	1.92950
H	0.72990	2.28920	2.08120
C	-0.38690	4.03280	-1.06680
C	1.49600	4.37490	-2.41970
C	-0.72600	3.51460	-2.45140
H	-1.30350	2.58440	-2.45270
C	0.98660	4.71060	-1.16510
O	1.42800	5.43320	-0.24250
H	-1.35100	4.26580	-2.94370
C	0.63050	3.43350	-3.18370
H	1.01110	2.38720	-3.17050
H	0.52580	3.67850	-4.24730
C	-1.42590	4.87050	-0.36500
H	-2.37880	4.35890	-0.22310
H	-1.03770	5.25470	0.57770
H	-1.60370	5.74300	-1.00270
H	2.42240	4.78440	-2.80160

TS2

37
REACTION1_A_NOTOBS1_O_BOUND_TS_REDONE_DEF2_TZVP_SP_TRIFLUOROETHANOL(NOTOBS_MAJOR)

C	-1.21070	2.22760	0.03410
C	-2.58970	2.06660	0.11840
C	-3.18380	0.99120	-0.54200
C	-2.40780	0.09290	-1.28320
C	-1.02080	0.22080	-1.35430
C	-0.44480	1.28770	-0.67620
C	-0.28630	3.25010	0.56350
C	1.01280	2.68240	0.31560
N	0.91160	1.61220	-0.48100
H	-3.19300	2.76370	0.68850
H	-4.25660	0.85060	-0.48580
H	-2.88900	-0.73020	-1.79830
H	-0.42560	-0.49250	-1.91040
H	1.97530	3.06640	0.60350
C	2.05200	1.02720	-1.18030
H	2.59710	1.85270	-1.64420
H	2.68830	0.48120	-0.48020
H	1.69440	0.34380	-1.94700
C	-0.61820	3.93240	1.87230
H	0.19100	4.58010	2.20930

H	-1.52220	4.53900	1.77850
H	-0.80130	3.18350	2.64810
C	-0.21750	4.69350	-0.87500
C	0.01090	3.60530	-2.91590
C	-1.57120	4.76610	-1.54630
H	-2.40690	4.51480	-0.88900
C	0.74930	4.11130	-1.86260
O	1.99080	4.06410	-1.60240
H	-1.73060	5.80080	-1.87030
C	-1.46080	3.83940	-2.78190
H	-2.00870	2.89910	-2.63330
H	-1.90480	4.31640	-3.66490
C	0.28420	5.90340	-0.13350
H	-0.40540	6.27010	0.62830
H	1.26600	5.70870	0.29970
H	0.41780	6.70160	-0.87260
H	0.46260	3.12860	-3.77800

TS4

37
REACTION1_A_MAJOR_SECOND_TS_BS_HESSREF_REDONE_SP_TFE_DEF2_TZVP

C	-1.59140	2.07310	-0.06780
C	-2.85490	2.25320	0.45740
C	-3.82060	1.25160	0.27250
C	-3.49140	0.08480	-0.41100
C	-2.21390	-0.10870	-0.95590
C	-1.27370	0.89940	-0.77170
C	-0.37360	2.96280	-0.05860
C	0.69460	2.12740	-0.77740
N	0.06990	0.91920	-1.15500
H	-3.09680	3.14800	1.02150
H	-4.80230	1.35470	0.71790
H	-4.23820	-0.69250	-0.52980
H	-1.96940	-1.02110	-1.48560
H	1.68470	2.00190	-0.33530
C	0.74610	-0.18060	-1.80140
H	1.82480	-0.01870	-1.76110
H	0.51060	-1.11820	-1.28900
H	0.46130	-0.28070	-2.85580
C	0.04980	3.26610	1.42100
H	0.93860	3.90130	1.43810
H	-0.76550	3.78250	1.93160
H	0.25030	2.32750	1.94080
C	-0.47090	4.28880	-0.85940
C	1.08340	3.32970	-2.29870
C	-1.22070	4.13950	-2.11750

H	-2.19710	3.66700	-1.98940
C	0.98850	4.45560	-1.34220
O	1.86160	5.16070	-0.89080
H	-1.45500	5.12030	-2.54890
C	-0.24990	3.33540	-3.06180
H	-0.58890	2.30250	-3.20200
H	-0.16460	3.79980	-4.04710
H	-0.83990	5.07910	-0.20450
C	2.36700	2.98990	-2.98430
H	2.36360	1.99570	-3.44300
H	2.59390	3.70300	-3.78720
H	3.19790	3.05380	-2.27450

INT2

37
REACTION1_A_NOTOBS_1_O_BOUND_REDONE_DEF2_TZVP_SP_TRIFLUOROETHANO
L

C	-1.16270	2.23230	-0.15710
C	-2.53810	2.07930	-0.11810
C	-3.10000	0.81600	-0.34470
C	-2.27470	-0.27670	-0.59550
C	-0.88290	-0.14620	-0.60170
C	-0.34060	1.11300	-0.35800
C	-0.29330	3.45390	0.10990
C	1.12820	2.88460	-0.25420
N	1.00200	1.46120	-0.20540
H	-3.18050	2.92850	0.08550
H	-4.17620	0.69250	-0.33030
H	-2.71450	-1.24980	-0.78370
H	-0.24950	-1.00550	-0.78370
H	1.94790	3.23210	0.37740
C	2.08520	0.61970	-0.67380
H	3.03430	1.04870	-0.34730
H	1.99690	-0.37780	-0.23700
H	2.10190	0.53250	-1.76710
C	-0.39880	3.83700	1.59350
H	0.33520	4.59250	1.87900
H	-1.39640	4.22630	1.81210
H	-0.24060	2.95410	2.21720
C	-0.42760	4.62970	-0.91900
C	-0.01540	4.44360	-3.26600
C	-1.74680	4.94370	-1.66070
H	-2.38480	4.06200	-1.68670
C	0.41240	4.10140	-2.05360
O	1.44560	3.34150	-1.61600
H	-2.30750	5.76370	-1.20430

```
C          -1.29150        5.25400       -3.12100
H          -2.05680        4.96330       -3.84710
H          -1.11650        6.32880       -3.26640
C           0.20190        5.93120       -0.37520
H          -0.38570        6.33950        0.45090
H           1.22520        5.77220       -0.02550
H           0.23750        6.68090       -1.16860
H           0.45160        4.19160       -4.20770
```

Reaction 8a + 7 (R^2 = CH_3): unobserved regioisomer; minor stereoisomer

TS2

```
37
REACTION1_A_NOTOBS2_O_BOUND_TS_REDONE_SP_TFE_DEF2_TZVP
C          -1.70100        1.80350       -0.19550
C          -2.20530        1.97840        1.09040
C          -3.39460        1.33910        1.43870
C          -4.08180        0.53800        0.51730
C          -3.59190        0.34420       -0.77330
C          -2.39720        0.97430       -1.09810
C          -0.56730        2.39560       -0.91840
C          -0.58130        1.72920       -2.18560
N          -1.67260        0.95000       -2.29870
H          -1.68610        2.60420        1.80690
H          -3.79670        1.46310        2.43740
H          -5.00750        0.05810        0.81210
H          -4.12430       -0.27100       -1.48850
H           0.13860        1.80030       -2.98060
C          -2.11970        0.30300       -3.51990
H          -1.33980        0.39360       -4.27330
H          -2.32840       -0.75240       -3.33200
H          -3.02490        0.78960       -3.89270
C           0.74370        2.72100       -0.24300
H           1.37210        3.32490       -0.90060
H           0.58410        3.27400        0.68400
H           1.28180        1.80340        0.01180
C          -1.32500        4.24690       -1.52660
C           0.48860        5.49540       -2.24020
C          -0.37140        4.48870       -2.65100
O          -0.44080        3.80570       -3.71430
C          -1.06080        5.24200       -0.42160
H          -1.92000        5.91590       -0.33840
H          -0.95550        4.77660        0.56220
C           0.20320        6.02310       -0.86860
H           1.04960        5.88490       -0.18130
H          -0.00790        7.10110       -0.86340
C          -2.75270        3.98600       -1.91280
```

```
H          -3.37610        3.66680       -1.07650
H          -3.15640        4.93140       -2.29440
H          -2.80820        3.26900       -2.73090
H           1.25530        5.91020       -2.88400
```

Reaction 8a + 11 (R^2 = H): observed regioisomer; major stereoisomer

15a

```
34
BROMO_H_OXALLYL_MAJOR_PROD
C          -1.52840        1.96470       -0.07940
C          -2.74420        2.07430        0.57060
C          -3.68100        1.03910        0.45750
C          -3.37220       -0.09140       -0.29530
C          -2.14800       -0.21410       -0.95860
C          -1.23290        0.83420       -0.85790
C          -0.34250        2.90200       -0.07040
C           0.70430        2.13100       -0.98570
N           0.01020        0.94720       -1.47110
H          -2.96610        2.95010        1.17160
H          -4.63630        1.11250        0.96270
H          -4.09300       -0.89820       -0.36990
H          -1.92370       -1.10170       -1.53690
H           1.60490        1.86270       -0.42330
C           0.76860       -0.20950       -1.90610
H           1.65330        0.12780       -2.44490
H           1.08350       -0.84400       -1.06360
H           0.17240       -0.81520       -2.59200
C           0.19310        3.14440        1.34640
H           1.08590        3.77630        1.33310
H          -0.56490        3.64280        1.95660
H           0.44490        2.19610        1.82630
C          -0.52480        4.25800       -0.82650
C           1.06840        3.17660       -2.07340
C          -1.19680        4.03930       -2.20240
H          -2.09260        3.42530       -2.11320
C           0.92710        4.48400       -1.26040
O           1.70440        5.36390       -1.04070
H          -1.48280        4.99810       -2.63790
C          -0.08680        3.34370       -3.07000
H          -0.40160        2.37900       -3.46280
H           0.22920        3.97840       -3.89800
H          -0.94950        5.05280       -0.21680
Br          2.84490        2.87300       -2.92340
```

TS2

```
34
BROMO_H_OXALLYL_MAJOR_O_BOUND_TS_BS
C         -0.85970        3.43470         0.22820
C         -2.13860        2.89090         0.14510
C         -2.32810        1.73880        -0.61720
C         -1.25810        1.14790        -1.30120
C          0.03500        1.66150        -1.20820
C          0.21090        2.79310        -0.42250
C         -0.32220        4.65570         0.84240
C          1.09390        4.51000         0.72150
N          1.38640        3.49010        -0.09630
H         -2.97150        3.35370         0.66220
H         -3.31540        1.29850        -0.69020
H         -1.43320        0.26470        -1.90400
H          0.86150        1.19660        -1.72980
H          1.86320        5.17760         1.06870
C          2.70040        3.25220        -0.68360
H          3.20250        4.21530        -0.76450
H          3.27270        2.54280        -0.08160
H          2.56840        2.86540        -1.69310
C         -0.99410        5.33940         2.00560
H         -0.43260        6.22040         2.32360
H         -2.00260        5.66290         1.73450
H         -1.08010        4.66100         2.85930
C         -0.50150        6.05940        -0.68220
C          0.24690        5.13420        -2.60480
C         -1.73590        5.77270        -1.49310
H         -2.53060        5.27260        -0.93430
C          0.70990        5.87580        -1.51500
O          1.86460        6.20200        -1.14840
H         -2.14780        6.72420        -1.84800
C         -1.22510        4.93140        -2.68830
H         -1.48700        3.87120        -2.58800
H         -1.64750        5.27680        -3.63850
H         -0.50160        6.89100         0.01490
Br         1.40630        4.43690        -3.97740
```

INT2

```
34
BROMO_H_OXALLYL_MAJOR_O_BOUND
C         -0.68120        3.09340         0.18860
C         -1.94560        2.53800         0.09420
C         -2.07750        1.16640        -0.15800
C         -0.94120        0.37500        -0.30430
C          0.34170        0.91700        -0.18320
```

C	0.45540	2.27870	0.08340
C	-0.25220	4.51910	0.48770
C	1.30670	4.41020	0.29390
N	1.61100	3.01770	0.34550
H	-2.82860	3.15610	0.21260
H	-3.06260	0.72430	-0.24510
H	-1.04850	-0.68370	-0.51190
H	1.21750	0.28940	-0.29020
H	1.90910	4.98190	1.00200
C	2.93760	2.55200	-0.01180
H	3.67800	3.23950	0.40080
H	3.11350	1.56490	0.42080
H	3.07900	2.50100	-1.09790
C	-0.63690	4.92960	1.91350
H	-0.24410	5.92160	2.15590
H	-1.72440	4.95730	2.01630
H	-0.24890	4.21220	2.64040
C	-0.62520	5.57880	-0.58540
C	0.08520	5.67710	-2.82630
C	-1.88240	5.53960	-1.47620
H	-2.21220	4.50790	-1.59980
C	0.47850	5.41090	-1.58500
O	1.62600	4.97530	-1.03650
H	-2.71360	6.12680	-1.08080
C	-1.37650	6.06160	-2.85790
H	-1.90570	5.59610	-3.69300
H	-1.50450	7.14710	-2.95620
H	-0.53120	6.56870	-0.11480
Br	1.18590	5.61590	-4.41110

TS4

34
BROMO_H_OXALLYL_MAJOR_SECOND_TS_BS_HESSREF_SP

C	-1.57410	2.01720	-0.01730
C	-2.85430	2.18120	0.48280
C	-3.83740	1.20300	0.26500
C	-3.52670	0.04760	-0.44660
C	-2.23330	-0.14340	-0.95770
C	-1.29290	0.84790	-0.73300
C	-0.38020	2.89000	0.03940
C	0.68950	2.05700	-0.74820
N	0.04530	0.87960	-1.13610
H	-3.08290	3.08910	1.03300
H	-4.83040	1.34310	0.67530
H	-4.27820	-0.70940	-0.64090
H	-1.98750	-1.03640	-1.51760

H	1.69890	1.92030	-0.36670
C	0.69940	-0.22460	-1.81420
H	1.75110	0.02280	-1.95480
H	0.63560	-1.15230	-1.23570
H	0.25300	-0.38790	-2.80010
C	0.09450	3.21410	1.44250
H	0.98210	3.85220	1.41640
H	-0.70440	3.72950	1.98660
H	0.36370	2.32610	2.02360
C	-0.48050	4.19220	-0.81000
C	1.05630	3.28990	-2.25030
C	-1.23870	3.98560	-2.23220
H	-2.18170	3.45100	-2.13740
C	0.99530	4.42350	-1.32670
O	1.84080	5.18540	-0.93630
H	-1.40940	5.00150	-2.59290
C	-0.21880	3.23530	-3.07870
H	-0.55680	2.21620	-3.25210
H	-0.03240	3.76680	-4.01420
H	-0.90900	5.01250	-0.23060
Br	2.72430	3.04260	-3.19540

Reaction 8a + 11 (R^2 = H): observed regioisomer; minor stereoisomer

16a

34

BROMO_H_OXALLYL_MINOR_PROD

C	-1.38600	1.67930	-0.16390
C	-2.73490	1.63890	0.13300
C	-3.44130	0.44170	-0.04460
C	-2.77580	-0.69050	-0.50860
C	-1.41250	-0.66210	-0.81550
C	-0.72580	0.54050	-0.64700
C	-0.39600	2.82020	-0.00610
C	0.94100	2.18190	-0.60150
N	0.60850	0.80080	-0.89570
H	-3.24180	2.52260	0.50710
H	-4.49990	0.39720	0.18040
H	-3.32480	-1.61610	-0.64250
H	-0.90940	-1.54560	-1.18830
H	1.78540	2.24670	0.09350
C	1.59620	-0.24830	-1.03460
H	2.59300	0.18570	-0.97450
H	1.48500	-1.00020	-0.24190
H	1.51660	-0.74700	-2.00580
C	-0.25880	3.23300	1.46130
H	0.54990	3.95130	1.61490

H	-1.18940	3.67750	1.82470
H	-0.04760	2.35170	2.07160
C	-0.71420	3.98760	-0.99030
C	1.21520	3.08130	-1.83700
C	-0.22310	3.38040	-2.31150
Br	2.43510	2.30150	-3.19900
H	-1.75840	4.29140	-0.99040
O	-0.75480	3.22460	-3.36770
C	0.31550	5.13000	-0.85300
H	0.03520	5.95870	-1.50700
H	0.38250	5.52220	0.16110
C	1.65530	4.47440	-1.34100
H	2.39710	4.38280	-0.54450
H	2.10520	5.04300	-2.15360

TS2

34
BROMO_H_OXALLYL_MINOR_O_BOUND_TS_LH

C	-1.23320	1.48920	0.03890
C	-2.48310	1.94020	0.45970
C	-3.60460	1.16010	0.18570
C	-3.49140	-0.05360	-0.50700
C	-2.25250	-0.52650	-0.93360
C	-1.13980	0.25440	-0.64020
C	0.11150	2.06730	0.07220
C	0.95270	1.04260	-0.43790
N	0.21230	0.02460	-0.91950
H	-2.58090	2.87660	0.99660
H	-4.58190	1.49380	0.51380
H	-4.38120	-0.63780	-0.70920
H	-2.16330	-1.46700	-1.46360
H	2.02240	1.06100	-0.55270
C	0.72530	-1.09780	-1.68890
H	1.77920	-0.92540	-1.89750
H	0.60200	-2.03230	-1.13580
H	0.19030	-1.16890	-2.63830
C	0.58570	3.09300	1.06390
H	1.53320	3.52860	0.73990
H	-0.14060	3.90140	1.16940
H	0.72600	2.64480	2.05170
C	0.02900	3.13230	-1.75780
C	1.60950	4.74590	-1.79540
C	1.46070	3.37110	-2.01230
Br	3.29640	5.64880	-1.97180
H	-0.45170	2.31490	-2.28560
O	2.28050	2.46370	-2.28500

C	-0.72460	4.41580	-1.56430
H	-1.38280	4.58270	-2.42380
H	-1.37730	4.40310	-0.68610
C	0.36950	5.51050	-1.48860
H	0.42250	5.99870	-0.50770
H	0.17860	6.30610	-2.21880

INT2

34
BROMO_H_OXALLYL_MINOR_O_BOUND

C	-1.38660	1.54750	-0.09100
C	-2.65190	1.71480	0.43780
C	-3.63220	0.74390	0.18870
C	-3.32340	-0.37100	-0.58550
C	-2.04700	-0.55020	-1.13070
C	-1.08540	0.42540	-0.88260
C	-0.16490	2.43300	-0.02210
C	0.94900	1.47900	-0.57700
N	0.23230	0.48290	-1.33880
H	-2.88220	2.58270	1.04660
H	-4.62570	0.85690	0.60560
H	-4.08270	-1.12350	-0.76730
H	-1.82000	-1.42720	-1.72410
H	1.58520	1.03020	0.19620
C	0.94480	-0.68580	-1.81300
H	1.91020	-0.36740	-2.20730
H	1.10510	-1.43200	-1.01980
H	0.39110	-1.15630	-2.62740
C	0.16890	2.97680	1.36380
H	1.09850	3.55260	1.34670
H	-0.62770	3.62990	1.72750
H	0.28170	2.15690	2.07650
C	-0.15170	3.51740	-1.15030
C	1.72720	4.64450	-2.00900
C	1.27680	3.46320	-1.59970
Br	3.49140	5.02810	-2.69200
H	-0.79700	3.15740	-1.96060
O	1.85110	2.25610	-1.40370
C	-0.37410	5.02500	-0.92360
H	-1.40210	5.33880	-1.11240
H	-0.11830	5.29680	0.10240
C	0.65000	5.70040	-1.89080
H	1.04070	6.64040	-1.49340
H	0.19890	5.92260	-2.86580

Reaction 8a + 11 (R^2 = H): unobserved regioisomer; major stereoisomer

TS2

```
34
BROMO_H_OXALLYL_NOTOBS_O_BOUND_TS_LH
C          -0.85480        3.50710         0.19080
C          -2.13530        2.96600         0.11260
C          -2.32390        1.79050        -0.61160
C          -1.24620        1.16720        -1.25350
C           0.04780        1.67810        -1.16190
C           0.22080        2.84340        -0.42450
C          -0.32180        4.71820         0.83330
C           1.10040        4.56600         0.72120
N           1.39860        3.53350        -0.07660
H          -2.96890        3.44880         0.60930
H          -3.31280        1.35360        -0.68250
H          -1.41530        0.25830        -1.81890
H           0.87710        1.17600        -1.64370
H           1.86850        5.22250         1.09170
C           2.73100        3.31180        -0.63260
H           3.04260        4.24940        -1.09940
H           3.42490        3.02470         0.16000
H           2.68730        2.51740        -1.37380
C          -0.99430        5.27470         2.06290
H          -0.41240        6.07730         2.51150
H          -1.98180        5.67430         1.82180
H          -1.12030        4.47820         2.80230
C          -0.47550        6.16910        -0.68380
C           0.24220        5.21840        -2.63560
C          -1.73430        5.90460        -1.46600
H          -2.51910        5.42440        -0.87850
C           0.72830        5.91290        -1.53560
O           1.90080        6.18780        -1.17420
H          -2.13510        6.85640        -1.82230
C          -1.24230        5.05060        -2.66110
H          -1.53160        3.99750        -2.55170
H          -1.69790        5.40240        -3.59520
Br         -0.43110        7.88460         0.30050
H           0.89060        4.87550        -3.43330
```

INT2

```
34
BROMO_H_OXALLYL_NOTOBS_O_BOUND
C          -0.69930        3.13070         0.15120
C          -1.95750        2.57030         0.01850
C          -2.07570        1.19390        -0.21170
```

C	-0.93340	0.40150	-0.29100
C	0.34170	0.94870	-0.12340
C	0.44290	2.31630	0.11720
C	-0.27310	4.56120	0.48970
C	1.27750	4.44760	0.26560
N	1.58500	3.06240	0.41540
H	-2.84720	3.18450	0.09620
H	-3.05580	0.74820	-0.33050
H	-1.03040	-0.66170	-0.48020
H	1.22240	0.32110	-0.17520
H	1.88770	5.08020	0.90910
C	2.92380	2.58290	0.12900
H	3.64760	3.29170	0.53470
H	3.08430	1.61890	0.61640
H	3.10520	2.47640	-0.94750
C	-0.65820	4.86740	1.94070
H	-0.23380	5.81060	2.27980
H	-1.74500	4.92460	2.03260
H	-0.30420	4.06040	2.58580
C	-0.65330	5.57490	-0.60740
C	-0.03870	5.62370	-2.87580
C	-1.94720	5.64760	-1.40720
H	-2.34440	4.63520	-1.50070
C	0.38490	5.35970	-1.63670
O	1.53940	4.89040	-1.12030
H	-2.70690	6.28460	-0.95750
C	-1.46930	6.11640	-2.81130
H	-2.09660	5.70820	-3.60860
H	-1.51370	7.21010	-2.88660
Br	-0.29880	7.55920	0.14900
H	0.54300	5.53640	-3.78220

Product

34
BROMO_H_OXALLYL_NOTOBS_PROD

C	-1.53360	1.97240	-0.08620
C	-2.74840	2.08940	0.56460
C	-3.68840	1.05810	0.44740
C	-3.38330	-0.07310	-0.30620
C	-2.16000	-0.20010	-0.97090
C	-1.24130	0.84480	-0.86910
C	-0.34770	2.90590	-0.07690
C	0.69970	2.14710	-1.00250
N	-0.00270	0.96620	-1.49530
H	-2.96500	2.97430	1.15200
H	-4.64530	1.13600	0.94870

H	-4.10770	-0.87650	-0.38270
H	-1.94230	-1.08740	-1.55230
H	1.58330	1.85910	-0.42050
C	0.76380	-0.18140	-1.92920
H	1.60360	0.15570	-2.53990
H	1.15770	-0.77150	-1.08690
H	0.14860	-0.83460	-2.55110
C	0.20950	3.12130	1.33560
H	1.08550	3.77590	1.32410
H	-0.54100	3.57520	1.98280
H	0.49510	2.15740	1.76250
C	-0.53010	4.24110	-0.86600
C	1.10340	3.18520	-2.09100
C	-1.19480	4.01110	-2.23750
H	-2.07960	3.38340	-2.14190
C	0.93650	4.48310	-1.28970
O	1.69100	5.38060	-1.06200
H	-1.49720	4.96710	-2.66360
C	-0.06060	3.34250	-3.09140
H	-0.36700	2.37800	-3.49300
H	0.23760	3.98940	-3.91850
Br	-1.39660	5.71150	0.16310
H	2.08970	3.04590	-2.52760

TS4

34
BROMO_H_OXALLYL_NOTOBS_SECOND_TS_BS_HESSREF_SP_DEF2_TZVP_TFE

C	-1.52790	2.04390	0.00270
C	-2.79210	2.22440	0.52640
C	-3.78020	1.25040	0.32720
C	-3.46030	0.10630	-0.40050
C	-2.18210	-0.10060	-0.93530
C	-1.22130	0.88050	-0.71830
C	-0.30470	2.94570	0.04410
C	0.74630	2.09180	-0.74860
N	0.11330	0.89810	-1.12630
H	-3.00870	3.15750	1.03590
H	-4.78270	1.37550	0.71630
H	-4.22160	-0.64930	-0.55900
H	-1.95850	-0.99770	-1.50030
H	1.72640	1.98460	-0.28700
C	0.78180	-0.19400	-1.79330
H	1.86050	-0.04790	-1.72060
H	0.52980	-1.14330	-1.31010
H	0.51520	-0.25990	-2.85490
C	0.18430	3.19100	1.48320

H	1.04660	3.86320	1.48790
H	-0.59550	3.64310	2.09600
H	0.46830	2.23790	1.93620
C	-0.43720	4.23720	-0.80880
C	1.11980	3.32830	-2.24610
C	-1.19270	4.02150	-2.15040
H	-2.13550	3.49650	-2.01720
C	0.98900	4.46700	-1.29090
O	1.82050	5.22350	-0.85630
H	-1.40060	5.00900	-2.56380
C	-0.16640	3.25450	-3.04540
H	-0.47210	2.21560	-3.20610
H	-0.07060	3.71530	-4.03210
Br	-1.23790	5.75350	0.18540
H	2.09170	3.14760	-2.69100

Reaction 8a + 11 (R^2 = H): unobserved regioisomer; minor stereoisomer

TS2

34

BROMO_H_OXALLYL_NOTOBS_MINOR_O_BOUND_TS_BS

C	-1.33870	1.42950	0.18000
C	-2.58020	1.89060	0.61250
C	-3.69510	1.07480	0.43510
C	-3.58020	-0.18620	-0.16630
C	-2.34740	-0.66980	-0.59750
C	-1.24490	0.15240	-0.40530
C	-0.00380	2.02830	0.13920
C	0.84140	0.98190	-0.33930
N	0.10720	-0.07670	-0.71090
H	-2.67820	2.86300	1.08100
H	-4.66810	1.41730	0.76720
H	-4.46540	-0.79740	-0.29660
H	-2.25950	-1.64060	-1.06910
H	1.90440	1.02590	-0.50810
C	0.59450	-1.20310	-1.48960
H	1.68220	-1.17630	-1.50600
H	0.25790	-2.14050	-1.04310
H	0.22240	-1.12540	-2.51390
C	0.47310	3.03820	1.14850
H	1.42620	3.47340	0.84610
H	-0.25090	3.84600	1.27040
H	0.59970	2.56070	2.12450
C	-0.00510	3.16210	-1.67610
C	1.58030	4.79130	-1.50480
C	1.46080	3.43240	-1.79020
H	2.53770	5.29780	-1.53890

Br	-0.74980	1.98020	-3.08230
O	2.30810	2.54410	-2.03620
C	-0.79470	4.41720	-1.42540
H	-1.43300	4.64000	-2.28180
H	-1.46100	4.32350	-0.56390
C	0.29520	5.50650	-1.24340
H	0.26530	5.96270	-0.24440
H	0.11530	6.32610	-1.95220

INT2

34
BROMO_H_OXALLYL_NOTOBS_MINOR_O_BOUND

C	-1.38960	1.45260	-0.10620
C	-2.65760	1.63400	0.40860
C	-3.61560	0.62790	0.23250
C	-3.27910	-0.53650	-0.45340
C	-1.99840	-0.72990	-0.98050
C	-1.05940	0.28340	-0.80910
C	-0.18330	2.35900	-0.07120
C	0.94420	1.41490	-0.62740
N	0.25170	0.33670	-1.27630
H	-2.91070	2.54650	0.93730
H	-4.61470	0.75370	0.63170
H	-4.02260	-1.31530	-0.58150
H	-1.75300	-1.64130	-1.51110
H	1.62760	1.05280	0.15250
C	0.98830	-0.83670	-1.69330
H	1.93150	-0.51720	-2.13730
H	1.19500	-1.52580	-0.85970
H	0.42760	-1.37470	-2.45980
C	0.15290	2.86010	1.34010
H	1.09250	3.41900	1.35230
H	-0.64000	3.50570	1.72280
H	0.24870	2.00800	2.01560
C	-0.11980	3.52280	-1.10990
C	1.79040	4.60700	-1.96480
C	1.27030	3.42800	-1.61630
H	2.78460	4.77820	-2.35190
Br	-1.39510	3.13980	-2.75310
O	1.78060	2.17590	-1.53590
C	-0.27630	5.01090	-0.79900
H	-1.29790	5.37130	-0.90630
H	0.04990	5.19570	0.22760
C	0.73850	5.68000	-1.77040
H	1.14740	6.60770	-1.36080
H	0.24680	5.92720	-2.71930

Product

```
34
BROMO_H_OXALLYL_NOTOBS_MINOR_PROD
C         -1.55080        1.54150       -0.37810
C         -2.86880        1.45080        0.03660
C         -3.54740        0.23350       -0.08580
C         -2.88810       -0.87890       -0.60300
C         -1.55440       -0.80700       -1.00960
C         -0.89460        0.41740       -0.90000
C         -0.57540        2.70280       -0.25590
C          0.77260        2.05670       -0.81210
N          0.41250        0.71600       -1.26460
H         -3.37090        2.31590        0.44980
H         -4.58140        0.15700        0.22750
H         -3.41470       -1.82300       -0.68940
H         -1.05130       -1.68270       -1.40010
H          1.55030        2.02160       -0.03740
C          1.44120       -0.29600       -1.35790
H          2.34210        0.14270       -1.79130
H          1.69880       -0.73130       -0.37900
H          1.12050       -1.10140       -2.02120
C         -0.47530        3.17310        1.19810
H          0.37360        3.84050        1.36150
H         -1.38870        3.69590        1.49150
H         -0.35360        2.30710        1.85170
C         -0.79280        3.87190       -1.27800
C          1.19020        2.96190       -1.99120
C         -0.19660        3.26980       -2.57100
H          1.88920        2.50400       -2.68710
Br        -2.64880        4.57030       -1.41570
O         -0.65500        3.12720       -3.66270
C          0.22350        5.01120       -1.06130
H         -0.00860        5.82260       -1.75240
H          0.18660        5.41810       -0.05300
C          1.59320        4.34450       -1.42680
H          2.24430        4.23750       -0.55550
H          2.12850        4.93510       -2.17120
```

Reaction 8a + 11 ($R^2 = CH_3$): observed regioisomer; major stereoisomer

15b

```
37
REACTION2_B_MAJOR_DEF2_TZVP_SP_TRIFLUOROETHANOL
C         -1.53960        1.96940       -0.08320
C         -2.74840        2.07050        0.58180
```

C	-3.68640	1.03630	0.46710
C	-3.38490	-0.08690	-0.29880
C	-2.16550	-0.20450	-0.97180
C	-1.24970	0.84290	-0.87020
C	-0.35390	2.90740	-0.08100
C	0.69130	2.13450	-0.99400
N	-0.00930	0.95760	-1.48830
H	-2.96300	2.93640	1.19860
H	-4.63630	1.10450	0.98310
H	-4.10640	-0.89290	-0.37450
H	-1.94490	-1.08820	-1.55760
H	1.58460	1.85630	-0.42480
C	0.74400	-0.19810	-1.93430
H	1.63100	0.14050	-2.46860
H	1.05570	-0.84220	-1.09780
H	0.14580	-0.79470	-2.62660
C	0.19580	3.12120	1.33580
H	1.07610	3.77060	1.33240
H	-0.55910	3.57260	1.98270
H	0.47640	2.15980	1.77150
C	-0.54950	4.27220	-0.85050
C	1.07670	3.17610	-2.07580
C	-1.19190	3.99040	-2.23860
H	-2.06600	3.34650	-2.14380
C	0.91050	4.48590	-1.27800
O	1.67180	5.37950	-1.05280
H	-1.51330	4.93110	-2.68970
C	-0.05740	3.32940	-3.09460
H	-0.34500	2.36490	-3.50770
H	0.26240	3.98310	-3.90680
C	-1.18750	5.40740	-0.07510
H	-2.20670	5.14840	0.22430
H	-0.61350	5.65880	0.81760
H	-1.23500	6.30520	-0.69570
Br	2.87300	2.87510	-2.88730

TS1

37

REACTION2_B_MAJOR_TS_DEF2_TZVP_SP_TRIFLUOROETHANOL(DIRECT)

C	-1.50030	1.84480	0.34320
C	-2.79110	1.98060	0.84490
C	-3.76300	1.06050	0.44850
C	-3.46150	0.01990	-0.43700
C	-2.17150	-0.13770	-0.94760
C	-1.22040	0.77960	-0.53320
C	-0.27850	2.65250	0.47500

C	0.69870	1.89640	-0.25460
N	0.15170	0.87220	-0.88470
H	-3.03680	2.78600	1.52650
H	-4.77210	1.15180	0.83300
H	-4.23770	-0.67720	-0.72840
H	-1.93210	-0.94250	-1.63160
H	1.74250	2.15500	-0.36830
C	0.82070	0.03690	-1.87870
H	1.74290	0.52900	-2.18530
H	1.02620	-0.95200	-1.46480
H	0.17490	-0.06160	-2.75200
C	0.12620	3.30140	1.78280
H	1.00680	3.92310	1.61940
H	-0.68080	3.91680	2.18110
H	0.35170	2.53110	2.52660
C	-0.39690	4.12750	-0.92320
C	1.10550	3.70880	-2.61630
C	-1.20700	3.68140	-2.12840
H	-1.93440	2.89910	-1.90940
C	1.06770	4.23400	-1.33810
O	1.94740	4.59090	-0.51920
H	-1.78870	4.54460	-2.46590
C	-0.18350	3.26670	-3.21680
H	-0.21290	2.18600	-3.41960
H	-0.41150	3.75420	-4.17290
C	-0.94900	5.26160	-0.10880
H	-1.90280	5.02600	0.37150
H	-0.22030	5.60930	0.62070
H	-1.12350	6.08810	-0.80780
Br	2.78420	3.25120	-3.49640

TS2

37
REACTION2_B_MAJOR_O_BOUND_TS_RESTART_DEF2_TZVP_SP_TRIFLUOROETHANOL

C	-0.82040	3.53160	0.17870
C	-2.09370	2.97900	0.09840
C	-2.26980	1.80470	-0.63600
C	-1.19120	1.20460	-1.29490
C	0.09940	1.72420	-1.19620
C	0.25780	2.87250	-0.43340
C	-0.29330	4.77400	0.78380
C	1.13560	4.59940	0.70320
N	1.43410	3.56380	-0.08370
H	-2.93500	3.45070	0.59300
H	-3.25450	1.35860	-0.70840

H	-1.35580	0.30790	-1.88070
H	0.93520	1.25170	-1.69520
H	1.90750	5.24050	1.09100
C	2.75720	3.29360	-0.63440
H	3.30290	4.23440	-0.65920
H	3.27510	2.53520	-0.04320
H	2.64330	2.95520	-1.66360
C	-0.95420	5.32500	2.02800
H	-0.41030	6.17790	2.43400
H	-1.97770	5.64400	1.81640
H	-1.00150	4.55150	2.79940
C	-0.46660	6.16980	-0.67130
C	0.24000	5.13290	-2.58780
C	-1.71410	5.86340	-1.47650
H	-2.51350	5.40340	-0.89110
C	0.72970	5.88610	-1.53440
O	1.89310	6.20300	-1.17320
H	-2.10970	6.80950	-1.86110
C	-1.23960	4.97120	-2.64970
H	-1.53300	3.92280	-2.52690
H	-1.66120	5.31060	-3.60260
C	-0.40690	7.47710	0.07370
H	-1.25040	7.63330	0.74790
H	0.53650	7.57120	0.61260
H	-0.42590	8.27810	-0.67380
Br	1.35790	4.32970	-3.94600

TS3

37

REACTION2_B_MAJOR_INT_TS_REDONE_0_3_SP_TFE_DEF2_TZVP

C	-1.51670	0.80640	-0.16060
C	-2.79550	0.62550	0.36100
C	-3.62880	-0.31220	-0.24330
C	-3.21390	-1.04820	-1.36310
C	-1.94110	-0.88460	-1.89980
C	-1.11270	0.03650	-1.26480
C	-0.40440	1.72480	0.18530
C	0.64560	1.30280	-0.68790
N	0.21700	0.37970	-1.56150
H	-3.14350	1.24450	1.17450
H	-4.62910	-0.46000	0.14560
H	-3.89610	-1.75550	-1.82000
H	-1.61710	-1.44740	-2.76650
H	1.66220	1.66270	-0.72800
C	0.96550	-0.15800	-2.68530
H	1.99090	0.20590	-2.64320

H	0.96930	-1.24890	-2.63840
H	0.50950	0.15510	-3.62800
C	-0.08350	2.02080	1.63720
H	0.79210	2.66690	1.72350
H	-0.92140	2.52440	2.11940
H	0.12400	1.08860	2.16940
C	-0.87810	3.48600	-0.61290
C	-1.31020	4.95520	1.10490
C	0.33060	4.41030	-0.49770
H	1.28810	3.88510	-0.46740
C	-1.93940	3.95720	0.35340
O	-3.09250	3.49220	0.38620
H	0.36080	5.05240	-1.38330
C	0.09960	5.27510	0.76550
H	0.79610	5.03180	1.58030
H	0.24500	6.33980	0.54770
C	-1.40060	3.18750	-1.99590
H	-0.68210	2.65840	-2.62710
H	-2.33270	2.62780	-1.93230
H	-1.62370	4.14430	-2.47980
Br	-2.26340	6.00690	2.40810

INT2

37
REACTION2_B_MAJOR_O_BOUND_DEF2_TZVP_SP_TRIFLUOROETHANOL

C	-1.54590	1.98320	-0.10250
C	-2.74520	2.06840	0.58450
C	-3.67810	1.02930	0.47620
C	-3.39040	-0.08370	-0.30950
C	-2.16880	-0.20180	-0.97820
C	-1.24600	0.83230	-0.84580
C	-0.34730	2.92160	-0.12050
C	0.55450	2.22160	-1.20510
N	0.06670	0.88600	-1.31920
H	-2.96570	2.93080	1.20350
H	-4.62460	1.09560	0.99890
H	-4.12170	-0.87880	-0.40200
H	-1.94960	-1.07800	-1.57530
H	1.62800	2.24240	-1.01250
C	0.54750	0.03070	-2.38820
H	1.62290	0.17750	-2.50290
H	0.37420	-1.01650	-2.13130
H	0.06240	0.25350	-3.34580
C	0.31730	2.93140	1.26390
H	1.27750	3.45000	1.25890
H	-0.33480	3.42060	1.99170

H	0.48440	1.90610	1.60160
C	-0.59240	4.32750	-0.77630
C	-1.32730	4.66850	-2.99770
C	-1.96700	5.03970	-0.72750
H	-2.76160	4.31780	-0.55070
C	-0.50470	3.95770	-2.23460
O	0.36580	2.96100	-2.47240
H	-2.01710	5.80940	0.04650
C	-2.15370	5.61920	-2.16510
H	-3.20290	5.62210	-2.47010
H	-1.79030	6.65090	-2.24850
C	0.53620	5.32200	-0.42900
H	0.50480	5.59960	0.62740
H	1.52360	4.90840	-0.64890
H	0.41960	6.23160	-1.02220
Br	-1.48410	4.55410	-4.91960

INT3

37
REACTION2_B_MAJOR_INT_DEF2_TZVP_SP_TRIFLUOROETHANOL

C	-1.53650	0.81680	-0.26980
C	-2.79970	0.63550	0.27930
C	-3.59640	-0.39730	-0.21870
C	-3.15520	-1.23060	-1.25290
C	-1.88700	-1.07410	-1.80660
C	-1.10590	-0.05210	-1.27890
C	-0.43460	1.82110	0.00470
C	0.63690	1.30220	-0.86210
N	0.22270	0.29760	-1.61730
H	-3.17020	1.31920	1.02860
H	-4.58690	-0.54490	0.19430
H	-3.80620	-2.01180	-1.62720
H	-1.53400	-1.71830	-2.60220
H	1.63670	1.69490	-0.96770
C	0.98470	-0.36690	-2.66500
H	1.97670	0.07640	-2.72760
H	1.07260	-1.43080	-2.43610
H	0.47160	-0.24890	-3.62170
C	-0.01740	1.89900	1.48210
H	0.89460	2.48550	1.60360
H	-0.80920	2.39020	2.05070
H	0.15550	0.89830	1.88450
C	-0.81380	3.33730	-0.57830
C	-1.34800	4.84360	1.14110
C	0.36780	4.31990	-0.39870
H	1.34750	3.83400	-0.36090

```
C          -1.94610        3.88650        0.33490
O          -3.11190        3.45390        0.26010
H           0.39030        4.99140       -1.25970
C           0.08640        5.14410        0.88460
H           0.73810        4.85220        1.72120
H           0.26840        6.21210        0.71610
C          -1.31860        3.23330       -2.01690
H          -0.58280        2.79170       -2.69750
H          -2.24360        2.65750       -2.04860
H          -1.54850        4.23750       -2.38030
Br         -2.35850        5.91490        2.39970
```

Reaction 8a + 11 ($R^2 = CH_3$): observed regioisomer; minor stereoisomer

15b(minor stereoisomer)

```
37
REACTION2_B_MINOR_SP_TFE_DEF2_TZVP
C          -1.49490        1.55240       -0.12170
C          -2.73990        1.65820        0.47680
C          -3.70640        0.67420        0.23970
C          -3.40110       -0.41170       -0.57770
C          -2.14430       -0.54170       -1.17140
C          -1.19640        0.45670       -0.94200
C          -0.26490        2.42920        0.04230
C           0.80960        1.65090       -0.85030
N           0.10200        0.51670       -1.43260
H          -2.96330        2.49010        1.13480
H          -4.68470        0.75340        0.69790
H          -4.14910       -1.17650       -0.75520
H          -1.91650       -1.39540       -1.79690
H           1.67550        1.32180       -0.26430
C           0.81750       -0.70440       -1.75980
H           1.83210       -0.45830       -2.06400
H           0.85030       -1.40170       -0.90870
H           0.34040       -1.20670       -2.60420
C           0.13950        2.51170        1.51850
H           1.12470        2.96220        1.65520
H          -0.58820        3.09640        2.08770
H           0.16740        1.50710        1.94590
C          -0.39220        3.83400       -0.67130
C           1.23890        2.72420       -1.87770
C          -0.11050        3.42360       -2.12700
Br          2.17180        2.04420       -3.49910
O          -0.73260        3.61930       -3.12650
C           0.90490        4.64850       -0.42590
```

H	0.79900	5.62890	-0.89710
H	1.10250	4.81830	0.63200
C	2.02580	3.82480	-1.13940
H	2.74110	3.38290	-0.44200
H	2.58340	4.43760	-1.84590
C	-1.67510	4.60980	-0.45330
H	-1.82070	4.84540	0.60490
H	-1.63950	5.55050	-1.00760
H	-2.53880	4.04830	-0.80850

TS1

37
REACTION2_B_MINOR_TS_SP_TFE_DEF2_TZVP

C	-1.52340	1.54170	0.24050
C	-2.81910	1.70260	0.71930
C	-3.81900	0.86420	0.22520
C	-3.53490	-0.11640	-0.73120
C	-2.23700	-0.30420	-1.20560
C	-1.25860	0.52600	-0.68830
C	-0.25880	2.25750	0.49300
C	0.71000	1.46370	-0.20690
N	0.13280	0.53190	-0.94250
H	-3.05200	2.47070	1.44700
H	-4.83590	0.97790	0.58230
H	-4.33470	-0.74100	-1.11030
H	-2.01350	-1.05090	-1.95600
H	1.77350	1.63600	-0.26990
C	0.74490	-0.16640	-2.07170
H	1.81800	0.00940	-2.06250
H	0.53280	-1.23390	-2.00130
H	0.32460	0.26830	-2.98040
C	0.02180	2.86880	1.84530
H	1.06960	3.15060	1.95270
H	-0.58680	3.76100	2.01050
H	-0.21750	2.15430	2.63840
C	-0.20120	3.78310	-0.89280
C	1.40240	3.35240	-2.49220
C	0.06390	3.11070	-2.23750
Br	2.34990	2.55640	-3.99710
O	-0.82640	2.44730	-2.82110
C	1.00610	4.63870	-0.56370
H	0.75140	5.65950	-0.86830
H	1.25810	4.69220	0.49650
C	2.15440	4.11210	-1.45520
H	2.86130	3.48520	-0.88090
H	2.75260	4.93610	-1.85960

C	-1.57220	4.36920	-0.72650
H	-1.82570	4.61320	0.30840
H	-1.59200	5.30190	-1.30230
H	-2.31450	3.69880	-1.15660

TS2

37
REACTION2_B_MINOR_O_BOUND_TS_SP_TFE_DEF2_TZVP

C	-1.91150	1.97230	-0.65790
C	-2.42280	2.12820	0.62760
C	-3.62860	1.50700	0.95090
C	-4.32490	0.74180	0.00610
C	-3.82770	0.56610	-1.28410
C	-2.61800	1.17910	-1.58370
C	-0.75470	2.55140	-1.35850
C	-0.77120	1.90370	-2.63750
N	-1.88290	1.16390	-2.77940
H	-1.89530	2.72190	1.36480
H	-4.03600	1.61570	1.94910
H	-5.26240	0.27460	0.28280
H	-4.36410	-0.02470	-2.01670
H	-0.04980	1.99120	-3.43070
C	-2.32220	0.52430	-4.00840
H	-1.57490	0.69360	-4.78080
H	-2.45390	-0.54840	-3.85010
H	-3.27030	0.95890	-4.33490
C	0.56170	2.81060	-0.66260
H	1.22940	3.38700	-1.30540
H	0.41520	3.36410	0.26620
H	1.04990	1.86600	-0.40760
C	-1.43310	4.41960	-1.92610
C	0.42830	5.62830	-2.46990
C	-0.43590	4.68470	-3.00500
Br	1.92310	6.35720	-3.44630
O	-0.46810	4.06900	-4.10040
C	-1.19910	5.36350	-0.76440
H	-2.03780	6.06400	-0.70260
H	-1.16340	4.85400	0.20190
C	0.11340	6.11860	-1.09760
H	0.92580	5.91080	-0.39030
H	-0.04980	7.20270	-1.06980
C	-2.85230	4.22190	-2.38050
H	-3.51460	3.88390	-1.58230
H	-3.21450	5.19470	-2.73240
H	-2.89760	3.54310	-3.23120

INT2

```
37
REACTION2_B_MINOR_O_BOUND_SP_TFE_DEF2_TZVP
C         -1.97490        2.05030       -0.41210
C         -2.53950        1.98850        0.84800
C         -3.68490        1.20690        1.04990
C         -4.23950        0.50170       -0.01500
C         -3.68040        0.55810       -1.29600
C         -2.54970        1.34830       -1.48450
C         -0.76890        2.81390       -0.90710
C         -0.59080        2.21420       -2.34760
N         -1.85340        1.59510       -2.66880
H         -2.09690        2.53520        1.67410
H         -4.13260        1.14420        2.03440
H         -5.12020       -0.10940        0.14790
H         -4.12080        0.00100       -2.11360
H          0.24130        1.50240       -2.42500
C         -1.93220        0.71540       -3.81740
H         -1.38540        1.17160       -4.64290
H         -1.51450       -0.28240       -3.61320
H         -2.97160        0.60070       -4.13020
C          0.48270        2.59120       -0.05640
H          1.35380        3.08300       -0.49810
H          0.34460        2.98100        0.95440
H          0.69450        1.52270        0.02490
C         -1.03560        4.33560       -1.25690
C          0.34250        5.61150       -2.69410
C         -0.25970        4.43690       -2.54440
Br         1.39620        6.17540       -4.21160
O         -0.25380        3.29060       -3.25960
C         -0.40480        5.48200       -0.42590
H         -1.11620        5.92600        0.27390
H          0.45420        5.12980        0.14480
C          0.09650        6.50130       -1.49760
H          1.00670        7.01730       -1.18230
H         -0.65300        7.27170       -1.71400
C         -2.52810        4.63260       -1.51310
H         -3.10600        4.51570       -0.59400
H         -2.63970        5.66040       -1.86670
H         -2.94580        3.96720       -2.26880
```

Reaction 8a + 11 ($R^2 = CH_3$): unobserved regioisomer; major stereoisomer

TS2

```
37
REACTION2_B_NOTOBS1_O_BOUND_TS_DEF2_TZVP(SP_TFE)
```

C	-1.08550	3.36480	0.02670
C	-2.36830	2.84810	-0.12080
C	-2.52980	1.62250	-0.76760
C	-1.42160	0.93000	-1.26850
C	-0.12420	1.41440	-1.09890
C	0.01890	2.62530	-0.43120
C	-0.57240	4.62230	0.60320
C	0.85050	4.41390	0.64980
N	1.18220	3.30090	-0.01580
H	-3.22750	3.38750	0.26060
H	-3.52160	1.20450	-0.89080
H	-1.56870	-0.01330	-1.78140
H	0.72780	0.85690	-1.46600
H	1.60500	5.07460	1.04050
C	2.54900	2.99730	-0.42670
H	3.17970	2.86570	0.45430
H	2.56030	2.08190	-1.01330
H	2.89910	3.84410	-1.02240
C	-1.33470	5.28130	1.72850
H	-0.77290	6.10190	2.16950
H	-2.28540	5.68350	1.37130
H	-1.54730	4.54320	2.50720
C	-0.56270	5.91030	-0.99290
C	0.20450	4.75960	-2.82420
C	-1.79260	5.65970	-1.82860
H	-2.63390	5.26720	-1.25390
C	0.65510	5.53170	-1.75710
O	1.82930	5.76910	-1.36100
H	-2.11870	6.60070	-2.27640
C	-1.28600	4.68680	-2.91990
H	-1.64060	3.66160	-2.75340
H	-1.65700	4.98650	-3.90920
Br	-0.46760	7.71330	-0.15730
C	1.10630	4.07960	-3.78910
H	0.92810	2.99400	-3.78950
H	0.93950	4.42160	-4.81860
H	2.14840	4.27150	-3.52640

INT2

37
REACTION2_B_NOTOBS1_O_BOUND_DEF2_TZVP_SP_TRIFLUOROETHANOL

C	-0.99190	3.31710	-0.00590
C	-2.25480	2.78600	-0.19970
C	-2.40900	1.39720	-0.29630
C	-1.29850	0.56470	-0.18360
C	-0.02250	1.08600	0.04810

C	0.11330	2.46750	0.15460
C	-0.53840	4.76200	0.21450
C	1.02120	4.57510	0.15610
N	1.25400	3.20420	0.48060
H	-3.12150	3.43290	-0.27160
H	-3.39200	0.97320	-0.46160
H	-1.42210	-0.50900	-0.27050
H	0.83220	0.42860	0.14700
H	1.59350	5.25290	0.78840
C	2.59080	2.65220	0.36640
H	3.30760	3.37980	0.75010
H	2.67080	1.74630	0.97110
H	2.85460	2.41630	-0.67190
C	-1.04440	5.24410	1.57770
H	-0.61640	6.20780	1.84770
H	-2.13190	5.34300	1.55680
H	-0.78440	4.50710	2.34060
C	-0.77270	5.65110	-1.02310
C	0.06030	5.40390	-3.21190
C	-1.98280	5.67470	-1.94810
H	-2.42540	4.67710	-1.95590
C	0.34160	5.29120	-1.90830
O	1.43280	4.85880	-1.23390
H	-2.74440	6.39540	-1.65550
C	-1.35120	5.94320	-3.34200
H	-1.91160	5.45420	-4.14520
H	-1.33680	7.01860	-3.56110
Br	-0.41510	7.71320	-0.45110
C	0.95600	5.11450	-4.36710
H	0.48470	4.41350	-5.06530
H	1.17670	6.02760	-4.93340
H	1.90260	4.68670	-4.03170

TS3

37
REACTION2_B_NOTOBS_INT_TS_SP_TFE_DEF2_TZVP

C	-1.46740	0.77400	-0.14160
C	-2.73270	0.58910	0.40950
C	-3.57340	-0.35590	-0.16990
C	-3.17920	-1.09240	-1.29690
C	-1.92340	-0.91830	-1.86760
C	-1.08660	0.01060	-1.25720
C	-0.34960	1.68670	0.17900
C	0.67950	1.28490	-0.72350
N	0.23070	0.36720	-1.59040
H	-3.06720	1.21130	1.22610

H	-4.56420	-0.50770	0.24120
H	-3.86740	-1.80530	-1.73580
H	-1.62100	-1.47380	-2.74650
H	1.68510	1.66800	-0.80120
C	0.91240	-0.07740	-2.79350
H	1.95020	0.25100	-2.76230
H	0.88230	-1.16650	-2.85540
H	0.42370	0.35190	-3.67140
C	-0.02550	2.04210	1.61130
H	0.84780	2.69410	1.67390
H	-0.86790	2.55520	2.07640
H	0.18660	1.13150	2.17820
C	-0.82760	3.49760	-0.63190
C	-1.36300	4.99590	1.03050
C	0.36370	4.42870	-0.52880
H	1.31560	3.90000	-0.43830
C	-1.93060	3.95380	0.27180
O	-3.07310	3.46510	0.33560
H	0.43080	5.03960	-1.43020
C	0.05210	5.31650	0.69810
H	0.73380	5.11720	1.53910
H	0.18960	6.37920	0.45200
Br	-1.39460	3.11870	-2.51340
C	-2.16570	5.77920	2.00530
H	-1.67960	5.80900	2.99040
H	-2.28730	6.82480	1.68920
H	-3.15520	5.32930	2.10700

INT3

37
REACTION2_B_NOTOBS_INT_REDO_SP_TFE_DEF2_TZVP

C	-1.50850	0.73610	-0.24840
C	-2.76410	0.54360	0.30820
C	-3.55780	-0.48860	-0.19700
C	-3.11870	-1.30870	-1.24110
C	-1.85340	-1.14040	-1.79850
C	-1.07820	-0.12030	-1.26440
C	-0.40440	1.74340	0.03320
C	0.65600	1.25570	-0.88640
N	0.24180	0.25340	-1.62520
H	-3.13330	1.22960	1.05650
H	-4.54740	-0.64180	0.21640
H	-3.76880	-2.08670	-1.62310
H	-1.50470	-1.76850	-2.60830
H	1.63270	1.68890	-1.03690
C	0.93830	-0.29760	-2.77960

H	1.96020	0.07580	-2.79820
H	0.94550	-1.38620	-2.71870
H	0.41460	0.01830	-3.68460
C	0.03180	1.74680	1.51350
H	0.89580	2.39400	1.67150
H	-0.79350	2.12880	2.11500
H	0.28240	0.73480	1.84010
C	-0.73810	3.24900	-0.40150
C	-1.32670	4.87910	1.17070
C	0.44160	4.22380	-0.31160
H	1.40250	3.72220	-0.16340
C	-1.87610	3.82800	0.44450
O	-3.02740	3.33820	0.48200
H	0.52370	4.78920	-1.23770
C	0.10170	5.17190	0.86470
H	0.76050	4.97870	1.72730
H	0.27330	6.22390	0.59240
Br	-1.37390	3.13510	-2.44740
C	-2.12430	5.67820	2.14220
H	-1.66310	5.68630	3.14030
H	-2.21330	6.72990	1.83370
H	-3.12890	5.25780	2.22210

Product

37
REACTION2_B_NOTOBS1_DEF2_TZVP_SP_TRIFLUOROETHANOL

C	-1.43610	1.95020	-0.00760
C	-2.64960	2.06000	0.64790
C	-3.57920	1.01860	0.54220
C	-3.26620	-0.11540	-0.20430
C	-2.04460	-0.23450	-0.87290
C	-1.13610	0.82040	-0.78290
C	-0.25840	2.89350	-0.00810
C	0.78530	2.14660	-0.94500
N	0.10400	0.94020	-1.41150
H	-2.87150	2.94560	1.23220
H	-4.53480	1.09030	1.04730
H	-3.98290	-0.92640	-0.27190
H	-1.82010	-1.12340	-1.44940
H	1.68570	1.88340	-0.37610
C	0.88960	-0.23040	-1.74980
H	1.77530	0.07590	-2.30660
H	1.21170	-0.79310	-0.85960
H	0.31450	-0.90040	-2.39210
C	0.31110	3.11010	1.39940
H	1.17930	3.77490	1.38010

H	-0.43750	3.55300	2.05640
H	0.61270	2.14810	1.81960
C	-0.46310	4.22910	-0.79020
C	1.16750	3.18060	-2.05350
C	-1.15450	3.99500	-2.14690
H	-2.04070	3.37270	-2.03190
C	0.98690	4.47990	-1.24700
O	1.74350	5.38400	-1.04940
H	-1.45960	4.95080	-2.57190
C	-0.03800	3.32020	-3.01370
H	-0.34180	2.34550	-3.39340
H	0.23600	3.95540	-3.85910
Br	-1.32010	5.69410	0.25780
C	2.52210	2.99810	-2.70700
H	2.53460	2.11300	-3.34890
H	2.76890	3.86640	-3.32240
H	3.30870	2.89180	-1.95530

Reaction 8a + 11 ($R^2 = CH_3$): observed regioisomer; minor stereoisomer

TS2

37
REACTION2_NOTOBS2_O_BOUND_TS_DEF2_TZVP_SP_TRIFLUOROETHANOL

C	-1.57010	2.27560	0.14060
C	-2.16420	2.72550	1.31670
C	-3.44490	2.27700	1.63310
C	-4.13020	1.39290	0.78900
C	-3.55020	0.92390	-0.38750
C	-2.26920	1.37200	-0.68110
C	-0.30190	2.58710	-0.52880
C	-0.26490	1.69090	-1.64540
N	-1.43480	1.04860	-1.76480
H	-1.64170	3.40800	1.97660
H	-3.92070	2.61520	2.54610
H	-5.12800	1.06590	1.05630
H	-4.08210	0.24630	-1.04360
H	0.51480	1.60390	-2.38380
C	-1.85840	0.28290	-2.92490
H	-0.99970	0.12010	-3.57320
H	-2.26830	-0.67880	-2.61050
H	-2.61590	0.84690	-3.47430
C	0.95140	2.92650	0.23790
H	1.74890	3.23440	-0.43860
H	0.77250	3.73420	0.94970
H	1.28930	2.05490	0.80560
C	-0.67770	4.33410	-1.59460

C	1.30210	5.42330	-1.96490
C	0.52580	4.37360	-2.45990
C	2.61290	5.80010	-2.55710
H	2.90990	5.05120	-3.29410
H	2.57070	6.77750	-3.05750
H	3.39140	5.87980	-1.78630
Br	-2.39650	4.05200	-2.57370
O	0.75870	3.54430	-3.37590
C	-0.70430	5.48980	-0.62710
H	-1.52240	6.16900	-0.87050
H	-0.87110	5.16540	0.40310
C	0.67080	6.17330	-0.83670
H	1.29540	6.15130	0.06780
H	0.52740	7.23720	-1.07380

INT2

37
REACTION2_NOTOBS2_O_BOUND_DEF2_TZVP_SP_TRIFLUOROETHANOL

C	-1.77260	2.17570	-0.12510
C	-2.51340	2.37060	1.02310
C	-3.75400	1.73360	1.15130
C	-4.22140	0.91520	0.12610
C	-3.48040	0.71230	-1.04240
C	-2.25480	1.36140	-1.16190
C	-0.43020	2.73570	-0.52760
C	-0.11100	1.90540	-1.82550
N	-1.36980	1.34810	-2.23610
H	-2.14260	3.01380	1.81380
H	-4.34660	1.87440	2.04700
H	-5.17970	0.41900	0.23270
H	-3.85830	0.07340	-1.83080
H	0.64860	1.12840	-1.66440
C	-1.40550	0.34550	-3.27760
H	-0.70050	0.62970	-4.05920
H	-1.15100	-0.66000	-2.90770
H	-2.40000	0.31040	-3.72640
C	0.65090	2.53700	0.54350
H	1.63380	2.85000	0.18100
H	0.41540	3.10680	1.44460
H	0.70310	1.48150	0.81680
C	-0.38990	4.18810	-1.10200
C	1.21980	5.12520	-2.55480
C	0.48370	4.04320	-2.28060
C	2.20270	5.30500	-3.66090
H	2.35620	4.37050	-4.20370
H	1.85700	6.06100	-4.37630

H	3.16960	5.64990	-3.27740
Br	-2.30720	4.76840	-1.81540
O	0.46160	2.79420	-2.81700
C	0.17910	5.41820	-0.39420
H	-0.56950	5.98760	0.15400
H	0.95290	5.09930	0.30840
C	0.85140	6.20460	-1.55390
H	1.72330	6.77380	-1.21660
H	0.14330	6.91840	-1.99340

Compound 9a

Submitted by: **Jimmy Wu**

Date Collected: **July 5, 2022**

Solved by: **Richard J Staples**

Sample ID: **ZTP-3-143-major**

Center Crystallographic for Research
Michigan State University
Department of Chemistry
East Lansing, MI 48824
Dr. Richard J. Staples
staples@chemistry.msu.edu

Relative chirality determined for the crystal supplied.

Crystal Data and Experimental

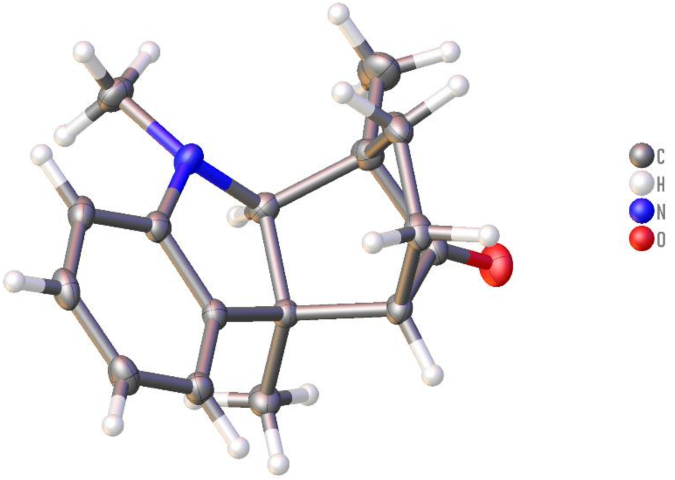

**Compound**	**JW722A**
Formula	$C_{16}H_{19}NO$
$D_{calc.}$/ g cm^{-3}	1.230
μ/mm^{-1}	0.592
Formula Weight	241.32
Color	colourless
Shape	irregular-shaped
Size/mm^3	0.21×0.16×0.14
T/K	100.00(10)
Crystal System	monoclinic
Space Group	$P2_1/n$
a/Å	11.82272(18)
b/Å	8.08217(10)
c/Å	14.6182(2)
α/°	90
β/°	111.1442(18)
γ/°	90
V/Å^3	1302.78(4)
Z	4
Z'	1
Wavelength/Å	1.54184
Radiation type	Cu K$_\alpha$
Θ_{min}/°	4.148
Θ_{max}/°	80.158
Measured Refl's.	17052
Indep't Refl's	2813
Refl's I≥2 σ(I)	2620
R_{int}	0.0319
Parameters	166
Restraints	0
Largest Peak	0.225
Deepest Hole	-0.310
GooF	1.084
wR_2 (all data)	0.1052
wR_2	0.1035
R_1 (all data)	0.0419
R_1	0.0401

Experimental. Single colourless irregular-shaped crystals of **JW722A** used as received. A suitable crystal with dimensions 0.21 × 0.16 × 0.14 mm^3 was selected and mounted on a nylon loop with paratone oil on a XtaLAB Synergy, Dualflex, HyPix diffractometer. The crystal was kept at a steady T = 100.00(10) K during data collection. The structure was solved with the **ShelXT** (Sheldrick, 2015) solution program using dual methods and by using **Olex2** 1.5 (Dolomanov et al., 2009) as the graphical interface. The model was refined with **ShelXL** 2018/3 (Sheldrick, 2015) using full matrix least squares minimisation on $\boldsymbol{F}^2$.

Crystal Data. $C_{16}H_{19}NO$, M_r = 241.32, monoclinic, $P2_1/n$ (No. 14), a = 11.82272(18) Å, b = 8.08217(10) Å, c = 14.6182(2) Å, β = 111.1442(18)°, α = γ = 90°, V = 1302.78(4) Å^3, T = 100.00(10) K, Z = 4, Z' = 1, μ(Cu K$_\alpha$) = 0.592, 17052 reflections measured, 2813 unique (R_{int} = 0.0319) which were used in all calculations. The final wR_2 was 0.1052 (all data) and R_1 was 0.0401 (I≥2 σ(I)).

The Rigaku Synergy S Diffractometer was purchased with Support from the MRI program by the National Science Foundation under Grant No. 1919565.

Structure Quality Indicators

**Reflections:**	d min (Cu\a) 2Θ=160.3° 0.78	I/σ(I) 47.4	Rint 3.19%	Full 135.4° 99% to 160.3° 100
**Refinement:**	Shift 0.001	Max Peak 0.2	Min Peak -0.3	GooF 1.084

A colourless irregular-shaped-shaped crystal with dimensions 0.21×0.16×0.14 mm^3 was mounted on a nylon loop with paratone oil. Data were collected using a XtaLAB Synergy, Dualflex, HyPix diffractometer equipped with an Oxford Cryosystems low-temperature device, operating at T = 100.00(10) K.

MSU Data were measured using ω scans using Cu K_α radiation (micro-focus sealed X-ray tube, 50 kV, 1 mA). The total number of runs and images was based on the strategy calculation from the program CrysAlisPro 1.171.42.60a (Rigaku OD, 2022). The achieved resolution was Θ = 80.158.

Cell parameters were retrieved using the CrysAlisPro 1.171.42.60a (Rigaku OD, 2022) software and refined using CrysAlisPro 1.171.42.60a (Rigaku OD, 2022) on 10444 reflections, 61 % of the observed reflections. Data reduction was performed using the CrysAlisPro 1.171.42.60a (Rigaku OD, 2022) software which corrects for Lorentz polarization. The final completeness is 100.00 out to 80.158 in Θ CrysAlisPro 1.171.42.60a (Rigaku Oxford Diffraction, 2022) Numerical absorption correction based on gaussian integration over a multifaceted crystal model Empirical absorption correction using spherical harmonics, implemented in SCALE3 ABSPACK scaling algorithm.

The structure was solved in the space group $P2_1/n$ (# 14) by using dual methods using the ShelXT (Sheldrick, 2015) structure solution program. The structure was refined by Least Squares ShelXL incorporated in Olex2 software program. All non-hydrogen atoms were refined anisotropically. Hydrogen atom positions were calculated geometrically and refined using the riding model, except for the hydrogen atom on the non-carbon atom(s) which were found by difference Fourier methods and refined isotropically when data permits.

There is a single molecule in the asymmetric unit, which is represented by the reported sum formula. In other words: Z is 4 and Z' is 1.

The Rigaku Synergy S Diffractometer was purchased with Support from the MRI program by the National Science Foundation under Grant No. 1919565.

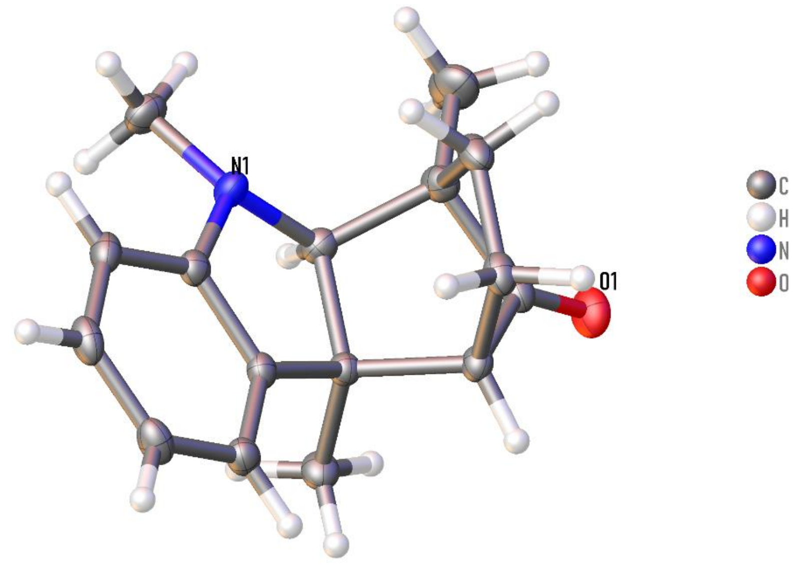

Figure 1:

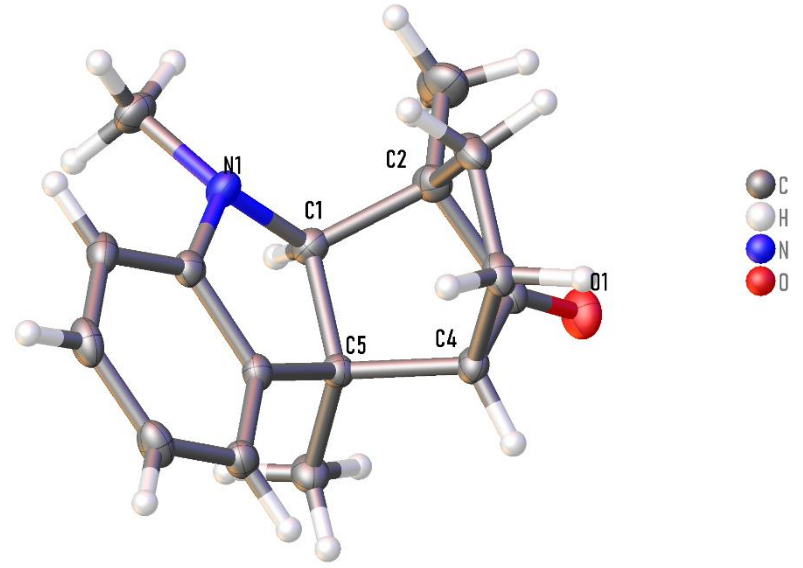

Figure 2: Compound resides in a centrosymmetric space group, relative chirality is shown. Model has Chirality at C1 (Centro SPGR) R. Model has Chirality at C2 (Centro SPGR) R. Model has Chirality at C4 (Centro SPGR) S. Model has Chirality at C5 (Centro SPGR) S.

The Rigaku Synergy S Diffractometer was purchased with Support from the MRI program by the National Science Foundation under Grant No. 1919565.

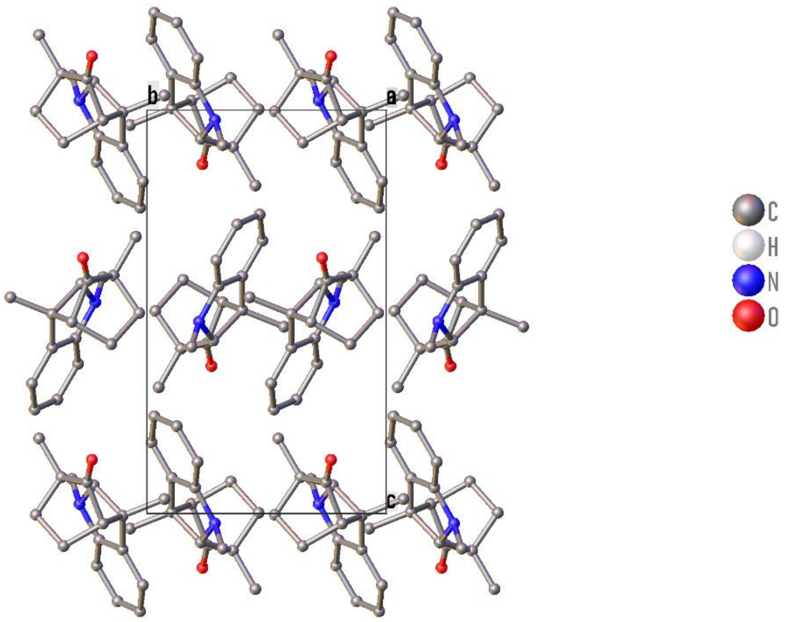

Figure 3: Packing diagram of JW722A.

Data Plots: Diffraction Data

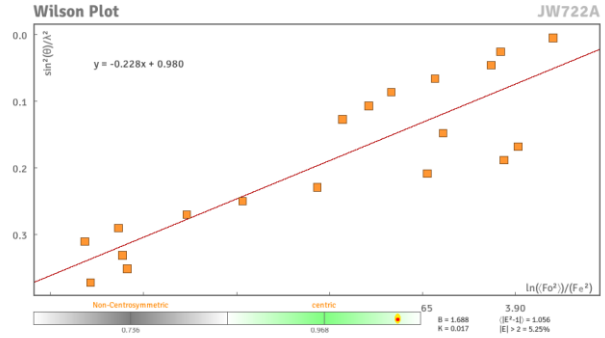

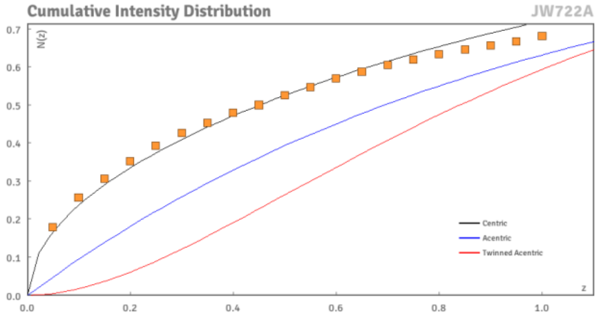

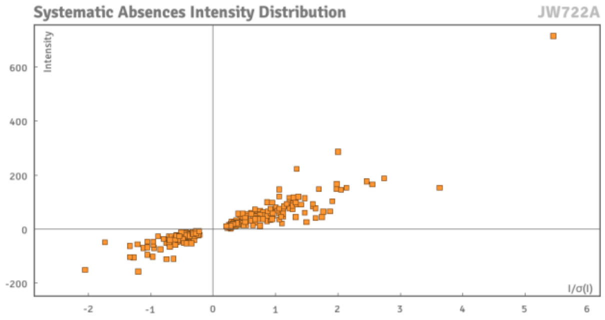

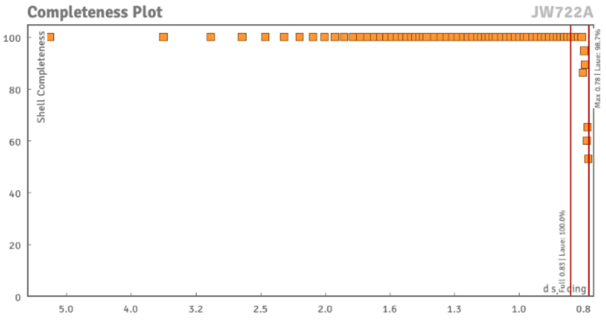

The Rigaku Synergy S Diffractometer was purchased with Support from the MRI program by the National Science Foundation under Grant No. 1919565.

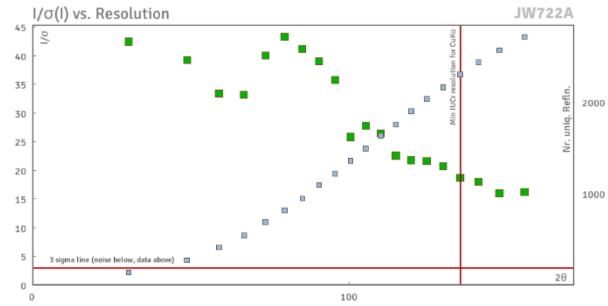

Data Plots: Refinement and Data

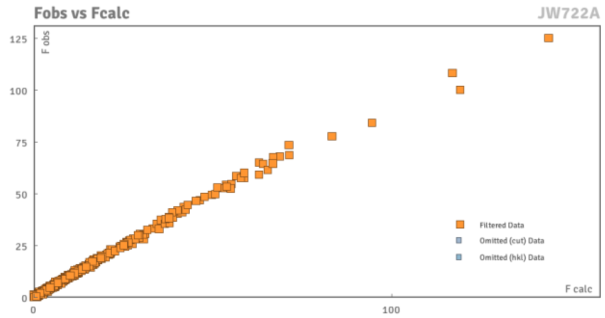

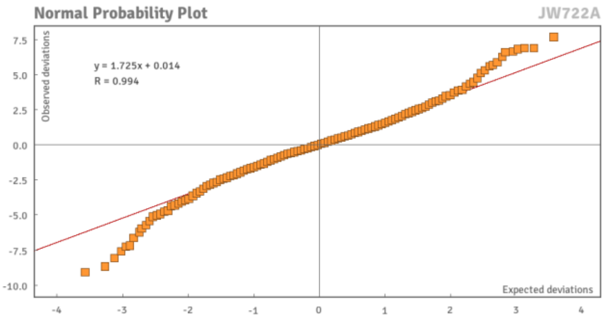

Reflection Statistics

Total reflections (after filtering)	17668	Unique reflections	2813
Completeness	0.987	Mean I/σ	29.28
hkl_{max} collected	(14, 9, 17)	hkl_{min} collected	(-15, -10, -18)
hkl_{max} used	(14, 10, 18)	hkl_{min} used	(-15, 0, 0)
Lim d_{max} collected	100.0	Lim d_{min} collected	0.77
d_{max} used	13.63	d_{min} used	0.78
Friedel pairs	2504	Friedel pairs merged	1
Inconsistent equivalents	2	R_{int}	0.0319
R_{sigma}	0.0211	Intensity transformed	0
Omitted reflections	0	Omitted by user (OMIT hkl)	0
Multiplicity	(3016, 2111, 1281, 679, 369, 187, 61, 34, 8, 10, 3)	Maximum multiplicity	20
Removed systematic absences	616	Filtered off (Shel/OMIT)	0

Selected Crystal Pictures

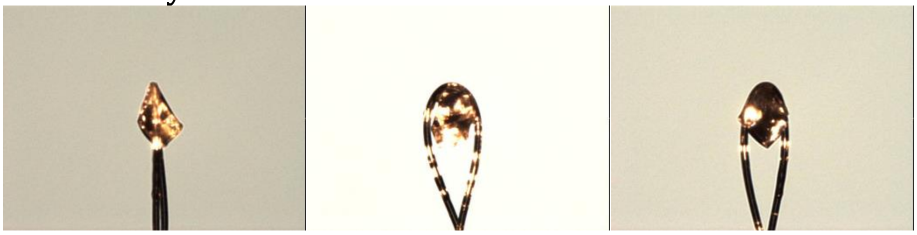

The Rigaku Synergy S Diffractometer was purchased with Support from the MRI program by the National Science Foundation under Grant No. 1919565.

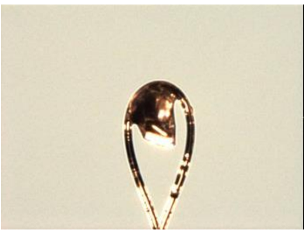

Table 1: Fractional Atomic Coordinates (×10^4) and Equivalent Isotropic Displacement Parameters (Å^2×10^3) for **JW722A**. U_{eq} is defined as 1/3 of the trace of the orthogonalised U_{ij}.

Atom	x	y	z	U_{eq}
O1	9326.2(7)	2313.3(10)	8661.4(6)	26.8(2)
N1	6022.6(7)	2828.9(11)	9759.7(6)	18.9(2)
C1	6855.2(9)	2235.9(12)	9293.3(7)	17.8(2)
C2	7671.1(10)	3589.9(13)	9091.2(7)	21.3(2)
C3	8791.6(9)	2520.1(13)	9216.7(7)	19.3(2)
C4	9023.3(9)	1850.3(12)	10243.6(7)	17.1(2)
C5	7768.9(8)	1025.2(12)	10047.4(7)	15.5(2)
C6	7314.9(8)	1028.2(12)	10889.0(7)	15.9(2)
C7	7748.7(9)	181.6(13)	11767.9(7)	19.6(2)
C8	7124.4(10)	297.9(14)	12415.8(7)	23.3(2)
C9	6076.9(10)	1238.7(13)	12168.6(8)	23.1(2)
C10	5632.8(9)	2106.9(13)	11283.1(8)	20.6(2)
C11	6271.1(9)	2007.6(12)	10649.2(7)	16.9(2)
C13	8212.3(10)	4660.0(13)	10024.2(8)	23.4(2)
C14	9116.6(9)	3494.5(13)	10798.0(7)	20.8(2)
C16	7128.8(12)	4519.8(17)	8135.6(9)	34.8(3)
C17	4769.9(9)	3152.6(14)	9137.4(8)	25.3(2)
C18	7749.9(10)	-718.1(13)	9634.6(8)	21.7(2)

Table 2: Anisotropic Displacement Parameters (×10^4) for **JW722A**. The anisotropic displacement factor exponent takes the form: $-2\pi^2[h^2a^{*2} \times U_{11}+ ... +2hka^* \times b^* \times U_{12}]$

Atom	U_{11}	U_{22}	U_{33}	U_{23}	U_{13}	U_{12}
O1	30.0(4)	32.0(4)	25.7(4)	-4.3(3)	18.9(3)	-3.6(3)
N1	15.2(4)	22.3(4)	19.5(4)	2.3(3)	6.8(3)	2.7(3)
C1	17.8(5)	20.0(5)	15.6(4)	-0.3(3)	6.1(4)	1.2(4)
C2	25.2(5)	21.1(5)	21.3(5)	4.3(4)	12.8(4)	2.2(4)
C3	22.0(5)	18.2(5)	20.6(5)	-2.9(4)	11.3(4)	-4.6(4)
C4	16.1(4)	18.4(5)	18.9(5)	-1.7(4)	8.8(4)	-0.8(3)
C5	15.5(4)	15.5(4)	16.2(4)	-0.6(3)	6.7(3)	-0.7(3)
C6	15.0(4)	16.5(4)	17.1(4)	-1.7(3)	6.9(4)	-3.1(3)
C7	17.9(5)	20.4(5)	20.2(5)	1.7(4)	6.5(4)	-2.1(4)
C8	25.4(5)	27.0(5)	17.9(5)	1.5(4)	8.2(4)	-7.6(4)
C9	25.8(5)	25.7(5)	23.5(5)	-5.7(4)	15.7(4)	-9.4(4)
C10	17.5(5)	20.5(5)	26.7(5)	-5.0(4)	11.6(4)	-3.3(4)
C11	15.8(4)	16.5(4)	18.2(5)	-2.1(3)	6.1(4)	-3.0(3)
C13	27.7(5)	16.2(5)	32.5(6)	-1.9(4)	18.3(5)	-2.3(4)
C14	22.1(5)	20.9(5)	23.0(5)	-6.3(4)	12.3(4)	-6.4(4)
C16	39.5(7)	38.3(7)	30.7(6)	16.3(5)	17.6(5)	7.1(5)
C17	17.3(5)	27.9(6)	26.9(5)	1.0(4)	3.1(4)	3.8(4)
C18	23.2(5)	17.7(5)	26.5(5)	-4.6(4)	11.5(4)	-1.7(4)

The Rigaku Synergy S Diffractometer was purchased with Support from the MRI program by the National Science Foundation under Grant No. 1919565.

Table 3: Bond Lengths in Å for **JW722A**.

Atom	Atom	Length/Å
O1	C3	1.2068(12)
N1	C1	1.4655(12)
N1	C11	1.3936(13)
N1	C17	1.4552(13)
C1	C2	1.5559(14)
C1	C5	1.5750(13)
C2	C3	1.5365(14)
C2	C13	1.5453(15)
C2	C16	1.5103(14)
C3	C4	1.5248(13)
C4	C5	1.5554(13)
C4	C14	1.5396(13)
C5	C6	1.5092(12)
C5	C18	1.5298(13)
C6	C7	1.3808(13)
C6	C11	1.4001(13)
C7	C8	1.3983(14)
C8	C9	1.3855(16)
C9	C10	1.3976(15)
C10	C11	1.3926(13)
C13	C14	1.5612(15)

Table 4: Bond Angles in ° for **JW722A**.

Atom	Atom	Atom	Angle/°
C11	N1	C1	109.11(8)
C11	N1	C17	119.57(8)
C17	N1	C1	118.10(8)
N1	C1	C2	115.14(8)
N1	C1	C5	106.48(7)
C2	C1	C5	104.73(8)
C3	C2	C1	98.53(8)
C3	C2	C13	98.33(8)
C13	C2	C1	108.28(8)
C16	C2	C1	116.12(9)
C16	C2	C3	116.78(9)
C16	C2	C13	116.12(10)
O1	C3	C2	129.48(10)
O1	C3	C4	131.40(10)
C4	C3	C2	99.11(8)
C3	C4	C5	98.62(7)
C3	C4	C14	99.50(8)
C14	C4	C5	110.47(8)
C4	C5	C1	103.31(7)
C6	C5	C1	102.34(7)
C6	C5	C4	116.41(8)
C6	C5	C18	111.35(8)
C18	C5	C1	112.79(8)
C18	C5	C4	110.18(8)
C7	C6	C5	129.47(9)
C7	C6	C11	120.74(9)
C11	C6	C5	109.72(8)
C6	C7	C8	119.16(9)
C9	C8	C7	120.05(10)
C8	C9	C10	121.26(9)
C11	C10	C9	118.34(9)
N1	C11	C6	111.90(8)
C10	C11	N1	127.66(9)
C10	C11	C6	120.42(9)
C2	C13	C14	105.02(8)
C4	C14	C13	103.83(8)

Table 5: Torsion Angles in ° for **JW722A**.

Atom	Atom	Atom	Atom	Angle/°
O1	C3	C4	C5	-123.88(12)
O1	C3	C4	C14	123.55(12)
N1	C1	C2	C3	148.13(8)
N1	C1	C2	C13	46.37(11)
N1	C1	C2	C16	-86.35(11)
N1	C1	C5	C4	-119.85(8)
N1	C1	C5	C6	1.46(9)
N1	C1	C5	C18	121.21(9)
C1	N1	C11	C6	7.17(11)
C1	N1	C11	C10	-174.51(9)
C1	C2	C3	O1	125.85(11)
C1	C2	C3	C4	-55.25(8)
C1	C2	C13	C14	68.65(10)
C1	C5	C6	C7	179.48(10)

The Rigaku Synergy S Diffractometer was purchased with Support from the MRI program by the National Science Foundation under Grant No. 1919565.

Atom	Atom	Atom	Atom	Angle/°
C1	C5	C6	C11	2.65(10)
C2	C1	C5	C4	2.55(9)
C2	C1	C5	C6	123.86(8)
C2	C1	C5	C18	-116.39(9)
C2	C3	C4	C5	57.25(9)
C2	C3	C4	C14	-55.33(9)
C2	C13	C14	C4	0.19(10)
C3	C2	C13	C14	-33.25(9)
C3	C4	C5	C1	-35.99(9)
C3	C4	C5	C6	-147.26(8)
C3	C4	C5	C18	84.74(9)
C3	C4	C14	C13	33.36(9)
C4	C5	C6	C7	-68.69(13)
C4	C5	C6	C11	114.48(9)
C5	C1	C2	C3	31.55(9)
C5	C1	C2	C13	-70.21(9)
C5	C1	C2	C16	157.07(9)
C5	C4	C14	C13	-69.63(9)
C5	C6	C7	C8	-175.84(9)
C5	C6	C11	N1	-6.20(11)
C5	C6	C11	C10	175.35(8)
C6	C7	C8	C9	0.71(15)
C7	C6	C11	N1	176.65(9)
C7	C6	C11	C10	-1.81(14)
C7	C8	C9	C10	-1.02(16)
C8	C9	C10	C11	-0.08(15)
C9	C10	C11	N1	-176.71(9)
C9	C10	C11	C6	1.47(14)
C11	N1	C1	C2	-120.69(9)
C11	N1	C1	C5	-5.12(10)
C11	C6	C7	C8	0.69(15)
C13	C2	C3	O1	-124.12(11)
C13	C2	C3	C4	54.79(9)
C14	C4	C5	C1	67.59(9)
C14	C4	C5	C6	-43.68(11)
C14	C4	C5	C18	-171.68(8)
C16	C2	C3	O1	0.79(17)
C16	C2	C3	C4	179.70(10)
C16	C2	C13	C14	-158.63(9)
C17	N1	C1	C2	98.33(11)
C17	N1	C1	C5	-146.10(9)
C17	N1	C11	C6	147.48(9)
C17	N1	C11	C10	-34.20(15)
C18	C5	C6	C7	58.73(13)
C18	C5	C6	C11	-118.10(9)

Table 6: Hydrogen Fractional Atomic Coordinates (×10^4) and Equivalent Isotropic Displacement Parameters (Å^2×10^3) for **JW722A**. U_{eq} is defined as 1/3 of the trace of the orthogonalised U_{ij}.

Atom	x	y	z	U_{eq}
H1	6394.33	1632.5	8673.23	21
H4	9739.41	1098.83	10512.06	21
H7	8462.37	-471.68	11930.31	24
H8	7418.65	-268.66	13026.03	28
H9	5652.45	1294.85	12609.45	28

The Rigaku Synergy S Diffractometer was purchased with Support from the MRI program by the National Science Foundation under Grant No. 1919565.

Atom	x	y	z	U_{eq}
H10	4913.39	2748.66	11117.75	25
H13A	7567.49	5048.22	10255.55	28
H13B	8640.44	5633.81	9895.14	28
H14A	9952.39	3939.84	11017.72	25
H14B	8877.29	3348.85	11375.9	25
H16A	6477.23	5241.98	8165.56	52
H16B	6799.16	3729.75	7595.8	52
H16C	7758.2	5191	8025.41	52
H17A	4331.08	2102.52	8956.46	38
H17B	4750.34	3731.57	8542.81	38
H17C	4384.53	3841.78	9493.75	38
H18A	8324.1	-1422.37	10134.11	33
H18B	7981.91	-667.43	9055.69	33
H18C	6931.68	-1181.31	9449.43	33

Citations

CrysAlisPro (Rigaku, V1.171.42.60a, 2022)

CrysAlisPro (ROD), Rigaku Oxford Diffraction, Poland (?).

O.V. Dolomanov and L.J. Bourhis and R.J. Gildea and J.A.K. Howard and H. Puschmann, Olex2: A complete structure solution, refinement and analysis program, *J. Appl. Cryst.*, (2009), **42**, 339-341.

Sheldrick, G.M., Crystal structure refinement with ShelXL, *Acta Cryst.*, (2015), **C71**, 3-8.

Sheldrick, G.M., ShelXT-Integrated space-group and crystal-structure determination, *Acta Cryst.*, (2015), **A71**, 3-8.

The Rigaku Synergy S Diffractometer was purchased with Support from the MRI program by the National Science Foundation under Grant No. 1919565.

Compound 13a

Submitted by: **Jimmy Wu**

Date Collected: **July 5, 2022**

Solved by: **Richard J Staples**

Sample ID: **ZTP-3-143-minor**

Center Crystallographic for Research
Michigan State University
Department of Chemistry
East Lansing, MI 48824
Dr. Richard J. Staples
staples@chemistry.msu.edu

Relative chirality determined for the crystal supplied.

Crystal Data and Experimental

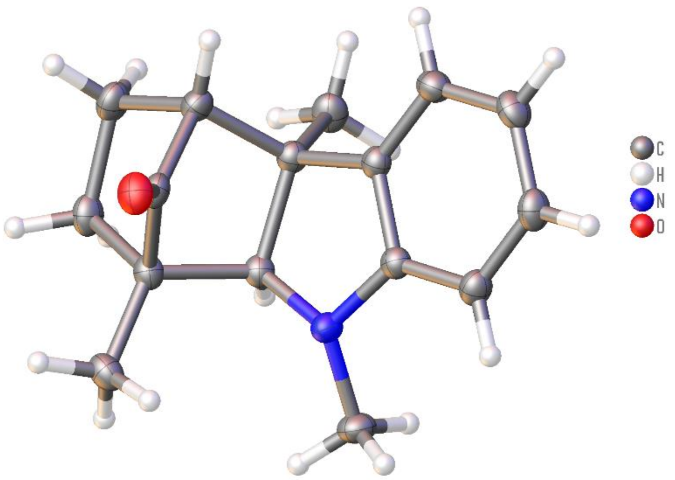

**Compound**	**JW722B**
Formula	$C_{16}H_{19}NO$
$D_{calc.}$/ g cm^{-3}	1.236
μ/mm^{-1}	0.595
Formula Weight	241.32
Color	colourless
Shape	irregular-shaped
Size/mm^3	0.22×0.20×0.06
T/K	100.00(10)
Crystal System	monoclinic
Space Group	$P2_1/c$
a/Å	16.0584(2)
b/Å	6.13979(7)
c/Å	13.79498(19)
α/°	90
β/°	107.5140(15)
γ/°	90
V/Å^3	1297.07(3)
Z	4
Z'	1
Wavelength/Å	1.54184
Radiation type	Cu K_α
Θ_{min}/°	2.886
Θ_{max}/°	79.865
Measured Refl's.	18674
Indep't Refl's	2798
Refl's I≥2 σ(I)	2570
R_{int}	0.0363
Parameters	167
Restraints	0
Largest Peak	0.306
Deepest Hole	-0.192
GooF	1.078
wR_2 (all data)	0.1011
wR_2	0.0987
R_1 (all data)	0.0402
R_1	0.0376

Experimental. Single colourless irregular-shaped crystals of **JW722B** Used as recieved. A suitable crystal with dimensions 0.22 × 0.20 × 0.06 mm^3 was selected and mounted on a nylon loop with paratone oil on a XtaLAB Synergy, Dualflex, HyPix diffractometer. The crystal was kept at a steady T = 100.00(10) K during data collection. The structure was solved with the **ShelXT** (Sheldrick, 2015) solution program using dual methods and by using **Olex2** 1.5 (Dolomanov et al., 2009) as the graphical interface. The model was refined with **ShelXL** 2018/3 (Sheldrick, 2015) using full matrix least squares minimisation on $\boldsymbol{F^2}$.

Crystal Data. $C_{16}H_{19}NO$, M_r = 241.32, monoclinic, $P2_1/c$ (No. 14), a = 16.0584(2) Å, b = 6.13979(7) Å, c = 13.79498(19) Å, β = 107.5140(15)°, α = γ = 90°, V = 1297.07(3) Å^3, T = 100.00(10) K, Z = 4, Z' = 1, μ(Cu K_α) = 0.595, 18674 reflections measured, 2798 unique (R_{int} = 0.0363) which were used in all calculations. The final wR_2 was 0.1011 (all data) and R_1 was 0.0376 (I≥2 σ(I)).

The Rigaku Synergy S Diffractometer was purchased with Support from the MRI program by the National Science Foundation under Grant No. 1919565.

Structure Quality Indicators

**Reflections:**	d min (Cu\a) 2Θ=159.7° 0.78	I/σ(I) 40.5	Rint 3.63%	Full 135.4° 99% to 159.7° 100
**Refinement:**	Shift -0.001	Max Peak 0.3	Min Peak -0.2	GooF 1.078

A colourless irregular-shaped-shaped crystal with dimensions 0.22×0.20×0.06 mm^3 was mounted on a nylon loop with paratone oil. Data were collected using a XtaLAB Synergy, Dualflex, HyPix diffractometer equipped with an Oxford Cryosystems low-temperature device, operating at T = 100.00(10) K.

MSU Data were measured using ω scans using Cu K_α radiation (micro-focus sealed X-ray tube, 50 kV, 1 mA). The total number of runs and images was based on the strategy calculation from the program CrysAlisPro 1.171.42.60a (Rigaku OD, 2022). The achieved resolution was Θ = 79.865.

Cell parameters were retrieved using the CrysAlisPro 1.171.42.60a (Rigaku OD, 2022) software and refined using CrysAlisPro 1.171.42.60a (Rigaku OD, 2022) on 11062 reflections, 59 % of the observed reflections. Data reduction was performed using the CrysAlisPro 1.171.42.60a (Rigaku OD, 2022) software which corrects for Lorentz polarization. The final completeness is 100.00 out to 79.865 in Θ CrysAlisPro 1.171.42.60a (Rigaku Oxford Diffraction, 2022) Numerical absorption correction based on gaussian integration over a multifaceted crystal model Empirical absorption correction using spherical harmonics, implemented in SCALE3 ABSPACK scaling algorithm.

The structure was solved in the space group $P2_1/c$ (# 14) by using dual methods using the ShelXT (Sheldrick, 2015) structure solution program. The structure was refined by Least Squares ShelXL incorporated in Olex2 software program. All non-hydrogen atoms were refined anisotropically. Hydrogen atom positions were calculated geometrically and refined using the riding model, except for the hydrogen atom on the non-carbon atom(s) which were found by difference Fourier methods and refined isotropically when data permits.

There is a single molecule in the asymmetric unit, which is represented by the reported sum formula. In other words: Z is 4 and Z' is 1.

The Rigaku Synergy S Diffractometer was purchased with Support from the MRI program by the National Science Foundation under Grant No. 1919565.

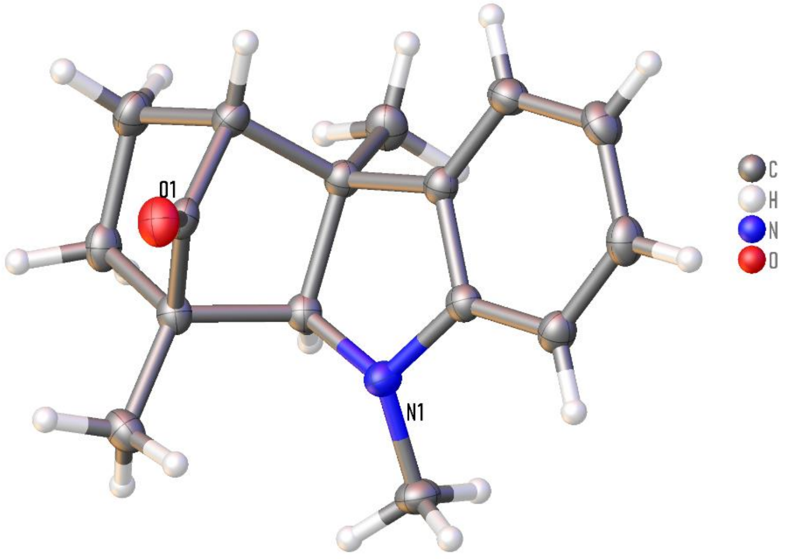

Figure 4:

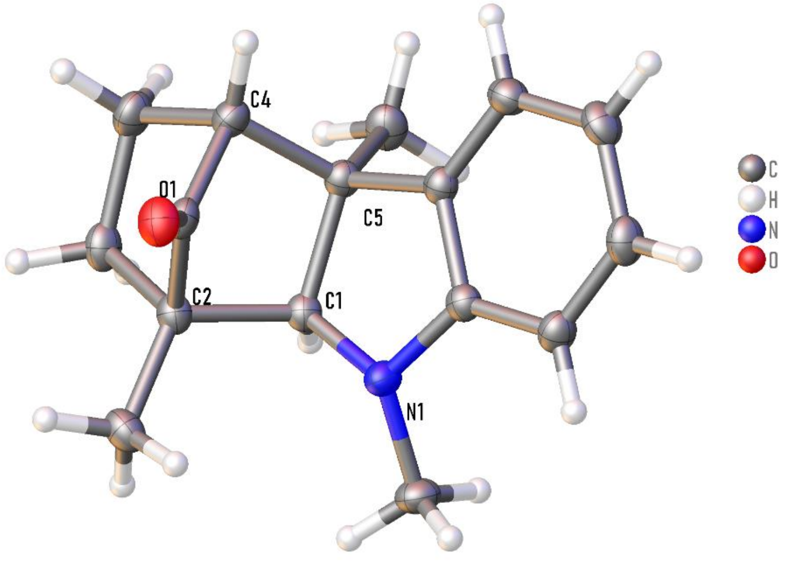

Figure 5: Compound resides in a centrosymmetric space group, relative chirality is shown. Model has Chirality at C1 (Centro SPGR) R. Model has Chirality at C2 (Centro SPGR) S. Model has Chirality at C4 (Centro SPGR) R. Model has Chirality at C5 (Centro SPGR) S.

The Rigaku Synergy S Diffractometer was purchased with Support from the MRI program by the National Science Foundation under Grant No. 1919565.

Data Plots: Diffraction Data

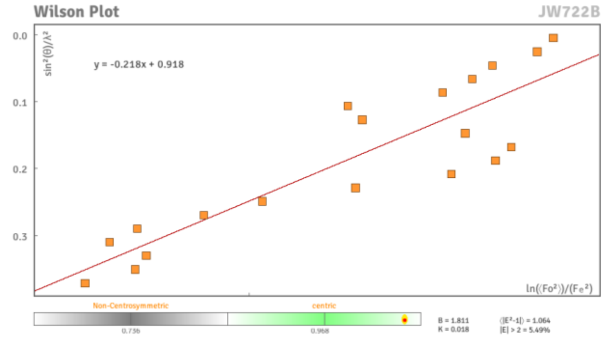

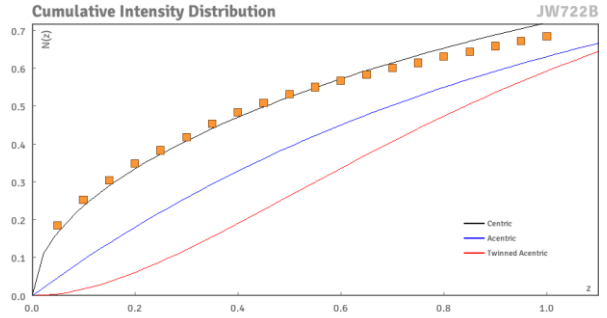

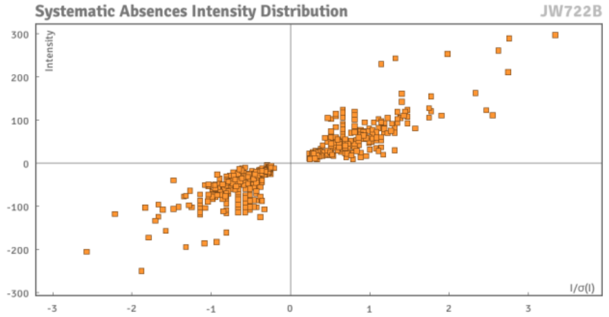

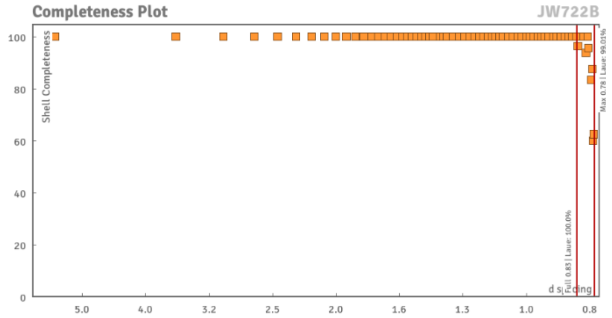

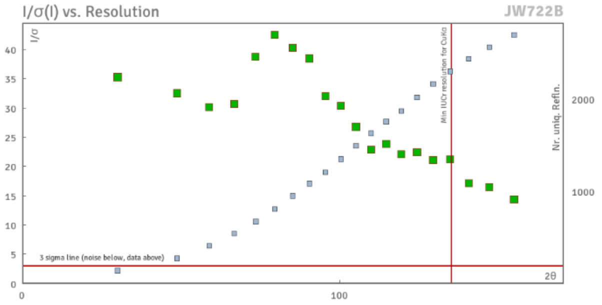

Data Plots: Refinement and Data

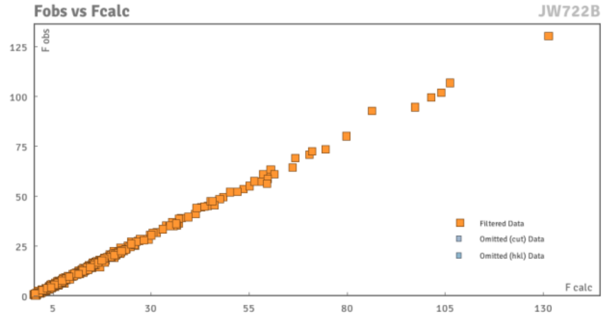

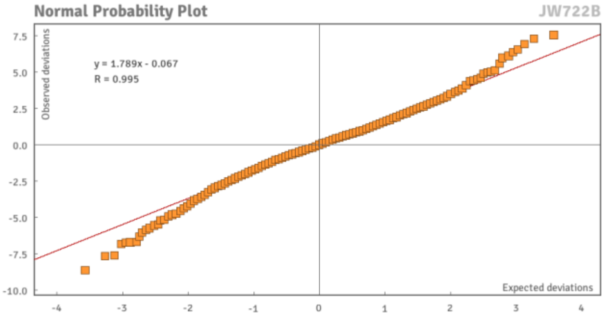

Reflection Statistics

Total reflections (after filtering)	19780	Unique reflections	2798
Completeness	0.99	Mean I/σ	28.11
hkl_{max} collected	(20, 7, 14)	hkl_{min} collected	(-20, -7, -17)
hkl_{max} used	(19, 7, 17)	hkl_{min} used	(-20, 0, 0)
Lim d_{max} collected	100.0	Lim d_{min} collected	0.77

The Rigaku Synergy S Diffractometer was purchased with Support from the MRI program by the National Science Foundation under Grant No. 1919565.

d_{max} used	15.31	d_{min} used	0.78
Friedel pairs	1508	Friedel pairs merged	1
Inconsistent equivalents	0	R_{int}	0.0363
R_{sigma}	0.0247	Intensity transformed	0
Omitted reflections	0	Omitted by user (OMIT hkl)	0
Multiplicity	(2284, 1692, 1117, 636, 421, 274, 167, 111, 82, 75, 47, 25, 6, 2)	Maximum multiplicity	24
Removed systematic absences	1106	Filtered off (Shel/OMIT)	0

Selected Crystal Pictures

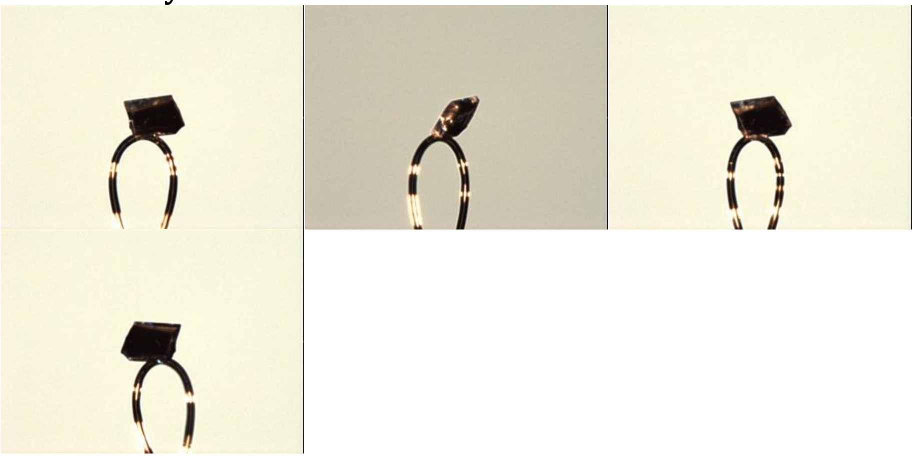

Table 7: Fractional Atomic Coordinates (×10[4]) and Equivalent Isotropic Displacement Parameters (Å[2]×10[3]) for **JW722B**. U_{eq} is defined as 1/3 of the trace of the orthogonalised U_{ij}.

Atom	x	y	z	U_{eq}
O1	1377.8(5)	9716.6(12)	7959.7(6)	26.29(19)
N1	2919.4(5)	5244.5(14)	8459.6(6)	20.6(2)
C11	3532.0(6)	6733.5(16)	8338.8(7)	19.0(2)
C1	2224.1(6)	4976.6(16)	7495.7(7)	18.7(2)
C3	1444.6(6)	8213.5(16)	7424.2(8)	20.0(2)
C6	3276.7(6)	7674.9(16)	7370.2(7)	19.0(2)
C5	2453.1(6)	6583.2(16)	6712.0(7)	19.3(2)
C16	1057.6(7)	5089.6(18)	8467.5(8)	25.2(2)
C10	4311.6(7)	7346.4(18)	9056.5(8)	23.2(2)
C2	1321.7(6)	5772.5(16)	7548.7(7)	19.9(2)
C17	3193.1(7)	3341.4(18)	9102.5(8)	26.3(2)
C8	4560.5(7)	9924.3(18)	7837.4(9)	25.9(2)
C9	4816.9(7)	8945.7(18)	8786.9(8)	25.5(2)
C13	663.6(7)	5248.0(18)	6499.6(8)	25.0(2)
C7	3780.0(7)	9279.9(18)	7121.8(8)	23.4(2)
C4	1663.4(6)	8174.7(17)	6421.6(8)	21.7(2)
C14	837.5(7)	7008.2(19)	5765.4(8)	26.9(2)
C18	2625.9(7)	5413.4(19)	5813.5(8)	26.4(2)

Table 8: Anisotropic Displacement Parameters (×10[4]) for **JW722B**. The anisotropic displacement factor exponent takes the form: $-2\pi^2[h^2a^{*2} \times U_{11} + ... +2hka^* \times b^* \times U_{12}]$

Atom	U_{11}	U_{22}	U_{33}	U_{23}	U_{13}	U_{12}
O1	25.1(4)	21.8(4)	32.6(4)	-6.4(3)	9.7(3)	1.3(3)

The Rigaku Synergy S Diffractometer was purchased with Support from the MRI program by the National Science Foundation under Grant No. 1919565.

Atom	U_{11}	U_{22}	U_{33}	U_{23}	U_{13}	U_{12}
N1	19.1(4)	20.5(4)	20.7(4)	2.9(3)	3.9(3)	-1.0(3)
C11	19.0(5)	18.6(5)	20.0(5)	-1.0(4)	6.9(4)	1.2(4)
C1	18.4(4)	17.4(5)	20.1(5)	-2.1(3)	5.7(4)	-0.4(3)
C3	14.3(4)	20.5(5)	23.4(5)	-1.2(4)	2.8(4)	1.7(3)
C6	17.6(4)	20.2(5)	19.1(5)	-1.5(4)	5.6(4)	1.2(4)
C5	18.6(5)	20.8(5)	17.9(4)	-1.2(4)	4.7(4)	0.2(4)
C16	25.9(5)	23.9(5)	29.8(5)	-2.1(4)	14.3(4)	-2.2(4)
C10	20.9(5)	27.0(5)	20.3(5)	-1.8(4)	4.1(4)	0.9(4)
C2	18.1(4)	19.2(5)	22.7(5)	-3.5(4)	6.5(4)	-0.9(4)
C17	29.5(5)	24.2(5)	24.2(5)	6.6(4)	6.7(4)	1.5(4)
C8	23.9(5)	22.6(5)	33.5(6)	-2.4(4)	12.1(4)	-4.7(4)
C9	19.4(5)	27.8(5)	27.7(5)	-7.5(4)	4.9(4)	-2.5(4)
C13	18.6(5)	27.3(5)	27.3(5)	-6.9(4)	4.3(4)	-2.3(4)
C7	24.8(5)	22.8(5)	24.2(5)	2.6(4)	9.7(4)	0.1(4)
C4	20.2(5)	23.1(5)	20.2(5)	1.1(4)	3.6(4)	1.6(4)
C14	20.5(5)	34.1(6)	22.5(5)	-2.1(4)	1.1(4)	1.5(4)
C18	25.5(5)	32.9(6)	21.0(5)	-5.6(4)	7.5(4)	-0.1(4)

Table 9: Bond Lengths in Å for **JW722B**.

Atom	Atom	Length/Å
O1	C3	1.2069(13)
N1	C11	1.3893(13)
N1	C1	1.4657(12)
N1	C17	1.4528(13)
C11	C6	1.3992(14)
C11	C10	1.3938(14)
C1	C5	1.5863(14)
C1	C2	1.5517(13)
C3	C2	1.5281(14)
C3	C4	1.5263(14)
C6	C5	1.5165(13)
C6	C7	1.3815(14)
C5	C4	1.5549(14)
C5	C18	1.5286(14)
C16	C2	1.5118(14)
C10	C9	1.3938(16)
C2	C13	1.5475(13)
C8	C9	1.3862(16)
C8	C7	1.3990(15)
C13	C14	1.5627(16)
C4	C14	1.5391(14)

Table 10: Bond Angles in ° for **JW722B**.

Atom	Atom	Atom	Angle/°
C11	N1	C1	109.80(8)
C11	N1	C17	120.49(8)
C17	N1	C1	118.13(8)
N1	C11	C6	111.82(8)
N1	C11	C10	127.56(9)
C10	C11	C6	120.61(9)
N1	C1	C5	106.19(7)
N1	C1	C2	112.90(8)
C2	C1	C5	104.34(8)
O1	C3	C2	130.01(10)
O1	C3	C4	130.82(10)
C4	C3	C2	99.17(8)
C11	C6	C5	109.96(9)
C7	C6	C11	120.42(9)
C7	C6	C5	129.54(9)
C6	C5	C1	101.90(7)
C6	C5	C4	111.91(8)
C6	C5	C18	110.78(8)
C4	C5	C1	103.18(8)
C18	C5	C1	113.35(8)
C18	C5	C4	114.79(8)
C9	C10	C11	118.12(9)
C3	C2	C1	98.60(8)
C3	C2	C13	100.02(8)
C16	C2	C1	116.98(9)
C16	C2	C3	116.39(9)
C16	C2	C13	116.20(9)
C13	C2	C1	105.96(8)
C9	C8	C7	119.51(10)
C8	C9	C10	121.75(10)
C2	C13	C14	104.91(8)
C6	C7	C8	119.57(10)
C3	C4	C5	99.71(8)
C3	C4	C14	98.49(8)

The Rigaku Synergy S Diffractometer was purchased with Support from the MRI program by the National Science Foundation under Grant No. 1919565.

Atom	Atom	Atom	Angle/°
C14	C4	C5	110.53(8)

Atom	Atom	Atom	Angle/°
C4	C14	C13	103.36(8)

Table 11: Torsion Angles in ° for **JW722B**.

Atom	Atom	Atom	Atom	Angle/°
O1	C3	C2	C1	-124.58(11)
O1	C3	C2	C16	1.39(16)
O1	C3	C2	C13	127.41(11)
O1	C3	C4	C5	125.05(11)
O1	C3	C4	C14	-122.28(11)
N1	C11	C6	C5	-6.29(11)
N1	C11	C6	C7	176.70(9)
N1	C11	C10	C9	-177.45(10)
N1	C1	C5	C6	-1.85(9)
N1	C1	C5	C4	-118.02(8)
N1	C1	C5	C18	117.22(9)
N1	C1	C2	C3	80.19(9)
N1	C1	C2	C16	-45.37(12)
N1	C1	C2	C13	-176.74(8)
C11	N1	C1	C5	-1.65(10)
C11	N1	C1	C2	-115.39(9)
C11	C6	C5	C1	4.77(10)
C11	C6	C5	C4	114.40(9)
C11	C6	C5	C18	-116.11(9)
C11	C6	C7	C8	1.59(15)
C11	C10	C9	C8	0.27(16)
C1	N1	C11	C6	4.96(11)
C1	N1	C11	C10	-176.29(10)
C1	C5	C4	C3	32.42(9)
C1	C5	C4	C14	-70.53(10)
C1	C2	C13	C14	-75.34(10)
C3	C2	C13	C14	26.69(10)
C3	C4	C14	C13	-39.22(9)
C6	C11	C10	C9	1.20(15)
C6	C5	C4	C3	-76.39(9)
C6	C5	C4	C14	-179.33(8)
C5	C1	C2	C3	-34.68(9)
C5	C1	C2	C16	-160.23(8)
C5	C1	C2	C13	68.40(9)
C5	C6	C7	C8	-174.77(10)
C5	C4	C14	C13	64.55(10)
C16	C2	C13	C14	152.85(9)
C10	C11	C6	C5	174.87(9)
C10	C11	C6	C7	-2.15(15)
C2	C1	C5	C6	117.65(8)
C2	C1	C5	C4	1.49(9)
C2	C1	C5	C18	-123.28(9)
C2	C3	C4	C5	-55.64(8)
C2	C3	C4	C14	57.03(9)
C2	C13	C14	C4	7.80(10)
C17	N1	C11	C6	147.48(9)
C17	N1	C11	C10	-33.78(15)
C17	N1	C1	C5	-145.16(9)
C17	N1	C1	C2	101.10(10)
C9	C8	C7	C6	-0.13(16)

The Rigaku Synergy S Diffractometer was purchased with Support from the MRI program by the National Science Foundation under Grant No. 1919565.

Atom	Atom	Atom	Atom	Angle/°
C7	C6	C5	C1	-178.57(10)
C7	C6	C5	C4	-68.94(13)
C7	C6	C5	C18	60.56(14)
C7	C8	C9	C10	-0.81(16)
C4	C3	C2	C1	56.10(8)
C4	C3	C2	C16	-177.93(8)
C4	C3	C2	C13	-51.90(9)
C18	C5	C4	C3	156.24(9)
C18	C5	C4	C14	53.29(12)

Table 12: Hydrogen Fractional Atomic Coordinates (×10[4]) and Equivalent Isotropic Displacement Parameters (Å^2×10^3) for **JW722B**. U_{eq} is defined as 1/3 of the trace of the orthogonalised U_{ij}.

Atom	x	y	z	U_{eq}
H1	2191.79	3435.55	7252.48	22
H16A	990.25	3503.11	8466.66	38
H16B	502.32	5783.9	8443.83	38
H16C	1509.11	5539.01	9087.71	38
H10	4493.3	6692.47	9710.75	28
H17A	3564.19	2419.58	8824.25	39
H17B	2677.52	2514.87	9126.12	39
H17C	3522.35	3806.01	9790.85	39
H8	4912.09	11024.29	7673.92	31
H9	5351.31	9376.64	9266.65	31
H13A	55.89	5344.81	6527.77	30
H13B	764.01	3766.31	6274.61	30
H7	3597.39	9941.75	6469.26	28
H4	1780.28	9629.83	6163.87	26
H14A	940.36	6324.69	5161.49	32
H14B	340.7	8033.04	5538.83	32
H18A	3129.5	4442.15	6062.76	40
H18B	2748.21	6490.16	5349.73	40
H18C	2110.93	4558.02	5451.77	40

Citations

CrysAlisPro (Rigaku, V1.171.42.60a, 2022)

CrysAlisPro (ROD), Rigaku Oxford Diffraction, Poland (?).

O.V. Dolomanov and L.J. Bourhis and R.J. Gildea and J.A.K. Howard and H. Puschmann, Olex2: A complete structure solution, refinement and analysis program, *J. Appl. Cryst.*, (2009), **42**, 339-341.

Sheldrick, G.M., Crystal structure refinement with ShelXL, *Acta Cryst.*, (2015), **C71**, 3-8.

Sheldrick, G.M., ShelXT-Integrated space-group and crystal-structure determination, *Acta Cryst.*, (2015), **A71**, 3-8.

The Rigaku Synergy S Diffractometer was purchased with Support from the MRI program by the National Science Foundation under Grant No. 1919565.

Compound 15b

Submitted by: **Jimmy Wu**

Date Collected: **6-14-2022**

Solved by: **Richard J Staples**

Sample ID: **ZTP-3-187**

Center Crystallographic for Research
Michigan State University
Department of Chemistry
East Lansing, MI 48824
Dr. Richard J. Staples
staples@chemistry.msu.edu

Crystal structure from crystals provided.

Crystal Data and Experimental

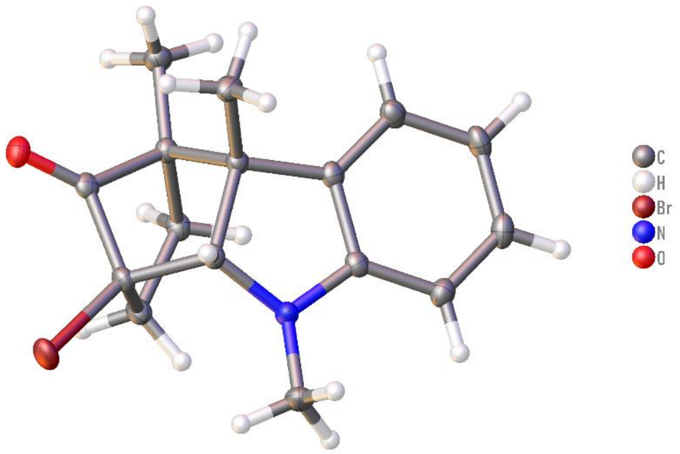

**Compound**	**JW622A**
Formula	$C_{16}H_{18}BrNO$
$D_{calc.}$/ g cm^{-3}	1.520
μ/mm^{-1}	3.924
Formula Weight	320.22
Color	colourless
Shape	irregular-shaped
Size/mm^3	0.18×0.15×0.12
T/K	100.01(10)
Crystal System	monoclinic
Space Group	$P2_1/n$
a/Å	8.88043(12)
b/Å	10.96832(16)
c/Å	14.65499(19)
α/°	90
β/°	101.3867(13)
γ/°	90
V/Å^3	1399.35(3)
Z	4
Z'	1
Wavelength/Å	1.54184
Radiation type	Cu K_α
Θ_{min}/°	5.073
Θ_{max}/°	76.789
Measured Refl's.	17600
Indep't Refl's	2868
Refl's I≥2 σ(I)	2750
R_{int}	0.0331
Parameters	175
Restraints	0
Largest Peak	0.298
Deepest Hole	-0.415
GooF	1.062
wR_2 (all data)	0.0580
wR_2	0.0575
R_1 (all data)	0.0236
R_1	0.0227

Experimental. Single colourless irregular-shaped crystals of **JW622A** used as received. A suitable crystal with dimensions 0.18 × 0.15 × 0.12 mm^3 was selected and mounted on a nylon loop with paratone oil on a XtaLAB Synergy, Dualflex, HyPix diffractometer. The crystal was kept at a steady T = 100.01(10) K during data collection. The structure was solved with the **ShelXS** (Sheldrick, 2008) solution program using direct methods and by using **Olex2** 1.5 (Dolomanov et al., 2009) as the graphical interface. The model was refined with **ShelXL** 2018/3 (Sheldrick, 2015) using full matrix least squares minimisation on $\boldsymbol{F^2}$.

Crystal Data. $C_{16}H_{18}BrNO$, M_r = 320.22, monoclinic, $P2_1/n$ (No. 14), a = 8.88043(12) Å, b = 10.96832(16) Å, c = 14.65499(19) Å, β = 101.3867(13)°, α = γ = 90°, V = 1399.35(3) Å^3, T = 100.01(10) K, Z = 4, Z' = 1, μ(Cu K_α) = 3.924, 17600 reflections measured, 2868 unique (R_{int} = 0.0331) which were used in all calculations. The final wR_2 was 0.0580 (all data) and R_1 was 0.0227 (I≥2 σ(I)).

The Rigaku Synergy S Diffractometer was purchased with Support from the MRI program by the National Science Foundation under Grant No. 1919565.

Structure Quality Indicators

**Reflections:**	d min (Cu\a) 2Θ=154.2° 0.79	I/σ(I) 51.1	Rint 3.31%	Full 135.4° 97% to 154.2° 100
**Refinement:**	Shift -0.001	Max Peak 0.3	Min Peak -0.4	GooF 1.062

A colourless irregular-shaped-shaped crystal with dimensions 0.18×0.15×0.12 mm^3 was mounted on a nylon loop with paratone oil. Data were collected using a XtaLAB Synergy, Dualflex, HyPix diffractometer equipped with an Oxford Cryosystems low-temperature device, operating at T = 100.01(10) K.

MSU Data were measured using ω scans using Cu K_α radiation (micro-focus sealed X-ray tube, 50 kV, 1 mA). The total number of runs and images was based on the strategy calculation from the program CrysAlisPro 1.171.42.58a (Rigaku OD, 2022). The achieved resolution was Θ = 76.789.

Cell parameters were retrieved using the CrysAlisPro 1.171.42.58a (Rigaku OD, 2022) software and refined using CrysAlisPro 1.171.42.58a (Rigaku OD, 2022) on 12002 reflections, 68 % of the observed reflections. Data reduction was performed using the CrysAlisPro 1.171.42.58a (Rigaku OD, 2022) software which corrects for Lorentz polarization. The final completeness is 100.00 out to 76.789 in Θ CrysAlisPro 1.171.42.58a (Rigaku Oxford Diffraction, 2022) Empirical absorption correction using spherical harmonics, implemented in SCALE3 ABSPACK scaling algorithm.

The structure was solved in the space group $P2_1/n$ (# 14) by using direct methods using the ShelXS (Sheldrick, 2008) structure solution program. The structure was refined by Least Squares ShelXL incorporated in Olex2 software program. All non-hydrogen atoms were refined anisotropically. Hydrogen atom positions were calculated geometrically and refined using the riding model, except for the hydrogen atom on the non-carbon atom(s) which were found by difference Fourier methods and refined isotropically when data permits.

There is a single molecule in the asymmetric unit, which is represented by the reported sum formula. In other words: Z is 4 and Z' is 1.

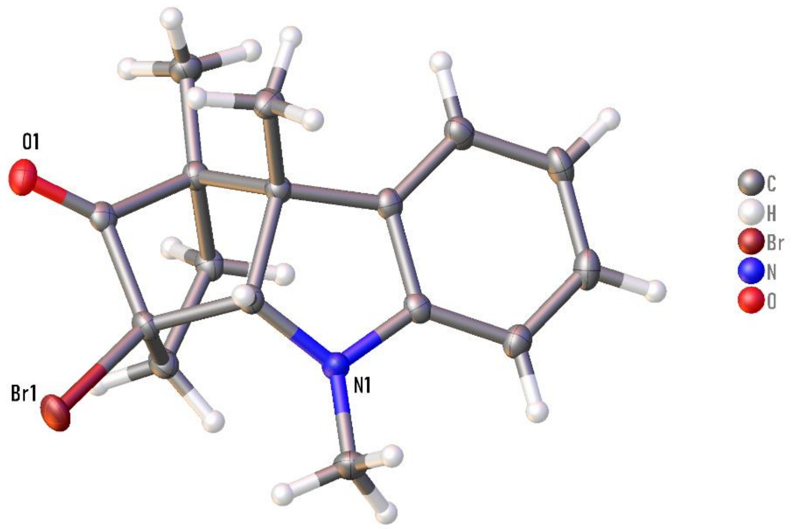

Figure 6:

The Rigaku Synergy S Diffractometer was purchased with Support from the MRI program by the National Science Foundation under Grant No. 1919565.

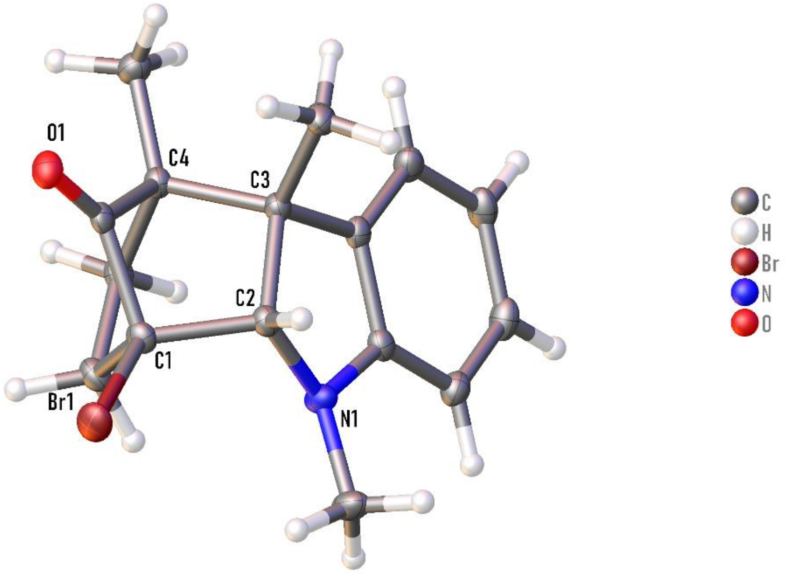

Figure 7: Model has Chirality at C1 (Centro SPGR) R Verify, Model has Chirality at C2 (Centro SPGR) S Verify, Model has Chirality at C3 (Centro SPGR) S Verify, Model has Chirality at C4 (Centro SPGR) R Verify. Due to Centrosymmetric space group the other diastereomer is present in the crystal lattice.

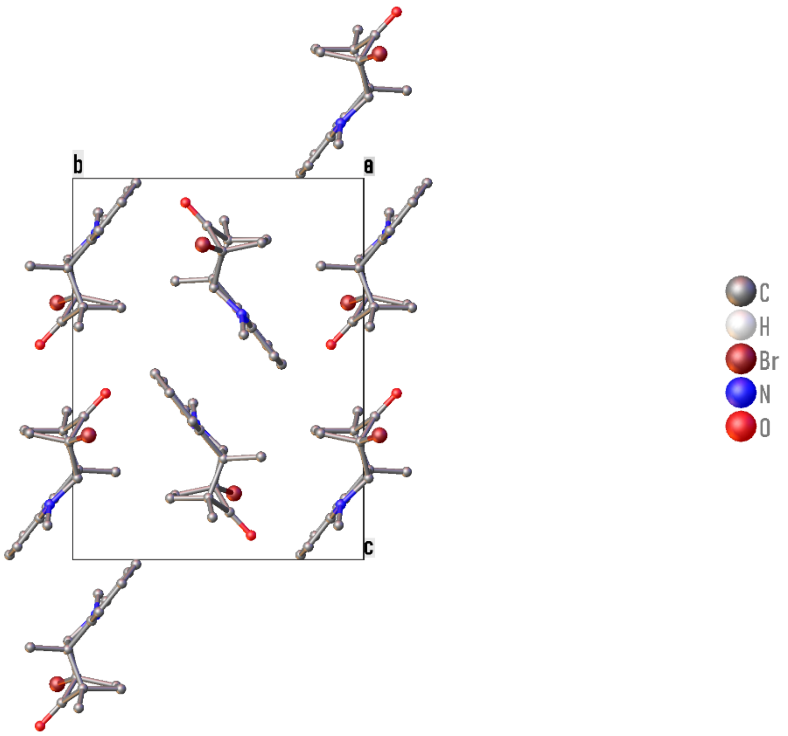

Figure 8: Packing diagram of JW622A.

The Rigaku Synergy S Diffractometer was purchased with Support from the MRI program by the National Science Foundation under Grant No. 1919565.

Data Plots: Diffraction Data

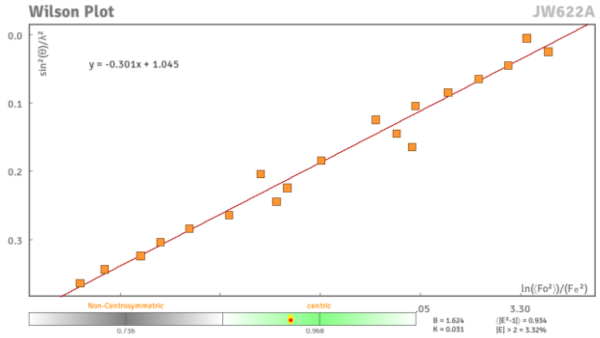

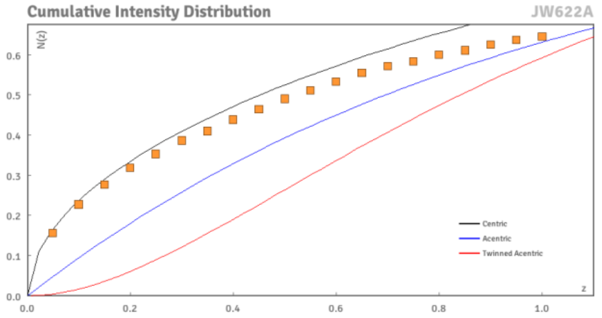

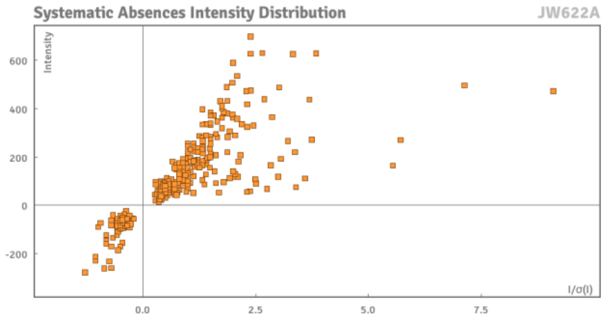

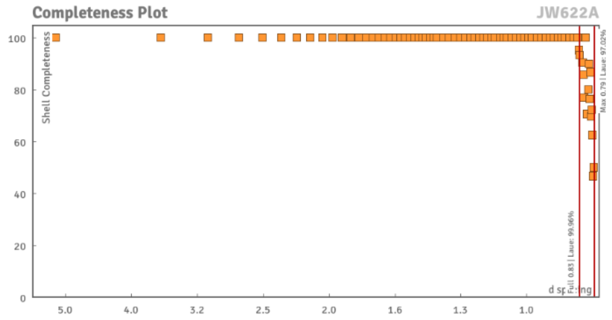

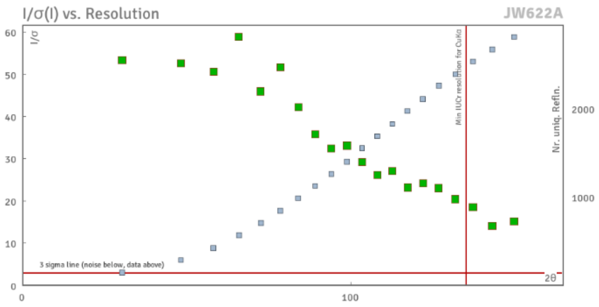

Data Plots: Refinement and Data

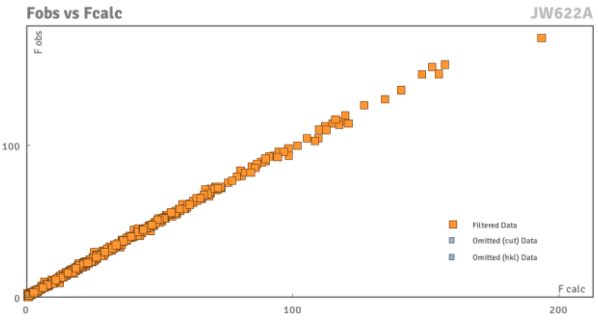

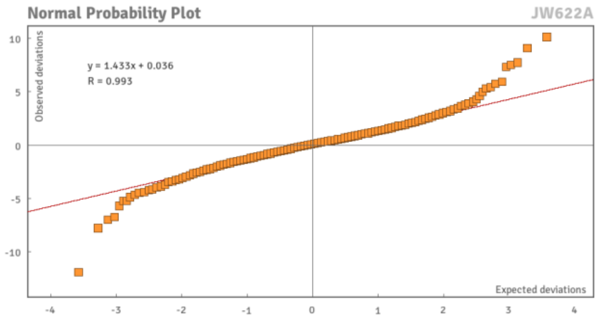

Reflection Statistics

Total reflections (after filtering)	18221	Unique reflections	2868
Completeness	0.97	Mean I/σ	34.49
hkl_{max} collected	(10, 13, 18)	hkl_{min} collected	(-11, -13, -14)
hkl_{max} used	(10, 13, 18)	hkl_{min} used	(-11, 0, 0)
Lim d_{max} collected	100.0	Lim d_{min} collected	0.77

The Rigaku Synergy S Diffractometer was purchased with Support from the MRI program by the National Science Foundation under Grant No. 1919565.

d_{max} used	14.37	d_{min} used	0.79
Friedel pairs	1260	Friedel pairs merged	1
Inconsistent equivalents	0	R_{int}	0.0331
R_{sigma}	0.0196	Intensity transformed	0
Omitted reflections	0	Omitted by user (OMIT hkl)	0
Multiplicity	(2451, 1568, 977, 714, 412, 232, 157, 141, 84, 29, 10, 1)	Maximum multiplicity	22
Removed systematic absences	621	Filtered off (Shel/OMIT)	0

Selected Crystal Pictures

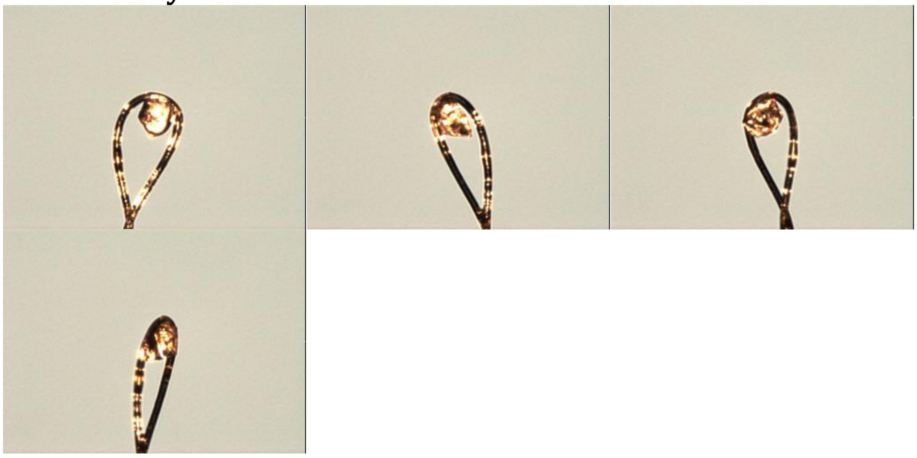

Table 13: Fractional Atomic Coordinates (×10[4]) and Equivalent Isotropic Displacement Parameters (Å[2]×10[3]) for **JW622A.** U_{eq} is defined as 1/3 of the trace of the orthogonalised U_{ij}.

Atom	x	y	z	U_{eq}
Br1	1338.0(2)	4461.5(2)	8263.1(2)	22.37(7)
O1	4788.4(13)	3873.7(10)	9362.9(7)	19.6(2)
N1	3274.3(15)	5797.4(13)	6442.0(9)	17.7(3)
C1	3273.6(17)	5129.5(14)	8091.6(10)	16.0(3)
C2	3692.1(17)	4846.0(14)	7134.5(10)	15.2(3)
C3	5508.9(17)	4824.9(14)	7355.1(10)	14.5(3)
C4	5913.1(18)	5338.9(14)	8377.8(10)	14.8(3)
C5	5255.2(18)	6649.8(14)	8392.4(11)	17.0(3)
C6	3487.8(18)	6487.2(14)	8311.7(11)	18.4(3)
C7	4691.2(18)	4617.4(14)	8755.1(10)	15.0(3)
C8	5896.9(18)	5643.1(14)	6603.8(10)	15.2(3)
C9	4541.7(17)	6142.5(14)	6086.9(10)	15.6(3)
C10	4581.3(18)	6911.2(15)	5337.5(10)	18.6(3)
C11	6006.2(19)	7173.8(15)	5123.4(11)	20.9(3)
C12	7349.8(19)	6677.4(16)	5624.5(11)	21.4(3)
C13	7298.2(18)	5892.3(16)	6372.6(11)	19.4(3)
C14	6135.8(19)	3532.4(15)	7301.2(11)	19.2(3)
C15	7557.9(18)	5203.0(16)	8888.2(11)	20.3(3)
C16	1722.5(18)	5874.1(17)	5902.0(12)	23.0(3)

Table 14: Anisotropic Displacement Parameters (×10[4]) for **JW622A.** The anisotropic displacement factor exponent takes the form: $-2\pi^2[h^2a^{*2} \times U_{11} + \ldots + 2hka^* \times b^* \times U_{12}]$

Atom	U_{11}	U_{22}	U_{33}	U_{23}	U_{13}	U_{12}
Br1	16.94(10)	30.12(12)	21.98(11)	1.25(6)	8.53(7)	-5.43(6)

The Rigaku Synergy S Diffractometer was purchased with Support from the MRI program by the National Science Foundation under Grant No. 1919565.

Atom	U_{11}	U_{22}	U_{33}	U_{23}	U_{13}	U_{12}
O1	25.9(6)	17.6(6)	16.2(5)	1.4(4)	6.4(4)	0.1(4)
N1	13.8(6)	23.3(7)	16.3(6)	4.9(5)	3.6(5)	1.5(5)
C1	14.1(7)	17.9(7)	17.2(7)	0.4(6)	6.4(6)	-1.5(6)
C2	15.9(7)	15.5(7)	14.8(7)	-0.1(6)	4.9(5)	-0.2(6)
C3	14.4(7)	16.1(7)	13.3(7)	-1.0(5)	3.4(5)	0.3(6)
C4	15.9(7)	15.6(7)	13.5(7)	-0.8(5)	4.3(6)	-0.4(6)
C5	20.2(7)	15.0(7)	16.8(7)	-1.8(6)	6.1(6)	-1.8(6)
C6	19.6(8)	17.4(8)	20.0(7)	-0.5(6)	7.9(6)	1.8(6)
C7	18.3(7)	14.4(7)	13.2(7)	-3.9(5)	4.9(6)	0.8(6)
C8	17.6(7)	16.2(7)	12.5(7)	-2.5(5)	4.2(6)	-1.5(6)
C9	17.1(7)	15.9(7)	14.9(7)	-2.7(5)	5.4(6)	-0.7(6)
C10	22.3(8)	19.1(8)	14.2(7)	0.4(6)	3.3(6)	0.3(6)
C11	28.0(8)	20.6(8)	15.2(7)	-0.1(6)	6.7(6)	-6.4(7)
C12	21.3(8)	26.6(9)	18.4(7)	-2.8(6)	9.1(6)	-7.6(6)
C13	17.2(7)	25.2(8)	16.1(7)	-2.4(6)	3.8(6)	-2.2(6)
C14	22.5(8)	18.8(8)	16.6(7)	-0.9(6)	4.5(6)	4.9(6)
C15	17.0(8)	25.1(8)	18.1(7)	0.3(6)	1.7(6)	0.4(6)
C16	14.8(7)	31.7(9)	22.0(8)	4.9(7)	2.2(6)	3.6(7)

Table 15: Bond Lengths in Å for **JW622A**.

Atom	Atom	Length/Å
Br1	C1	1.9301(15)
O1	C7	1.1983(19)
N1	C2	1.452(2)
N1	C9	1.383(2)
N1	C16	1.450(2)
C1	C2	1.552(2)
C1	C6	1.528(2)
C1	C7	1.537(2)
C2	C3	1.582(2)
C3	C4	1.575(2)
C3	C8	1.512(2)

Atom	Atom	Length/Å
C3	C14	1.531(2)
C4	C5	1.554(2)
C4	C7	1.532(2)
C4	C15	1.511(2)
C5	C6	1.561(2)
C8	C9	1.401(2)
C8	C13	1.380(2)
C9	C10	1.390(2)
C10	C11	1.393(2)
C11	C12	1.383(2)
C12	C13	1.402(2)

Table 16: Bond Angles in ° for **JW622A**.

Atom	Atom	Atom	Angle/°
C9	N1	C2	110.19(12)
C9	N1	C16	122.36(13)
C16	N1	C2	120.49(13)
C2	C1	Br1	114.83(10)
C6	C1	Br1	114.81(10)
C6	C1	C2	110.13(12)
C6	C1	C7	99.81(12)
C7	C1	Br1	114.72(10)
C7	C1	C2	100.73(12)
N1	C2	C1	114.46(13)
N1	C2	C3	105.25(12)
C1	C2	C3	103.58(12)
C4	C3	C2	102.76(11)
C8	C3	C2	102.26(12)
C8	C3	C4	116.01(12)

Atom	Atom	Atom	Angle/°
C8	C3	C14	112.00(12)
C14	C3	C2	111.70(13)
C14	C3	C4	111.33(12)
C5	C4	C3	109.19(12)
C7	C4	C3	96.54(11)
C7	C4	C5	100.25(12)
C15	C4	C3	116.88(13)
C15	C4	C5	114.45(13)
C15	C4	C7	117.04(13)
C4	C5	C6	105.58(12)
C1	C6	C5	102.05(12)
O1	C7	C1	130.45(14)
O1	C7	C4	131.90(15)
C4	C7	C1	97.64(12)
C9	C8	C3	109.33(13)

The Rigaku Synergy S Diffractometer was purchased with Support from the MRI program by the National Science Foundation under Grant No. 1919565.

Atom	Atom	Atom	Angle/˚
C13	C8	C3	129.98(14)
C13	C8	C9	120.65(14)
N1	C9	C8	111.44(13)
N1	C9	C10	127.78(14)
C10	C9	C8	120.75(14)
C9	C10	C11	117.98(15)
C12	C11	C10	121.73(15)
C11	C12	C13	119.93(15)
C8	C13	C12	118.94(15)

Table 17: Torsion Angles in ˚ for **JW622A**.

Atom	Atom	Atom	Atom	Angle/˚
Br1	C1	C2	N1	95.47(14)
Br1	C1	C2	C3	-150.51(10)
Br1	C1	C6	C5	164.16(10)
Br1	C1	C7	O1	-0.2(2)
Br1	C1	C7	C4	178.75(10)
N1	C2	C3	C4	110.44(13)
N1	C2	C3	C8	-10.19(15)
N1	C2	C3	C14	-130.12(13)
N1	C9	C10	C11	177.41(15)
C1	C2	C3	C4	-10.05(15)
C1	C2	C3	C8	-130.67(12)
C1	C2	C3	C14	109.39(13)
C2	N1	C9	C8	-10.48(18)
C2	N1	C9	C10	171.49(15)
C2	C1	C6	C5	-64.37(15)
C2	C1	C7	O1	-124.15(17)
C2	C1	C7	C4	54.85(13)
C2	C3	C4	C5	-60.43(14)
C2	C3	C4	C7	42.84(13)
C2	C3	C4	C15	167.68(13)
C2	C3	C8	C9	4.54(15)
C2	C3	C8	C13	-173.02(16)
C3	C4	C5	C6	74.83(14)
C3	C4	C7	O1	118.87(17)
C3	C4	C7	C1	-60.11(12)
C3	C8	C9	N1	3.20(18)
C3	C8	C9	C10	-178.62(14)
C3	C8	C13	C12	178.80(15)
C4	C3	C8	C9	-106.41(15)
C4	C3	C8	C13	76.0(2)
C4	C5	C6	C1	-9.33(15)
C5	C4	C7	O1	-130.22(17)
C5	C4	C7	C1	50.80(13)
C6	C1	C2	N1	-35.99(17)
C6	C1	C2	C3	78.04(15)
C6	C1	C7	O1	123.00(17)
C6	C1	C7	C4	-58.00(13)
C7	C1	C2	N1	-140.71(13)
C7	C1	C2	C3	-26.68(14)
C7	C1	C6	C5	40.98(14)
C7	C4	C5	C6	-25.87(14)
C8	C3	C4	C5	50.23(17)
C8	C3	C4	C7	153.50(13)
C8	C3	C4	C15	-81.66(17)
C8	C9	C10	C11	-0.5(2)
C9	N1	C2	C1	125.85(14)
C9	N1	C2	C3	12.81(16)

The Rigaku Synergy S Diffractometer was purchased with Support from the MRI program by the National Science Foundation under Grant No. 1919565.

Atom	Atom	Atom	Atom	Angle/°
C9	C8	C13	C12	1.5(2)
C9	C10	C11	C12	1.0(2)
C10	C11	C12	C13	-0.3(2)
C11	C12	C13	C8	-0.9(2)
C13	C8	C9	N1	-178.98(14)
C13	C8	C9	C10	-0.8(2)
C14	C3	C4	C5	179.87(12)
C14	C3	C4	C7	-76.85(14)
C14	C3	C4	C15	47.98(18)
C14	C3	C8	C9	124.27(14)
C14	C3	C8	C13	-53.3(2)
C15	C4	C5	C6	-152.01(13)
C15	C4	C7	O1	-5.9(2)
C15	C4	C7	C1	175.17(13)
C16	N1	C2	C1	-82.69(18)
C16	N1	C2	C3	164.27(13)
C16	N1	C9	C8	-161.31(15)
C16	N1	C9	C10	20.7(3)

Table 18: Hydrogen Fractional Atomic Coordinates (×10[4]) and Equivalent Isotropic Displacement Parameters (Å^2×10^3) for **JW622A**. U_{eq} is defined as 1/3 of the trace of the orthogonalised U_{ij}.

Atom	x	y	z	U_{eq}
H2	3265.61	4039.74	6890.41	18
H5A	5474.44	7133.24	7863.21	20
H5B	5711.85	7070.04	8979.73	20
H6A	3169.37	6695.08	8903.17	22
H6B	2901.97	6994.47	7804.46	22
H10	3664.19	7247.14	4982.43	22
H11	6056.72	7707.77	4619.92	25
H12	8306.55	6868.29	5461.8	26
H13	8213.3	5537.6	6715	23
H14A	5725.38	3186.26	6685.86	29
H14B	5823.81	3023.39	7781.24	29
H14C	7259.52	3560.22	7401.94	29
H15A	7651.6	5493.41	9529.36	30
H15B	8231.34	5684.84	8572.39	30
H15C	7857.4	4342.69	8893.84	30
H16A	1495.21	6719.68	5706.25	35
H16B	995.45	5602.96	6283.49	35
H16C	1627.91	5351.4	5351.25	35

Citations

CrysAlisPro (Rigaku, V1.171.42.58a, 2022)

CrysAlisPro (ROD), Rigaku Oxford Diffraction, Poland (?).

O.V. Dolomanov and L.J. Bourhis and R.J. Gildea and J.A.K. Howard and H. Puschmann, Olex2: A complete structure solution, refinement and analysis program, *J. Appl. Cryst.*, (2009), **42**, 339-341.

Sheldrick, G.M., A short history of ShelX, *Acta Cryst.*, (2008), **A64**, 339-341.

Sheldrick, G.M., Crystal structure refinement with ShelXL, *Acta Cryst.*, (2015), **C71**, 3-8.

The Rigaku Synergy S Diffractometer was purchased with Support from the MRI program by the National Science Foundation under Grant No. 1919565.

Compound 16b

Submitted by: **Jimmy Wu**

Date Collected: **June 28, 2022**

Solved by: **Richard J Staples**

Sample ID: **ZTP-3-187-minor**

Center Crystallographic for Research
Michigan State University
Department of Chemistry
East Lansing, MI 48824
Dr. Richard J. Staples
staples@chemistry.msu.edu

Crystal structure from crystals provided shows the relative stereochemistry.

Crystal Data and Experimental

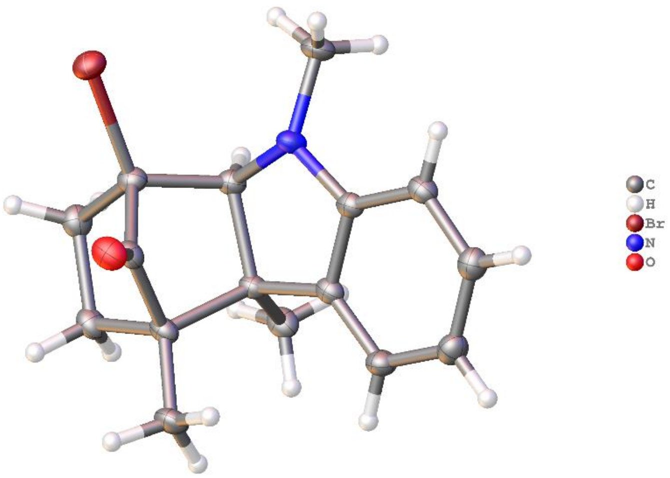

**Compound**	**JW622B**
Formula	$C_{16}H_{18}BrNO$
$D_{calc.}$/ g cm^{-3}	1.539
μ/mm^{-1}	3.972
Formula Weight	320.22
Color	colourless
Shape	block-shaped
Size/mm^3	0.35×0.20×0.17
T/K	100.00(10)
Crystal System	monoclinic
Space Group	$P2_1/c$
a/Å	13.68696(11)
b/Å	6.65657(6)
c/Å	15.48510(13)
α/°	90
β/°	101.5354(8)
γ/°	90
V/Å^3	1382.32(2)
Z	4
Z'	1
Wavelength/Å	1.54184
Radiation type	Cu K$_\alpha$
Θ_{min}/°	3.296
Θ_{max}/°	80.122
Measured Refl's.	19284
Indep't Refl's	2959
Refl's I≥2 σ(I)	2910
R_{int}	0.0267
Parameters	175
Restraints	0
Largest Peak	0.556
Deepest Hole	-0.620
GooF	1.126
wR_2 (all data)	0.0719
wR_2	0.0715
R_1 (all data)	0.0283
R_1	0.0278

Experimental. Single colourless block-shaped crystals of **JW622B** used as received. A suitable crystal with dimensions 0.35 × 0.20 × 0.17 mm^3 was selected and mounted on a nylon loop with paratone oil on a XtaLAB Synergy, Dualflex, HyPix diffractometer. The crystal was kept at a steady T = 100.00(10) K during data collection. The structure was solved with the **ShelXT** (Sheldrick, 2015) solution program using dual methods and by using **Olex2** 1.5 (Dolomanov et al., 2009) as the graphical interface. The model was refined with **ShelXL** 2018/3 (Sheldrick, 2015) using full matrix least squares minimisation on F^2.

Crystal Data. $C_{16}H_{18}BrNO$, M_r = 320.22, monoclinic, $P2_1/c$ (No. 14), a = 13.68696(11) Å, b = 6.65657(6) Å, c = 15.48510(13) Å, β = 101.5354(8)°, α = γ = 90°, V = 1382.32(2) Å^3, T = 100.00(10) K, Z = 4, Z' = 1, μ(Cu K$_\alpha$) = 3.972, 19284 reflections measured, 2959 unique (R_{int} = 0.0267) which were used in all calculations. The final wR_2 was 0.0719 (all data) and R_1 was 0.0278 (I≥2 σ(I)).

The Rigaku Synergy S Diffractometer was purchased with Support from the MRI program by the National Science Foundation under Grant No. 1919565.

Structure Quality Indicators

**Reflections:**	d min (Cu\a) 2Θ=160.2° 0.78	I/σ(I) 68.3	Rint 2.67%	Full 135.4° 98% to 160.2° 100
**Refinement:**	Shift 0.002	Max Peak 0.6	Min Peak -0.6	GooF 1.126

A colourless block-shaped-shaped crystal with dimensions 0.35×0.20×0.17 mm^3 was mounted on a nylon loop with paratone oil. Data were collected using a XtaLAB Synergy, Dualflex, HyPix diffractometer equipped with an Oxford Cryosystems low-temperature device, operating at T = 100.00(10) K.

MSU Data were measured using ω scans using Cu K_α radiation (micro-focus sealed X-ray tube, 50 kV, 1 mA). The total number of runs and images was based on the strategy calculation from the program CrysAlisPro 1.171.42.59a (Rigaku OD, 2022). The achieved resolution was Θ = 80.122.

Cell parameters were retrieved using the CrysAlisPro 1.171.42.59a (Rigaku OD, 2022) software and refined using CrysAlisPro 1.171.42.59a (Rigaku OD, 2022) on 14864 reflections, 77 % of the observed reflections. Data reduction was performed using the CrysAlisPro 1.171.42.59a (Rigaku OD, 2022) software which corrects for Lorentz polarization. The final completeness is 100.00 out to 80.122 in Θ CrysAlisPro 1.171.42.59a (Rigaku Oxford Diffraction, 2022) Numerical absorption correction based on gaussian integration over a multifaceted crystal model Empirical absorption correction using spherical harmonics, implemented in SCALE3 ABSPACK scaling algorithm.

The structure was solved in the space group $P2_1/c$ (# 14) by using dual methods using the ShelXT (Sheldrick, 2015) structure solution program. The structure was refined by Least Squares ShelXL incorporated in Olex2 software program. All non-hydrogen atoms were refined anisotropically. Hydrogen atom positions were calculated geometrically and refined using the riding model, except for the hydrogen atom on the non-carbon atom(s) which were found by difference Fourier methods and refined isotropically when data permits.

There is a single molecule in the asymmetric unit, which is represented by the reported sum formula. In other words: Z is 4 and Z' is 1.

The Rigaku Synergy S Diffractometer was purchased with Support from the MRI program by the National Science Foundation under Grant No. 1919565.

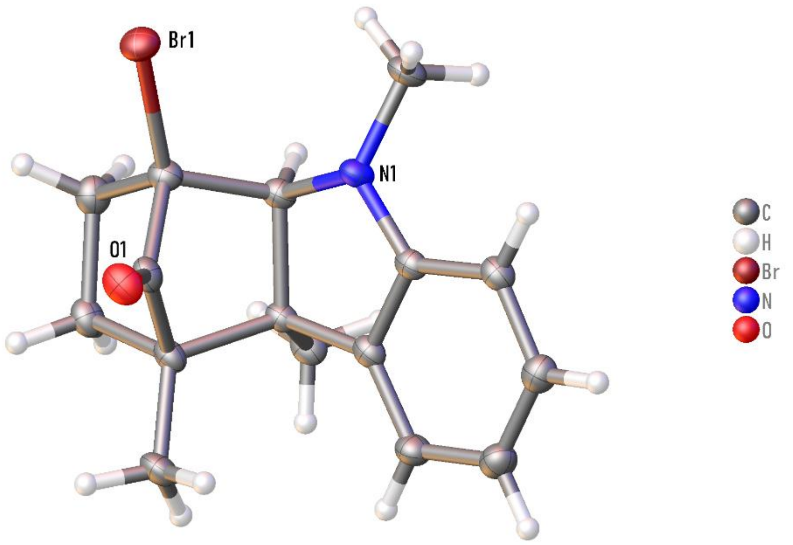

Figure 9: drawing at 50% ellipsoid showing heter-atom labeling scheme.

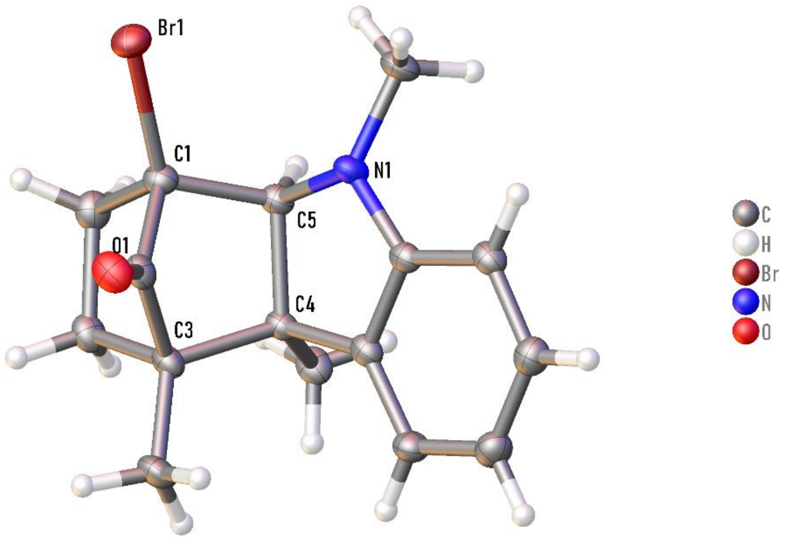

Figure 10: Drawing showing relative stereo chemistry, This enantiomer shown has all chiral centers as S.

The Rigaku Synergy S Diffractometer was purchased with Support from the MRI program by the National Science Foundation under Grant No. 1919565.

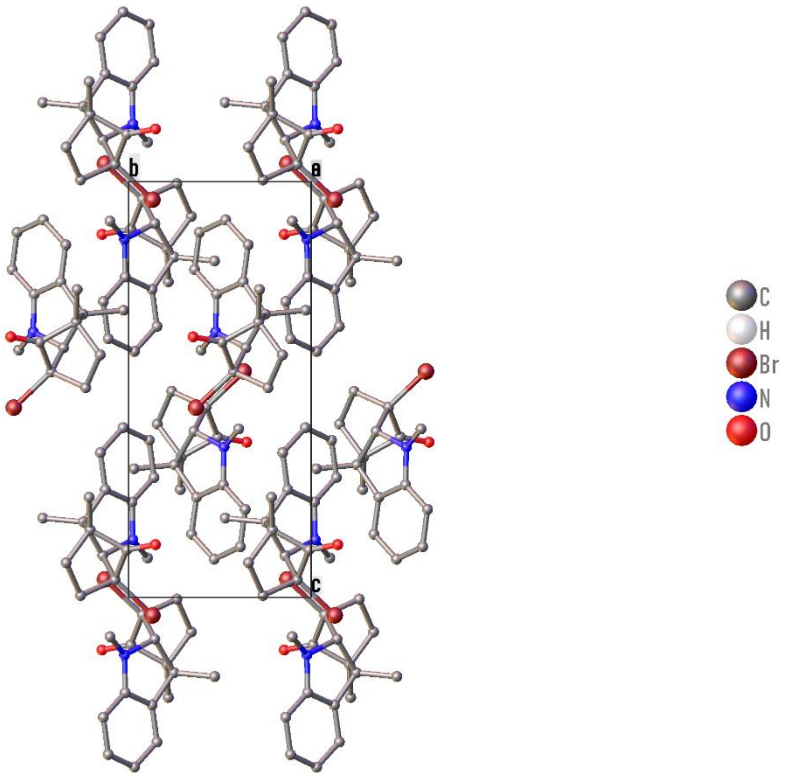

Figure 11: Packing diagram of JW622B.

Data Plots: Diffraction Data

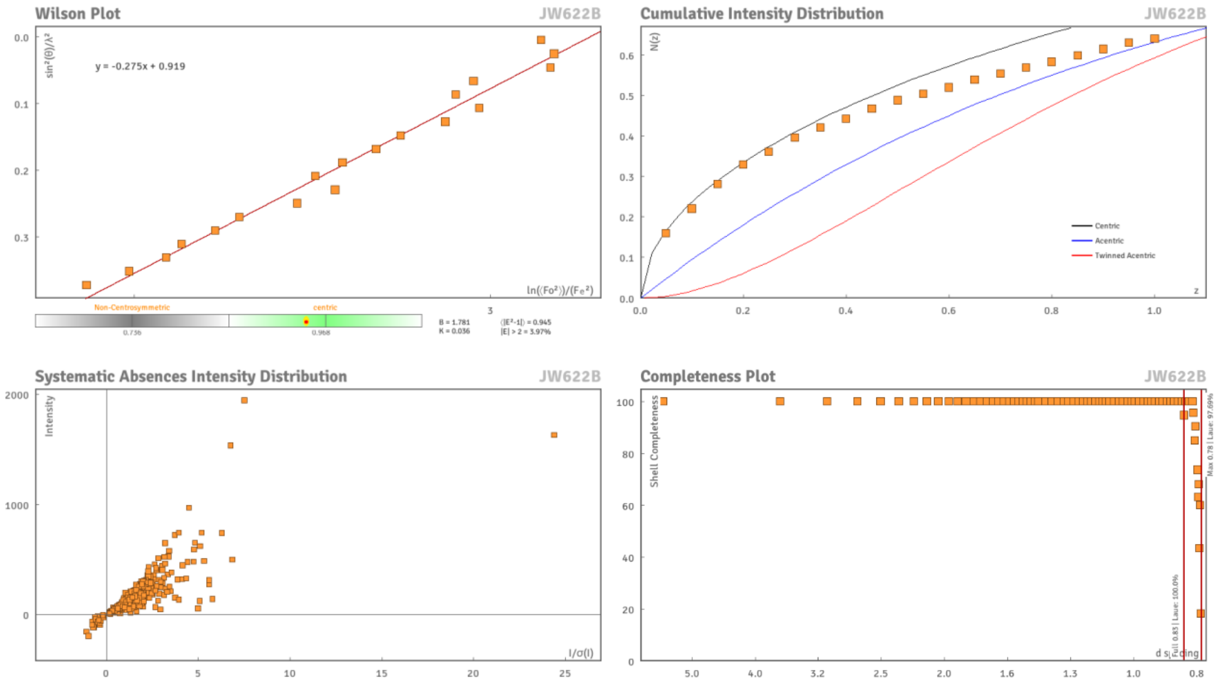

The Rigaku Synergy S Diffractometer was purchased with Support from the MRI program by the National Science Foundation under Grant No. 1919565.

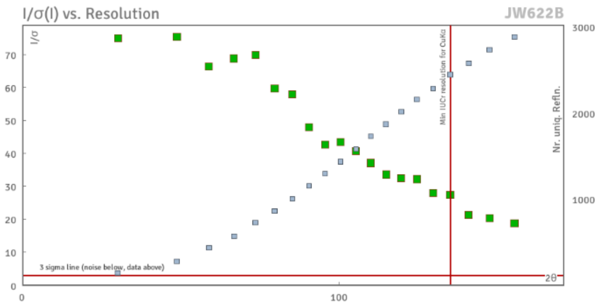

Data Plots: Refinement and Data

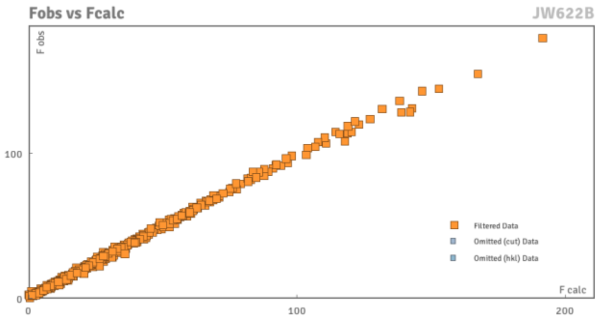

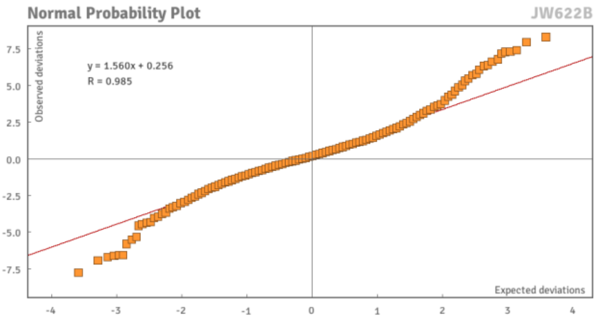

Reflection Statistics

Total reflections (after filtering)	20452	Unique reflections	2959
Completeness	0.977	Mean I/σ	45.42
hkl_{max} collected	(17, 8, 19)	hkl_{min} collected	(-17, -5, -19)
hkl_{max} used	(17, 8, 19)	hkl_{min} used	(-17, 0, 0)
Lim d_{max} collected	100.0	Lim d_{min} collected	0.77
d_{max} used	15.17	d_{min} used	0.78
Friedel pairs	2917	Friedel pairs merged	1
Inconsistent equivalents	3	R_{int}	0.0267
R_{sigma}	0.0146	Intensity transformed	0
Omitted reflections	0	Omitted by user (OMIT hkl)	0
Multiplicity	(2908, 2200, 1459, 850, 490, 267, 134, 37, 9)	Maximum multiplicity	21
Removed systematic absences	1168	Filtered off (Shel/OMIT)	0

Selected Crystal Pictures

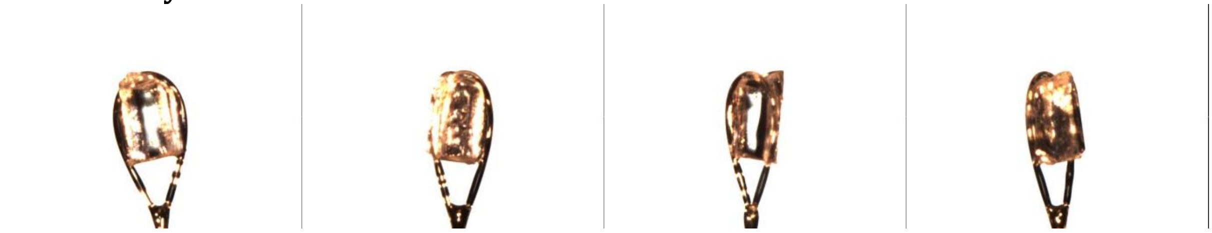

Table 19: Fractional Atomic Coordinates (×10[4]) and Equivalent Isotropic Displacement Parameters (Å^2×10^3) for **JW622B**. U_{eq} is defined as 1/3 of the trace of the orthogonalised U_{ij}.

Atom	x	y	z	U_{eq}
Br1	8020.1(2)	6302.6(3)	5434.7(2)	23.90(8)

The Rigaku Synergy S Diffractometer was purchased with Support from the MRI program by the National Science Foundation under Grant No. 1919565.

Atom	x	y	z	U_{eq}
O1	9066.7(10)	6468(2)	3732.6(10)	24.4(3)
N1	6495.8(11)	5230(2)	3616.3(10)	18.9(3)
C1	8132.6(14)	4247(3)	4576.9(12)	18.5(3)
C2	8700.0(13)	4883(3)	3860.0(12)	18.3(4)
C3	8654.8(13)	2884(3)	3375.8(12)	18.3(4)
C4	7495.5(13)	2515(3)	3187.0(12)	16.4(3)
C5	7139.9(13)	3572(3)	3995.1(12)	17.2(4)
C6	8711.3(14)	2357(3)	4956.7(13)	22.4(4)
C7	9152.3(14)	1569(3)	4167.5(13)	22.0(4)
C8	6344.9(12)	5184(3)	2706.3(12)	16.6(3)
C9	6931.2(13)	3717(3)	2410.1(13)	17.3(4)
C10	6882.0(14)	3449(3)	1518.7(13)	21.5(4)
C11	6255.7(15)	4689(3)	918.2(13)	24.2(4)
C12	5674.5(15)	6123(3)	1219.0(13)	22.9(4)
C13	5693.7(14)	6390(3)	2116.3(13)	19.2(4)
C14	9165.0(14)	2828(3)	2592.7(14)	24.0(4)
C15	7203.7(14)	291(3)	3093.4(13)	21.7(4)
C16	5730.4(14)	6029(3)	4052.6(13)	22.7(4)

Table 20: Anisotropic Displacement Parameters (×10[4]) for **JW622B**. The anisotropic displacement factor exponent takes the form: $-2\pi^2[h^2a^{*2} \times U_{11} + \ldots + 2hka^* \times b^* \times U_{12}]$

Atom	U_{11}	U_{22}	U_{33}	U_{23}	U_{13}	U_{12}
Br1	28.53(13)	20.30(12)	23.42(12)	-5.26(7)	6.50(8)	-2.52(7)
O1	23.9(7)	15.5(7)	35.2(8)	1.0(6)	9.2(6)	-4.0(5)
N1	19.0(7)	19.1(8)	20.4(7)	-0.4(6)	8.0(6)	3.3(6)
C1	20.9(8)	14.1(8)	21.5(8)	-2.5(7)	6.3(7)	-1.9(7)
C2	14.4(8)	16.1(9)	24.2(9)	0.8(7)	3.1(6)	0.7(7)
C3	15.2(8)	14.4(8)	26.1(9)	-0.9(7)	6.5(7)	-0.6(7)
C4	16.3(8)	12.8(8)	21.5(8)	-0.7(7)	7.1(6)	-0.6(6)
C5	18.3(8)	13.8(8)	21.0(9)	-0.2(7)	7.3(7)	-1.6(6)
C6	23.1(9)	17.9(9)	25.3(9)	2.9(7)	3.0(7)	-0.4(7)
C7	19.1(8)	16.6(9)	29.7(10)	1.2(8)	3.8(7)	1.6(7)
C8	15.5(8)	13.6(8)	21.6(8)	-2.1(7)	6.2(6)	-3.1(6)
C9	15.5(8)	13.6(8)	23.9(9)	-0.8(7)	6.1(7)	-1.9(6)
C10	21.7(9)	20.2(9)	23.5(9)	-3.7(7)	7.0(7)	0.5(7)
C11	25.6(9)	26.5(10)	20.8(9)	0.3(8)	5.2(7)	-2.8(8)
C12	23.5(9)	21.2(10)	23.5(9)	1.6(7)	3.4(7)	-3.6(7)
C13	18.1(8)	14.2(9)	26.0(9)	-0.9(7)	6.0(7)	-0.5(6)
C14	19.3(9)	23.8(10)	31.9(10)	-3.0(8)	12.1(7)	0.3(7)
C15	21.3(9)	13.2(9)	30.9(10)	-1.0(7)	6.0(7)	-1.9(7)
C16	22.0(9)	21.9(10)	27.3(10)	-3.0(8)	12.0(7)	2.0(7)

Table 21: Bond Lengths in Å for **JW622B**.

Atom	Atom	Length/Å
Br1	C1	1.9347(18)
O1	C2	1.201(2)
N1	C5	1.460(2)
N1	C8	1.383(2)
N1	C16	1.456(2)
C1	C2	1.536(2)

Atom	Atom	Length/Å
C1	C5	1.539(2)
C1	C6	1.539(3)
C2	C3	1.523(3)
C3	C4	1.574(2)
C3	C7	1.549(3)
C3	C14	1.516(3)

The Rigaku Synergy S Diffractometer was purchased with Support from the MRI program by the National Science Foundation under Grant No. 1919565.

Atom	Atom	Length/Å
C4	C5	1.594(2)
C4	C9	1.521(3)
C4	C15	1.532(3)
C6	C7	1.558(3)
C8	C9	1.398(2)
C8	C13	1.396(3)
C9	C10	1.380(3)
C10	C11	1.401(3)
C11	C12	1.382(3)
C12	C13	1.396(3)

Table 22: Bond Angles in ° for **JW622B**.

Atom	Atom	Atom	Angle/°
C8	N1	C5	110.36(14)
C8	N1	C16	120.40(15)
C16	N1	C5	121.50(15)
C2	C1	Br1	114.99(13)
C2	C1	C5	99.91(14)
C2	C1	C6	101.86(14)
C5	C1	Br1	115.21(12)
C6	C1	Br1	114.91(13)
C6	C1	C5	108.18(15)
O1	C2	C1	130.45(17)
O1	C2	C3	131.59(17)
C3	C2	C1	97.96(14)
C2	C3	C4	99.67(13)
C2	C3	C7	97.81(15)
C7	C3	C4	109.03(14)
C14	C3	C2	115.80(16)
C14	C3	C4	117.14(15)
C14	C3	C7	114.80(15)
C3	C4	C5	103.88(14)
C9	C4	C3	113.47(14)
C9	C4	C5	101.65(14)
C9	C4	C15	110.62(15)
C15	C4	C3	113.72(14)
C15	C4	C5	112.72(14)
N1	C5	C1	113.97(15)
N1	C5	C4	105.84(14)
C1	C5	C4	102.47(14)
C1	C6	C7	103.06(15)
C3	C7	C6	104.63(15)
N1	C8	C9	111.78(16)
N1	C8	C13	126.87(16)
C13	C8	C9	121.34(17)
C8	C9	C4	110.00(16)
C10	C9	C4	129.69(17)
C10	C9	C8	120.14(17)
C9	C10	C11	119.26(18)
C12	C11	C10	120.03(18)
C11	C12	C13	121.74(18)
C8	C13	C12	117.44(17)

Table 23: Torsion Angles in ° for **JW622B**.

Atom	Atom	Atom	Atom	Angle/°
Br1	C1	C2	O1	-2.0(3)
Br1	C1	C2	C3	177.64(12)
Br1	C1	C5	N1	47.71(19)
Br1	C1	C5	C4	161.56(12)
Br1	C1	C6	C7	-151.72(12)
O1	C2	C3	C4	-126.0(2)
O1	C2	C3	C7	123.0(2)
O1	C2	C3	C14	0.6(3)
N1	C8	C9	C4	4.4(2)
N1	C8	C9	C10	-179.96(16)
N1	C8	C13	C12	178.60(17)
C1	C2	C3	C4	54.38(15)
C1	C2	C3	C7	-56.55(15)
C1	C2	C3	C14	-179.01(15)
C1	C6	C7	C3	-8.37(18)
C2	C1	C5	N1	-76.08(17)
C2	C1	C5	C4	37.76(16)
C2	C1	C6	C7	-26.73(17)
C2	C3	C4	C5	-30.56(17)
C2	C3	C4	C9	78.96(17)

The Rigaku Synergy S Diffractometer was purchased with Support from the MRI program by the National Science Foundation under Grant No. 1919565.

Atom	Atom	Atom	Atom	Angle/°
C2	C3	C4	C15	-153.45(15)
C2	C3	C7	C6	40.32(17)
C3	C4	C5	N1	115.12(15)
C3	C4	C5	C1	-4.57(17)
C3	C4	C9	C8	-111.59(17)
C3	C4	C9	C10	73.3(2)
C4	C3	C7	C6	-62.78(18)
C4	C9	C10	C11	176.02(18)
C5	N1	C8	C9	-6.5(2)
C5	N1	C8	C13	172.84(17)
C5	C1	C2	O1	122.0(2)
C5	C1	C2	C3	-58.41(15)
C5	C1	C6	C7	77.99(17)
C5	C4	C9	C8	-0.71(18)
C5	C4	C9	C10	-175.80(18)
C6	C1	C2	O1	-126.9(2)
C6	C1	C2	C3	52.71(16)
C6	C1	C5	N1	177.85(15)
C6	C1	C5	C4	-68.31(17)
C7	C3	C4	C5	71.25(17)
C7	C3	C4	C9	-179.23(15)
C7	C3	C4	C15	-51.6(2)
C8	N1	C5	C1	117.52(16)
C8	N1	C5	C4	5.69(19)
C8	C9	C10	C11	1.3(3)
C9	C4	C5	N1	-2.90(17)
C9	C4	C5	C1	-122.59(14)
C9	C8	C13	C12	-2.1(3)
C9	C10	C11	C12	-1.8(3)
C10	C11	C12	C13	0.3(3)
C11	C12	C13	C8	1.6(3)
C13	C8	C9	C4	-174.97(16)
C13	C8	C9	C10	0.7(3)
C14	C3	C4	C5	-156.26(16)
C14	C3	C4	C9	-46.7(2)
C14	C3	C4	C15	80.8(2)
C14	C3	C7	C6	163.51(16)
C15	C4	C5	N1	-121.33(16)
C15	C4	C5	C1	118.99(16)
C15	C4	C9	C8	119.22(16)
C15	C4	C9	C10	-55.9(2)
C16	N1	C5	C1	-93.1(2)
C16	N1	C5	C4	155.06(16)
C16	N1	C8	C9	-156.24(17)
C16	N1	C8	C13	23.1(3)

Table 24: Hydrogen Fractional Atomic Coordinates (×10^4) and Equivalent Isotropic Displacement Parameters (Å^2×10^3) for **JW622B**. U_{eq} is defined as 1/3 of the trace of the orthogonalised U_{ij}.

Atom	x	y	z	U_{eq}
H5	6777.83	2608.82	4314.61	21
H6A	8261.47	1351.89	5142.22	27
H6B	9246.97	2696.22	5466.49	27
H7A	9886.17	1729.01	4286.42	26
H7B	8987.56	132.77	4052.97	26

The Rigaku Synergy S Diffractometer was purchased with Support from the MRI program by the National Science Foundation under Grant No. 1919565.

Atom	x	y	z	U_{eq}
H10	7268.6	2435.03	1314.59	26
H11	6230.59	4544.27	303.8	29
H12	5250.63	6949.23	804.21	27
H13	5279.39	7355.41	2317.71	23
H14A	9050.73	1518.22	2301.32	36
H14B	9882.83	3040.98	2795.2	36
H14C	8891.31	3889.96	2175.51	36
H15A	6475.33	171.13	2961.89	33
H15B	7474	-412.66	3645.43	33
H15C	7475.05	-307.63	2613.13	33
H16A	5726.02	7498.74	4014.65	34
H16B	5872.57	5620.71	4673.16	34
H16C	5077.35	5505.9	3763.62	34

Citations

CrysAlisPro (Rigaku, V1.171.42.59a, 2022)

CrysAlisPro (ROD), Rigaku Oxford Diffraction, Poland (?).

O.V. Dolomanov and L.J. Bourhis and R.J. Gildea and J.A.K. Howard and H. Puschmann, Olex2: A complete structure solution, refinement and analysis program, *J. Appl. Cryst.*, (2009), **42**, 339-341.

Sheldrick, G.M., Crystal structure refinement with ShelXL, *Acta Cryst.*, (2015), **C71**, 3-8.

Sheldrick, G.M., ShelXT-Integrated space-group and crystal-structure determination, *Acta Cryst.*, (2015), **A71**, 3-8.

The Rigaku Synergy S Diffractometer was purchased with Support from the MRI program by the National Science Foundation under Grant No. 1919565.

Compound 10b

Submitted by: **Jimmy Wu**

Date Collected: **9-23-2022**

Solved by: **Richard J Staples**

Sample ID: **ZTP-4-33**

Center Crystallographic for Research
Michigan State University
Department of Chemistry
East Lansing, MI 48824
Dr. Richard J. Staples
staples@chemistry.msu.edu

Crystal structure and relative chirality was determined.

Crystal Data and Experimental

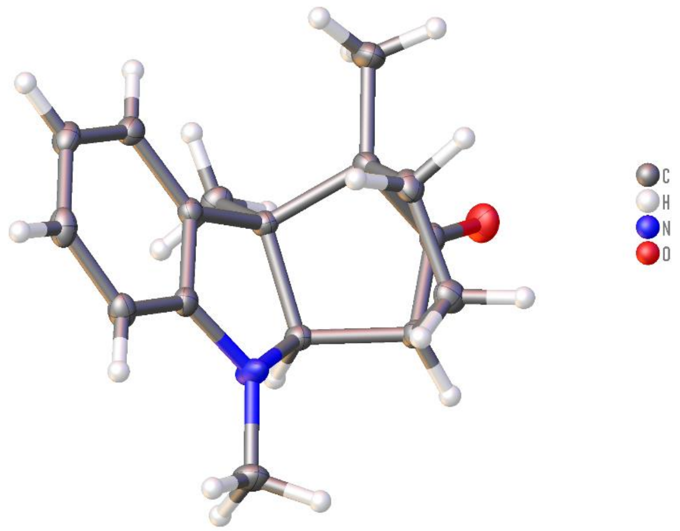

**Compound**	**JW922A**
Formula	$C_{16}H_{19}NO$
$D_{calc.}$/ g cm^{-3}	1.233
μ/mm^{-1}	0.593
Formula Weight	241.32
Color	colourless
Shape	irregular-shaped
Size/mm^3	0.20×0.17×0.17
T/K	100.00(10)
Crystal System	monoclinic
Space Group	$P2_1/n$
a/Å	14.04898(14)
b/Å	6.21142(6)
c/Å	15.50317(18)
α/°	90
β/°	106.1247(12)
γ/°	90
V/Å^3	1299.65(2)
Z	4
Z'	1
Wavelength/Å	1.54184
Radiation type	Cu K_α
Θ_{min}/°	3.760
Θ_{max}/°	80.079
Measured Refl's.	28434
Indep't Refl's	2808
Refl's I≥2 σ(I)	2690
R_{int}	0.0323
Parameters	167
Restraints	0
Largest Peak	0.304
Deepest Hole	-0.297
GooF	1.088
wR_2 (all data)	0.0967
wR_2	0.0959
R_1 (all data)	0.0390
R_1	0.0379

Experimental. Single colourless irregular-shaped crystals of **JW922A** used as receieved. A suitable crystal with dimensions 0.20 × 0.17 × 0.17 mm^3 was selected and mounted on a nylon loop with paratone oil on a XtaLAB Synergy, Dualflex, HyPix diffractometer. The crystal was kept at a steady T = 100.00(10) K during data collection. The structure was solved with the **ShelXS** (Sheldrick, 2008) solution program using direct methods and by using **Olex2** 1.5 (Dolomanov et al., 2009) as the graphical interface. The model was refined with **ShelXL** 2018/3 (Sheldrick, 2015) using full matrix least squares minimisation on $\boldsymbol{F}^2$.

Crystal Data. $C_{16}H_{19}NO$, M_r = 241.32, monoclinic, $P2_1/n$ (No. 14), a = 14.04898(14) Å, b = 6.21142(6) Å, c = 15.50317(18) Å, β = 106.1247(12)°, α = γ = 90°, V = 1299.65(2) Å^3, T = 100.00(10) K, Z = 4, Z' = 1, μ(Cu K_α) = 0.593, 28434 reflections measured, 2808 unique (R_{int} = 0.0323) which were used in all calculations. The final wR_2 was 0.0967 (all data) and R_1 was 0.0379 (I≥2 σ(I)).

The Rigaku Synergy S Diffractometer was purchased with Support from the MRI program by the National Science Foundation under Grant No. 1919565.

Structure Quality Indicators

**Reflections:**	d min (Cu\a) 2Θ=160.3° 0.78	I/σ(I) 70.8	Rint 3.23%	Full 135.4° 99% to 160.3° 100
**Refinement:**	Shift 0.000	Max Peak 0.3	Min Peak -0.3	GooF 1.088

A colourless irregular-shaped-shaped crystal with dimensions 0.20×0.17×0.17 mm^3 was mounted on a nylon loop with paratone oil. Data were collected using a XtaLAB Synergy, Dualflex, HyPix diffractometer equipped with an Oxford Cryosystems low-temperature device, operating at T = 100.00(10) K.

MSU Data were measured using ω scans using Cu K_{α} radiation (micro-focus sealed X-ray tube, 50 kV, 1 mA). The total number of runs and images was based on the strategy calculation from the program CrysAlisPro 1.171.42.69a (Rigaku OD, 2022). The achieved resolution was Θ = 80.079.

Cell parameters were retrieved using the CrysAlisPro 1.171.42.69a (Rigaku OD, 2022) software and refined using CrysAlisPro 1.171.42.69a (Rigaku OD, 2022) on 19091 reflections, 67 % of the observed reflections. Data reduction was performed using the CrysAlisPro 1.171.42.69a (Rigaku OD, 2022) software which corrects for Lorentz polarization. The final completeness is 100.00 out to 80.079 in Θ CrysAlisPro 1.171.42.69a (Rigaku Oxford Diffraction, 2022) Numerical absorption correction based on gaussian integration over a multifaceted crystal model Empirical absorption correction using spherical harmonics, implemented in SCALE3 ABSPACK scaling algorithm.

The structure was solved in the space group $P2_1/n$ (# 14) by using direct methods using the ShelXS (Sheldrick, 2008) structure solution program. The structure was refined by Least Squares ShelXL incorporated in Olex2 software program. All non-hydrogen atoms were refined anisotropically. Hydrogen atom positions were calculated geometrically and refined using the riding model, except for the hydrogen atom on the non-carbon atom(s) which were found by difference Fourier methods and refined isotropically when data permits.

There is a single molecule in the asymmetric unit, which is represented by the reported sum formula. In other words: Z is 4 and Z' is 1.

The Rigaku Synergy S Diffractometer was purchased with Support from the MRI program by the National Science Foundation under Grant No. 1919565.

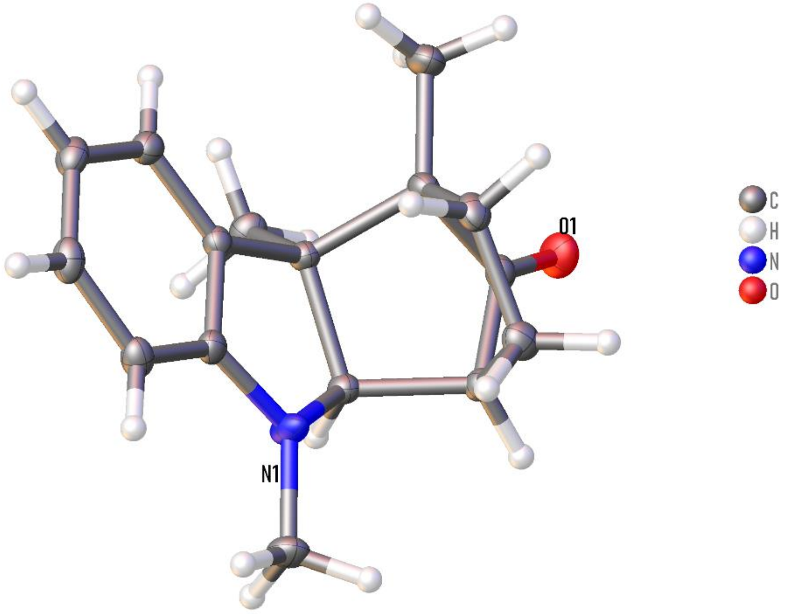

Figure 12:

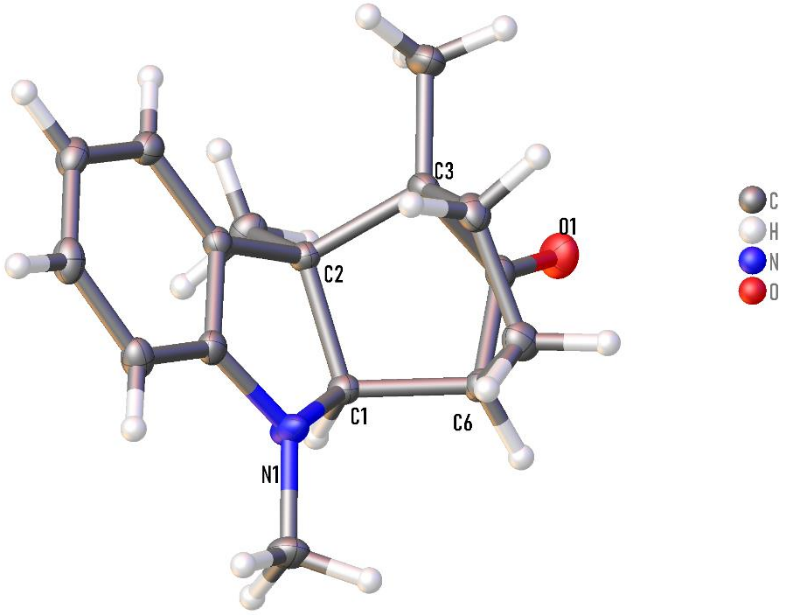

Figure 13: Model has Chirality at C1 (Centro SPGR) R. Model has Chirality at C2 (Centro SPGR) S. Model has Chirality at C3 (Centro SPGR) R. Model has Chirality at C6 (Centro SPGR) S.

The Rigaku Synergy S Diffractometer was purchased with Support from the MRI program by the National Science Foundation under Grant No. 1919565.

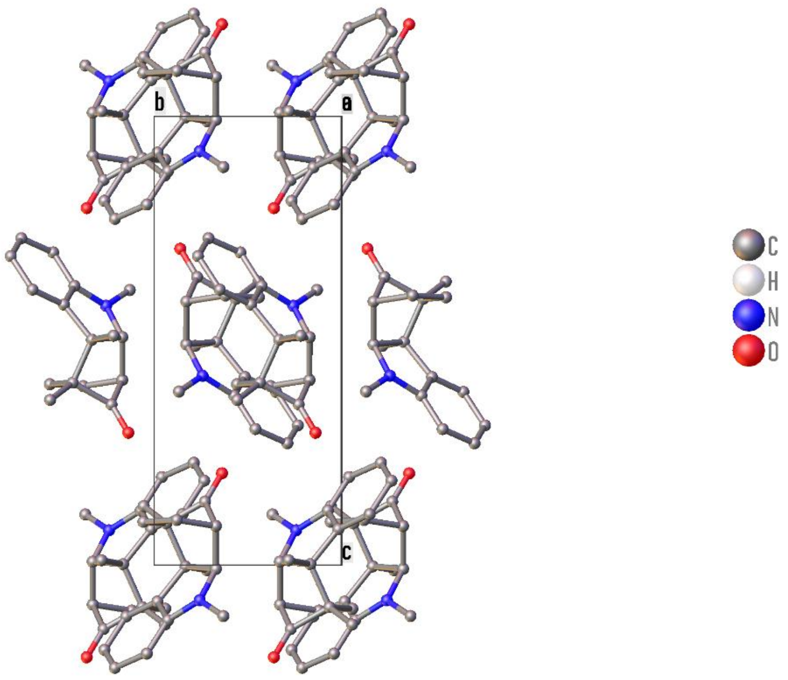

Figure 14: Packing diagram of JW922A.

Data Plots: Diffraction Data

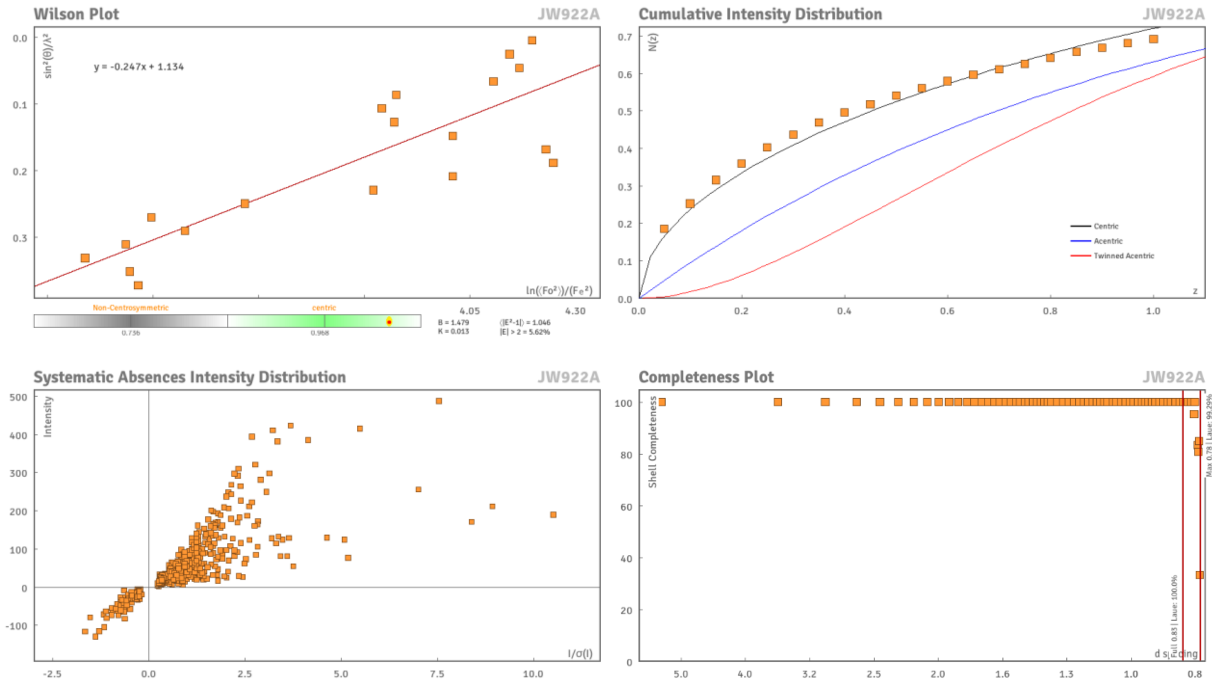

The Rigaku Synergy S Diffractometer was purchased with Support from the MRI program by the National Science Foundation under Grant No. 1919565.

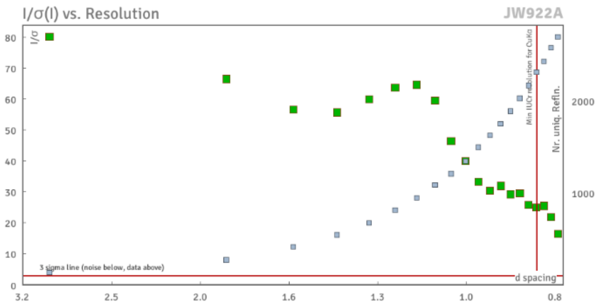

Data Plots: Refinement and Data

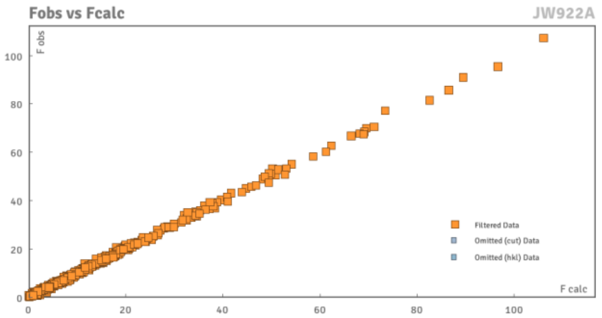

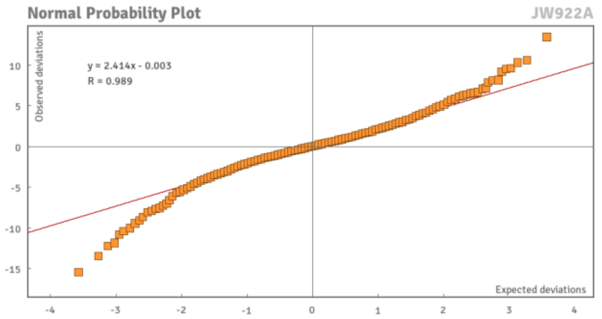

Reflection Statistics

Total reflections (after filtering)	29823	Unique reflections	2808
Completeness	0.993	Mean I/σ	43.28
hkl_{max} collected	(17, 7, 16)	hkl_{min} collected	(-17, -7, -19)
hkl_{max} used	(17, 7, 19)	hkl_{min} used	(-17, 0, 0)
Lim d_{max} collected	100.0	Lim d_{min} collected	0.77
d_{max} used	14.89	d_{min} used	0.78
Friedel pairs	3900	Friedel pairs merged	1
Inconsistent equivalents	1	R_{int}	0.0323
R_{sigma}	0.0141	Intensity transformed	0
Omitted reflections	0	Omitted by user (OMIT hkl)	0
Multiplicity	(1613, 2326, 2089, 1507, 915, 538, 243, 98, 54, 23, 17, 6)	Maximum multiplicity	26
Removed systematic absences	1389	Filtered off (Shel/OMIT)	0

Selected Crystal Pictures

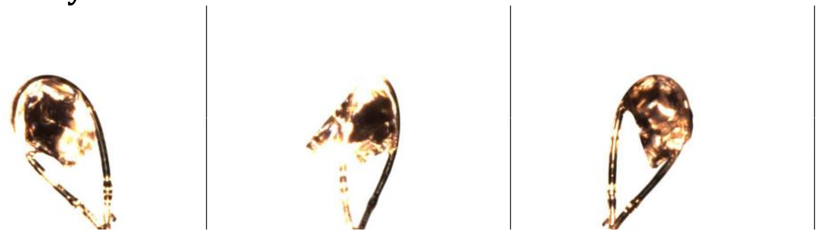

The Rigaku Synergy S Diffractometer was purchased with Support from the MRI program by the National Science Foundation under Grant No. 1919565.

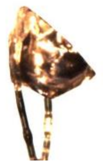

Table 25: Fractional Atomic Coordinates (×10[4]) and Equivalent Isotropic Displacement Parameters (Å[2]×10[3]) for **JW922A**. U_{eq} is defined as 1/3 of the trace of the orthogonalised U_{ij}.

Atom	x	y	z	U_{eq}
C1	7027.4(7)	1708.9(17)	4919.4(7)	15.6(2)
C2	7909.0(7)	3335.8(16)	4974.9(7)	14.8(2)
C3	8069.3(7)	4356.9(17)	5932.3(7)	17.0(2)
C4	7120.4(8)	5581.8(18)	5968.0(7)	20.2(2)
C5	6351.8(8)	3797.0(18)	6009.7(8)	20.9(2)
C6	6877.2(7)	1711.6(17)	5874.3(7)	16.1(2)
C7	7925.0(7)	2289.7(17)	6419.2(7)	16.8(2)
C8	6492.8(7)	4269.4(17)	3779.0(7)	16.1(2)
C9	5933.8(8)	5401.5(18)	3034.2(7)	19.2(2)
C10	6390.6(8)	7106.5(18)	2717.0(7)	20.5(2)
C11	7369.9(8)	7666.6(17)	3115.7(7)	19.8(2)
C12	7923.3(8)	6506.4(17)	3861.1(7)	17.4(2)
C13	7483.2(7)	4831.2(17)	4193.6(7)	14.9(2)
C14	5319.7(8)	1312.3(19)	3869.1(8)	23.1(2)
C15	8840.6(8)	2178.8(18)	4887.3(7)	19.9(2)
C16	9027.3(9)	5568(2)	6318.9(8)	25.4(3)
N1	6199.0(7)	2576.9(16)	4221.1(6)	21.9(2)
O1	8483.7(6)	1387.5(14)	7047.6(5)	25.3(2)

Table 26: Anisotropic Displacement Parameters (×10[4]) for **JW922A**. The anisotropic displacement factor exponent takes the form: $-2\pi^2[h^2a^{*2} \times U_{11} + \ldots + 2hka^* \times b^* \times U_{12}]$

Atom	U_{11}	U_{22}	U_{33}	U_{23}	U_{13}	U_{12}
C1	16.1(5)	15.4(5)	14.9(5)	-0.2(4)	3.7(4)	-2.4(4)
C2	14.8(5)	15.1(5)	14.3(5)	0.2(4)	3.7(4)	-2.0(4)
C3	18.4(5)	18.0(5)	13.6(5)	-0.3(4)	2.8(4)	-3.3(4)
C4	27.1(6)	16.9(5)	17.3(5)	-2.1(4)	7.1(4)	0.8(4)
C5	19.2(5)	22.9(6)	21.3(5)	-3.0(4)	6.5(4)	1.6(4)
C6	16.1(5)	17.1(5)	15.3(5)	1.7(4)	4.7(4)	-1.2(4)
C7	18.0(5)	19.7(5)	13.3(5)	-0.2(4)	5.1(4)	0.0(4)
C8	16.7(5)	18.8(5)	13.9(5)	-0.9(4)	6.0(4)	-0.8(4)
C9	16.2(5)	25.5(6)	16.0(5)	0.4(4)	4.7(4)	2.9(4)
C10	24.3(5)	21.7(5)	16.4(5)	3.8(4)	7.3(4)	7.8(4)
C11	26.8(5)	15.9(5)	19.5(5)	2.5(4)	10.9(4)	0.9(4)
C12	18.7(5)	17.1(5)	17.5(5)	-1.3(4)	6.7(4)	-1.7(4)
C13	16.4(5)	15.6(5)	13.0(5)	-1.0(4)	4.6(4)	0.1(4)
C14	17.2(5)	25.7(6)	23.9(5)	-2.2(4)	2.0(4)	-6.9(4)
C15	17.1(5)	21.8(5)	21.2(5)	2.0(4)	5.7(4)	1.5(4)
C16	25.5(6)	29.2(6)	19.4(5)	-3.0(5)	2.9(4)	-11.6(5)
N1	17.9(4)	27.4(5)	16.5(4)	5.6(4)	-1.7(3)	-9.1(4)
O1	22.6(4)	32.1(5)	18.5(4)	7.8(3)	1.2(3)	1.5(3)

The Rigaku Synergy S Diffractometer was purchased with Support from the MRI program by the National Science Foundation under Grant No. 1919565.

Table 27: Bond Lengths in Å for **JW922A**.

Atom	Atom	Length/Å
C1	C2	1.5823(14)
C1	C6	1.5523(14)
C1	N1	1.4551(13)
C2	C3	1.5718(14)
C2	C13	1.5117(14)
C2	C15	1.5318(14)
C3	C4	1.5491(15)
C3	C7	1.5313(14)
C3	C16	1.5129(14)
C4	C5	1.5615(15)
C5	C6	1.5339(15)

Atom	Atom	Length/Å
C6	C7	1.5227(14)
C7	O1	1.2060(13)
C8	C9	1.3931(15)
C8	C13	1.4051(14)
C8	N1	1.3794(14)
C9	C10	1.3966(16)
C10	C11	1.3870(16)
C11	C12	1.4000(15)
C12	C13	1.3812(14)
C14	N1	1.4374(13)

Table 28: Bond Angles in ° for **JW922A**.

Atom	Atom	Atom	Angle/°
C6	C1	C2	105.02(8)
N1	C1	C2	104.84(8)
N1	C1	C6	114.63(8)
C3	C2	C1	102.34(8)
C13	C2	C1	102.66(8)
C13	C2	C3	115.45(8)
C13	C2	C15	112.02(8)
C15	C2	C1	111.76(8)
C15	C2	C3	111.79(8)
C4	C3	C2	109.44(8)
C7	C3	C2	97.08(8)
C7	C3	C4	99.28(8)
C16	C3	C2	116.94(9)
C16	C3	C4	114.92(9)
C16	C3	C7	116.54(9)
C3	C4	C5	105.35(8)
C6	C5	C4	103.25(8)
C5	C6	C1	109.23(8)

Atom	Atom	Atom	Angle/°
C7	C6	C1	99.38(8)
C7	C6	C5	98.79(8)
C6	C7	C3	99.45(8)
O1	C7	C3	129.96(10)
O1	C7	C6	130.59(10)
C9	C8	C13	120.84(10)
N1	C8	C9	128.23(10)
N1	C8	C13	110.92(9)
C8	C9	C10	117.78(10)
C11	C10	C9	121.96(10)
C10	C11	C12	119.56(10)
C13	C12	C11	119.50(10)
C8	C13	C2	109.58(9)
C12	C13	C2	130.05(9)
C12	C13	C8	120.36(9)
C8	N1	C1	111.35(8)
C8	N1	C14	124.90(9)
C14	N1	C1	120.36(9)

Table 29: Torsion Angles in ° for **JW922A**.

Atom	Atom	Atom	Atom	Angle/°
C1	C2	C3	C4	-62.60(10)
C1	C2	C3	C7	39.88(9)
C1	C2	C3	C16	164.51(9)
C1	C2	C13	C8	3.22(11)
C1	C2	C13	C12	-175.46(10)
C1	C6	C7	C3	54.32(9)
C1	C6	C7	O1	-125.35(12)
C2	C1	C6	C5	75.20(10)
C2	C1	C6	C7	-27.60(10)
C2	C1	N1	C8	8.31(11)
C2	C1	N1	C14	168.52(9)
C2	C3	C4	C5	73.73(10)
C2	C3	C7	C6	-59.13(9)
C2	C3	C7	O1	120.54(12)

The Rigaku Synergy S Diffractometer was purchased with Support from the MRI program by the National Science Foundation under Grant No. 1919565.

Atom	Atom	Atom	Atom	Angle/°
C3	C2	C13	C8	-107.24(10)
C3	C2	C13	C12	74.09(14)
C3	C4	C5	C6	-6.90(10)
C4	C3	C7	C6	51.98(9)
C4	C3	C7	O1	-128.35(12)
C4	C5	C6	C1	-64.68(10)
C4	C5	C6	C7	38.53(9)
C5	C6	C7	C3	-56.99(9)
C5	C6	C7	O1	123.34(12)
C6	C1	C2	C3	-7.86(10)
C6	C1	C2	C13	-127.86(8)
C6	C1	C2	C15	111.91(9)
C6	C1	N1	C8	122.91(10)
C6	C1	N1	C14	-76.88(13)
C7	C3	C4	C5	-27.23(10)
C8	C9	C10	C11	0.59(16)
C9	C8	C13	C2	-179.59(9)
C9	C8	C13	C12	-0.77(15)
C9	C8	N1	C1	174.86(10)
C9	C8	N1	C14	15.73(18)
C9	C10	C11	C12	-0.29(16)
C10	C11	C12	C13	-0.54(16)
C11	C12	C13	C2	179.62(10)
C11	C12	C13	C8	1.06(15)
C13	C2	C3	C4	48.05(11)
C13	C2	C3	C7	150.53(8)
C13	C2	C3	C16	-84.84(11)
C13	C8	C9	C10	-0.06(15)
C13	C8	N1	C1	-6.62(12)
C13	C8	N1	C14	-165.75(10)
C15	C2	C3	C4	177.65(8)
C15	C2	C3	C7	-79.86(9)
C15	C2	C3	C16	44.77(12)
C15	C2	C13	C8	123.27(9)
C15	C2	C13	C12	-55.41(14)
C16	C3	C4	C5	-152.34(9)
C16	C3	C7	C6	175.94(9)
C16	C3	C7	O1	-4.38(17)
N1	C1	C2	C3	113.30(9)
N1	C1	C2	C13	-6.70(10)
N1	C1	C2	C15	-126.93(9)
N1	C1	C6	C5	-39.30(12)
N1	C1	C6	C7	-142.10(9)
N1	C8	C9	C10	178.33(10)
N1	C8	C13	C2	1.76(12)
N1	C8	C13	C12	-179.42(9)

Table 30: Hydrogen Fractional Atomic Coordinates (×10^4) and Equivalent Isotropic Displacement Parameters (Å^2×10^3) for **JW922A**. U_{eq} is defined as 1/3 of the trace of the orthogonalised U_{ij}.

Atom	x	y	z	U_{eq}
H1	7203.66	234.69	4754.84	19
H4A	6871.77	6486.73	5426.33	24
H4B	7254.8	6515.12	6506.03	24
H5A	6204.43	3801.44	6598.17	25

The Rigaku Synergy S Diffractometer was purchased with Support from the MRI program by the National Science Foundation under Grant No. 1919565.

Atom	x	y	z	U_{eq}
H5B	5726.63	3992.82	5527.59	25
H6	6600.86	355.5	6055.74	19
H9	5264.11	5026.15	2751.51	23
H10	6019.44	7906.41	2213.27	25
H11	7663.3	8829.8	2884.46	24
H12	8596.3	6868.54	4136.04	21
H14A	4862.78	2093.53	3375.47	35
H14B	4998.75	1045.08	4345.16	35
H14C	5498.19	-63.91	3646.79	35
H15A	8678.9	1338.11	4330.56	30
H15B	9087.72	1214.34	5401.15	30
H15C	9351.06	3243.73	4874.22	30
H16A	9589.46	4598.47	6365.01	38
H16B	9046.12	6112.05	6916.81	38
H16C	9067	6779.58	5925.21	38

Citations

CrysAlisPro (Rigaku, V1.171.42.69a, 2022)

CrysAlisPro (ROD), Rigaku Oxford Diffraction, Poland (?).

O.V. Dolomanov and L.J. Bourhis and R.J. Gildea and J.A.K. Howard and H. Puschmann, Olex2: A complete structure solution, refinement and analysis program, *J. Appl. Cryst.*, (2009), **42**, 339-341.

Sheldrick, G.M., A short history of ShelX, *Acta Cryst.*, (2008), **A64**, 339-341.

Sheldrick, G.M., Crystal structure refinement with ShelXL, *Acta Cryst.*, (2015), **C71**, 3-8.

The Rigaku Synergy S Diffractometer was purchased with Support from the MRI program by the National Science Foundation under Grant No. 1919565.

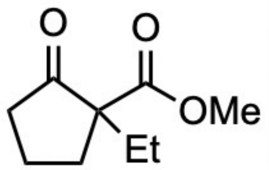
O
O
OMe
Et

18b

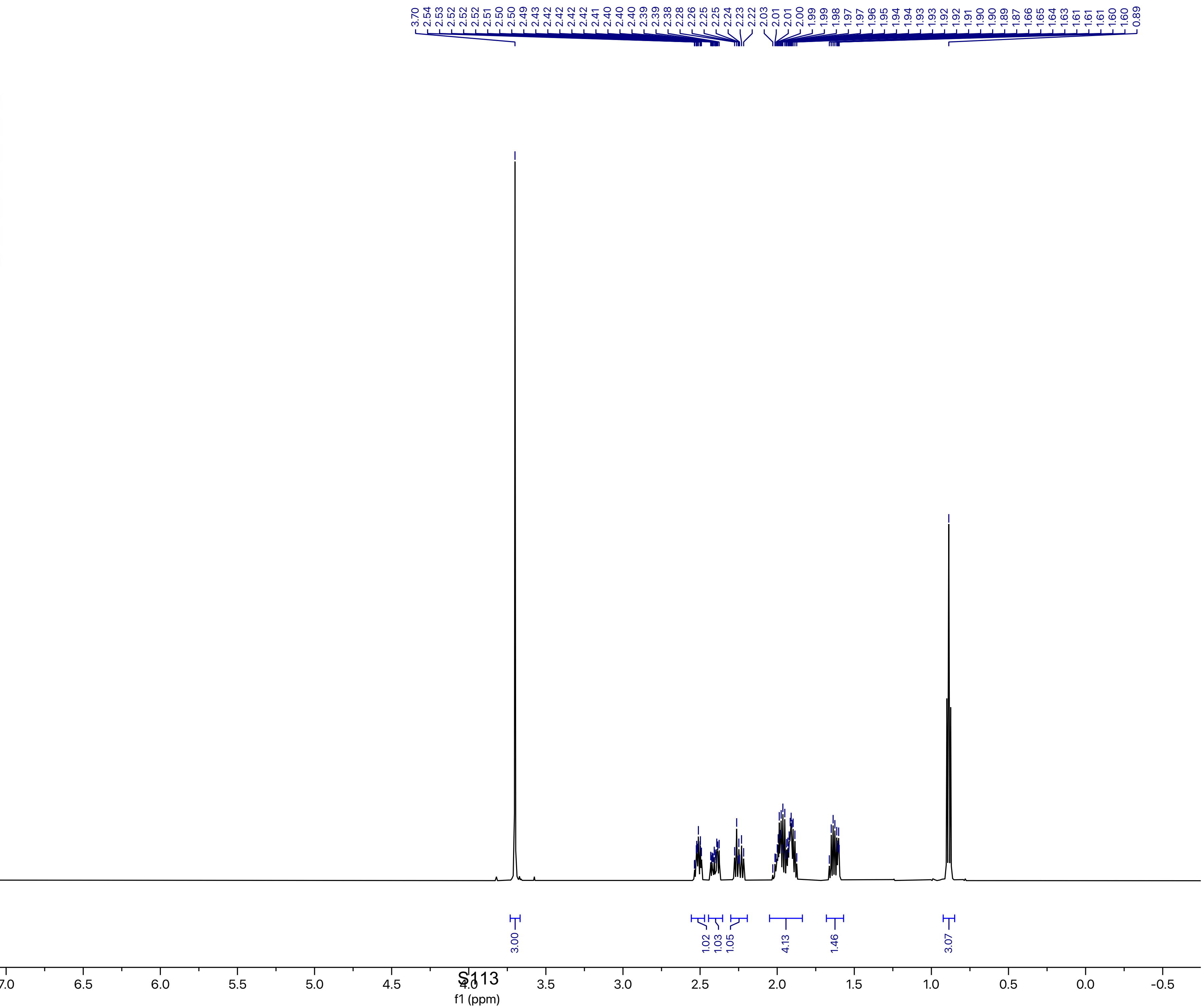
3.70 2.54 2.53 2.52 2.52 2.52 2.51 2.50 2.50 2.49 2.43 2.42 2.42 2.42 2.42 2.41 2.40 2.40 2.40 2.39 2.39 2.38 2.28 2.26 2.25 2.25 2.24 2.23 2.22 2.03 2.01 2.01 2.00 1.99 1.99 1.98 1.97 1.97 1.96 1.95 1.94 1.94 1.93 1.93 1.92 1.92 1.91 1.90 1.90 1.89 1.87 1.66 1.65 1.64 1.63 1.61 1.61 1.61 1.60 1.60 0.89
3.00
1.02
1.03
1.05
4.13
1.46
3.07
8.5 8.0 7.5 7.0 6.5 6.0 5.5 5.0 4.5 4.0 3.5 3.0 2.5 2.0 1.5 1.0 0.5 0.0 -0.5
f1 (ppm)

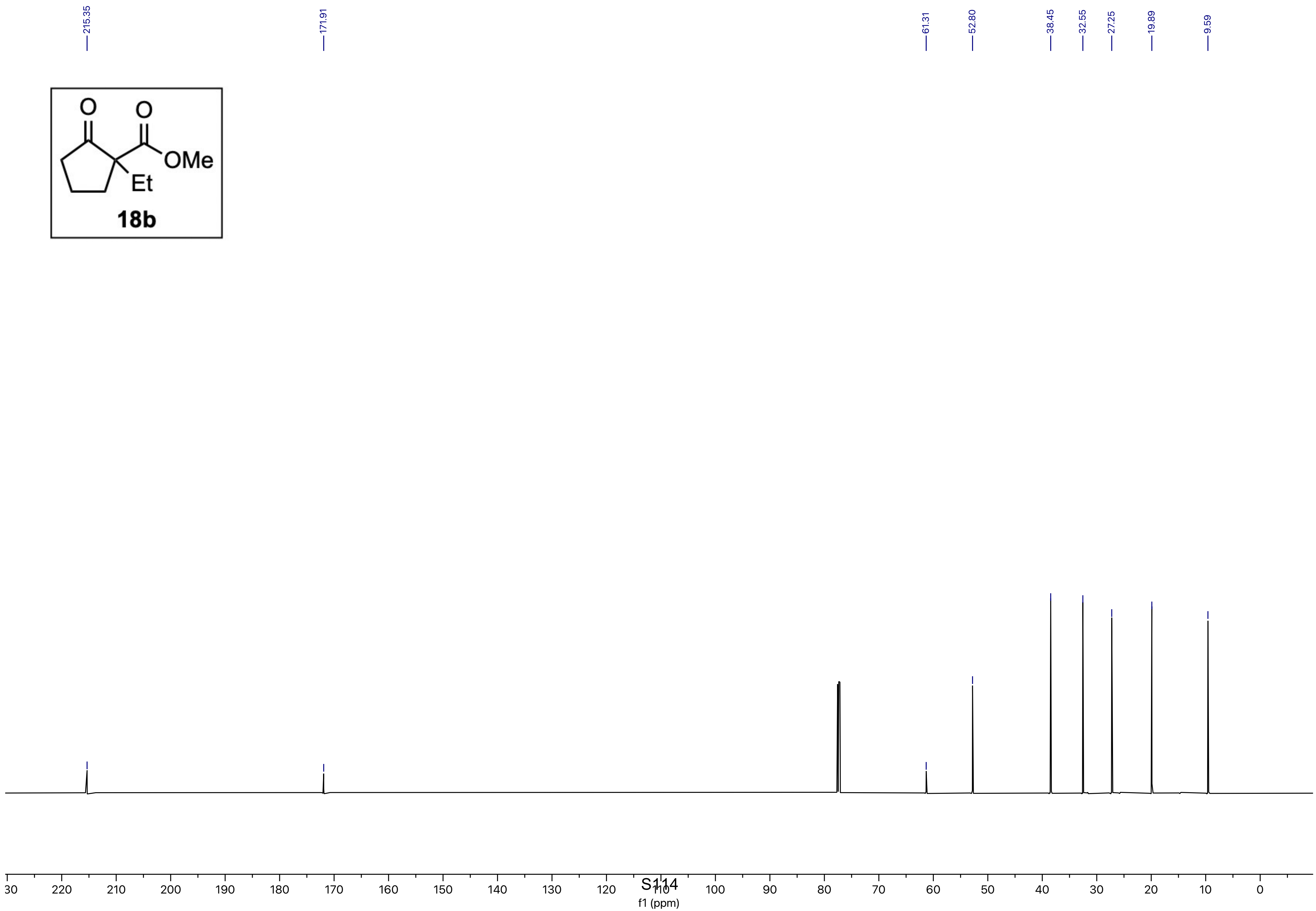
215.35
171.91
61.31
52.80
38.45
32.55
27.25
19.89
9.59
OMe
Et
18b
f1 (ppm)

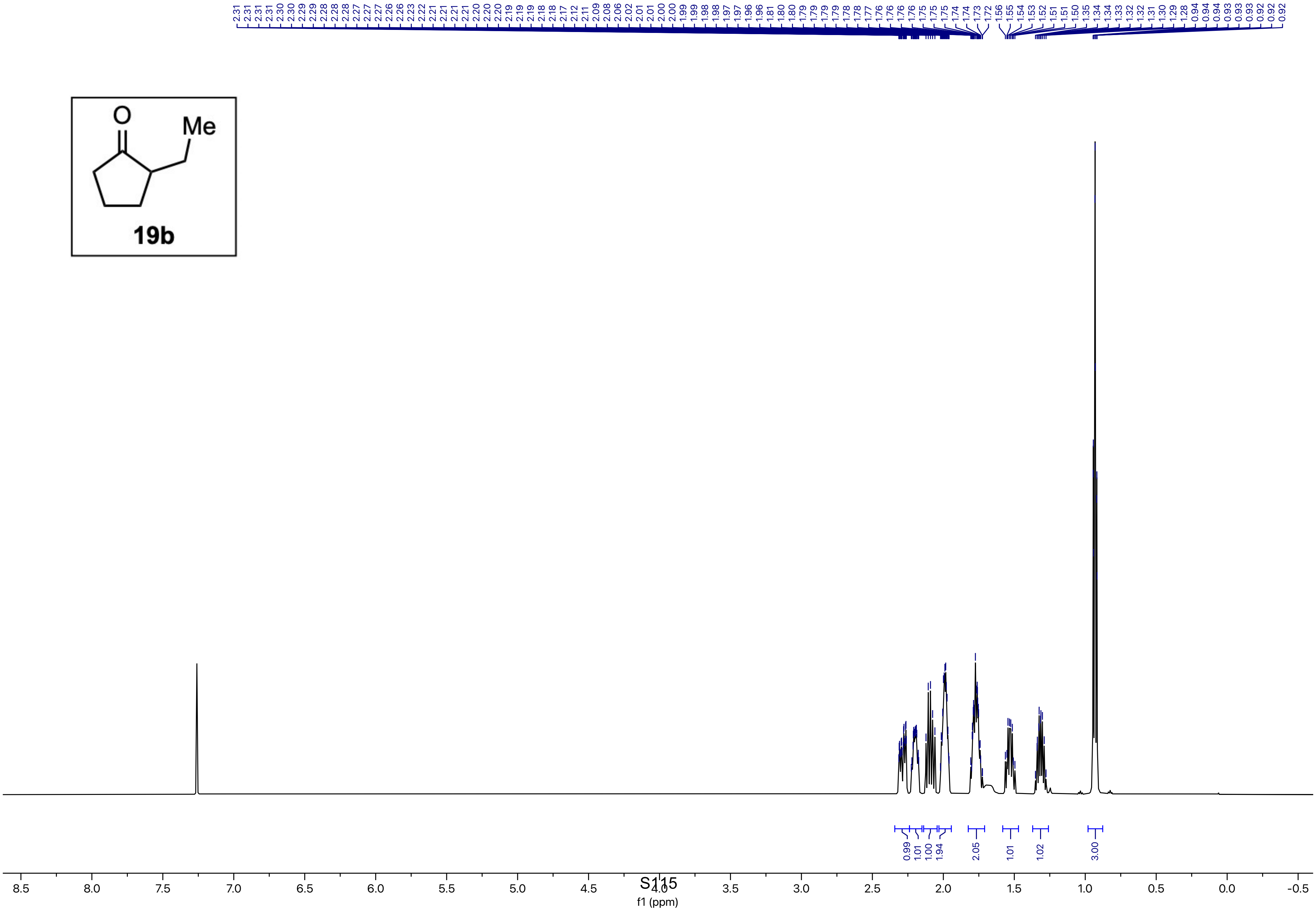
2.31 2.31 2.31 2.30 2.29 2.29 2.28 2.28 2.28 2.27 2.27 2.26 2.26 2.23 2.22 2.21 2.21 2.21 2.20 2.20 2.19 2.19 2.19 2.18 2.17 2.12 2.11 2.09 2.08 2.06 2.02 2.01 2.00 2.00 1.99 1.99 1.98 1.98 1.97 1.97 1.96 1.96 1.81 1.80 1.80 1.79 1.79 1.79 1.79 1.78 1.78 1.77 1.76 1.76 1.76 1.76 1.75 1.75 1.74 1.74 1.73 1.72 1.56 1.55 1.54 1.53 1.52 1.51 1.51 1.50 1.35 1.34 1.34 1.33 1.32 1.32 1.31 1.30 1.29 1.28 0.94 0.94 0.94 0.93 0.93 0.93 0.92 0.92 0.92
O
Me
19b
0.99
1.01
1.00
1.94
2.05
1.01
1.02
3.00
8.5 8.0 7.5 7.0 6.5 6.0 5.5 5.0 4.5 4.0 3.5 3.0 2.5 2.0 1.5 1.0 0.5 0.0 -0.5
f1 (ppm)

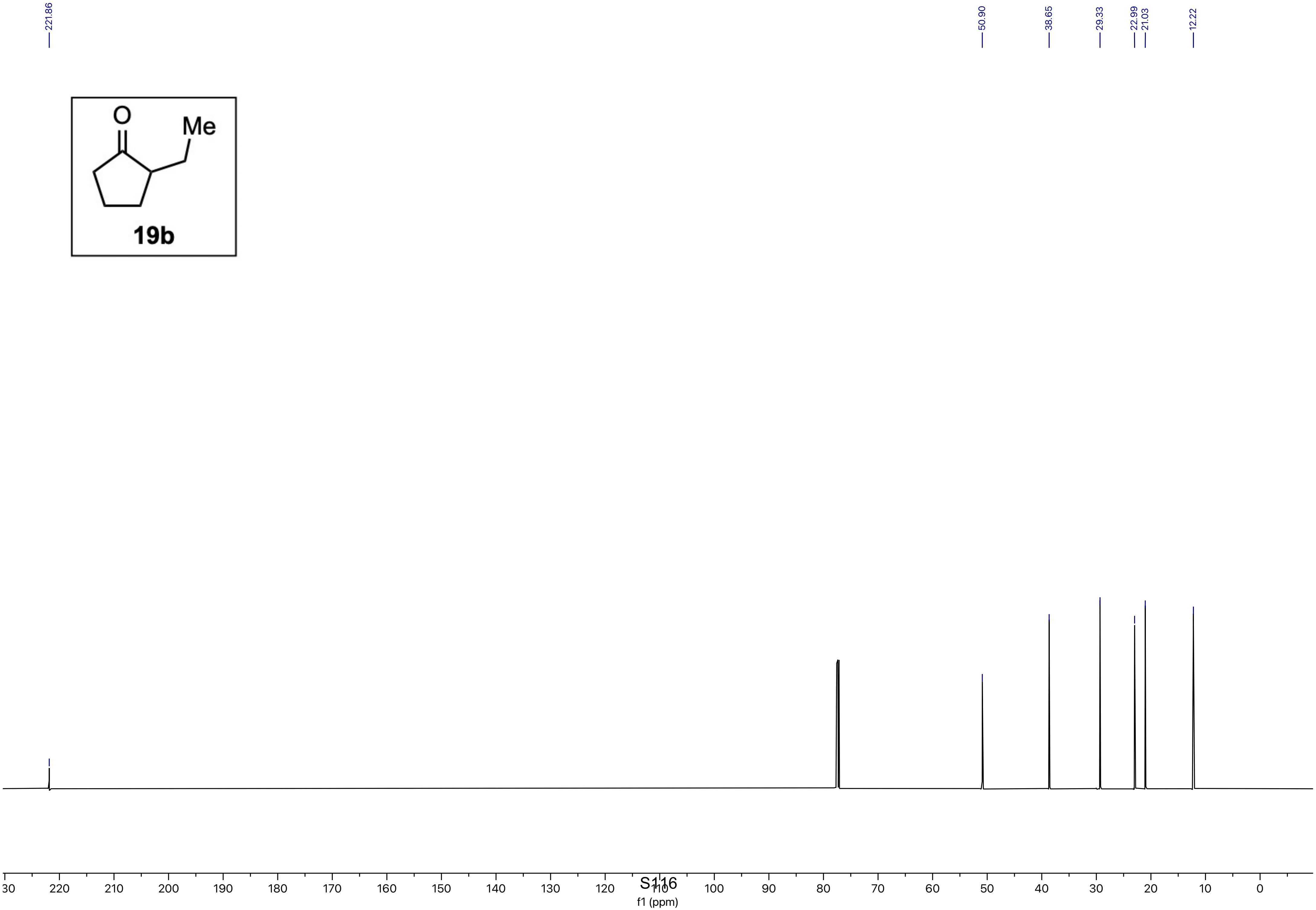
221.86
50.90
38.65
29.33
22.99
21.03
12.22
O
Me
19b
30 220 210 200 190 180 170 160 150 140 130 120 110 100 90 80 70 60 50 40 30 20 10 0
f1 (ppm)

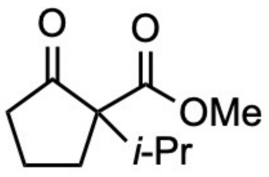
O
O
OMe
i-Pr

18c

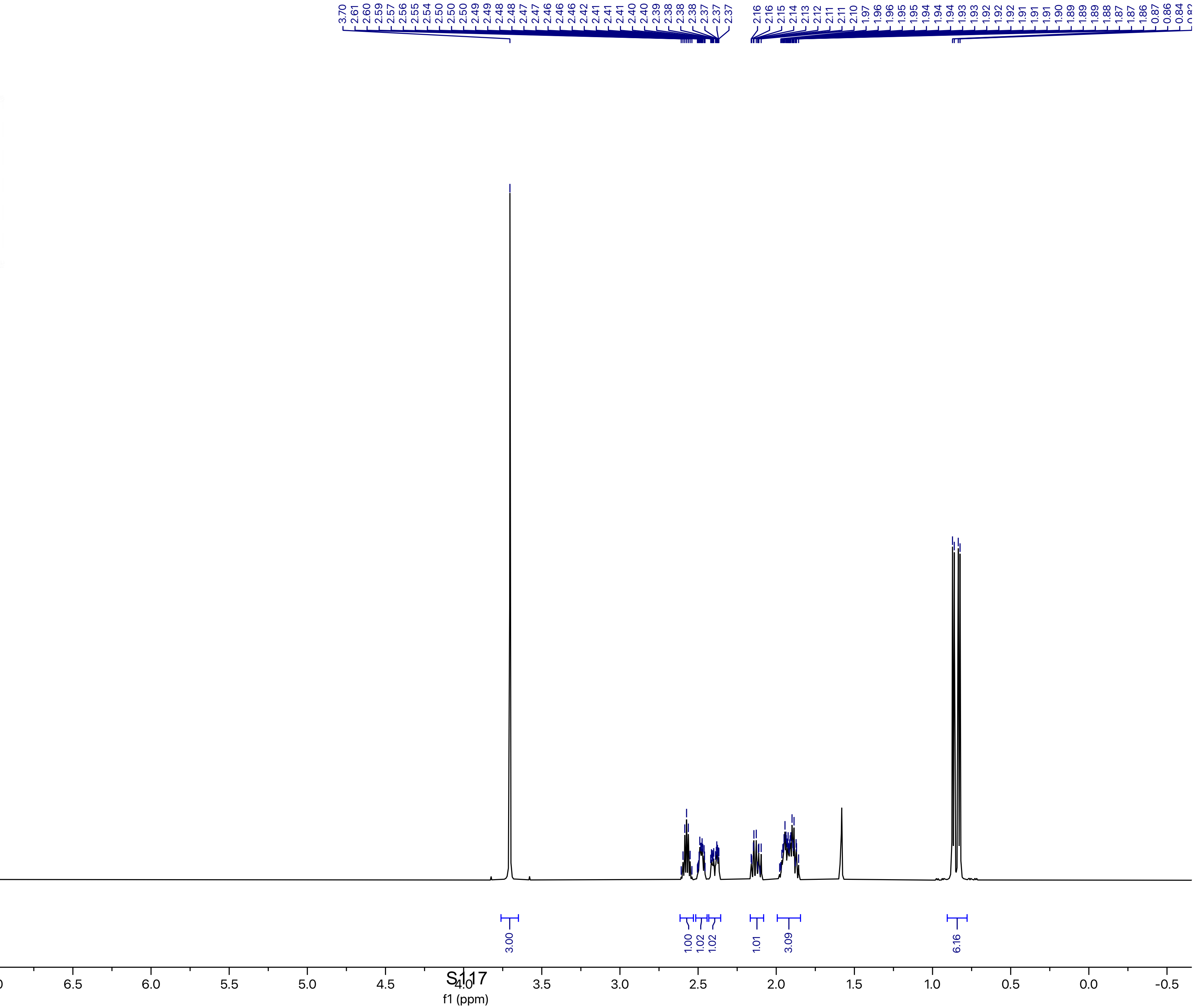
3.70
2.61 2.60 2.59 2.57 2.56 2.55 2.54 2.50 2.50 2.50 2.49 2.49 2.48 2.48 2.47 2.47 2.46 2.46 2.46 2.42 2.41 2.41 2.41 2.40 2.40 2.39 2.38 2.38 2.38 2.37 2.37 2.37
2.16 2.16 2.15 2.14 2.13 2.12 2.11 2.11 2.10 1.97 1.96 1.96 1.95 1.95 1.94 1.94 1.94 1.93 1.93 1.92 1.92 1.92 1.91 1.91 1.91 1.90 1.89 1.89 1.89 1.88 1.87 1.87 1.86
0.87 0.86 0.84
3.00
1.00
1.02
1.02
1.01
3.09
6.16
8.5 8.0 7.5 7.0 6.5 6.0 5.5 5.0 4.5 4.0 3.5 3.0 2.5 2.0 1.5 1.0 0.5 0.0 -0.5
f1 (ppm)

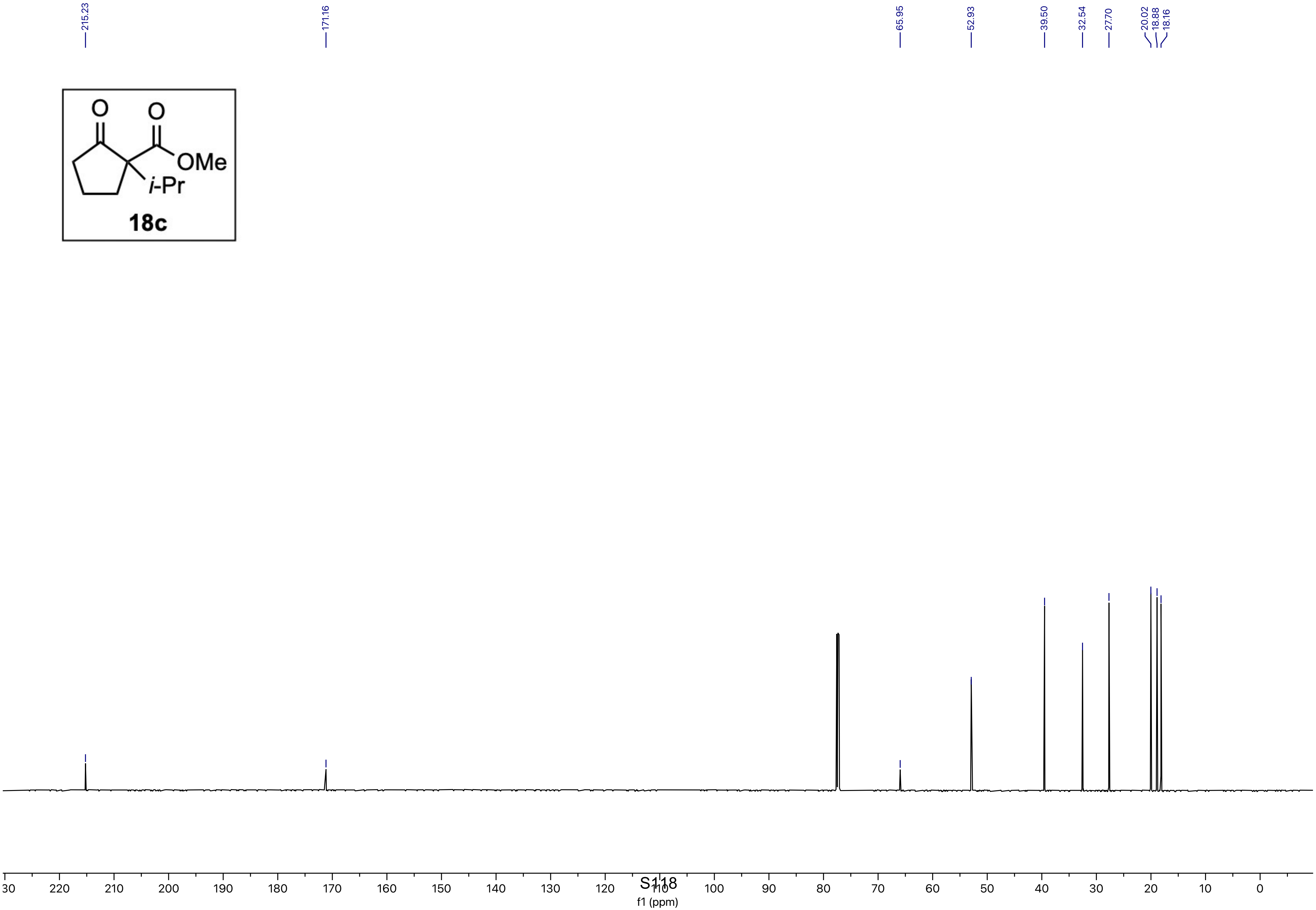
215.23
171.16
65.95
52.93
39.50
32.54
27.70
20.02
18.88
18.16
O
O
OMe
i-Pr
18c
30
220
210
200
190
180
170
160
150
140
130
120
110
100
90
80
70
60
50
40
30
20
10
0
f1 (ppm)

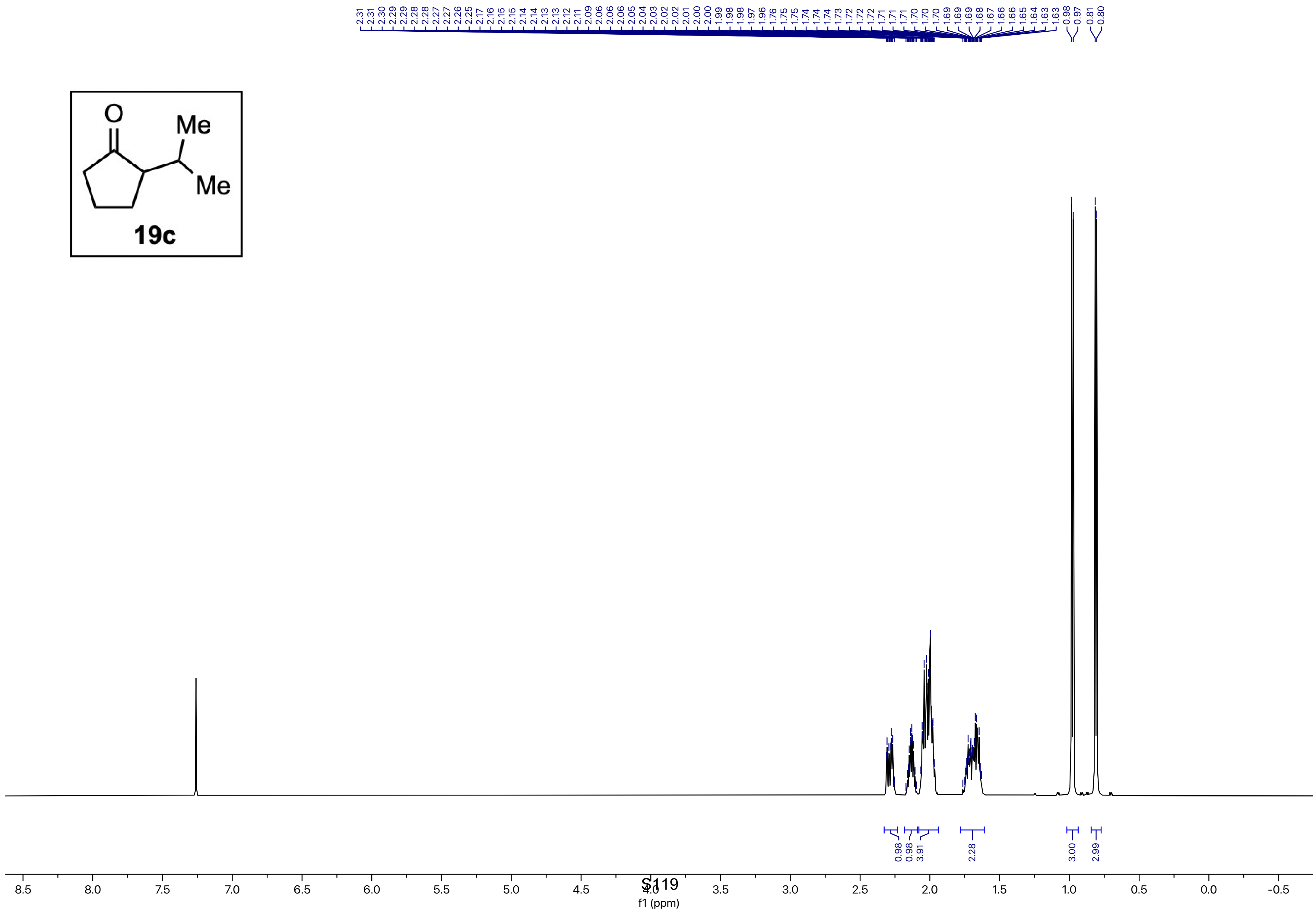
O
Me
Me
19c
f1 (ppm)

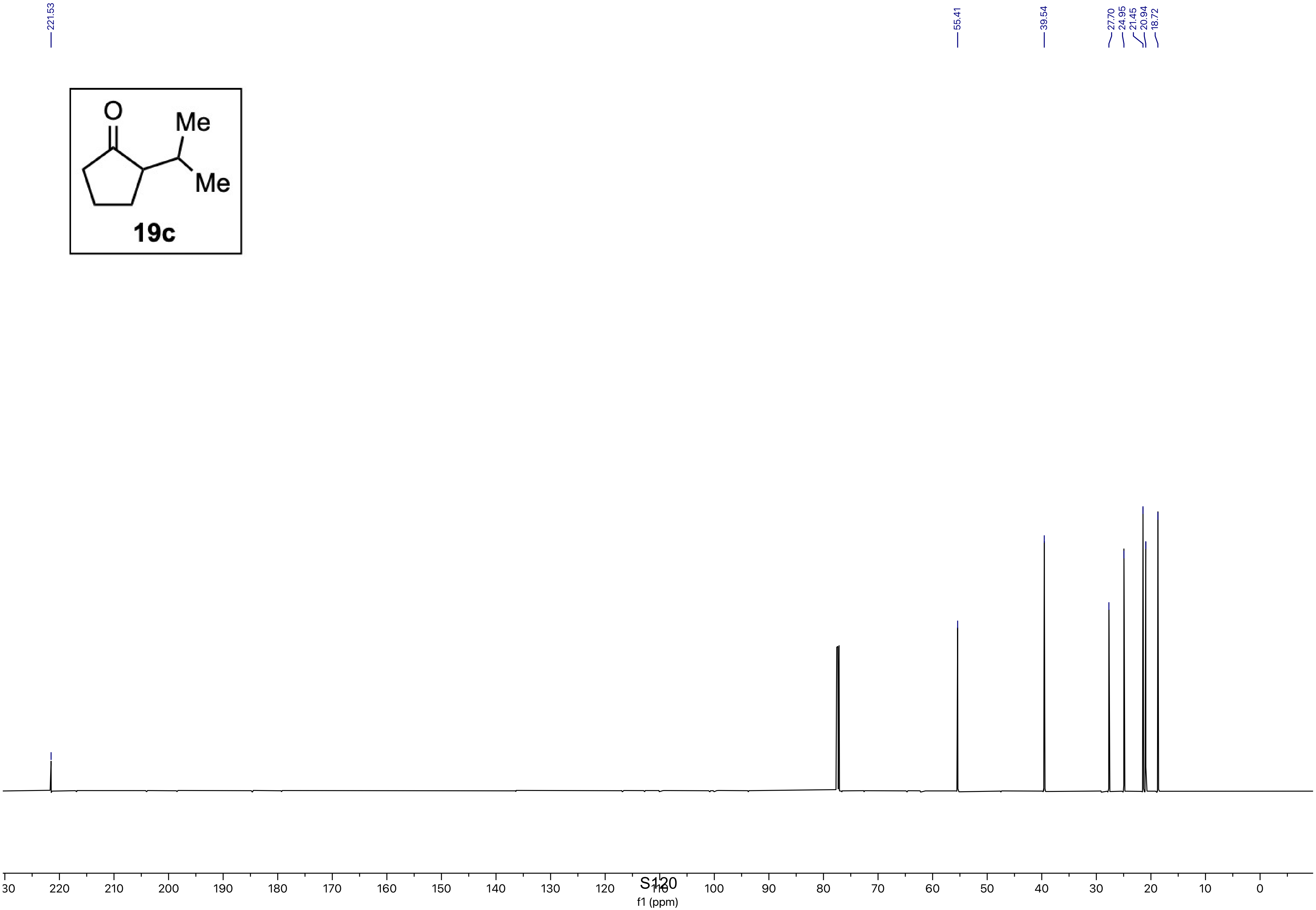

19c
221.53
55.41
39.54
27.70
24.95
21.45
20.94
18.72
f1 (ppm)

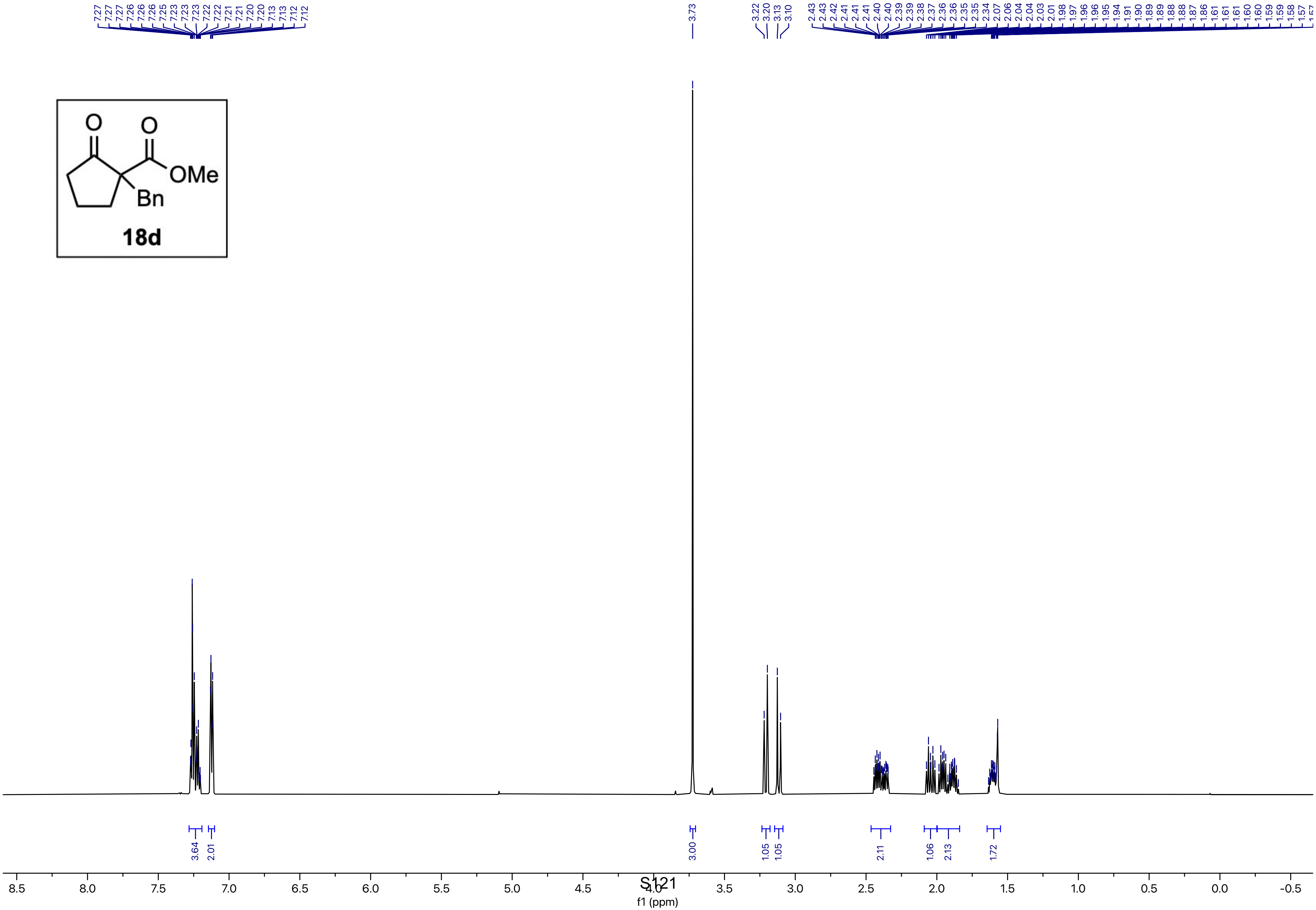
O
O
OMe
Bn
18d
3.64
2.01
3.00
1.05
1.05
2.11
1.06
2.13
1.72
f1 (ppm)

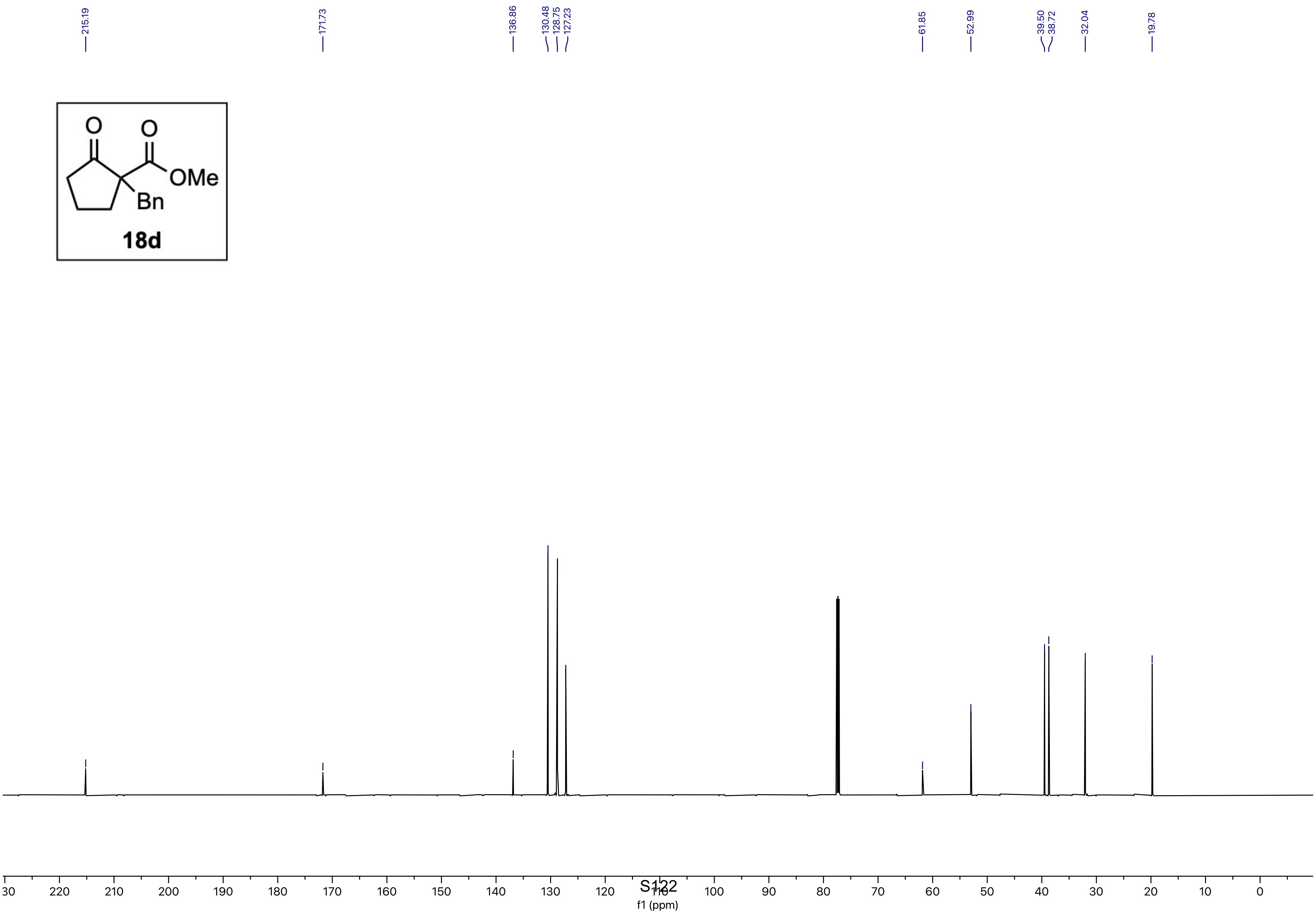
215.19
171.73
136.86
130.48
128.75
127.23
61.85
52.99
39.50
38.72
32.04
19.78
O
O
OMe
Bn
18d
30
220
210
200
190
180
170
160
150
140
130
120
100
90
80
70
60
50
40
30
20
10
0
f1 (ppm)

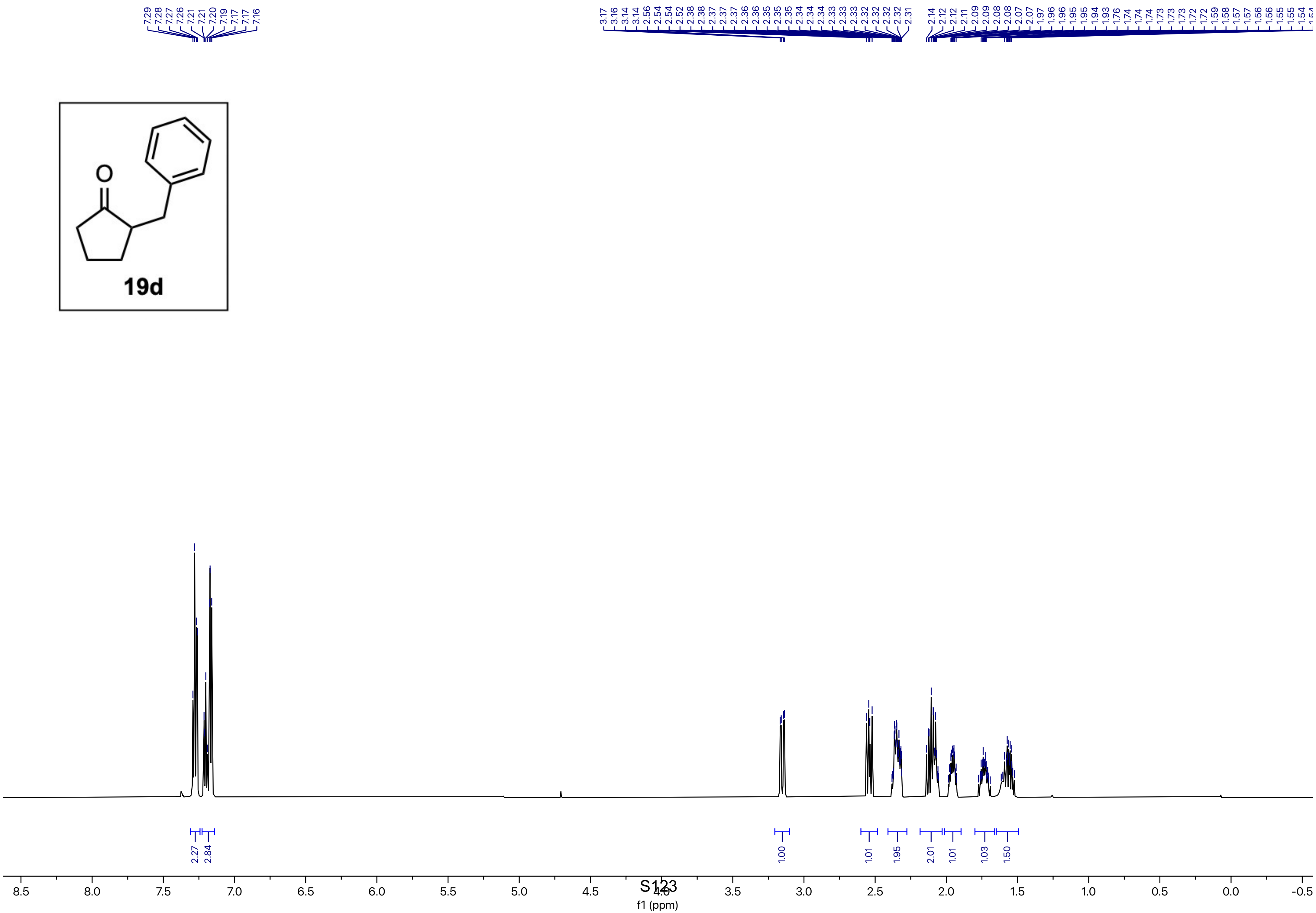

7.29 7.28 7.27 7.26 7.21 7.21 7.20 7.19 7.17 7.17 7.16
3.17 3.16 3.14 2.56 2.54 2.52 2.38 2.38 2.37 2.37 2.37 2.36 2.36 2.35 2.35 2.35 2.34 2.34 2.34 2.33 2.33 2.33 2.32 2.32 2.32 2.32 2.31
2.14 2.12 2.12 2.11 2.09 2.09 2.08 2.08 2.07 2.07 1.97 1.96 1.96 1.95 1.94 1.93 1.76 1.74 1.74 1.73 1.73 1.73 1.72 1.72 1.59 1.58 1.57 1.56 1.56 1.55 1.55 1.54
O
19d
2.27 2.84 1.00 1.01 1.95 2.01 1.01 1.03 1.50
8.5 8.0 7.5 7.0 6.5 6.0 5.5 5.0 4.5 4.0 3.5 3.0 2.5 2.0 1.5 1.0 0.5 0.0 -0.5
f1 (ppm)

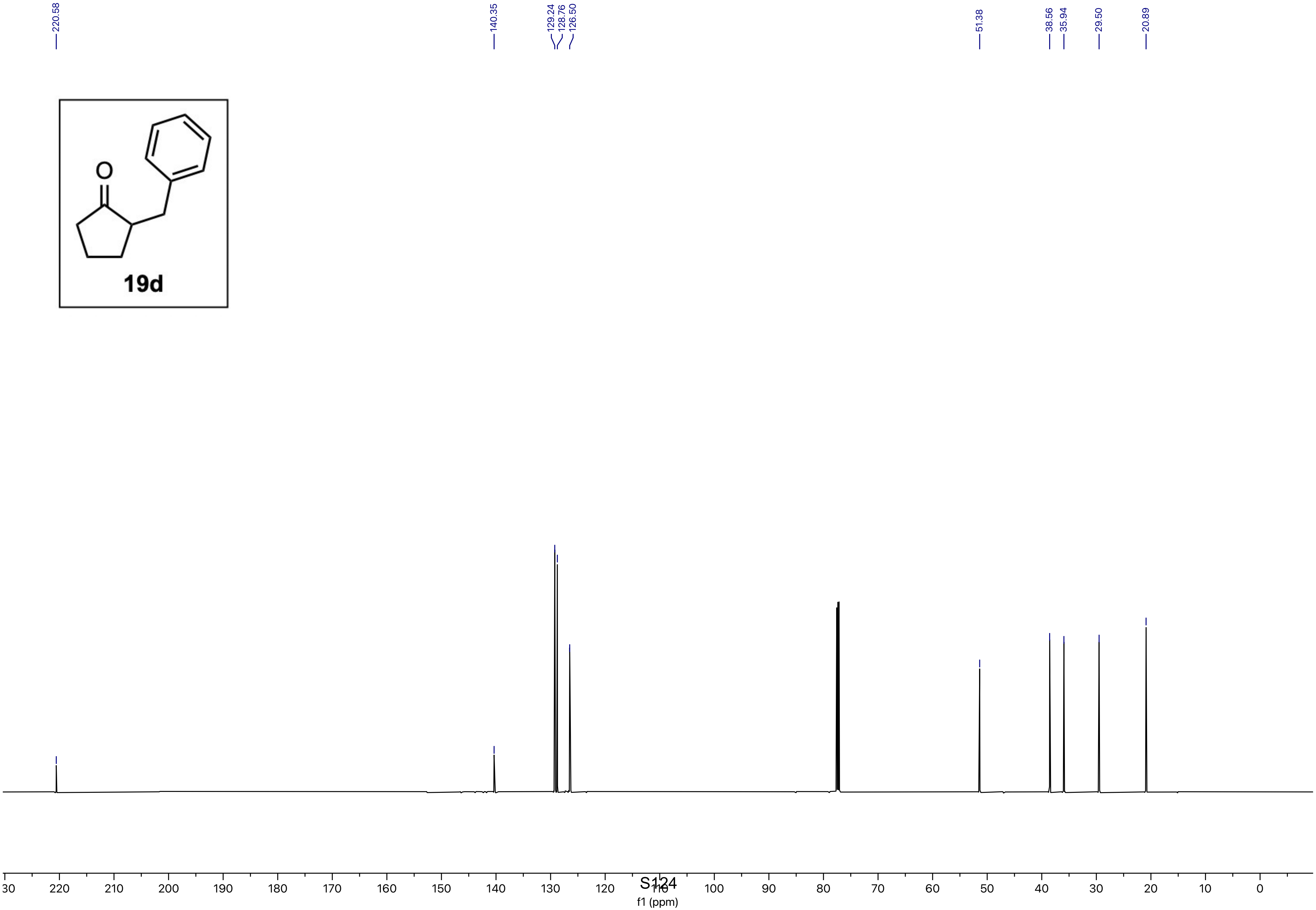
220.58
140.35
129.24
128.76
126.50
51.38
38.56
35.94
29.50
20.89
19d
30
220
210
200
190
180
170
160
150
140
130
120
110
100
90
80
70
60
50
40
30
20
10
0
f1 (ppm)

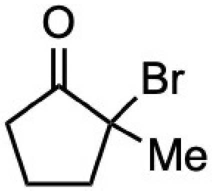
O
Br
Me

12a

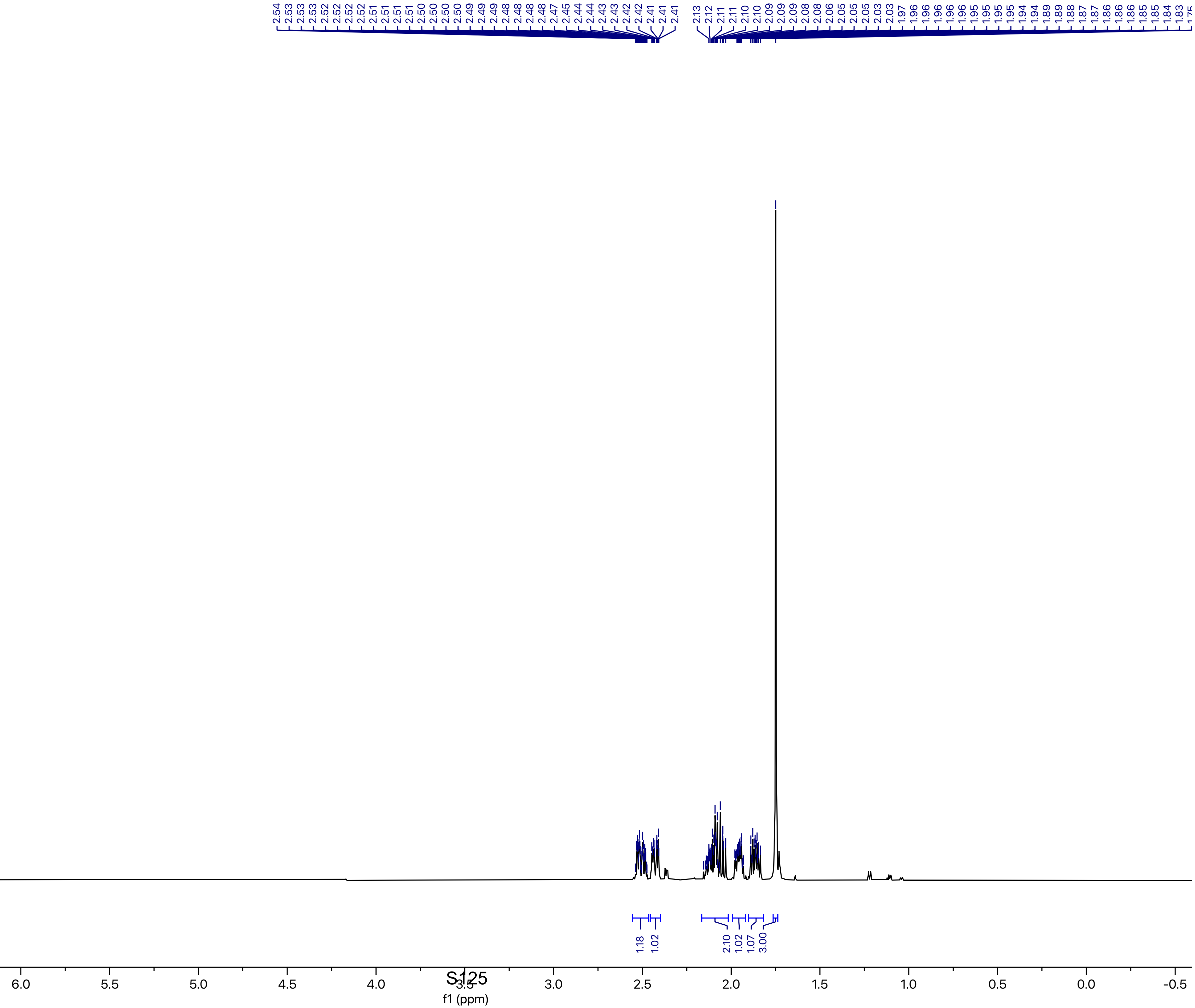
1.18
1.02
2.10
1.02
1.07
3.00
7.5
7.0
6.5
6.0
5.5
5.0
4.5
4.0
3.5
3.0
2.5
2.0
1.5
1.0
0.5
0.0
-0.5
f1 (ppm)

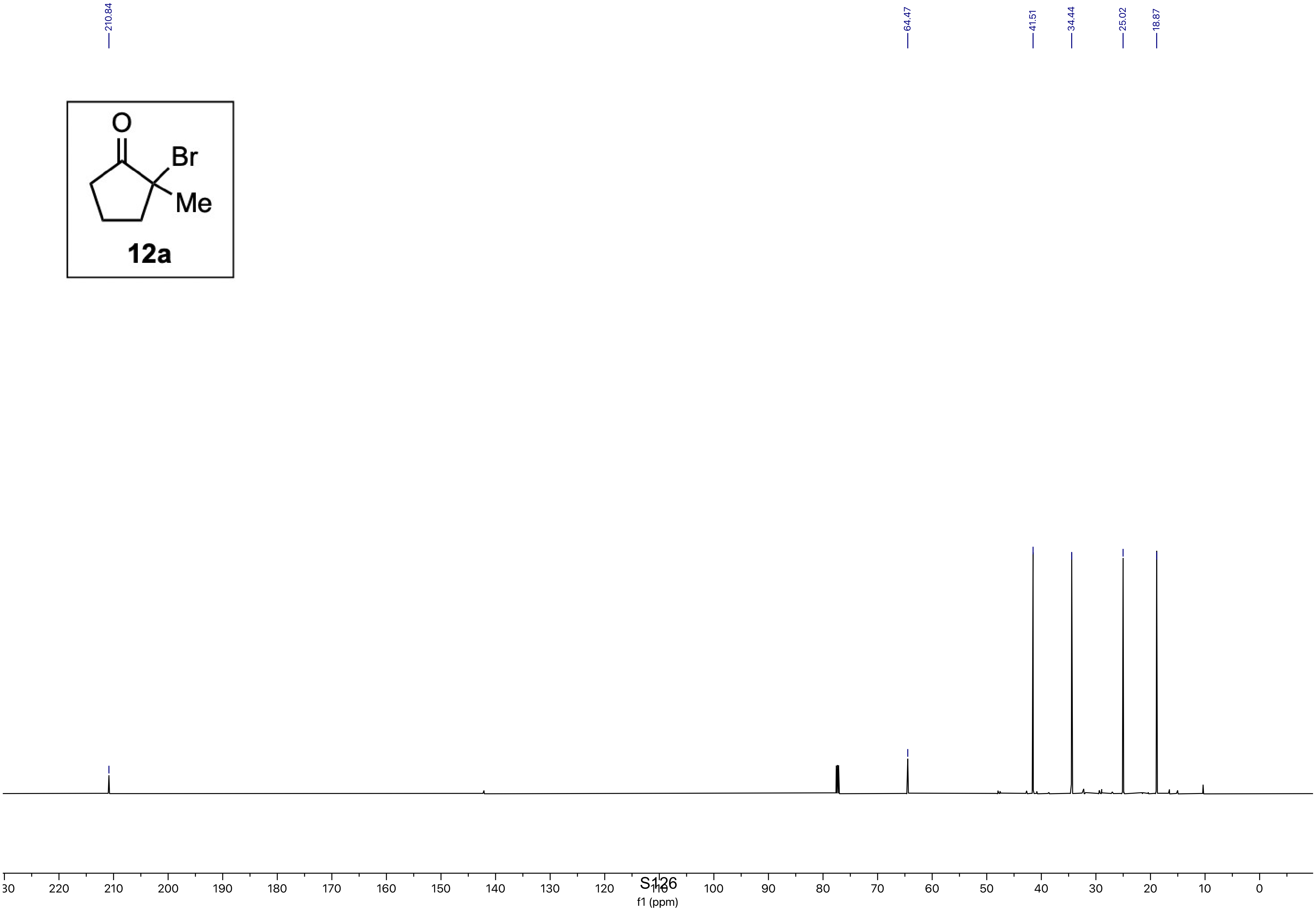
210.84
64.47
41.51
34.44
25.02
18.87
O
Br
Me
12a
30
220
210
200
190
180
170
160
150
140
130
120
110
100
90
80
70
60
50
40
30
20
10
0
f1 (ppm)

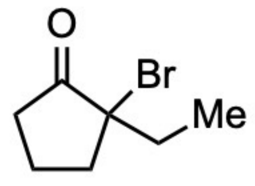
O
Br
Me

12b

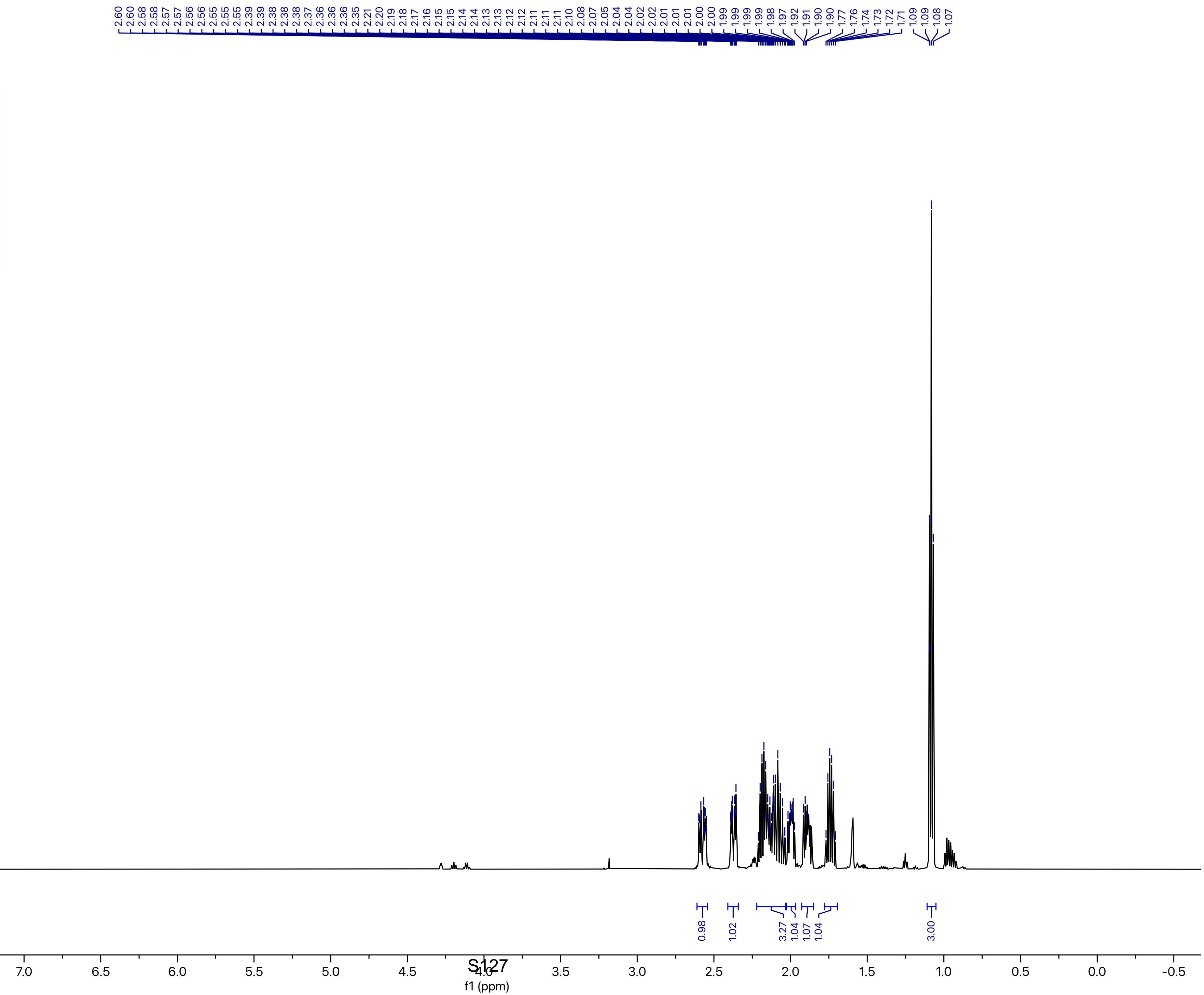
2.60 2.60 2.58 2.58 2.57 2.56 2.56 2.55 2.55 2.39 2.39 2.38 2.38 2.38 2.37 2.36 2.36 2.35 2.21 2.20 2.19 2.18 2.17 2.16 2.15 2.15 2.14 2.14 2.13 2.13 2.12 2.12 2.11 2.11 2.11 2.10 2.08 2.07 2.05 2.04 2.04 2.02 2.02 2.02 2.01 2.01 2.01 2.00 2.00 1.99 1.99 1.99 1.99 1.98 1.97 1.92 1.91 1.90 1.90 1.77 1.76 1.74 1.73 1.72 1.71 1.09 1.09 1.08 1.07
0.98
1.02
3.27
1.04
1.07
1.04
3.00
8.5 8.0 7.5 7.0 6.5 6.0 5.5 5.0 4.5 4.0 3.5 3.0 2.5 2.0 1.5 1.0 0.5 0.0 -0.5
f1 (ppm)

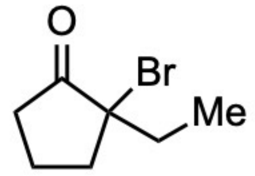
O
Br
Me

12b

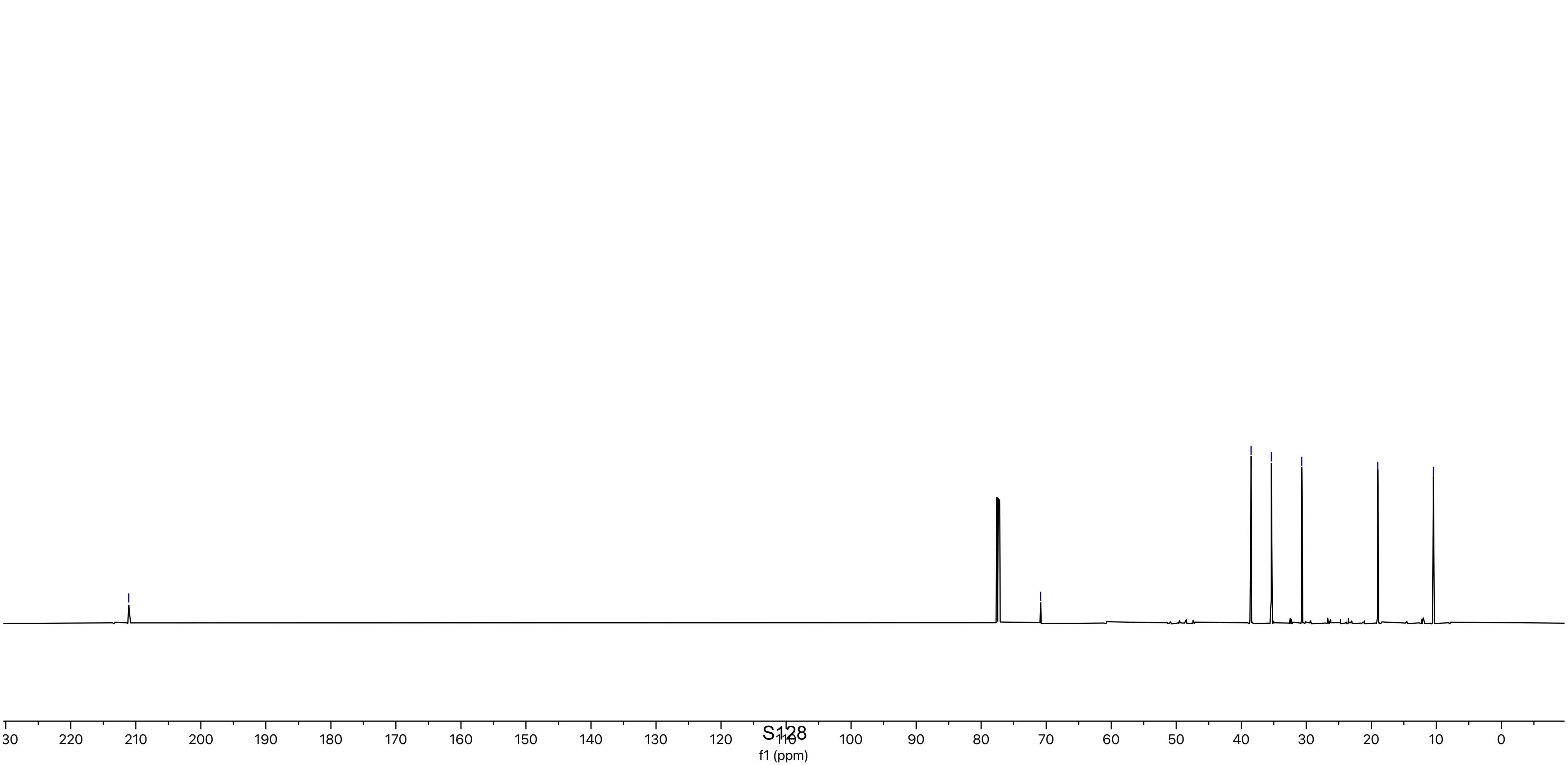
211.08
70.84
38.48
35.38
30.69
19.00
10.46
30
220
210
200
190
180
170
160
150
140
130
120
110
100
90
80
70
60
50
40
30
20
10
0
f1 (ppm)

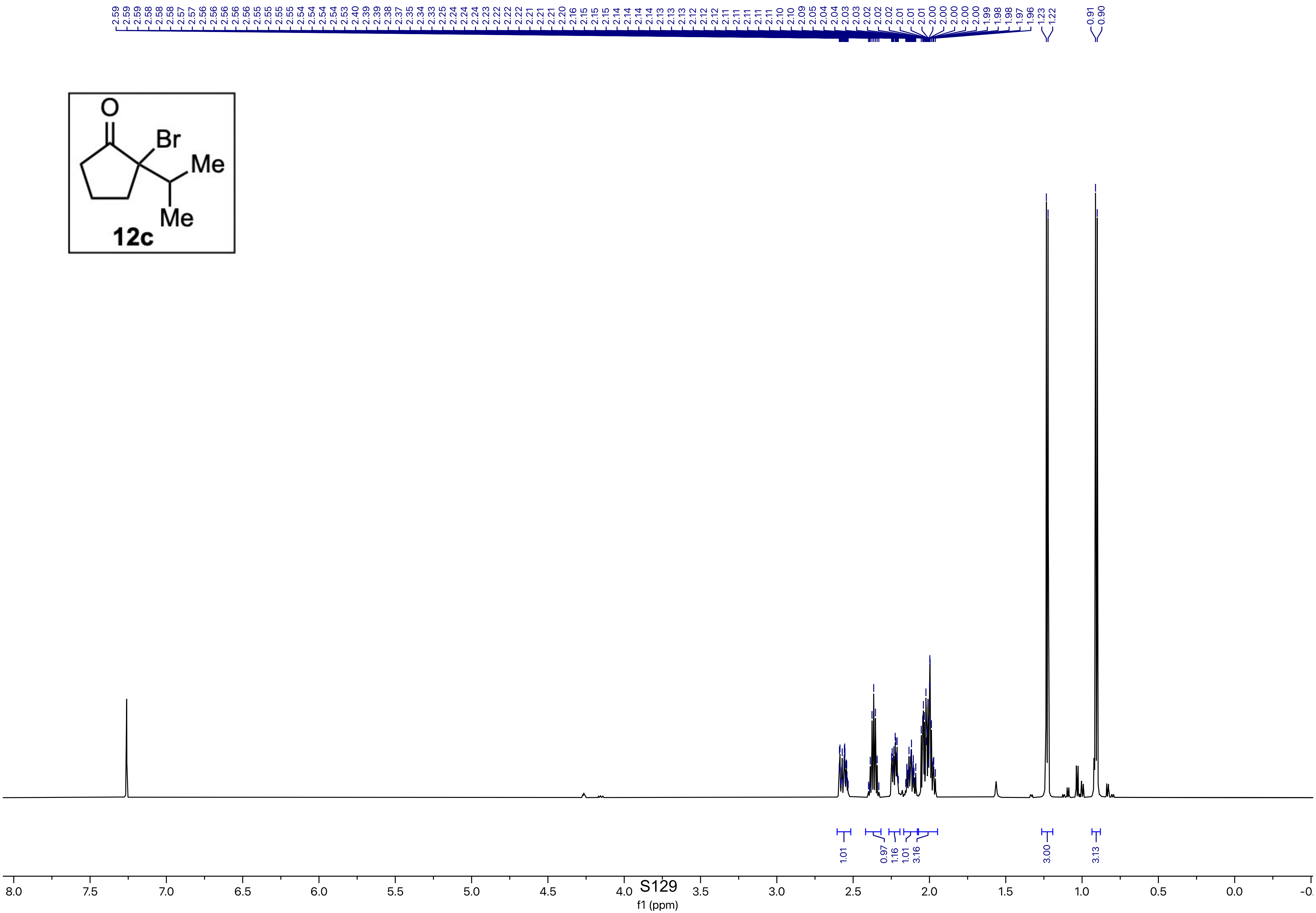
2.59 2.59 2.59 2.58 2.58 2.57 2.56 2.56 2.56 2.56 2.55 2.55 2.55 2.55 2.54 2.54 2.54 2.53 2.40 2.39 2.39 2.38 2.37 2.35 2.34 2.33 2.25 2.24 2.24 2.23 2.22 2.22 2.22 2.21 2.21 2.21 2.20 2.16 2.15 2.15 2.14 2.14 2.14 2.13 2.13 2.13 2.12 2.12 2.12 2.11 2.11 2.11 2.11 2.11 2.10 2.10 2.09 2.05 2.04 2.04 2.03 2.03 2.02 2.02 2.02 2.01 2.01 2.01 2.00 2.00 2.00 2.00 2.00 1.99 1.98 1.98 1.97 1.96 1.23 1.22 0.91 0.90
O
Br
Me
Me
12c
1.01
0.97
1.16
1.01
3.16
3.00
3.13
8.0 7.5 7.0 6.5 6.0 5.5 5.0 4.5 4.0 3.5 3.0 2.5 2.0 1.5 1.0 0.5 0.0
f1 (ppm)

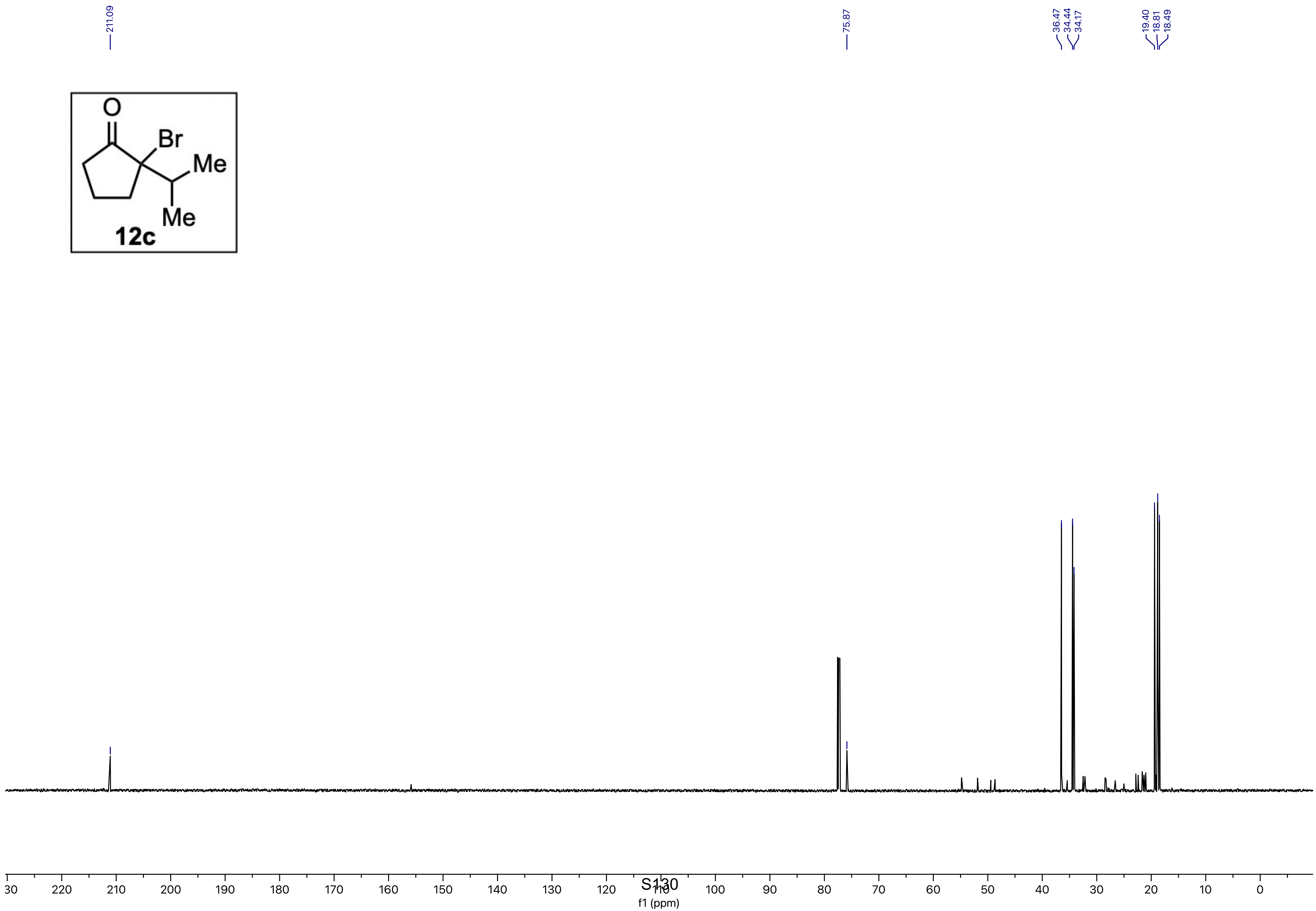

211.09
75.87
36.47
34.44
34.17
19.40
18.81
18.49
O
Br
Me
Me
12c
30
220
210
200
190
180
170
160
150
140
130
120
110
100
90
80
70
60
50
40
30
20
10
0
f1 (ppm)

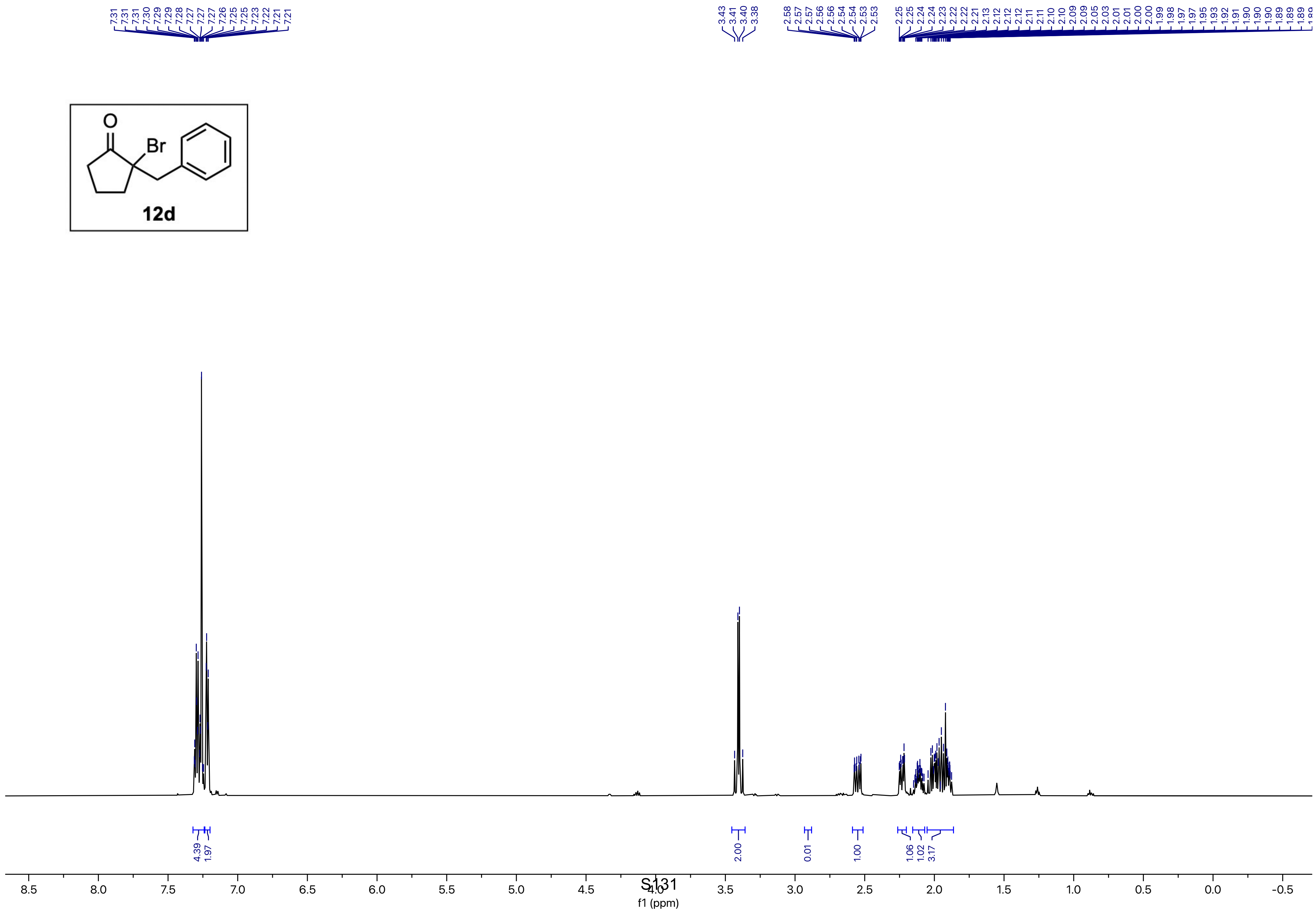
7.31
7.31
7.31
7.30
7.29
7.29
7.28
7.27
7.27
7.27
7.26
7.25
7.25
7.23
7.22
7.21
7.21
3.43
3.41
3.40
3.38
2.58
2.57
2.57
2.56
2.56
2.54
2.54
2.53
2.53
2.25
2.25
2.24
2.24
2.23
2.22
2.22
2.21
2.13
2.12
2.12
2.11
2.11
2.10
2.10
2.09
2.09
2.05
2.03
2.01
2.01
2.00
2.00
1.99
1.98
1.97
1.97
1.95
1.93
1.92
1.91
1.90
1.90
1.89
1.89
O
Br
12d
4.39
1.97
2.00
0.01
1.00
1.06
1.02
3.17
8.5
8.0
7.5
7.0
6.5
6.0
5.5
5.0
4.5
4.0
3.5
3.0
2.5
2.0
1.5
1.0
0.5
0.0
-0.5
f1 (ppm)

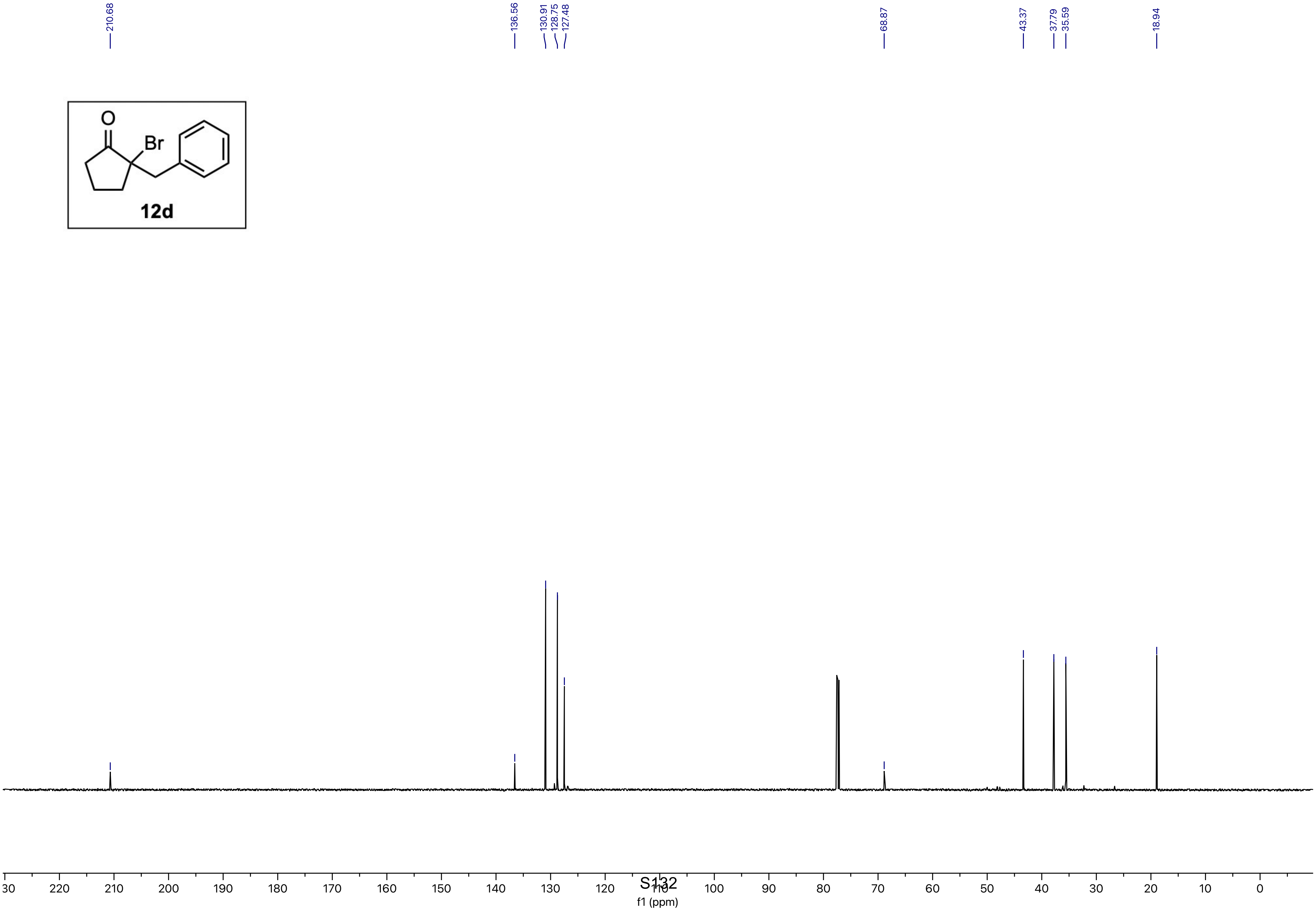

210.68
136.56
130.91
128.75
127.48
68.87
43.37
37.79
35.59
18.94
O
Br
12d
30 220 210 200 190 180 170 160 150 140 130 120 110 100 90 80 70 60 50 40 30 20 10 0
f1 (ppm)

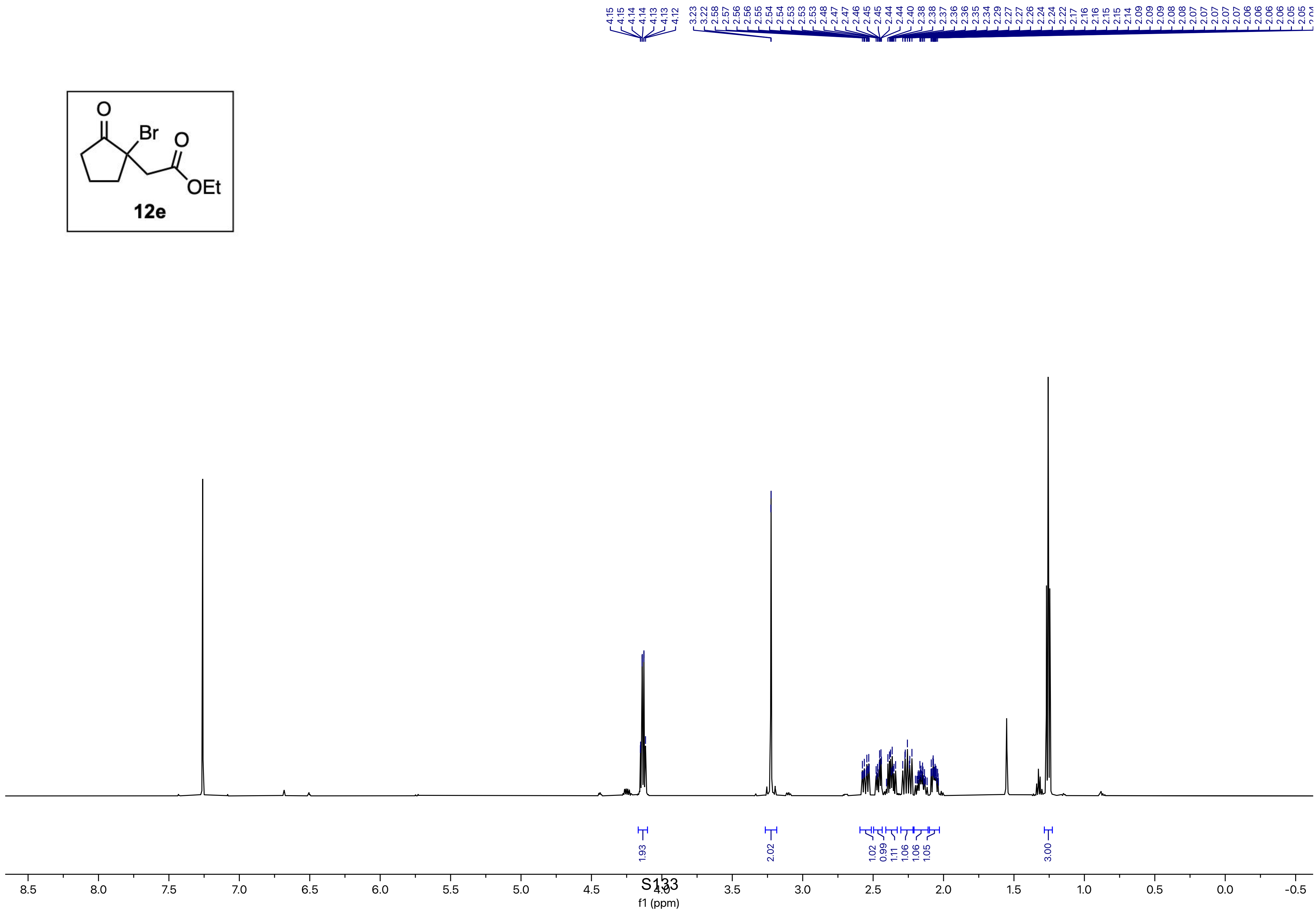

O
Br
O
OEt
12e
f1 (ppm)

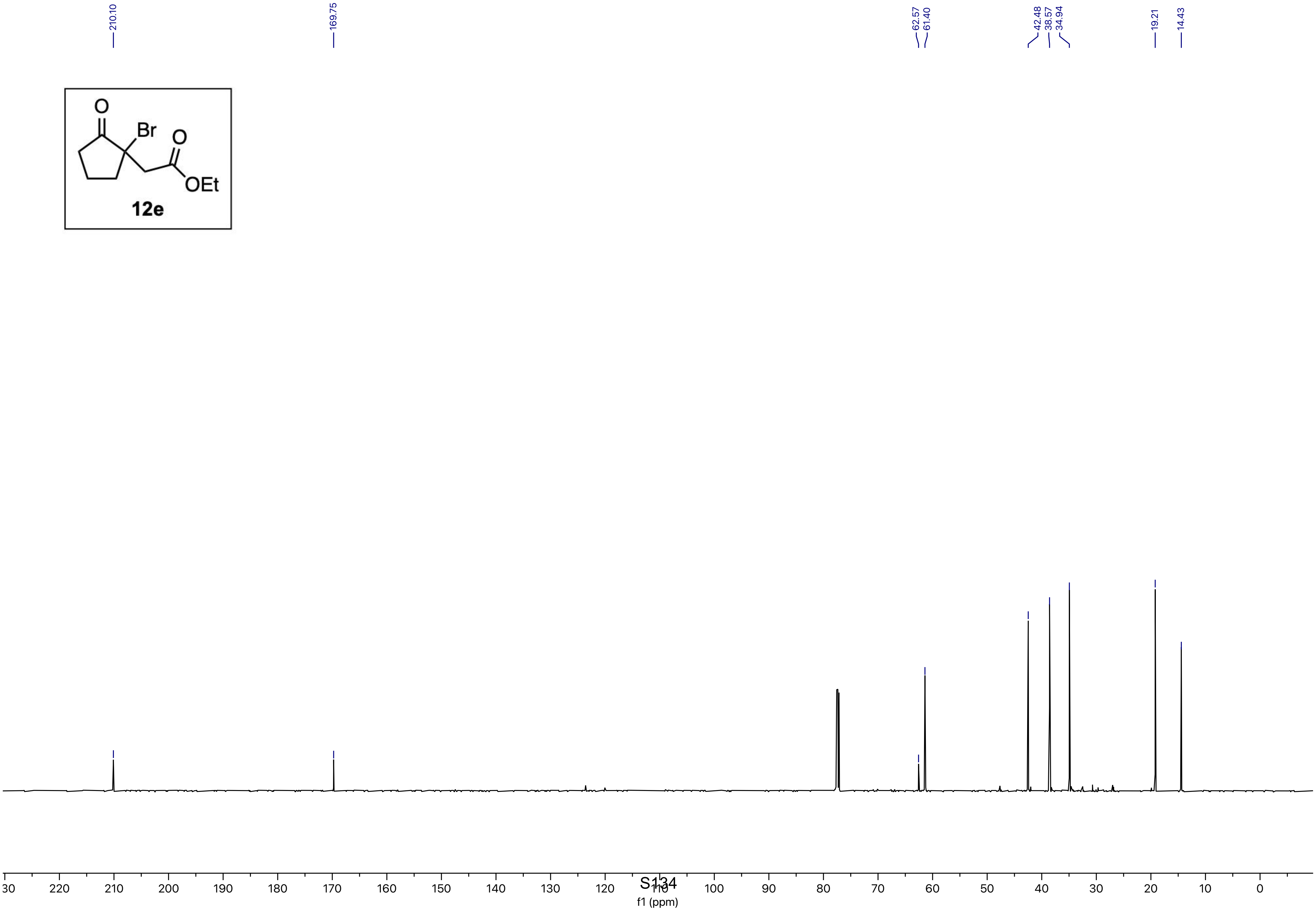
210.10
169.75
62.57
61.40
42.48
38.57
34.94
19.21
14.43
Br
O
OEt
12e
f1 (ppm)

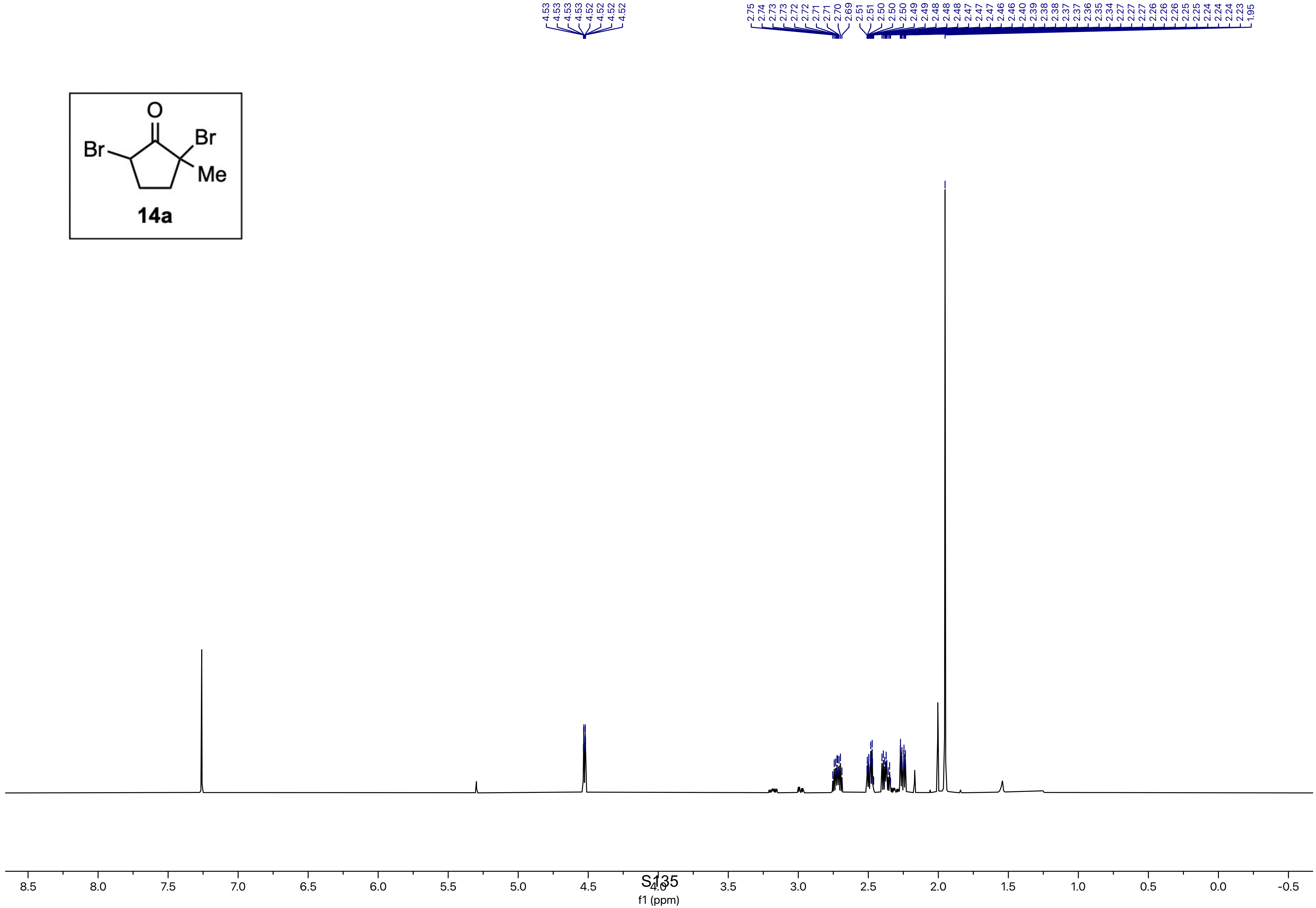
4.53 4.53 4.53 4.53 4.52 4.52 4.52 4.52
2.75 2.74 2.73 2.73 2.72 2.72 2.71 2.71 2.70 2.69 2.51 2.51 2.50 2.50 2.50 2.49 2.49 2.48 2.48 2.48 2.47 2.47 2.47 2.46 2.40 2.39 2.38 2.38 2.37 2.37 2.36 2.35 2.34 2.27 2.27 2.27 2.26 2.26 2.26 2.25 2.24 2.24 2.24 2.23 1.95
O
Br
Br
Me
14a
8.5 8.0 7.5 7.0 6.5 6.0 5.5 5.0 4.5 4.0 3.5 3.0 2.5 2.0 1.5 1.0 0.5 0.0 -0.5
f1 (ppm)

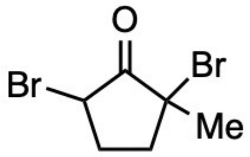
O
Br
Br
Me

14a

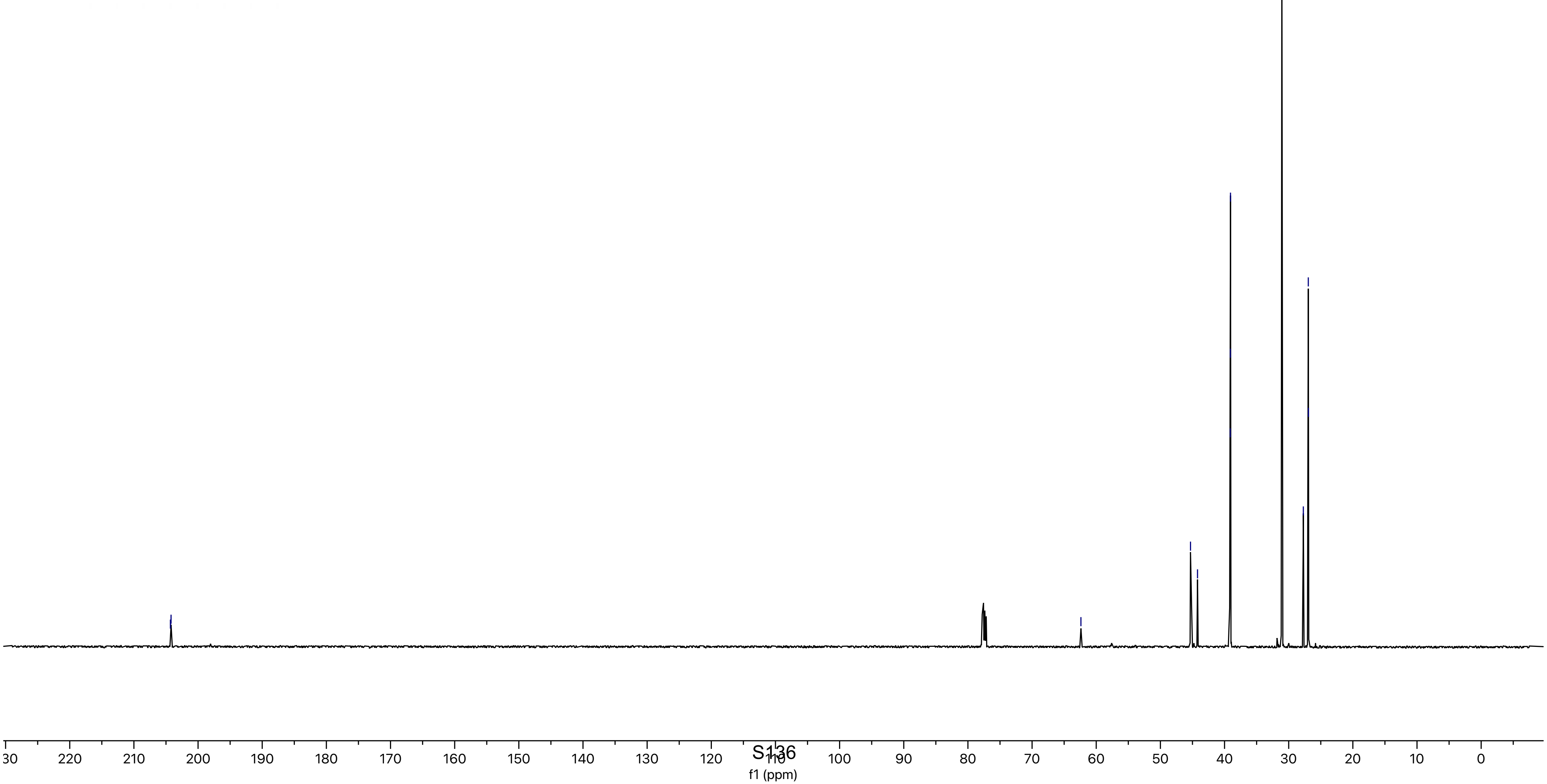
204.31
204.21
62.39
45.30
44.21
39.10
39.09
39.07
31.07
27.72
26.94
26.92
30 220 210 200 190 180 170 160 150 140 130 120 110 100 90 80 70 60 50 40 30 20 10 0
f1 (ppm)

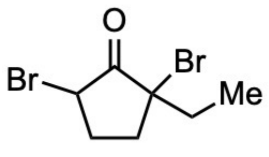
O
Br
Br
Me

14b

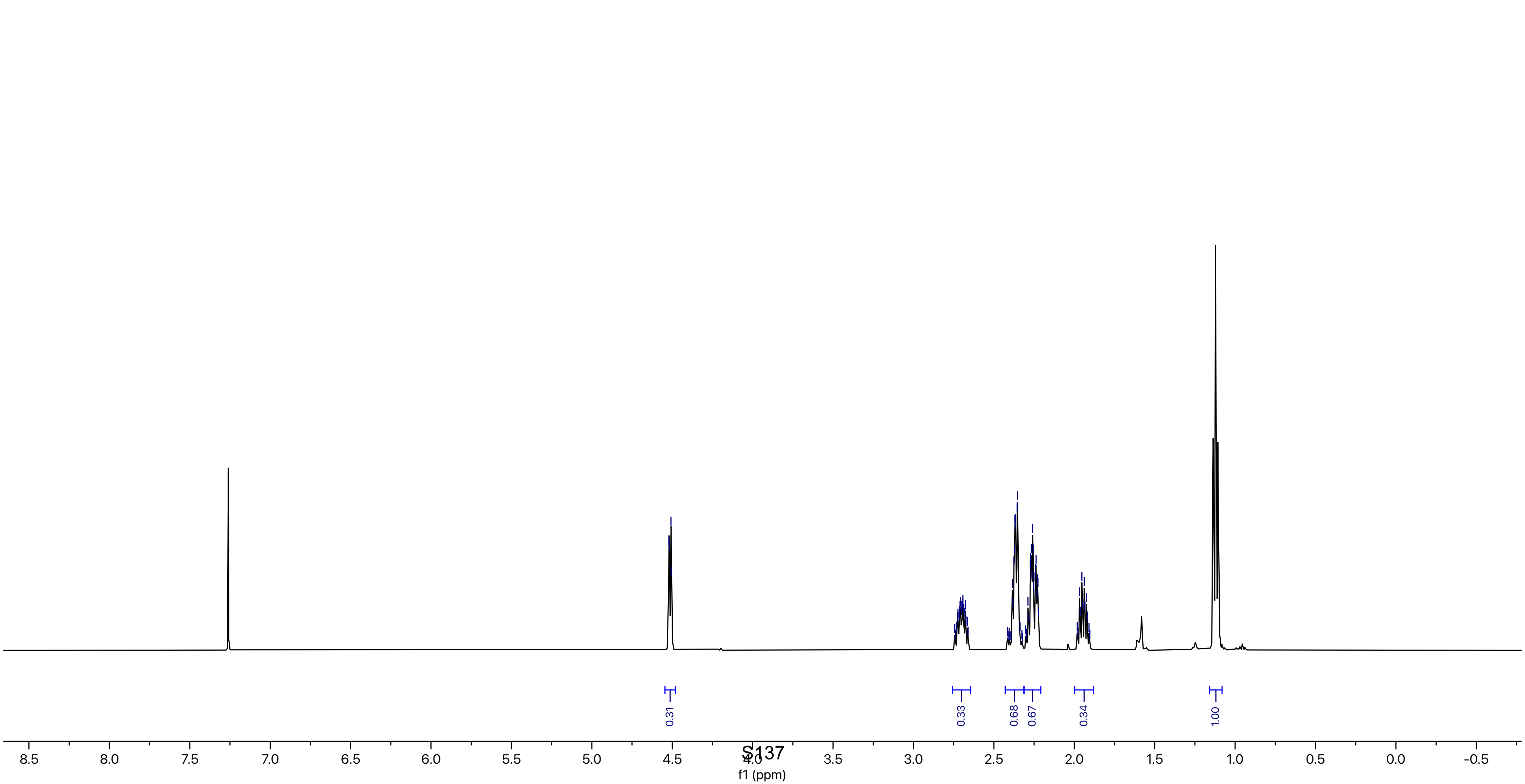
4.52
4.52
4.51
4.50
2.74
2.74
2.73
2.73
2.72
2.72
2.71
2.71
2.71
2.70
2.70
2.70
2.69
2.69
2.68
2.68
2.67
2.66
2.66
2.42
2.40
2.39
2.38
2.38
2.37
2.37
2.37
2.36
2.35
2.34
2.30
2.30
2.30
2.29
2.28
2.27
2.27
2.27
2.26
2.26
2.25
2.24
2.24
2.23
2.23
2.23
2.22
1.98
1.97
1.96
1.95
1.95
1.94
1.93
1.92
1.92
0.31
0.33
0.68
0.67
0.34
1.00
8.5
8.0
7.5
7.0
6.5
6.0
5.5
5.0
4.5
4.0
3.5
3.0
2.5
2.0
1.5
1.0
0.5
0.0
-0.5
f1 (ppm)

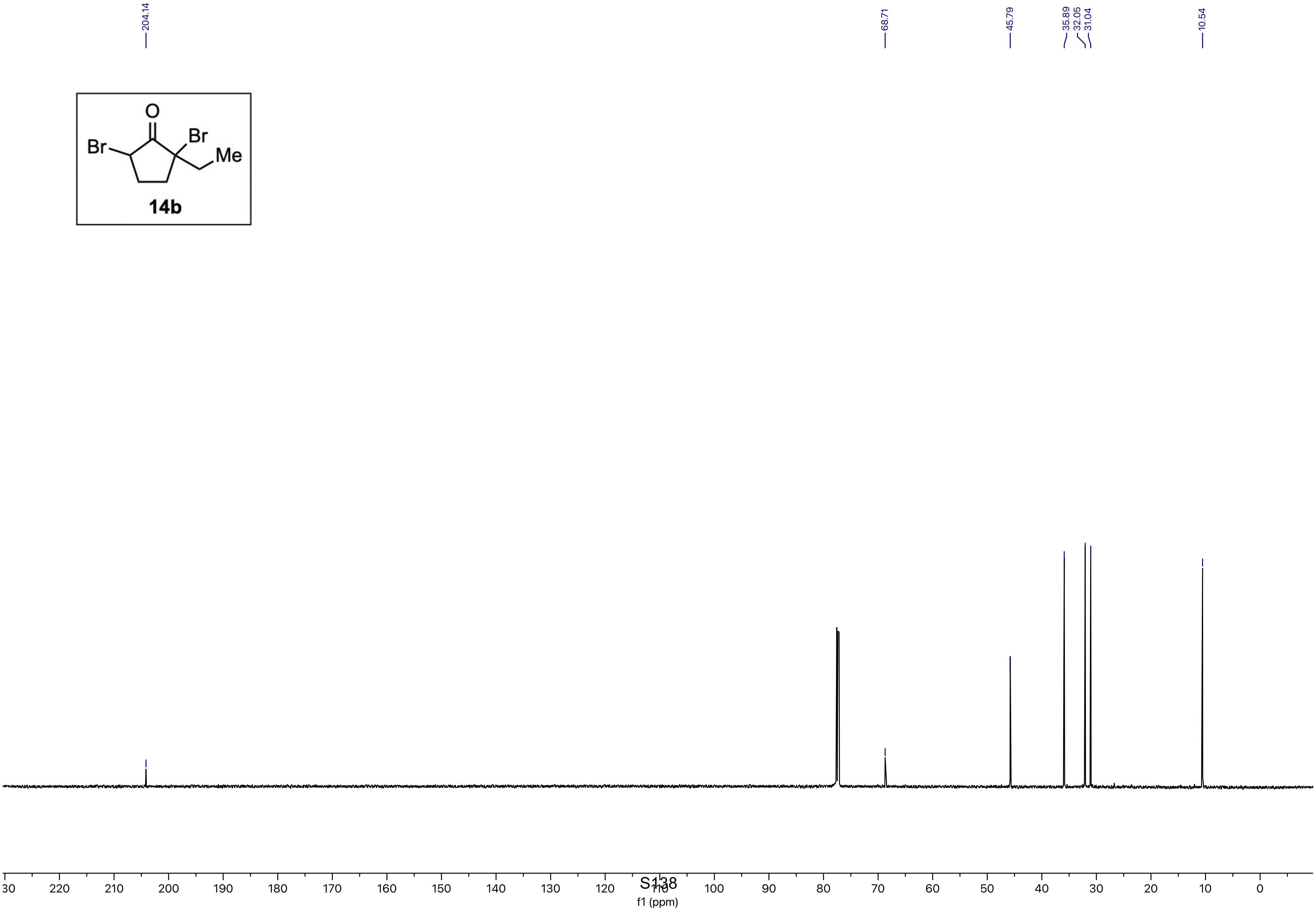
204.14
68.71
45.79
35.89
32.05
31.04
10.54
O
Br
Br
Me
14b
30 220 210 200 190 180 170 160 150 140 130 120 110 100 90 80 70 60 50 40 30 20 10 0
f1 (ppm)

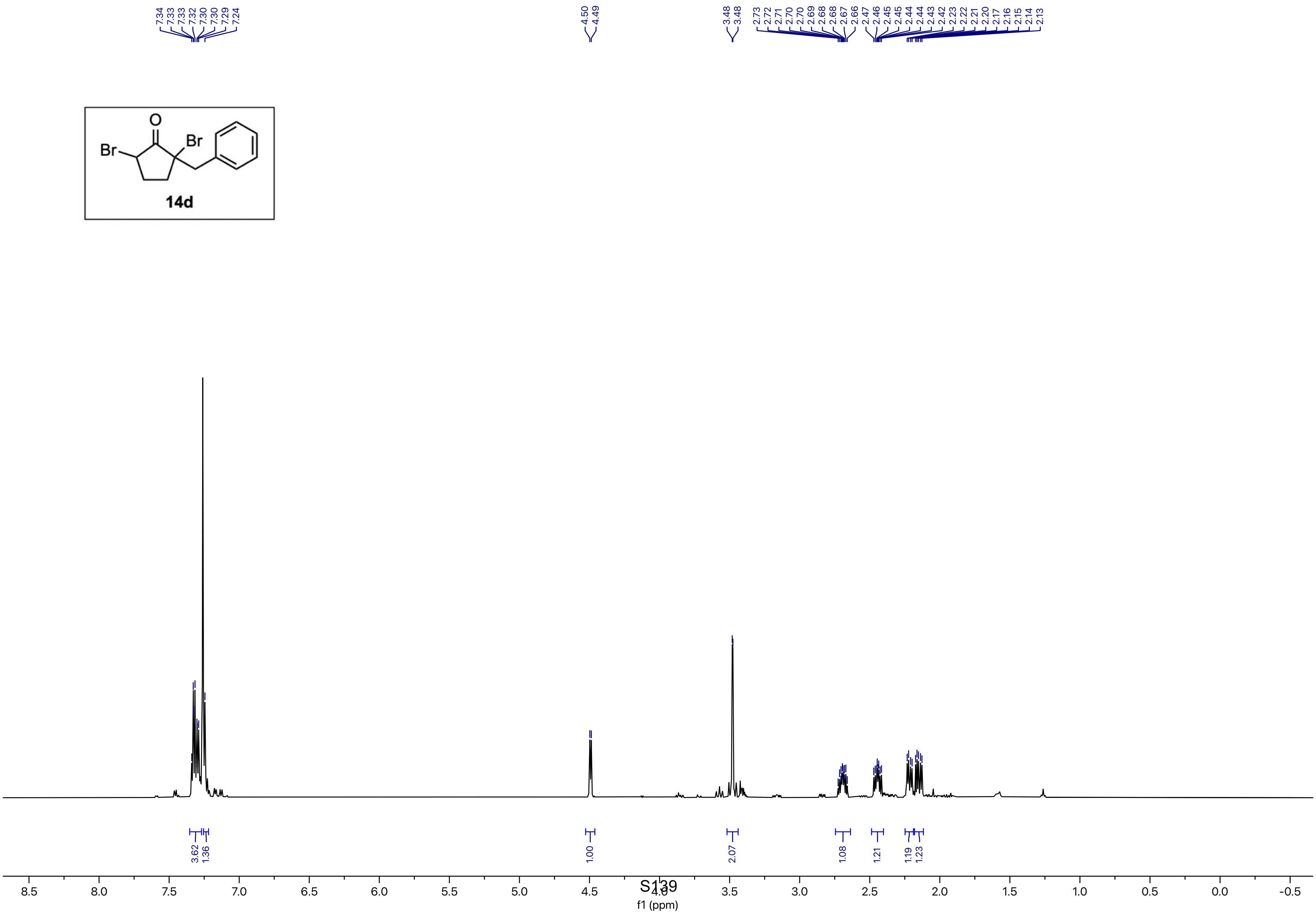
7.34
7.33
7.33
7.32
7.30
7.30
7.29
7.24
4.50
4.49
3.48
3.48
2.73
2.72
2.71
2.70
2.70
2.69
2.68
2.68
2.67
2.66
2.47
2.46
2.45
2.45
2.44
2.44
2.43
2.42
2.23
2.22
2.21
2.20
2.17
2.16
2.15
2.14
2.13
O
Br
Br
14d
3.62
1.36
1.00
2.07
1.08
1.21
1.19
1.23
8.5
8.0
7.5
7.0
6.5
6.0
5.5
5.0
4.5
4.0
3.5
3.0
2.5
2.0
1.5
1.0
0.5
0.0
-0.5
f1 (ppm)

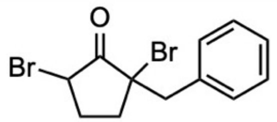
O
Br
Br

14d

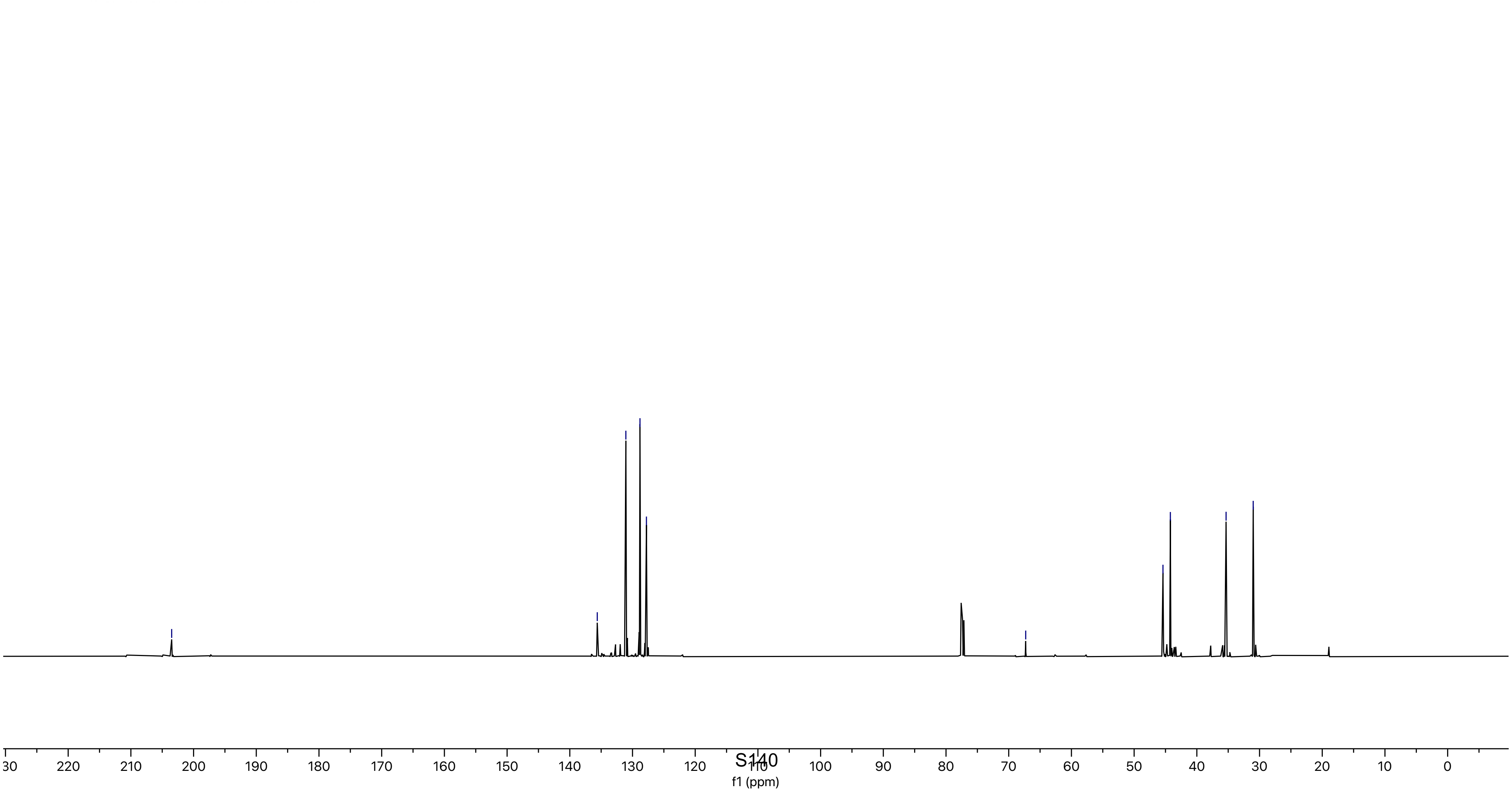
203.49
135.62
131.06
128.81
127.78
67.29
45.40
44.22
35.34
31.01
30
220
210
200
190
180
170
160
150
140
130
120
100
90
80
70
60
50
40
30
20
10
0
f1 (ppm)

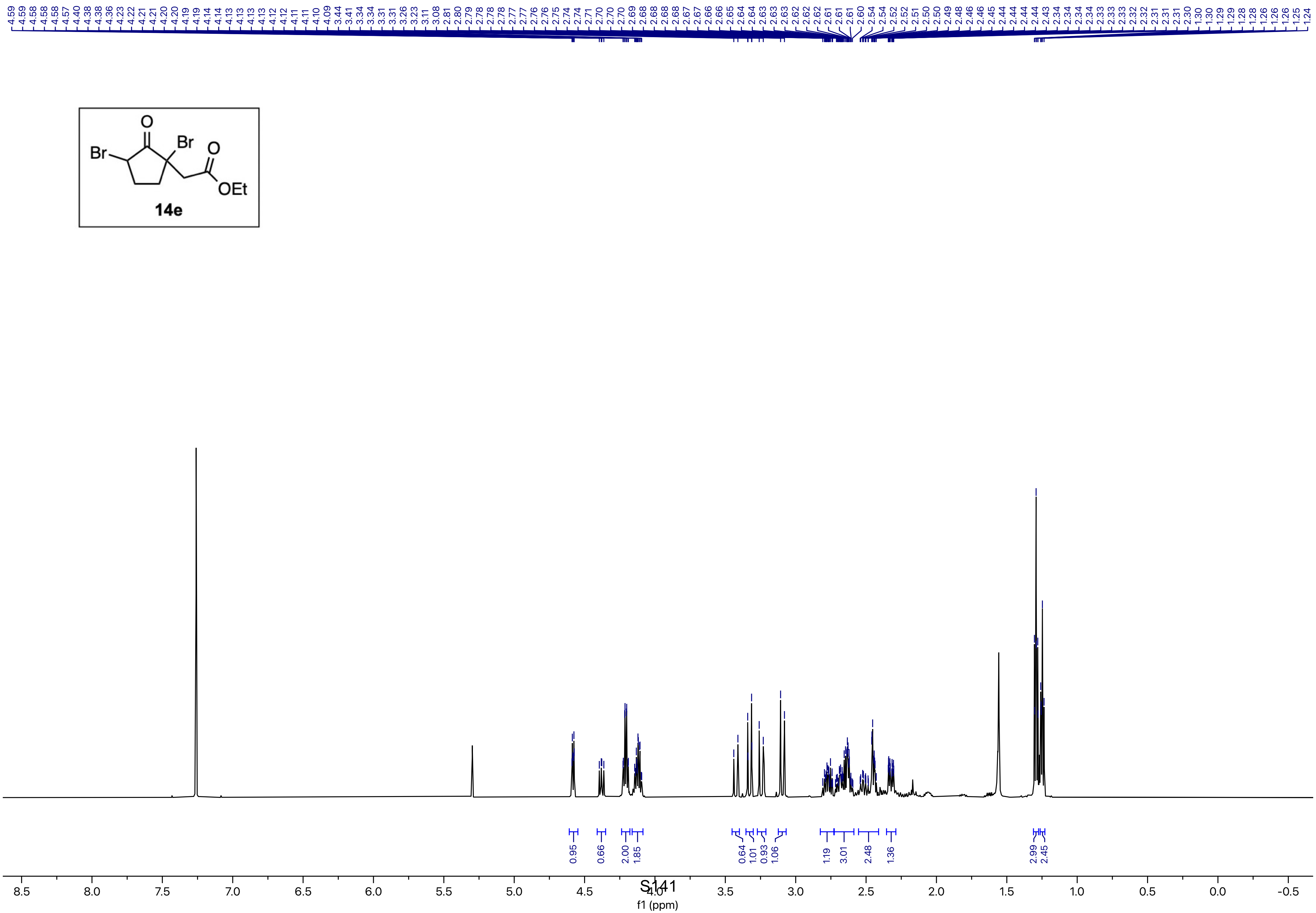

Br
O
Br
O
OEt
14e
f1 (ppm)

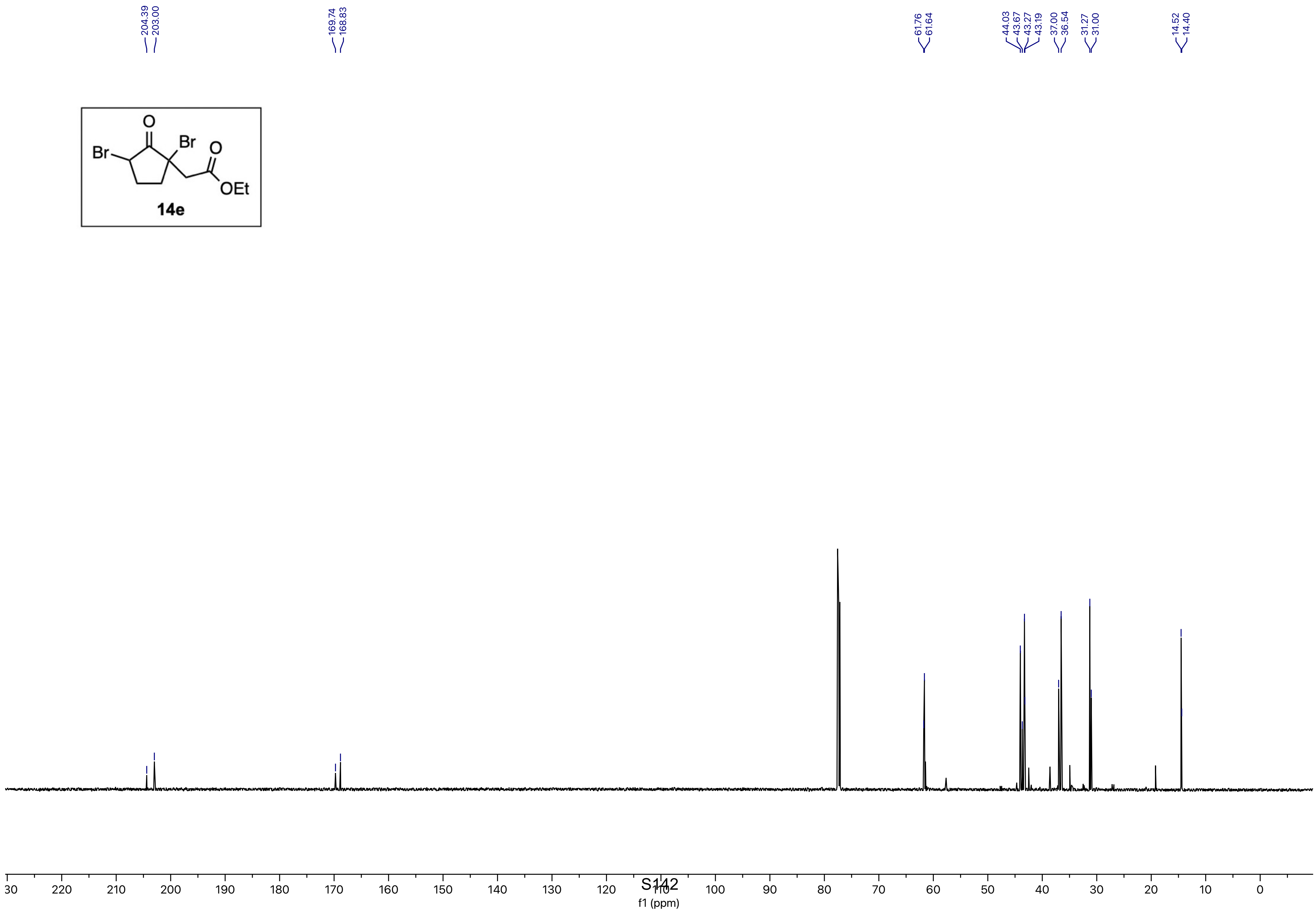
204.39
203.00
169.74
168.83
61.76
61.64
44.03
43.67
43.27
43.19
37.00
36.54
31.27
31.00
14.52
14.40
O
Br
Br
O
OEt
14e
f1 (ppm)

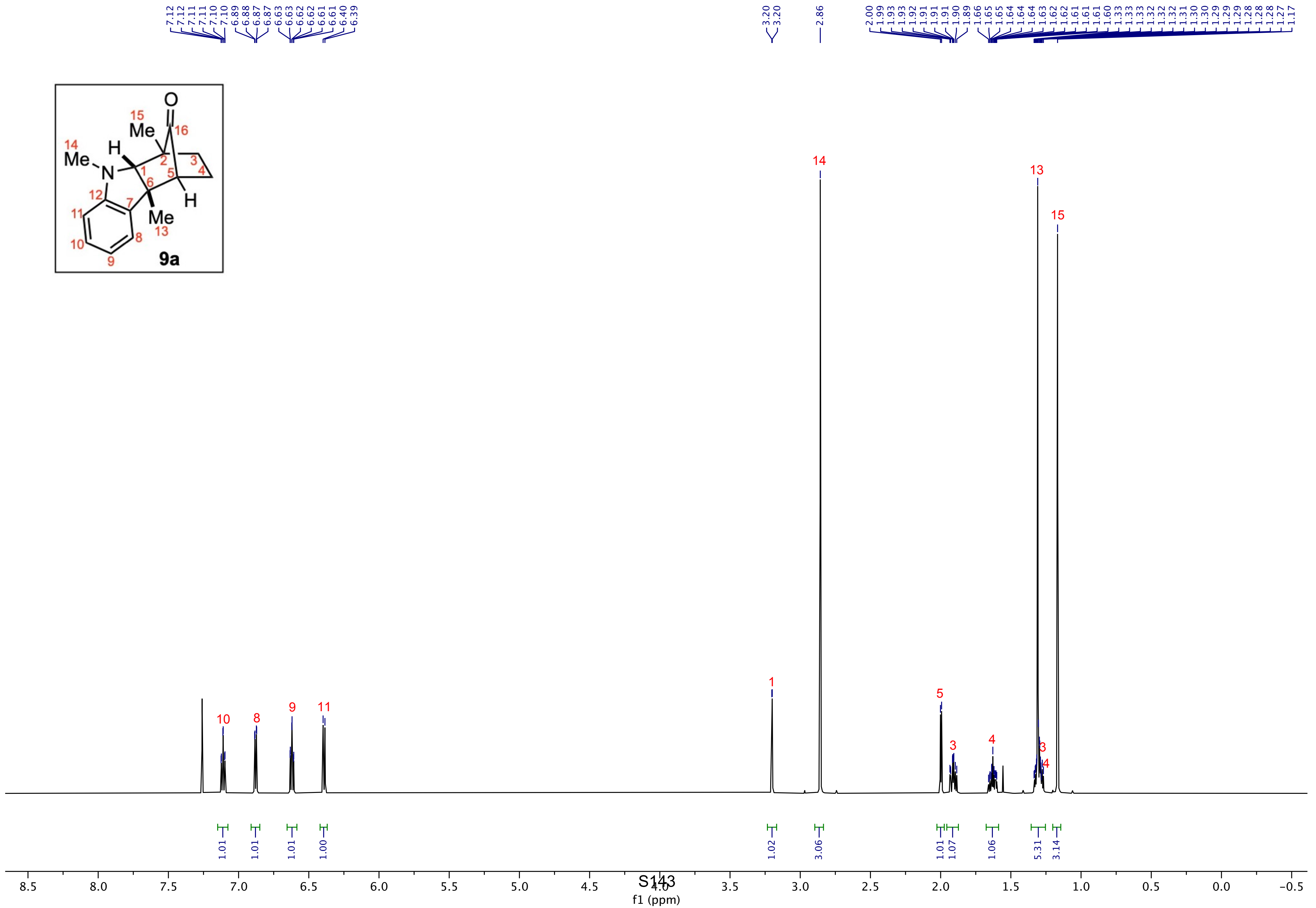
9a
14
1
5
3
4
13
15
10
8
9
11
1.01
1.01
1.01
1.00
1.02
3.06
1.01
1.07
1.06
5.31
3.14
8.5 8.0 7.5 7.0 6.5 6.0 5.5 5.0 4.5 4.0 3.5 3.0 2.5 2.0 1.5 1.0 0.5 0.0 −0.5
f1 (ppm)

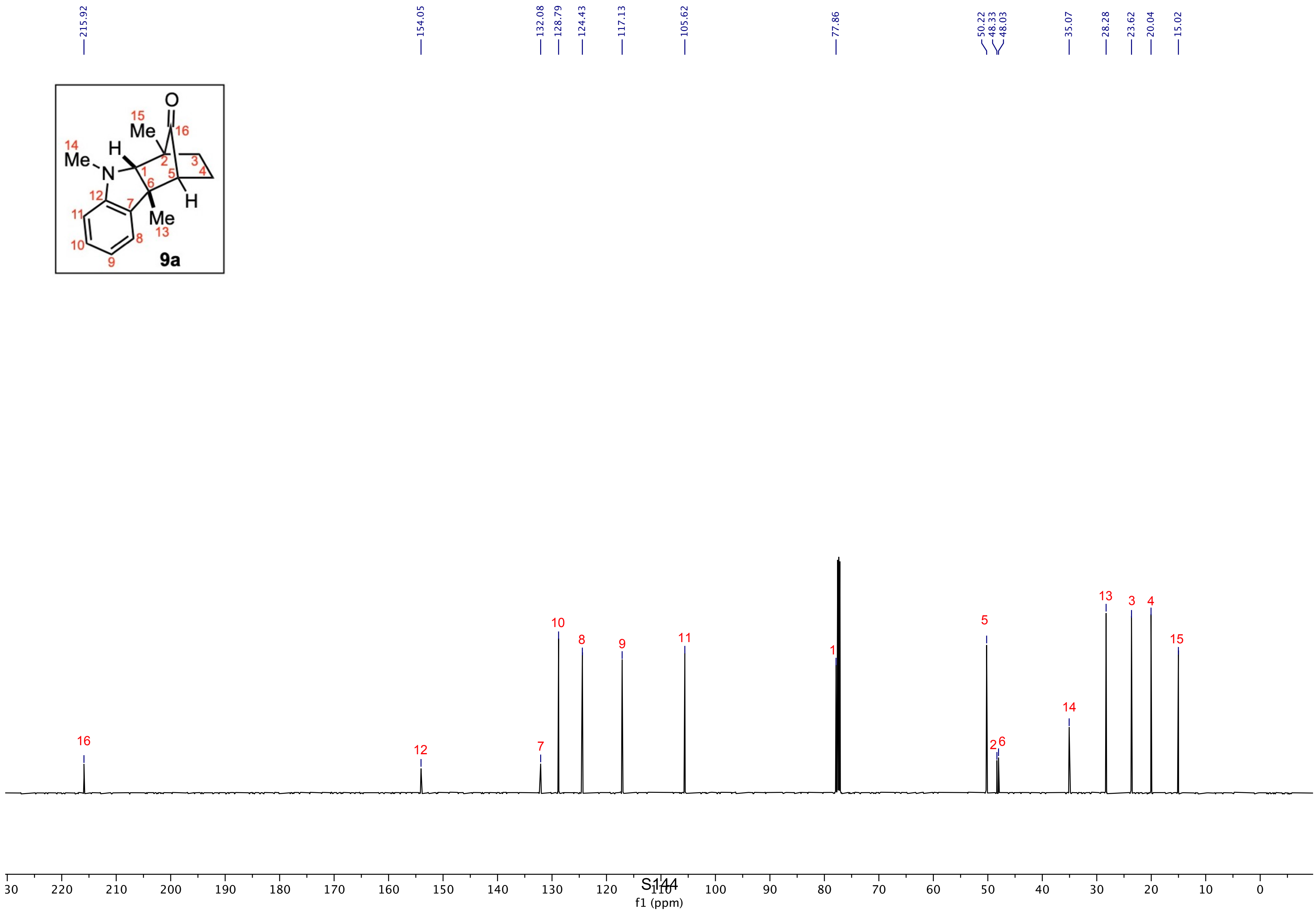
215.92
154.05
132.08
128.79
124.43
117.13
105.62
77.86
50.22
48.33
48.03
35.07
28.28
23.62
20.04
15.02
9a
f1 (ppm)

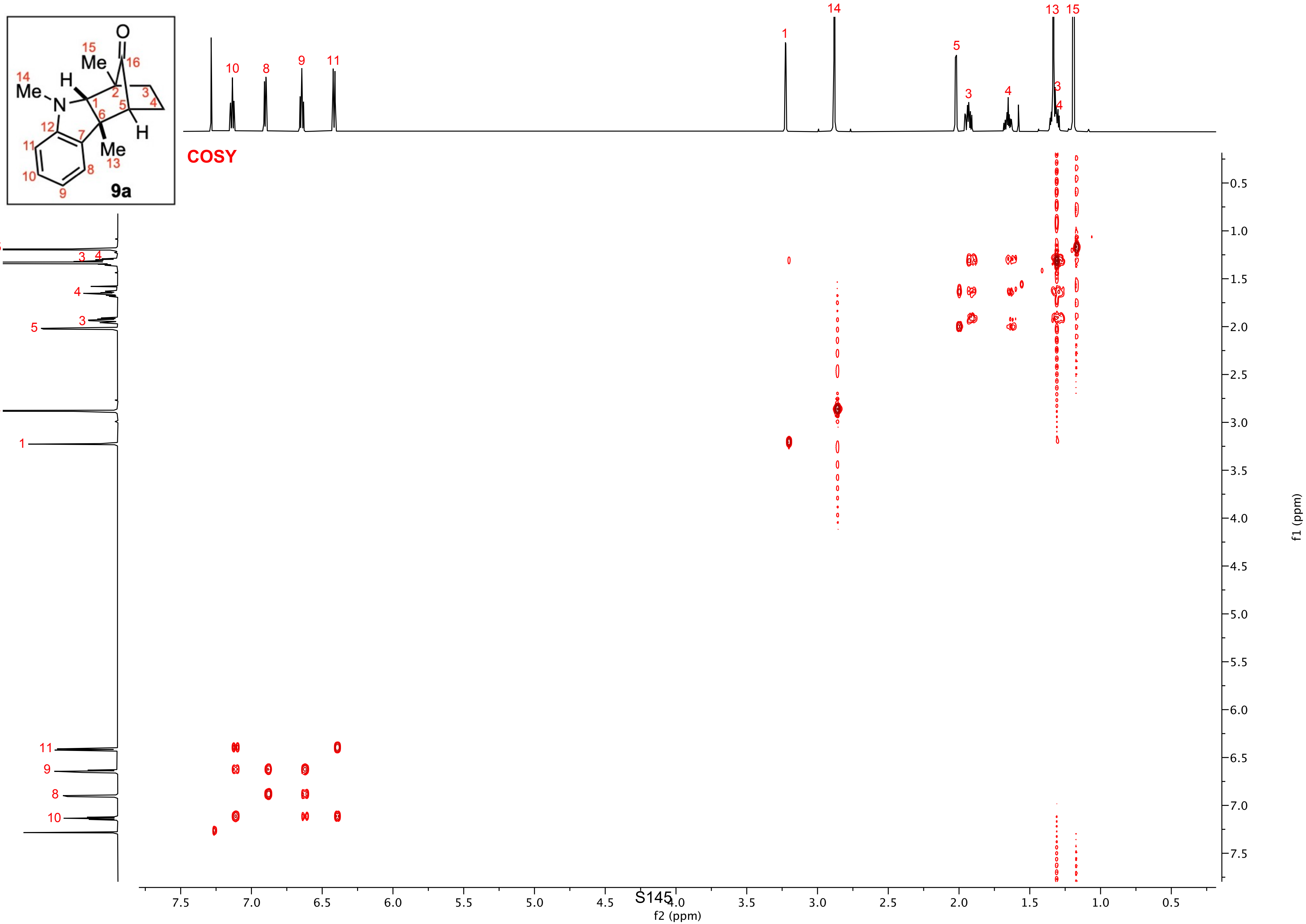

9a
COSY
f2 (ppm)
f1 (ppm)

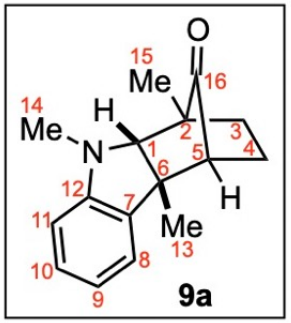

O
16
15
Me
14
Me
N
H
2
3
4
5
1
6
12
7
11
10
9
8
H
Me
13
9a

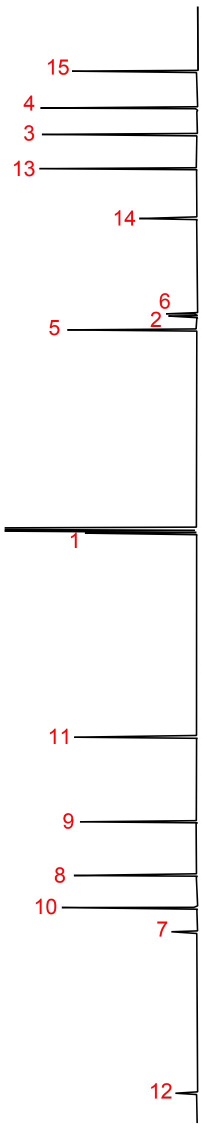

15
4
3
13
14
6
2
5
1
11
9
8
10
7
12

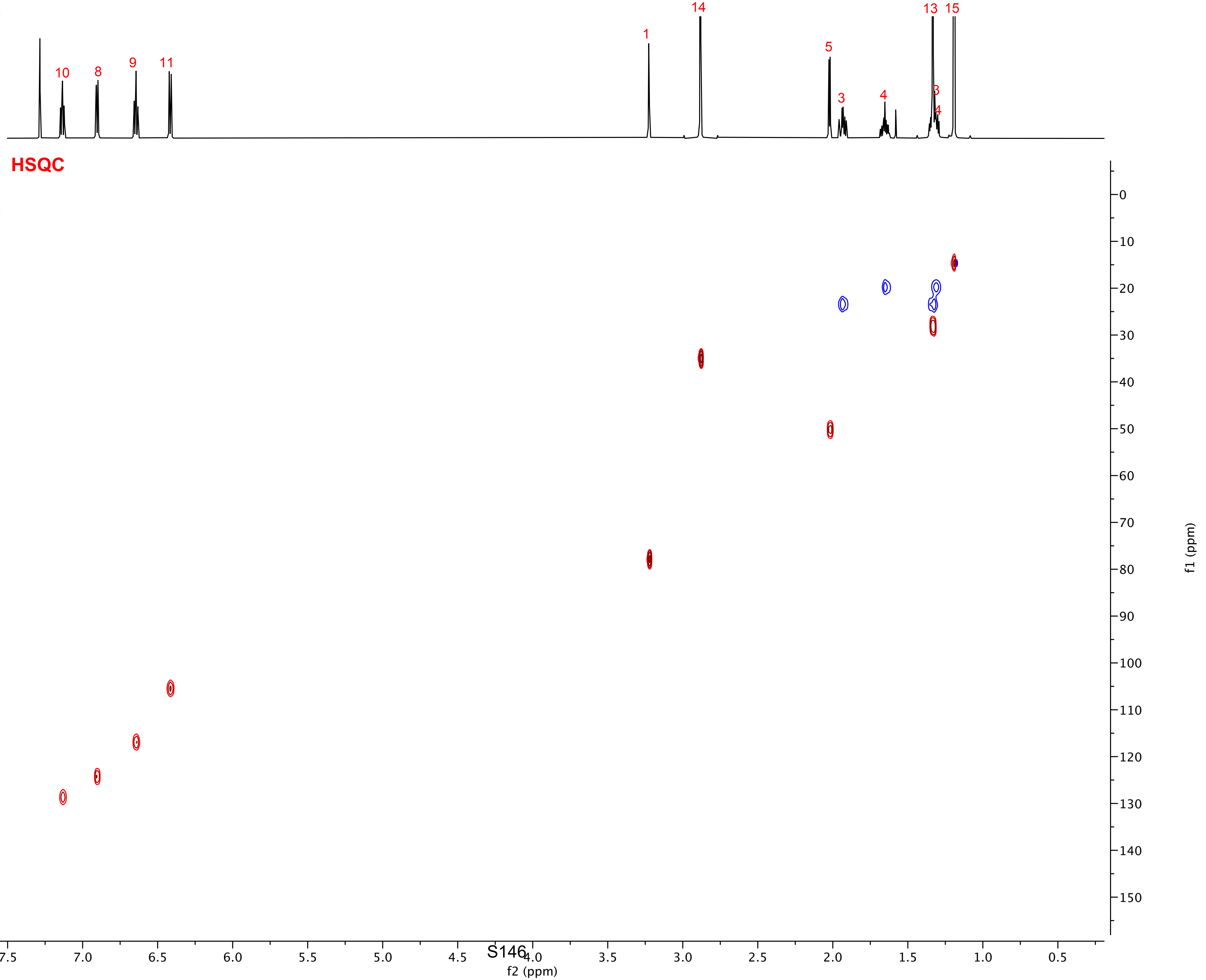

HSQC
10
8
9
11
1
14
5
3
4
13
15
3
4
f2 (ppm)
f1 (ppm)
7.5
7.0
6.5
6.0
5.5
5.0
4.5
4.0
3.5
3.0
2.5
2.0
1.5
1.0
0.5
0
10
20
30
40
50
60
70
80
90
100
110
120
130
140
150

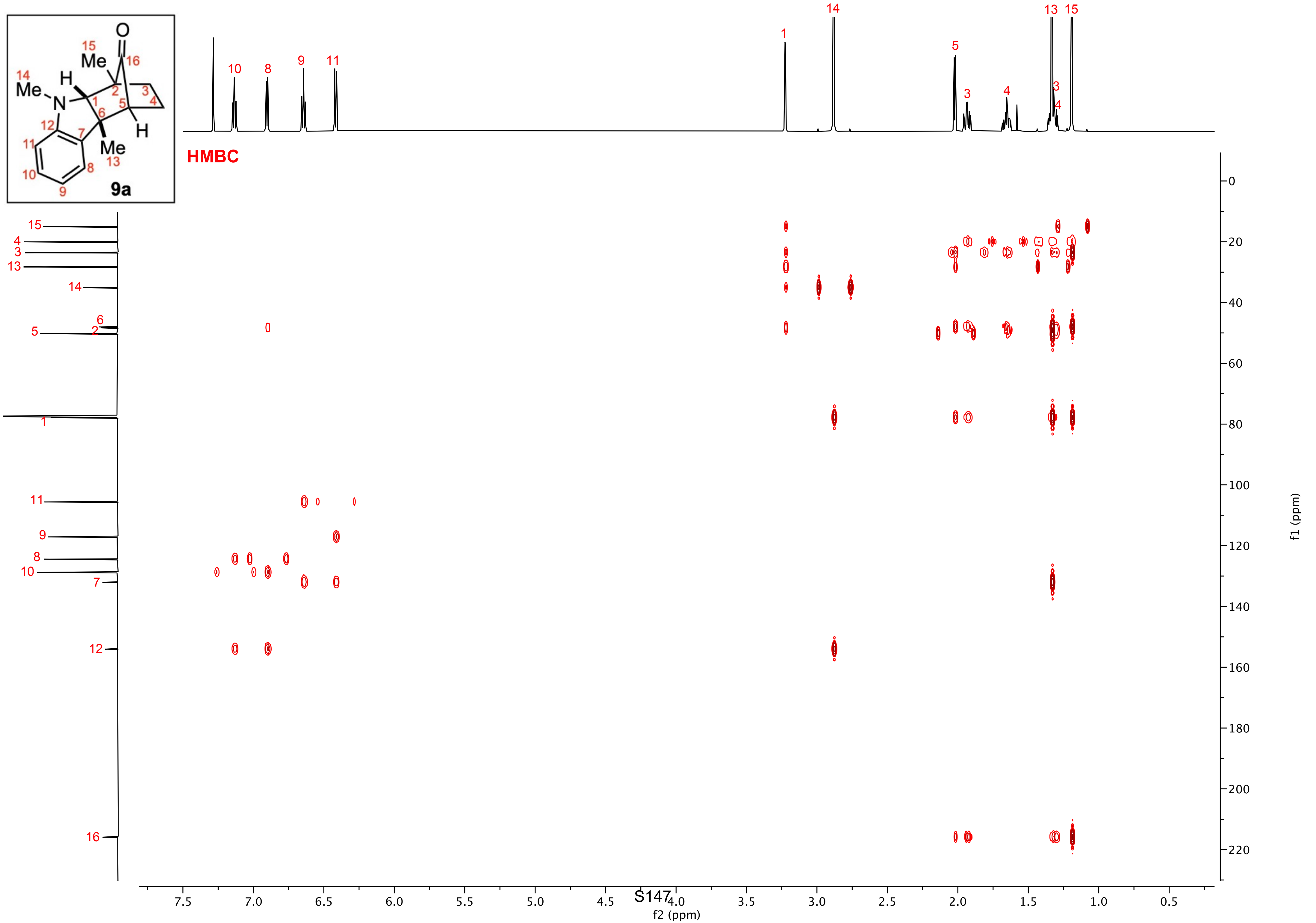
O
Me
H
Me
N
Me
H
9a
HMBC
f2 (ppm)
f1 (ppm)

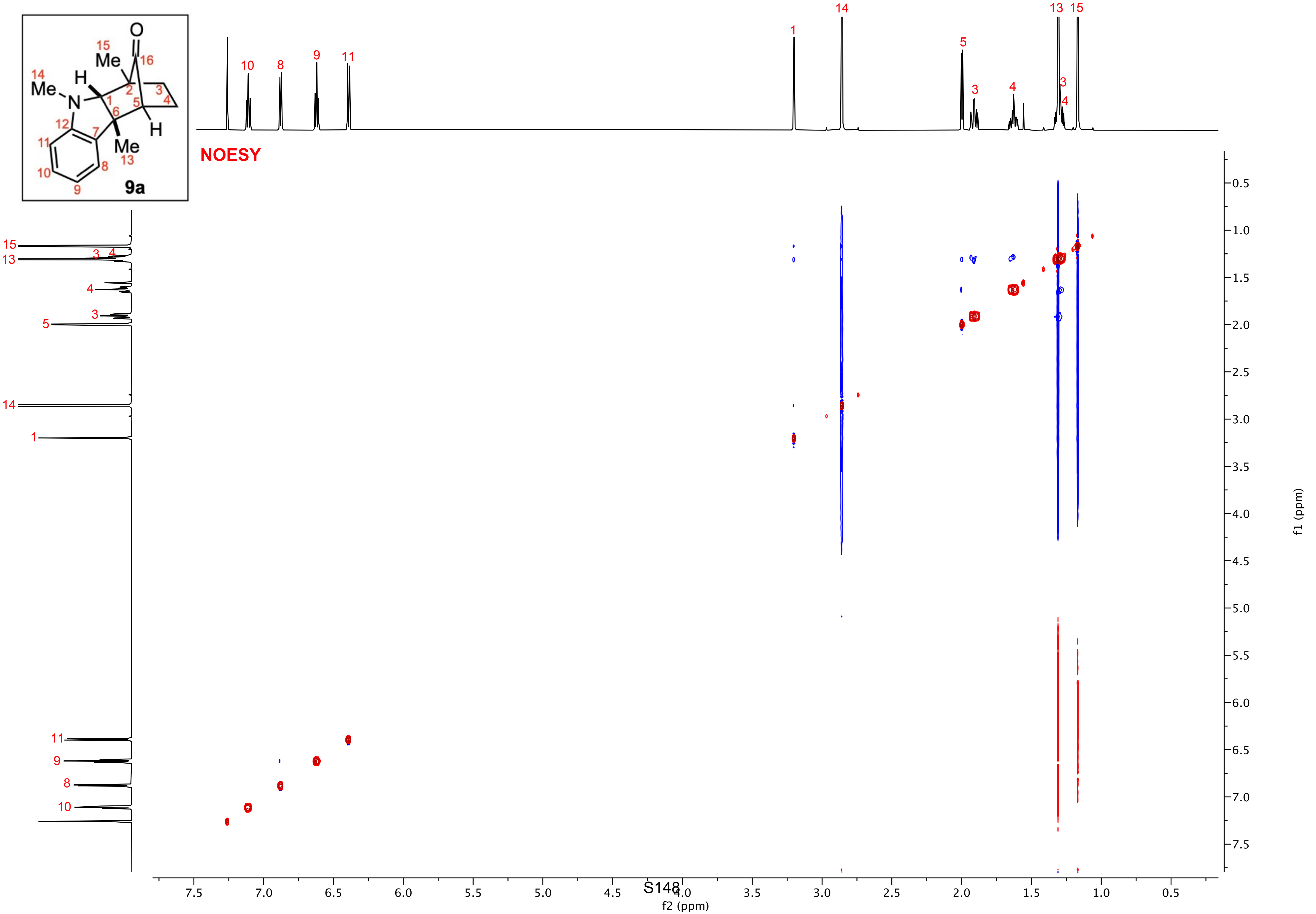
9a
NOESY
f2 (ppm)
f1 (ppm)

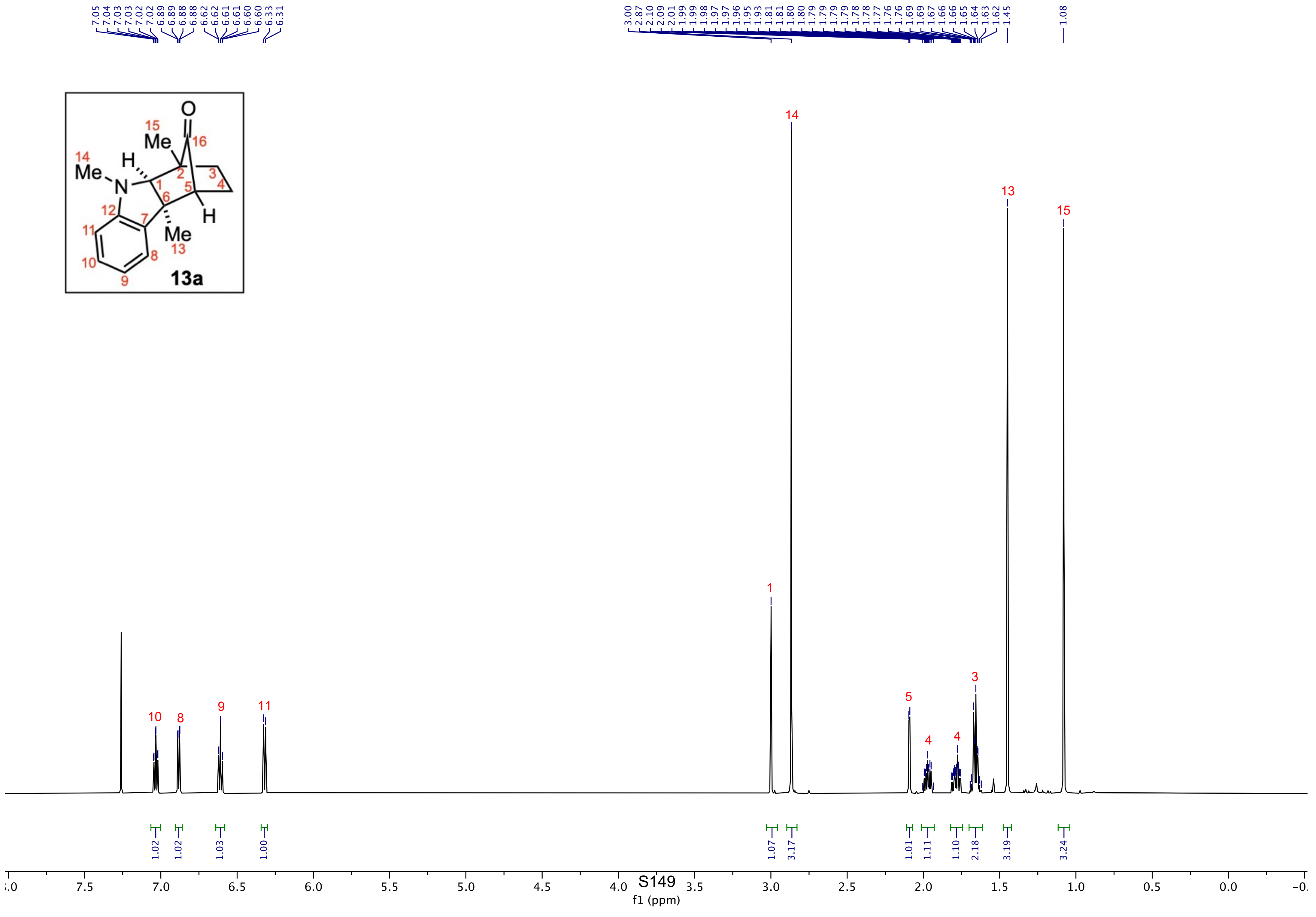
7.05 7.04 7.03 7.03 7.02 7.02 6.89 6.89 6.88 6.88 6.62 6.62 6.61 6.61 6.60 6.60 6.33 6.31
3.00 2.87 2.10 2.09 2.01 1.99 1.99 1.98 1.97 1.97 1.96 1.95 1.93 1.81 1.81 1.80 1.80 1.79 1.79 1.79 1.79 1.78 1.78 1.77 1.76 1.76 1.69 1.69 1.67 1.67 1.66 1.66 1.65 1.64 1.63 1.62 1.45
1.08
13a
1.02 1.02 1.03 1.00 1.07 3.17 1.01 1.11 1.10 2.18 3.19 3.24
f1 (ppm)

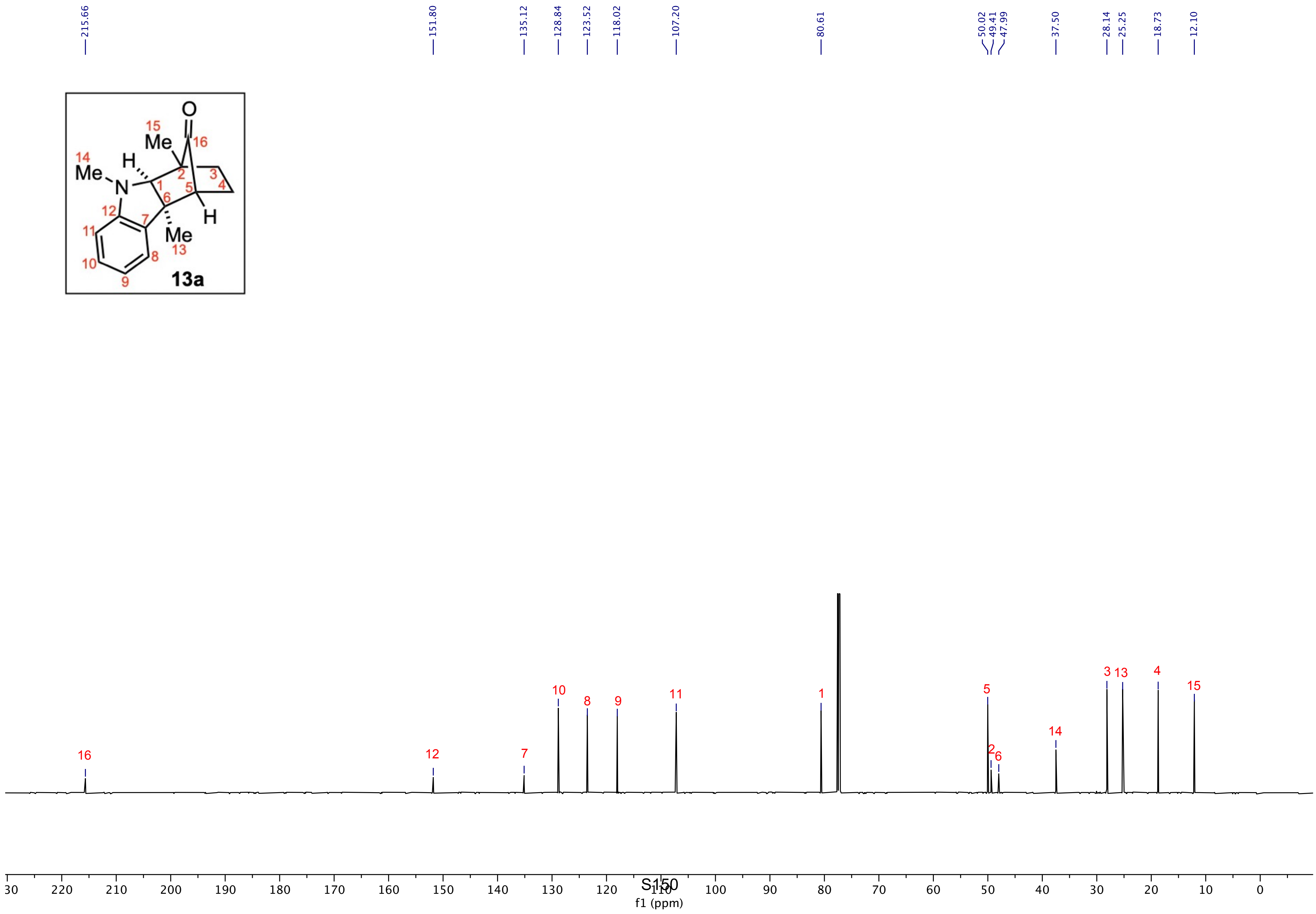
215.66
151.80
135.12
128.84
123.52
118.02
107.20
80.61
50.02
49.41
47.99
37.50
28.14
25.25
18.73
12.10
13a
f1 (ppm)

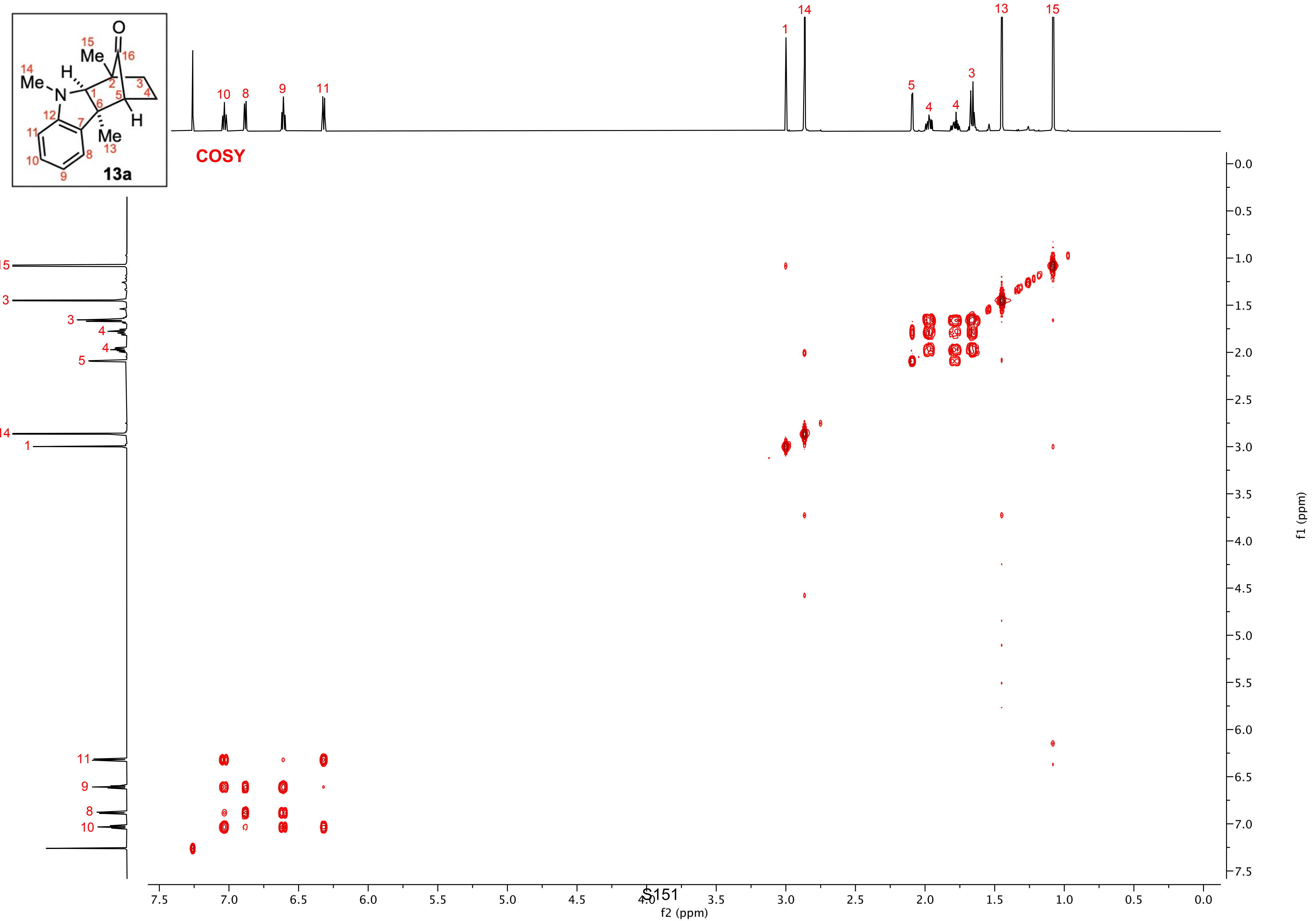
13a
COSY
O
Me
Me
H
H
N
Me
14
15
16
1
2
3
4
5
6
7
8
9
10
11
12
13
f2 (ppm)
f1 (ppm)

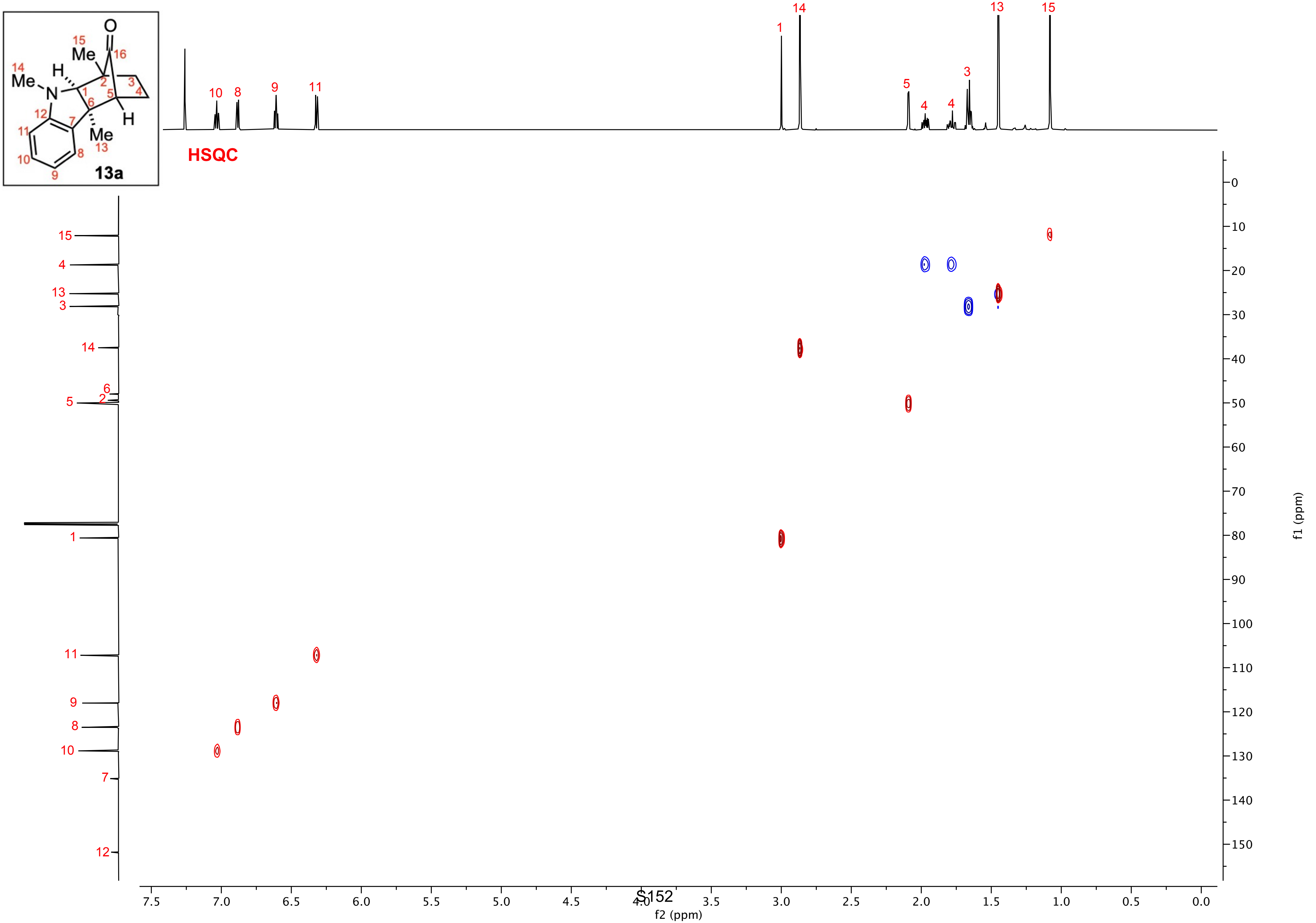
13a
HSQC
f2 (ppm)
f1 (ppm)
1
14
13
15
10
8
9
11
5
4
4
3
6
2
7
12

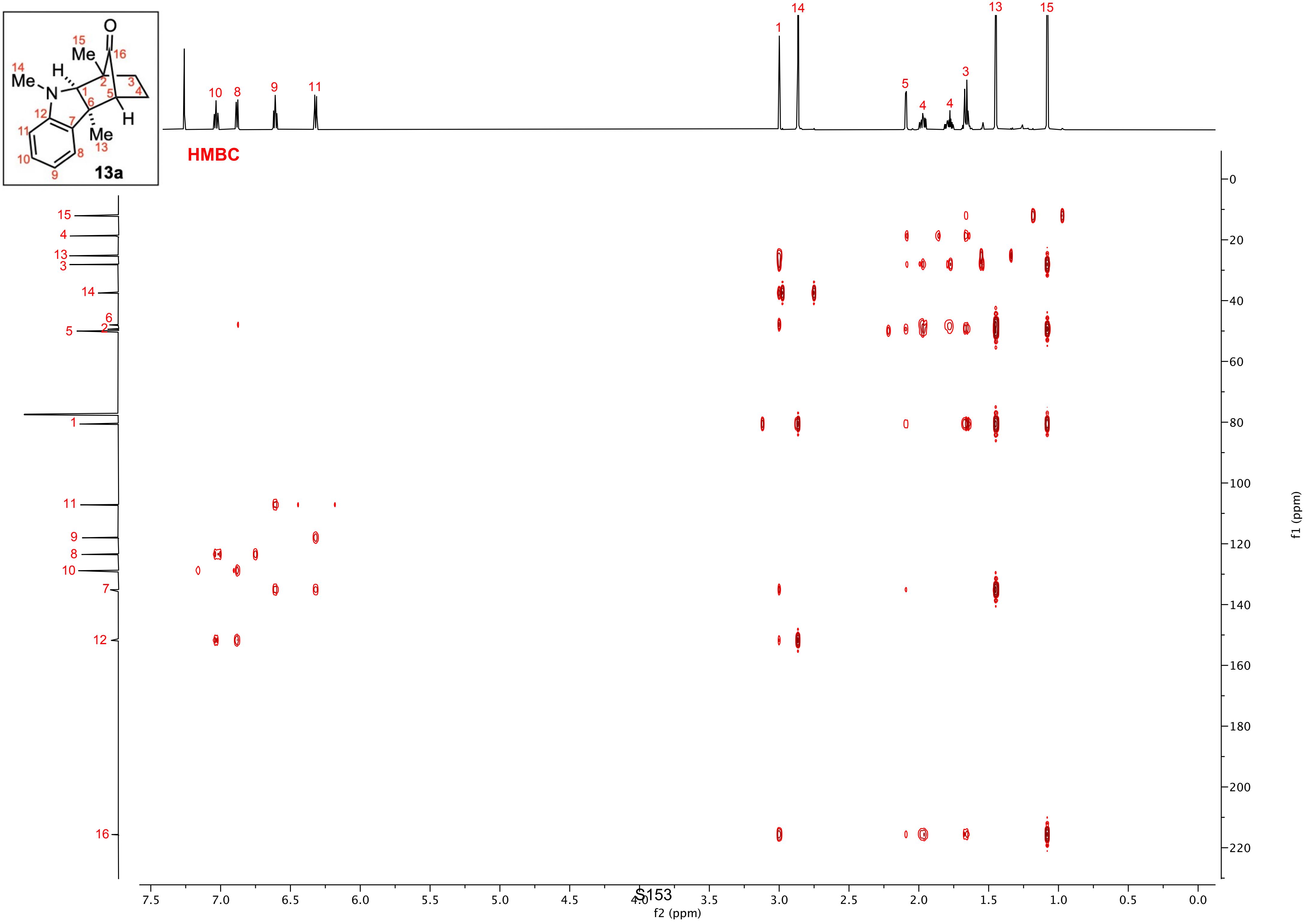
13a
HMBC
f1 (ppm)
f2 (ppm)

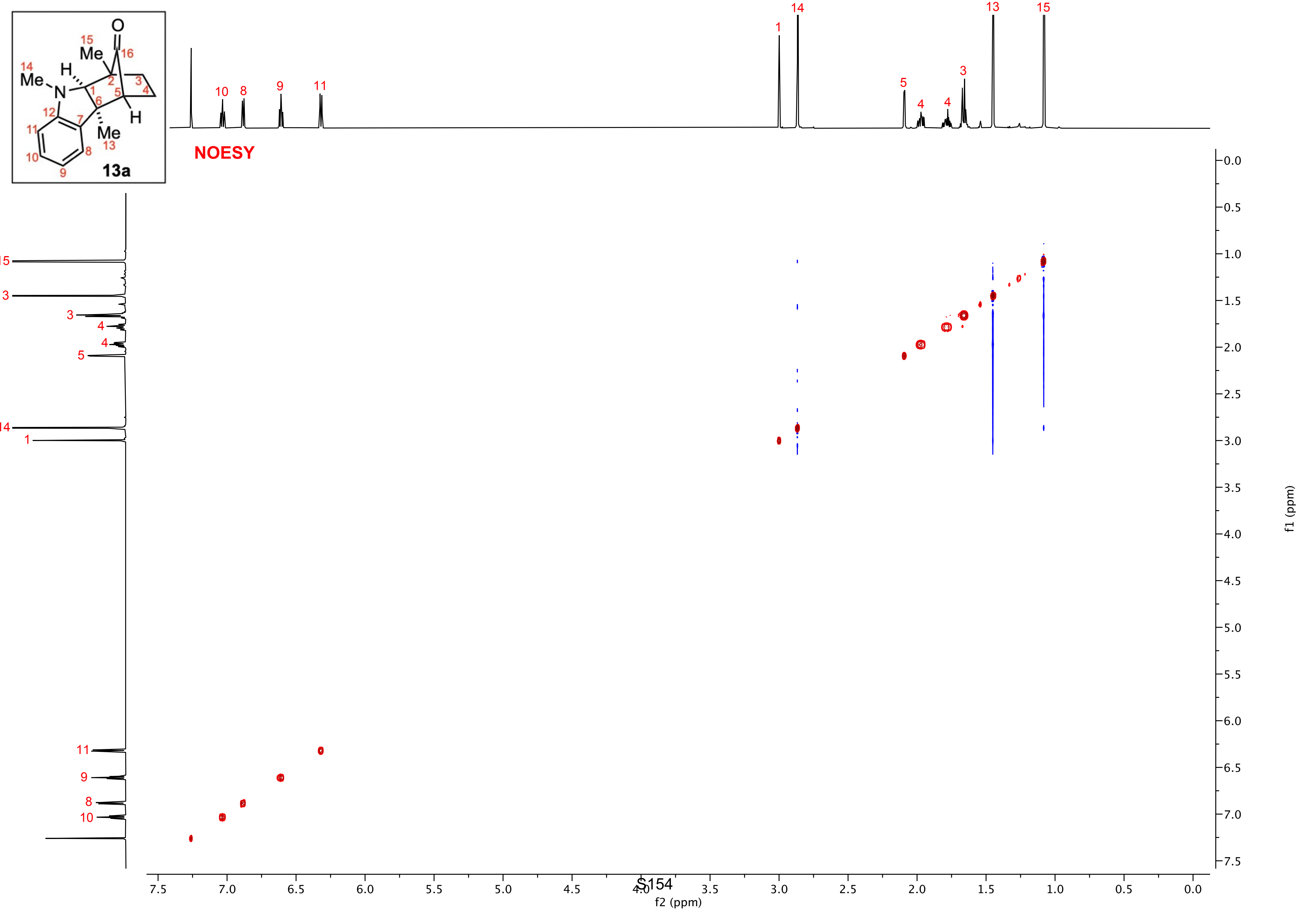
13a
NOESY
f1 (ppm)
f2 (ppm)

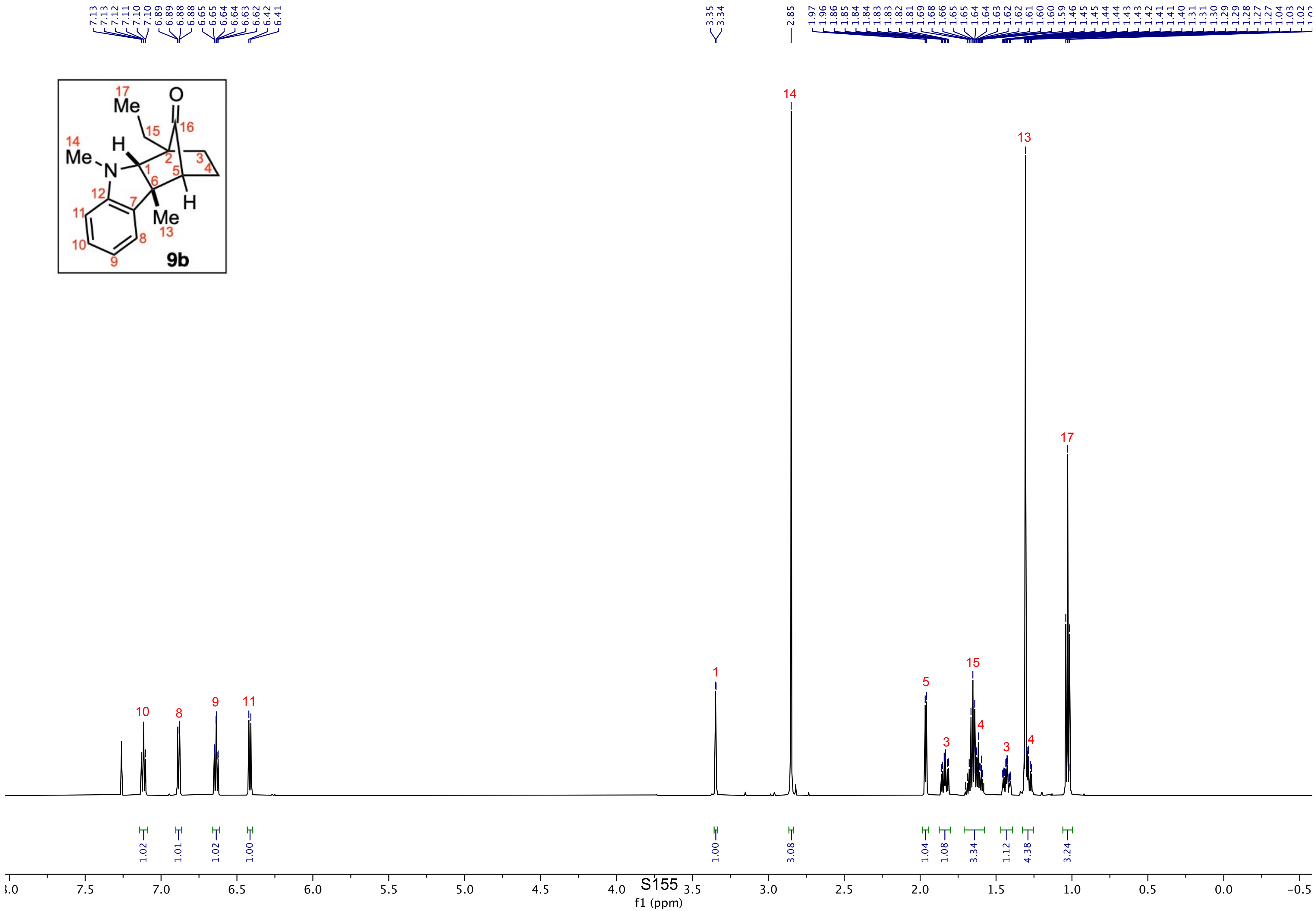
9b
f1 (ppm)

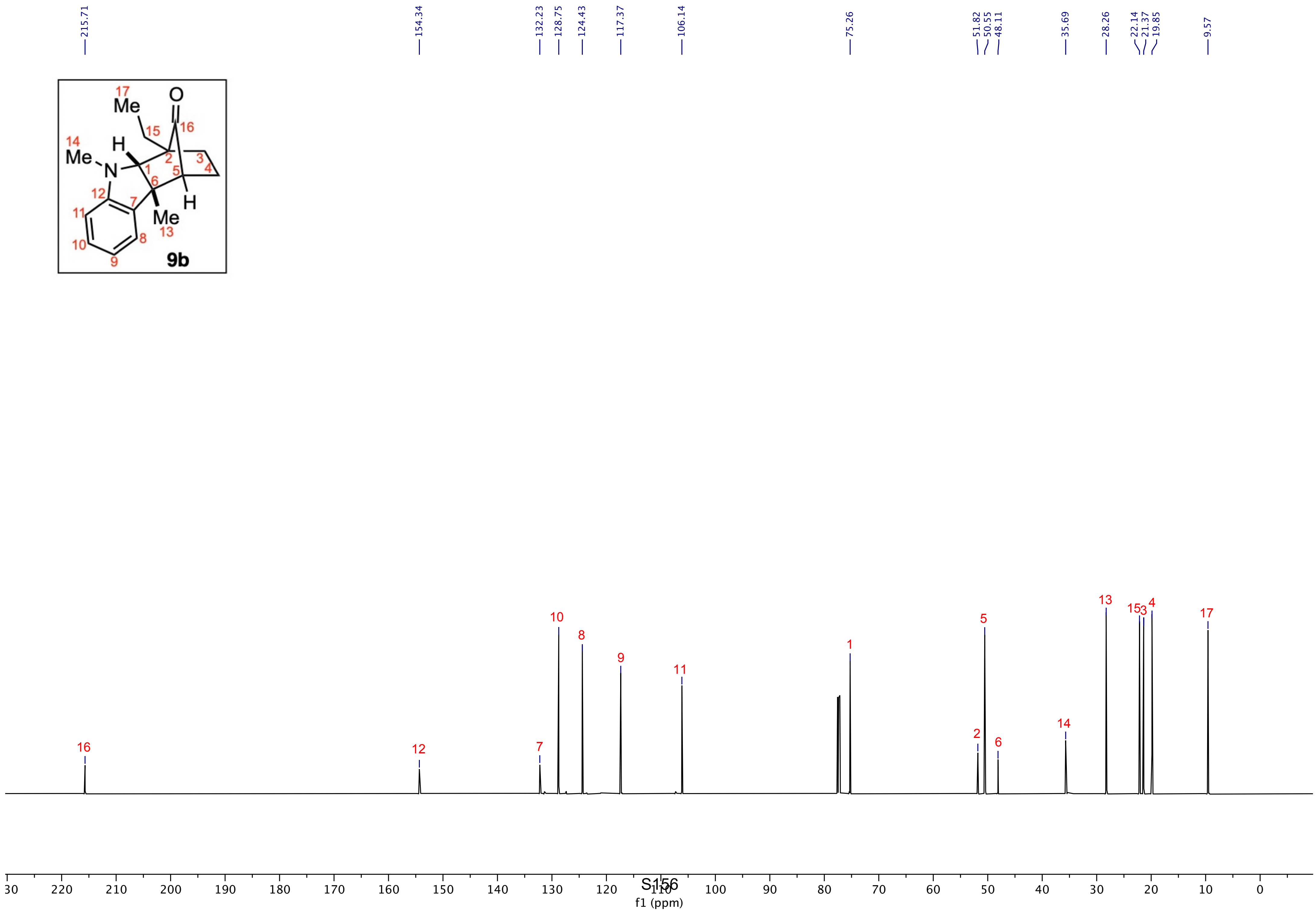
215.71
154.34
132.23
128.75
124.43
117.37
106.14
75.26
51.82
50.55
48.11
35.69
28.26
22.14
21.37
19.85
9.57
9b
f1 (ppm)

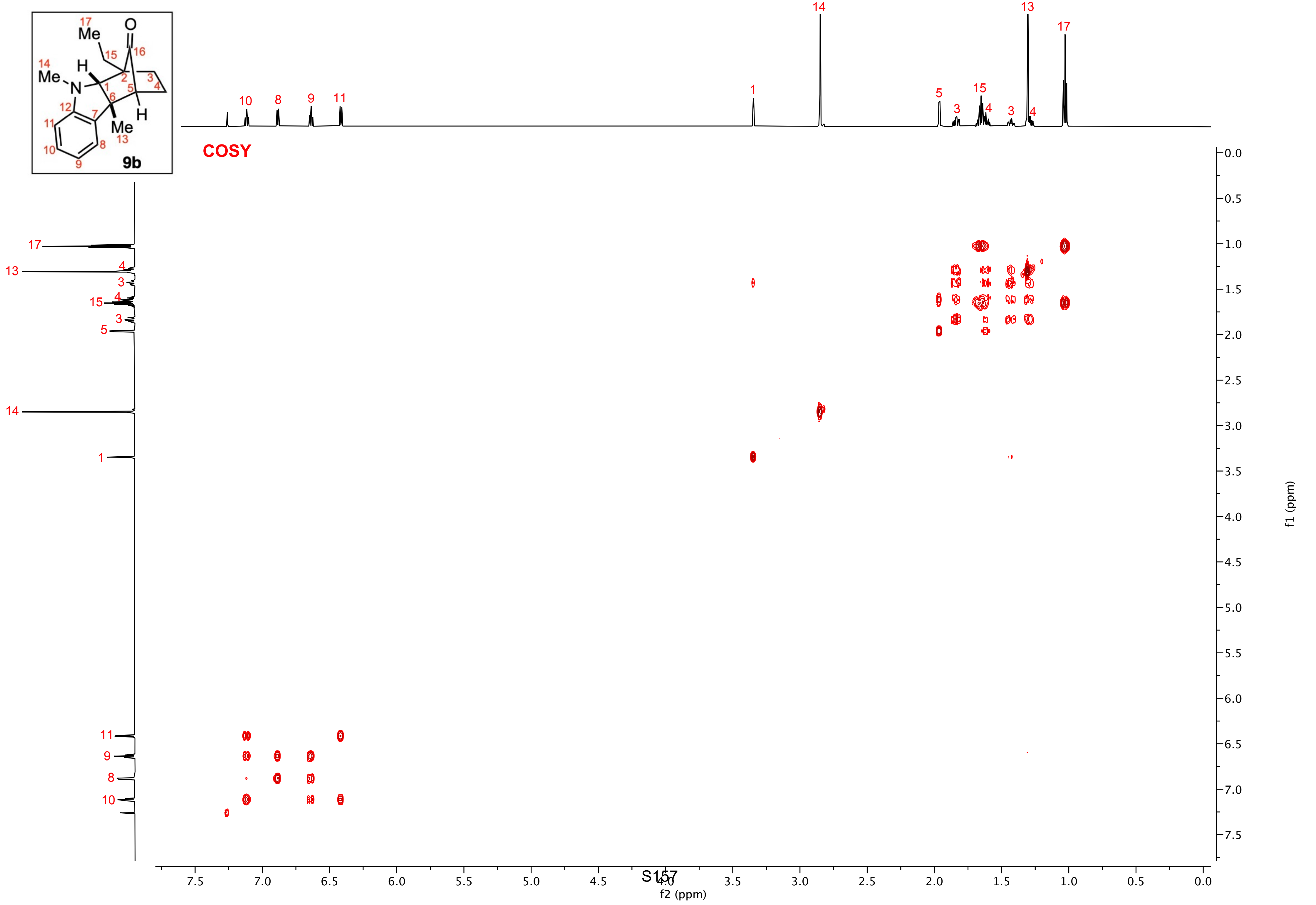

9b
COSY
f2 (ppm)
f1 (ppm)

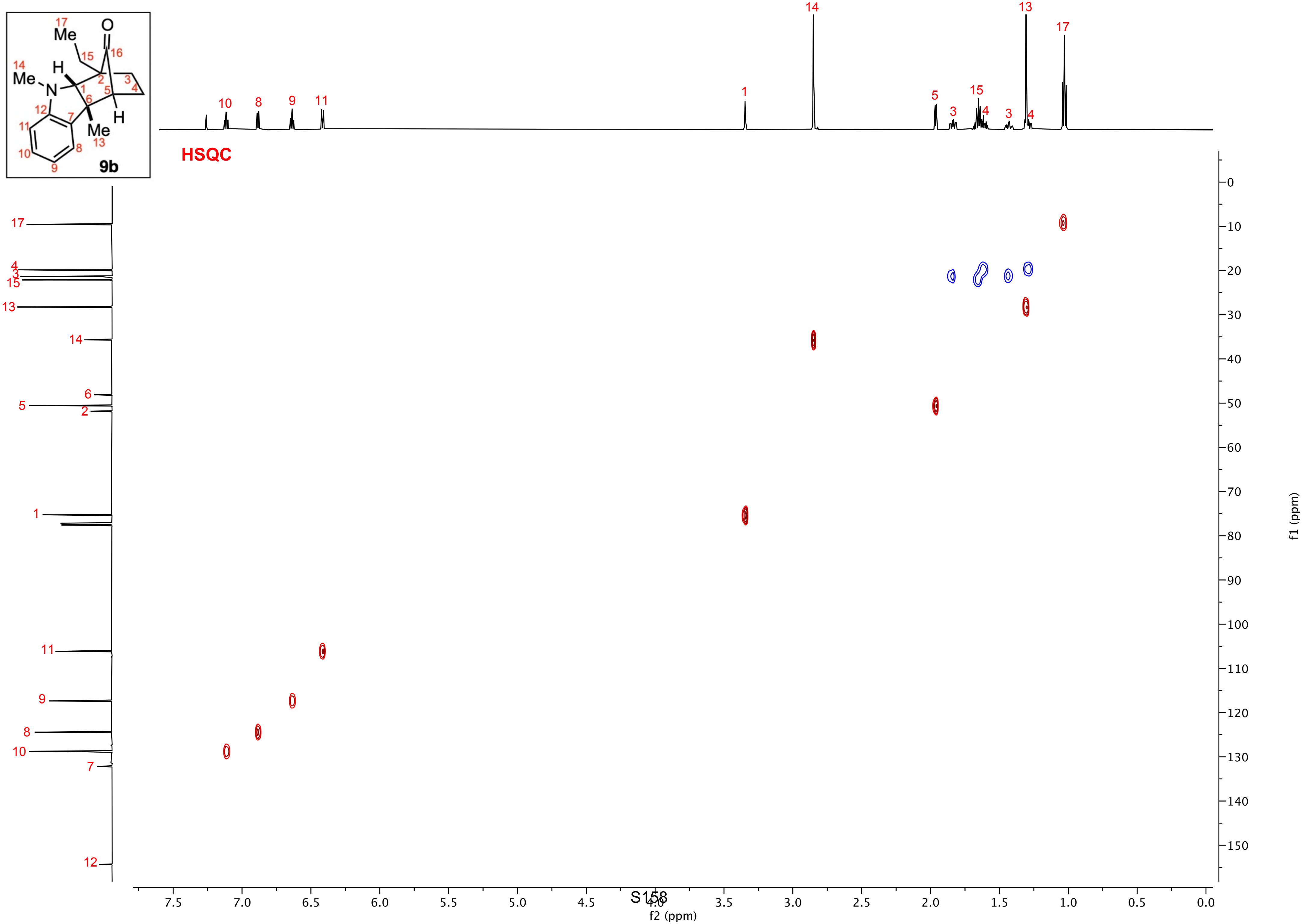

9b
HSQC
f2 (ppm)
f1 (ppm)

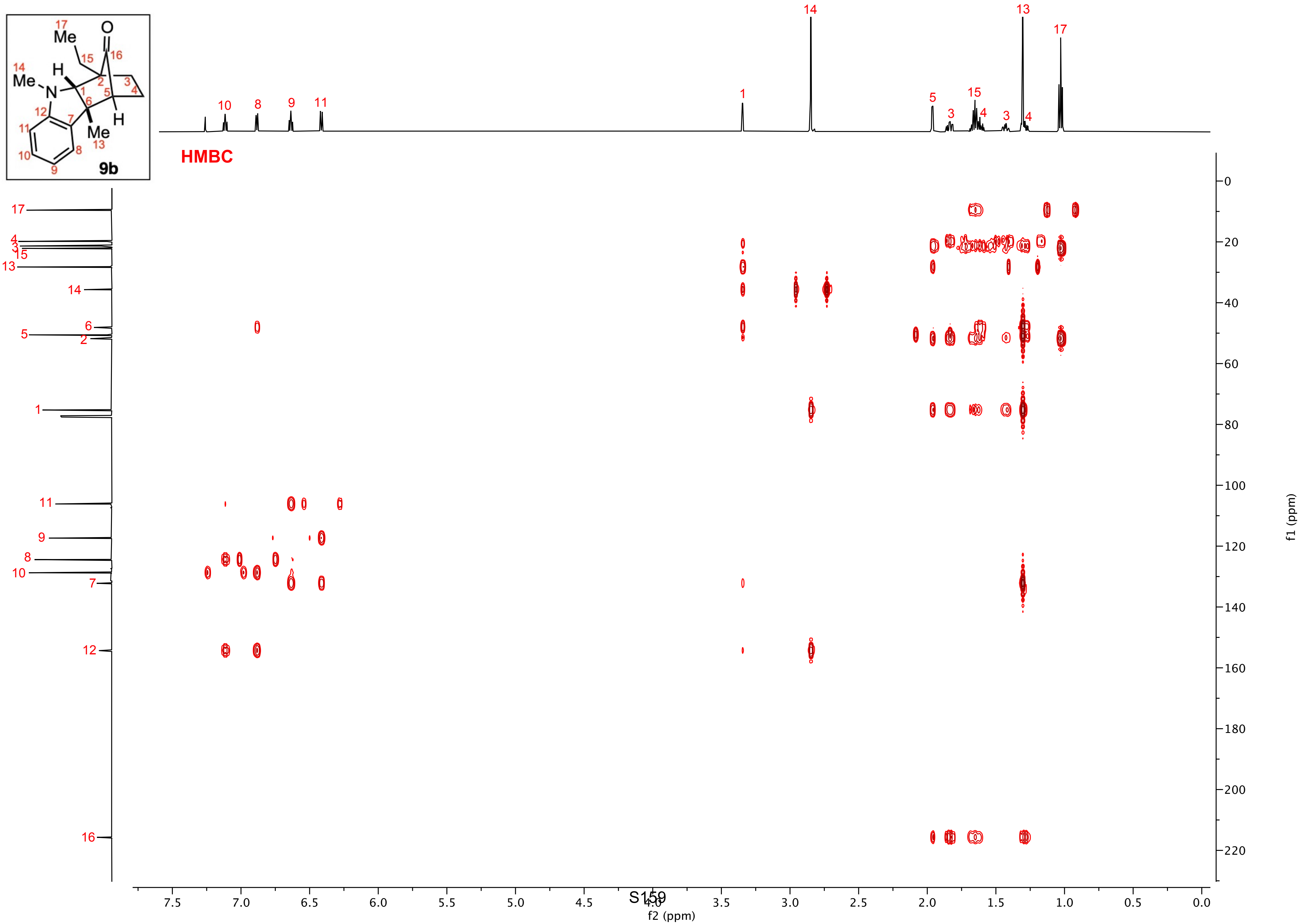

HMBC
9b
Me
O
H
N
f2 (ppm)
f1 (ppm)

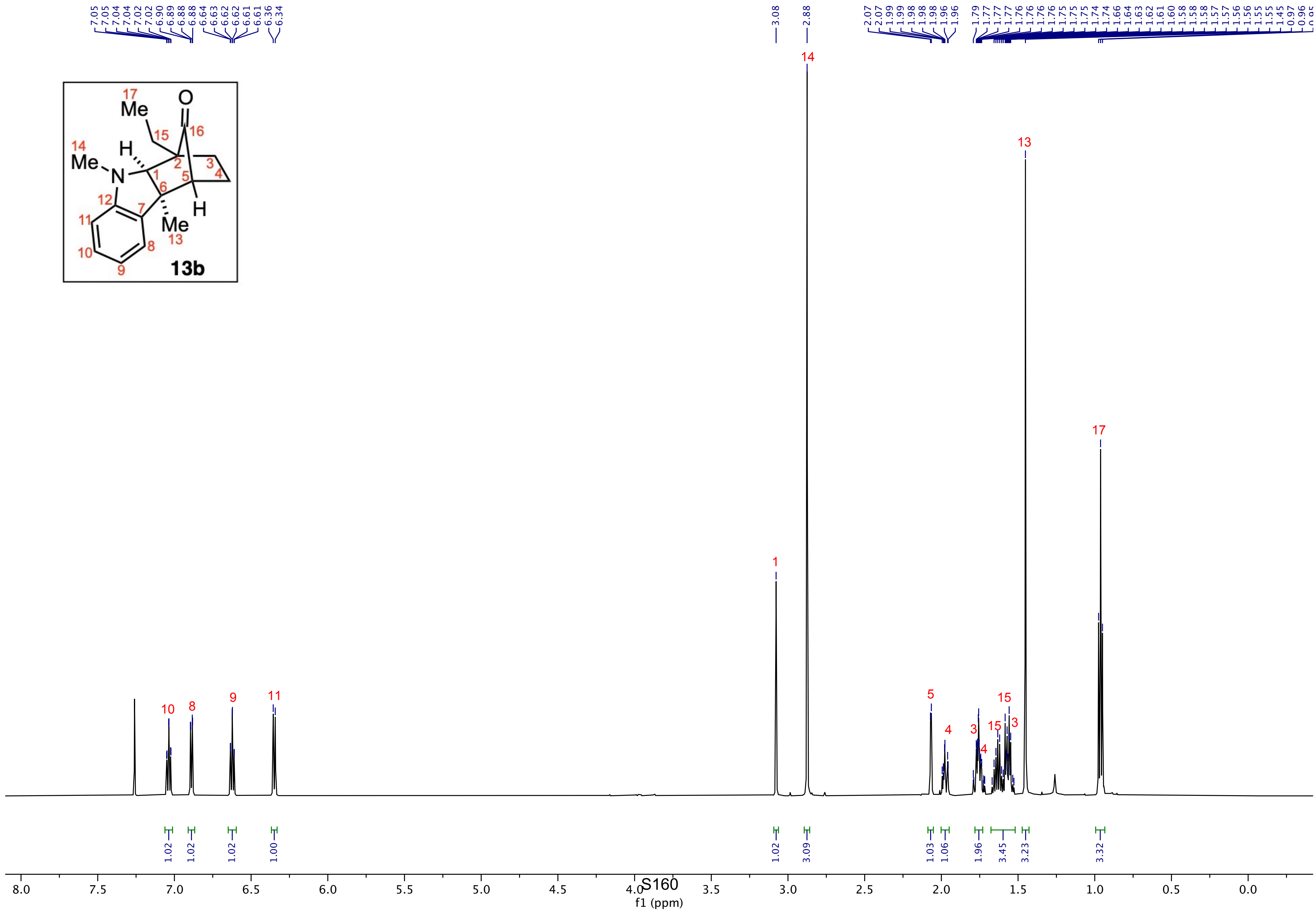
13b
f1 (ppm)

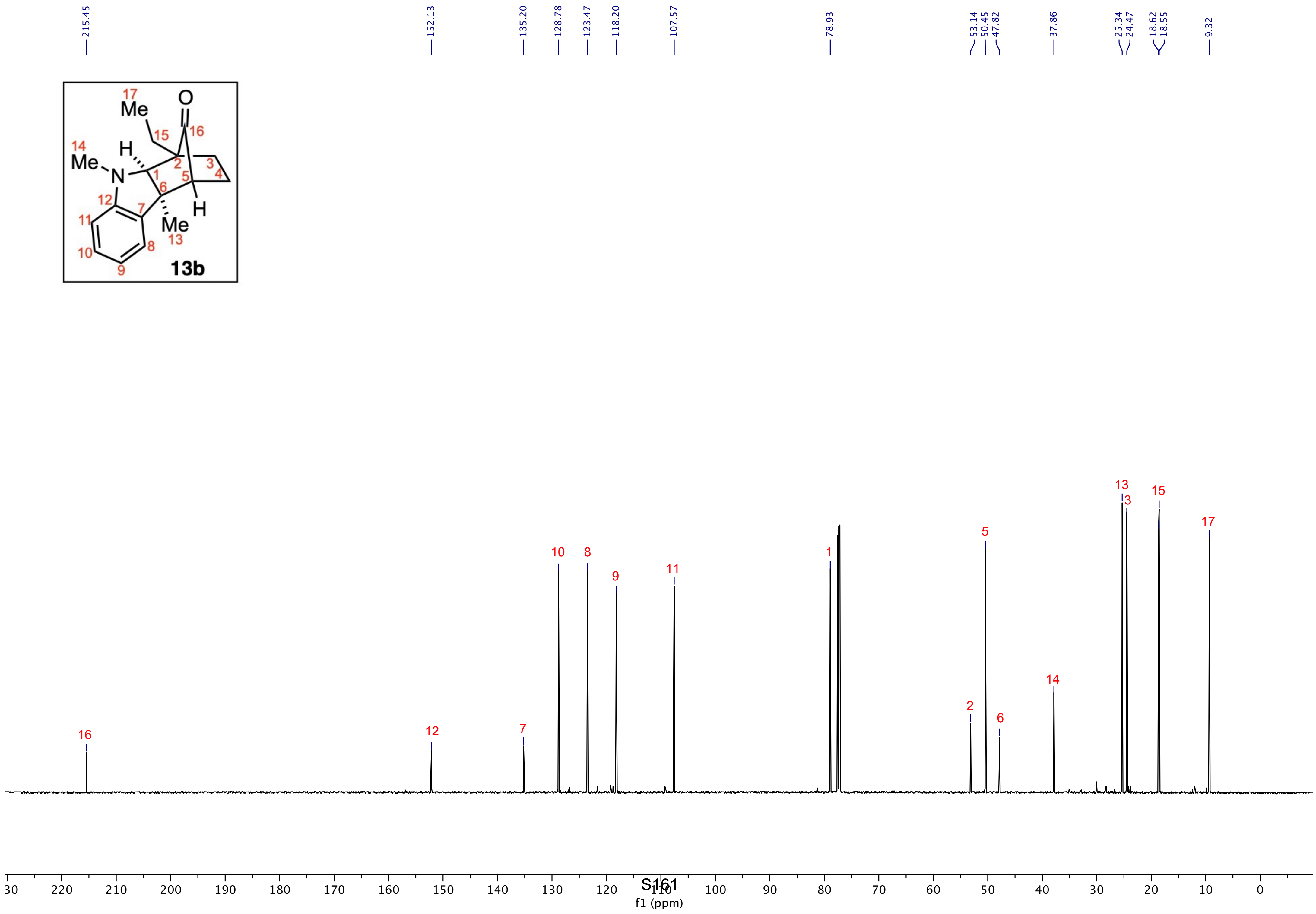
215.45
152.13
135.20
128.78
123.47
118.20
107.57
78.93
53.14
50.45
47.82
37.86
25.34
24.47
18.62
18.55
9.32
13b
f1 (ppm)

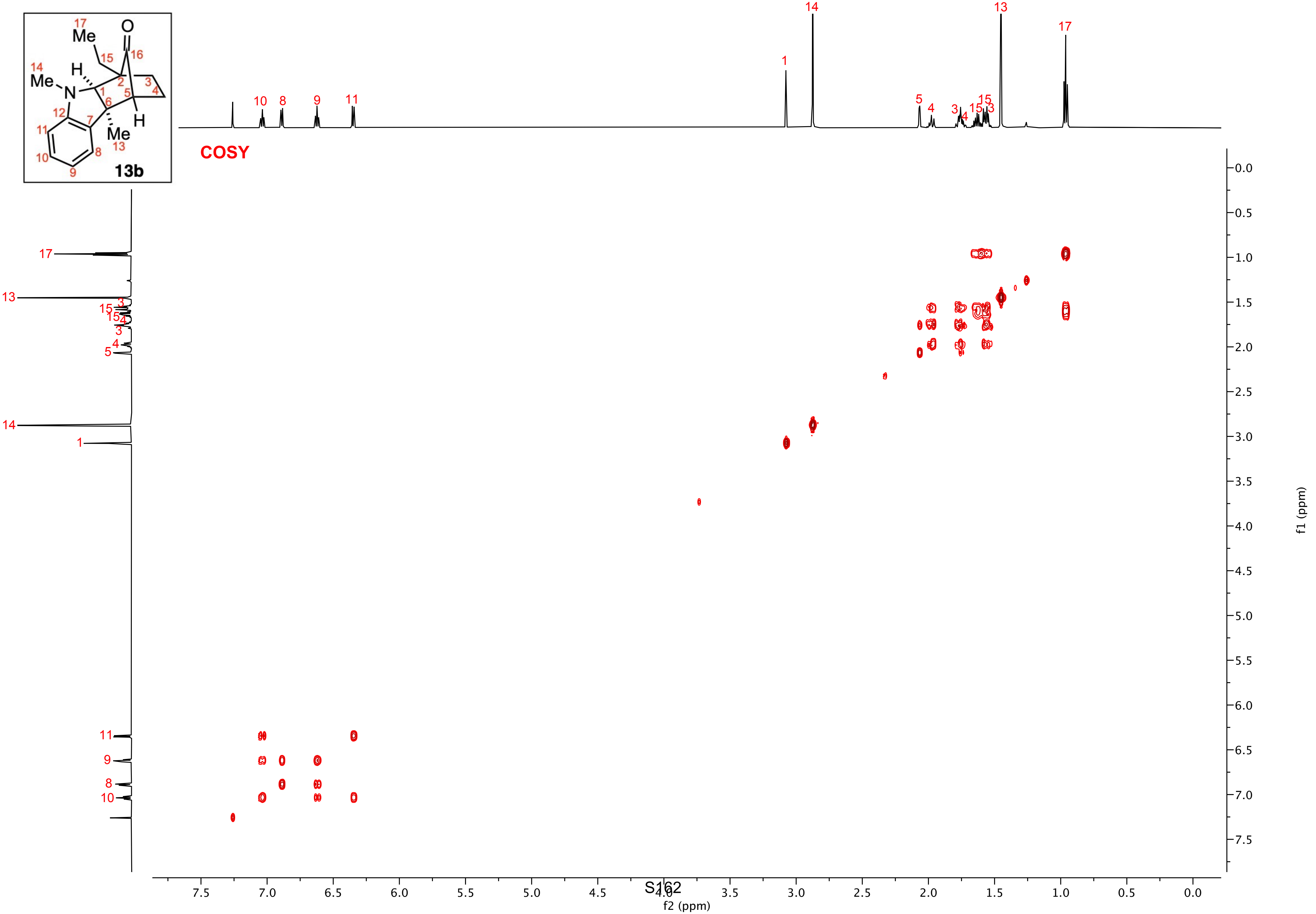

17
Me
O
16
15
14
H
Me
N
1
2
3
4
5
6
12
7
11
H
Me
13
10
8
9
13b
COSY
14
13
17
1
10
8
9
11
5
4
3
15
15
4
3
f2 (ppm)
f1 (ppm)

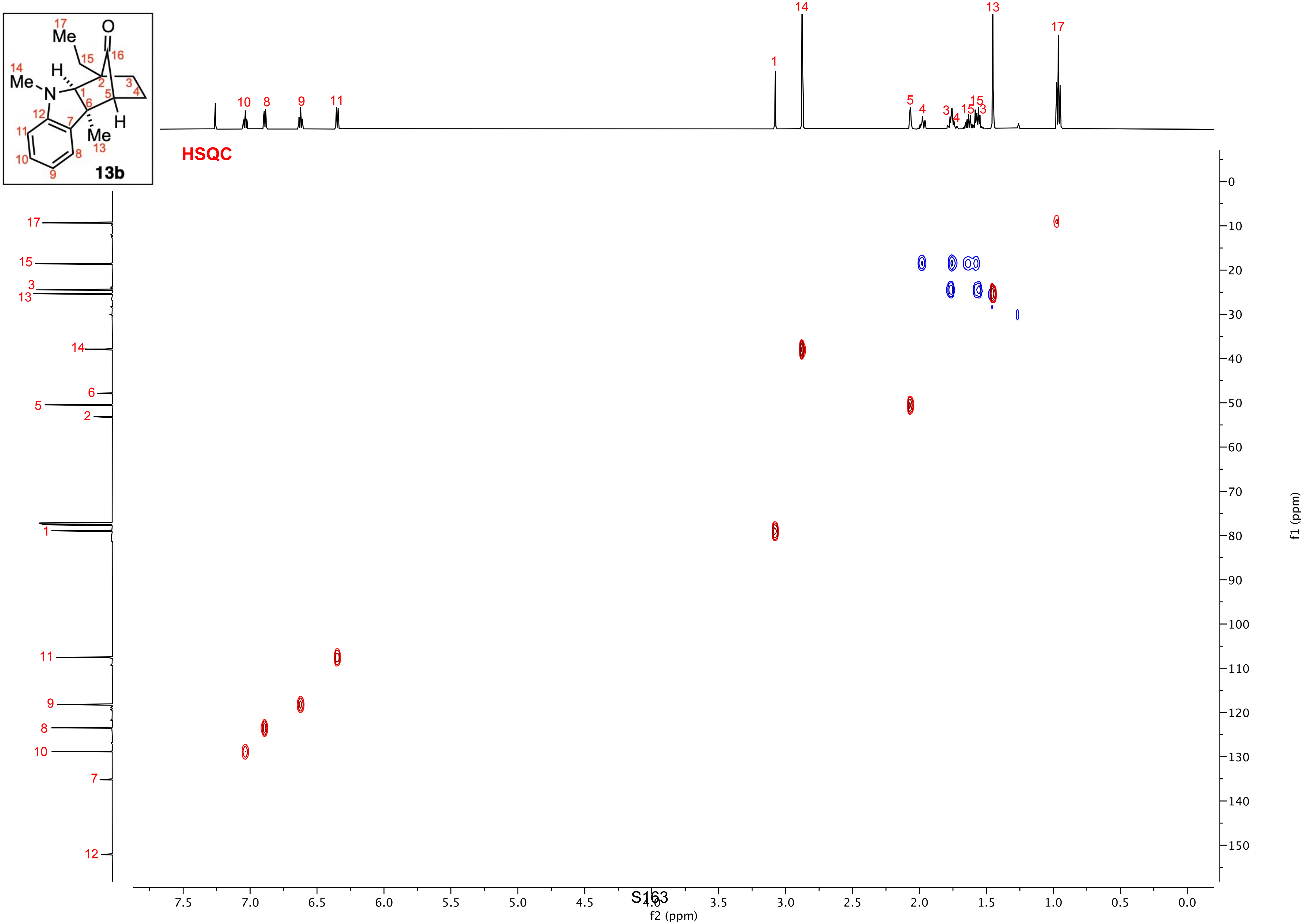
13b
HSQC
f2 (ppm)
f1 (ppm)

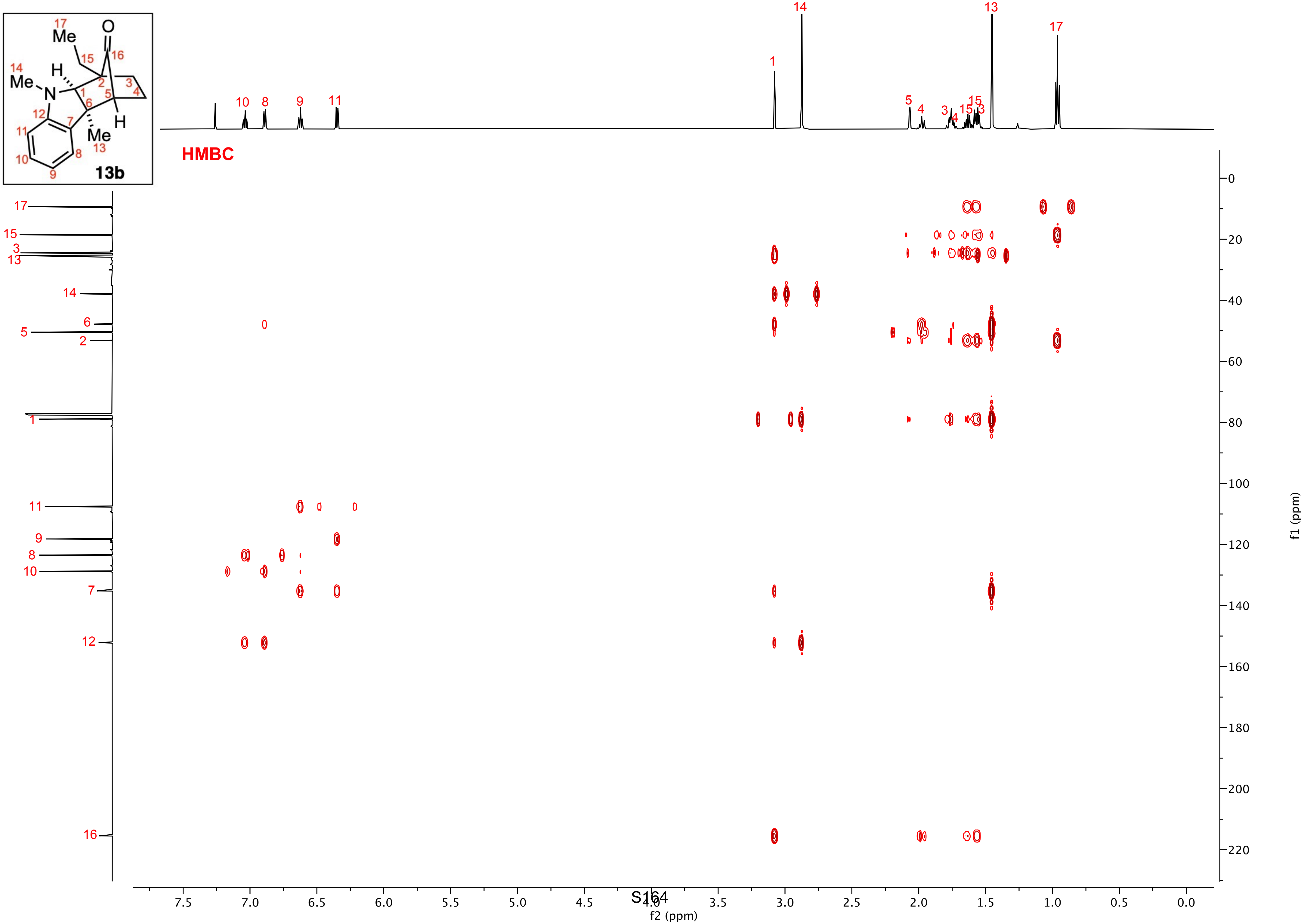

13b
HMBC
f2 (ppm)
f1 (ppm)

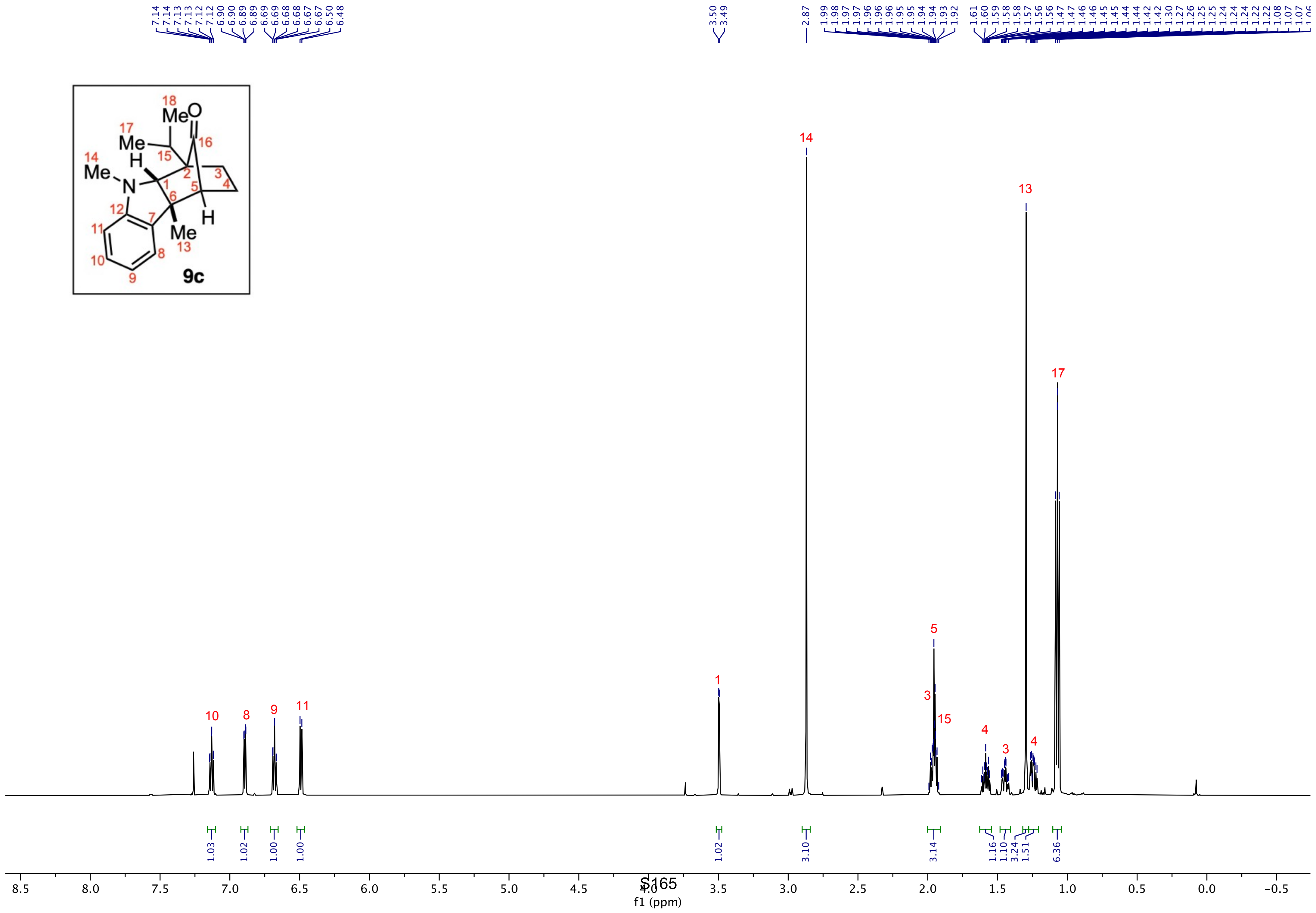
9c
f1 (ppm)

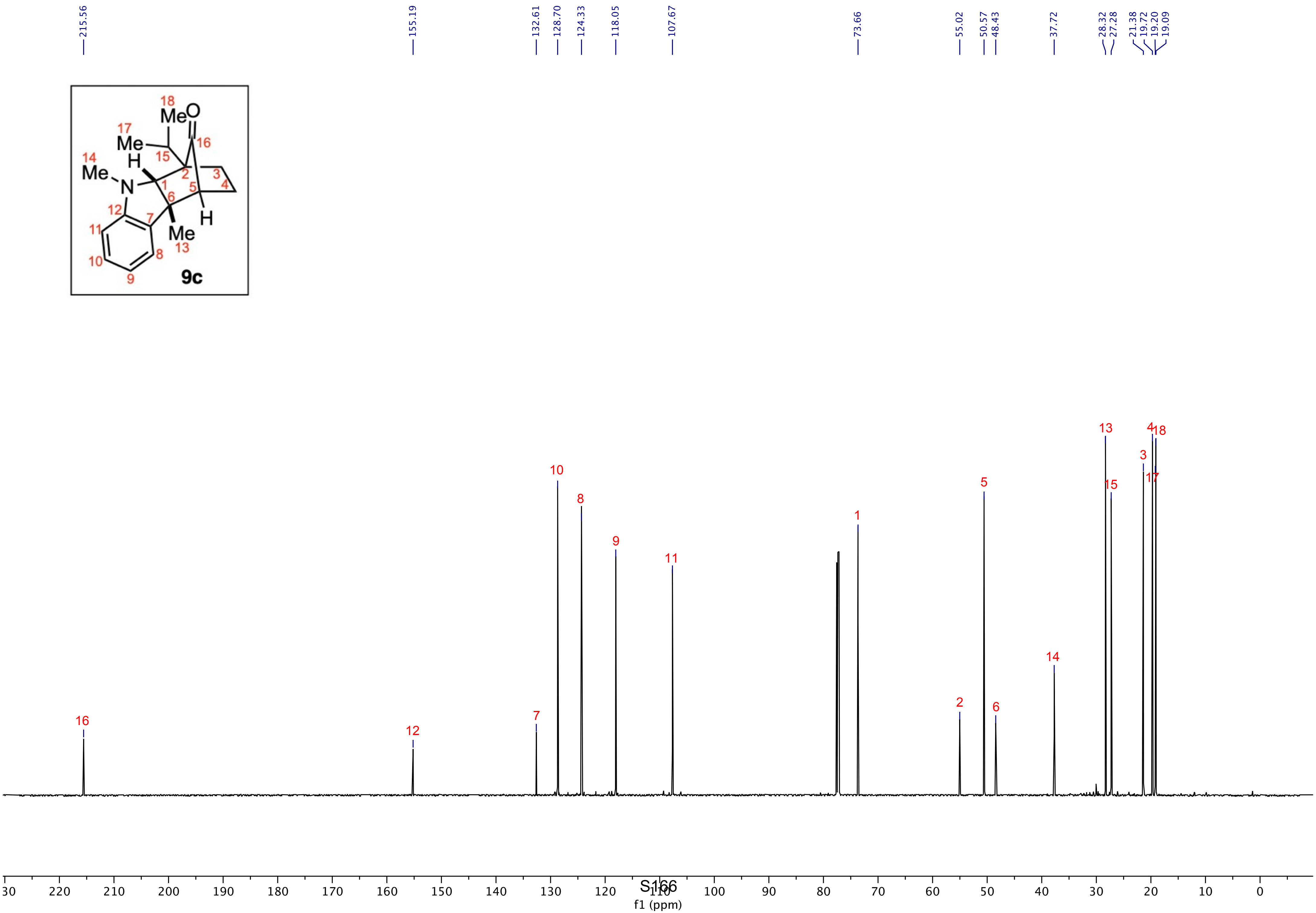
215.56
155.19
132.61
128.70
124.33
118.05
107.67
73.66
55.02
50.57
48.43
37.72
28.32
27.28
21.38
19.72
19.20
19.09
9c
f1 (ppm)

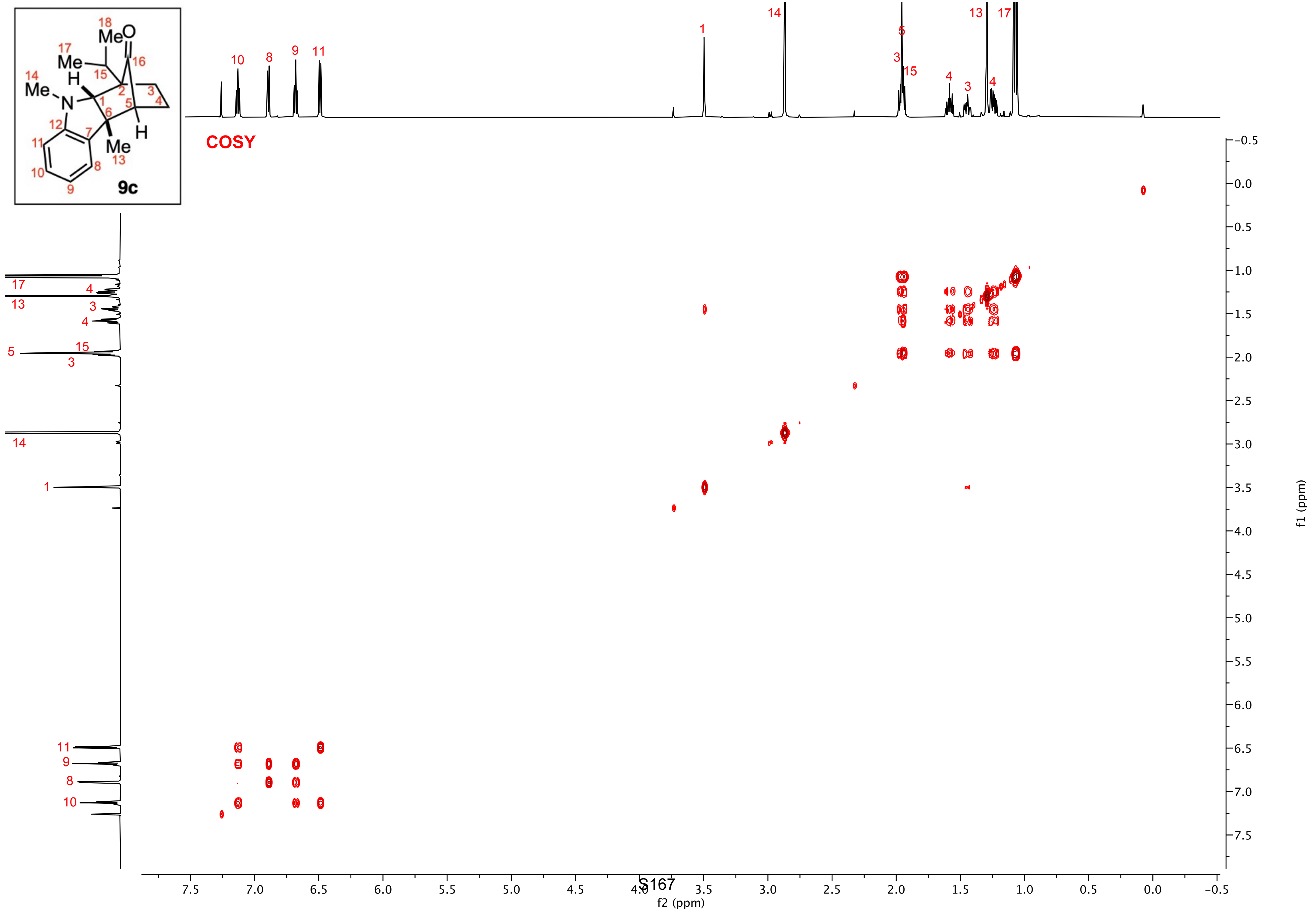
9c
COSY
f2 (ppm)
f1 (ppm)

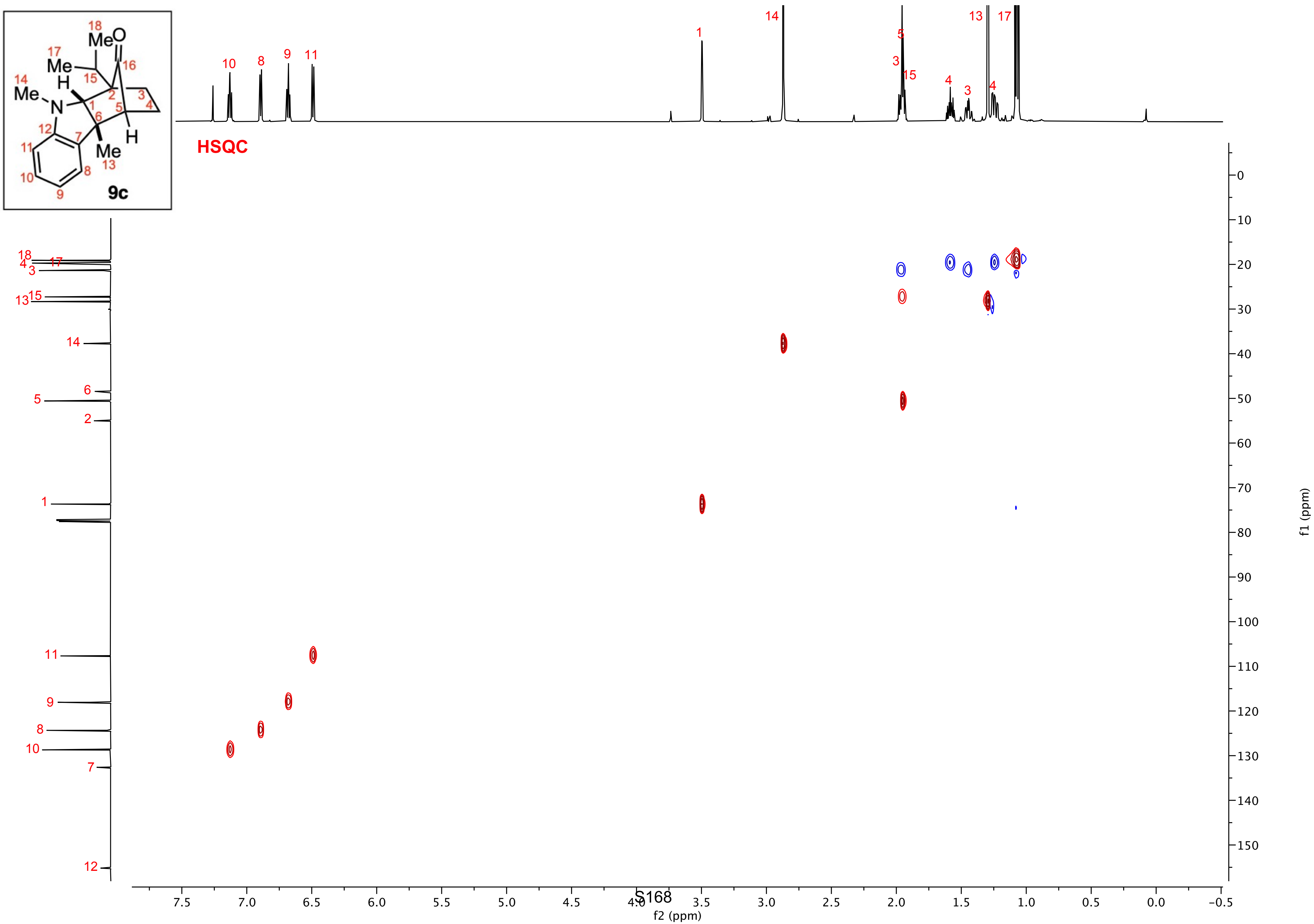

HSQC
9c
f2 (ppm)
f1 (ppm)

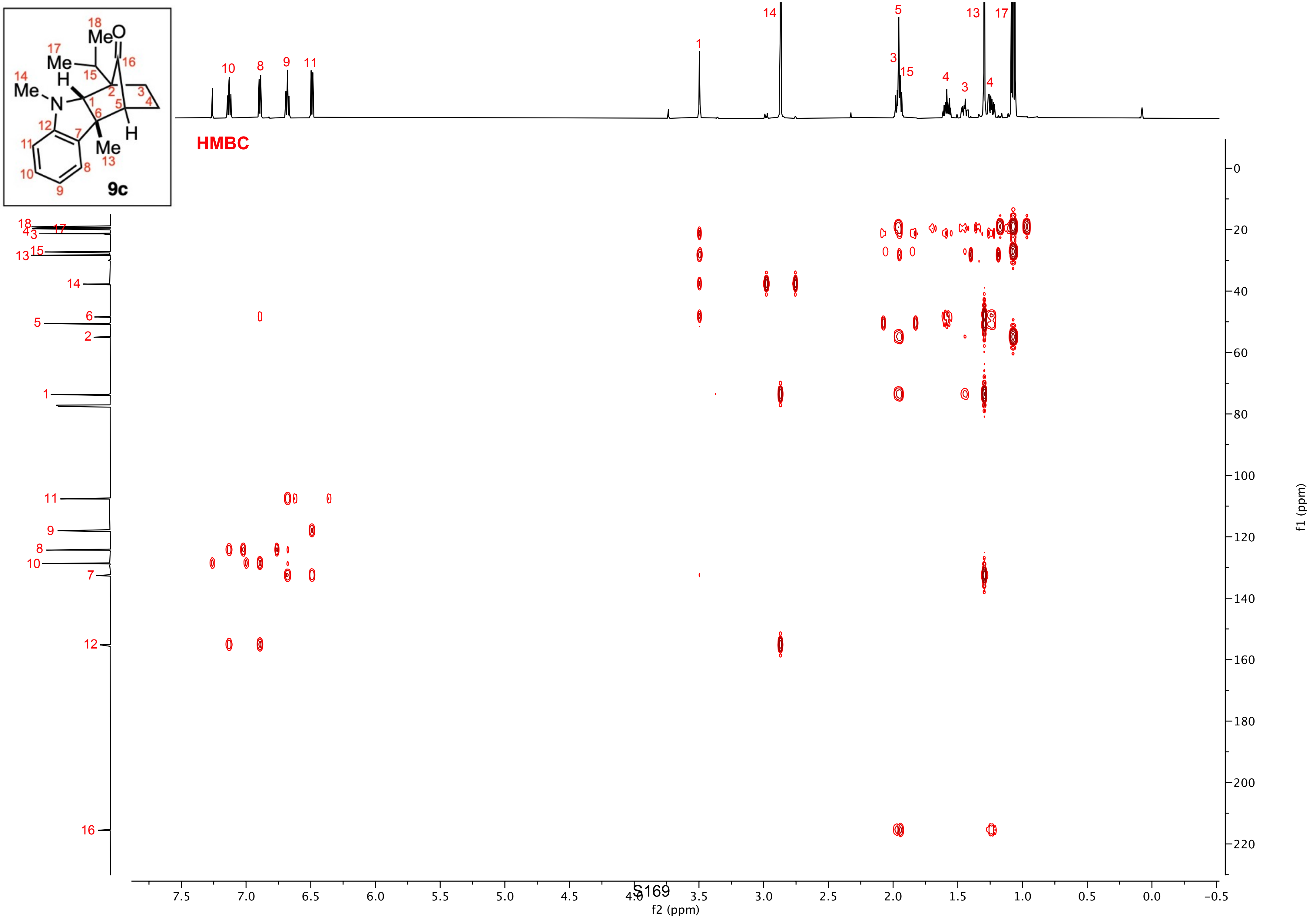
HMBC
9c
f2 (ppm)
f1 (ppm)

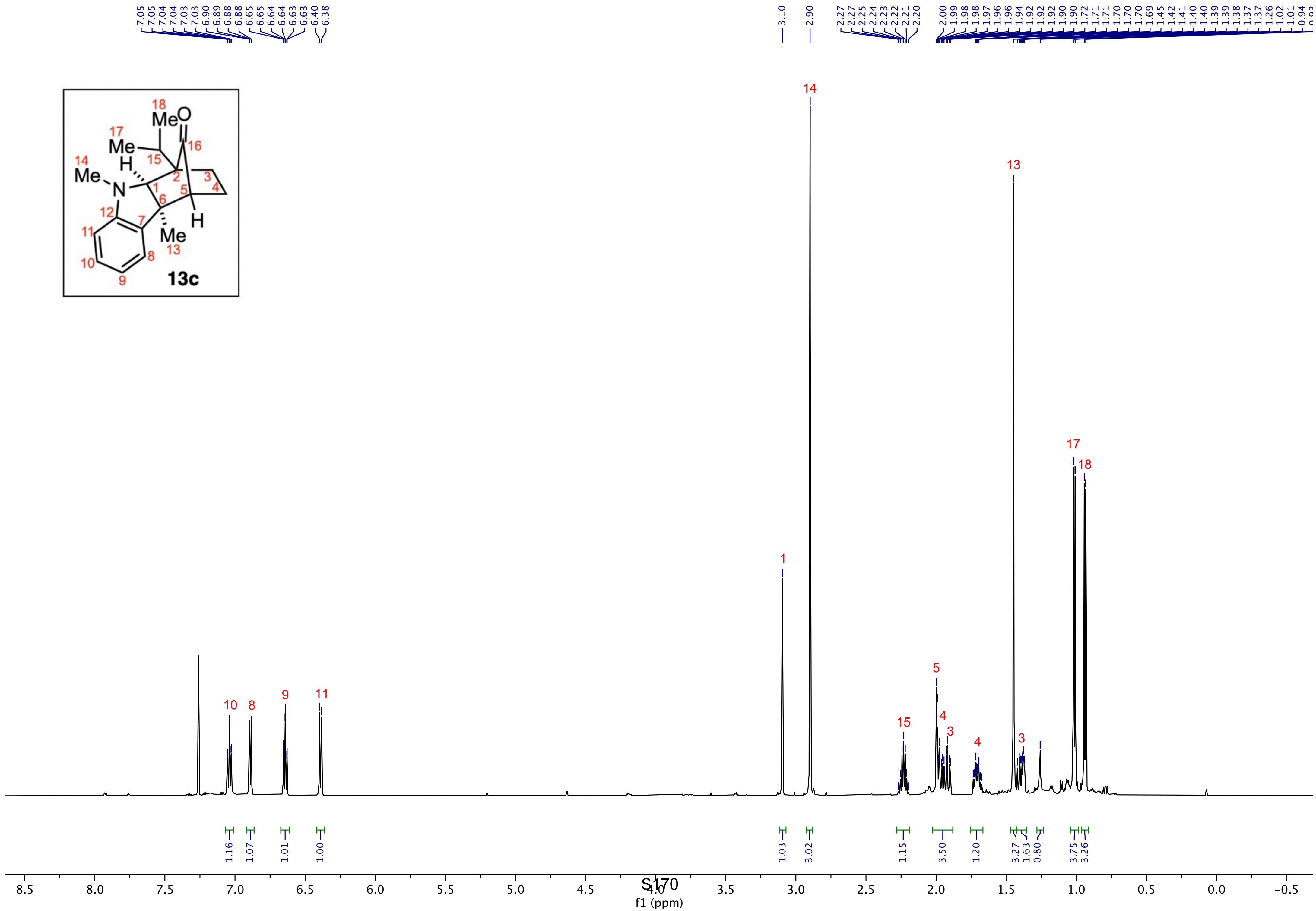
13c
f1 (ppm)

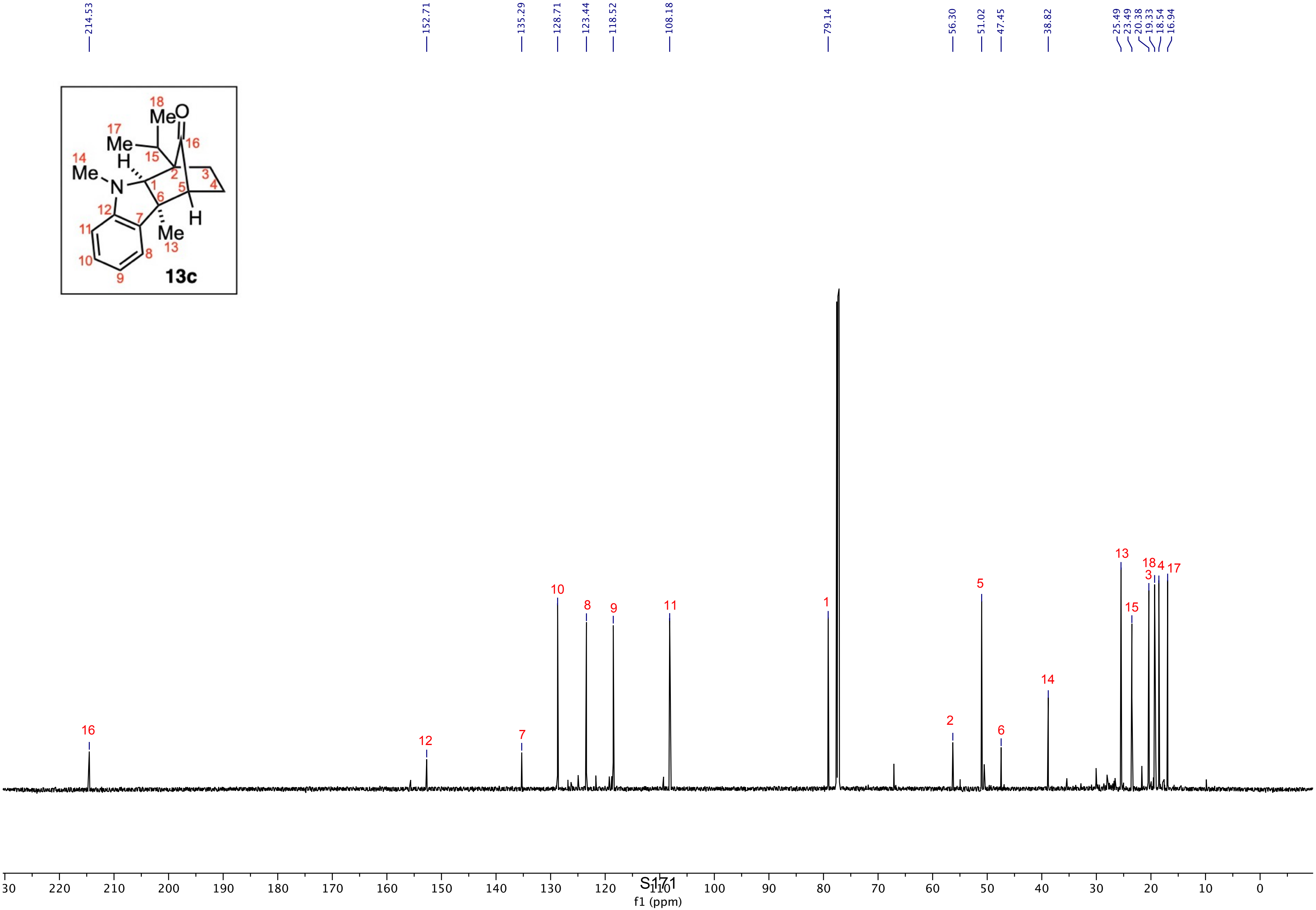
214.53
152.71
135.29
128.71
123.44
118.52
108.18
79.14
56.30
51.02
47.45
38.82
25.49
23.49
20.38
19.33
18.54
16.94
13c
f1 (ppm)

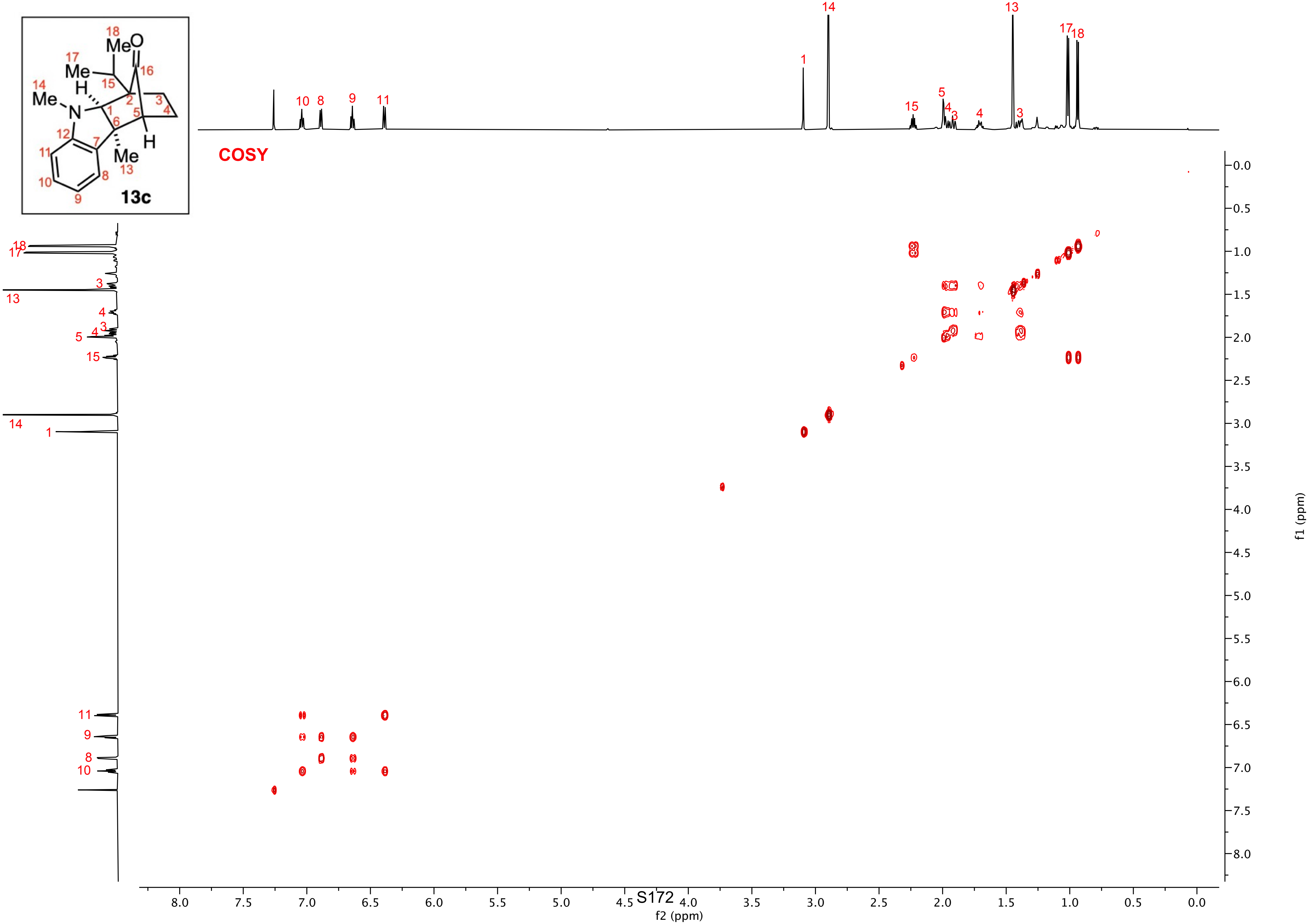
13c
COSY
f2 (ppm)
f1 (ppm)

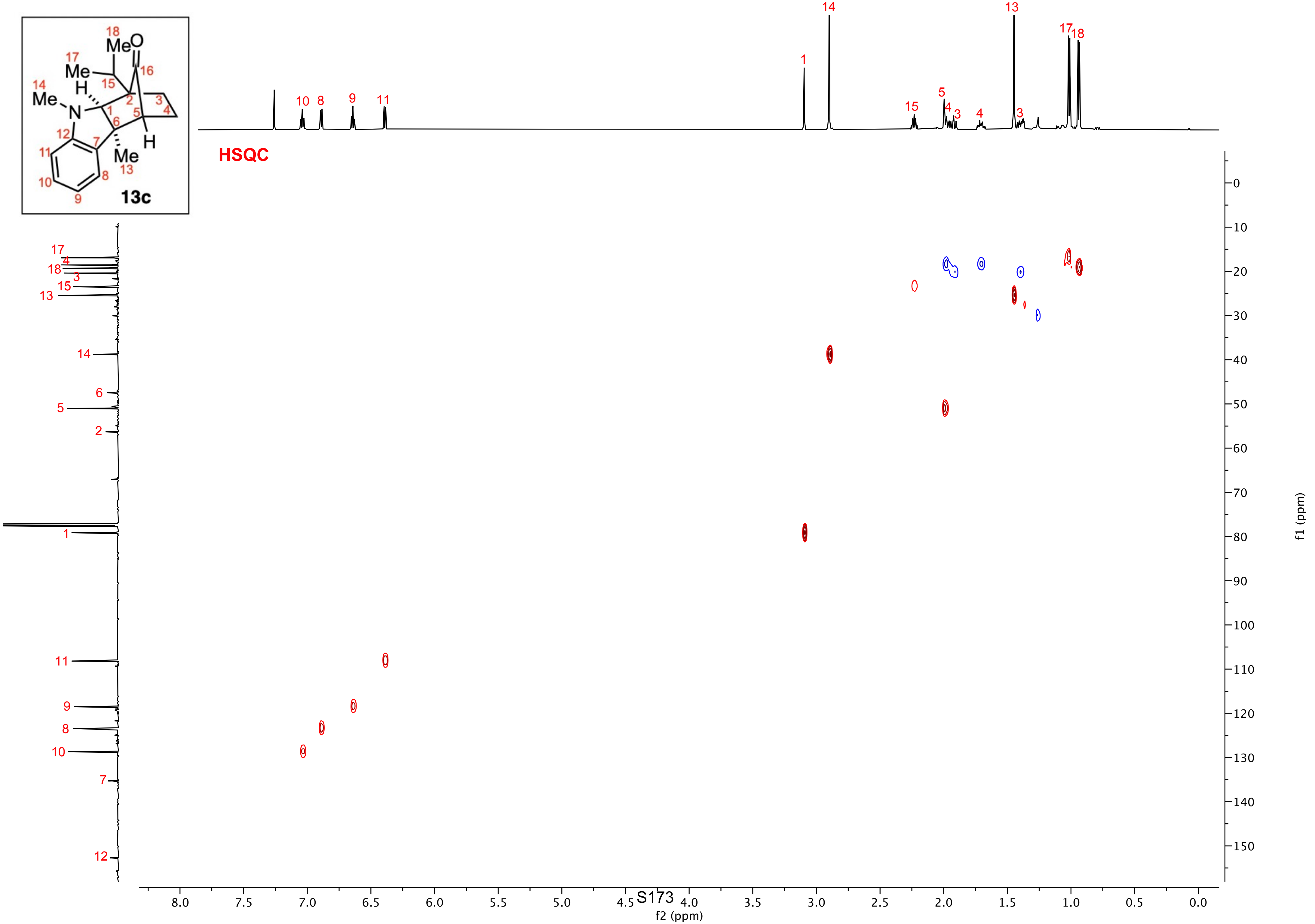
13c
HSQC
f2 (ppm)
f1 (ppm)

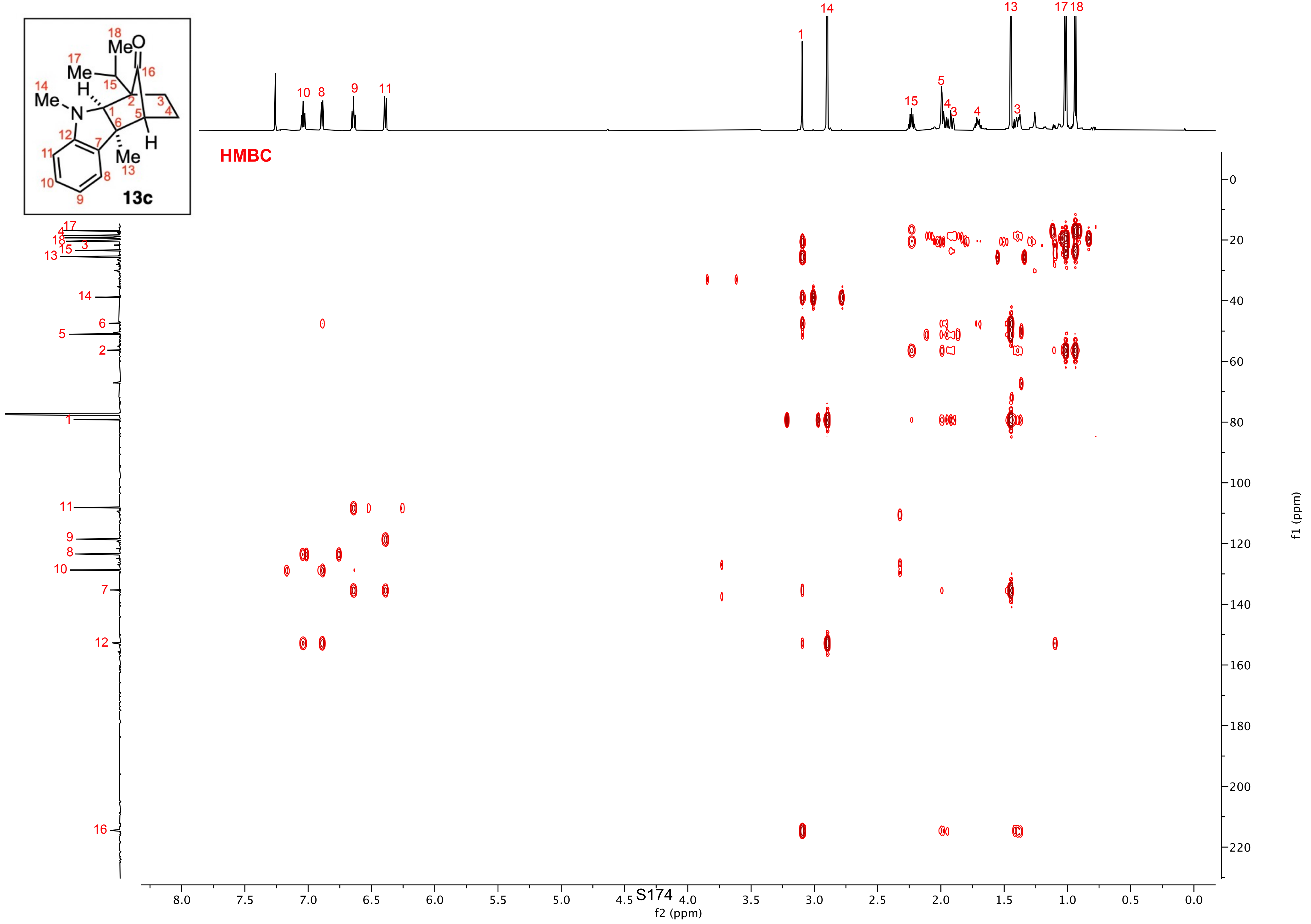
13c
HMBC
f2 (ppm)
f1 (ppm)

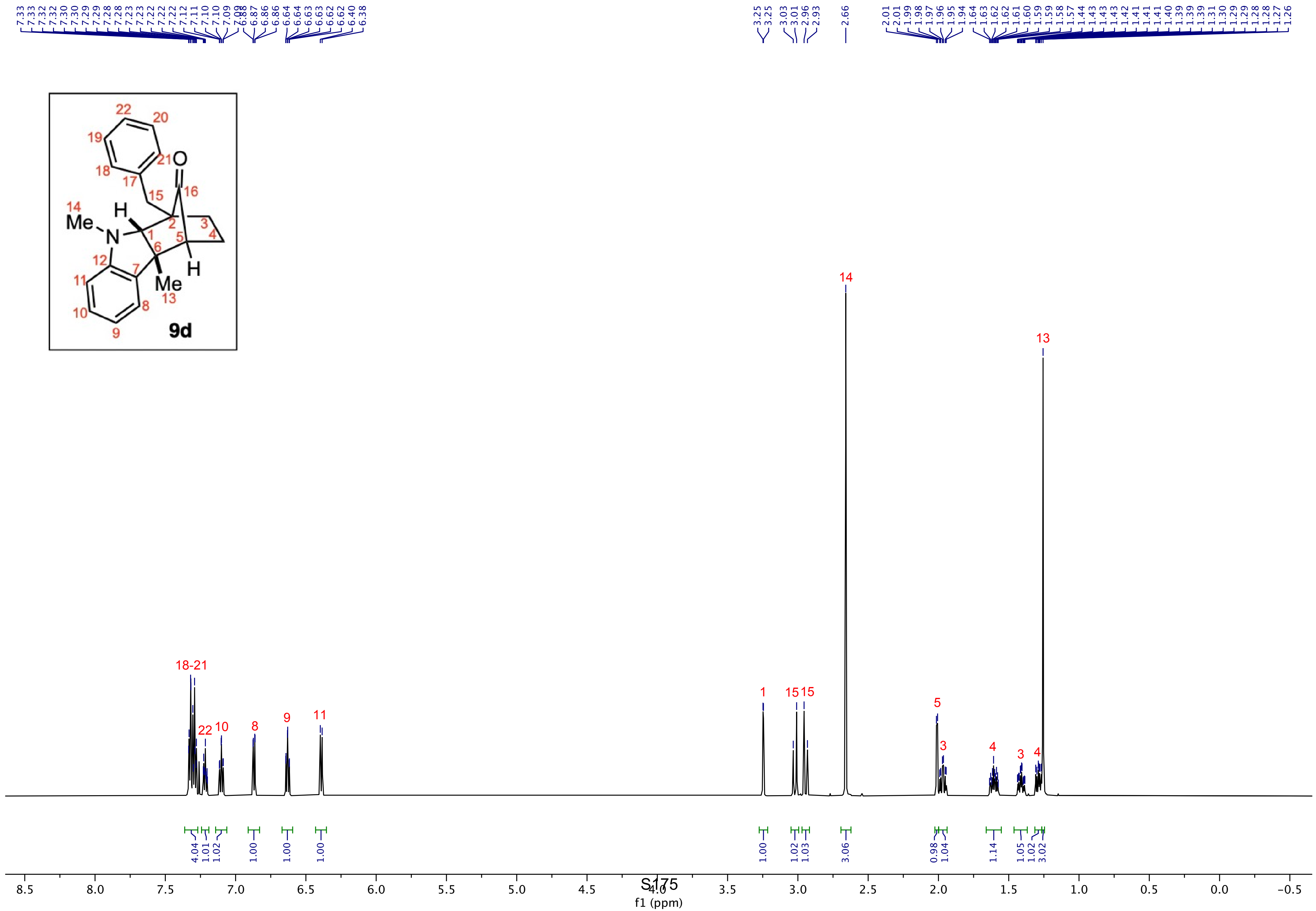
7.33 7.33 7.32 7.32 7.30 7.29 7.29 7.28 7.28 7.23 7.23 7.22 7.22 7.22 7.12 7.11 7.10 7.10 7.09 7.09 6.88 6.87 6.86 6.86 6.64 6.64 6.63 6.63 6.62 6.62 6.40 6.38
3.25 3.25 3.03 3.01 2.96 2.93 2.66
2.01 2.01 1.99 1.98 1.97 1.96 1.95 1.94 1.64 1.63 1.62 1.62 1.61 1.60 1.59 1.59 1.58 1.57 1.44 1.43 1.43 1.42 1.41 1.41 1.40 1.39 1.39 1.39 1.31 1.30 1.29 1.29 1.28 1.28 1.27 1.26
9d
18-21
22
10
8
9
11
1
15
15
14
5
3
4
3
4
13
4.04 1.01 1.02 1.00 1.00 1.00 1.00 1.02 1.03 3.06 0.98 1.04 1.14 1.05 1.02 3.02
8.5 8.0 7.5 7.0 6.5 6.0 5.5 5.0 4.5 4.0 3.5 3.0 2.5 2.0 1.5 1.0 0.5 0.0 −0.5
f1 (ppm)

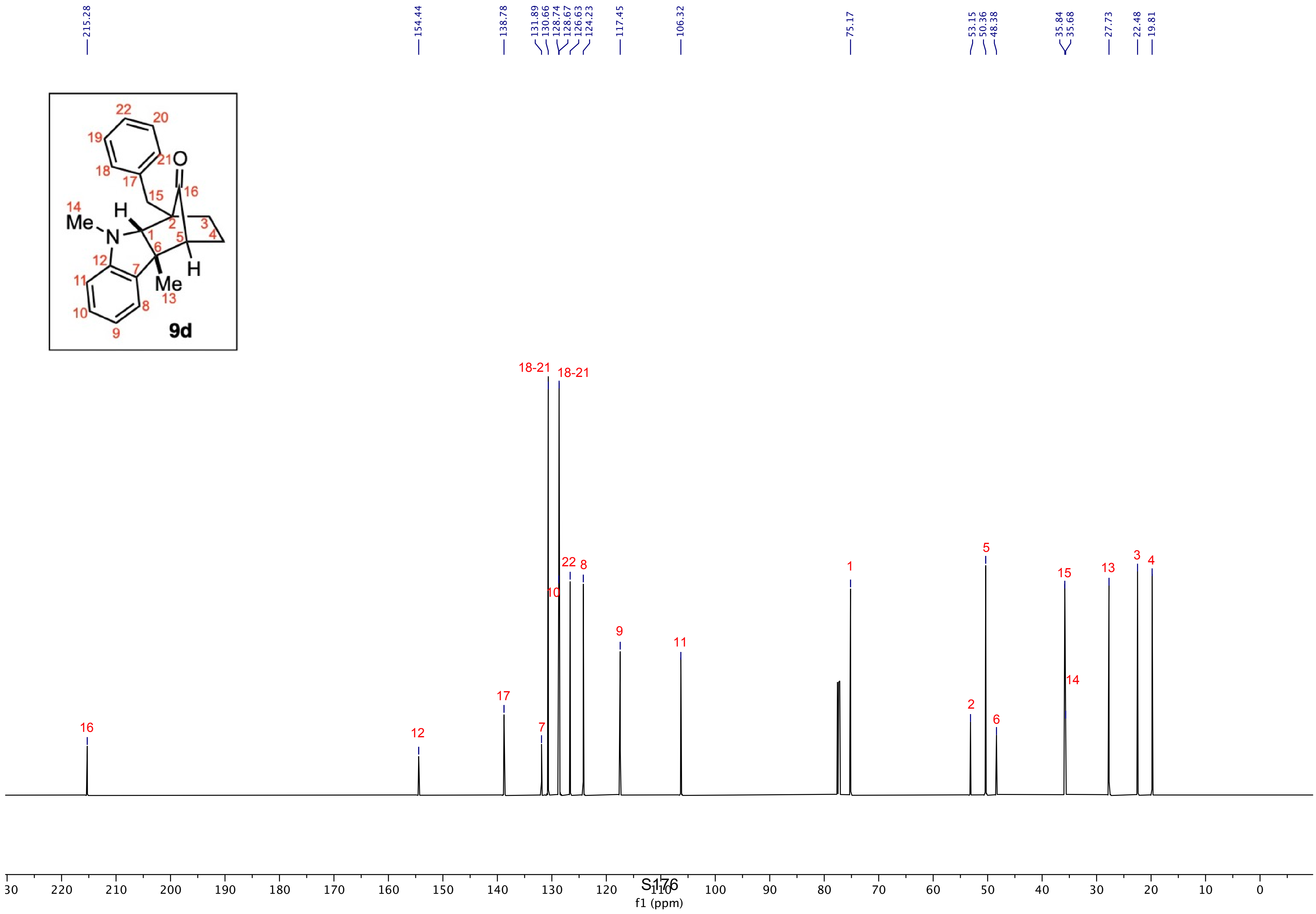
215.28
154.44
138.78
131.89
130.66
128.74
128.67
126.63
124.23
117.45
106.32
75.17
53.15
50.36
48.38
35.84
35.68
27.73
22.48
19.81
9d
Me
N
H
Me
H
O
16
12
17
7
18-21
18-21
10
22
8
9
11
1
2
5
6
15
14
13
3
4
f1 (ppm)

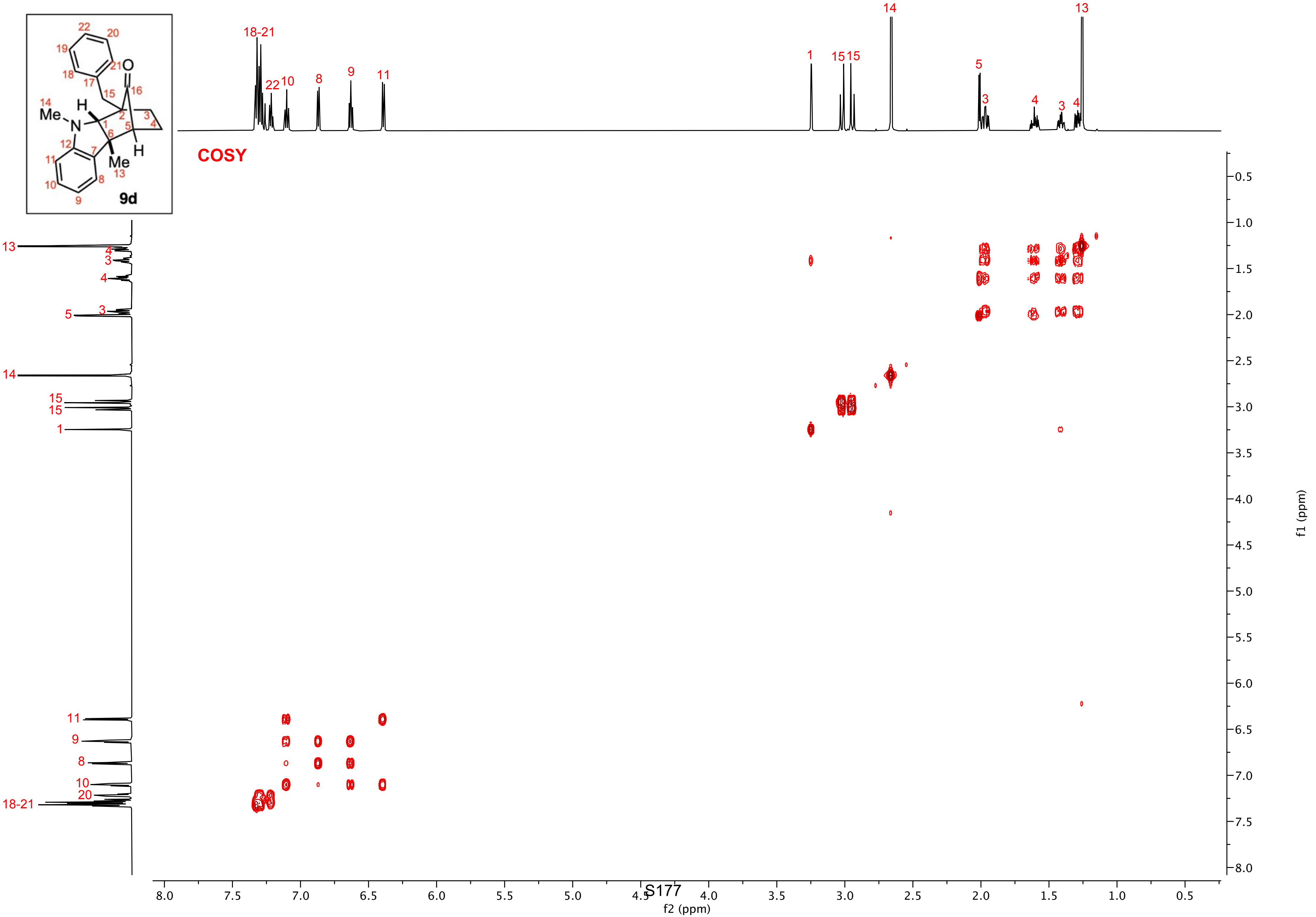

9d
COSY
18-21
22
10
8
9
11
1
15
15
14
5
3
4
3
4
13
f2 (ppm)
f1 (ppm)

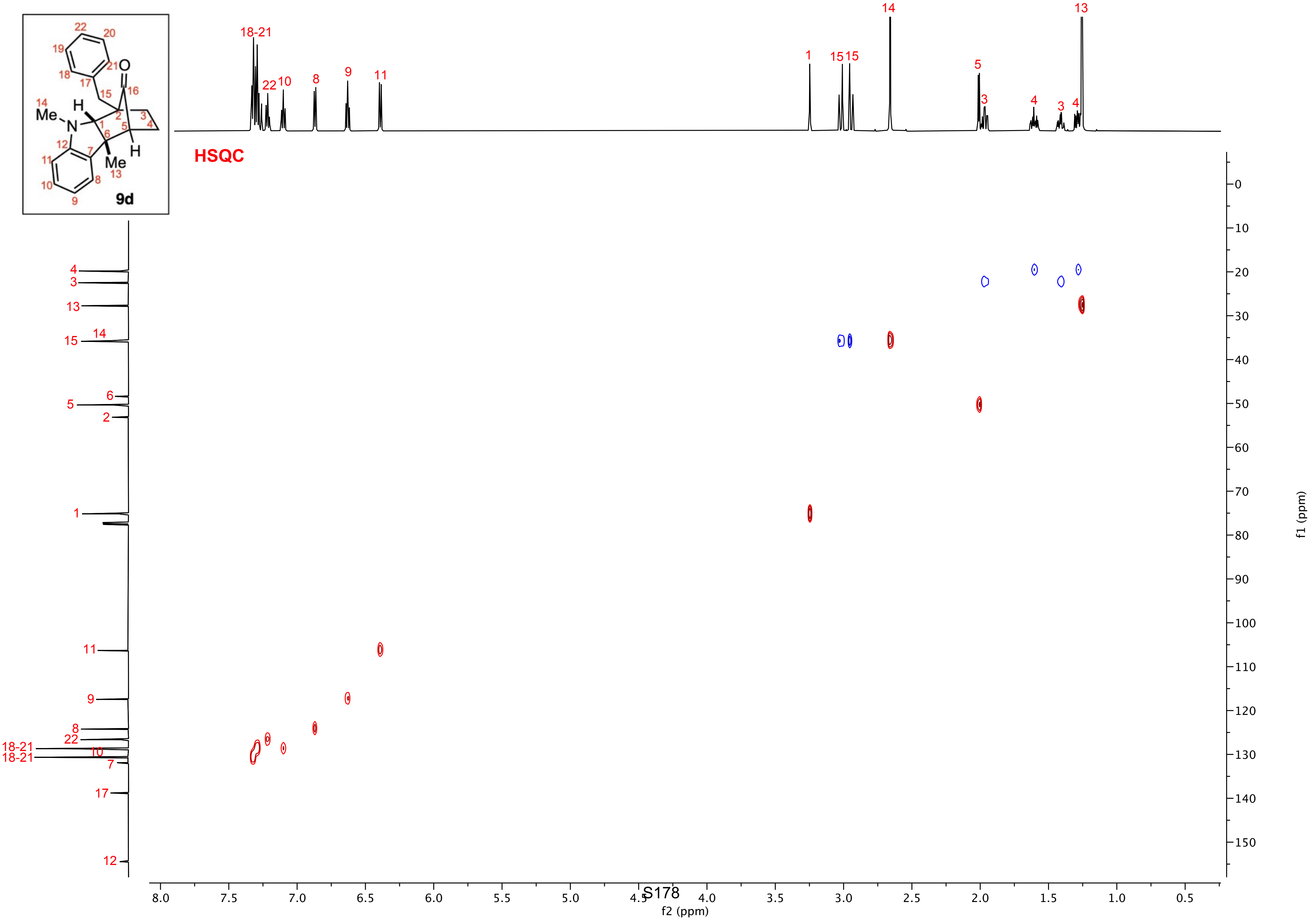

9d
HSQC
18-21
22
10
8
9
11
1
15
15
14
5
3
4
3
4
13
f2 (ppm)
f1 (ppm)

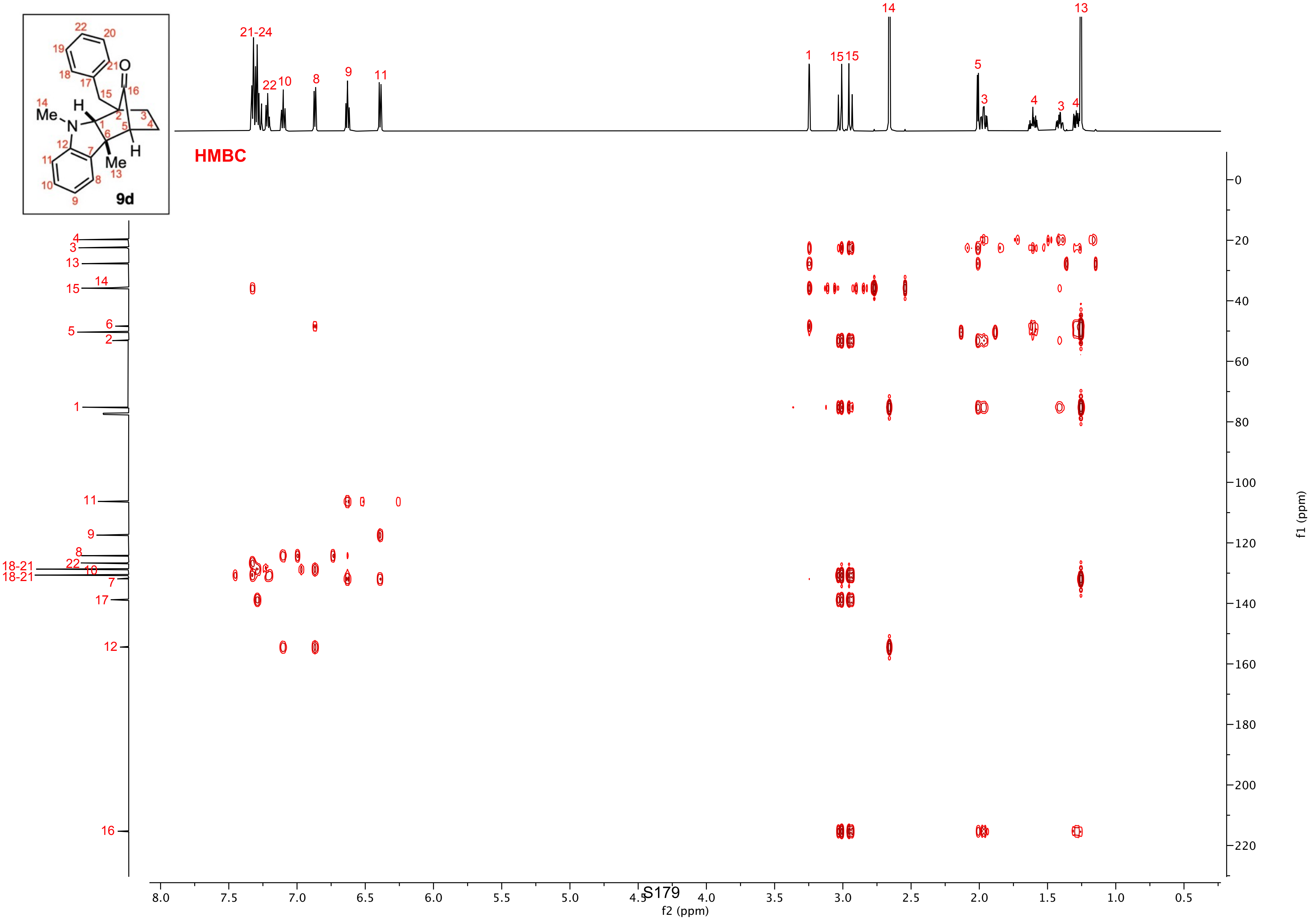
9d
HMBC
f2 (ppm)
f1 (ppm)

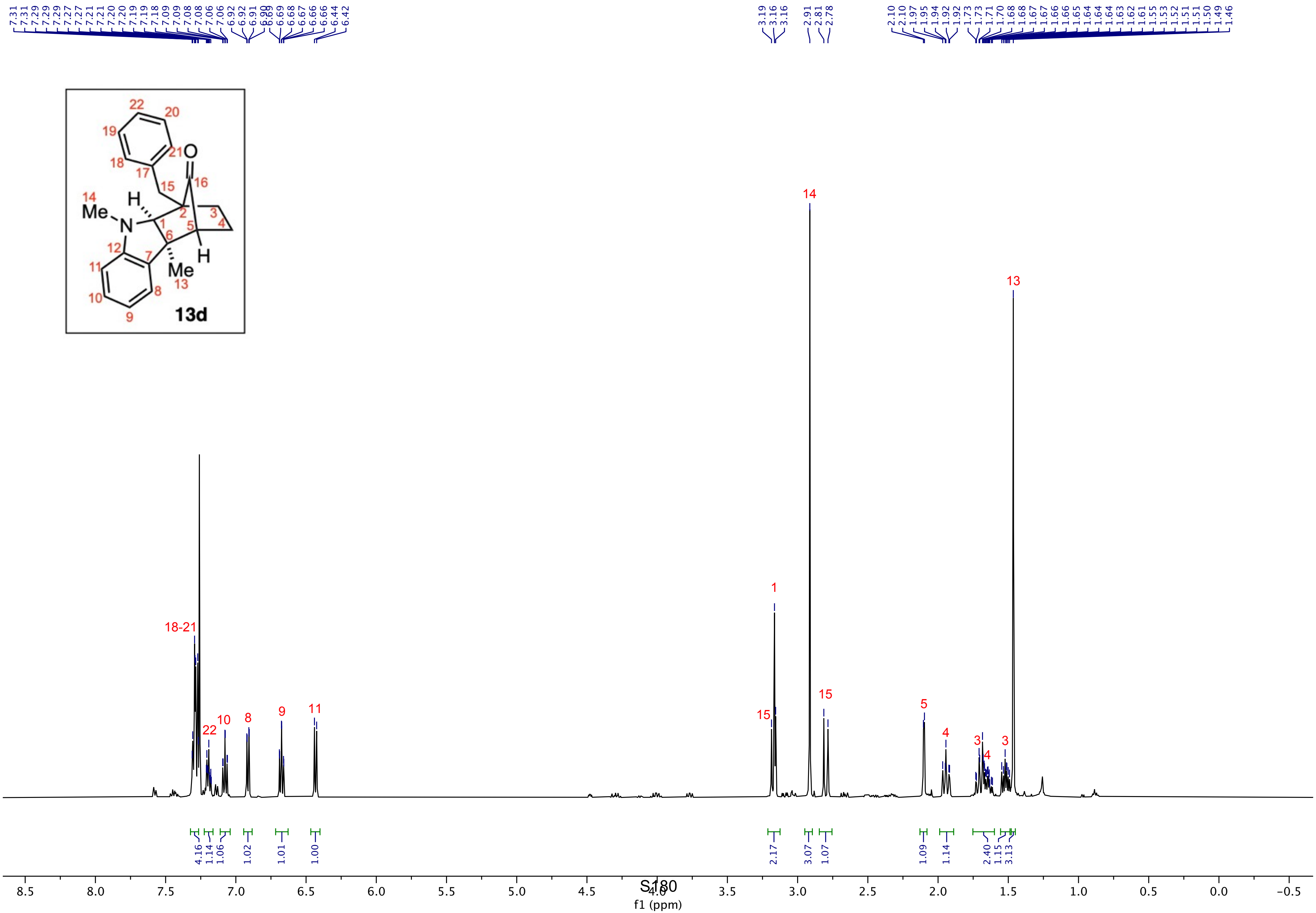
13d
14
13
1
15
15
18-21
22
10
8
9
11
5
4
3
4
3
f1 (ppm)

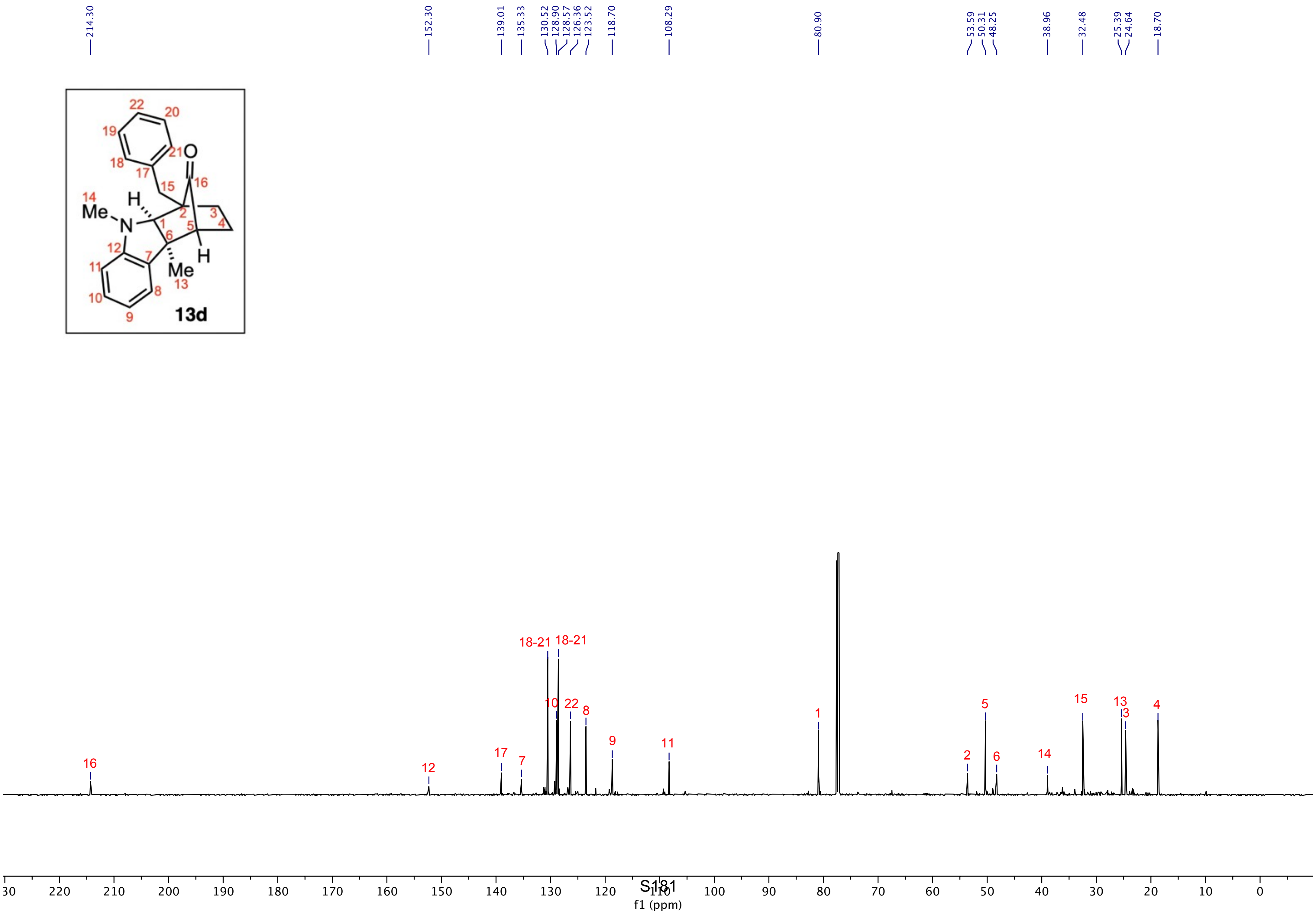

214.30
152.30
139.01
135.33
130.52
128.90
128.57
126.36
123.52
118.70
108.29
80.90
53.59
50.31
48.25
38.96
32.48
25.39
24.64
18.70
13d
16
12
17
7
18-21
18-21
10
22
8
9
11
1
2
5
6
14
15
13
3
4
f1 (ppm)

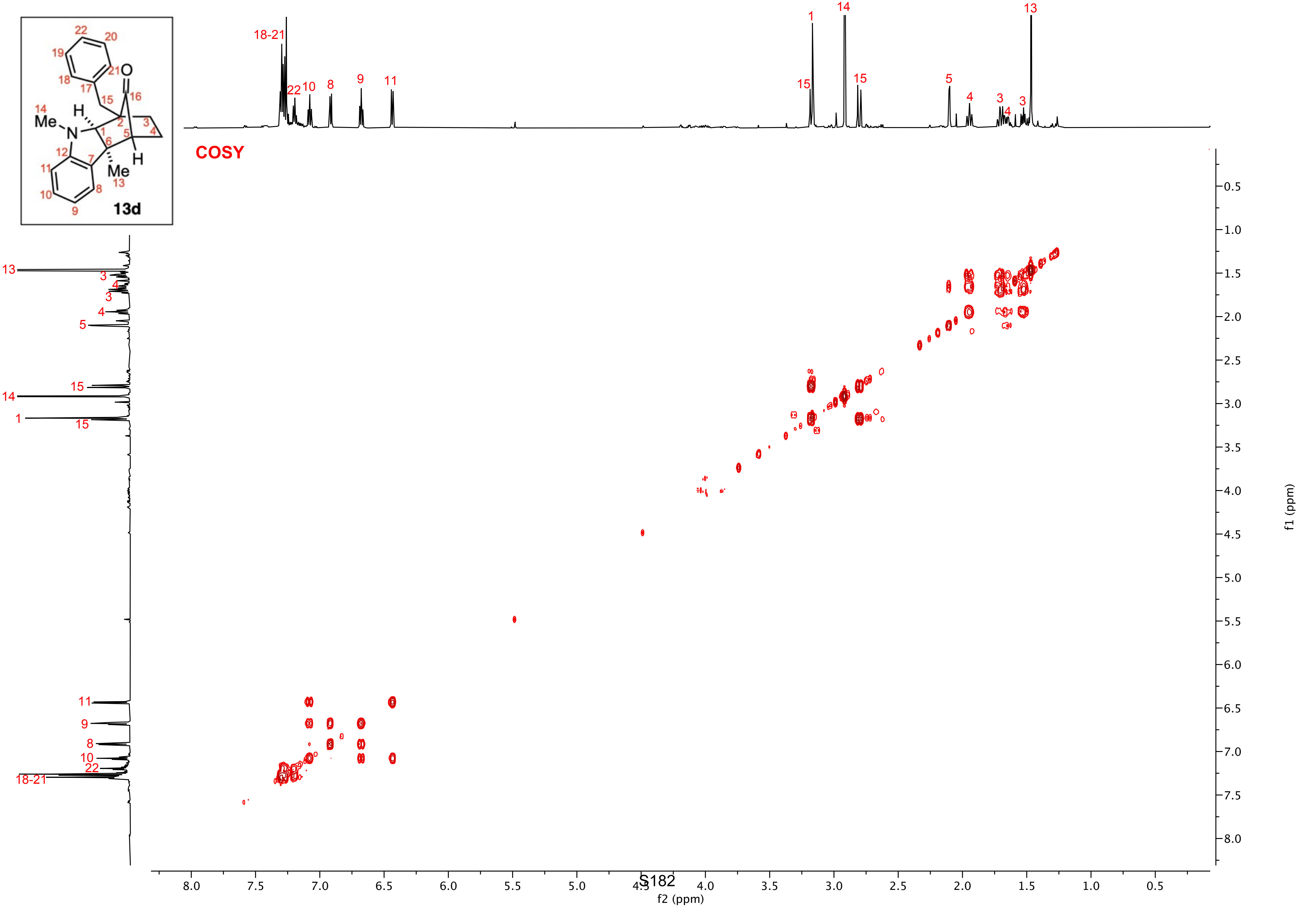

13d
COSY
18-21
22
10
8
9
11
15
1
14
5
4
3
13
f2 (ppm)
f1 (ppm)

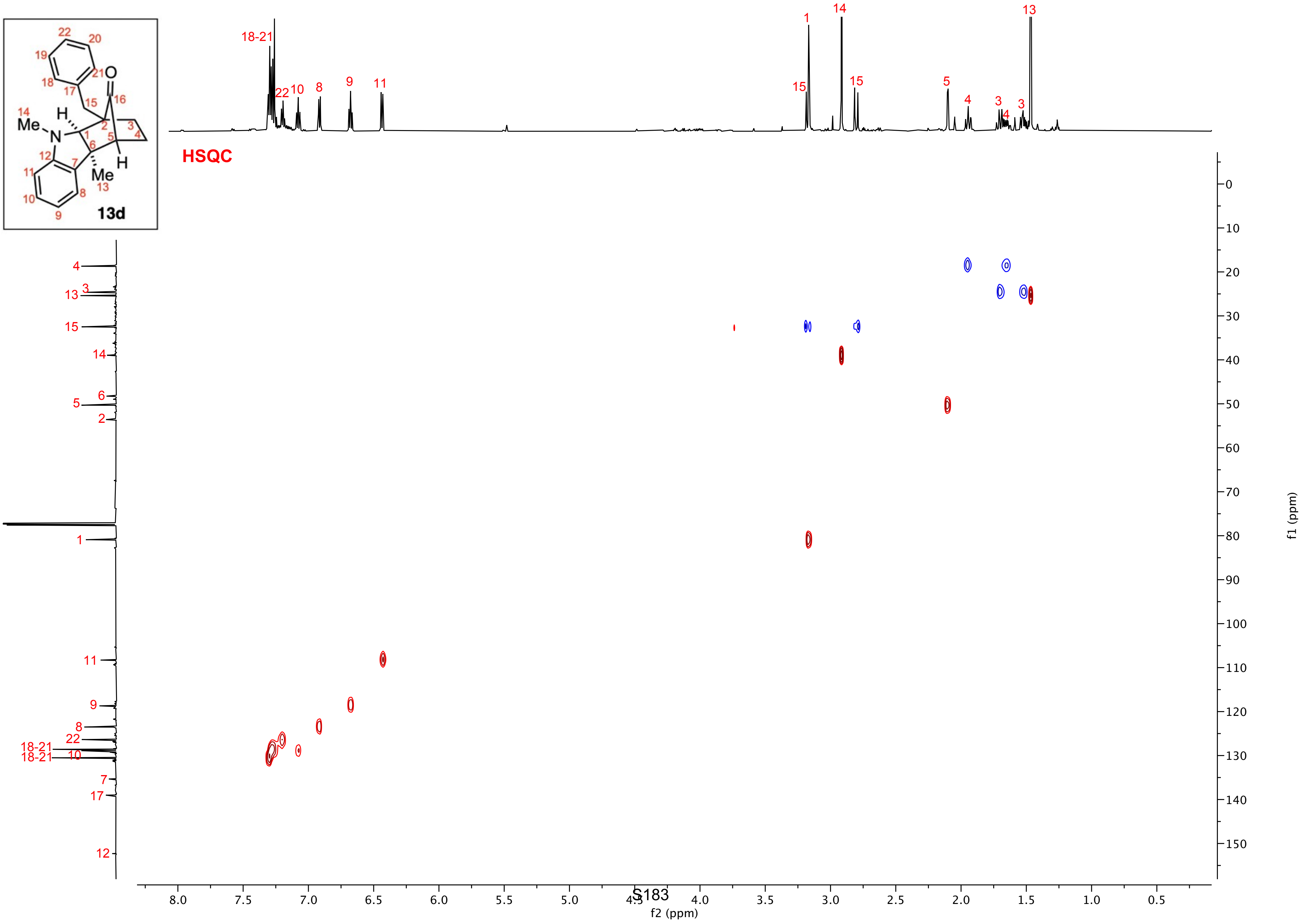
13d
HSQC
f2 (ppm)
f1 (ppm)

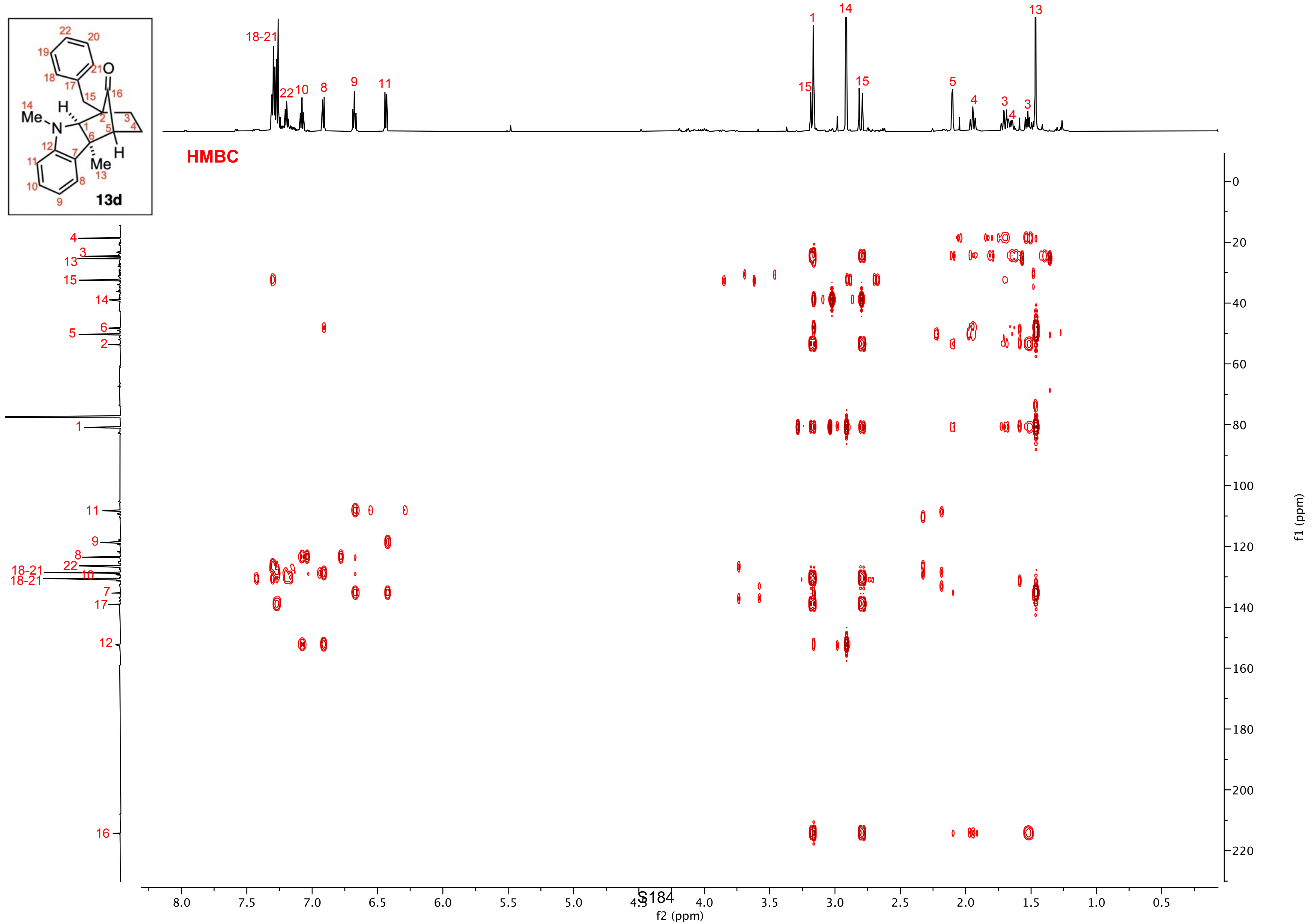

13d
HMBC
f2 (ppm)
f1 (ppm)

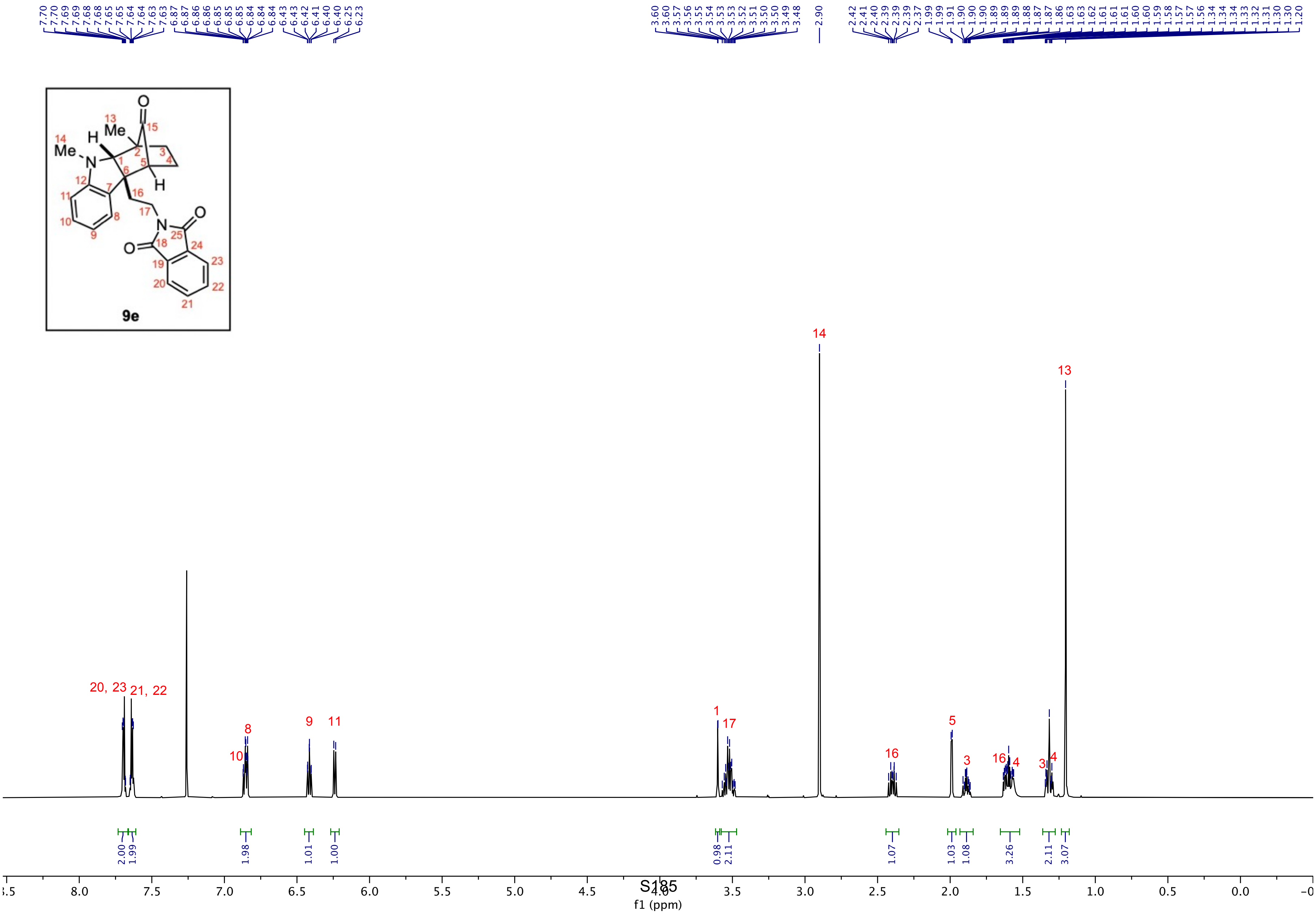

9e
14
13
20, 23
21, 22
10
8
9
11
1
17
16
5
3
16
4
3
4
2.00
1.99
1.98
1.01
1.00
0.98
2.11
1.07
1.03
1.08
3.26
2.11
3.07
f1 (ppm)

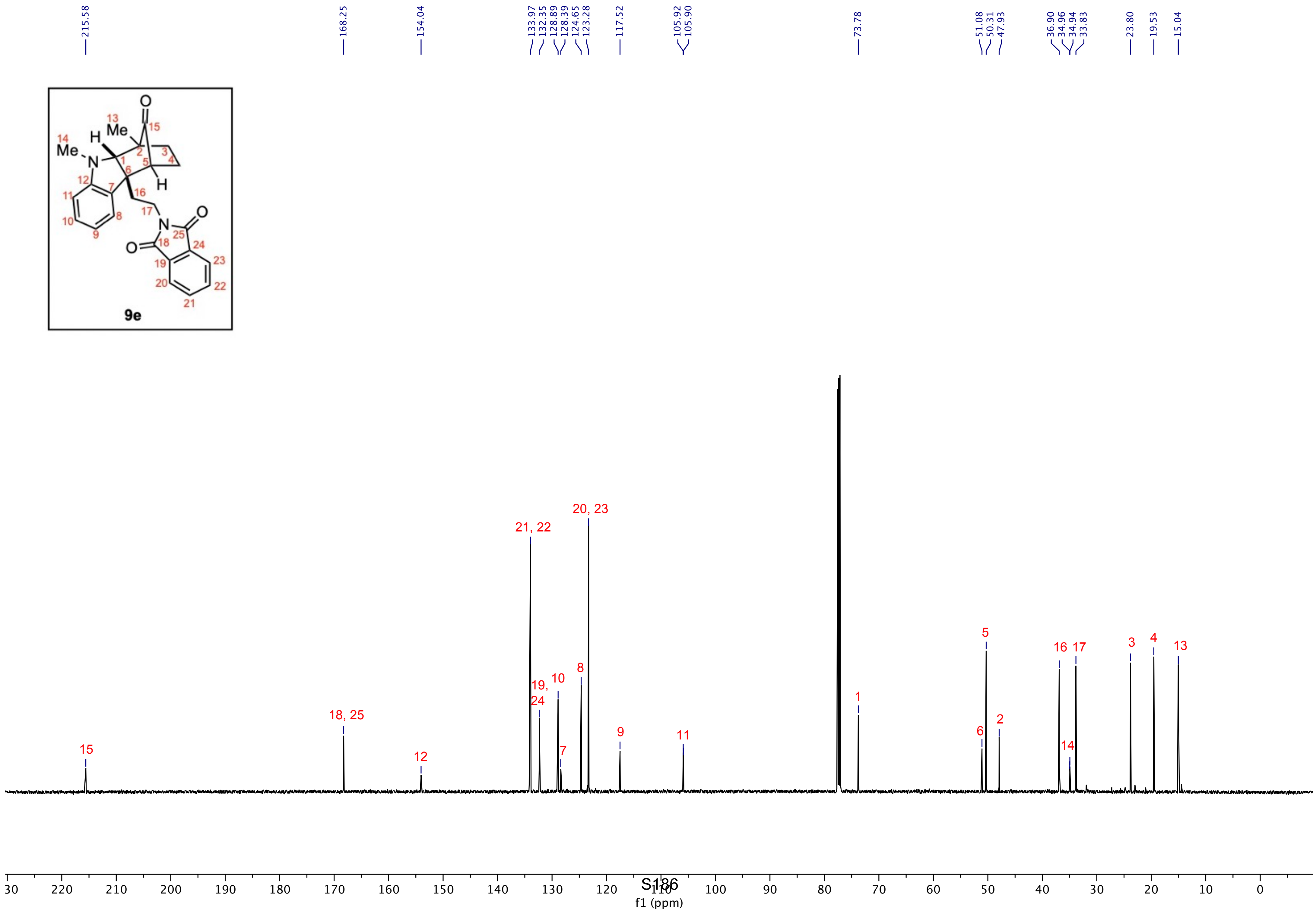
215.58
168.25
154.04
133.97
132.35
128.89
128.39
124.65
123.28
117.52
105.92
105.90
73.78
51.08
50.31
47.93
36.90
34.96
34.94
33.83
23.80
19.53
15.04
9e
15
18, 25
12
21, 22
19, 24
10
7
8
20, 23
9
11
1
6
5
2
16 17
14
3
4
13
f1 (ppm)

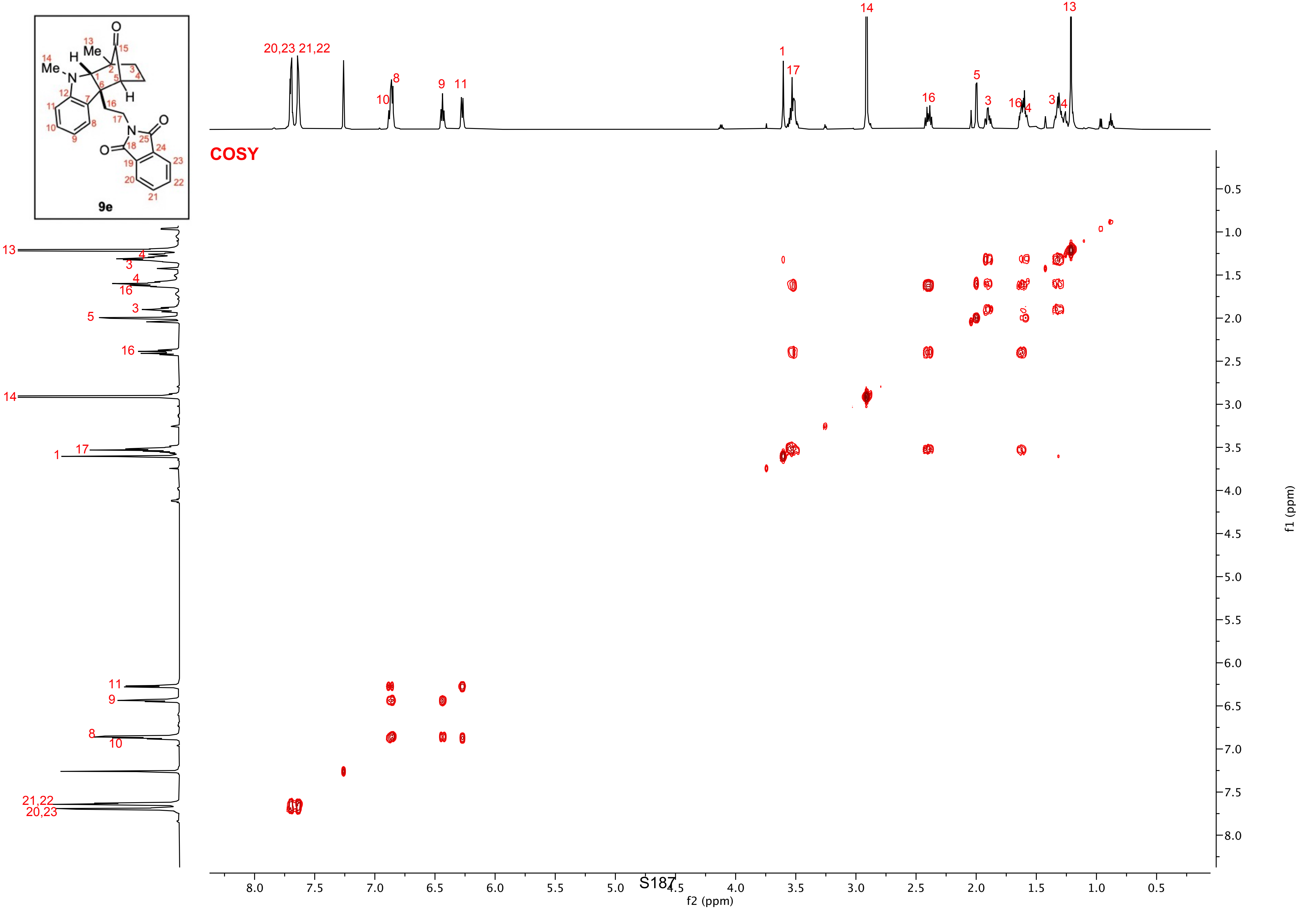
9e
COSY
20,23 21,22
10
8
9
11
1
17
14
16
5
3
16
4
3
4
13
f2 (ppm)
f1 (ppm)

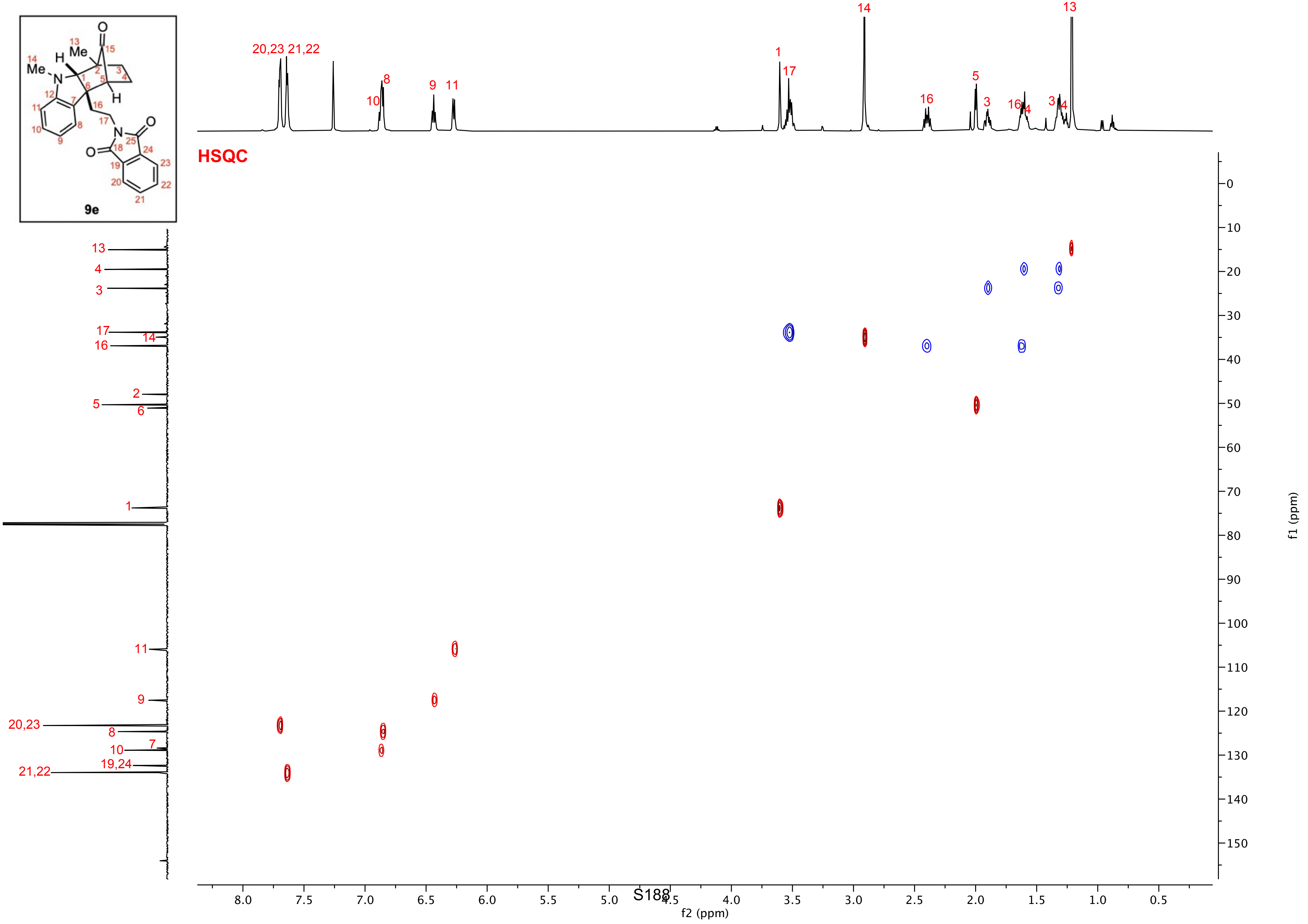
9e
HSQC
20,23 21,22
1
17
14
5
16
3
13
10
8
9
11
f2 (ppm)
f1 (ppm)
19,24
7
2
6
4

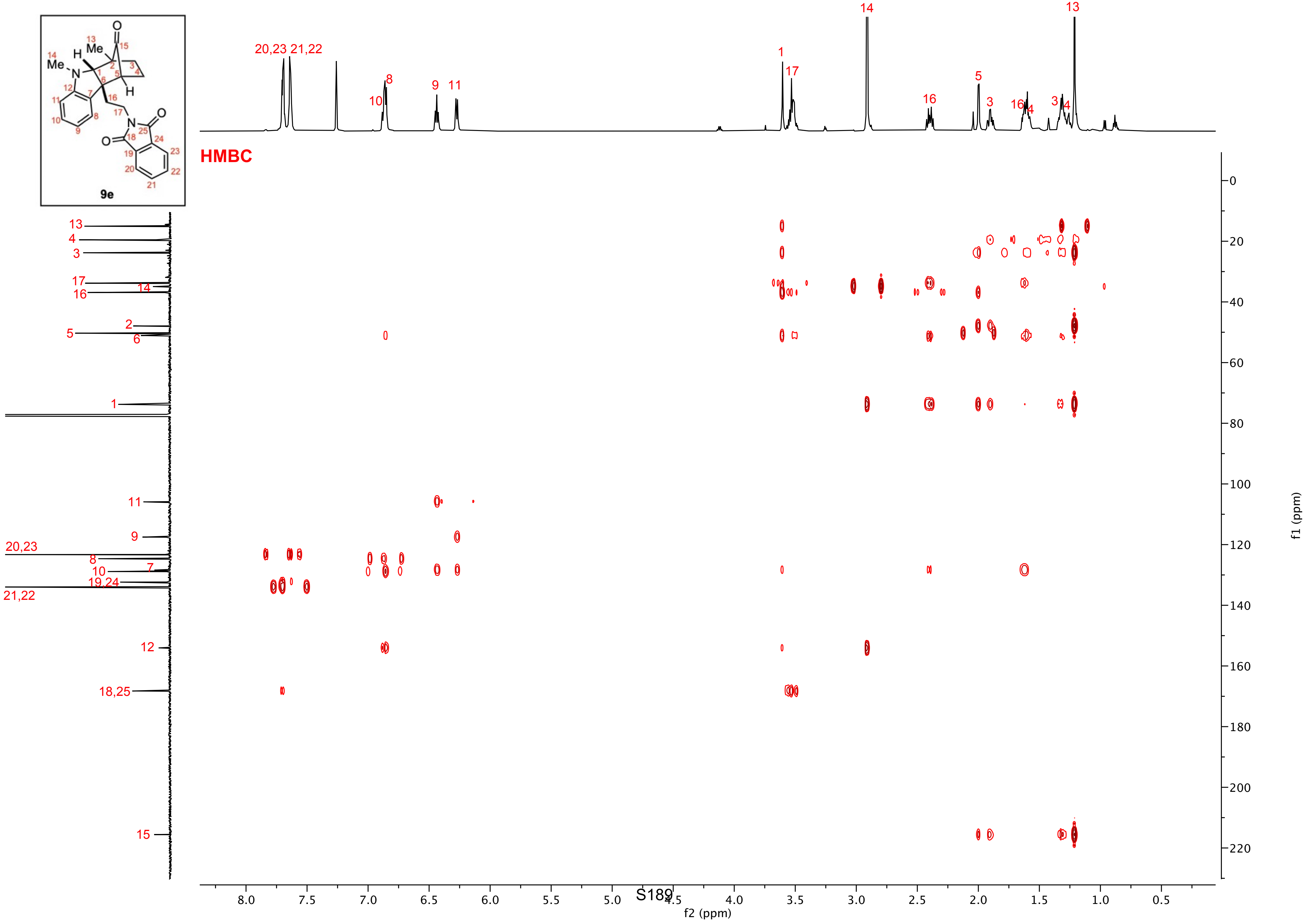
9e
HMBC
20,23 21,22
14
13
1
17
10
8
9
11
16
5
3
16
4
3
4
13
4
3
17
14
16
2
5
6
1
11
9
20,23
8
10
7
19,24
21,22
12
18,25
15
f2 (ppm)
f1 (ppm)

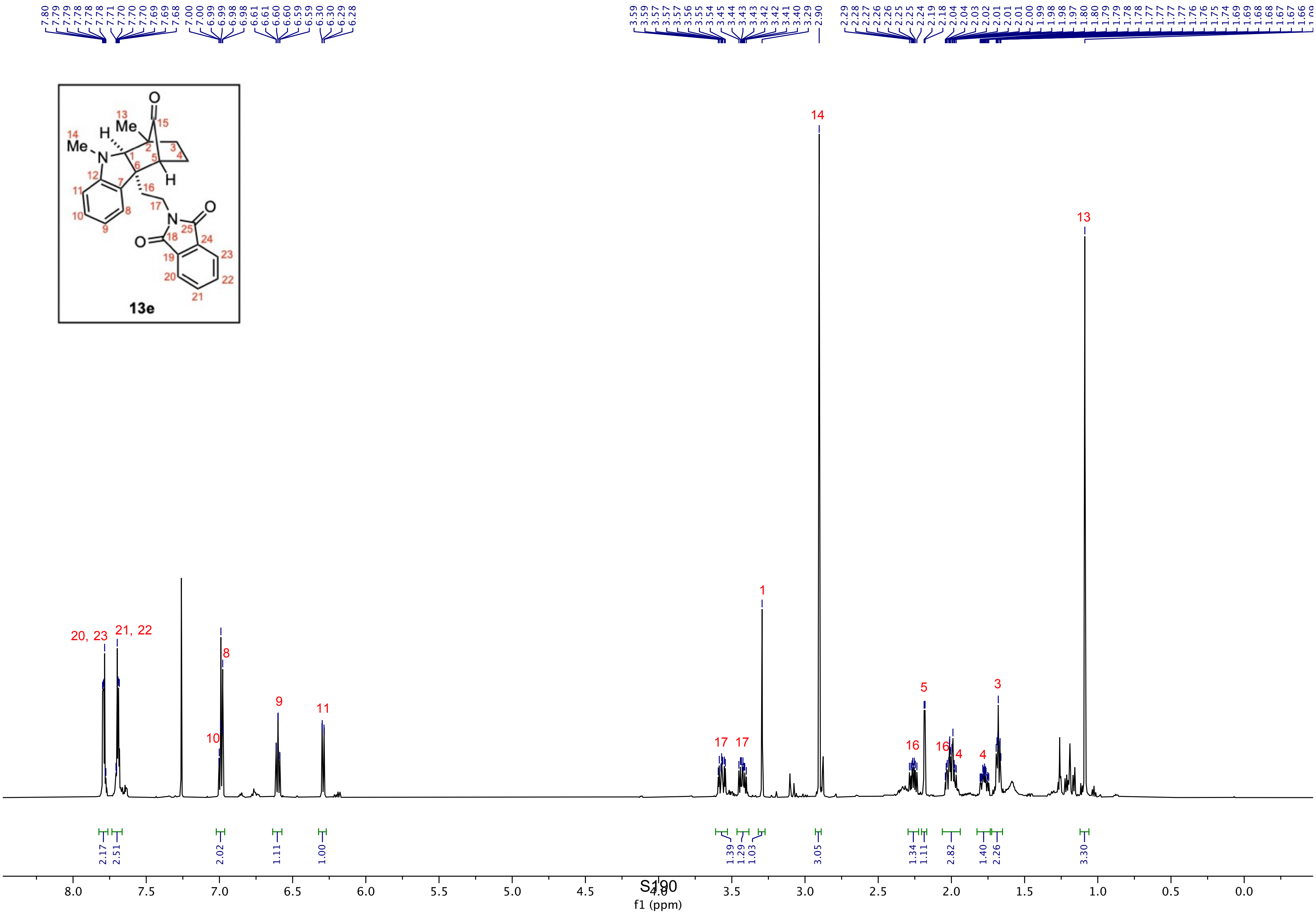
13e
20, 23
21, 22
10
8
9
11
17
17
1
14
16
5
16
4
4
3
13
2.17
2.51
2.02
1.11
1.00
1.39
1.29
1.03
3.05
1.34
1.11
2.82
1.40
2.26
3.30
f1 (ppm)

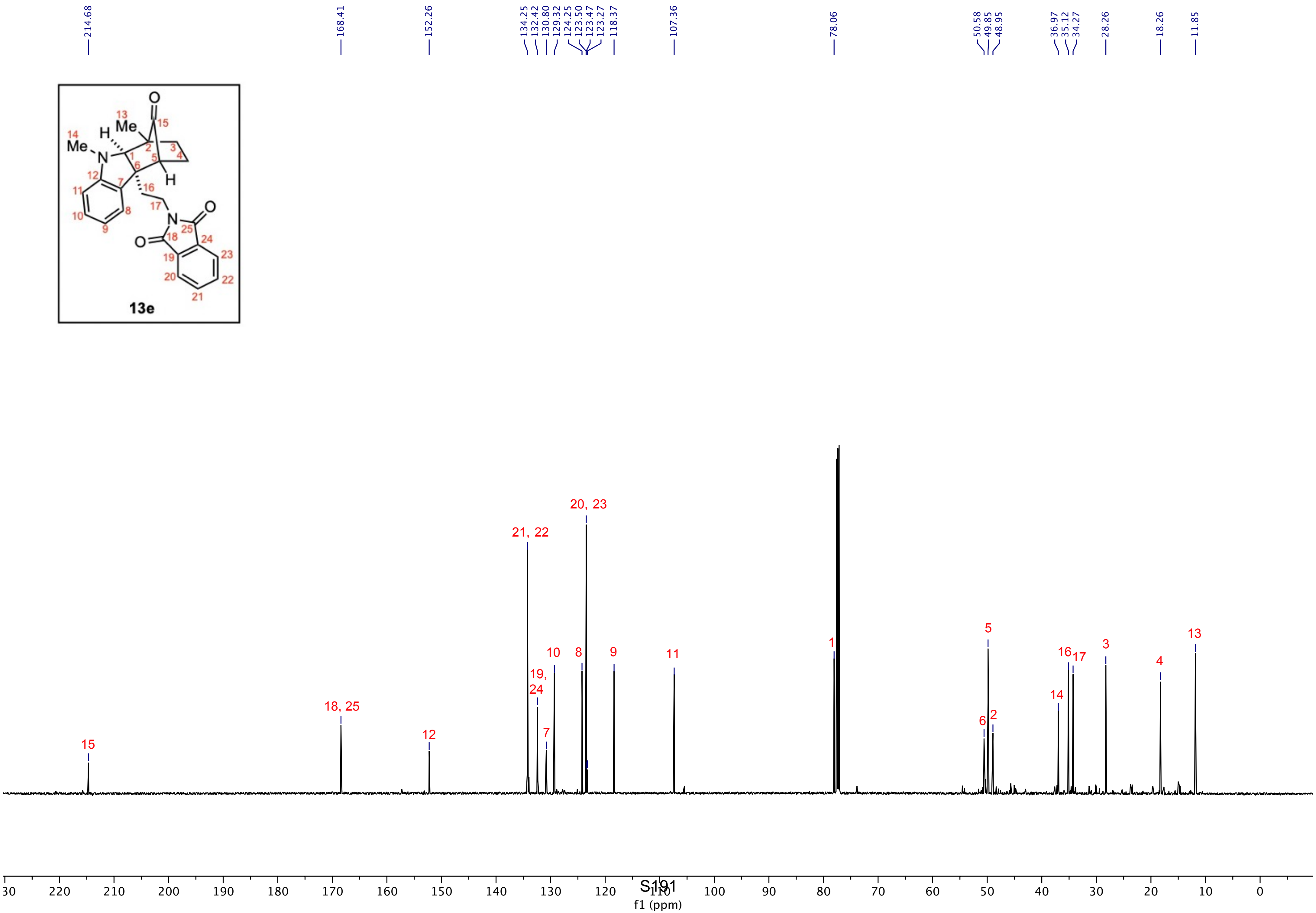

214.68
168.41
152.26
134.25
132.42
130.80
129.32
124.25
123.50
123.47
123.27
118.37
107.36
78.06
50.58
49.85
48.95
36.97
35.12
34.27
28.26
18.26
11.85
13e
15
18, 25
12
21, 22
19, 24
7
10
8
20, 23
9
11
1
6
5
2
14
16
17
3
4
13
f1 (ppm)

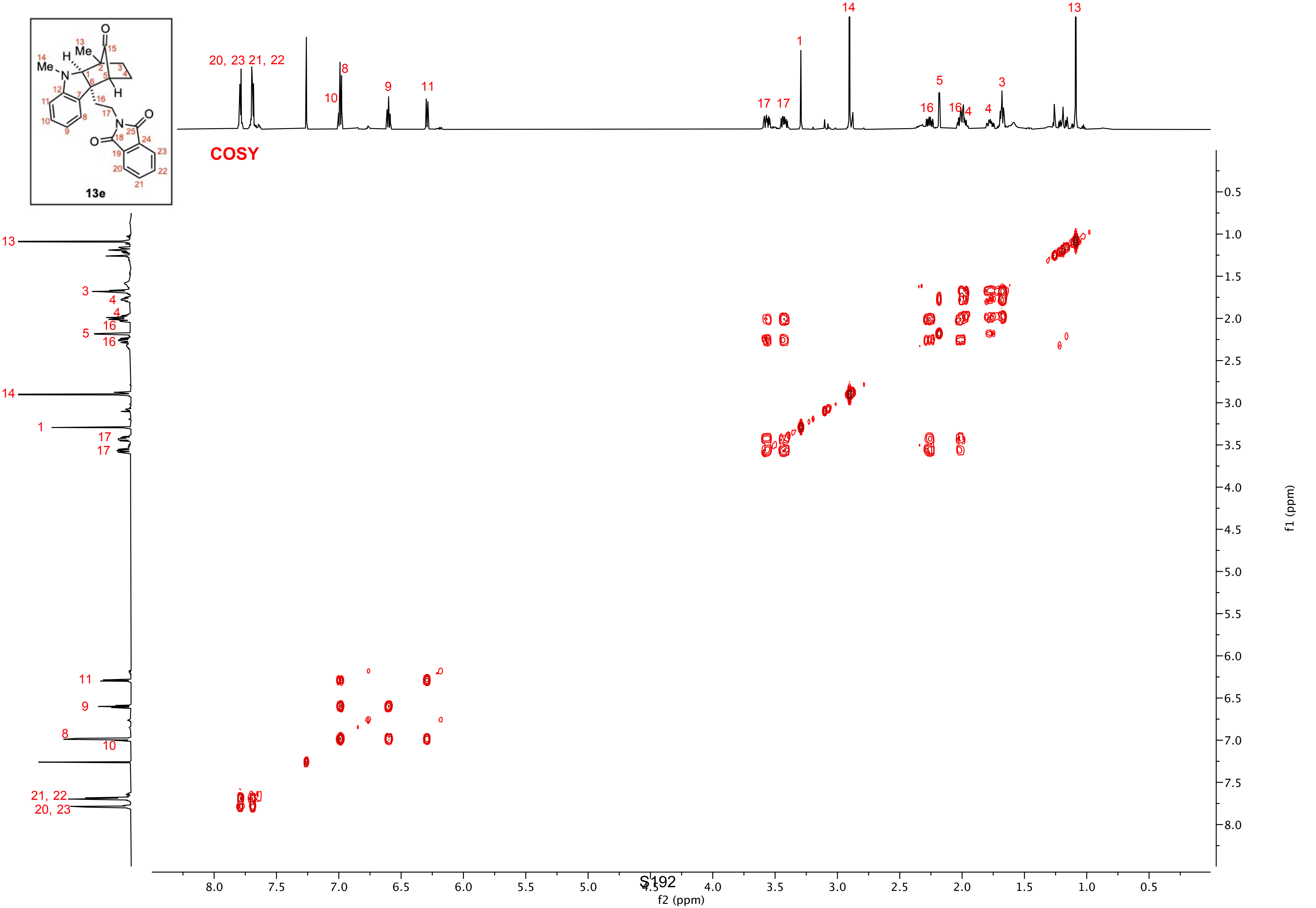

13e
COSY
20, 23 21, 22
10
8
9
11
17 17
1
14
16
5
16
4
4
3
13
f1 (ppm)
f2 (ppm)

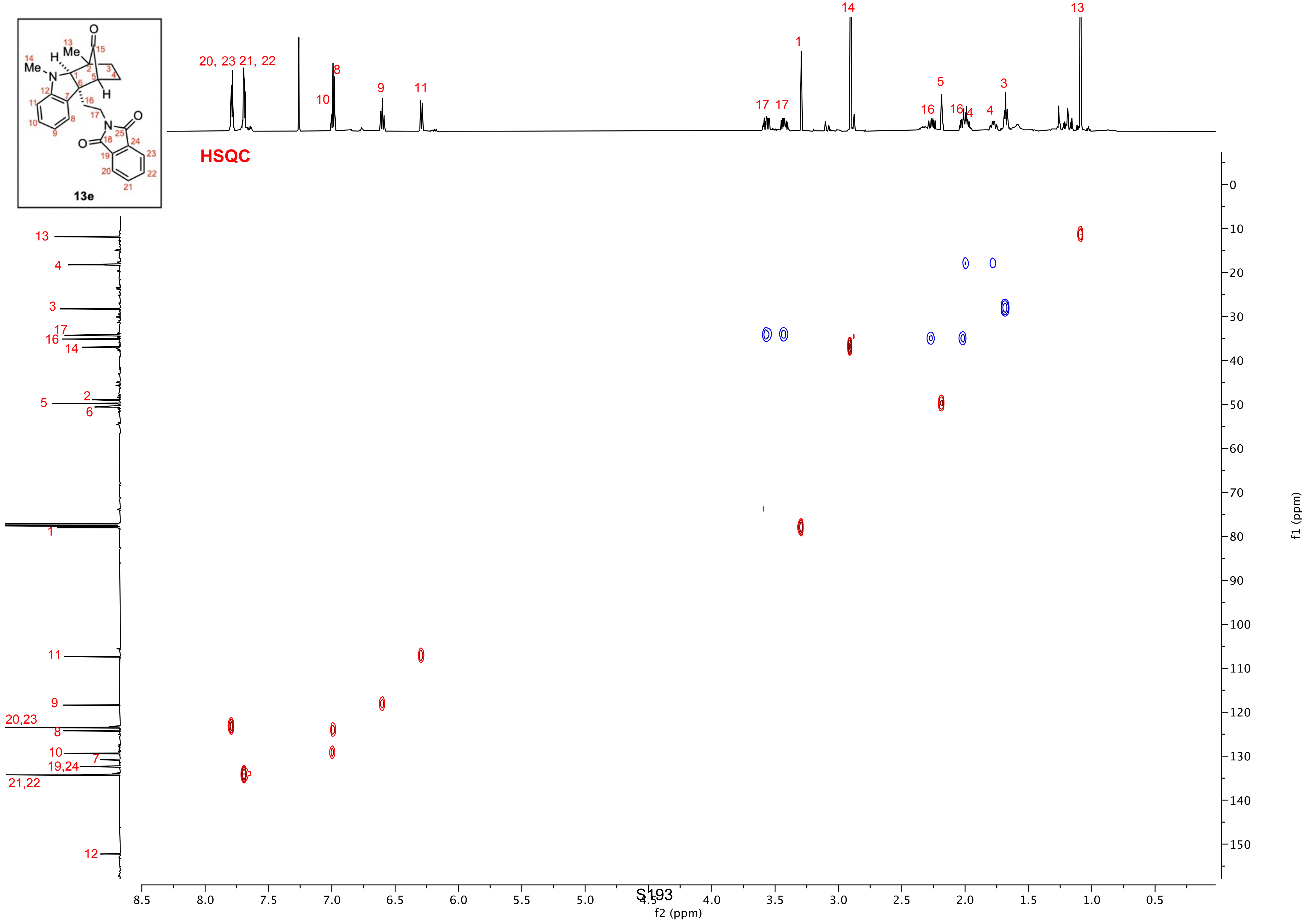

13e
HSQC
20, 23
21, 22
10
8
9
11
1
14
13
17
17
16
16
4
5
4
3
f2 (ppm)
f1 (ppm)
20,23
19,24
21,22
12

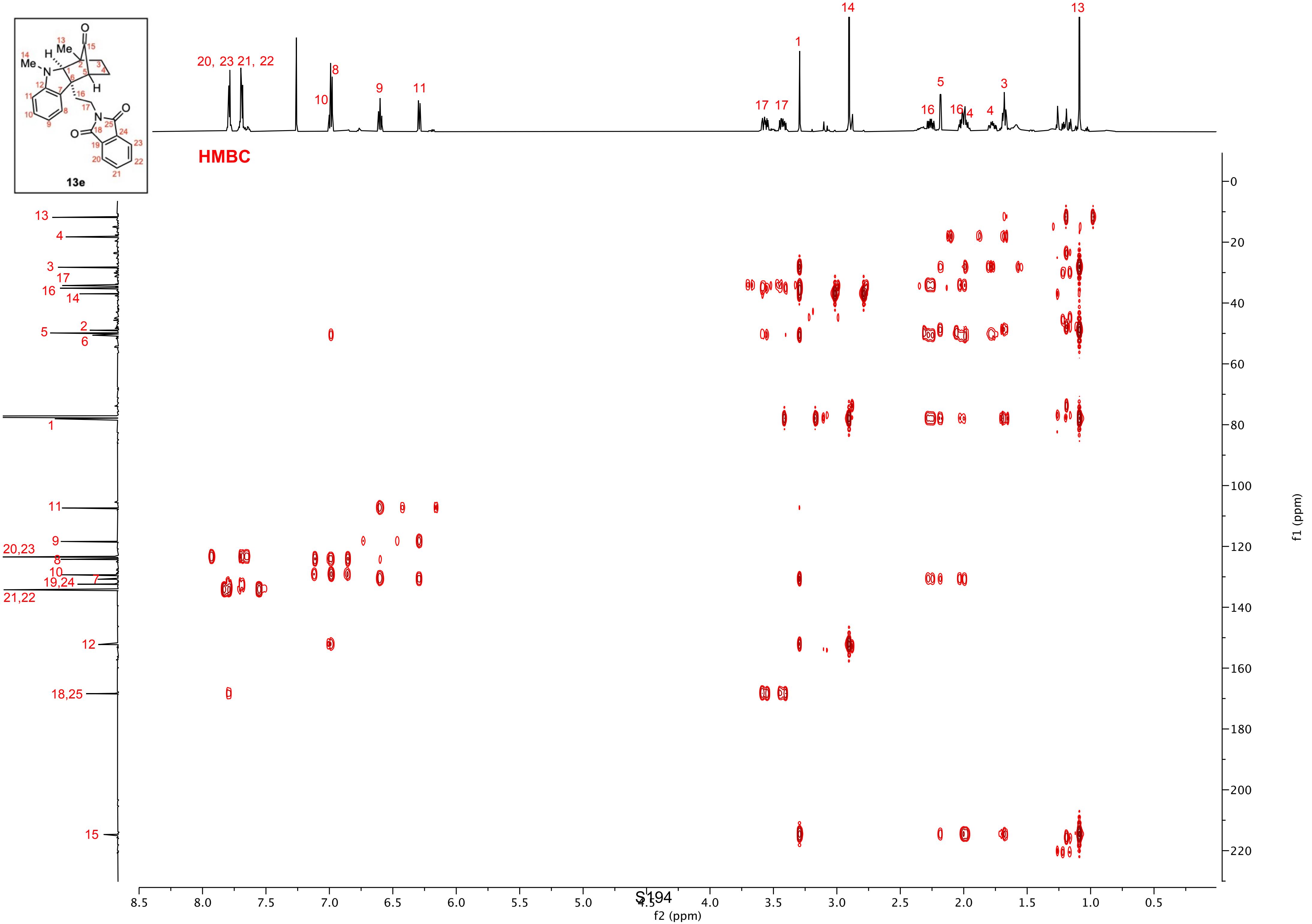
13e
HMBC
20, 23
21, 22
10
8
9
11
17
1
14
16
5
3
4
13
2
6
19,24
7
12
18,25
15
f2 (ppm)
f1 (ppm)

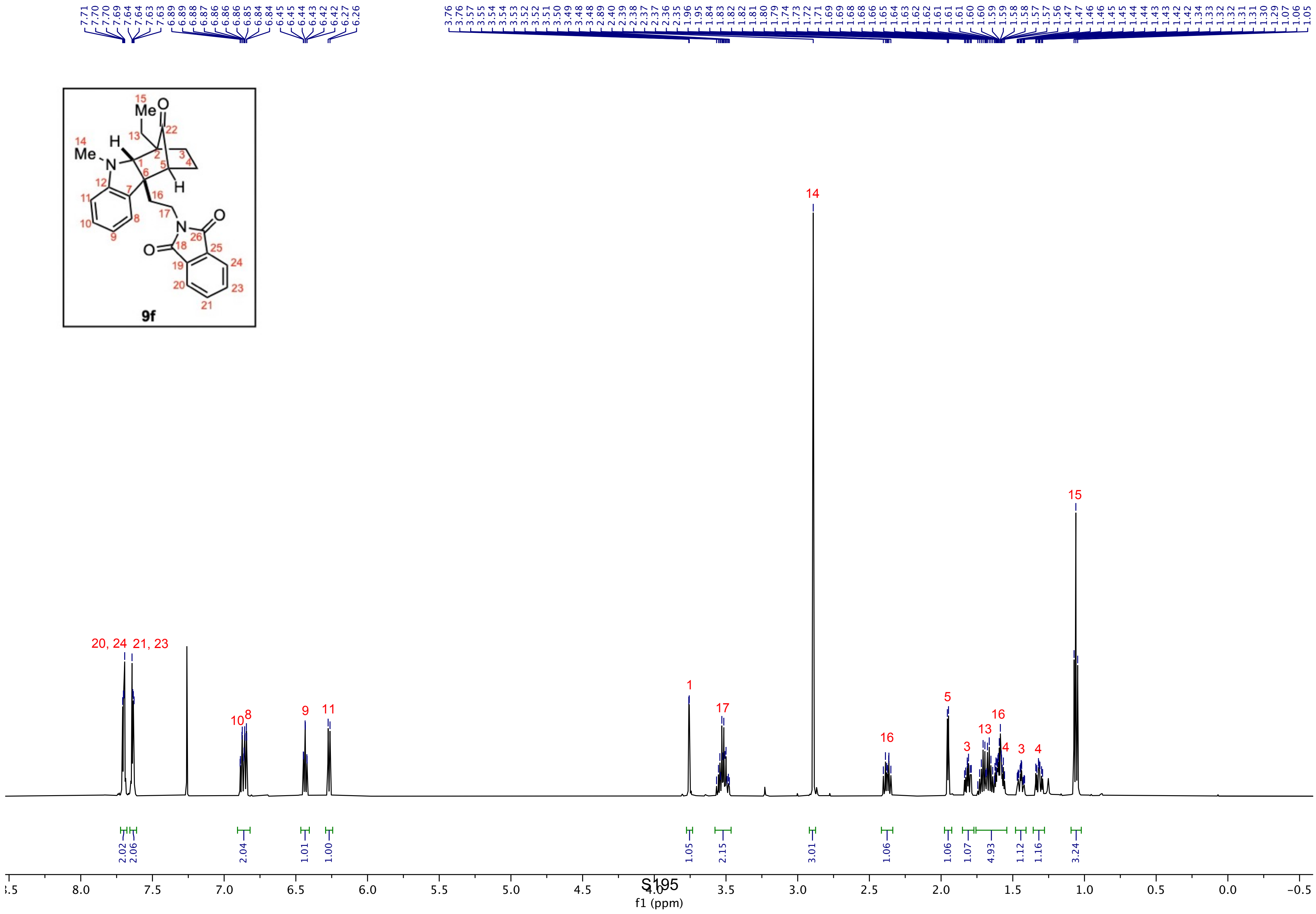
9f
20, 24
21, 23
f1 (ppm)

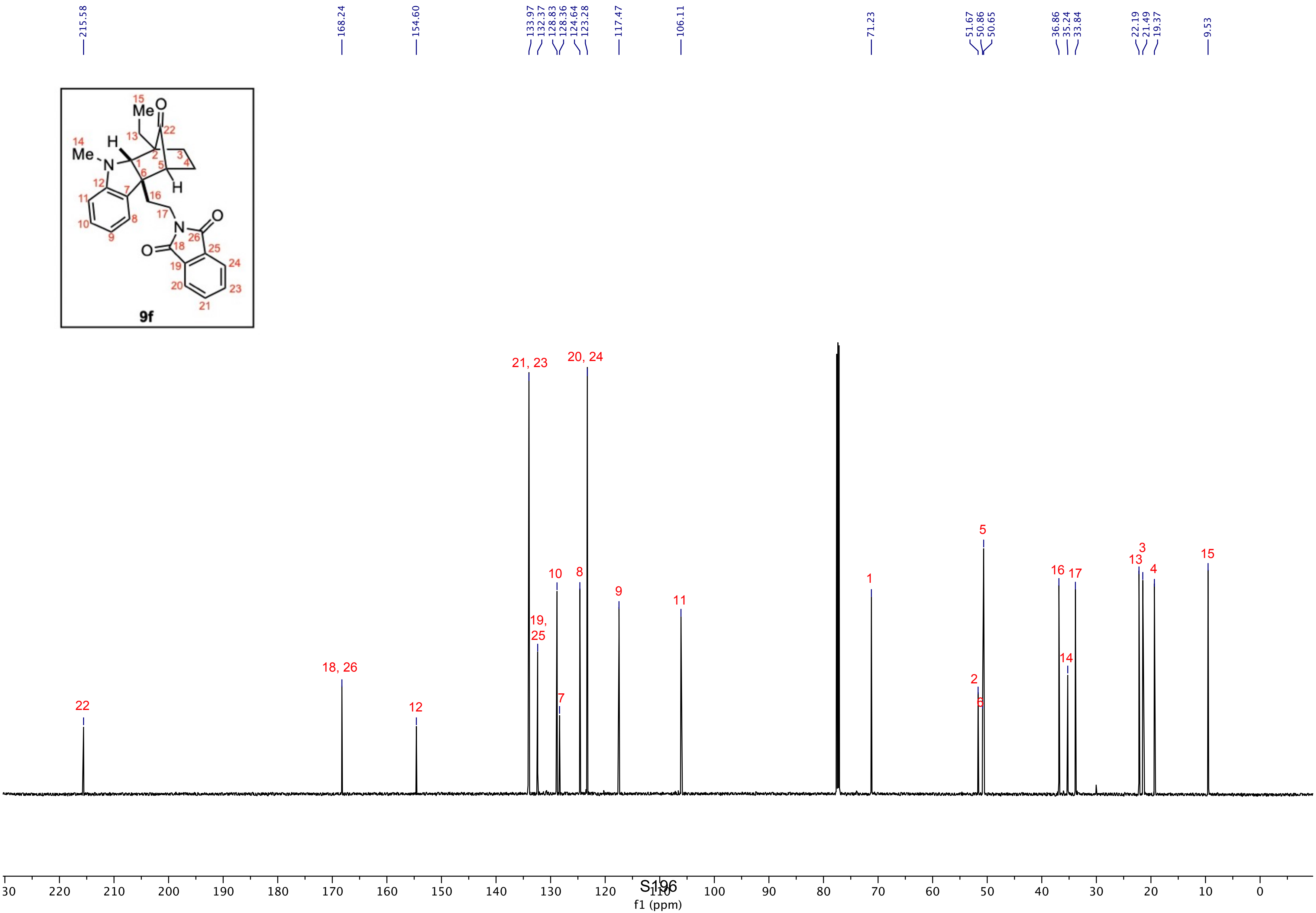
215.58
168.24
154.60
133.97
132.37
128.83
128.36
124.64
123.28
117.47
106.11
71.23
51.67
50.86
50.65
36.86
35.24
33.84
22.19
21.49
19.37
9.53
9f
22
18, 26
12
21, 23
19, 25
10
7
8
20, 24
9
11
1
2
6
5
16
14
17
13
3
4
15
f1 (ppm)

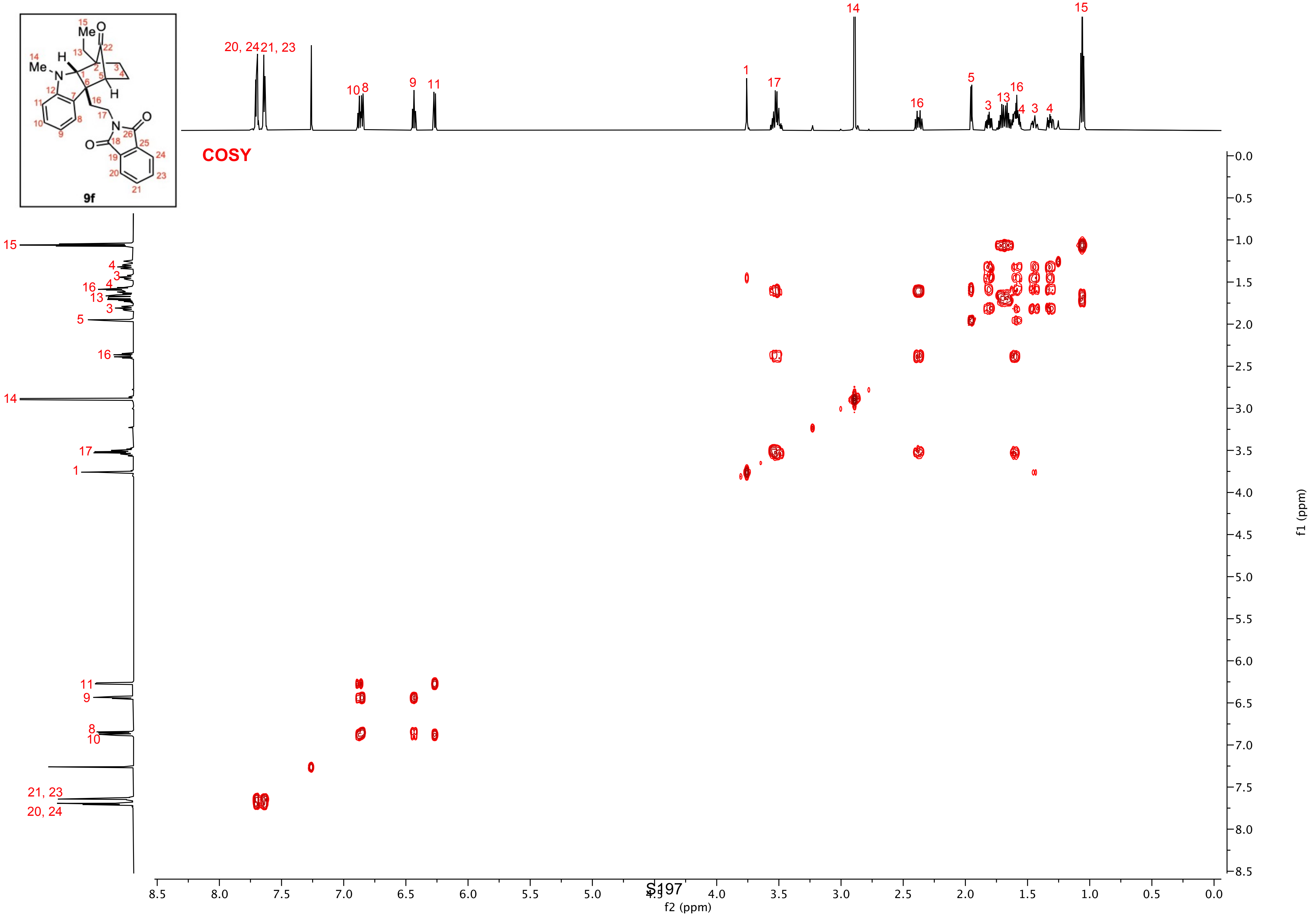

9f
COSY
20, 24
21, 23
10
8
9
11
1
17
14
16
5
3
13
16
4
3
4
15
f2 (ppm)
f1 (ppm)

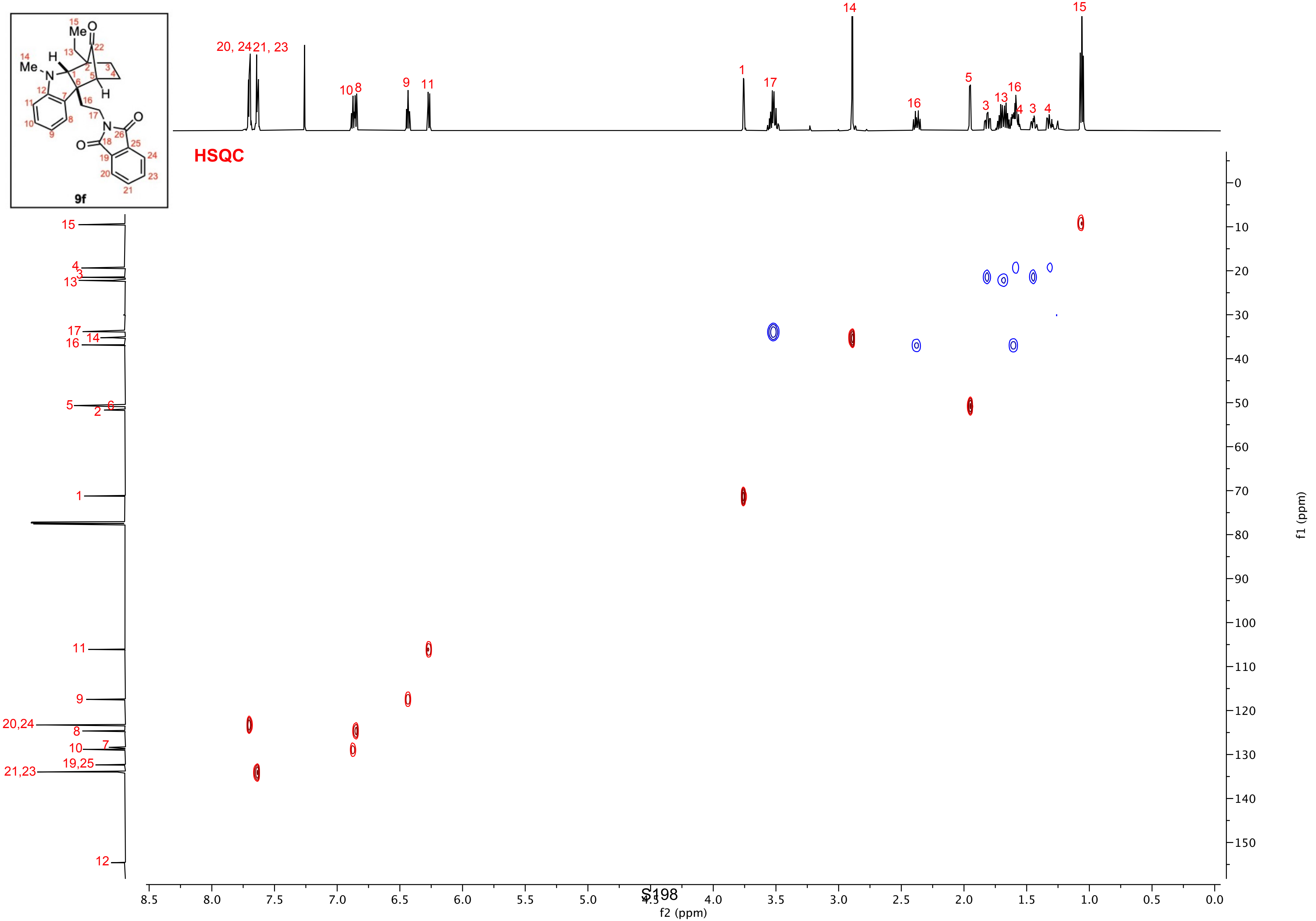
9f
HSQC
20, 24
21, 23
10
8
9
11
1
17
14
16
5
3
13
16
4
3
4
15
15
4
3
13
17
14
16
5
6
2
1
11
9
20,24
8
7
10
19,25
21,23
12
f2 (ppm)
f1 (ppm)

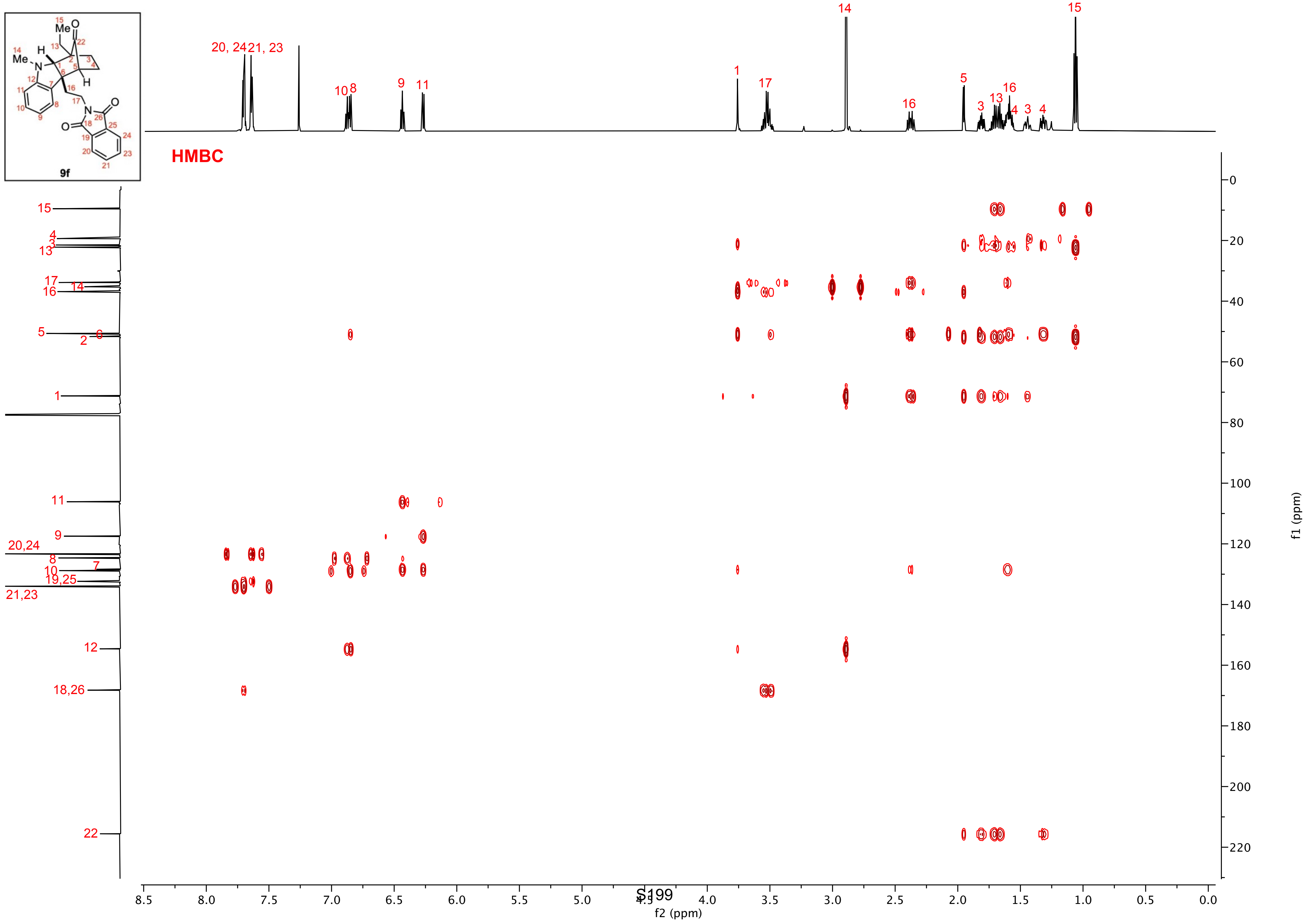
HMBC
9f
f2 (ppm)
f1 (ppm)

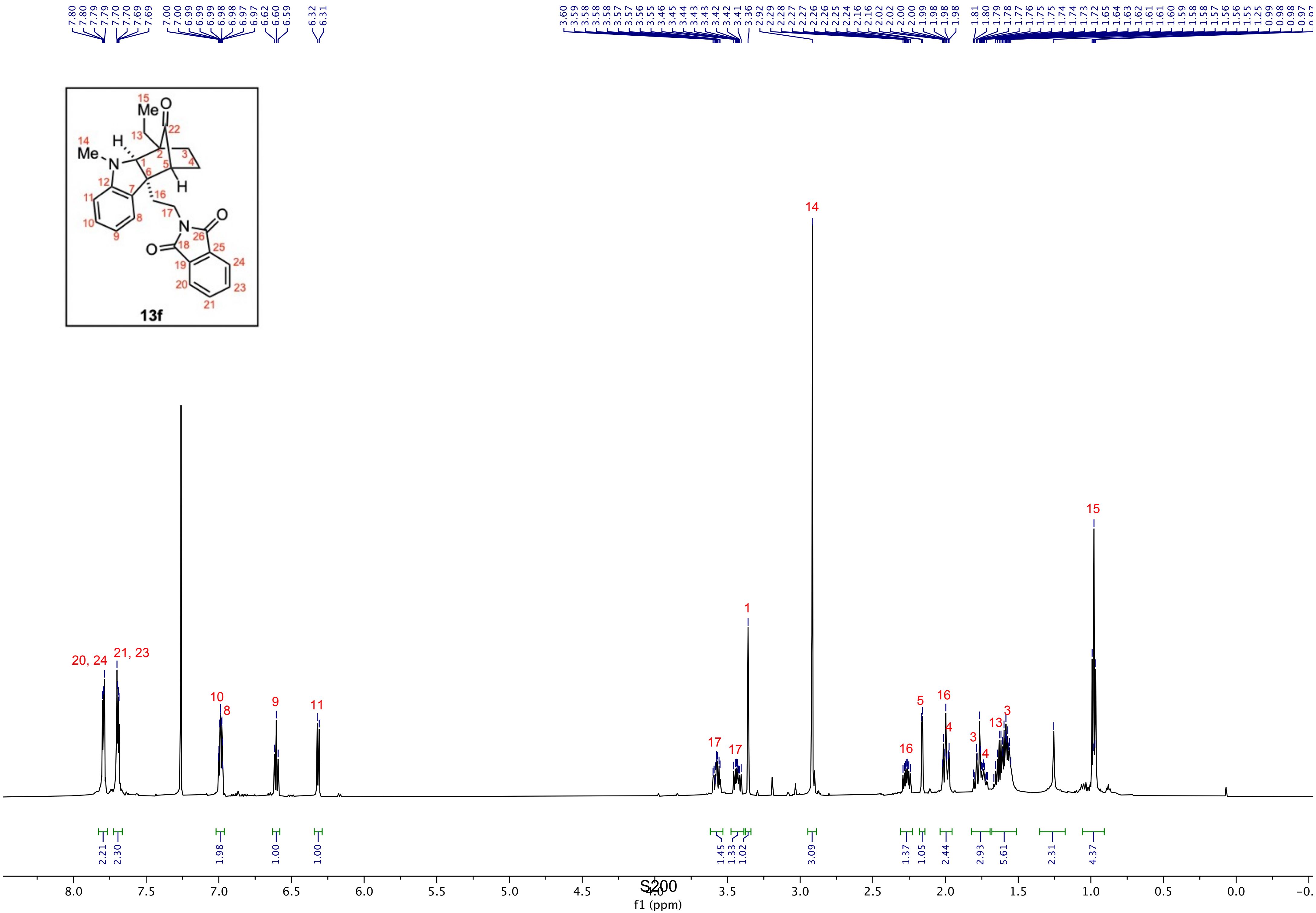
13f
20, 24
21, 23
10
8
9
11
17
17
1
14
16
5
16
4
3
4
13
3
15
f1 (ppm)

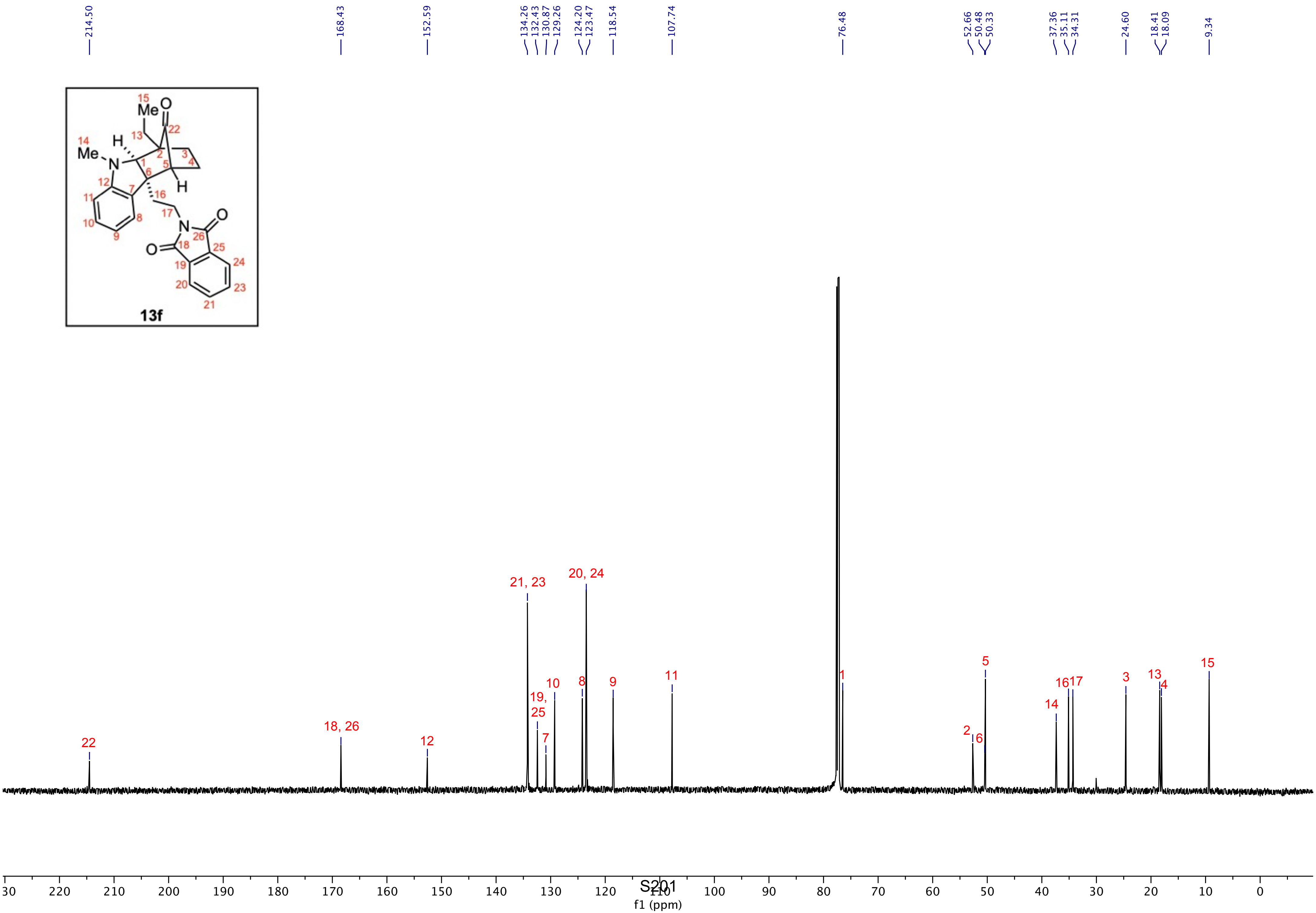
214.50
168.43
152.59
134.26
132.43
130.87
129.26
124.20
123.47
118.54
107.74
76.48
52.66
50.48
50.33
37.36
35.11
34.31
24.60
18.41
18.09
9.34
13f
22
18, 26
12
21, 23
19, 25
7
10
8
20, 24
9
11
1
2
16
5
14
16
17
3
13
4
15
f1 (ppm)

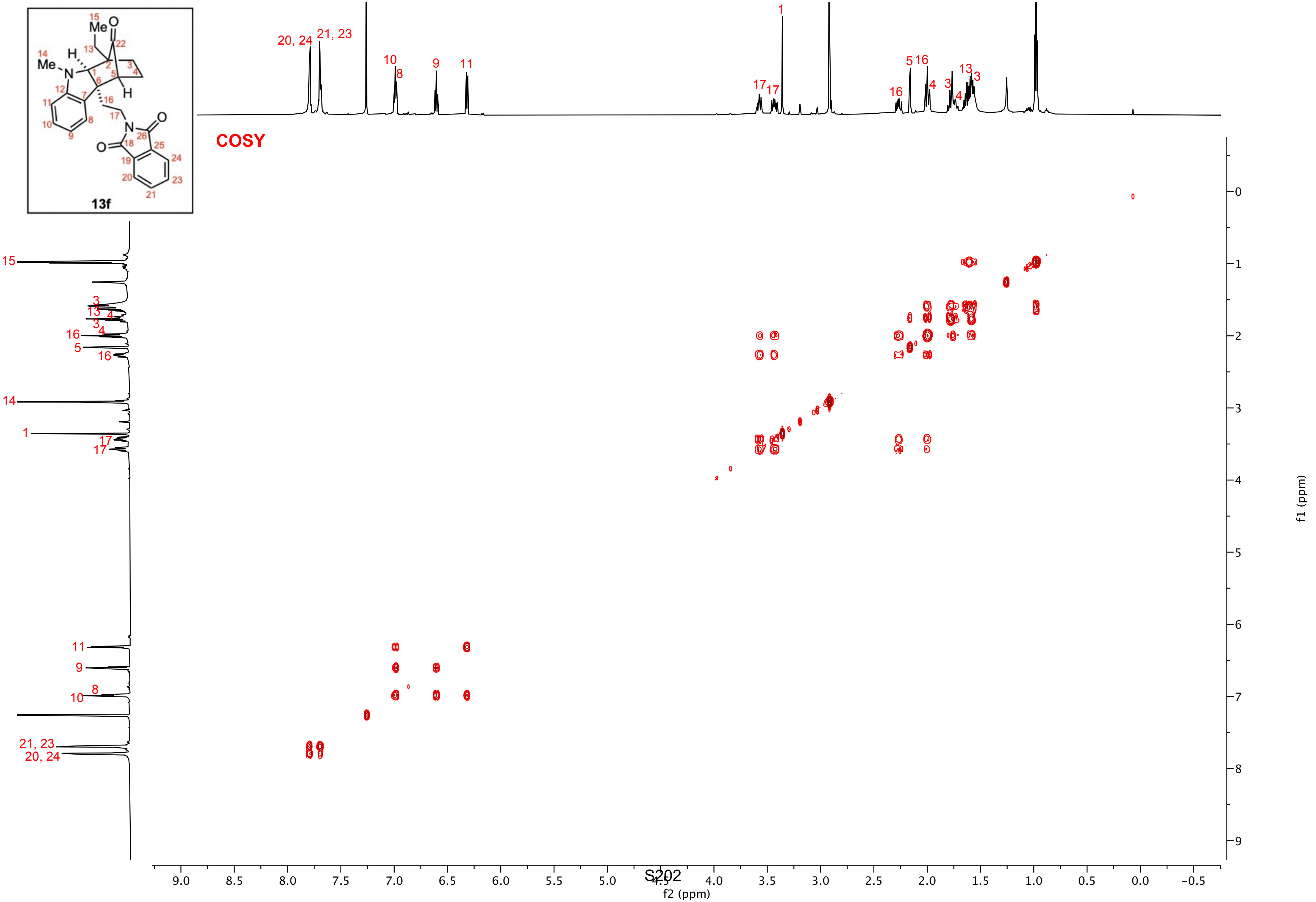
13f
COSY
f2 (ppm)
f1 (ppm)

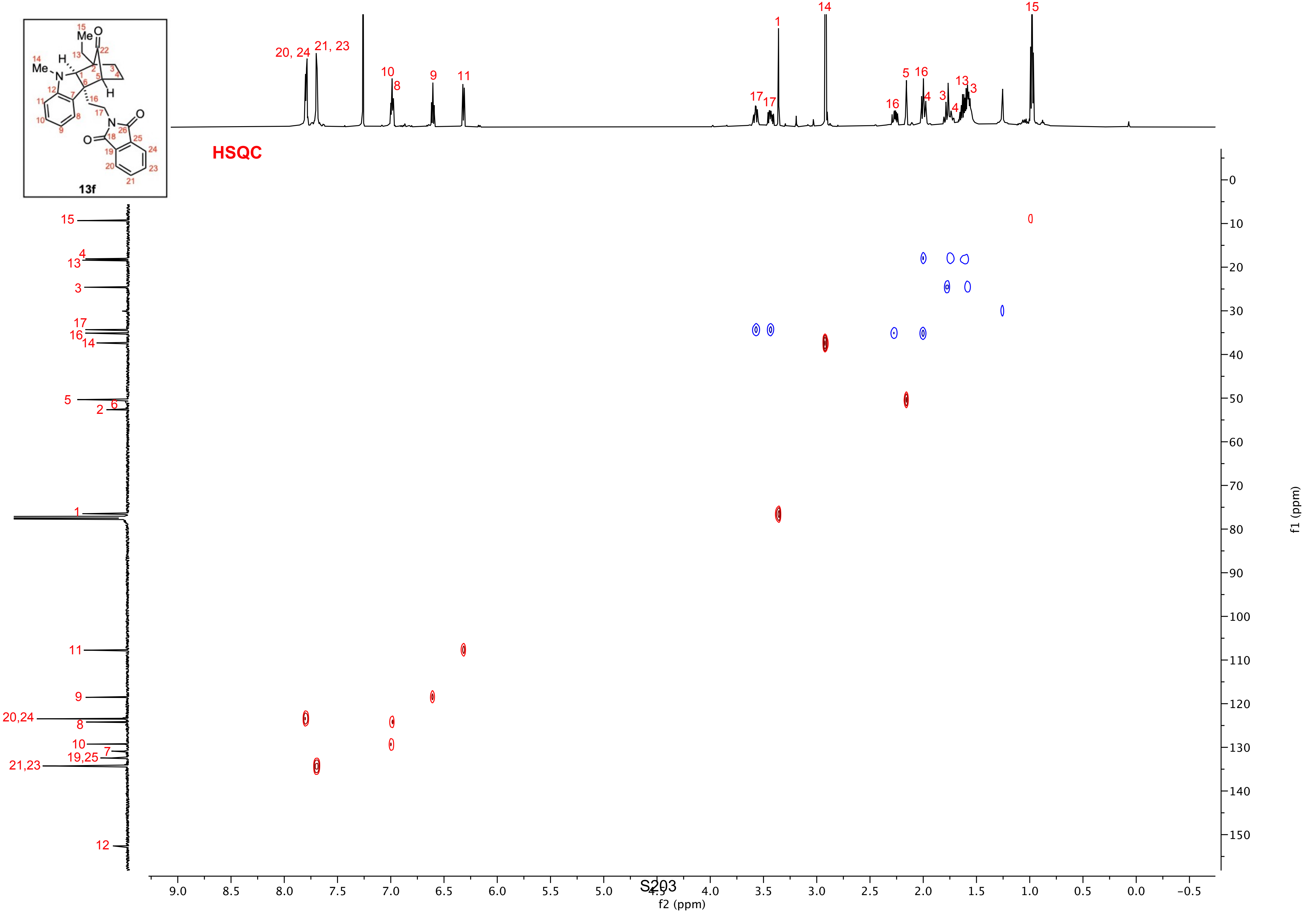
HSQC
13f
f2 (ppm)
f1 (ppm)

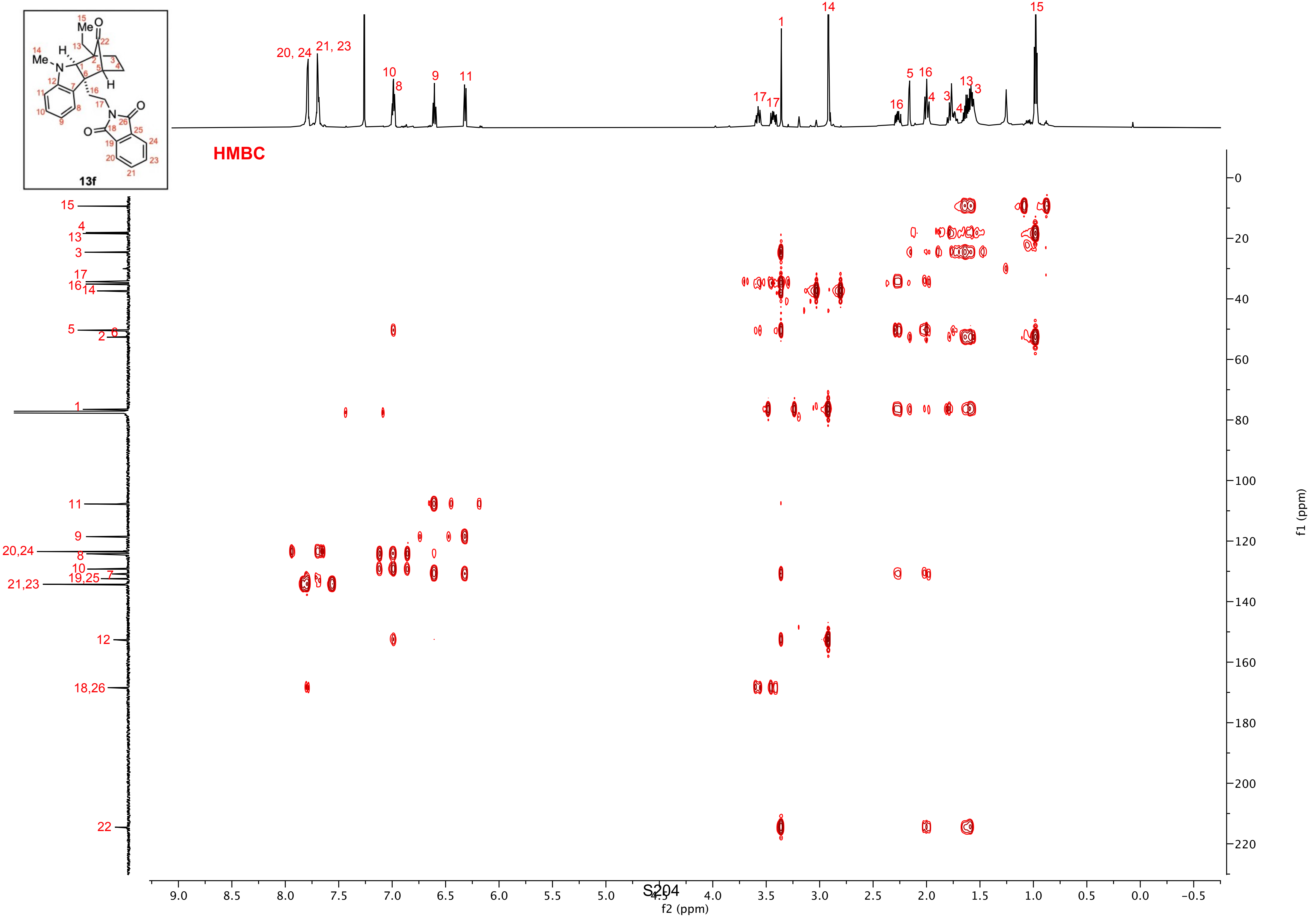
HMBC
13f
f2 (ppm)
f1 (ppm)

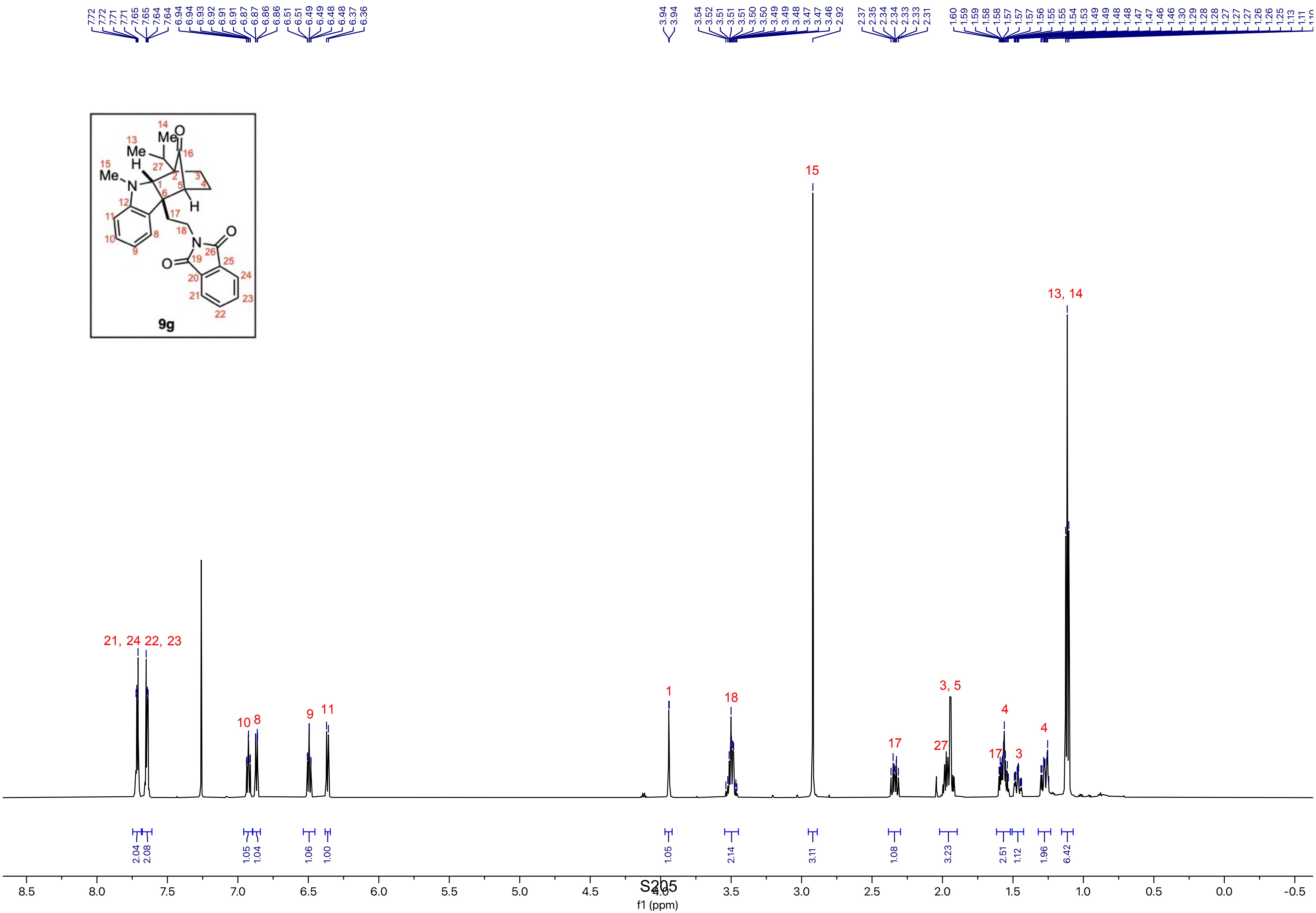
9g
15
21, 24 22, 23
10 8
9 11
1
18
17
27
3, 5
4
17 3
4
13, 14
f1 (ppm)

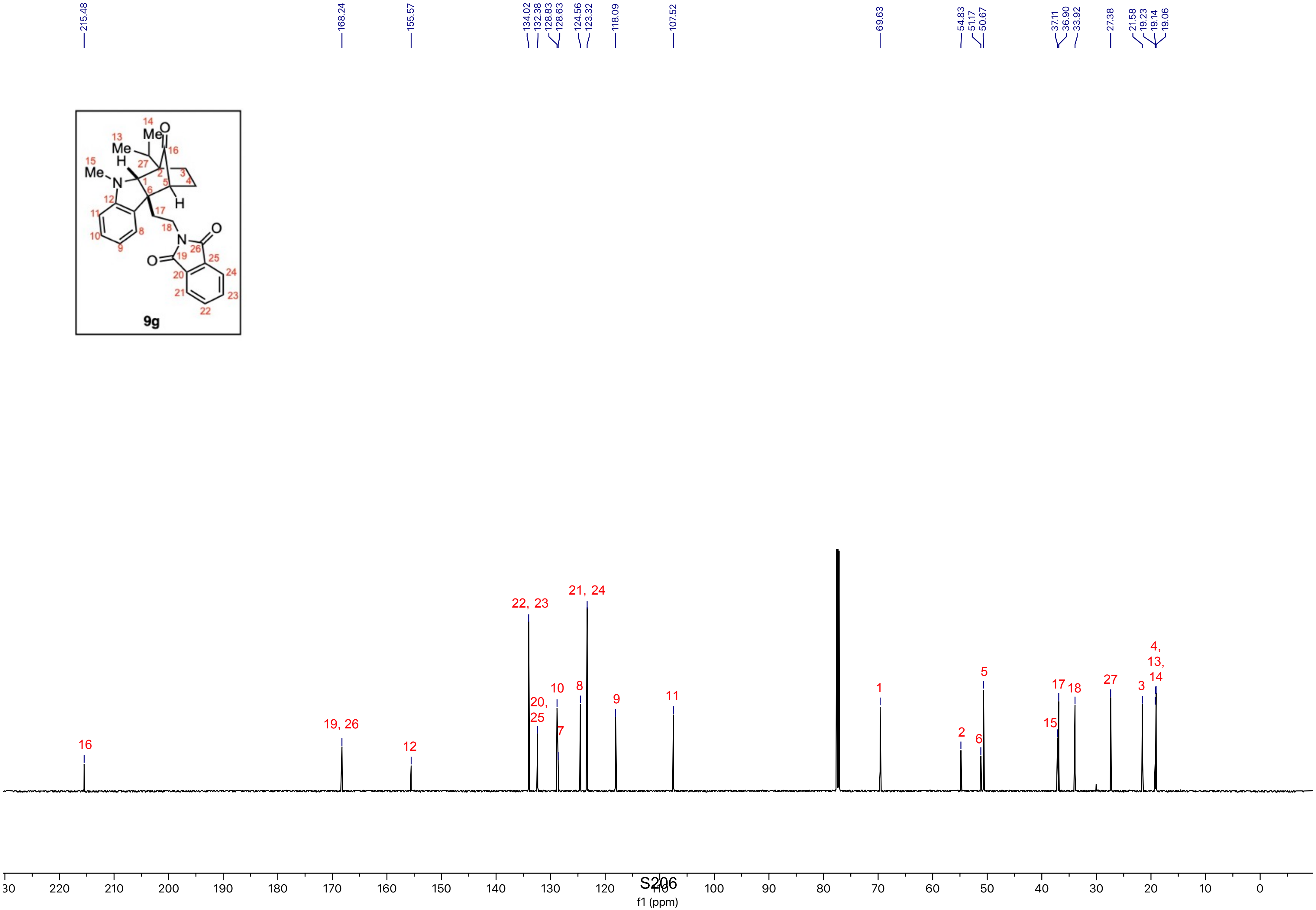
215.48
168.24
155.57
134.02
132.38
128.83
128.63
124.56
123.32
118.09
107.52
69.63
54.83
51.17
50.67
37.11
36.90
33.92
27.38
21.58
19.23
19.14
19.06
9g
16
19, 26
12
22, 23
20, 25
10
7
8
21, 24
9
11
1
2
6
5
15
17
18
27
3
4, 13, 14
f1 (ppm)

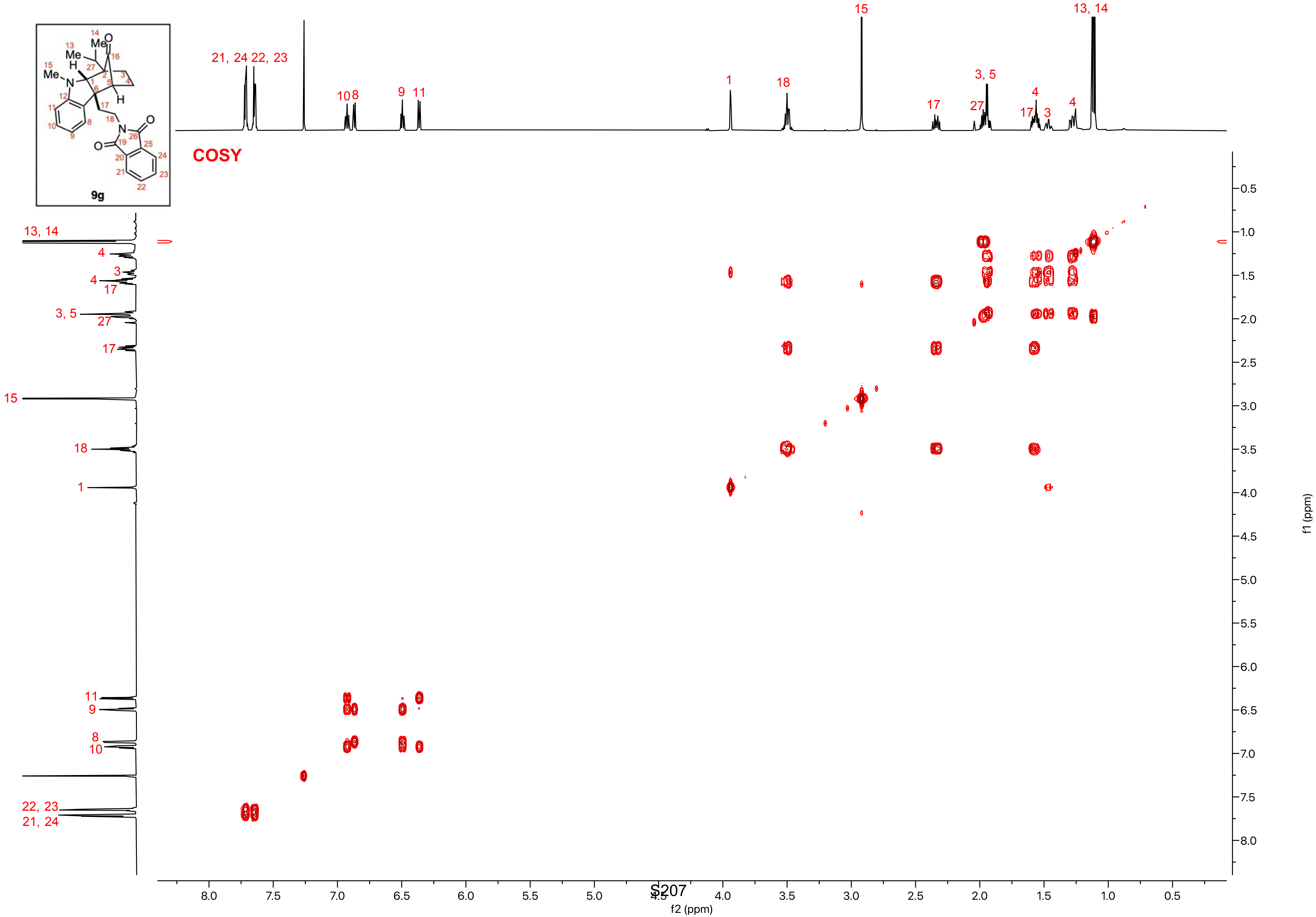
9g
COSY
21, 24 22, 23
10 8
9 11
1
18
15
17
27
3, 5
4
17 3
4
13, 14
f2 (ppm)
f1 (ppm)

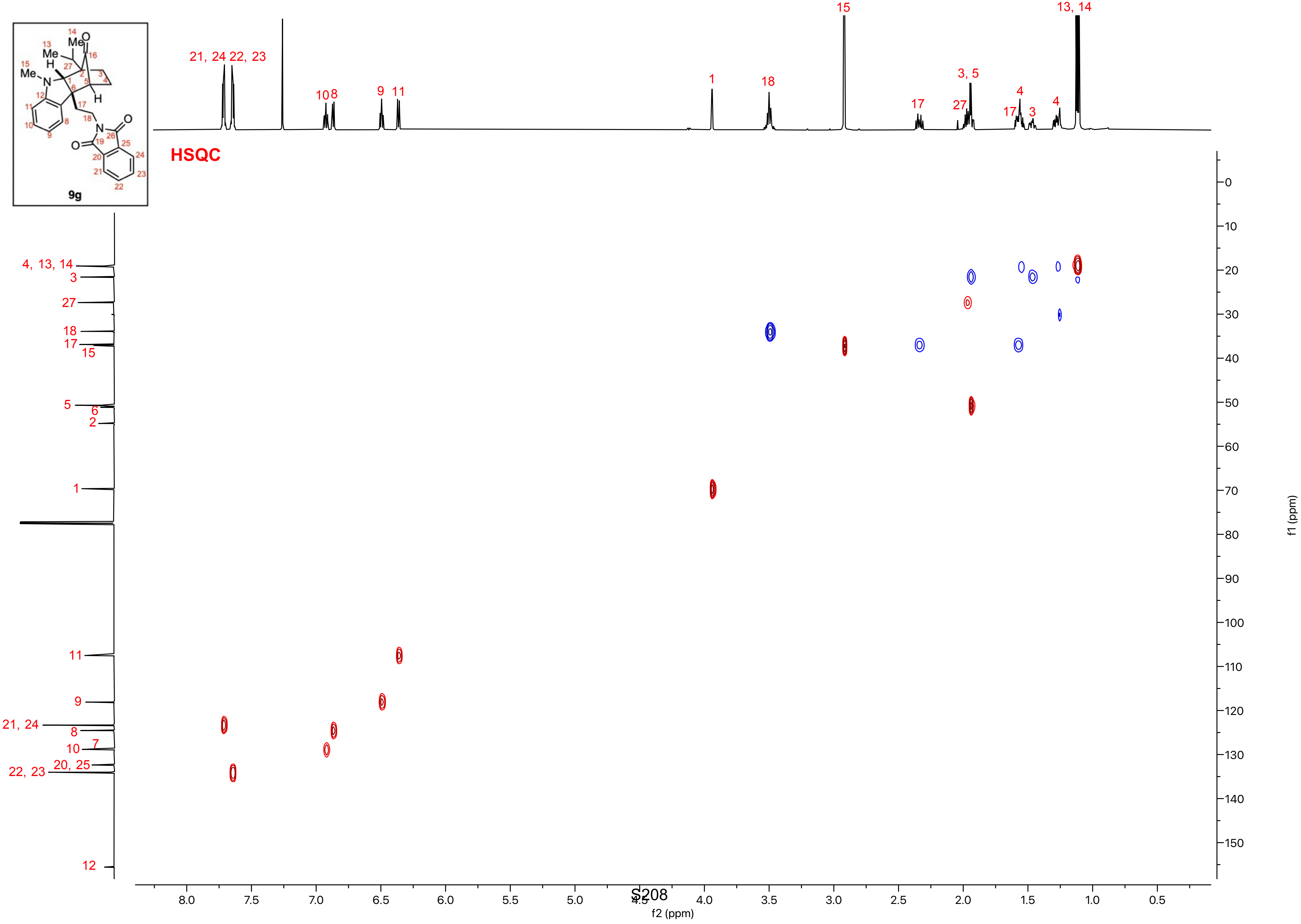
9g
HSQC
21, 24 22, 23
10 8
9 11
1
18
15
17
27
3, 5
17 4 3
4
13, 14
4, 13, 14
3
27
18
17
15
5
6
2
1
11
9
21, 24
8
10 7
20, 25
22, 23
12
f2 (ppm)
f1 (ppm)

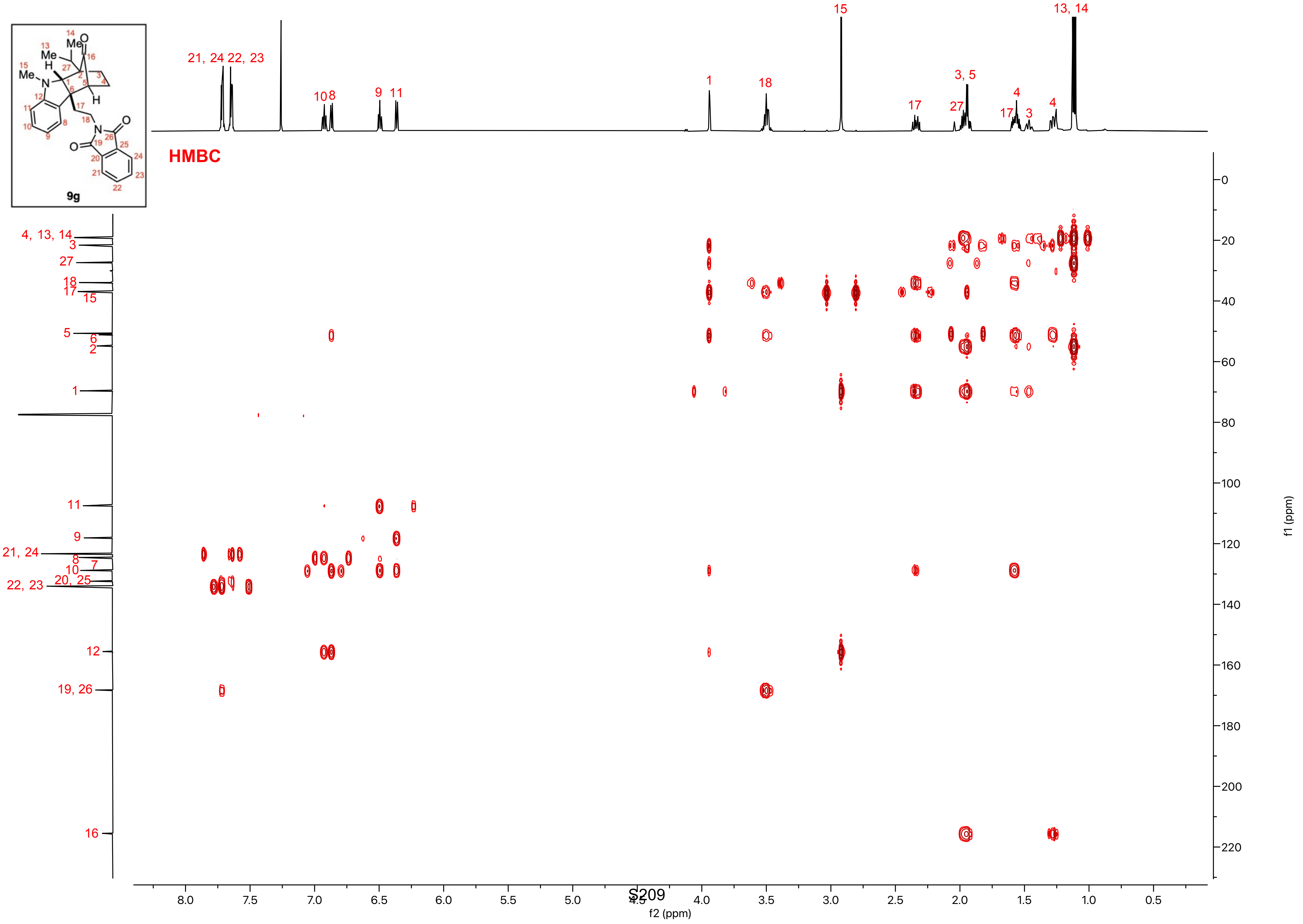
9g
HMBC
21, 24 22, 23
10 8
9 11
1
18
15
17
27
3, 5
17 3
4
13, 14
4, 13, 14
3
27
18
17
15
5
6
2
1
11
9
21, 24
8
7
10
20, 25
22, 23
12
19, 26
16
f2 (ppm)
f1 (ppm)

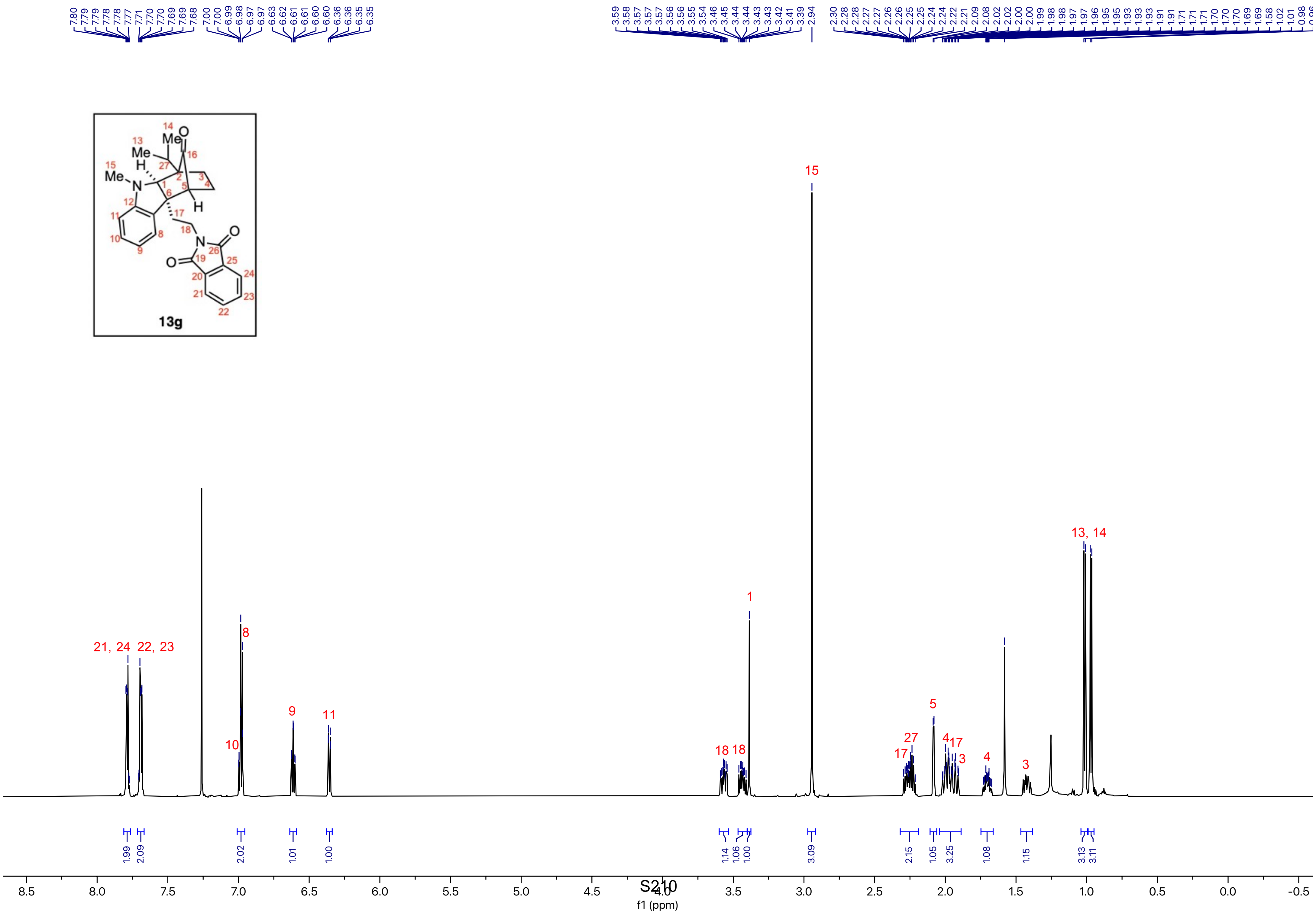
13g
15
1
13, 14
21, 24
22, 23
8
10
9
11
18
18
17
27
5
4
17
3
4
3
f1 (ppm)

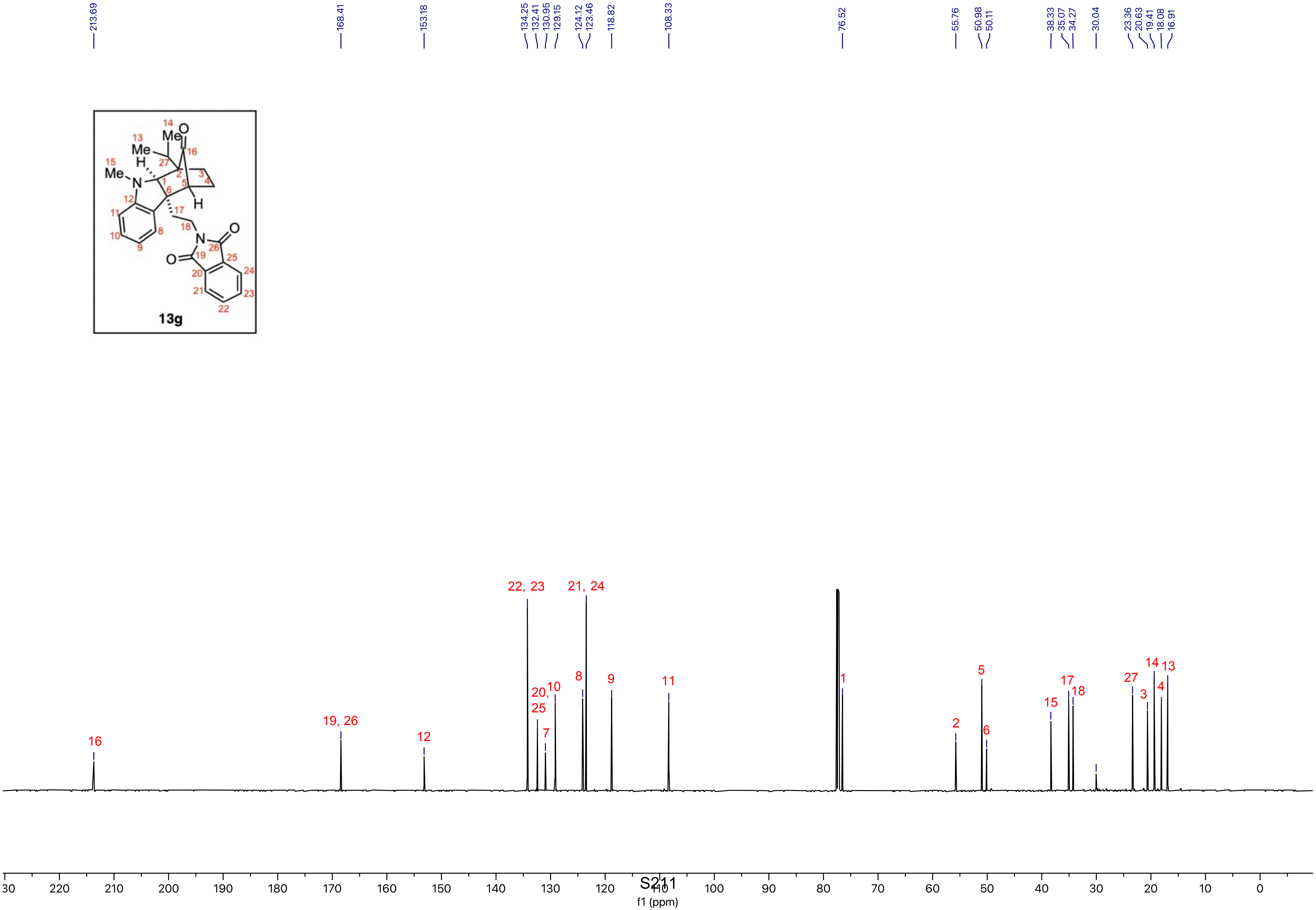
213.69
168.41
153.18
134.25
132.41
130.95
129.15
124.12
123.46
118.82
108.33
76.52
55.76
50.98
50.11
38.33
35.07
34.27
30.04
23.36
20.63
19.41
18.08
16.91
13g
16
19, 26
12
22, 23
20, 25
7
10
8
21, 24
9
11
1
2
5
6
15
17
18
27
3
14
4
13
f1 (ppm)

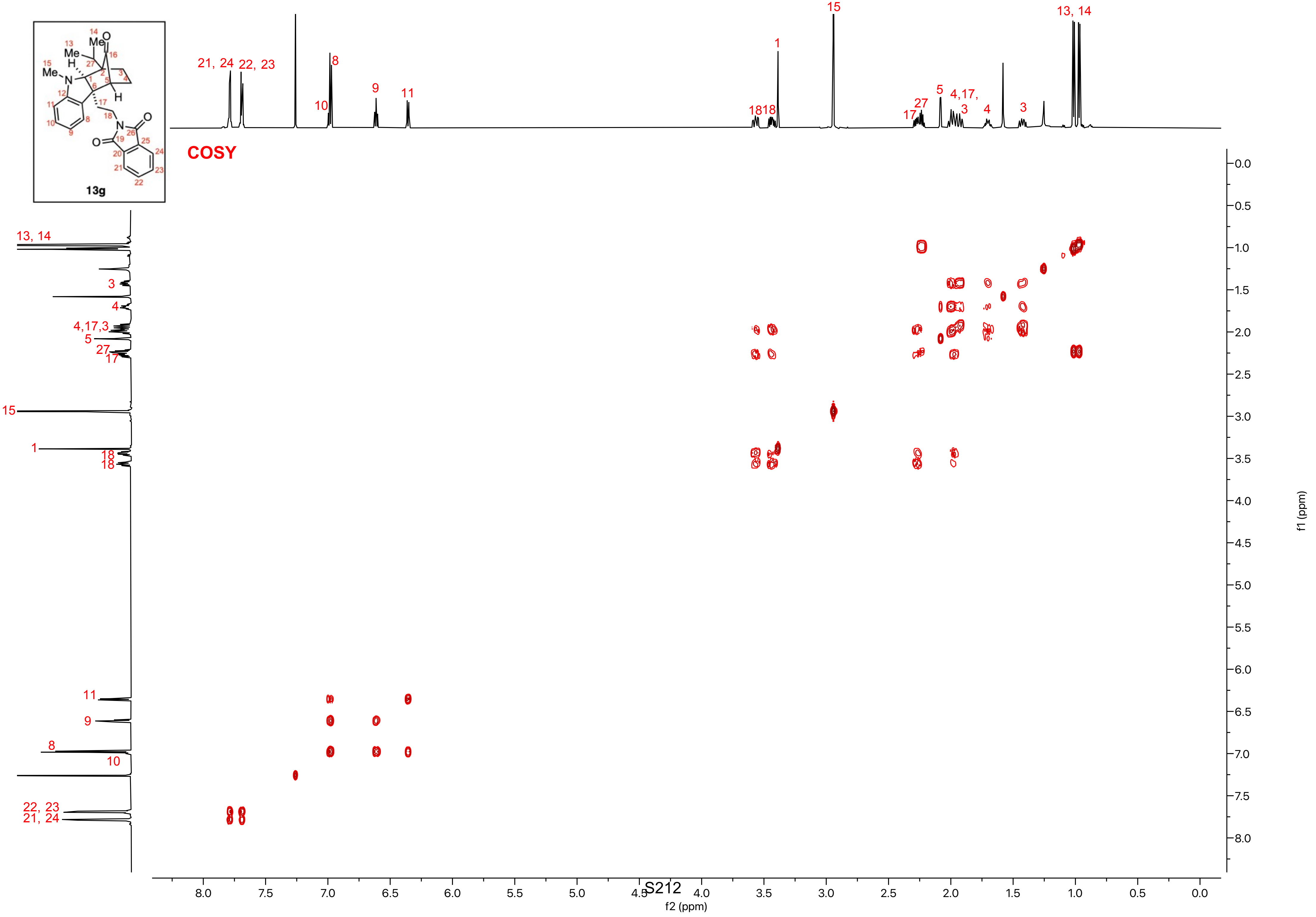

13g
COSY
21, 24
22, 23
8
10
9
11
18
18
1
15
17
27
5
4,17,
3
4
3
13, 14
f2 (ppm)
f1 (ppm)

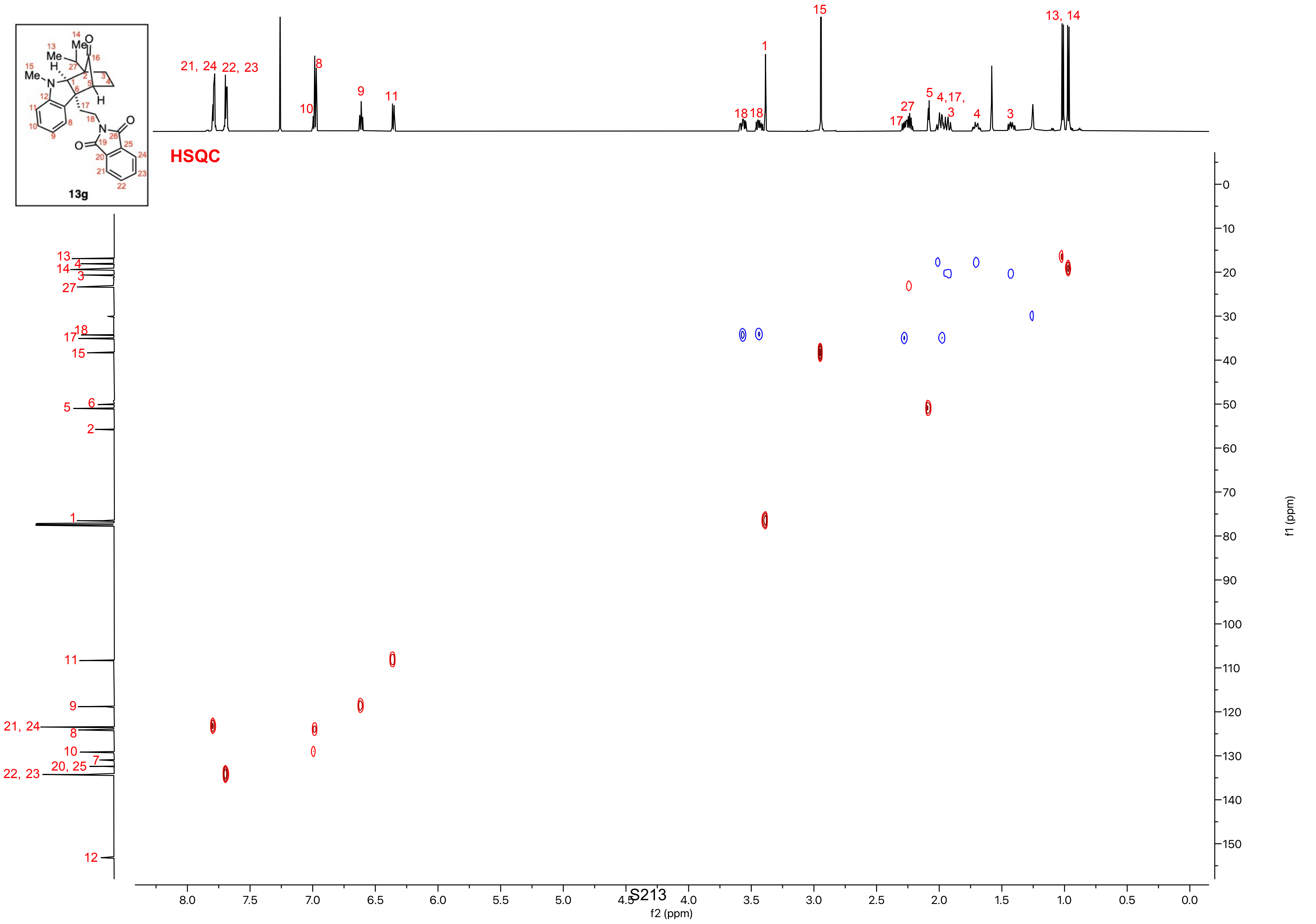
13g
HSQC
15
13, 14
1
21, 24
22, 23
8
10
9
11
18
18
27
17
5
4,17,
3
4
3
f2 (ppm)
f1 (ppm)

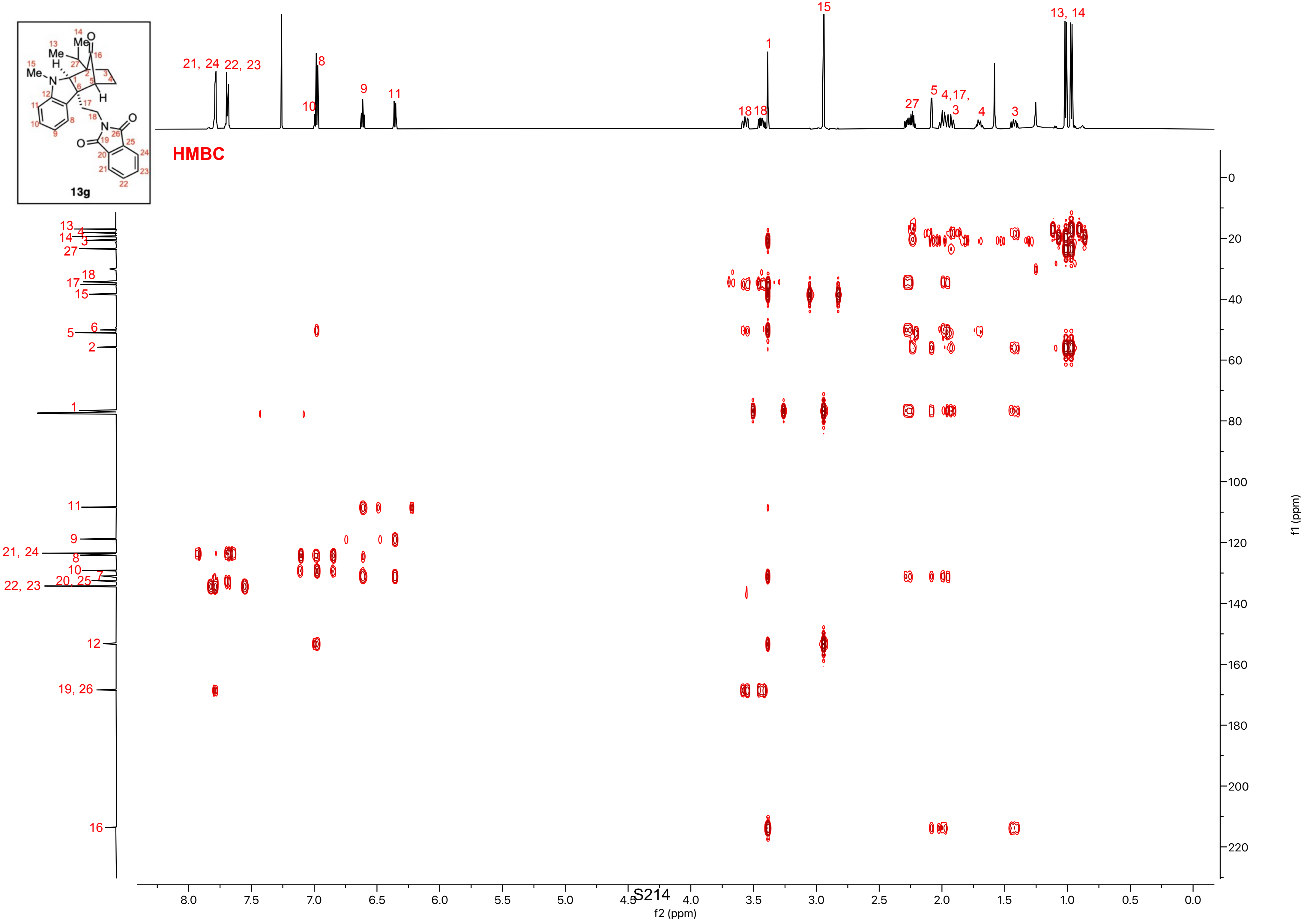
13g
HMBC
f2 (ppm)
f1 (ppm)

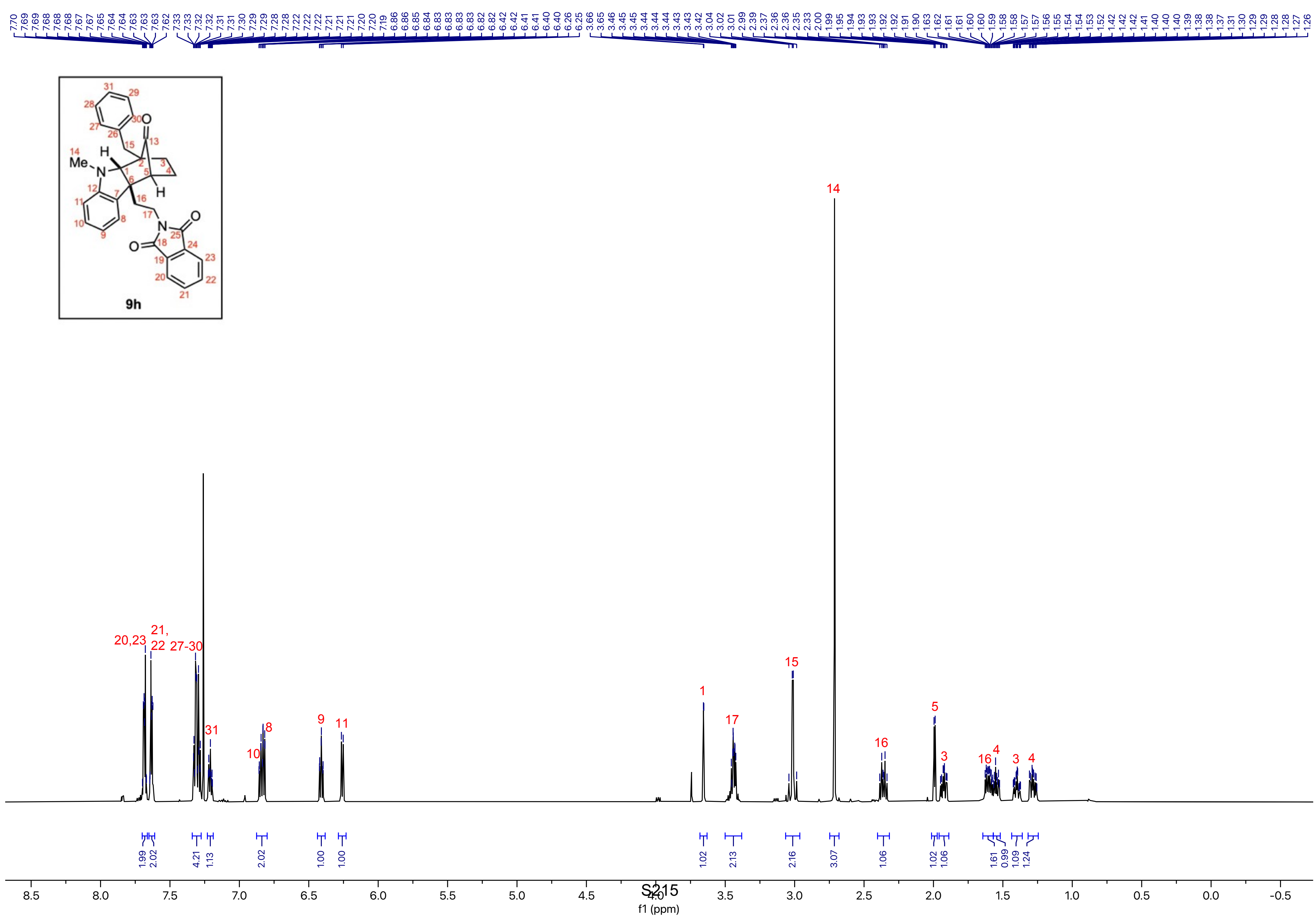
7.70 7.69 7.69 7.68 7.68 7.67 7.65 7.64 7.64 7.63 7.63 7.63 7.62 7.33 7.32 7.32 7.32 7.31 7.31 7.30 7.29 7.29 7.28 7.28 7.22 7.22 7.22 7.21 7.21 7.21 7.20 7.20 7.19 6.86 6.85 6.84 6.83 6.83 6.83 6.83 6.82 6.82 6.42 6.42 6.41 6.40 6.40 6.26 6.25 3.66 3.65 3.46 3.45 3.45 3.44 3.44 3.44 3.43 3.43 3.42 3.04 3.02 3.01 2.99 2.39 2.37 2.36 2.36 2.35 2.33 2.00 1.99 1.95 1.94 1.93 1.93 1.92 1.92 1.91 1.90 1.63 1.62 1.61 1.61 1.60 1.60 1.59 1.59 1.58 1.58 1.57 1.57 1.56 1.55 1.54 1.54 1.53 1.52 1.42 1.42 1.41 1.40 1.40 1.40 1.39 1.38 1.38 1.37 1.31 1.30 1.29 1.29 1.28 1.28 1.27 1.26
9h
14
20,23
21, 22
27-30
31
10
8
9
11
1
17
15
16
5
3
16 4
3
4
1.99 2.02 4.21 1.13 2.02 1.00 1.00 1.02 2.13 2.16 3.07 1.06 1.02 1.06 1.61 0.99 1.09 1.24
8.5 8.0 7.5 7.0 6.5 6.0 5.5 5.0 4.5 4.0 3.5 3.0 2.5 2.0 1.5 1.0 0.5 0.0 -0.5
f1 (ppm)

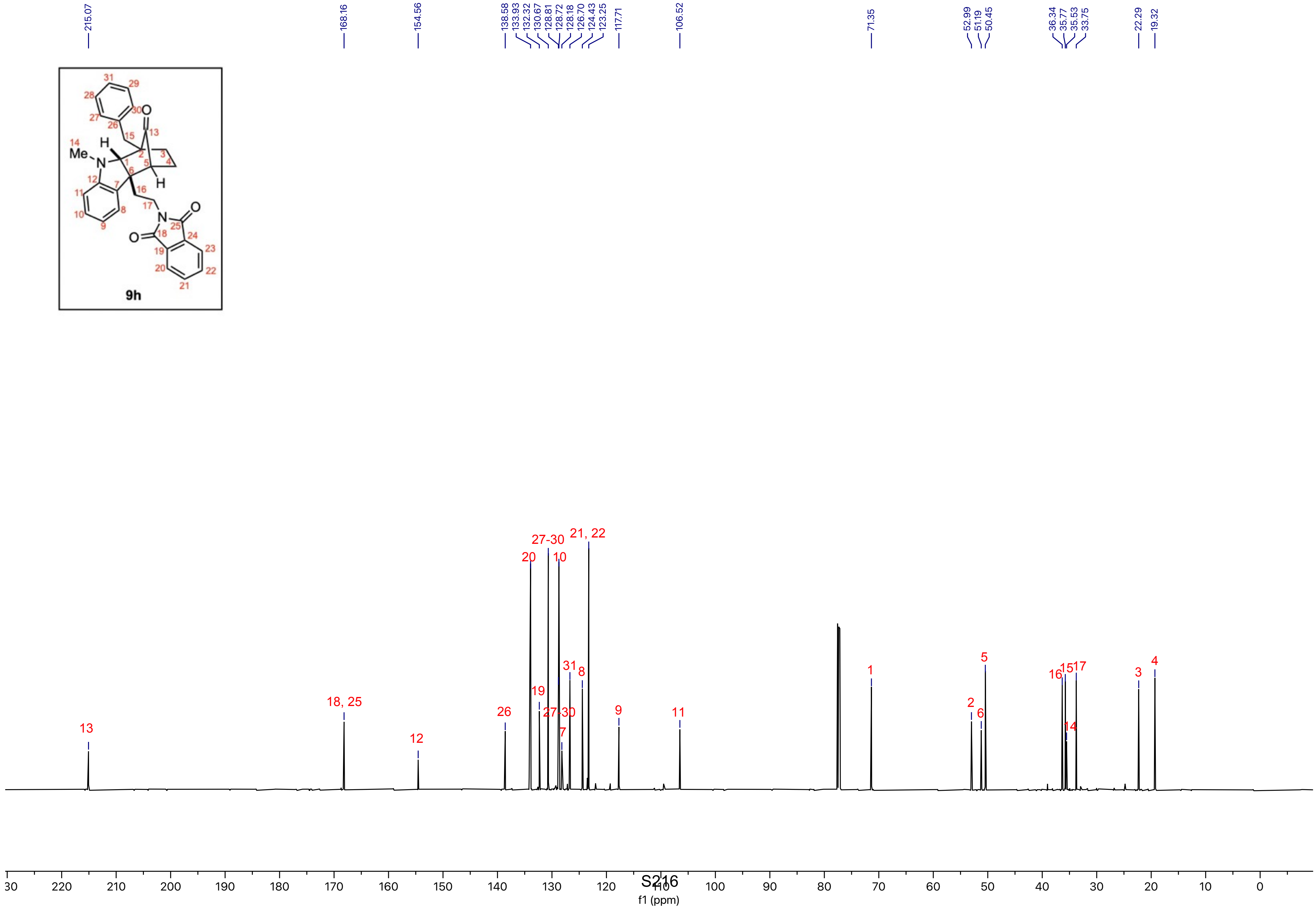

9h
215.07
168.16
154.56
138.58
133.93
132.32
130.67
128.81
128.72
128.18
126.70
124.43
123.25
117.71
106.52
71.35
52.99
51.19
50.45
36.34
35.77
35.53
33.75
22.29
19.32
13
18, 25
12
26
20
19
27-30
10
21, 22
31
8
7
9
11
1
2
6
5
16
15
17
14
3
4
f1 (ppm)

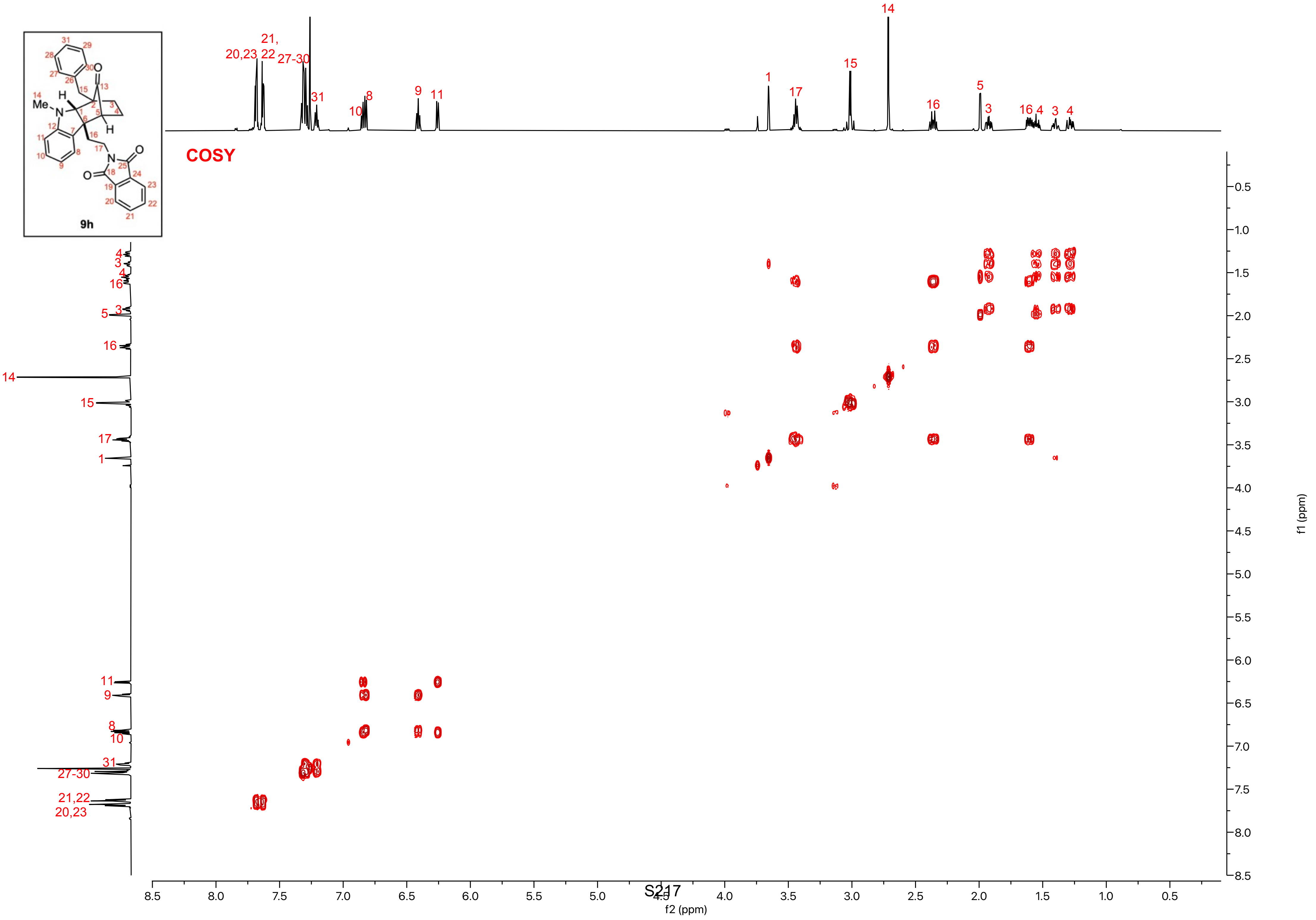
9h
COSY
f2 (ppm)
f1 (ppm)

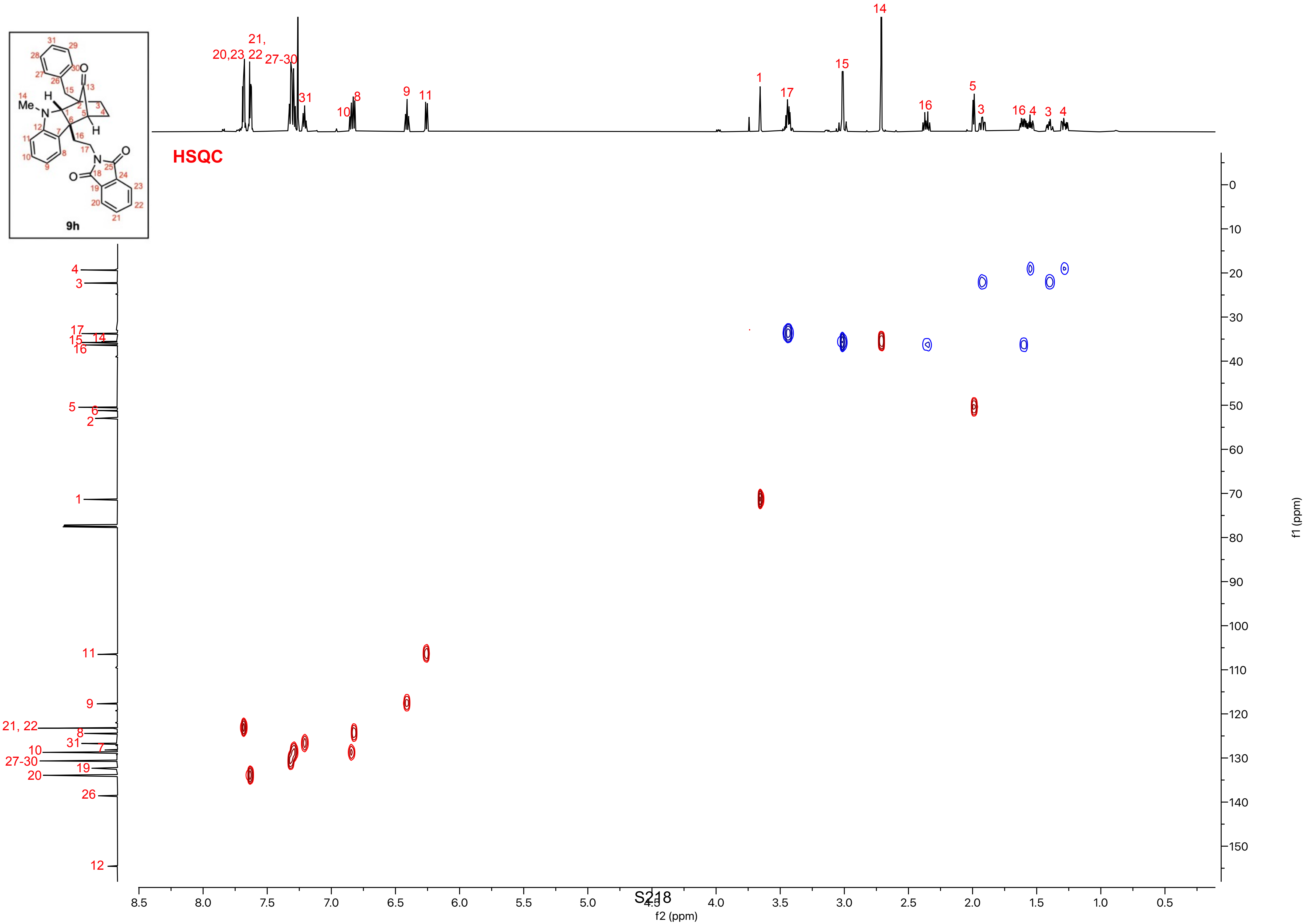
9h
HSQC
14
20,23
21, 22
27-30
31
10
8
9
11
1
17
15
16
5
3
4
12
19
20
26
7
2
6
f2 (ppm)
f1 (ppm)

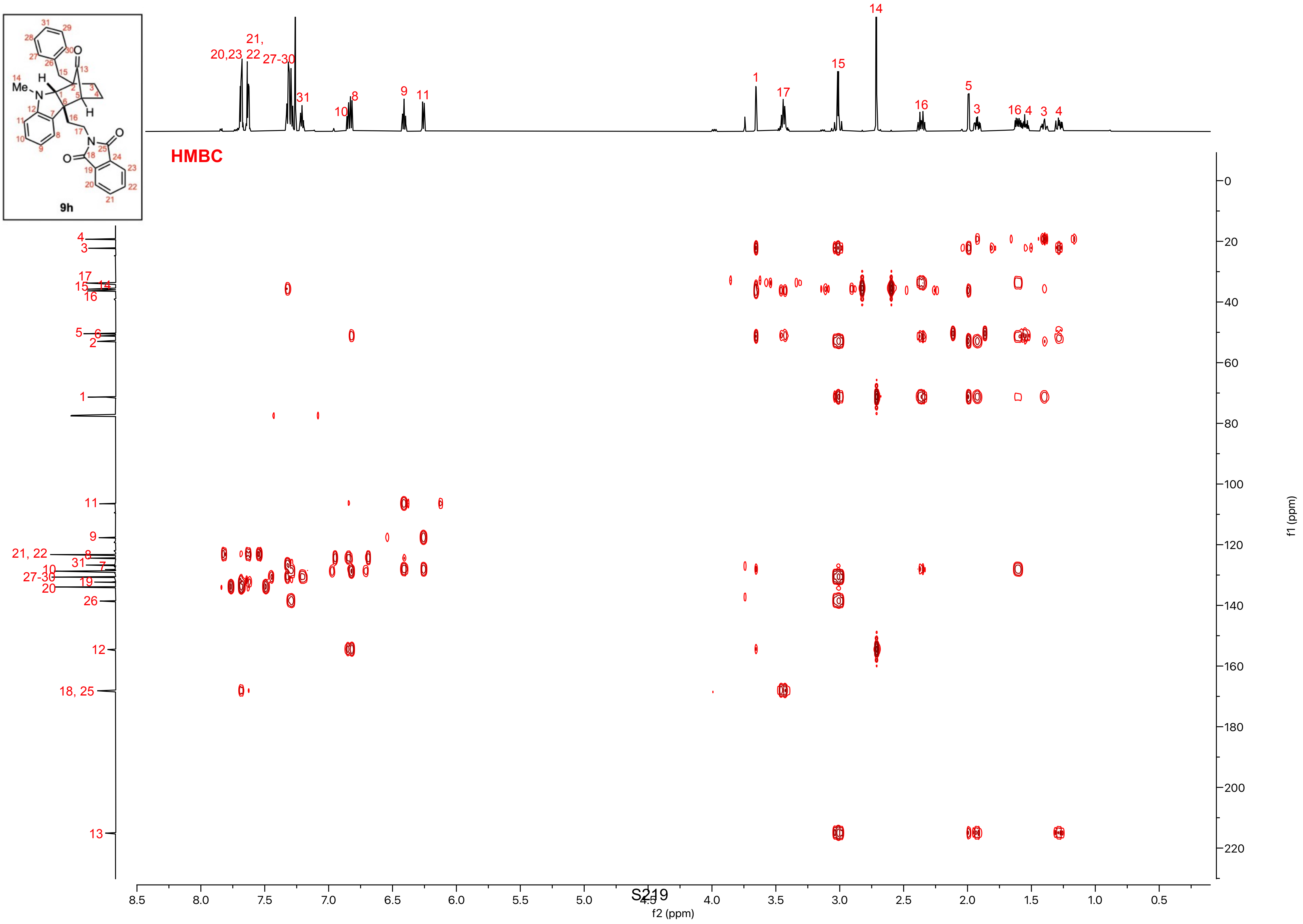
HMBC
9h
Me
N
H
O
20,23
21,
22
27-30
31
10
8
9
11
1
17
15
14
16
5
3
16
4
3
4
21, 22
31
27-30
20
19
26
12
18, 25
13
f1 (ppm)
f2 (ppm)

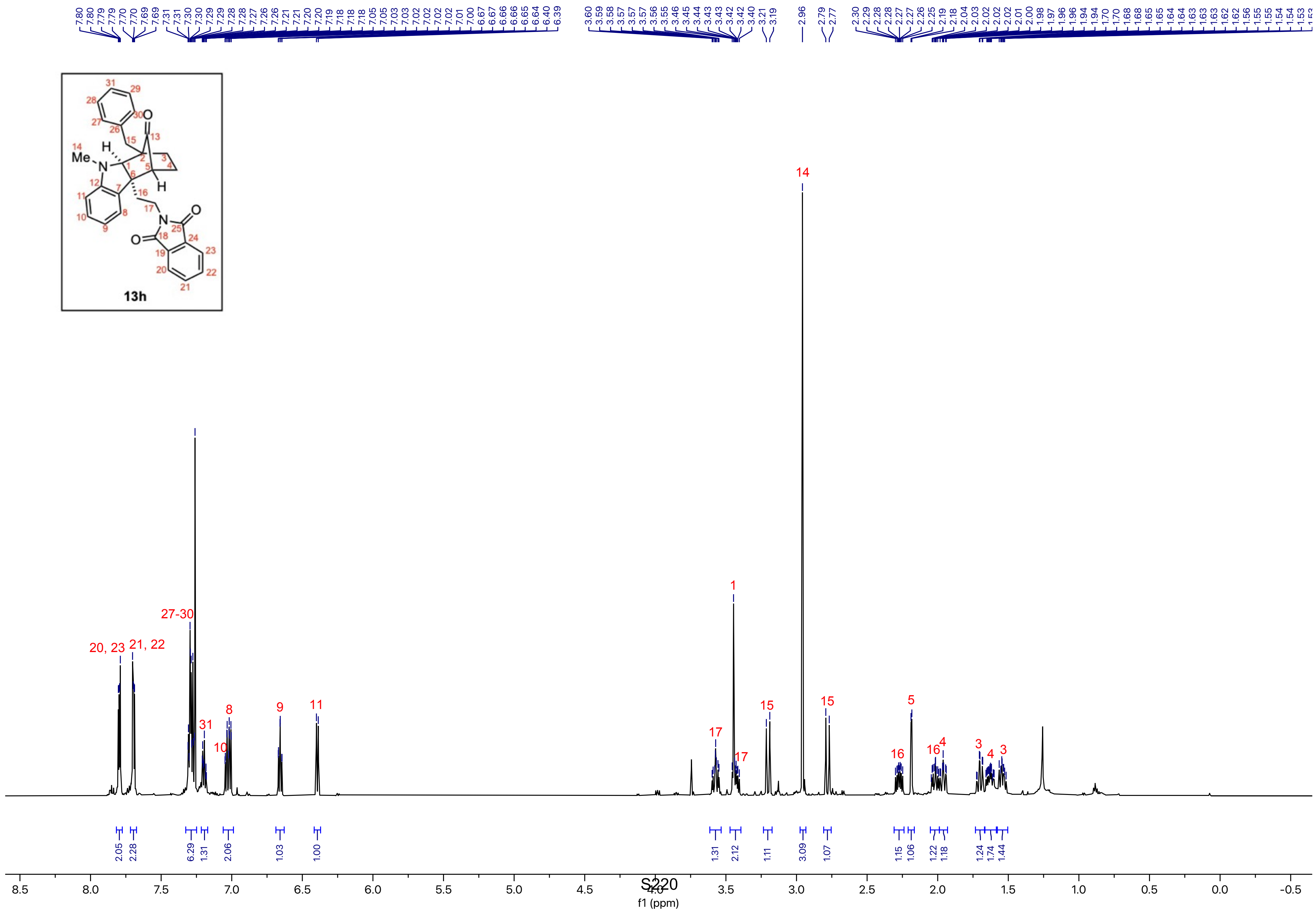

13h
Me
N
H
O
14
20, 23
21, 22
27-30
31
10
8
9
11
17
1
15
14
16
5
4
3
f1 (ppm)

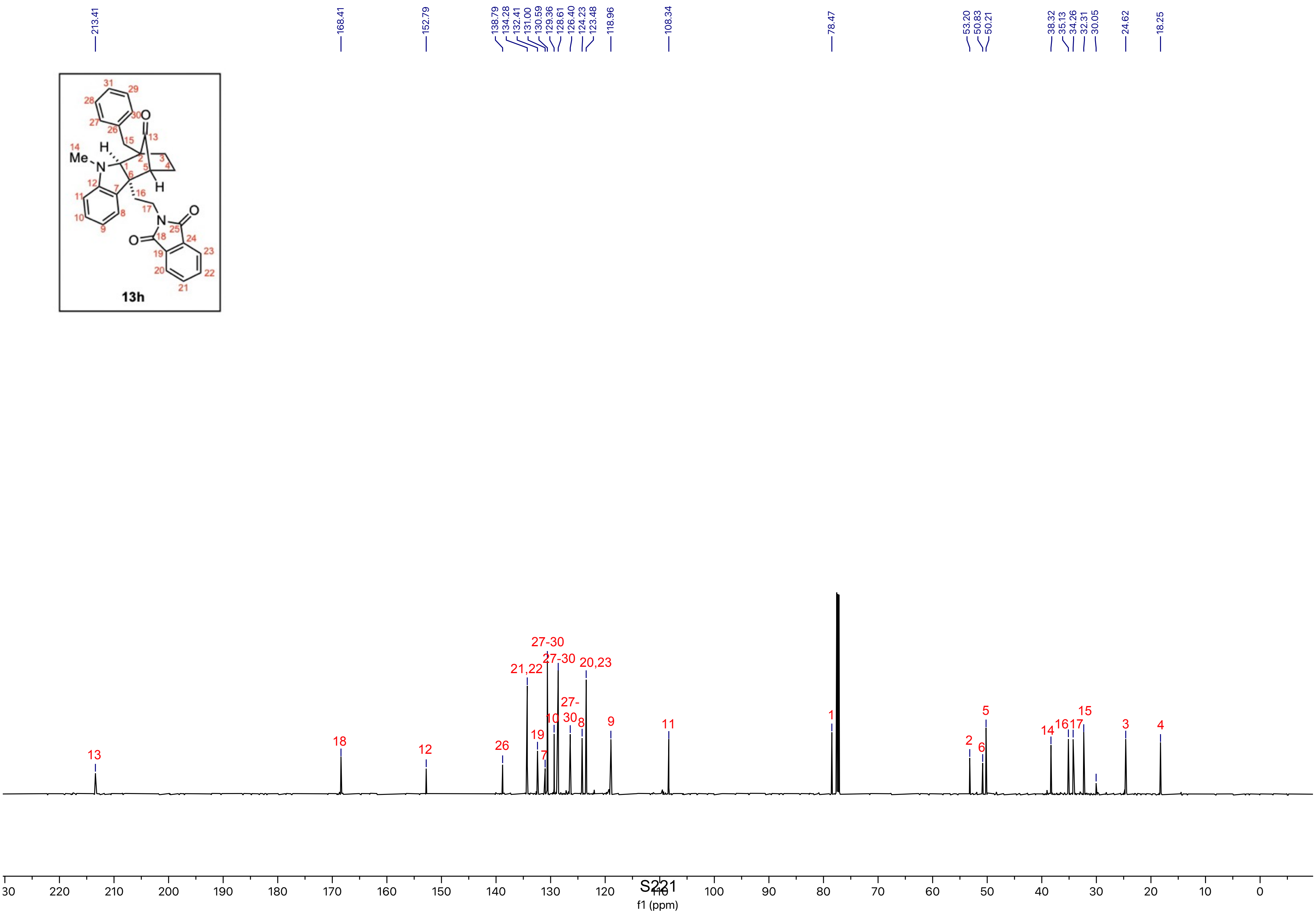
213.41
168.41
152.79
138.79
134.28
132.41
131.00
130.59
129.36
128.61
126.40
124.23
123.48
118.96
108.34
78.47
53.20
50.83
50.21
38.32
35.13
34.26
32.31
30.05
24.62
18.25
Me
N
H
O
13h
13
18
12
26
19
7
21,22
27-30
27-30
10
27-30
8
20,23
9
11
1
2
6
5
14
16
17
15
3
4
f1 (ppm)

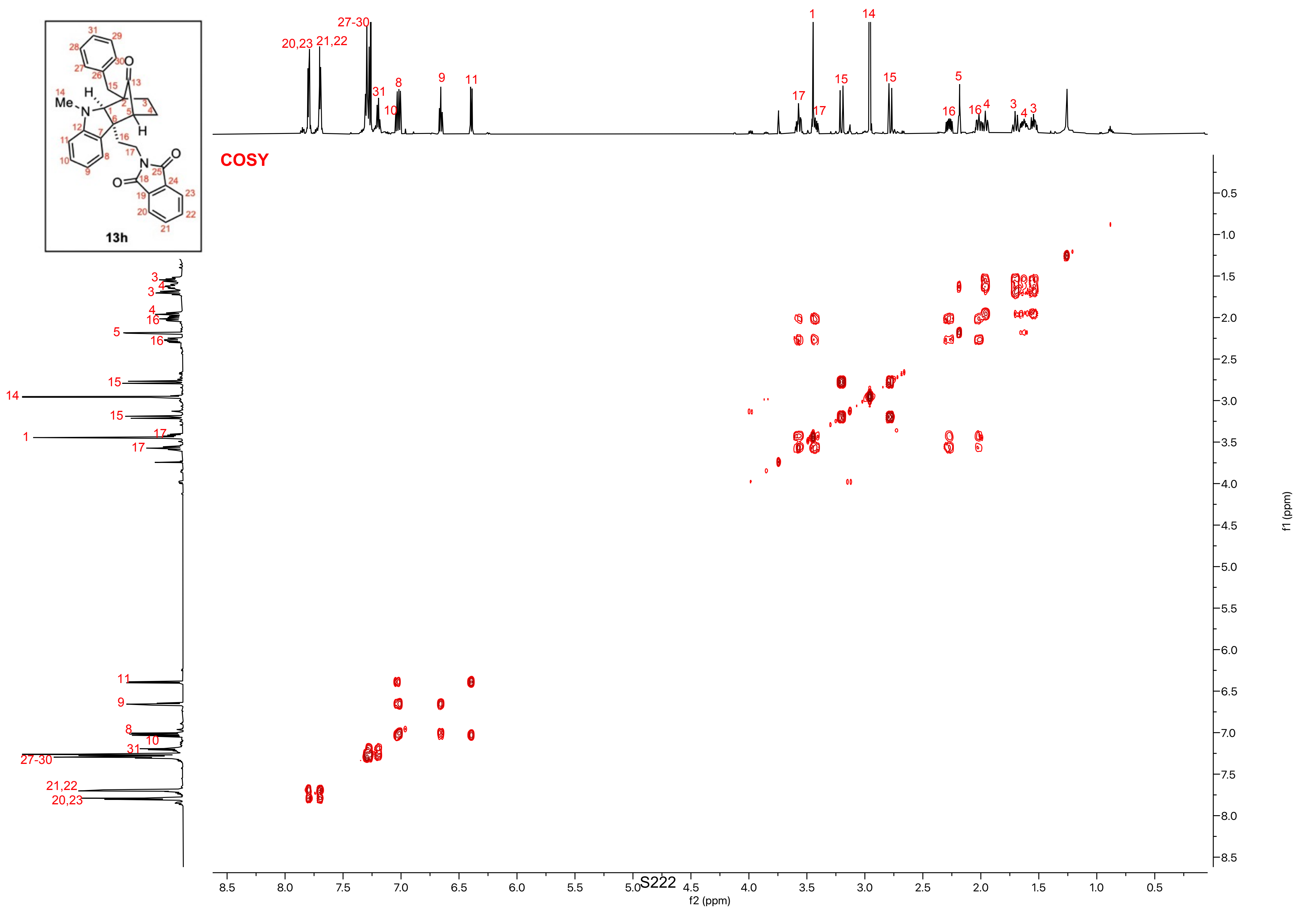
COSY
13h
f2 (ppm)
f1 (ppm)

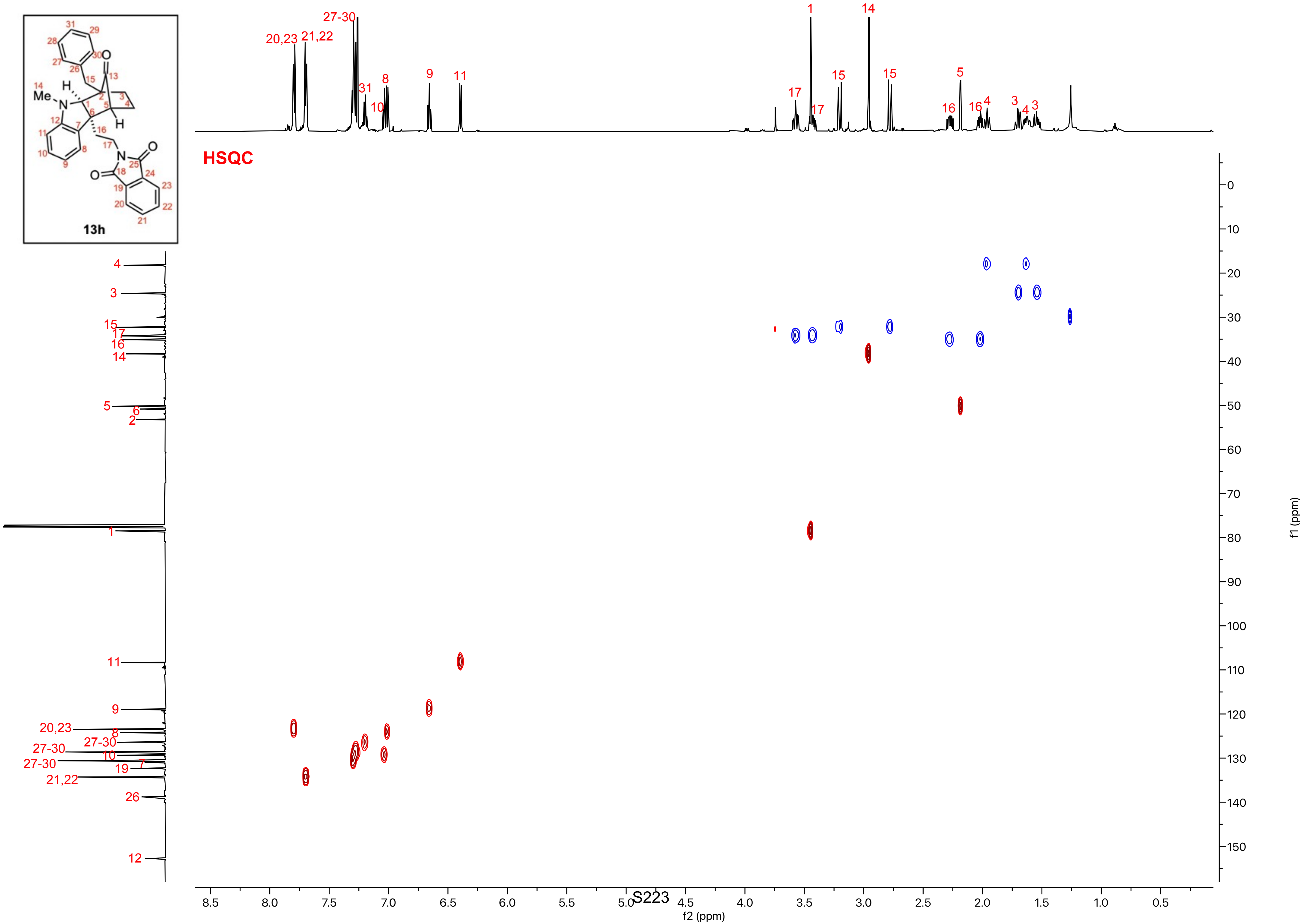
13h
HSQC
f2 (ppm)
f1 (ppm)

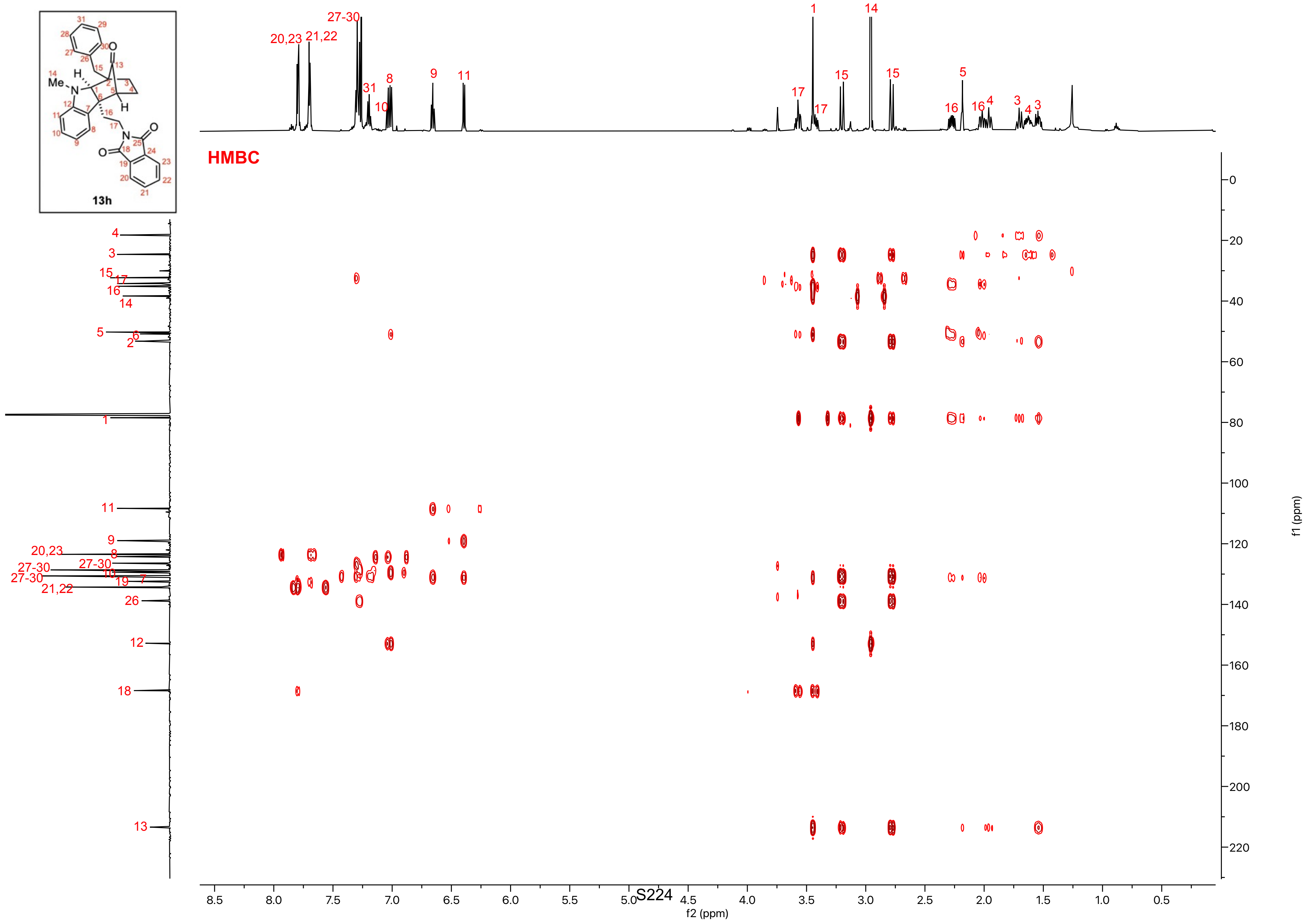
13h
HMBC
f2 (ppm)
f1 (ppm)

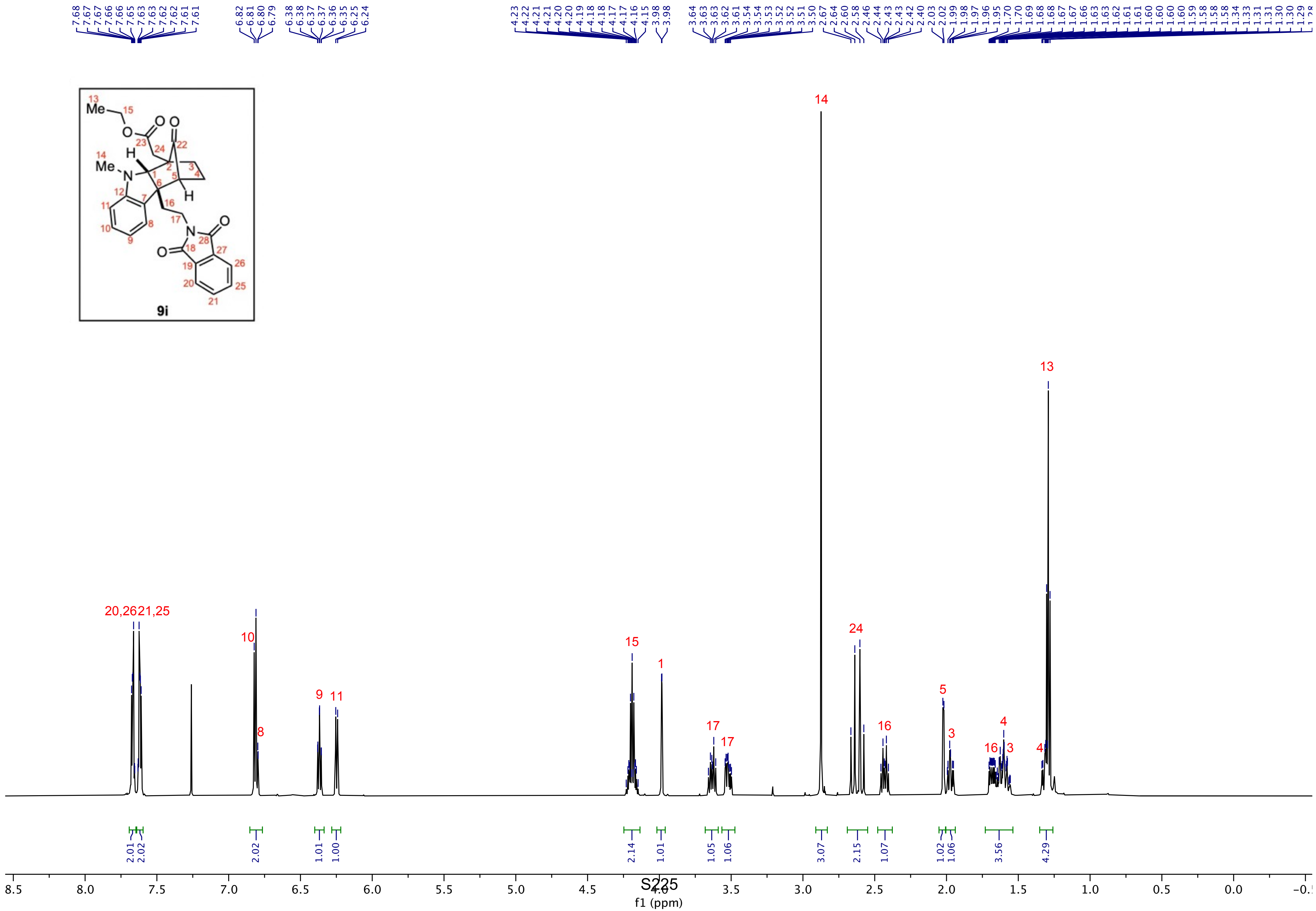
9i
20,26
21,25
10
8
9
11
15
1
17
17
14
24
16
5
3
16
4
3
4
13
f1 (ppm)

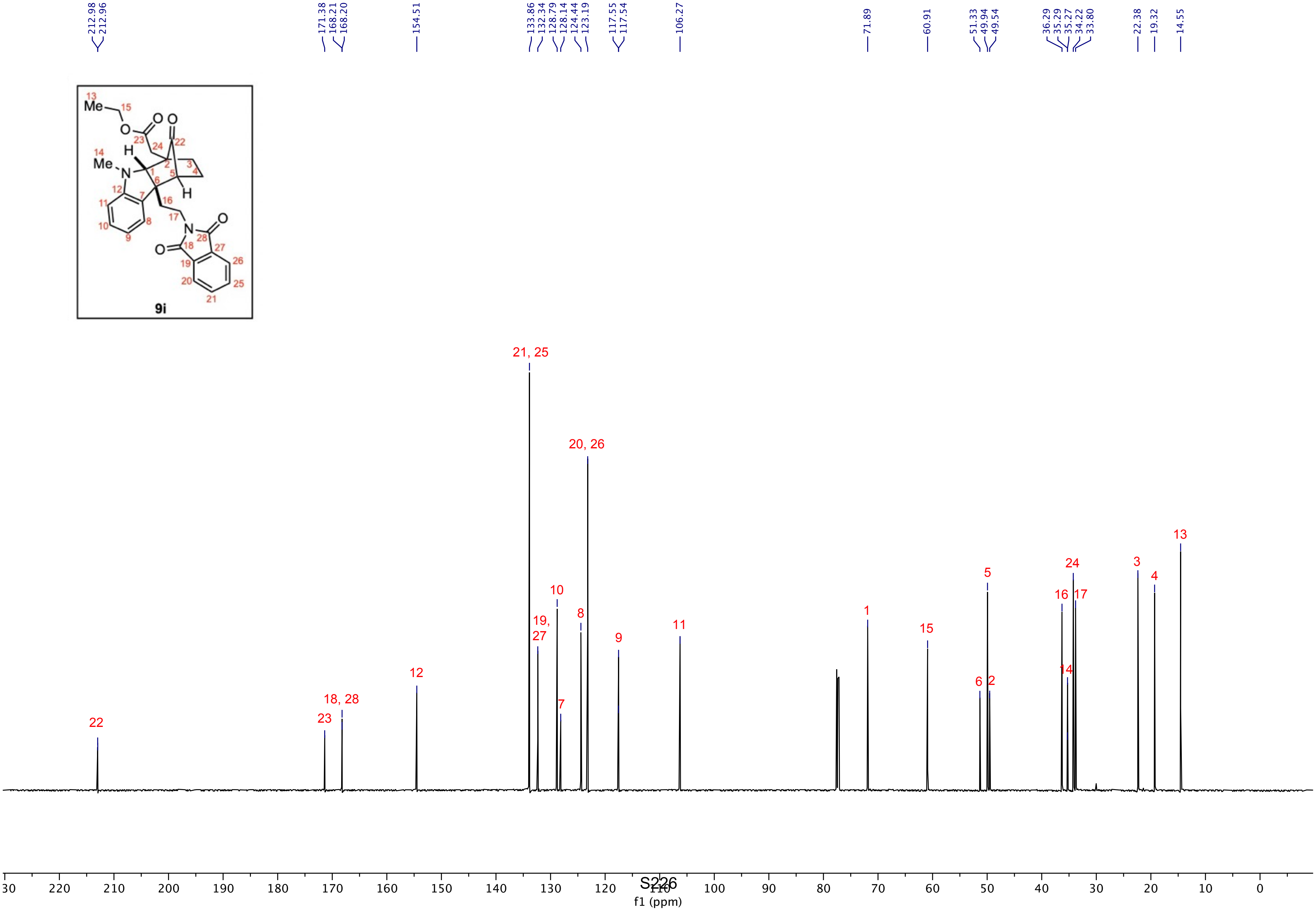
212.98
212.96
171.38
168.21
168.20
154.51
133.86
132.34
128.79
128.14
124.44
123.19
117.55
117.54
106.27
71.89
60.91
51.33
49.94
49.54
36.29
35.29
35.27
34.22
33.80
22.38
19.32
14.55
9i
21, 25
20, 26
10
19, 27
8
7
9
11
12
18, 28
23
22
1
15
6
2
5
16
14
24
17
3
4
13
f1 (ppm)

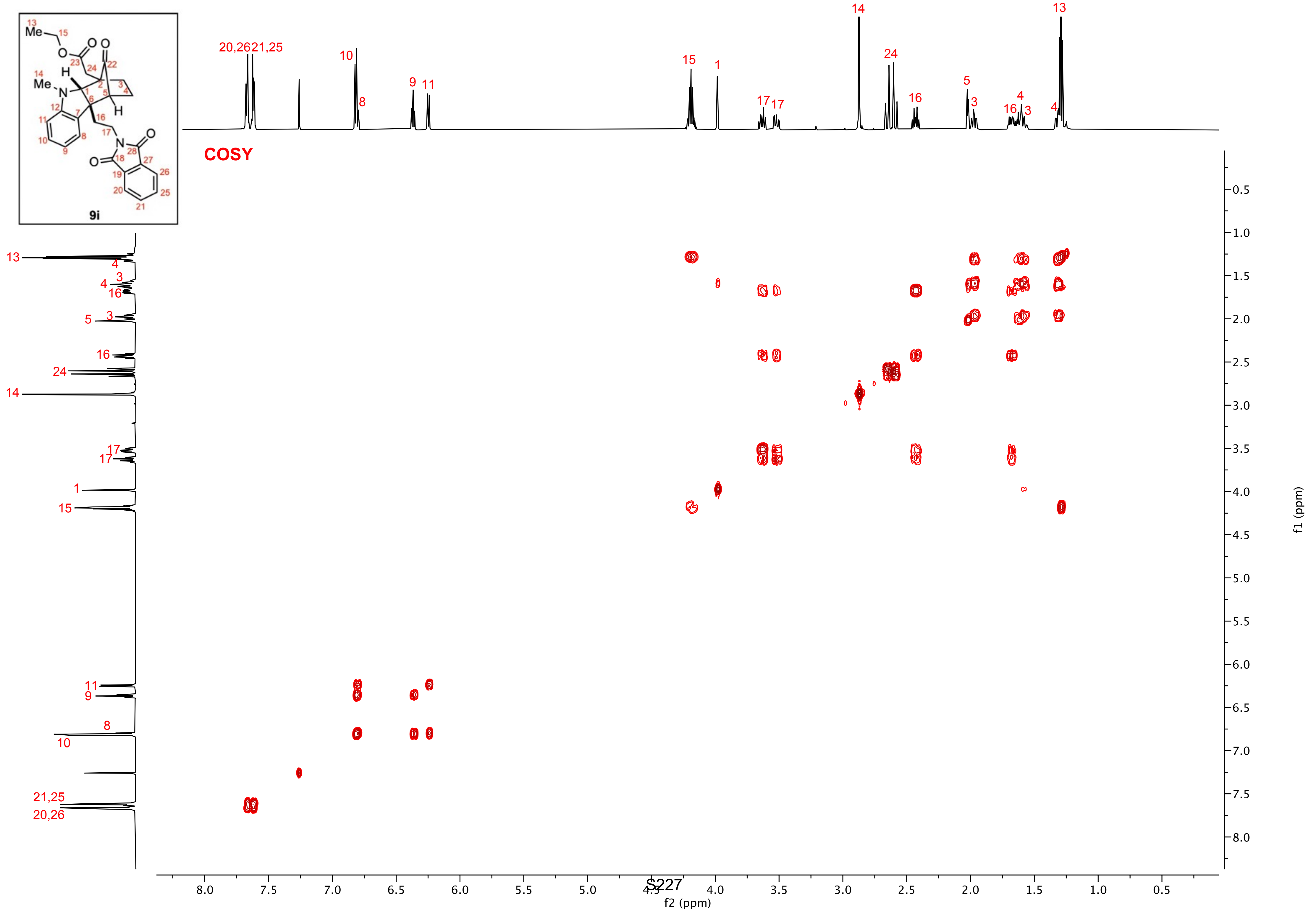
9i
COSY
20,26
21,25
10
8
9
11
15
1
17
14
24
16
5
3
4
13
f2 (ppm)
f1 (ppm)

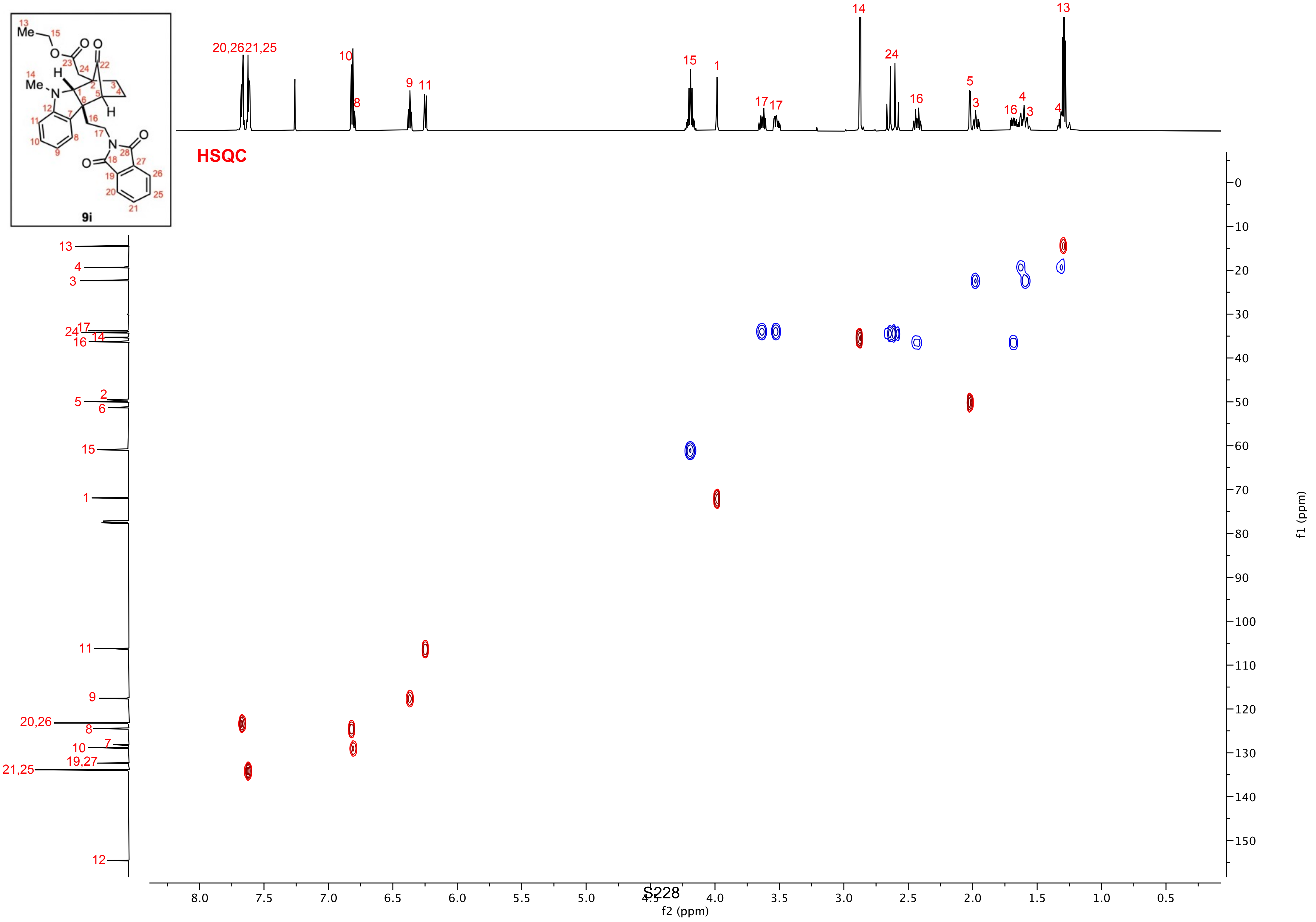
9i
HSQC
f2 (ppm)
f1 (ppm)

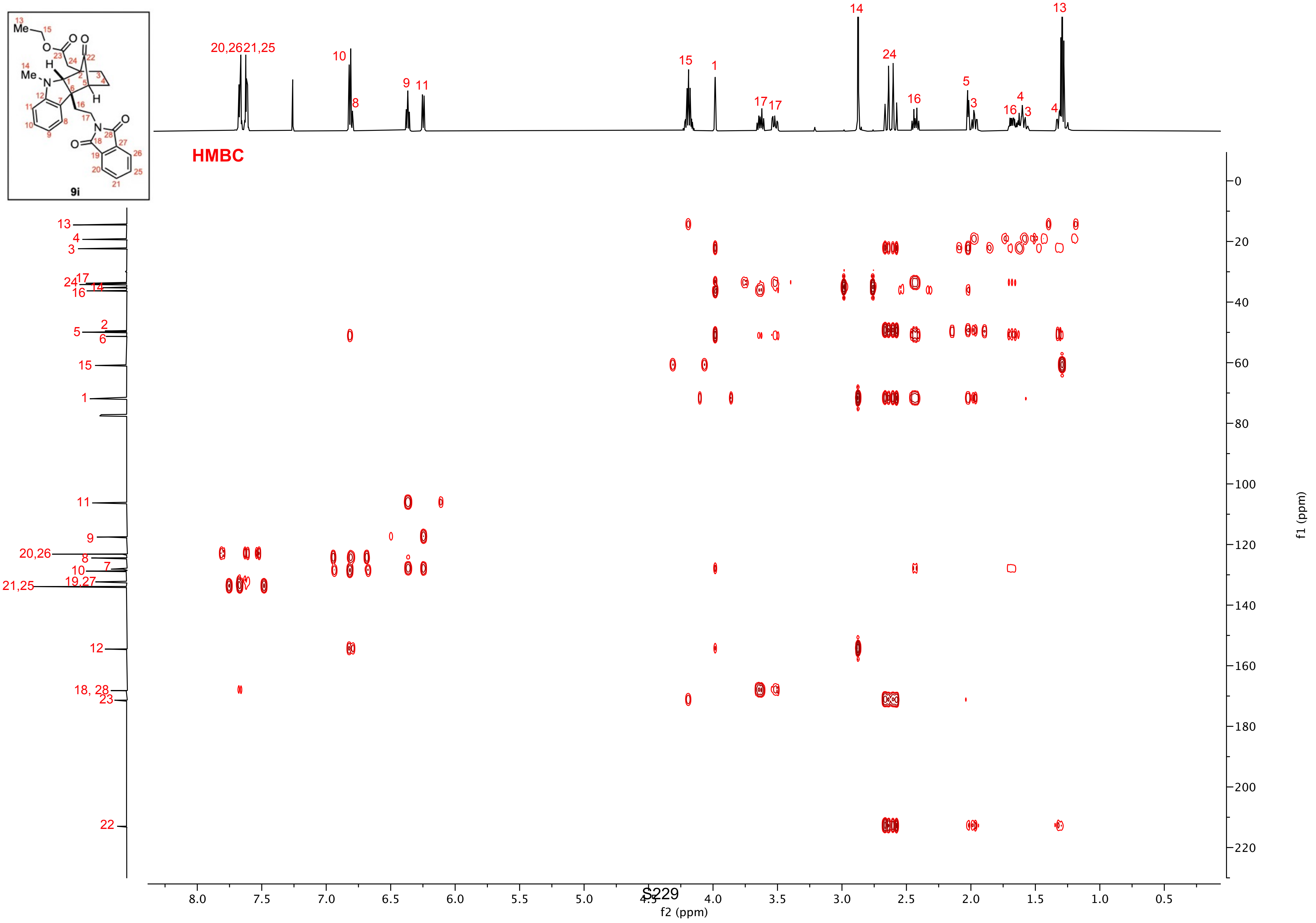
HMBC
9i
f2 (ppm)
f1 (ppm)

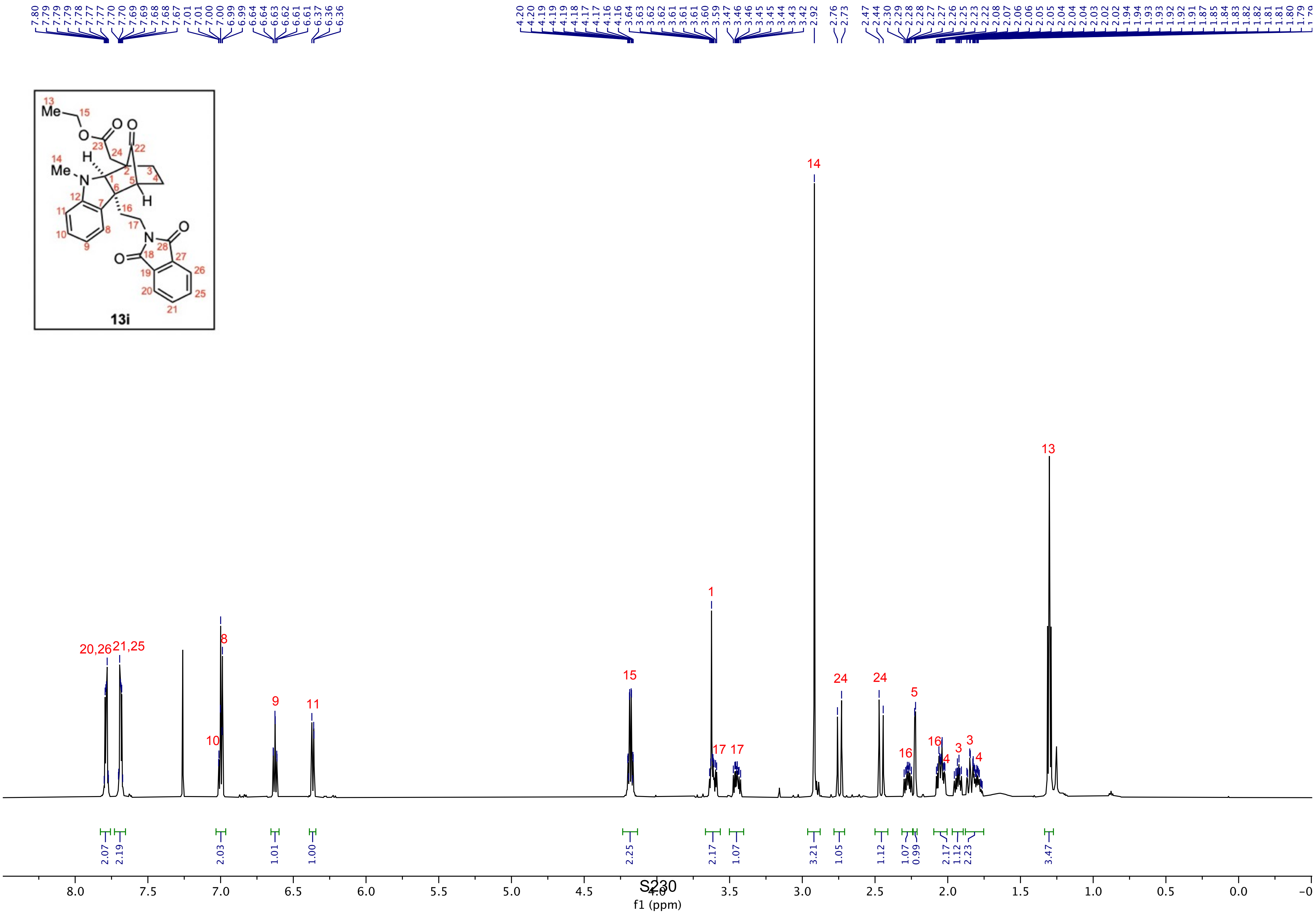
13i
14
13
1
15
24
24
5
20,26
21,25
8
10
9
11
17 17
16
16
4
3
3
4
f1 (ppm)

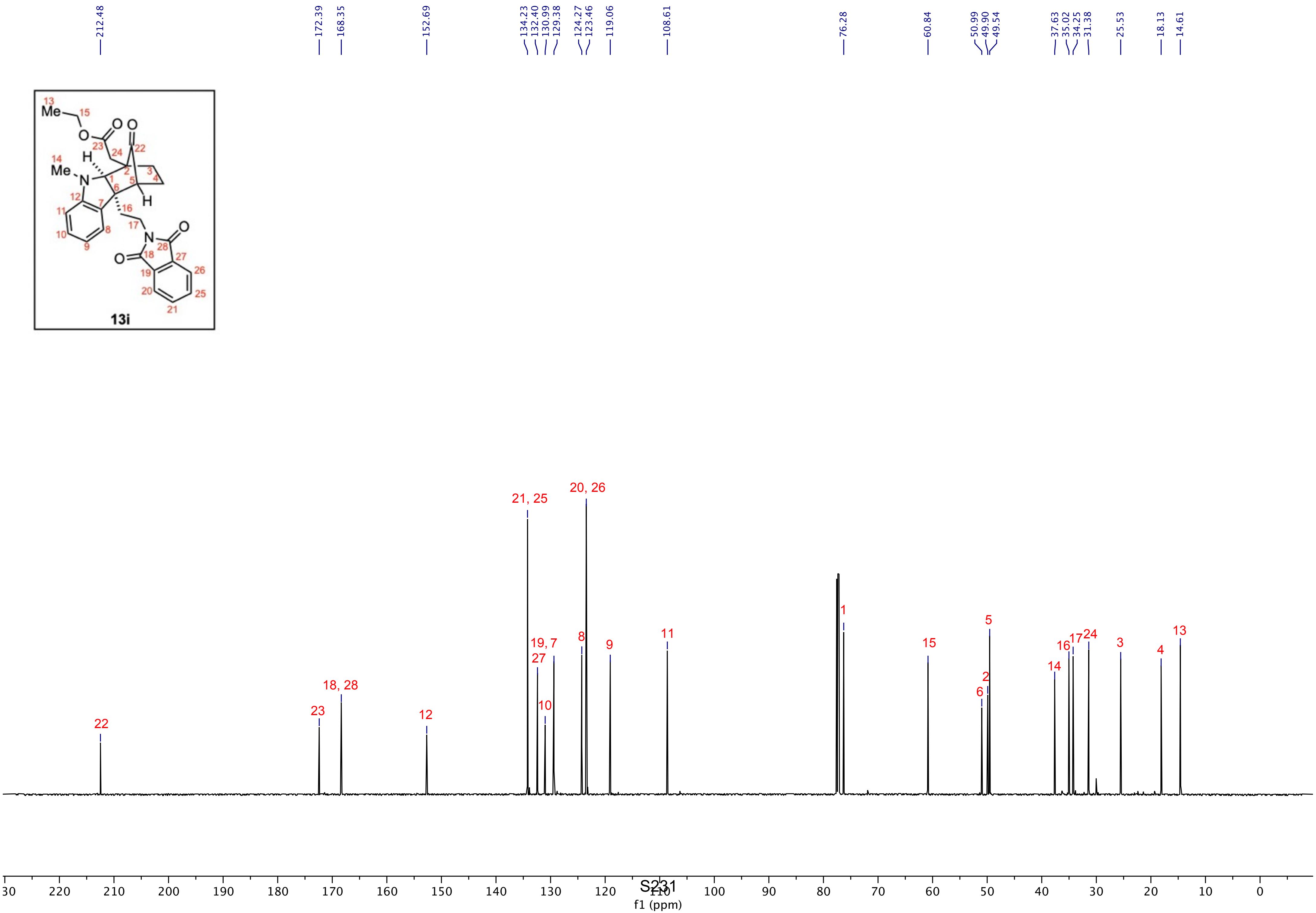

212.48
172.39
168.35
152.69
134.23
132.40
130.99
129.38
124.27
123.46
119.06
108.61
76.28
60.84
50.99
49.90
49.54
37.63
35.02
34.25
31.38
25.53
18.13
14.61
13i
22
23
18, 28
12
21, 25
19, 7
27
10
8
20, 26
9
11
1
15
6
2
5
14
16
17
24
3
4
13
f1 (ppm)

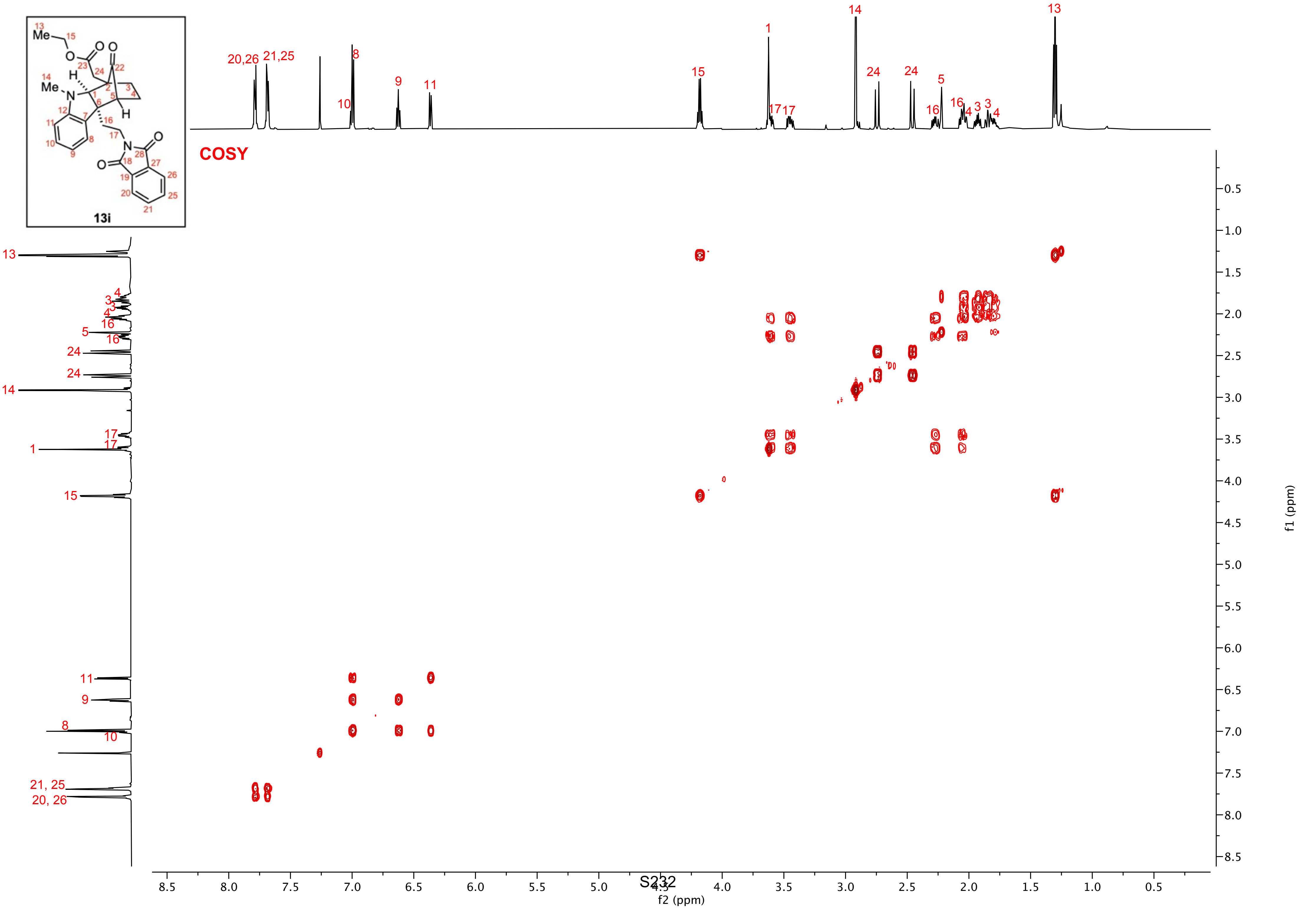
13i
COSY
f2 (ppm)
f1 (ppm)

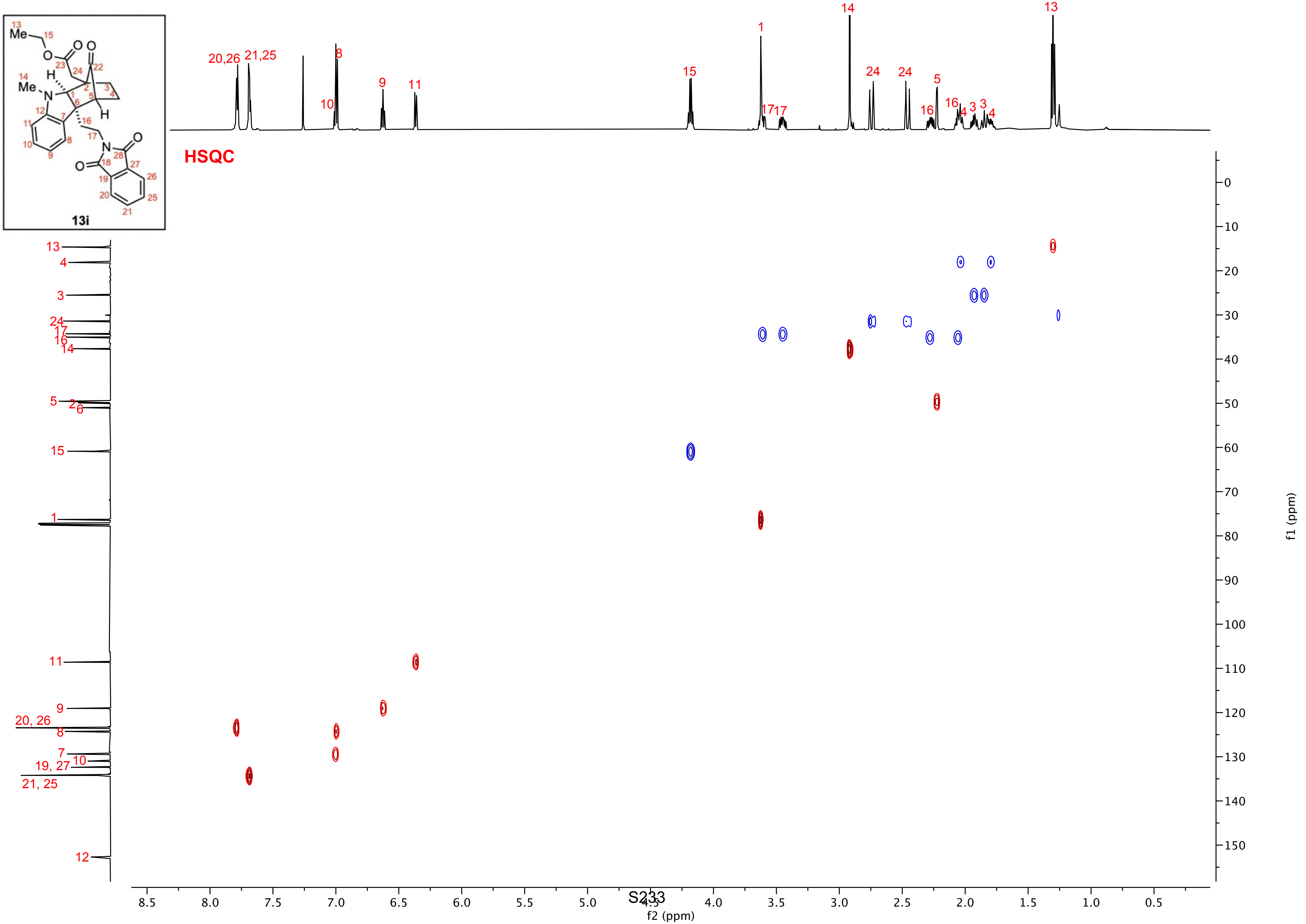
13i
HSQC
f2 (ppm)
f1 (ppm)

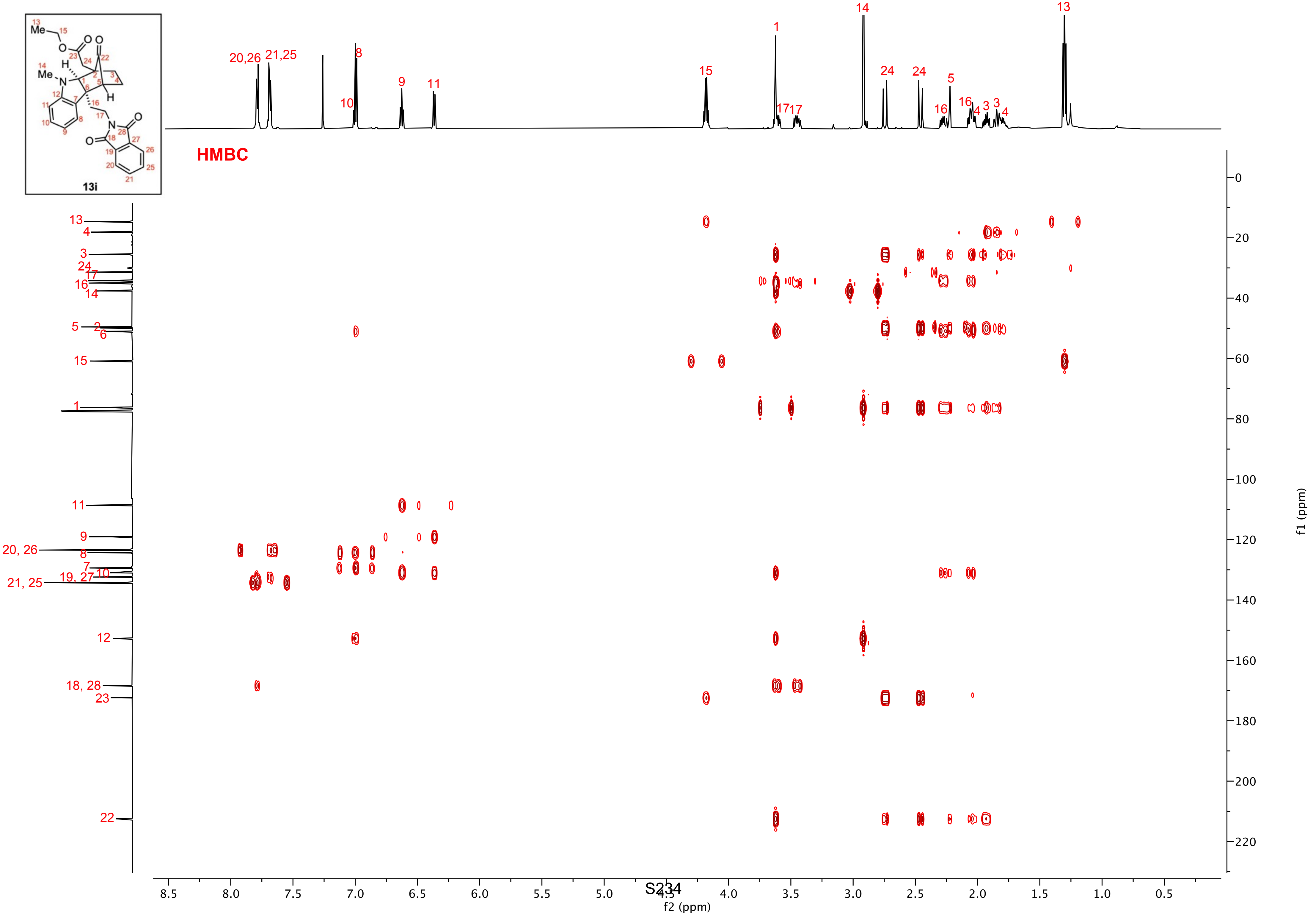
HMBC
13i
20,26
21,25
8
10
9
11
15
1
17
17
14
24
24
5
16
16
4
3
3
4
13
22
23
18, 28
12
21, 25
19, 27
10
7
8
20, 26
9
11
6
2
5
14
16
17
24
f2 (ppm)
f1 (ppm)

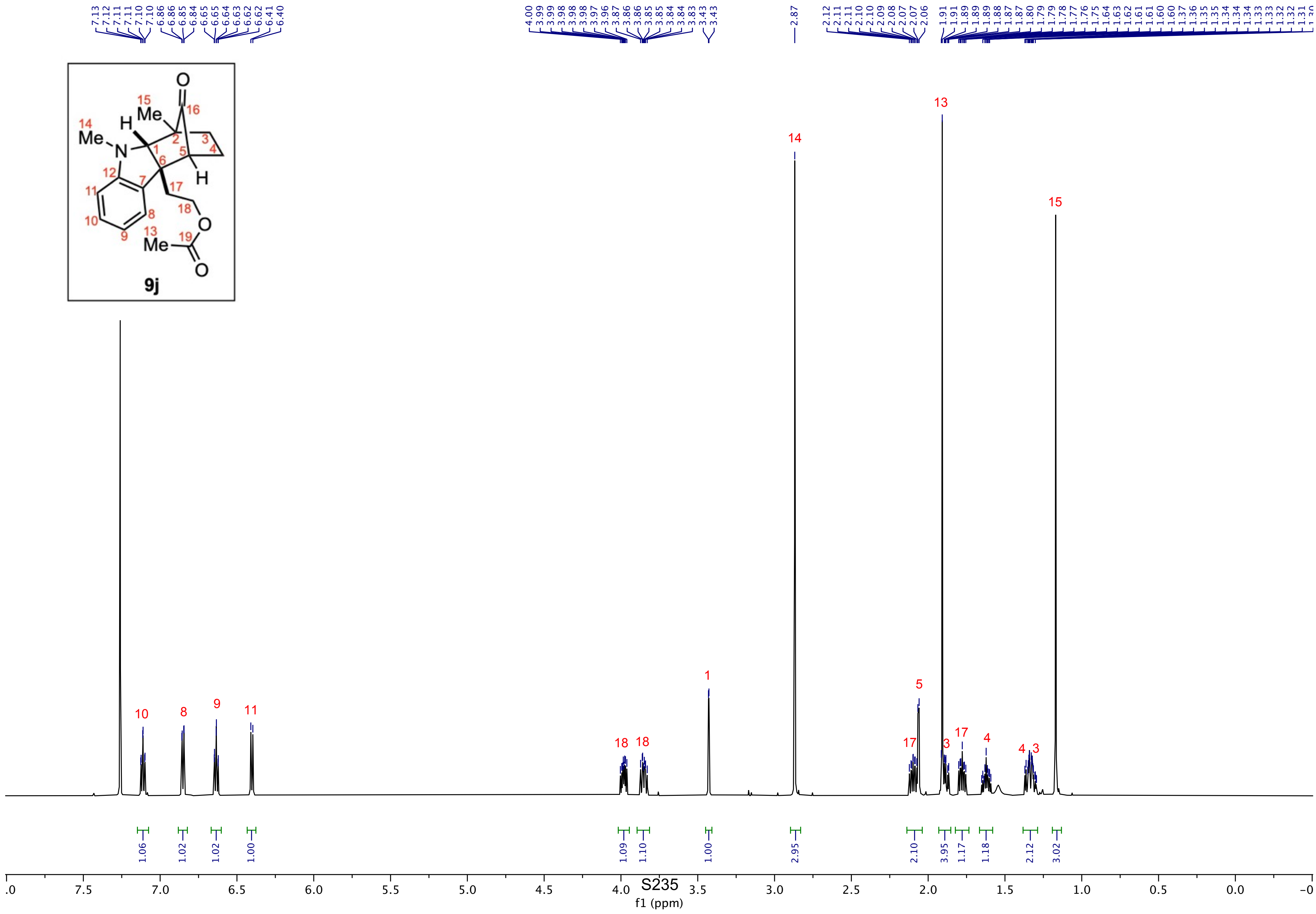
9j
f1 (ppm)

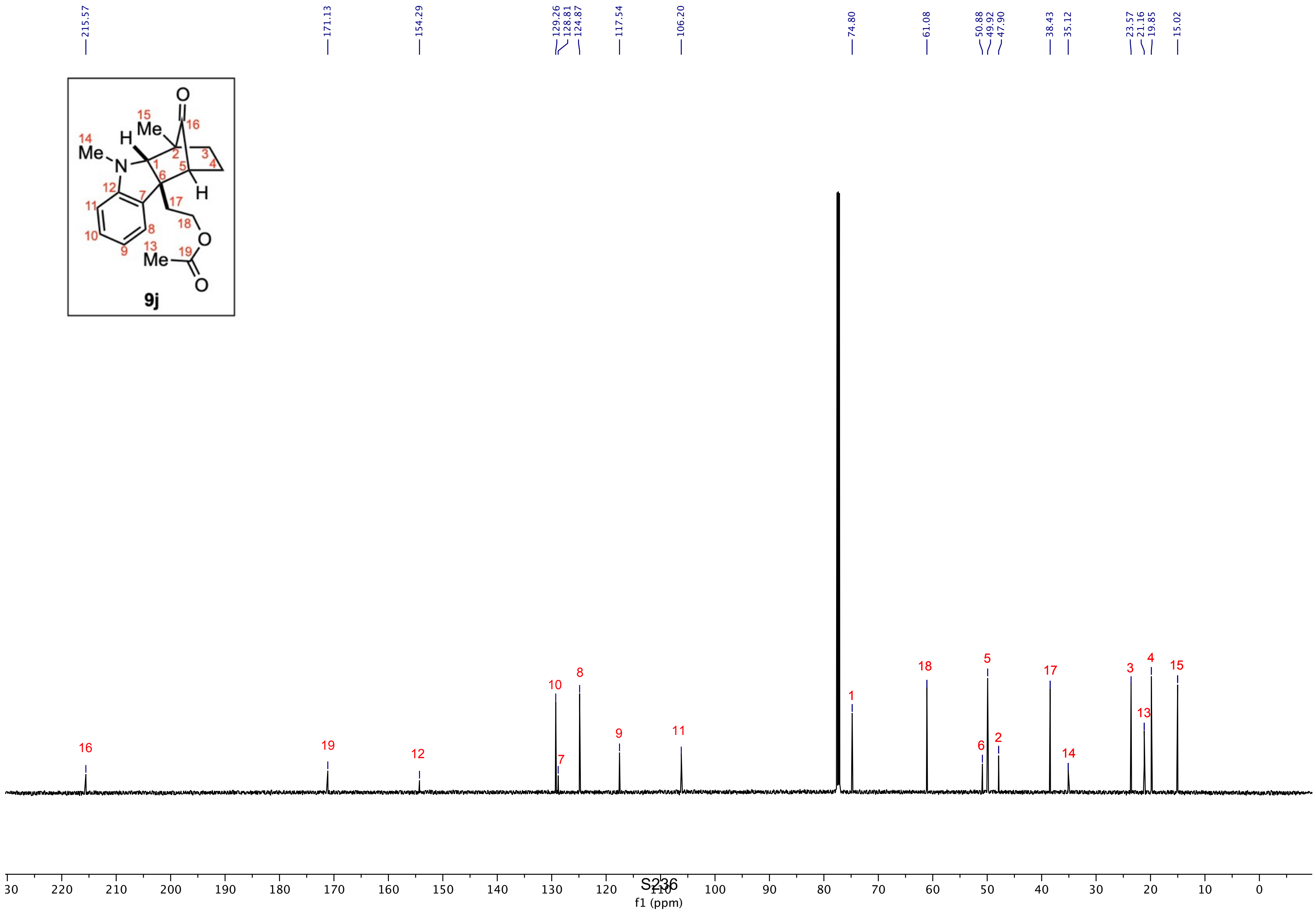
215.57
171.13
154.29
129.26
128.81
124.87
117.54
106.20
74.80
61.08
50.88
49.92
47.90
38.43
35.12
23.57
21.16
19.85
15.02
9j
f1 (ppm)

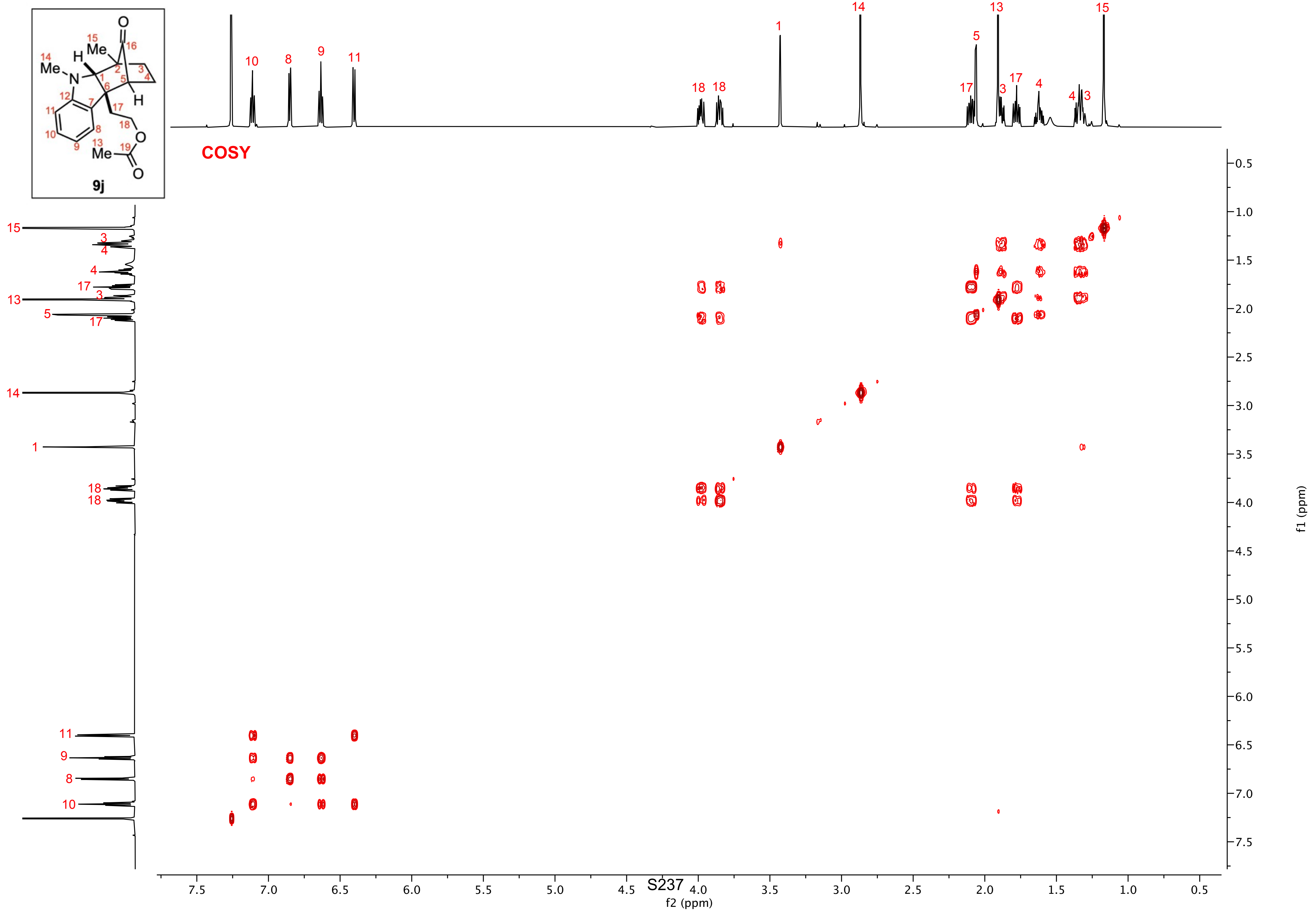
9j
COSY
f2 (ppm)
f1 (ppm)

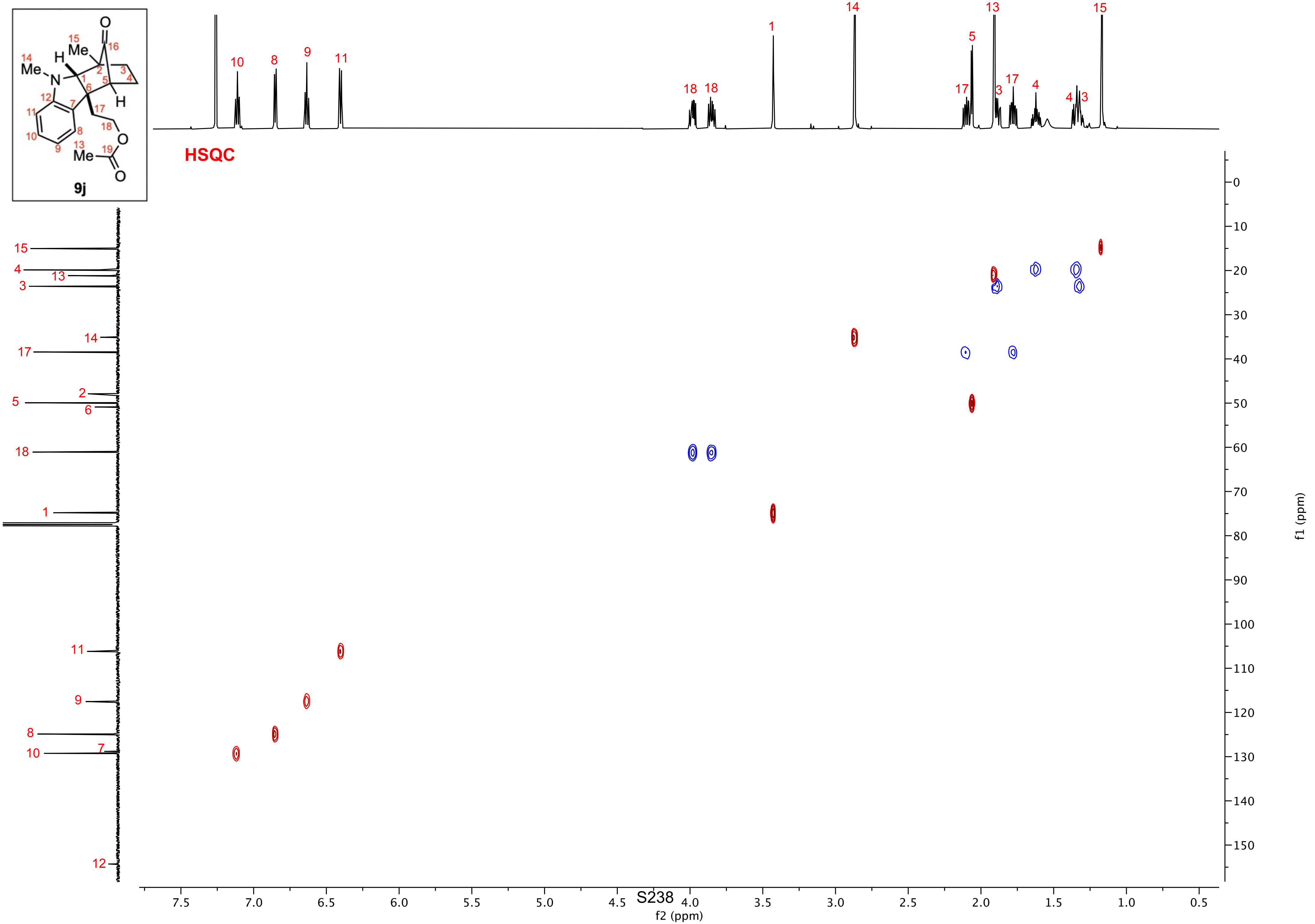

9j
HSQC
f2 (ppm)
f1 (ppm)

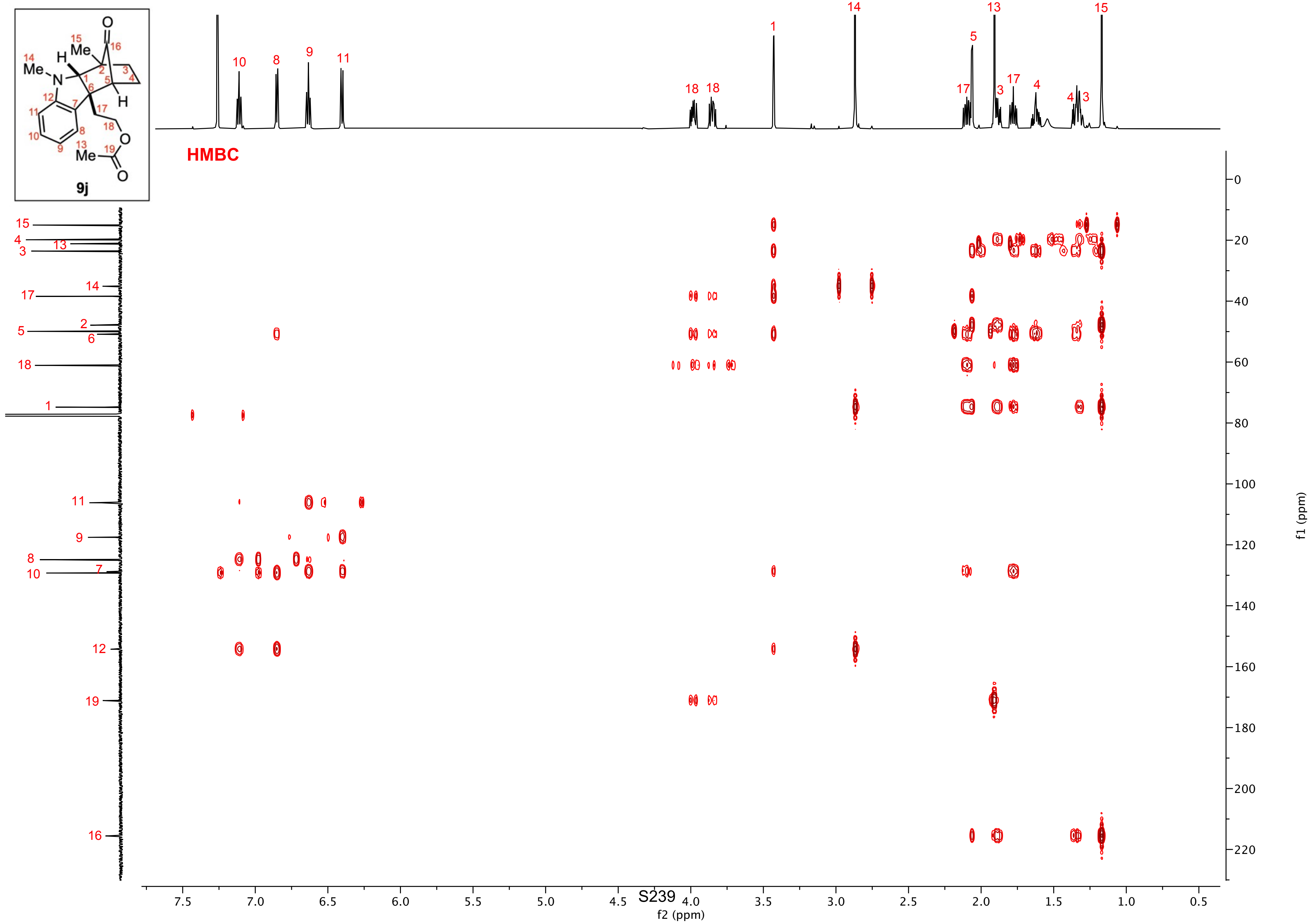
9j
HMBC
f2 (ppm)
f1 (ppm)

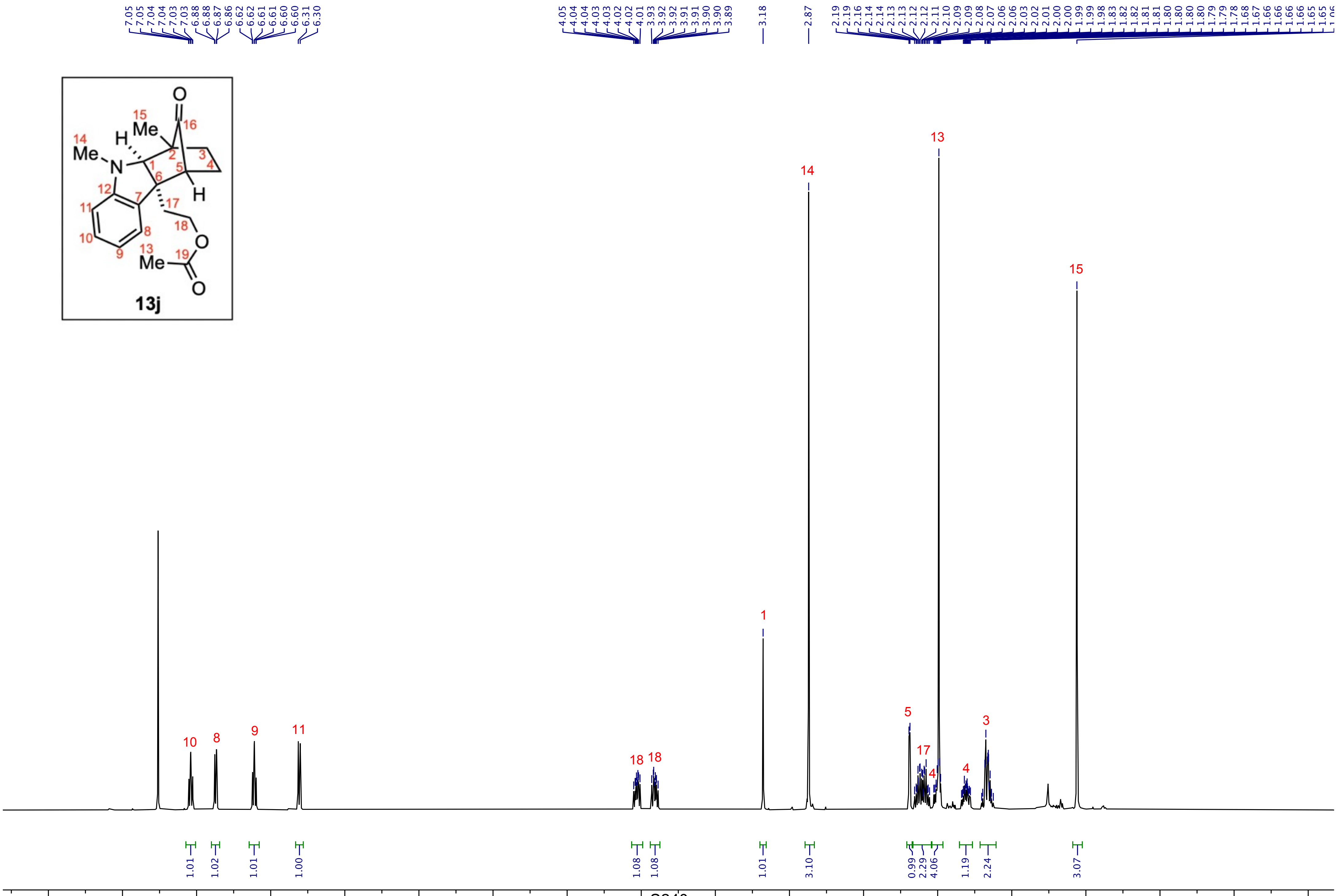
13j
14 Me N H 15 Me O 16 1 2 3 4 5 6 H 7 8 9 10 11 12 17 18 O 13 Me 19 O
10 8 9 11 18 18 1 14 5 17 4 13 4 3 15
1.01 1.02 1.01 1.00 1.08 1.08 1.01 3.10 0.99 2.29 4.06 1.19 2.24 3.07
8.0 7.5 7.0 6.5 6.0 5.5 5.0 4.5 4.0 3.5 3.0 2.5 2.0 1.5 1.0 0.5 0.0 -0.5
f1 (ppm)

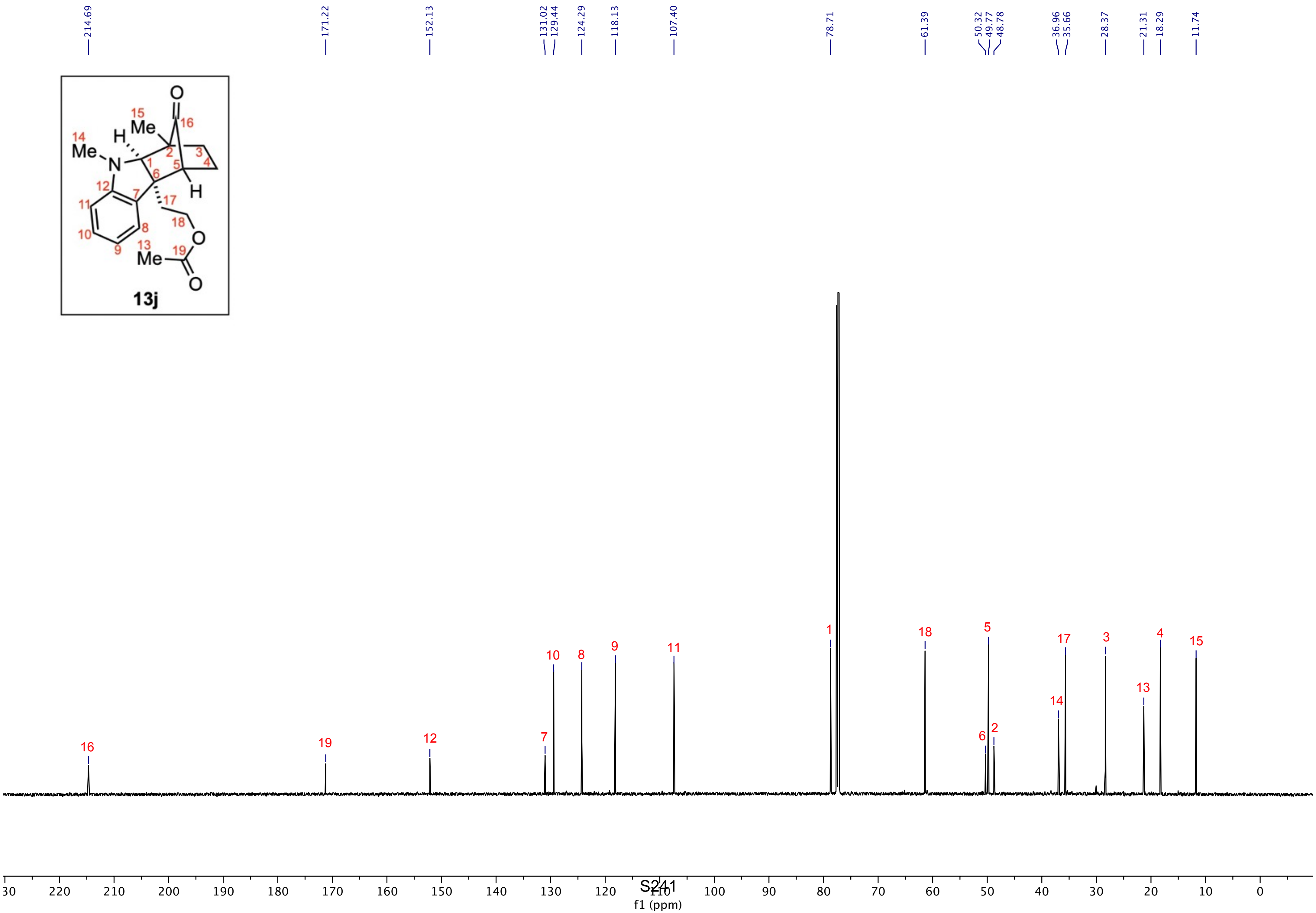

214.69
171.22
152.13
131.02
129.44
124.29
118.13
107.40
78.71
61.39
50.32
49.77
48.78
36.96
35.66
28.37
21.31
18.29
11.74
13j
f1 (ppm)

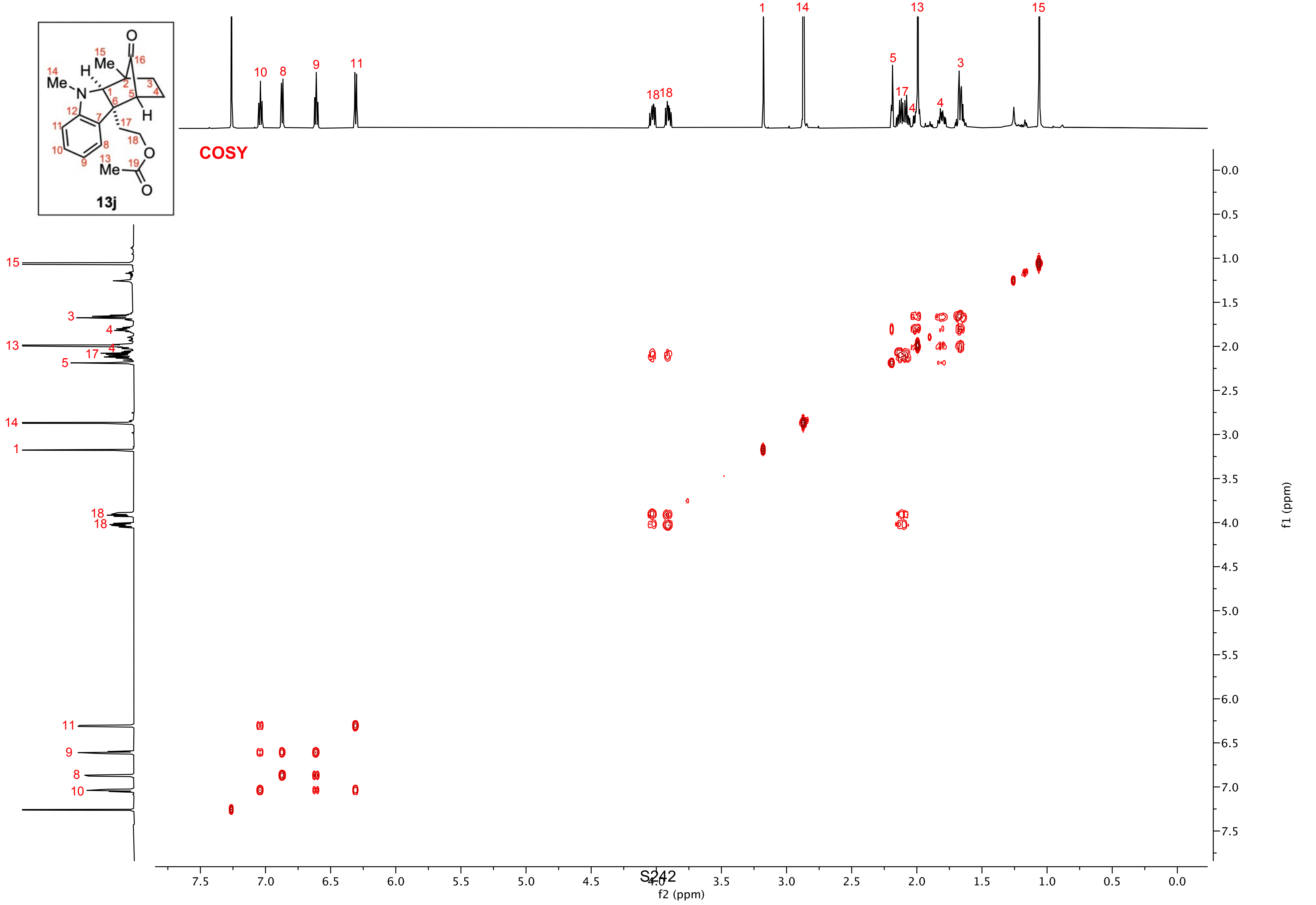
13j
COSY
f2 (ppm)
f1 (ppm)

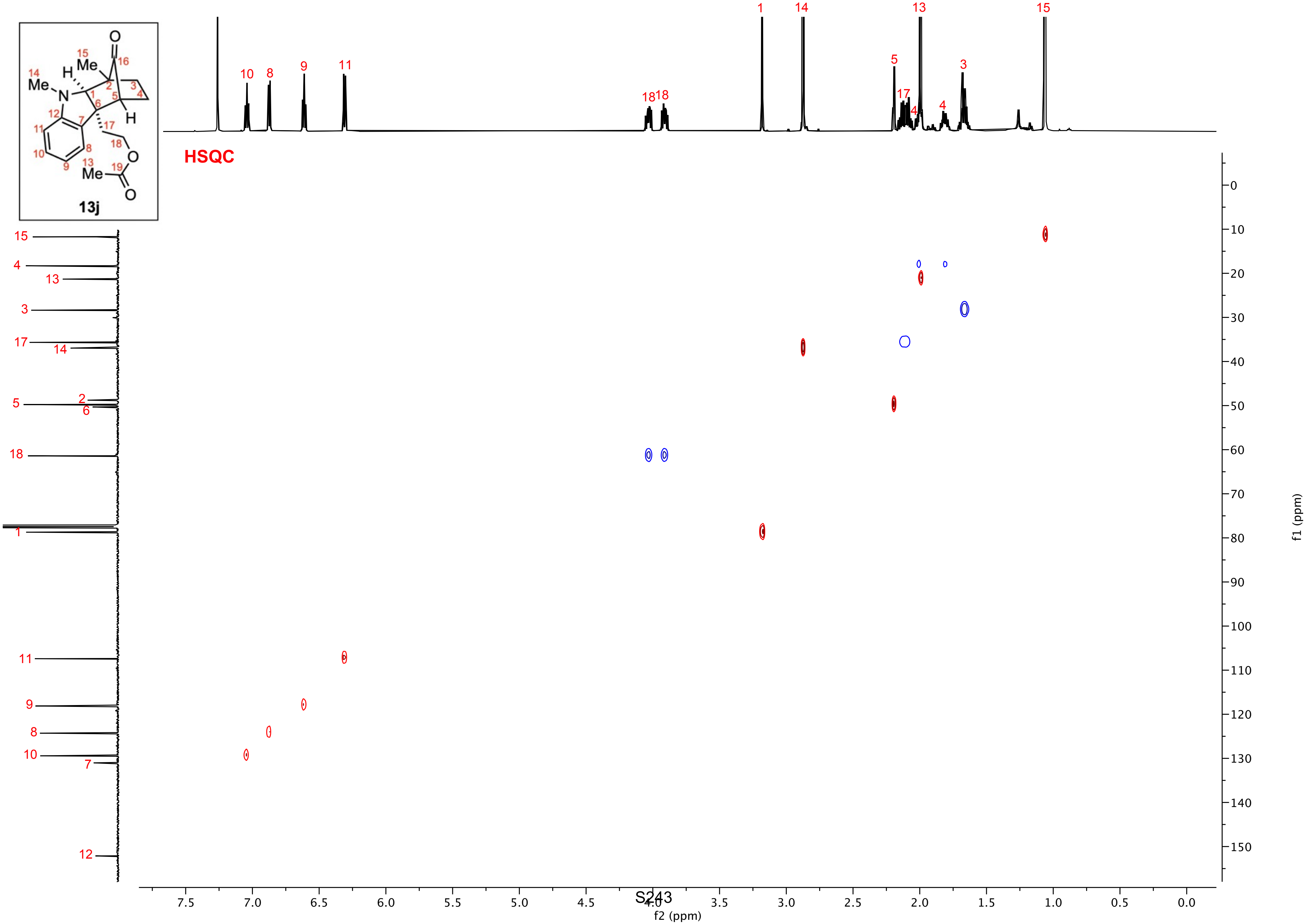
HSQC
13j
f2 (ppm)
f1 (ppm)

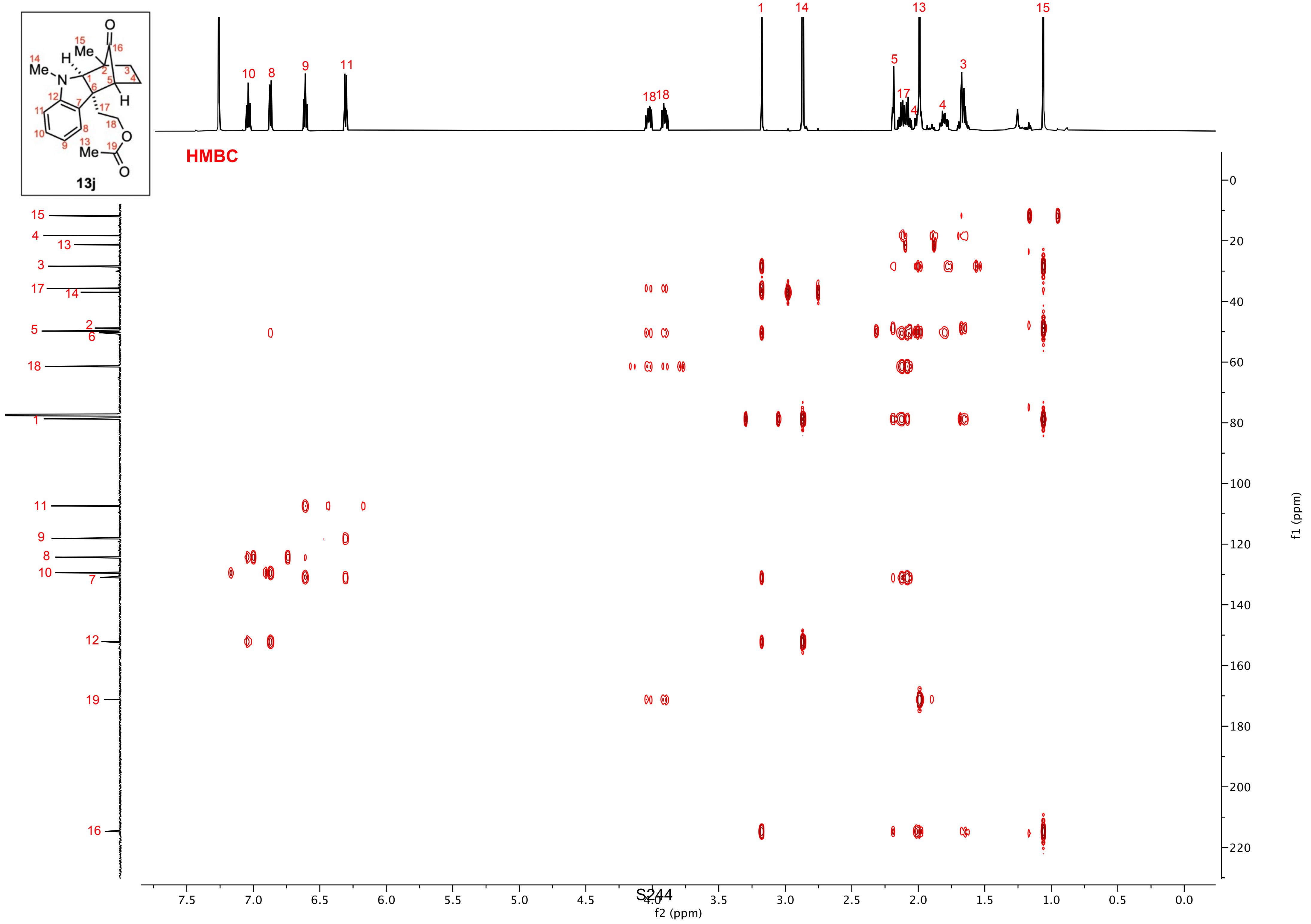

13j
HMBC
f2 (ppm)
f1 (ppm)

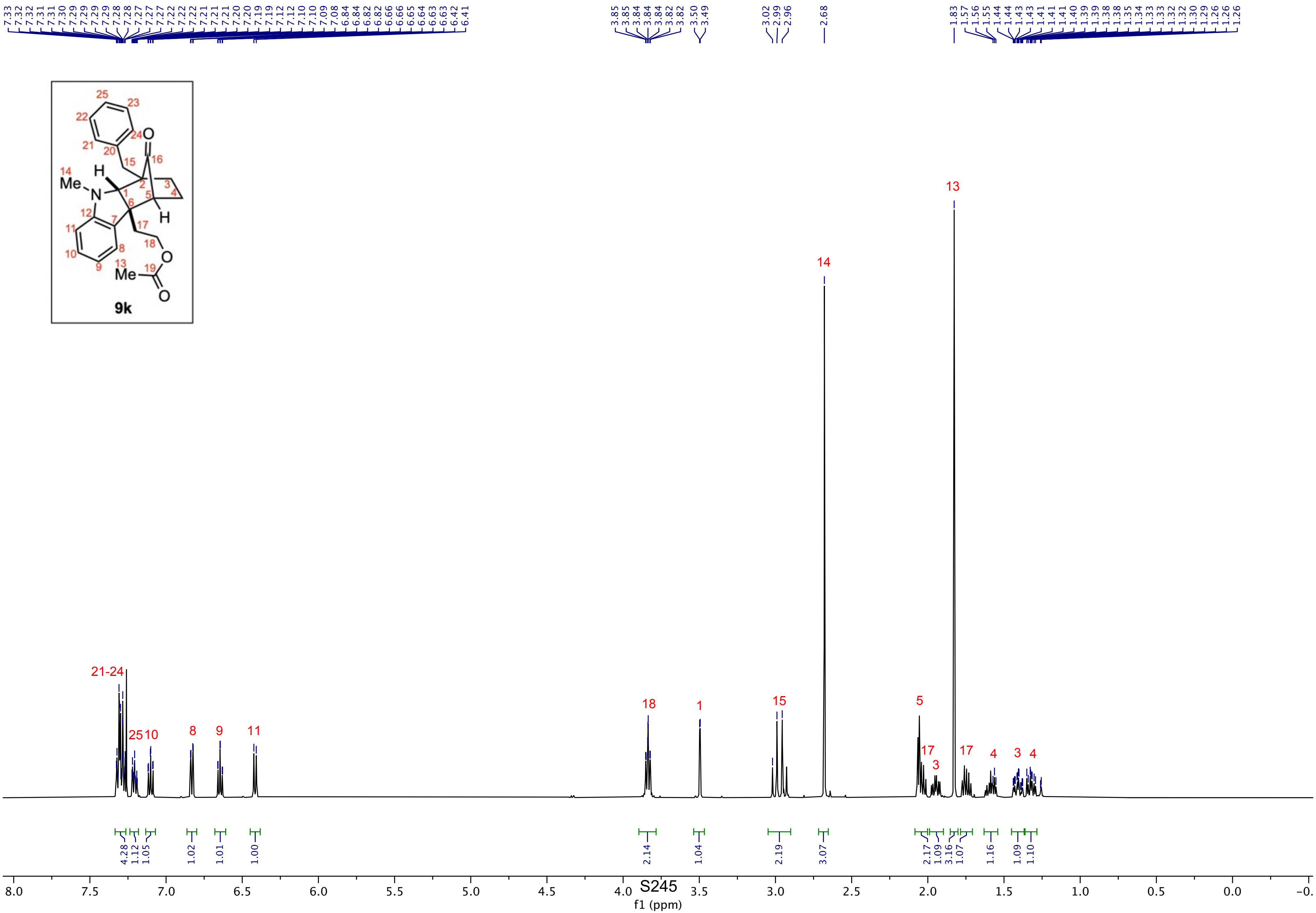
9k
21-24
25
10
8
9
11
18
1
15
14
5
17
3
17
13
4
3
4
f1 (ppm)

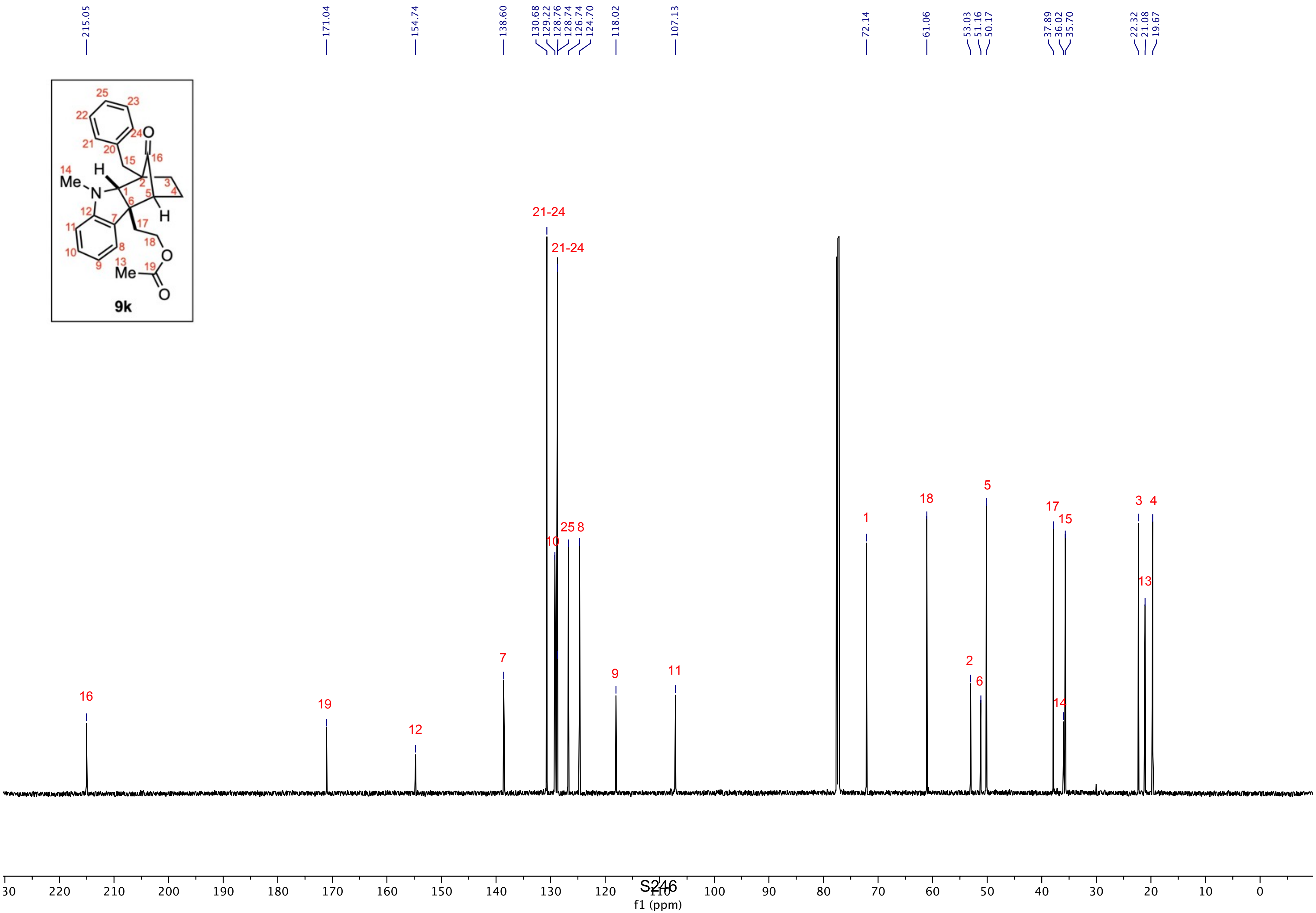
215.05
171.04
154.74
138.60
130.68
129.22
128.76
128.74
126.74
124.70
118.02
107.13
72.14
61.06
53.03
51.16
50.17
37.89
36.02
35.70
22.32
21.08
19.67
9k
21-24
21-24
f1 (ppm)

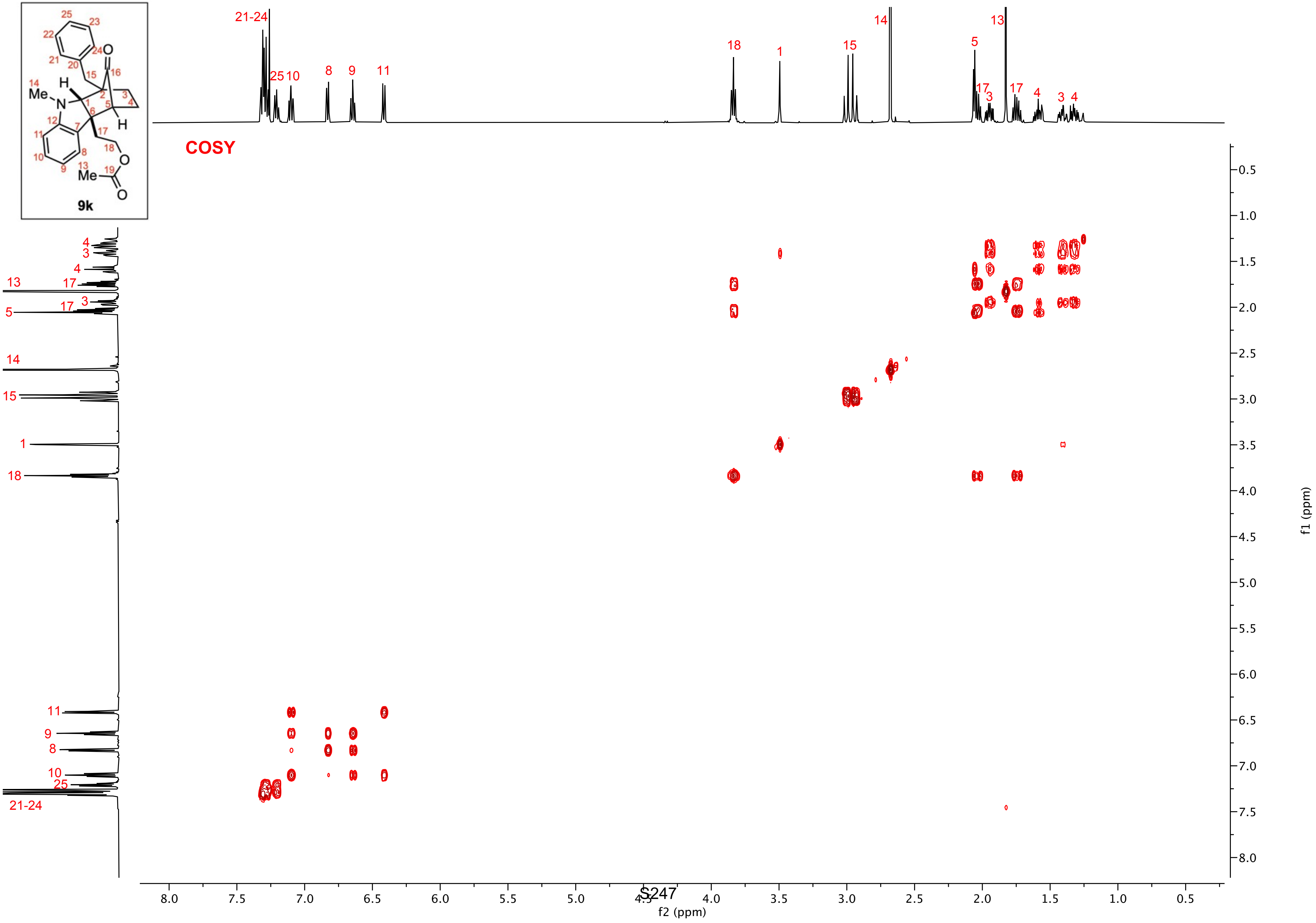
9k
COSY
21-24
25
10
8
9
11
18
1
15
14
5
13
17
3
4
f2 (ppm)
f1 (ppm)

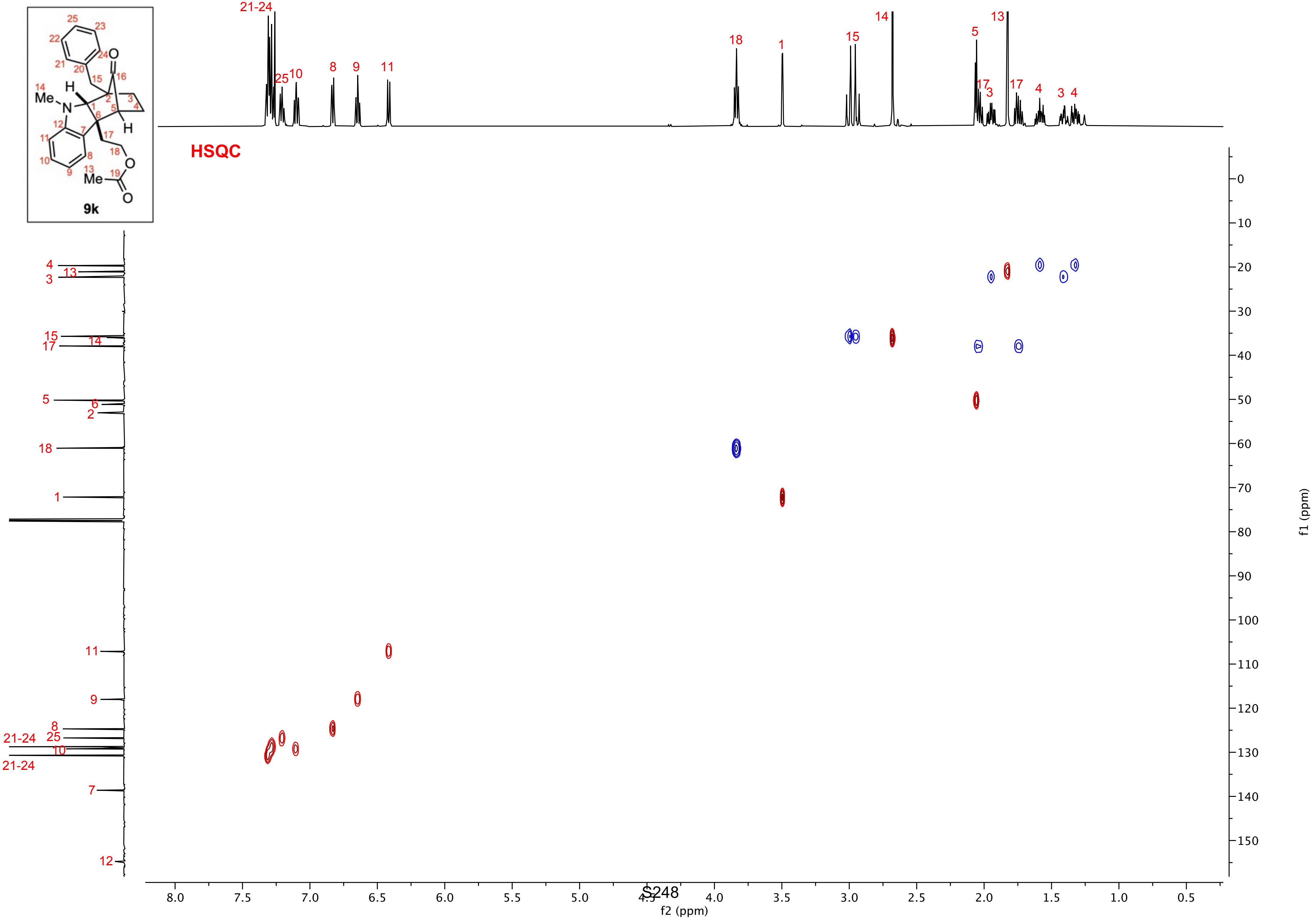
HSQC
9k
21-24
25
10
8
9
11
18
1
15
14
5
13
17
3
4
6
2
12
7
f2 (ppm)
f1 (ppm)

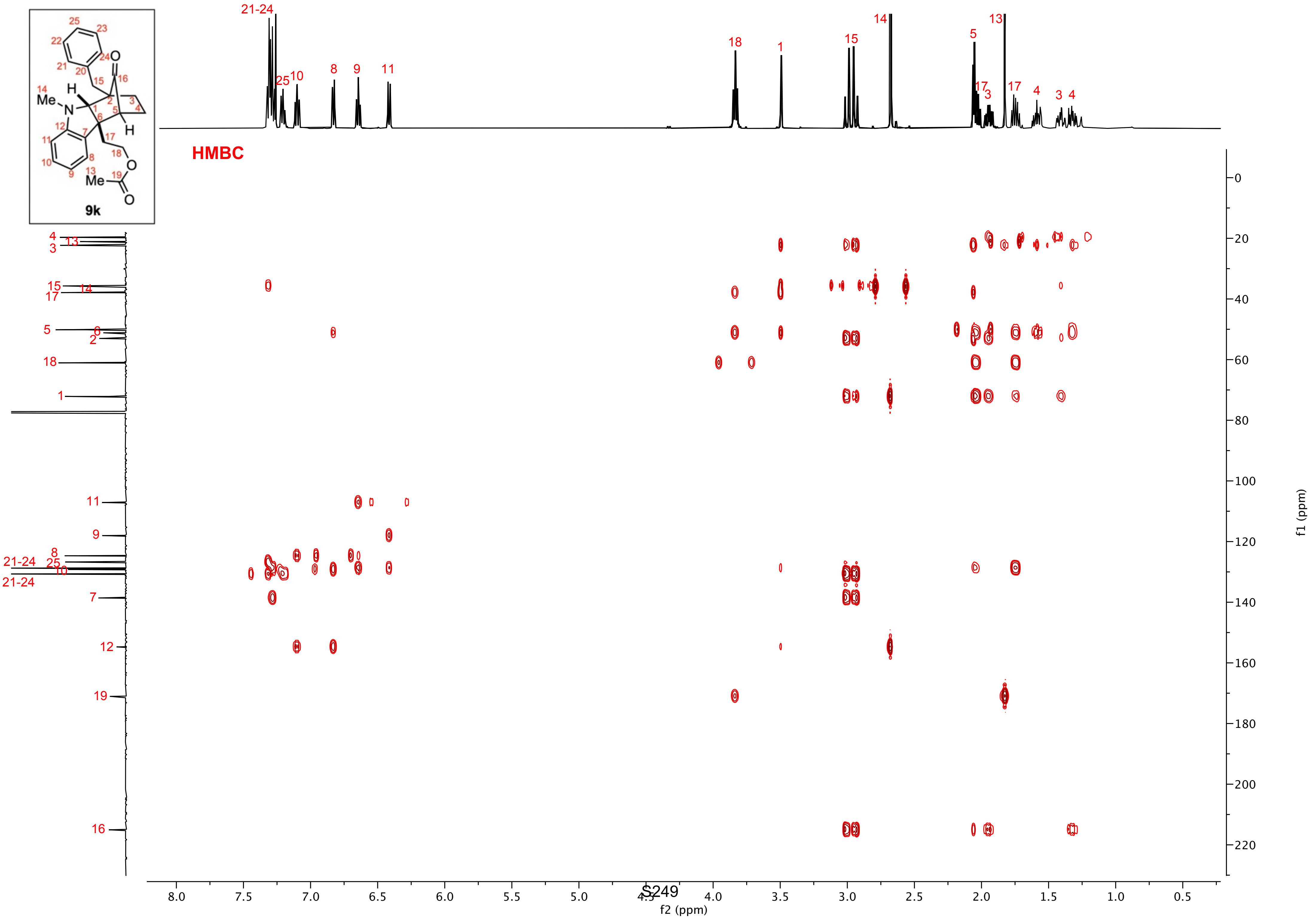
HMBC
9k
21-24
25
10
8
9
11
18
1
15
14
5
13
17
3
4
12
7
19
16
2
6
f2 (ppm)
f1 (ppm)

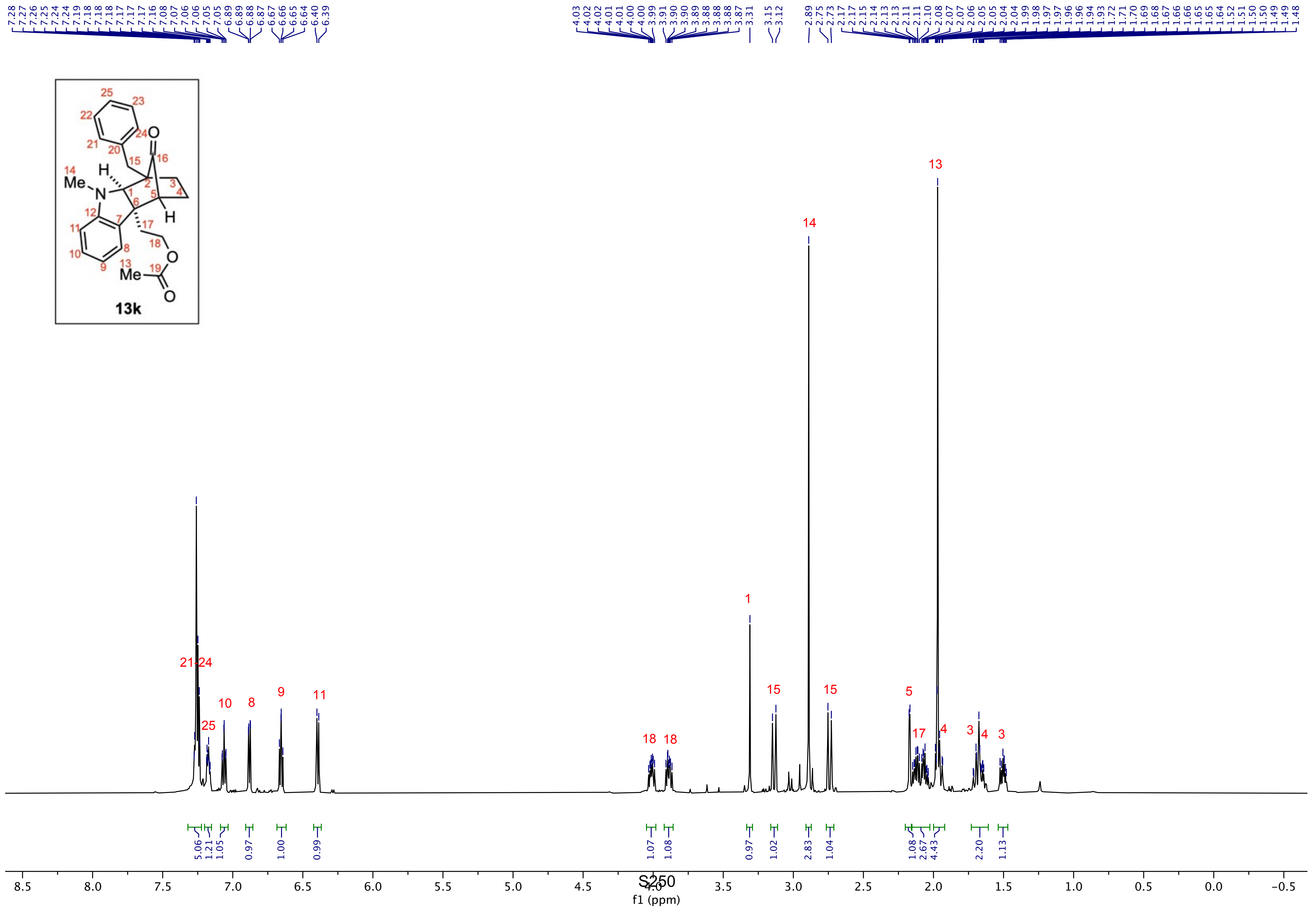

13k
f1 (ppm)

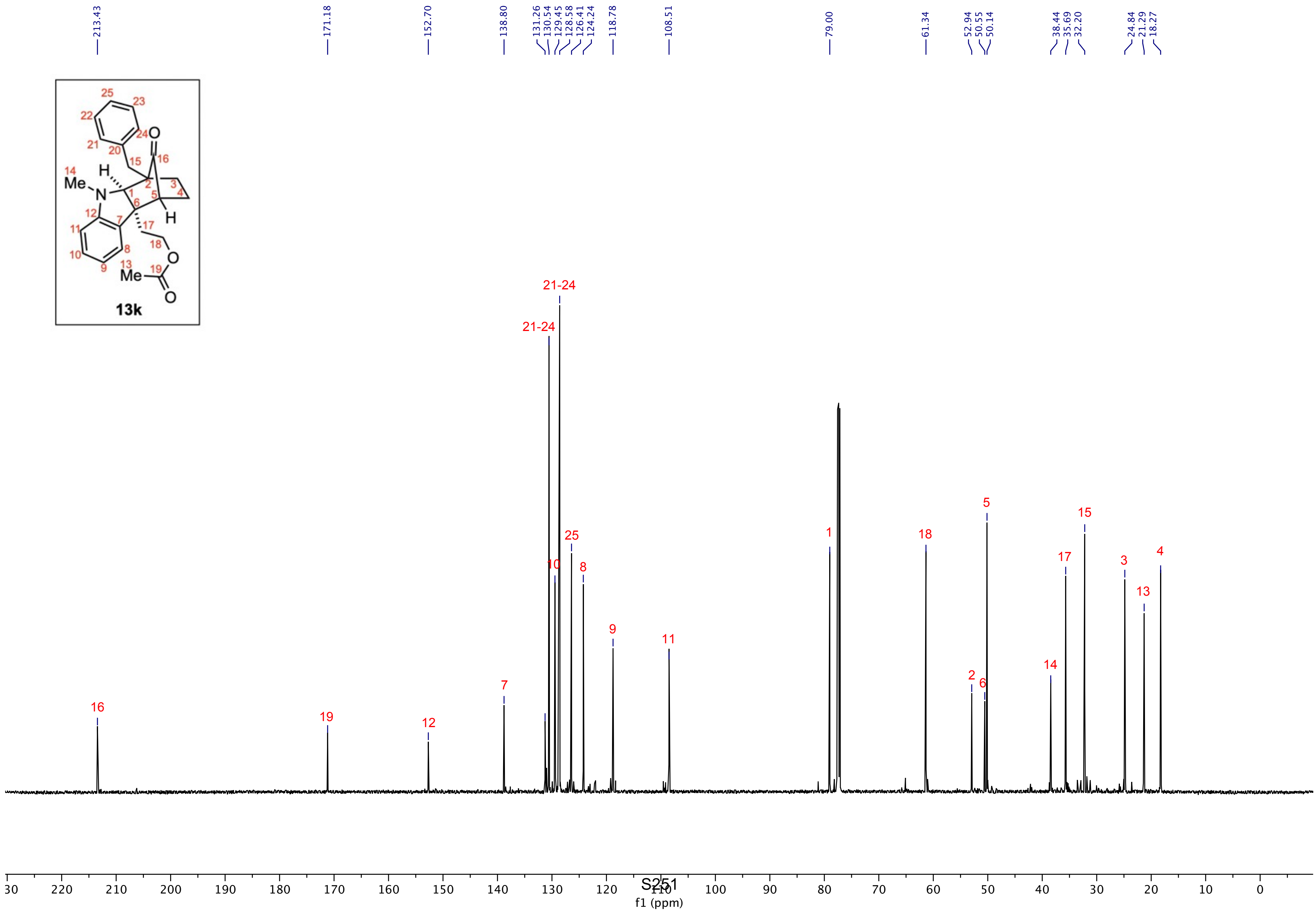
213.43
171.18
152.70
138.80
131.26
130.54
129.45
128.58
126.41
124.24
118.78
108.51
79.00
61.34
52.94
50.55
50.14
38.44
35.69
32.20
24.84
21.29
18.27
13k
f1 (ppm)

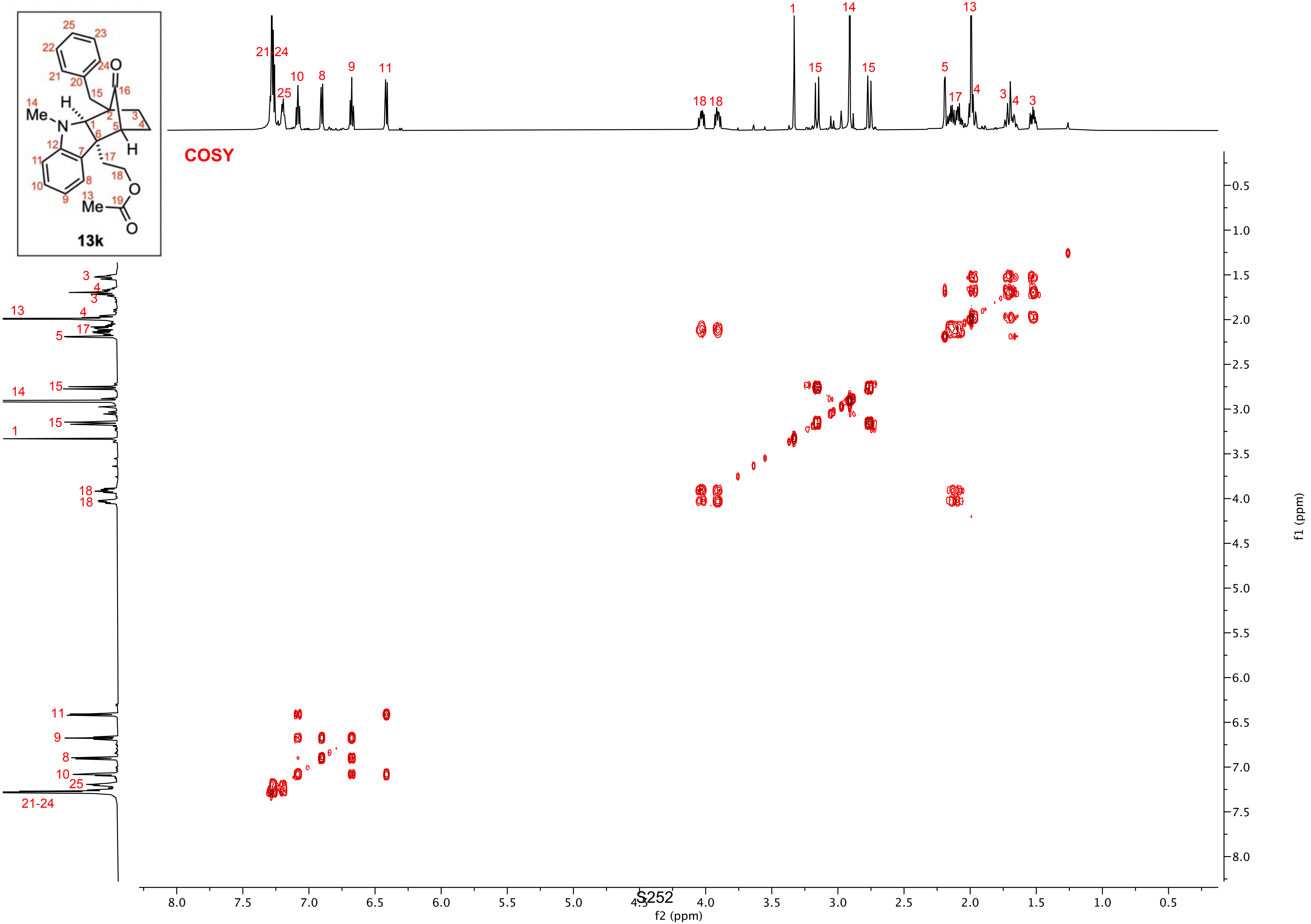

COSY
13k
21-24
25
10
8
9
11
18
18
1
15
14
15
5
17
13
4
3
4
3
f2 (ppm)
f1 (ppm)

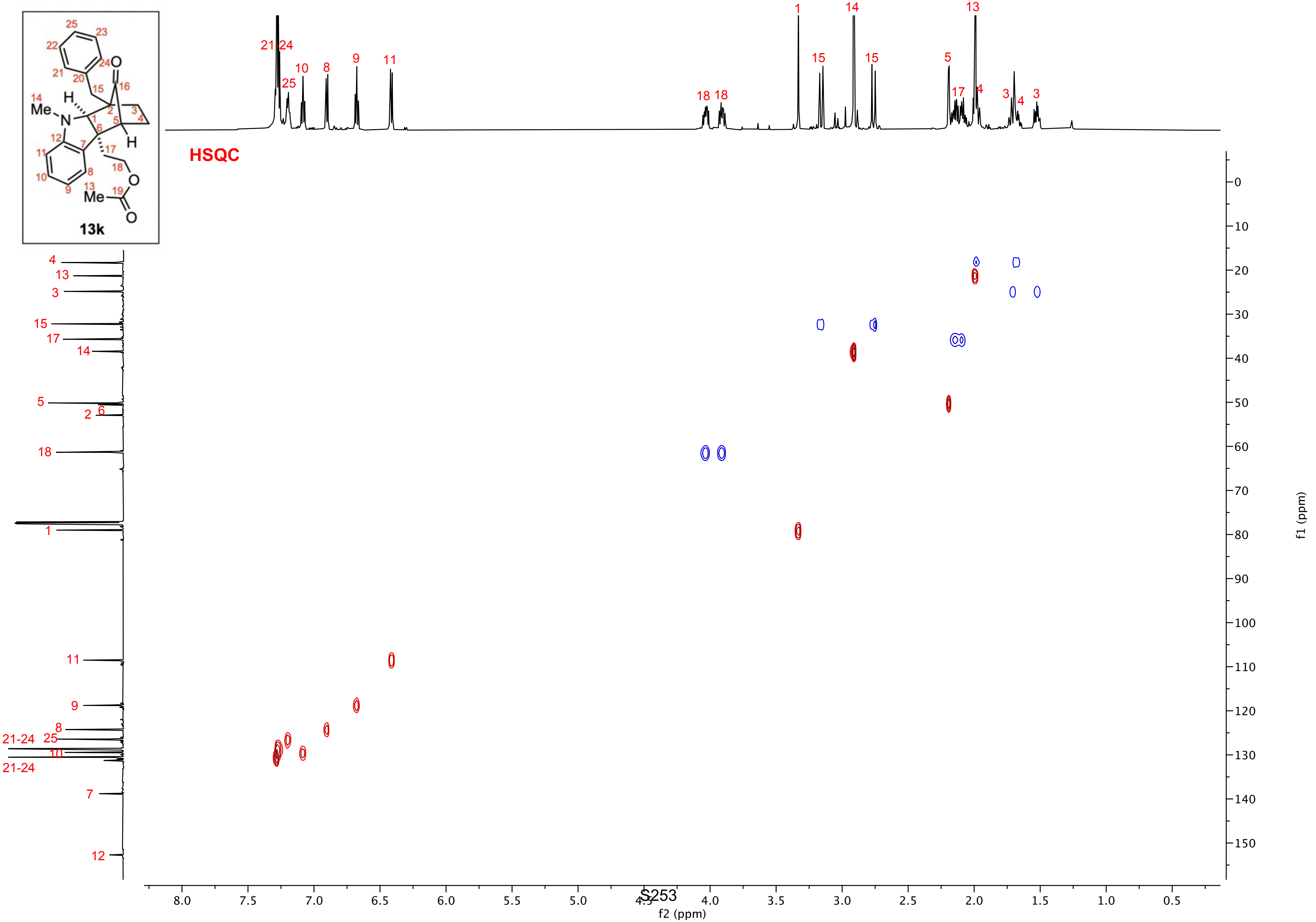

HSQC
13k
Me
N
H
O
H
Me
O
O
1
14
15
13
5
17
4
3
18
10
8
9
11
21-24
25
2
6
7
12
f1 (ppm)
f2 (ppm)

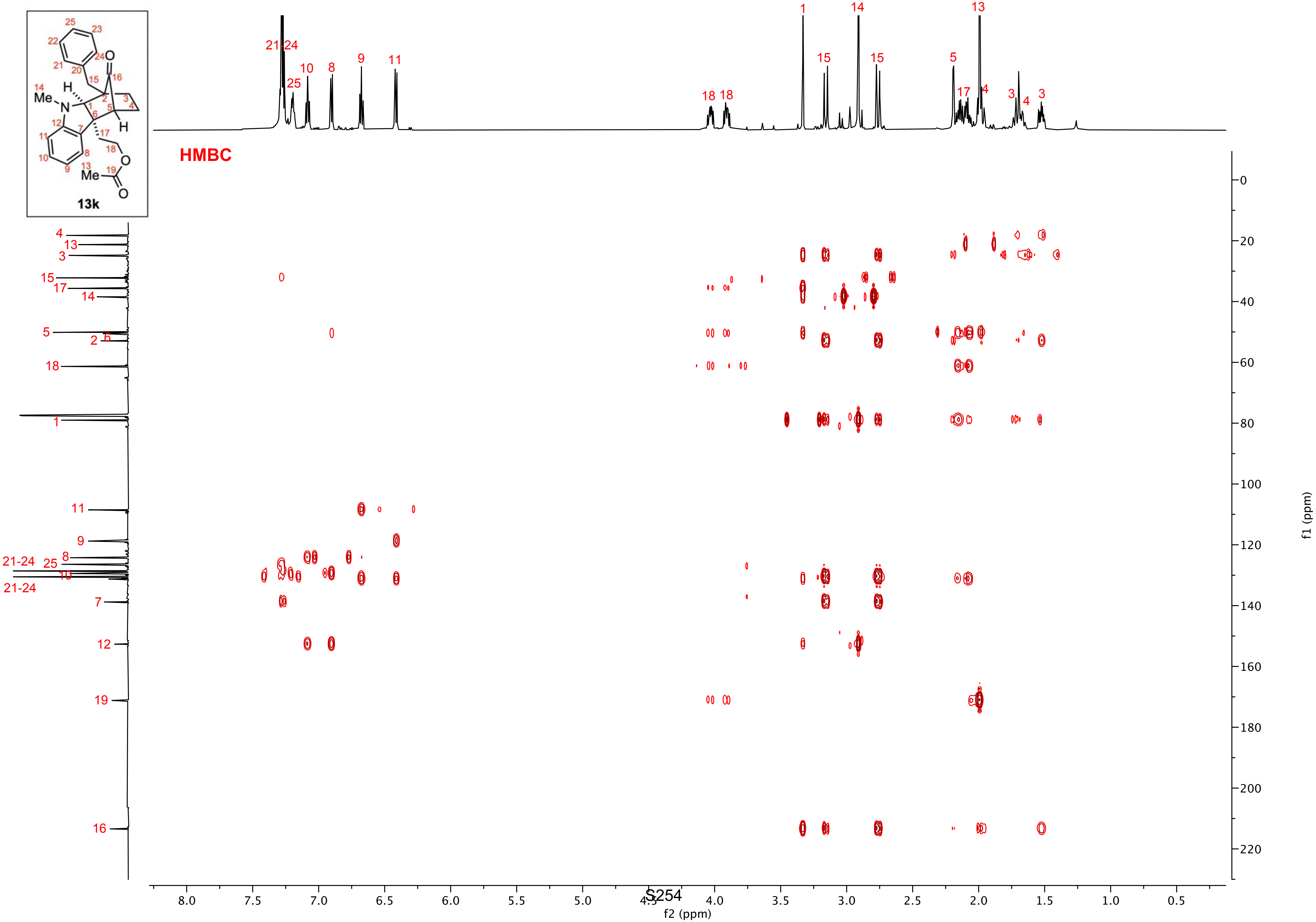

13k
HMBC
f2 (ppm)
f1 (ppm)

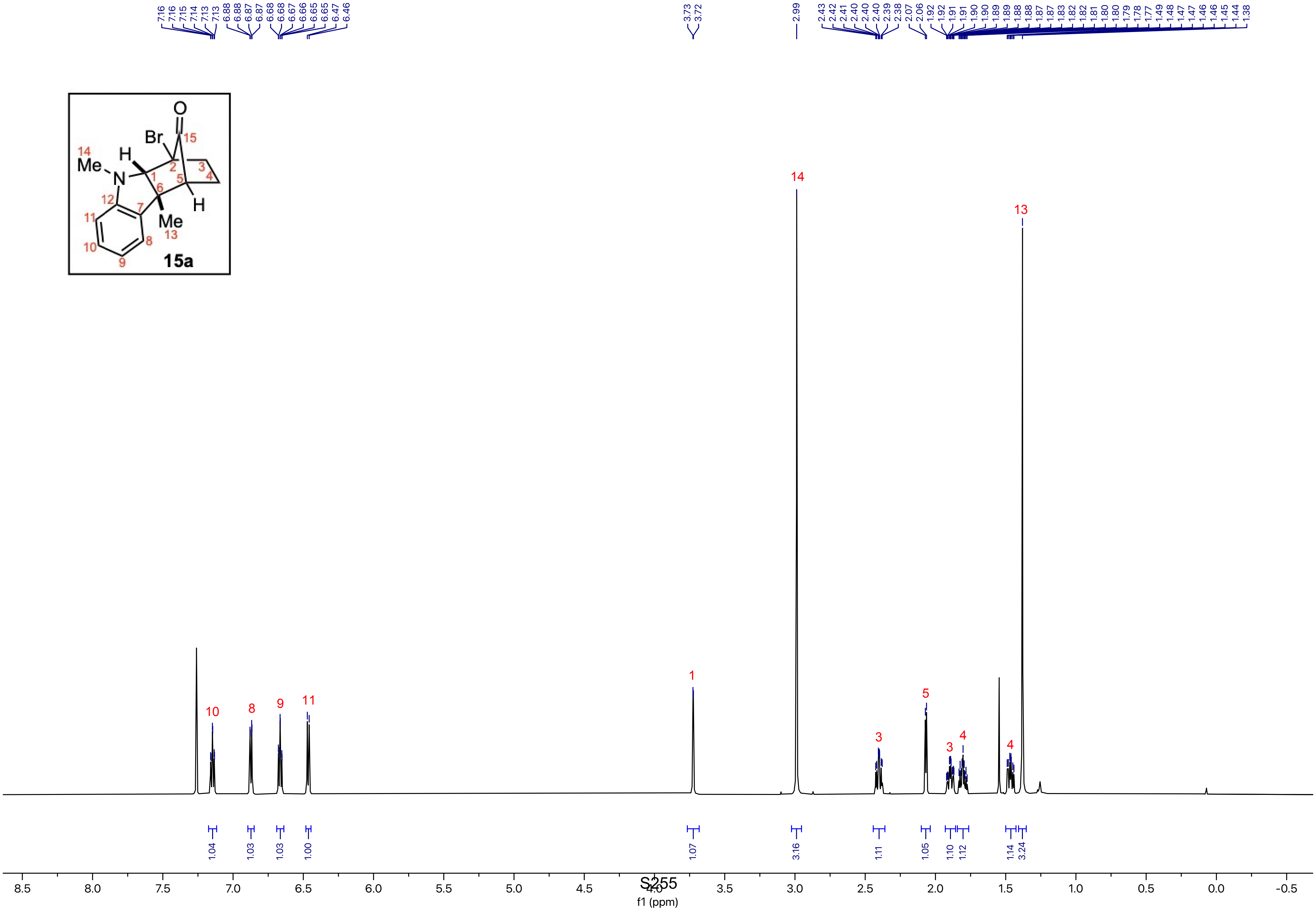
7.16
7.16
7.15
7.14
7.13
7.13
6.88
6.88
6.87
6.87
6.68
6.68
6.67
6.66
6.65
6.65
6.47
6.46
3.73
3.72
2.99
2.43
2.42
2.41
2.40
2.40
2.40
2.39
2.38
2.07
2.06
1.92
1.92
1.91
1.91
1.90
1.90
1.89
1.89
1.88
1.87
1.87
1.83
1.82
1.82
1.81
1.80
1.80
1.79
1.78
1.77
1.49
1.48
1.47
1.47
1.46
1.46
1.45
1.44
1.38
O
Br
15
14
Me
H
N
2
3
4
1
5
6
12
7
H
11
Me
13
10
8
9
15a
14
13
1
10
8
9
11
5
3
3
4
4
1.04
1.03
1.03
1.00
1.07
3.16
1.11
1.05
1.10
1.12
1.14
3.24
8.5
8.0
7.5
7.0
6.5
6.0
5.5
5.0
4.5
4.0
3.5
3.0
2.5
2.0
1.5
1.0
0.5
0.0
-0.5
f1 (ppm)

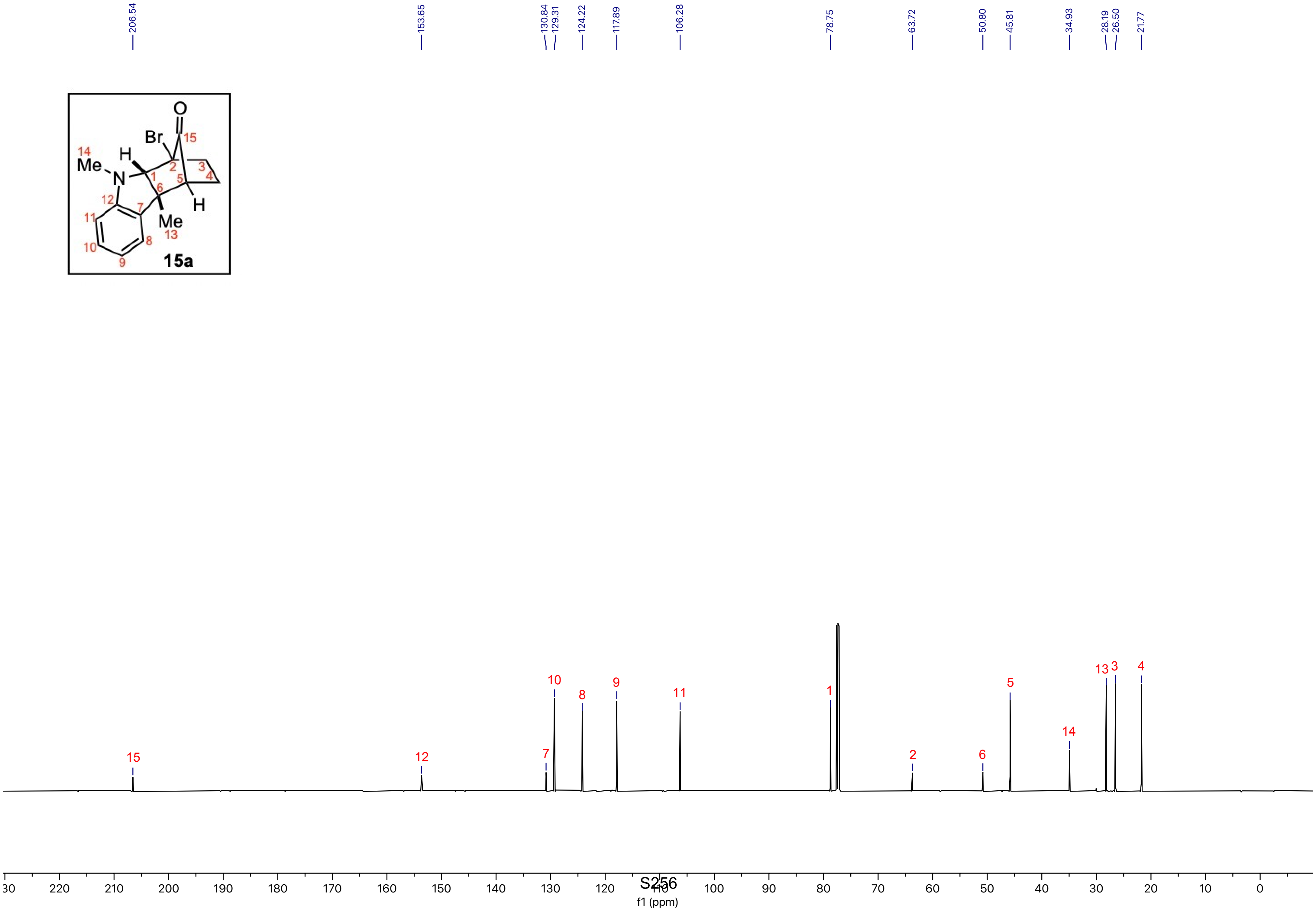
206.54
153.65
130.84
129.31
124.22
117.89
106.28
78.75
63.72
50.80
45.81
34.93
28.19
26.50
21.77
15a
Br
H
Me
N
O
15
12
7
10
8
9
11
1
2
6
5
14
13
3
4
f1 (ppm)
230
220
210
200
190
180
170
160
150
140
130
120
110
100
90
80
70
60
50
40
30
20
10
0

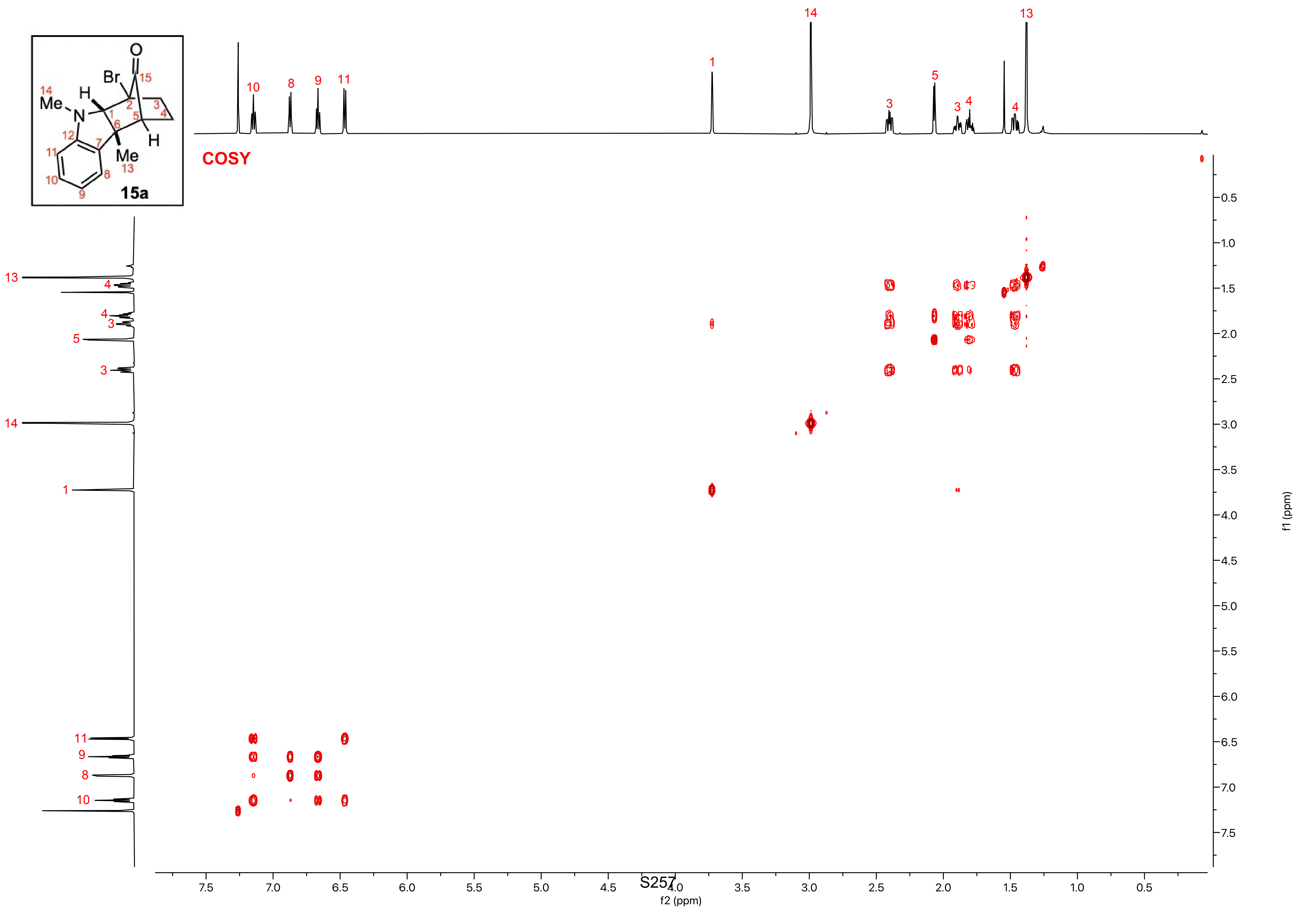

COSY
15a
f2 (ppm)
f1 (ppm)

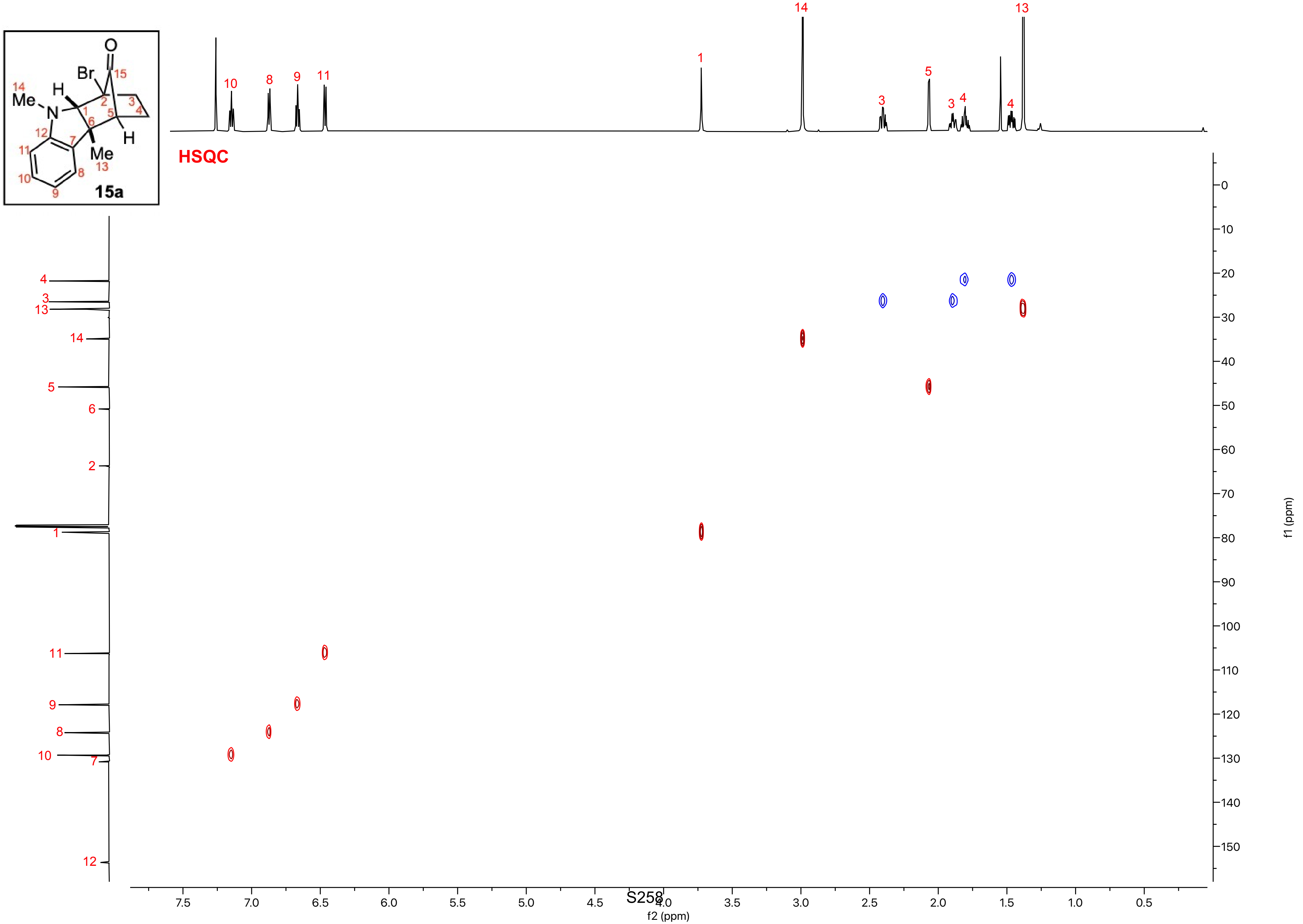
15a
HSQC
f2 (ppm)
f1 (ppm)

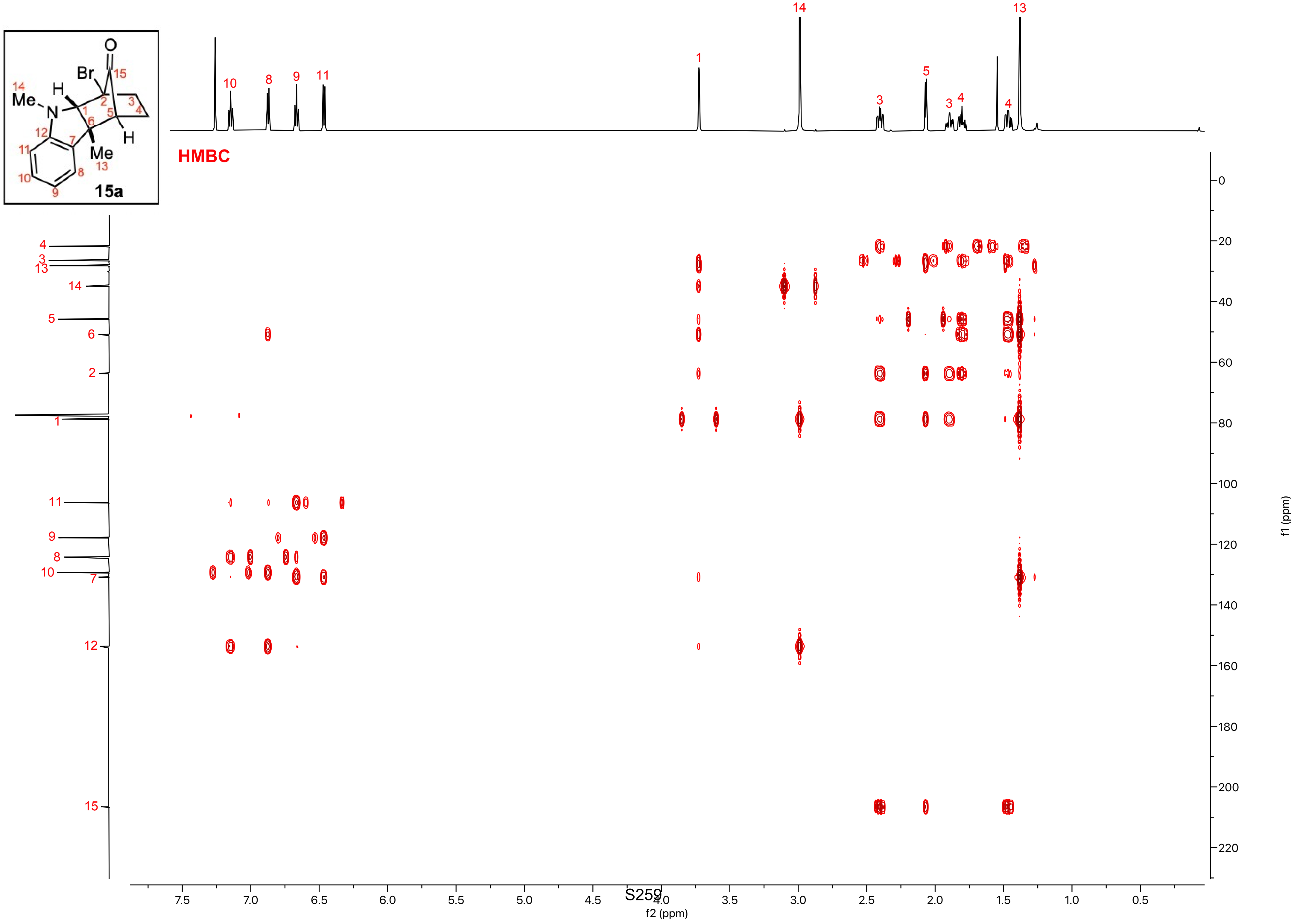

14
Me
N
H
Br
O
15
2
3
4
5
H
1
6
12
7
11
10
9
8
Me
13
15a
HMBC
f2 (ppm)
f1 (ppm)

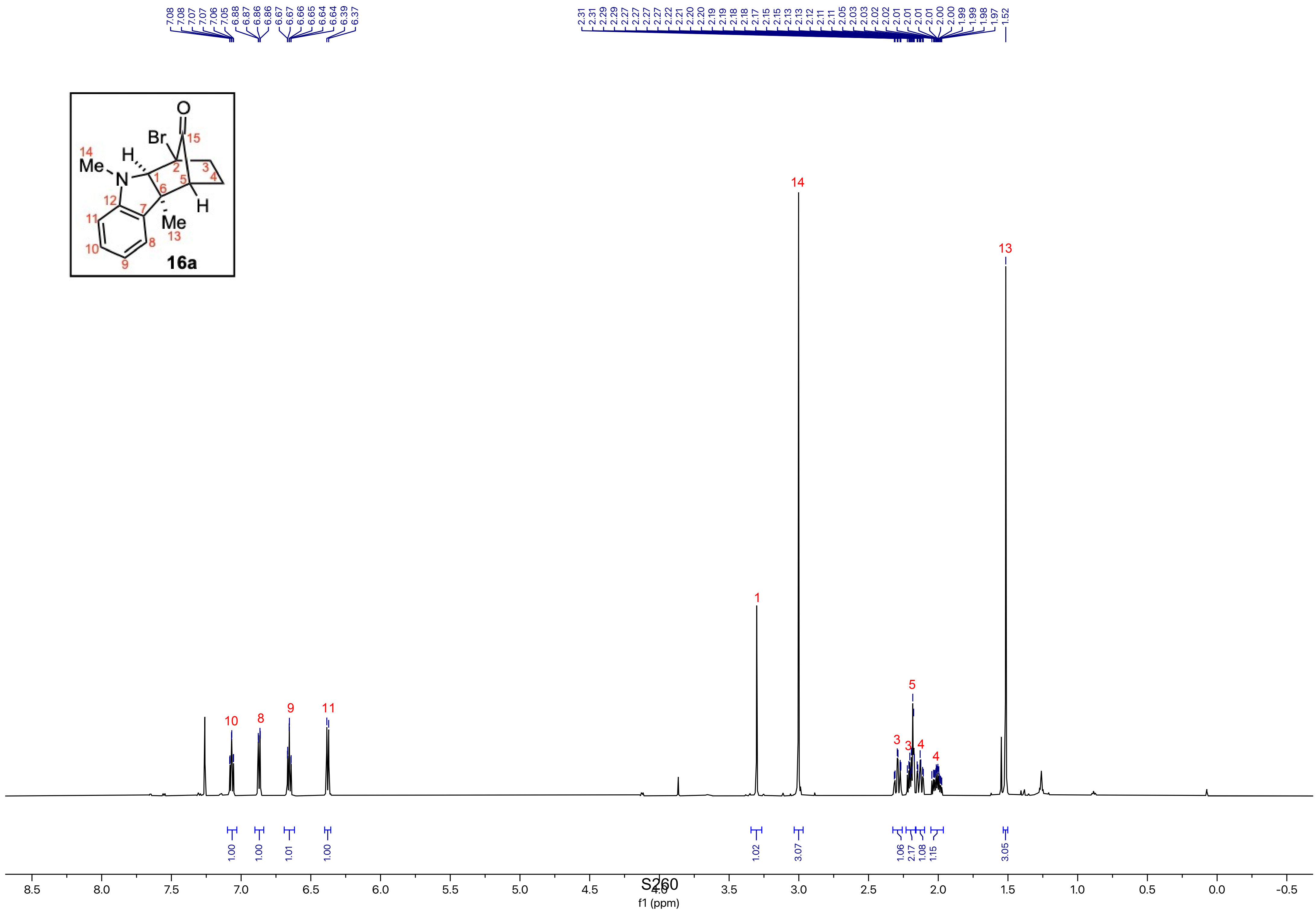
7.08 7.08 7.07 7.07 7.06 7.05 6.88 6.87 6.86 6.86 6.67 6.67 6.66 6.65 6.64 6.64 6.39 6.37
2.31 2.31 2.29 2.29 2.27 2.27 2.27 2.22 2.21 2.20 2.20 2.19 2.19 2.18 2.18 2.17 2.15 2.15 2.13 2.13 2.12 2.11 2.11 2.05 2.03 2.02 2.02 2.01 2.01 2.01 2.01 2.00 2.00 1.99 1.99 1.98 1.97 1.52
O
Br
15
14
Me
H
N
2
3
4
1
6
5
12
H
7
11
Me
13
10
8
9
16a
14
13
1
5
3
3
4
4
10
8
9
11
1.00
1.00
1.01
1.00
1.02
3.07
1.06
2.17
1.08
1.15
3.05
8.5 8.0 7.5 7.0 6.5 6.0 5.5 5.0 4.5 4.0 3.5 3.0 2.5 2.0 1.5 1.0 0.5 0.0 -0.5
f1 (ppm)

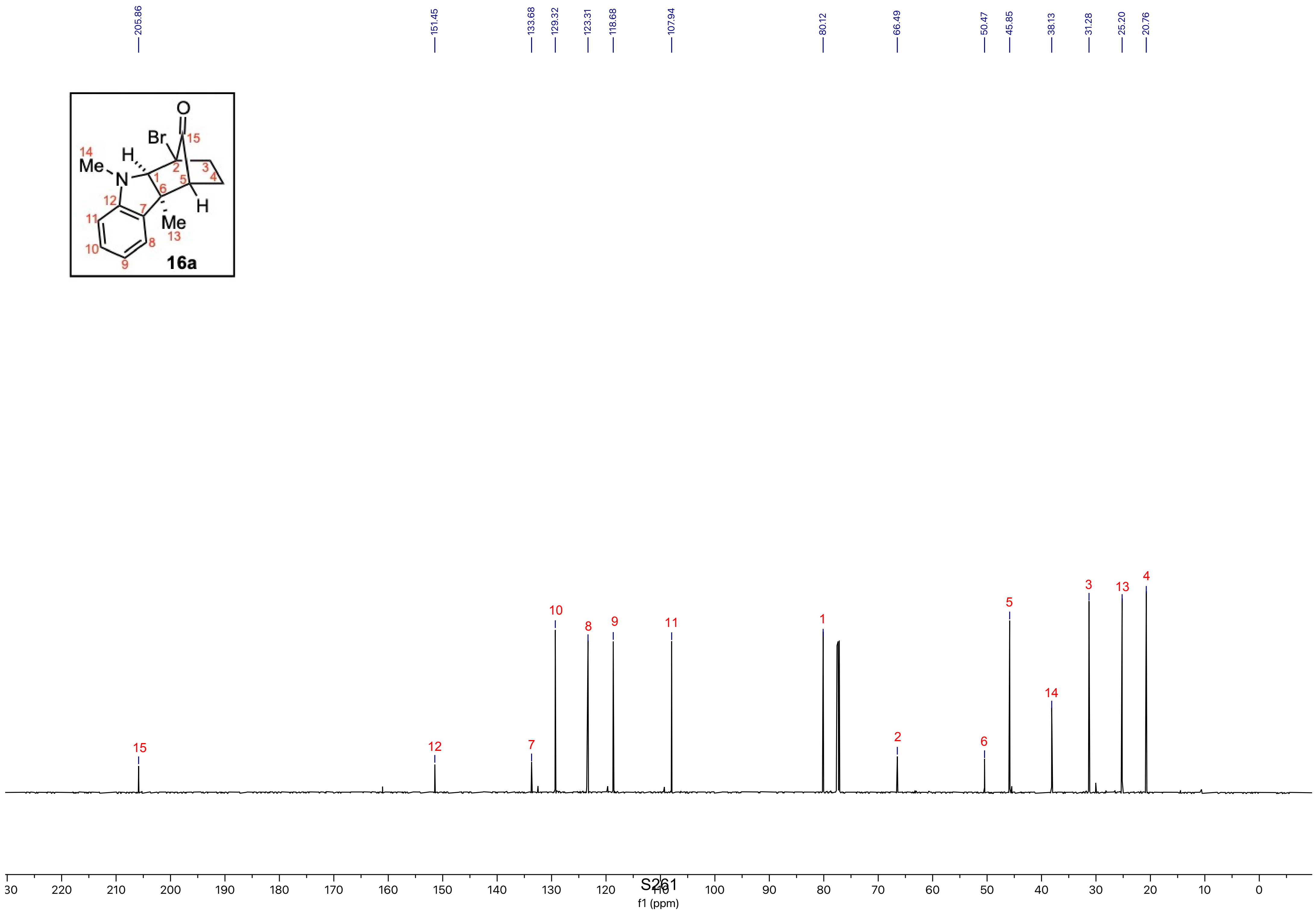
205.86
151.45
133.68
129.32
123.31
118.68
107.94
80.12
66.49
50.47
45.85
38.13
31.28
25.20
20.76
Br
Me
N
H
O
Me
H
16a
15
12
7
10
8
9
11
1
2
6
5
14
3
13
4
f1 (ppm)

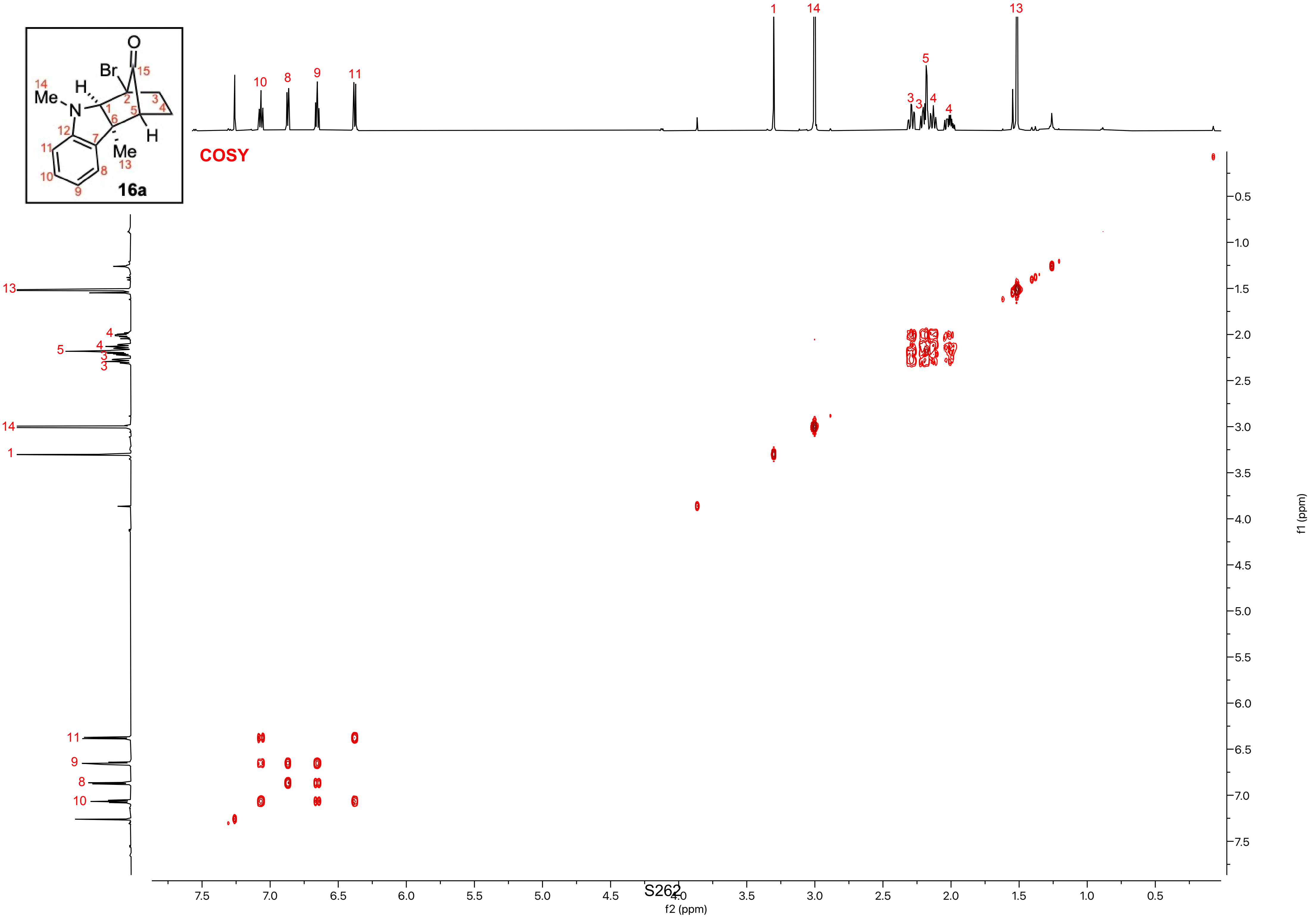
16a
COSY
f2 (ppm)
f1 (ppm)

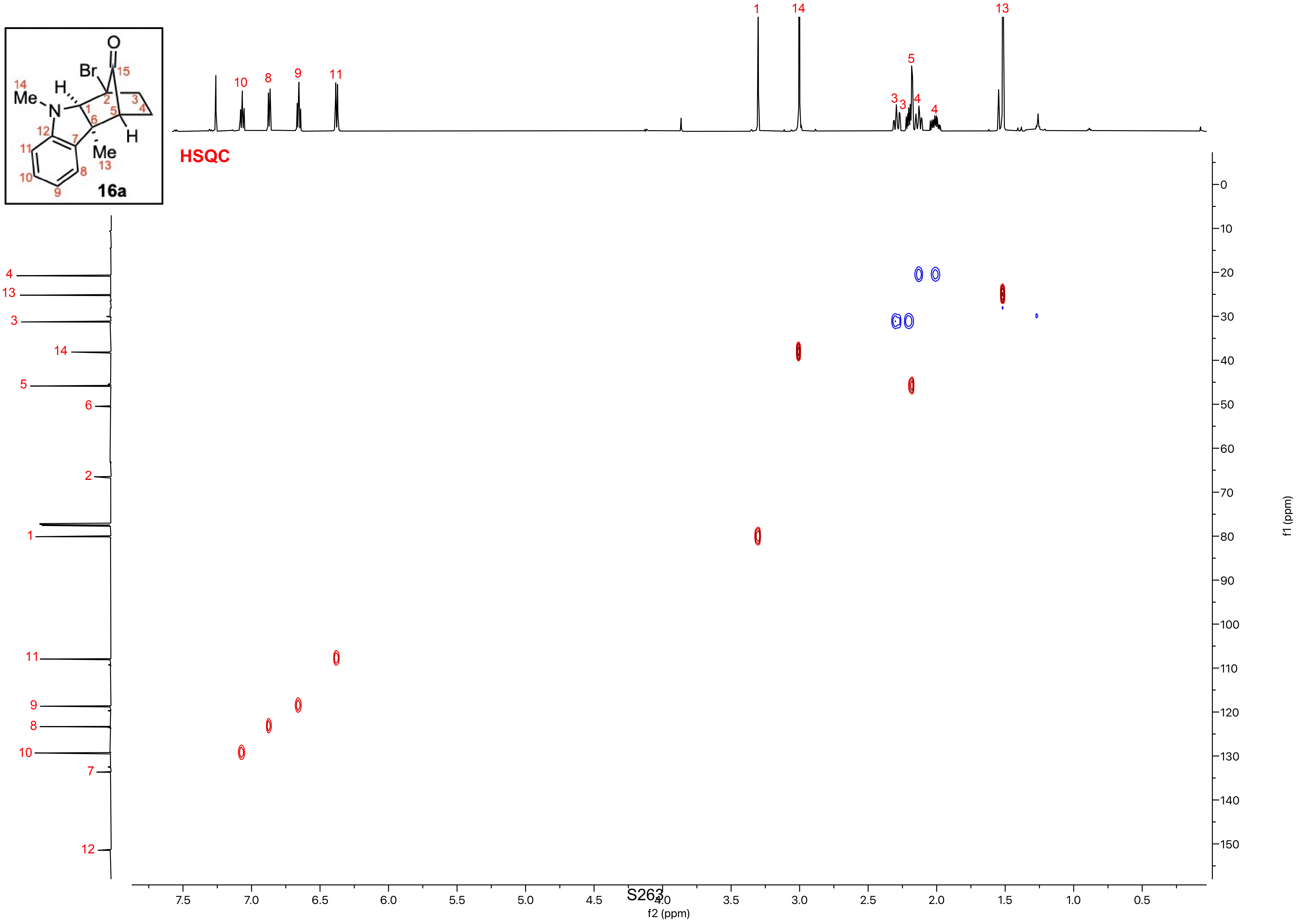

16a
HSQC
f2 (ppm)
f1 (ppm)

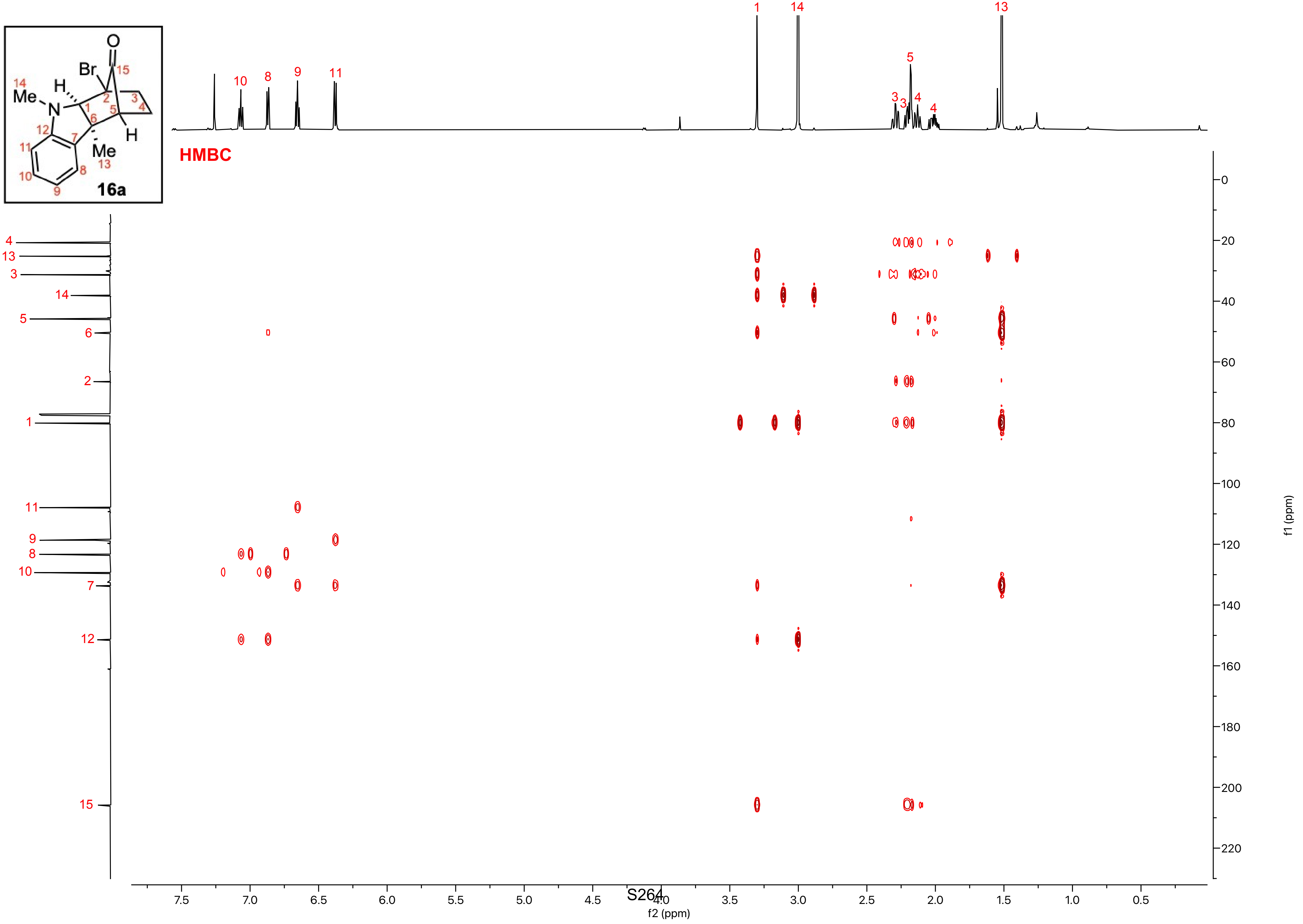
16a
HMBC
f2 (ppm)
f1 (ppm)

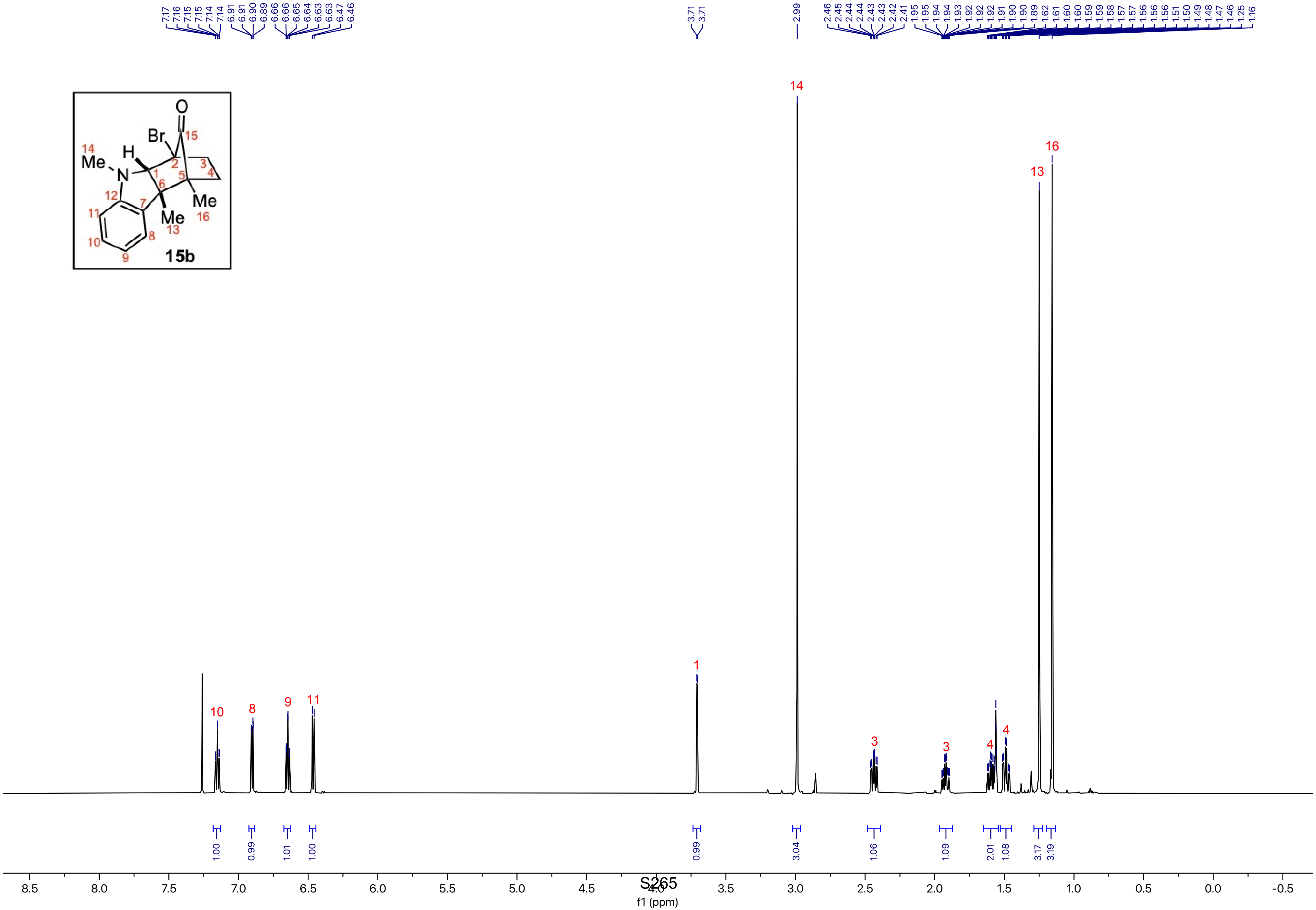
15b
f1 (ppm)

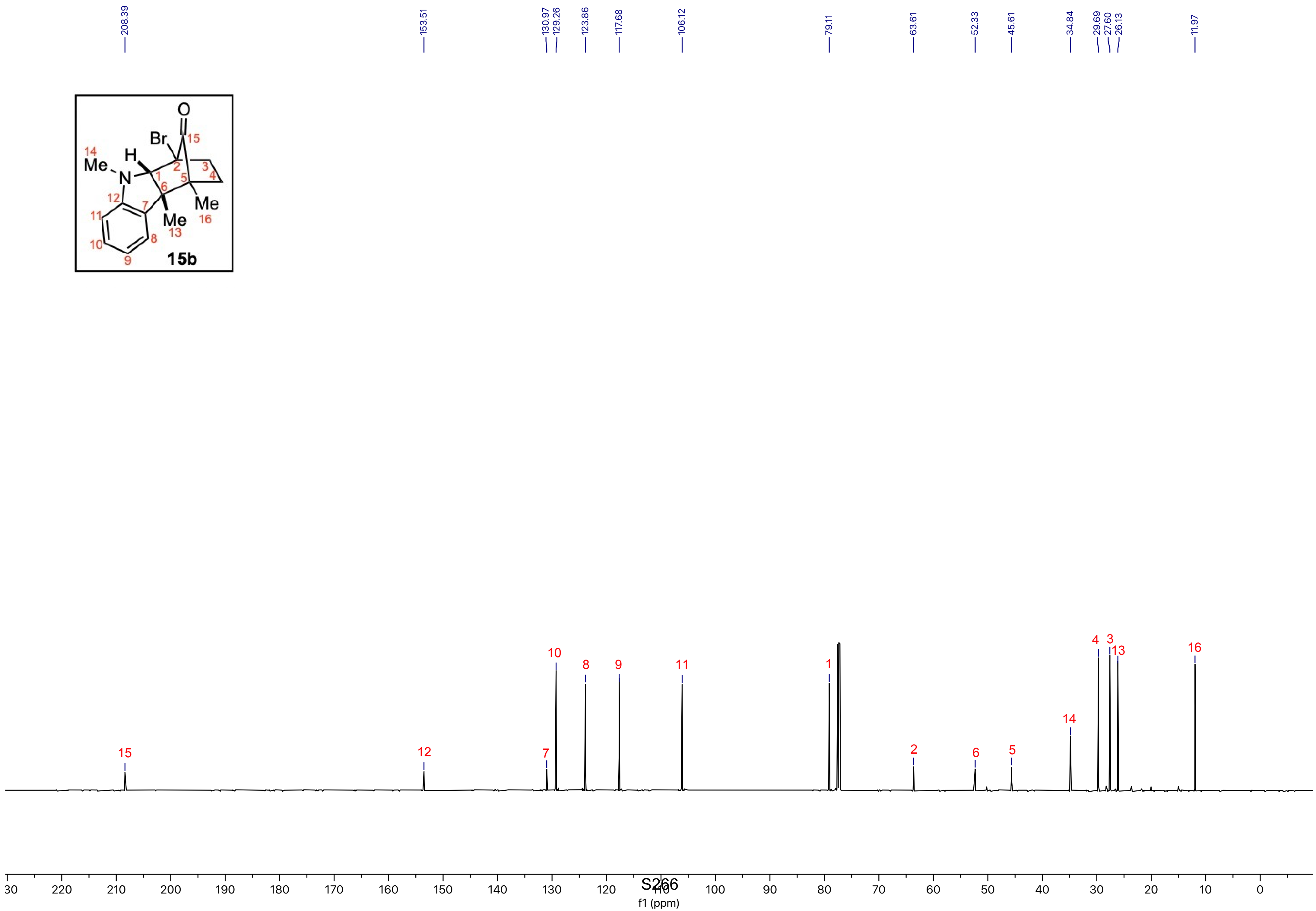
208.39
153.51
130.97
129.26
123.86
117.68
106.12
79.11
63.61
52.33
45.61
34.84
29.69
27.60
26.13
11.97
O
Br
15
14
Me
H
N
1
2
3
4
5
6
7
8
9
10
11
12
Me
16
Me
13
15b
f1 (ppm)

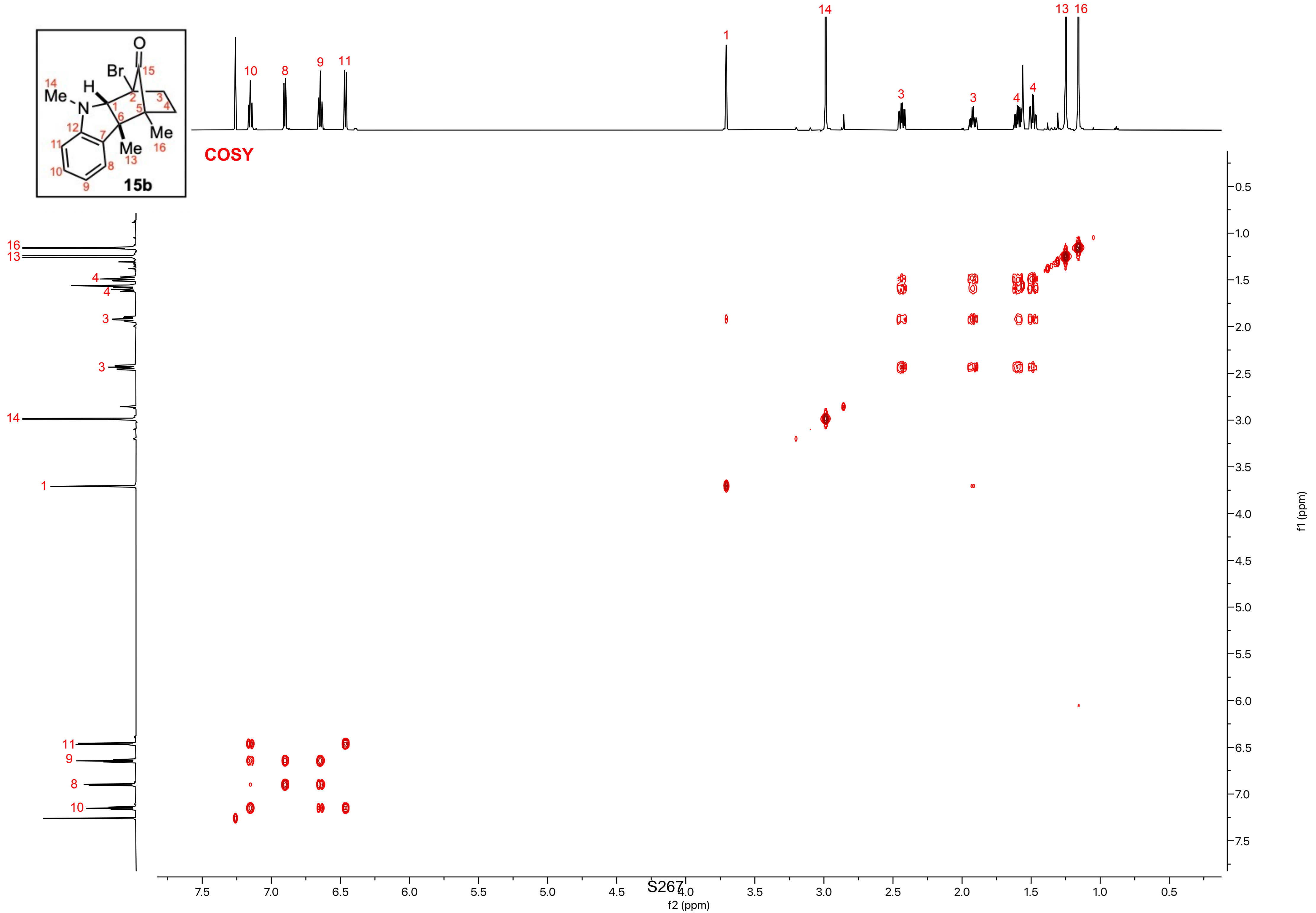
15b
Br
Me
N
H
O
Me
Me
COSY
f2 (ppm)
f1 (ppm)

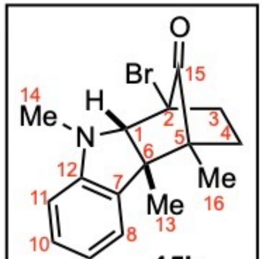

15b

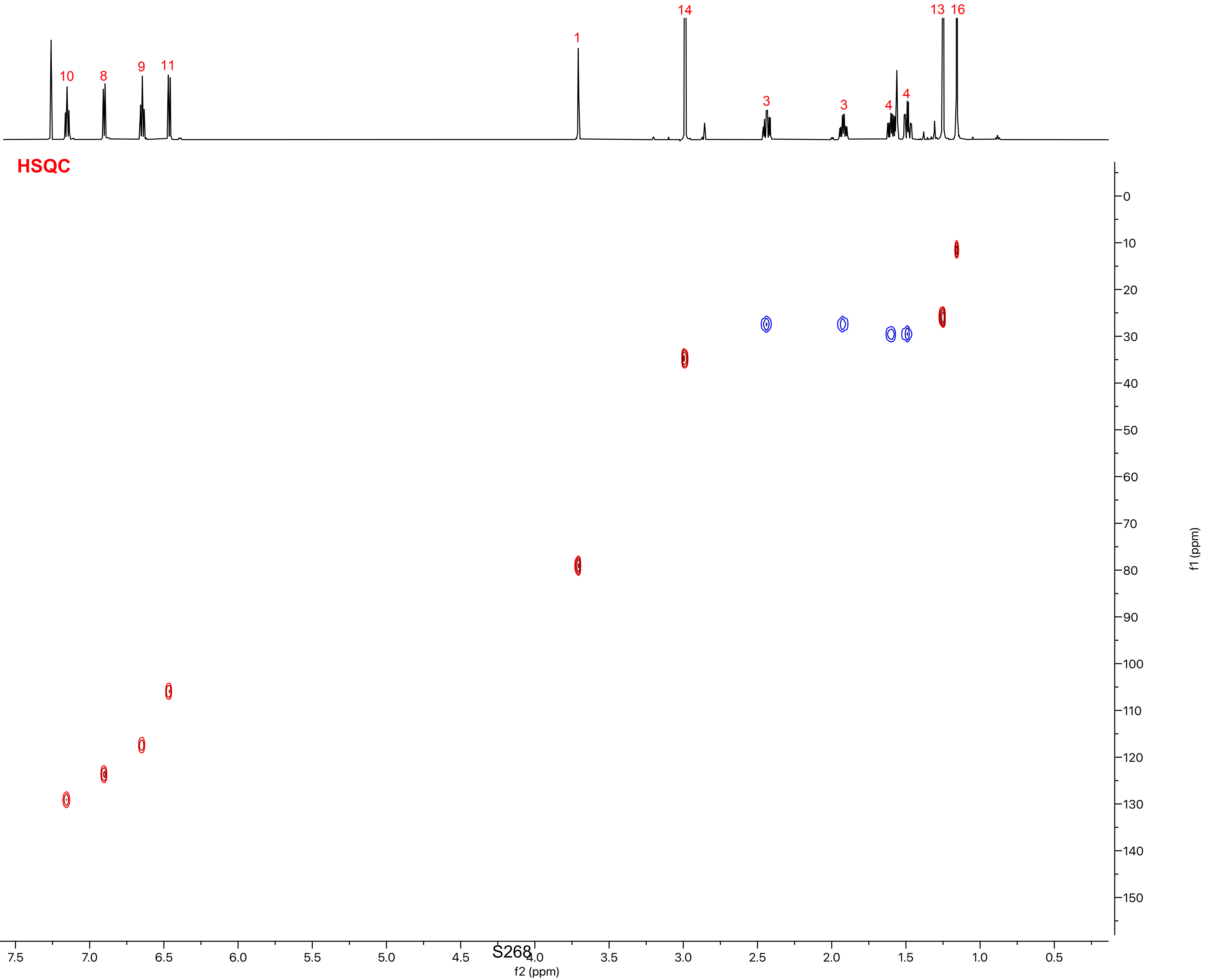

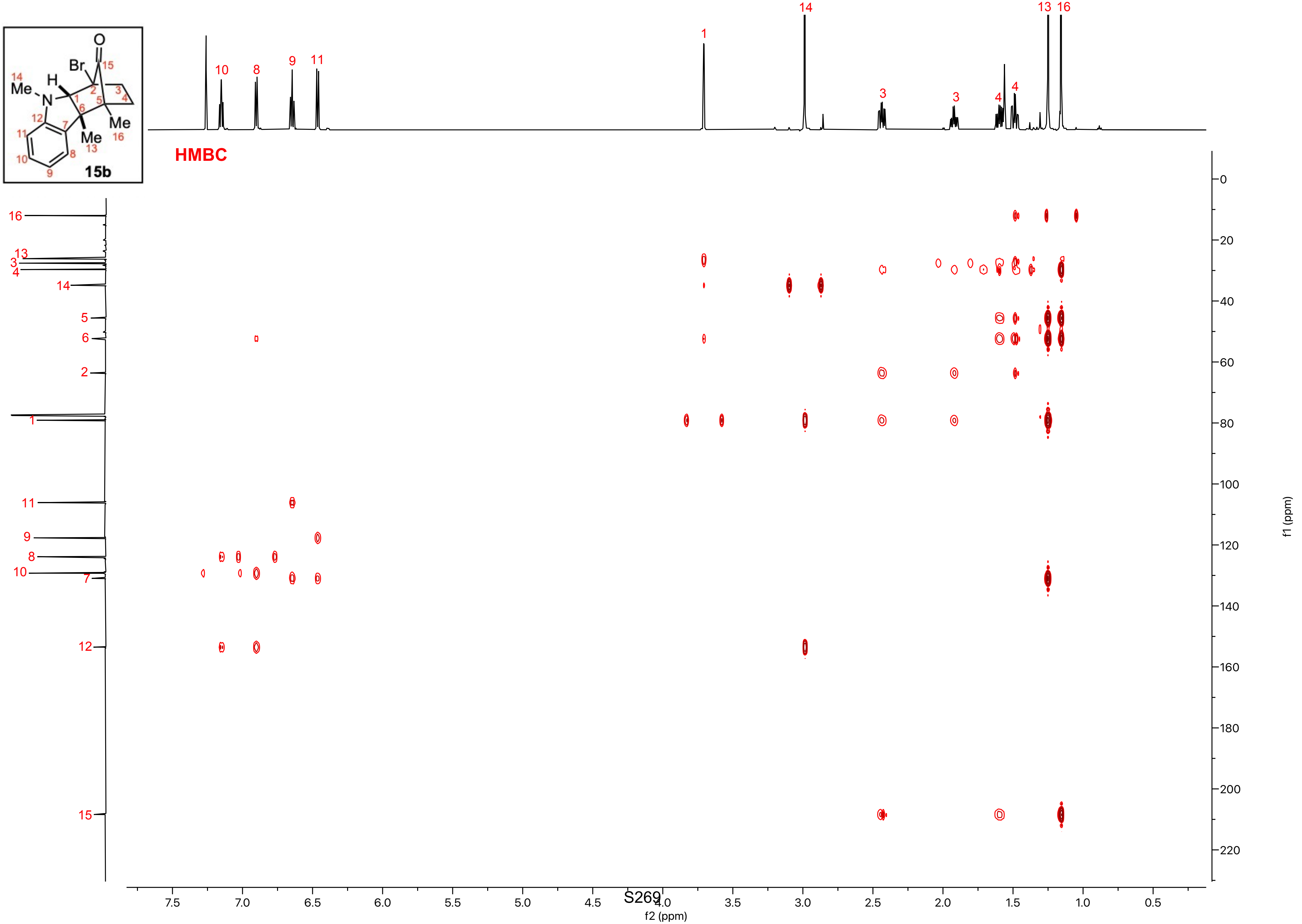
HMBC
15b
f2 (ppm)
f1 (ppm)

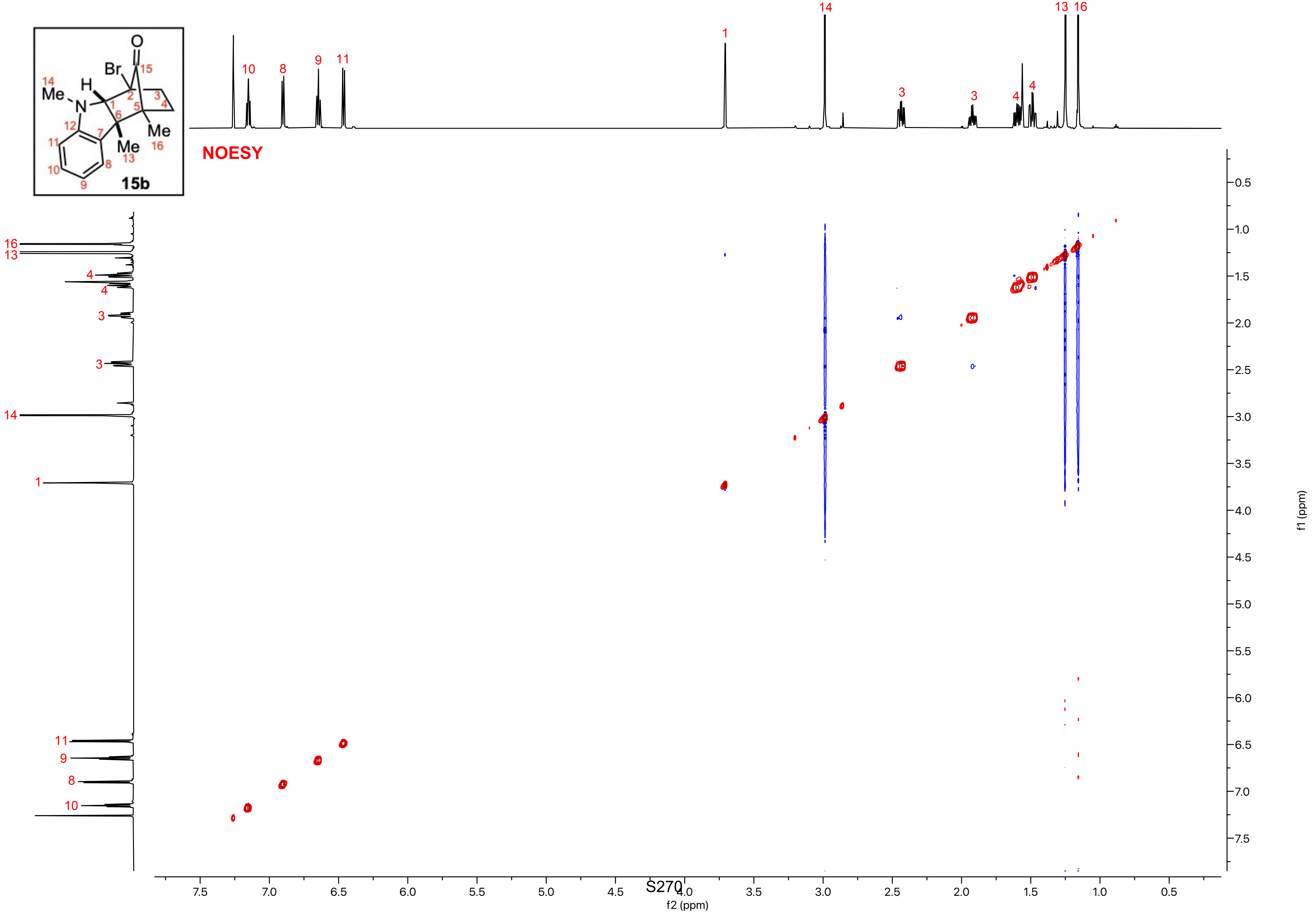

15b
Br
Me
N
H
O
Me
Me
NOESY
f1 (ppm)
f2 (ppm)

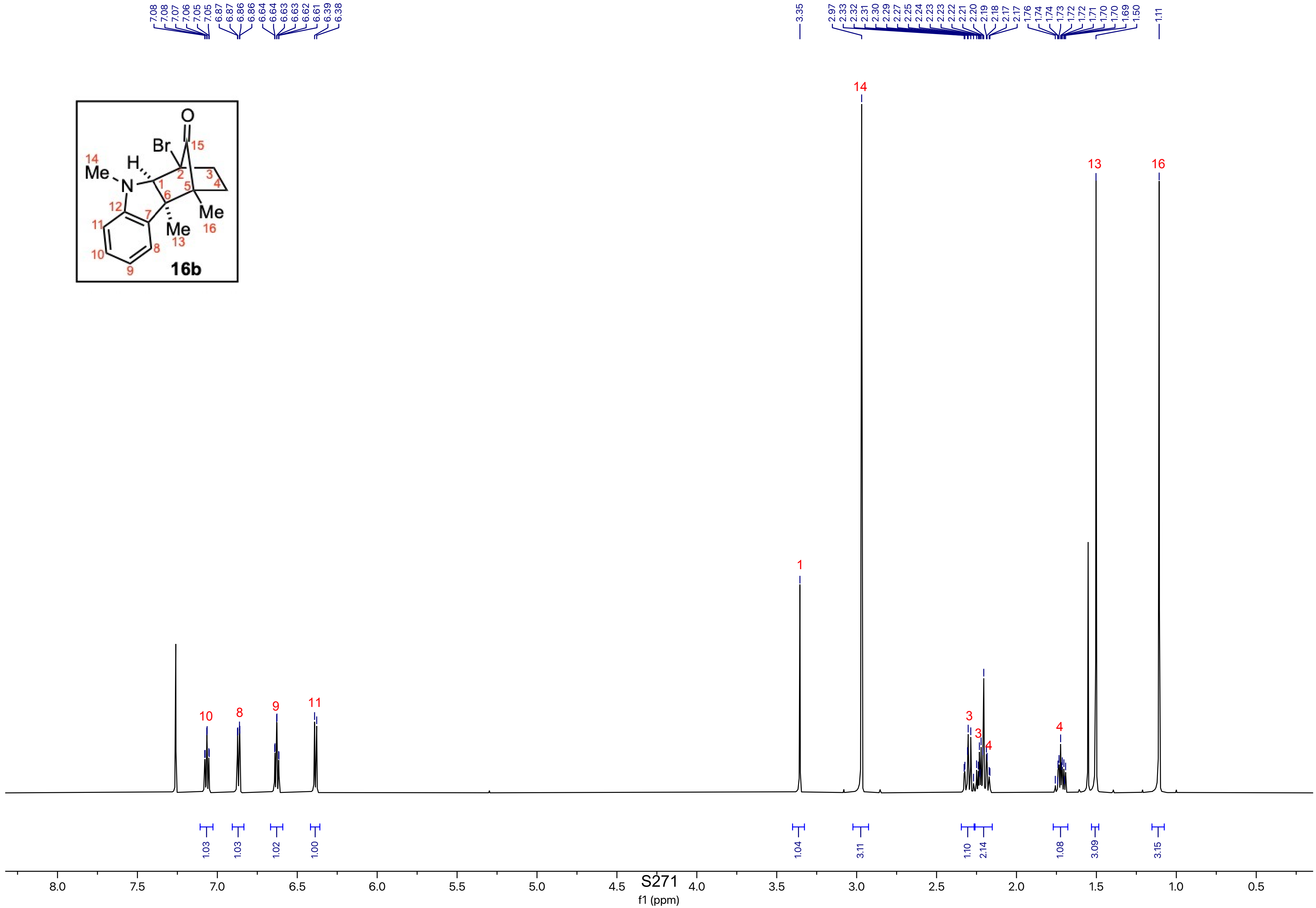

7.08 7.08 7.07 7.06 7.05 7.05 6.87 6.87 6.86 6.86 6.64 6.63 6.63 6.62 6.61 6.39 6.38
3.35
2.97 2.33 2.32 2.31 2.30 2.29 2.27 2.25 2.24 2.23 2.23 2.22 2.21 2.20 2.19 2.18 2.17 2.17 1.76 1.74 1.73 1.72 1.72 1.71 1.70 1.70 1.69 1.50
1.11
16b
1.03 1.03 1.02 1.00 1.04 3.11 1.10 2.14 1.08 3.09 3.15
f1 (ppm)

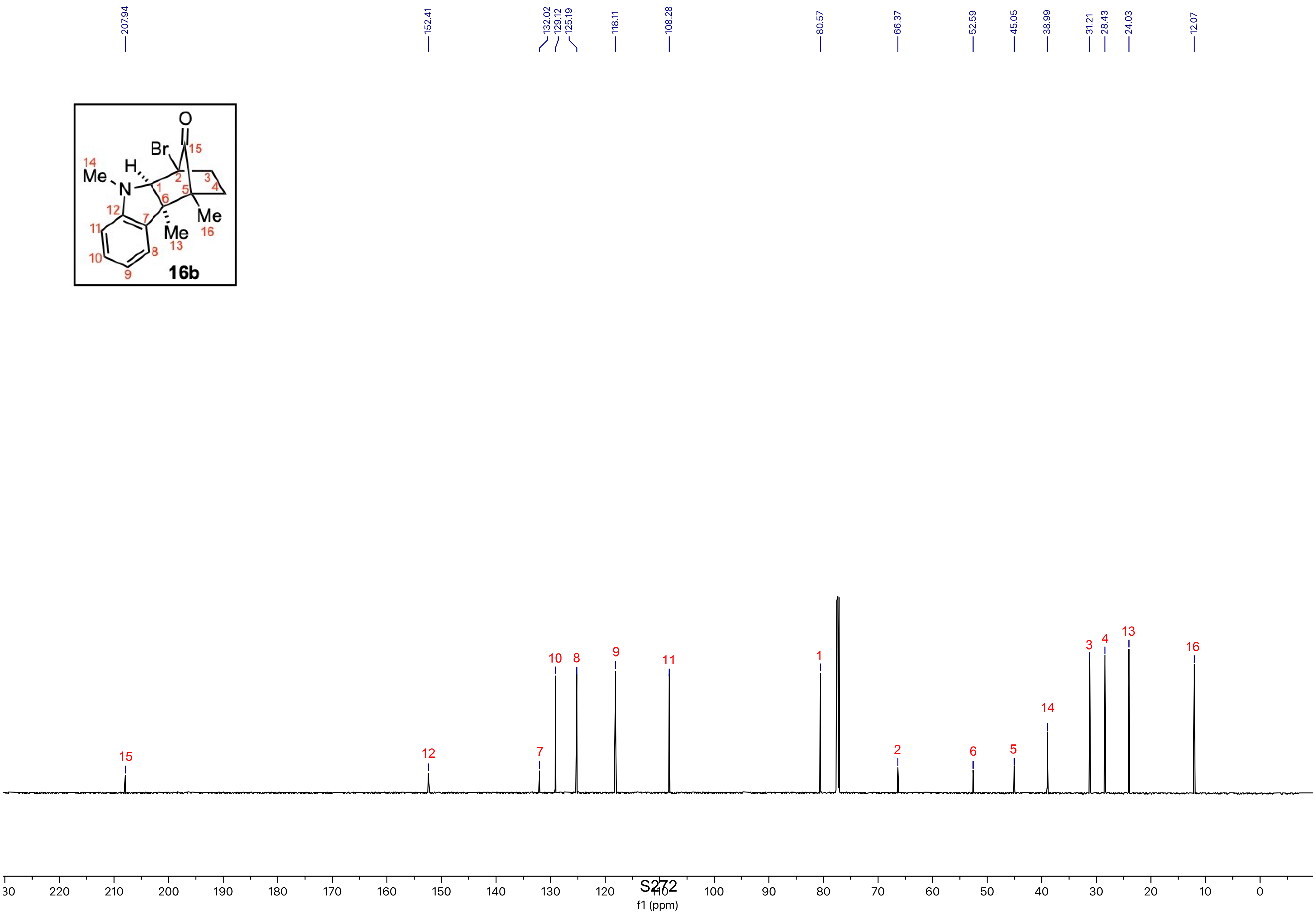

207.94
152.41
132.02
129.12
125.19
118.11
108.28
80.57
66.37
52.59
45.05
38.99
31.21
28.43
24.03
12.07
Br
O
H
Me
N
Me
Me
16b
f1 (ppm)

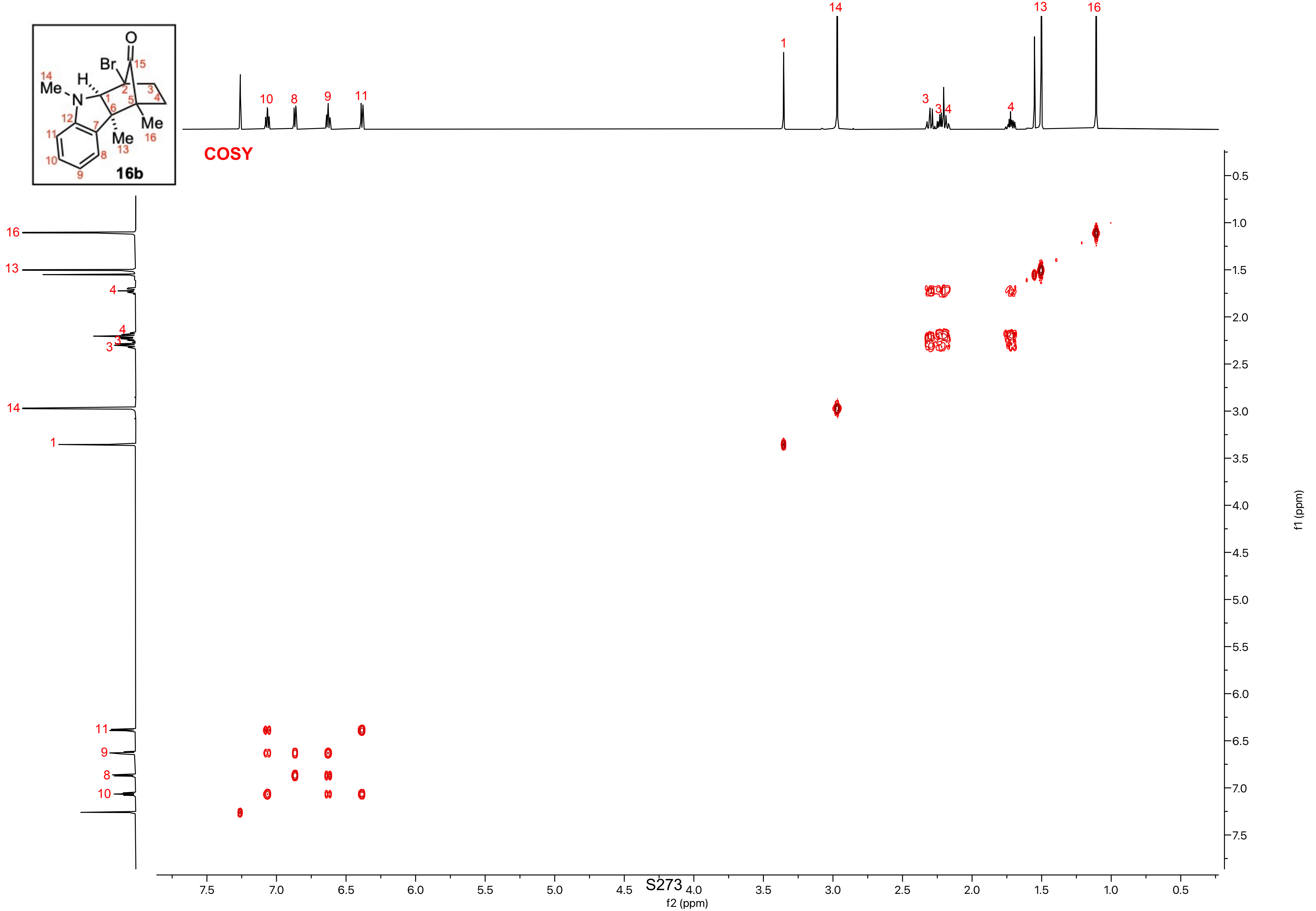
COSY
16b
Br
O
H
N
Me
Me
Me
14
15
1
2
3
4
5
6
7
8
9
10
11
12
13
16
7.5
7.0
6.5
6.0
5.5
5.0
4.5
4.0
3.5
3.0
2.5
2.0
1.5
1.0
0.5
f2 (ppm)
f1 (ppm)

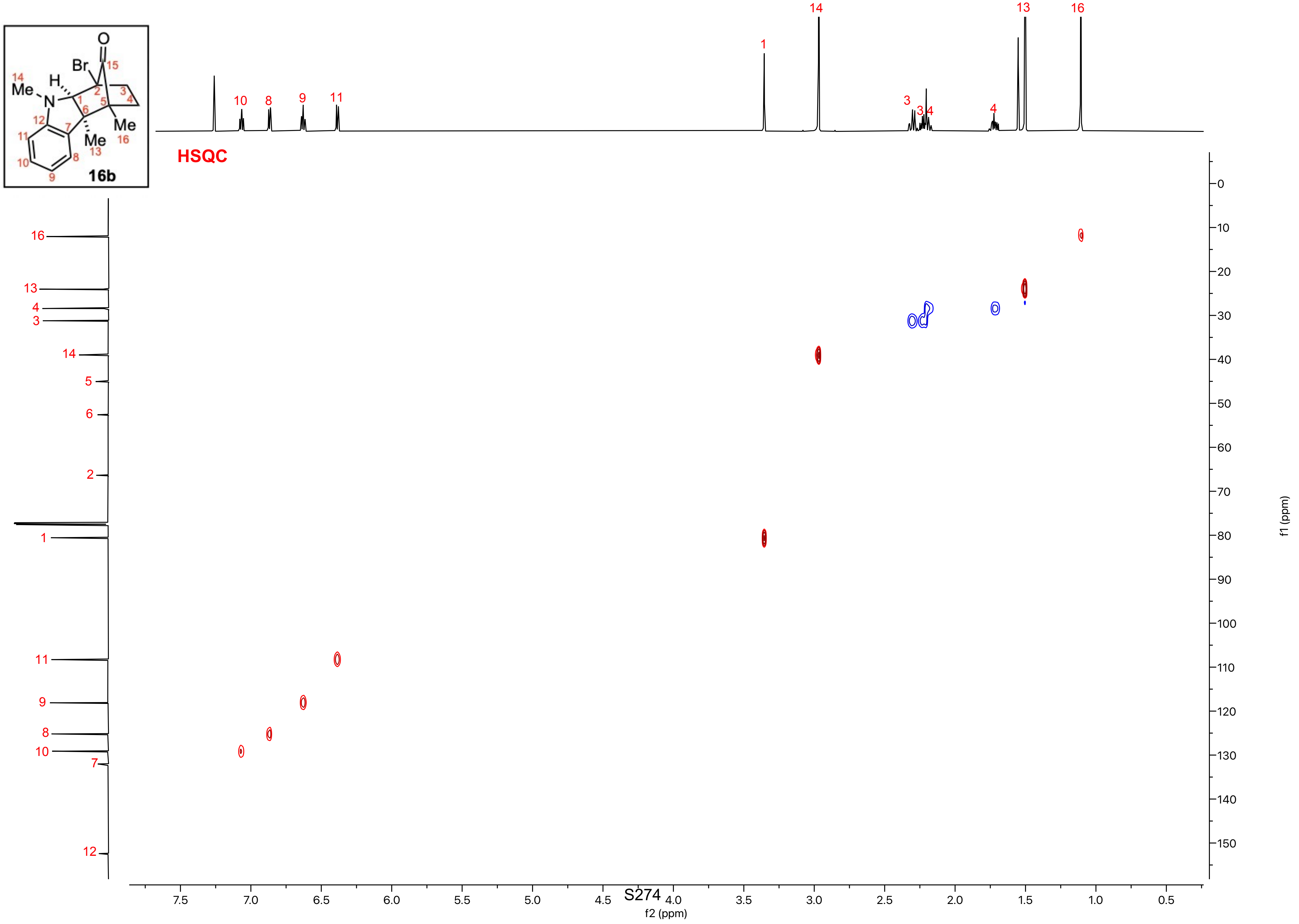
16b
HSQC
f2 (ppm)
f1 (ppm)

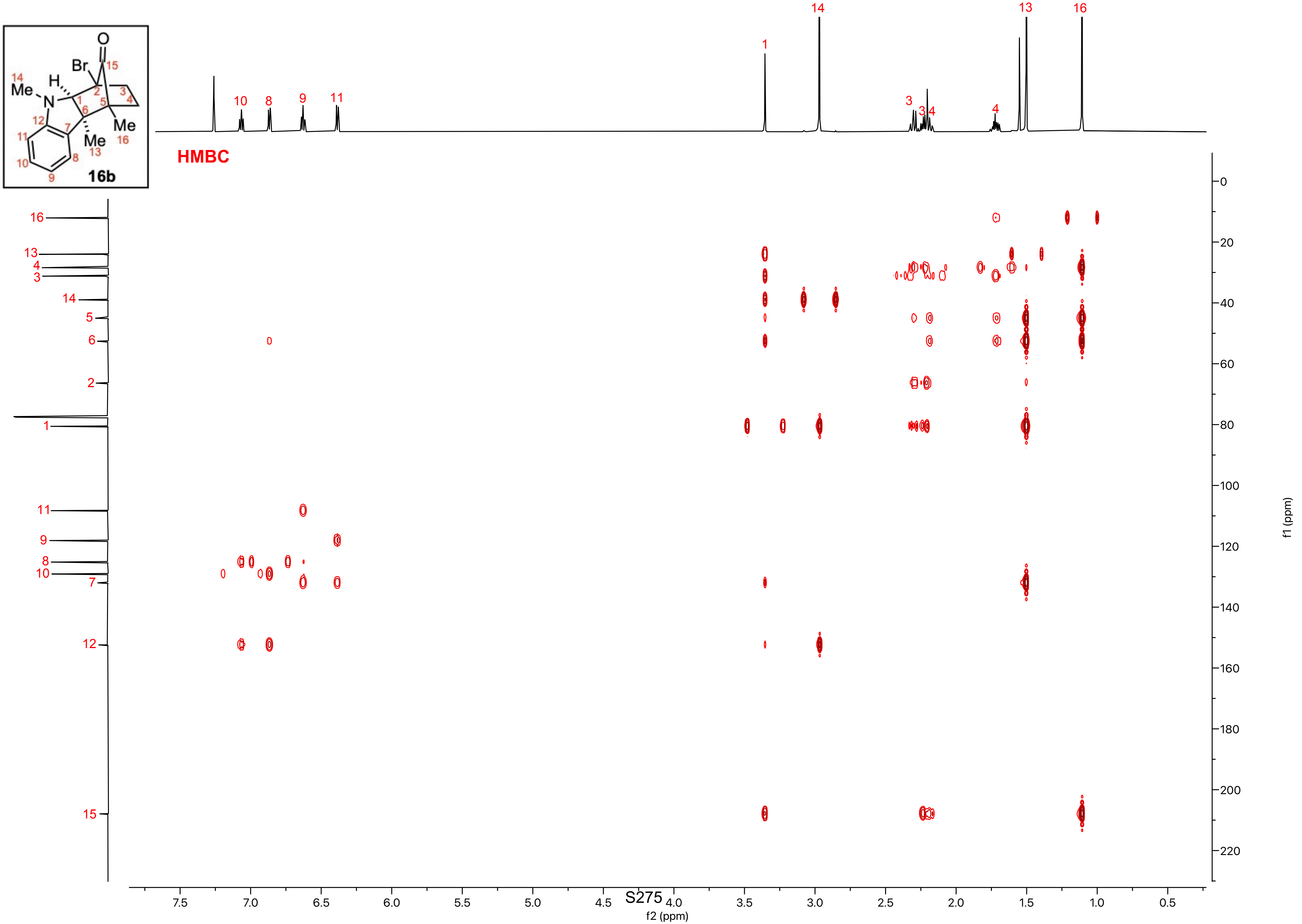

16b
HMBC
f2 (ppm)
f1 (ppm)

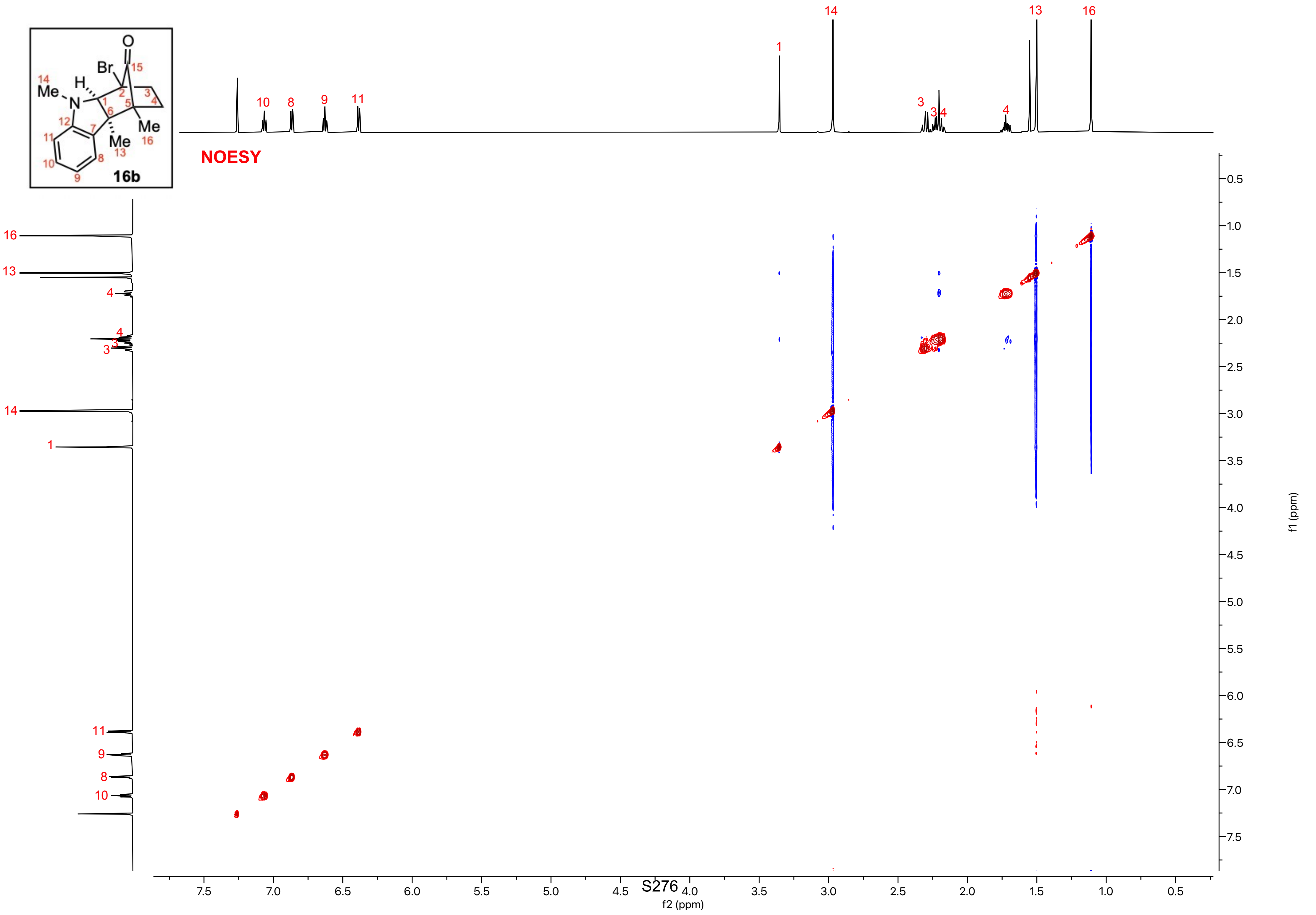
16b
NOESY
f2 (ppm)
f1 (ppm)

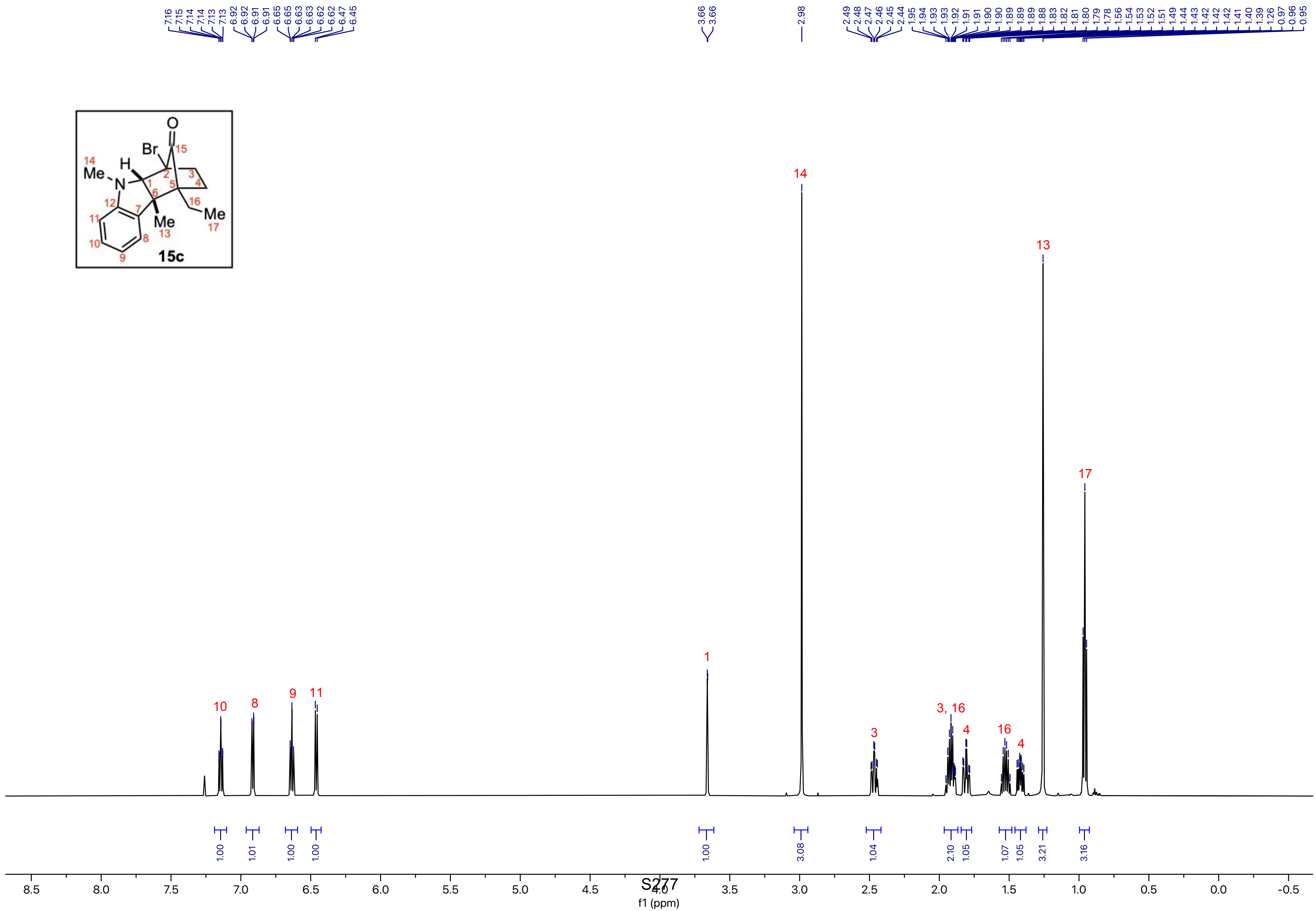
7.16
7.15
7.14
7.14
7.13
7.13
6.92
6.92
6.91
6.91
6.65
6.65
6.63
6.63
6.62
6.62
6.47
6.45
3.66
3.66
2.98
2.49
2.48
2.47
2.46
2.45
2.44
1.95
1.94
1.93
1.93
1.92
1.91
1.91
1.90
1.90
1.89
1.89
1.89
1.88
1.83
1.82
1.81
1.80
1.79
1.78
1.56
1.54
1.53
1.52
1.51
1.49
1.44
1.43
1.42
1.42
1.41
1.40
1.39
1.26
0.97
0.96
0.95
15c
14
13
1
10
8
9
11
3
3, 16
4
16
4
17
1.00
1.01
1.00
1.00
1.00
3.08
1.04
2.10
1.05
1.07
1.05
3.21
3.16
8.5
8.0
7.5
7.0
6.5
6.0
5.5
5.0
4.5
4.0
3.5
3.0
2.5
2.0
1.5
1.0
0.5
0.0
-0.5
f1 (ppm)

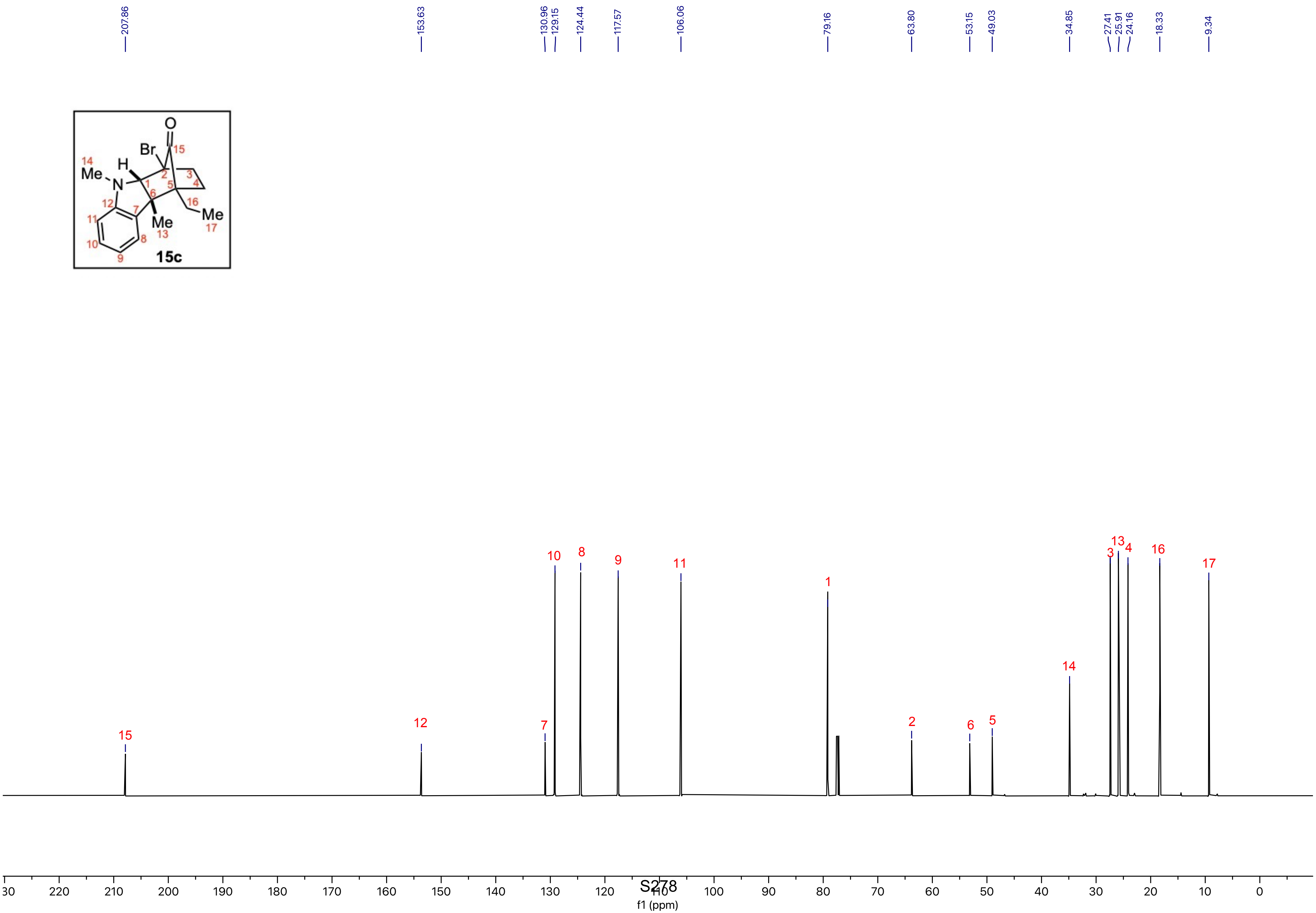
207.86
153.63
130.96
129.15
124.44
117.57
106.06
79.16
63.80
53.15
49.03
34.85
27.41
25.91
24.16
18.33
9.34
15c
f1 (ppm)

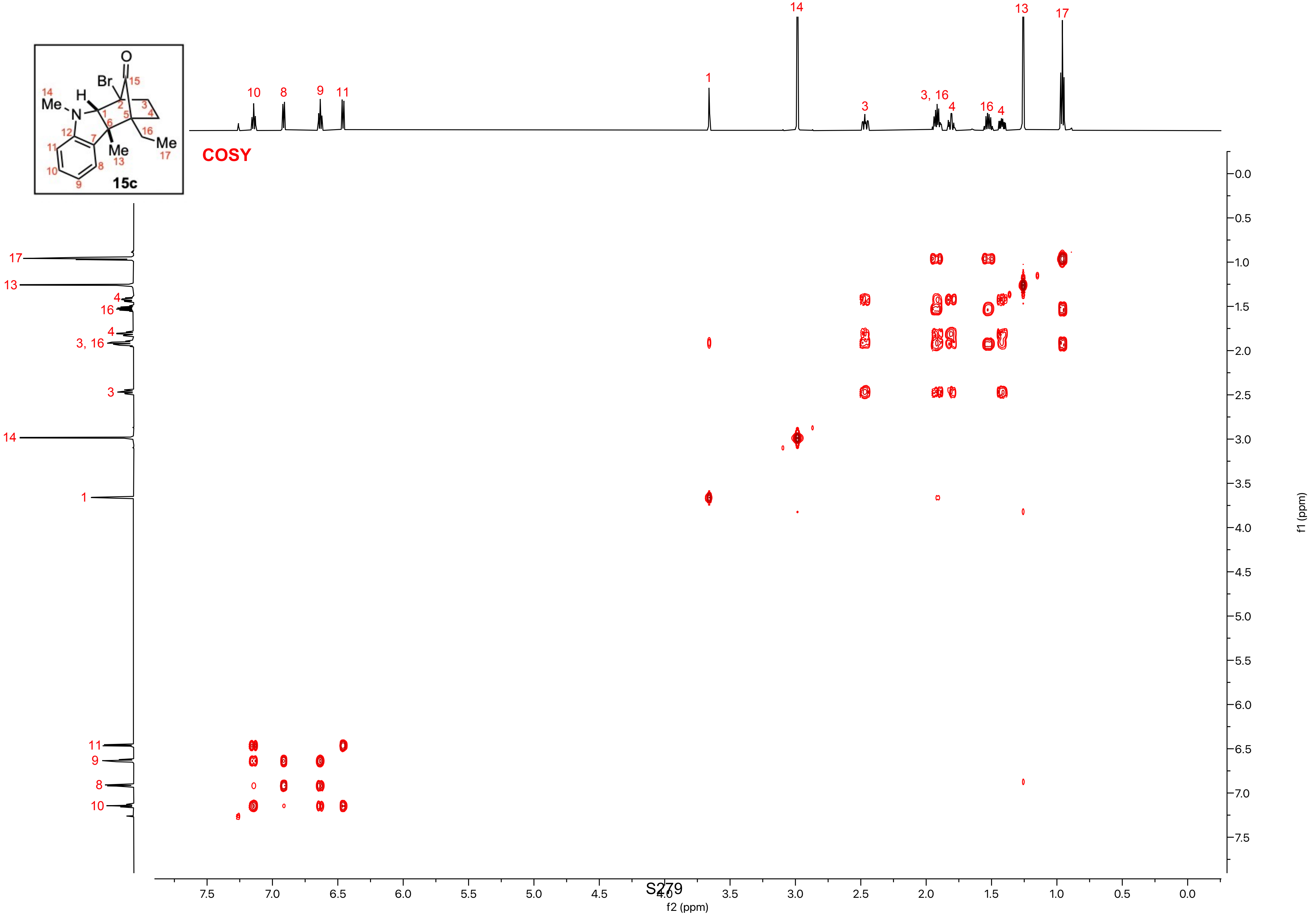

15c
COSY
14
13
17
10
8
9
11
1
3
3, 16
4
16
f2 (ppm)
f1 (ppm)

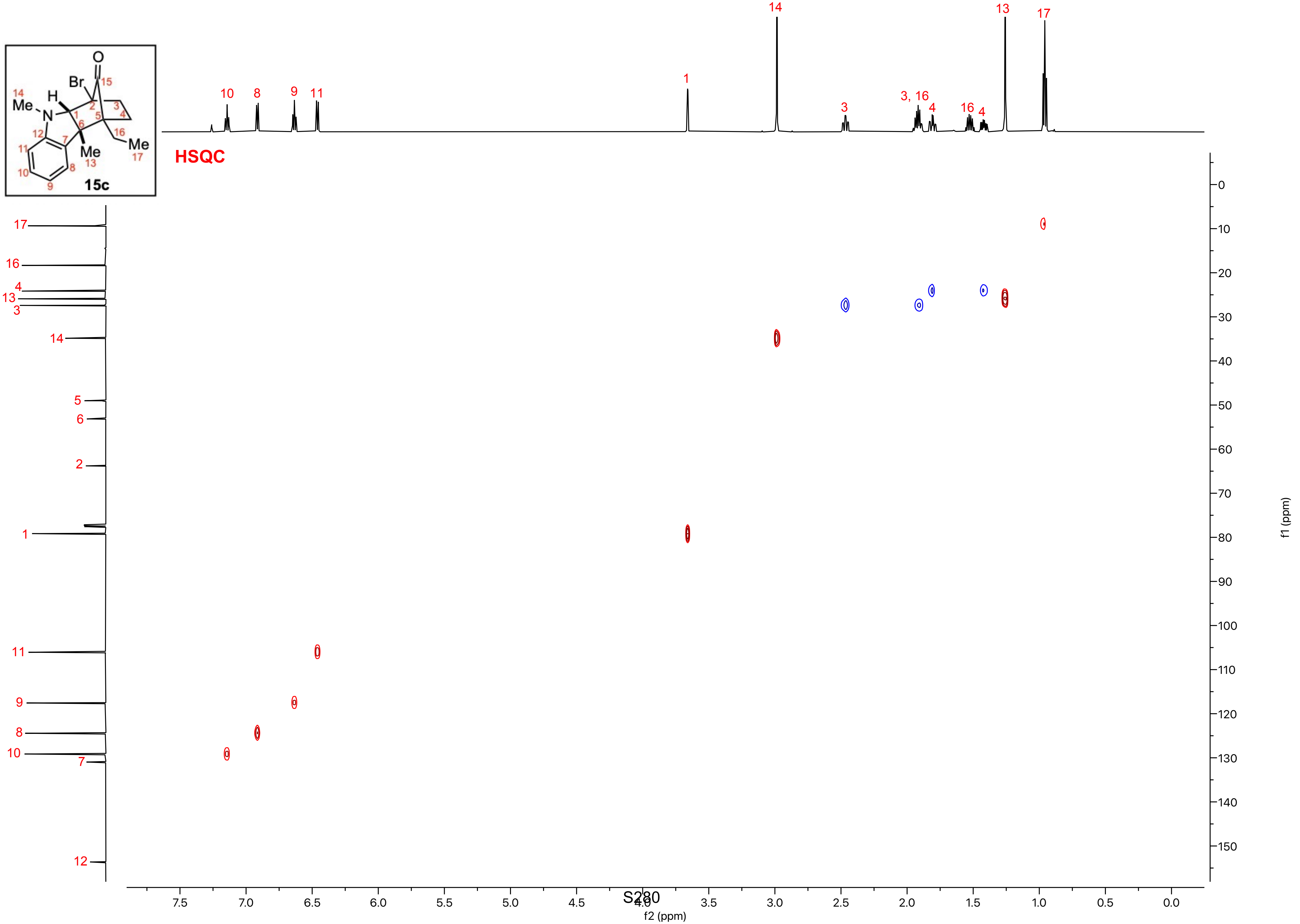

15c
HSQC
f2 (ppm)
f1 (ppm)

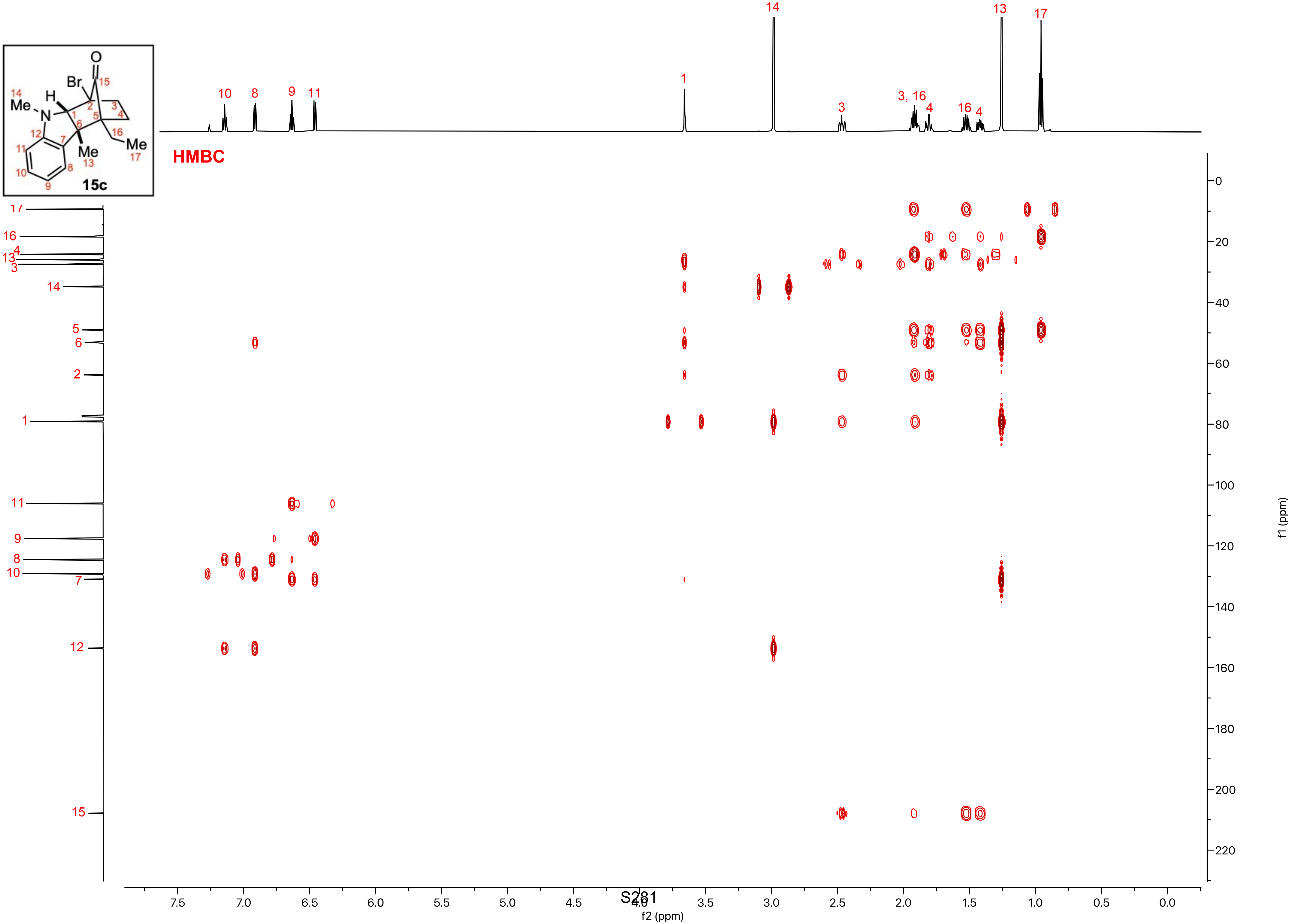

HMBC
15c
f2 (ppm)
f1 (ppm)

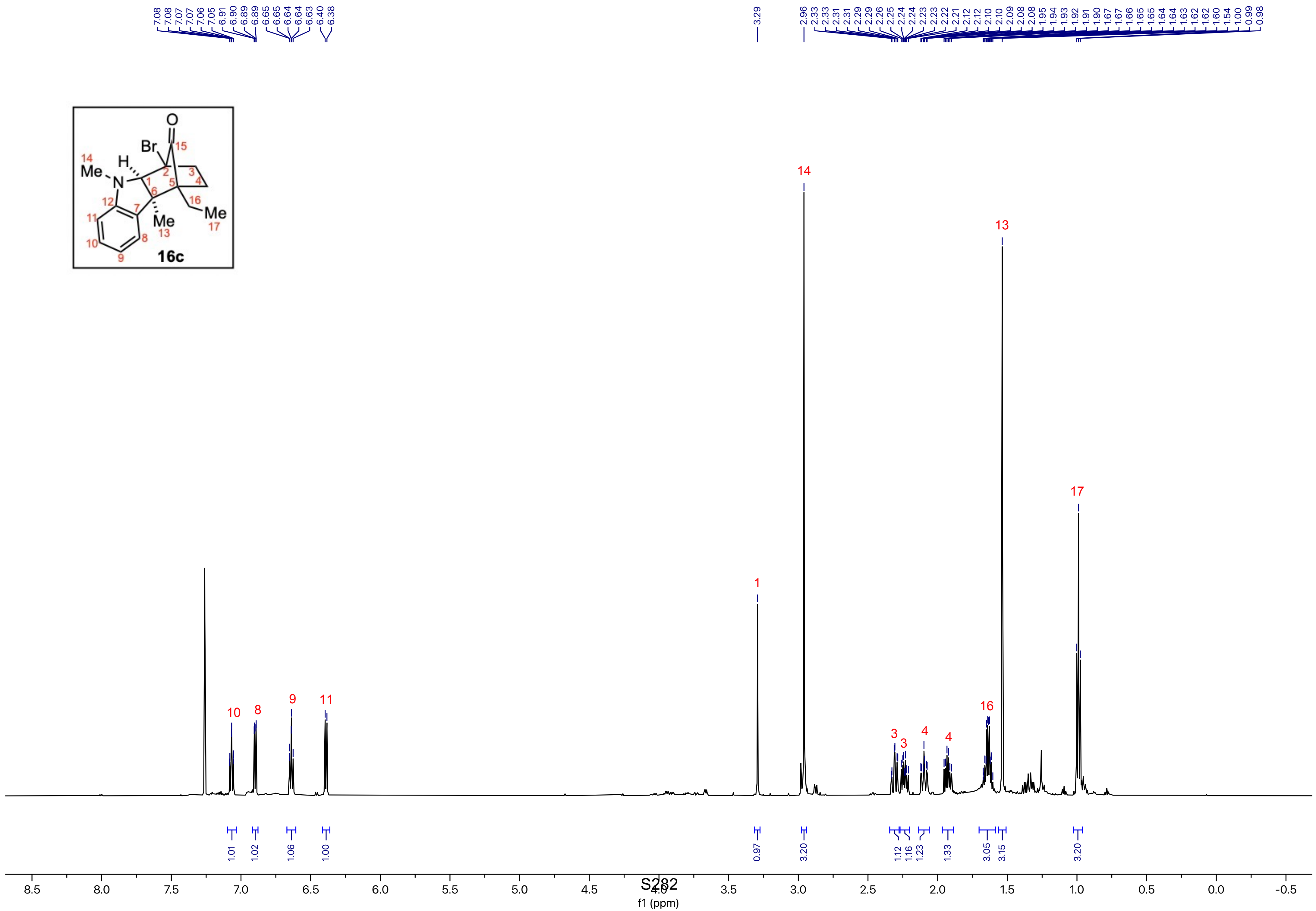
7.08 7.08 7.07 7.06 7.05 6.91 6.90 6.89 6.89 6.65 6.65 6.64 6.64 6.63 6.40 6.38
3.29
2.96 2.33 2.33 2.31 2.31 2.29 2.29 2.26 2.25 2.24 2.24 2.23 2.23 2.22 2.21 2.12 2.12 2.10 2.10 2.09 2.08 2.08 1.95 1.94 1.93 1.92 1.91 1.90 1.67 1.66 1.65 1.65 1.64 1.64 1.63 1.62 1.62 1.60 1.54 1.00 0.99 0.98
O
Br
H
Me
N
Me
Me
16c
10 8 9 11 1 14 3 3 4 4 16 13 17
1.01 1.02 1.06 1.00 0.97 3.20 1.12 1.16 1.23 1.33 3.05 3.15 3.20
8.5 8.0 7.5 7.0 6.5 6.0 5.5 5.0 4.5 4.0 3.5 3.0 2.5 2.0 1.5 1.0 0.5 0.0 -0.5
f1 (ppm)

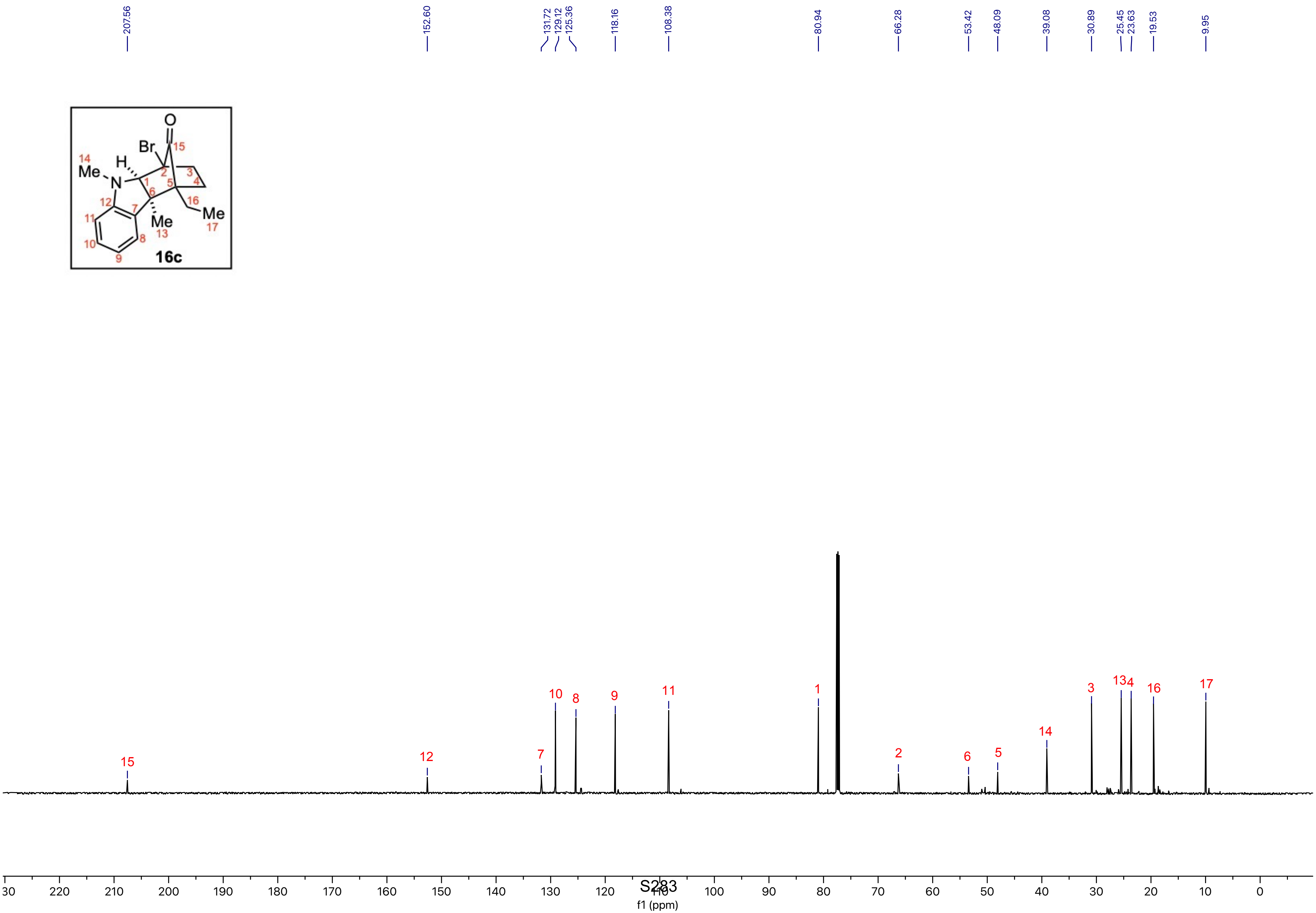

207.56
152.60
131.72
129.12
125.36
118.16
108.38
80.94
66.28
53.42
48.09
39.08
30.89
25.45
23.63
19.53
9.95
16c
15
12
7
10
8
9
11
1
2
6
5
14
3
13
4
16
17
f1 (ppm)

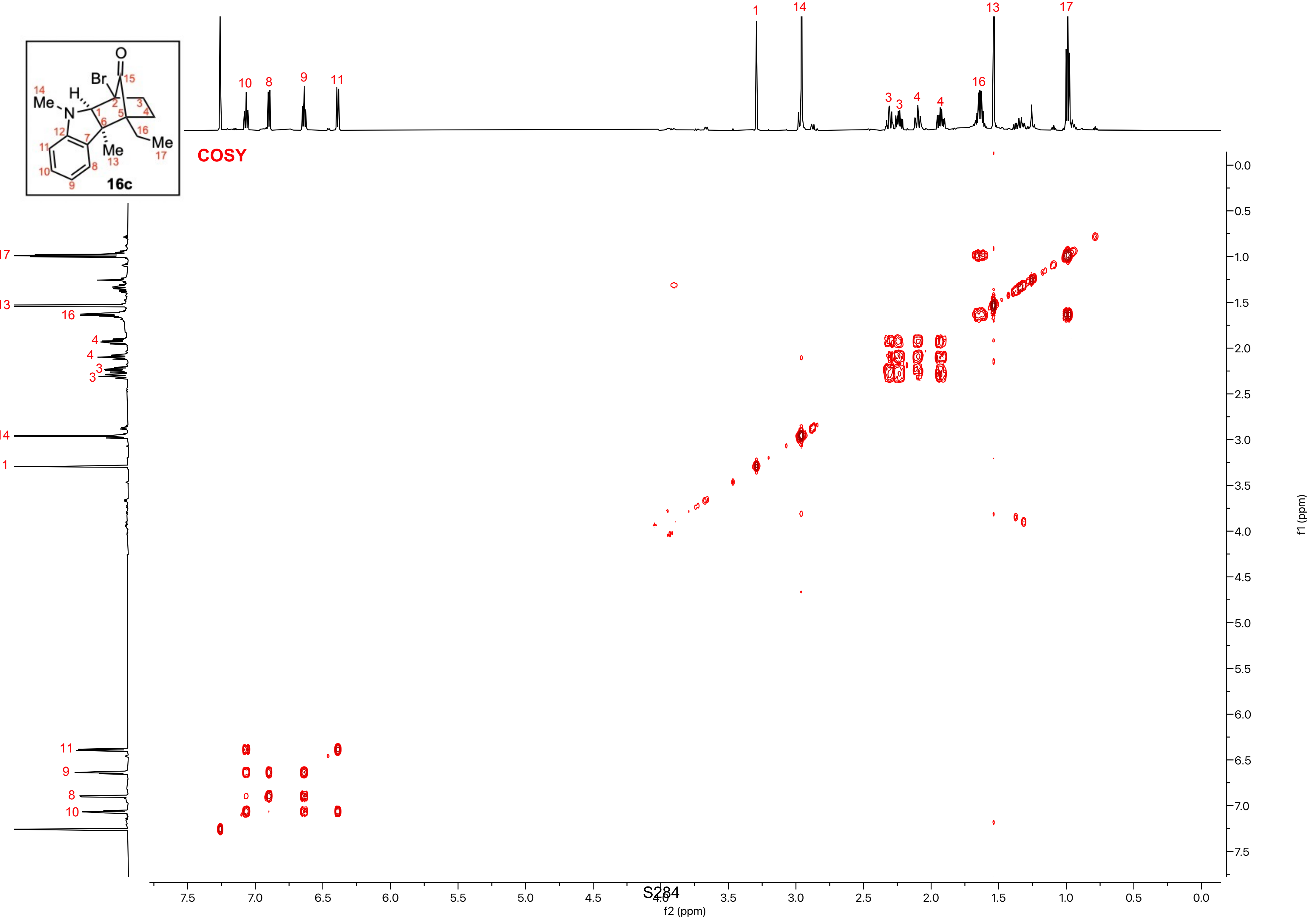

O
Br
15
14
Me
H
N
1
2
3
4
5
6
16
Me
17
12
7
Me
13
11
10
9
8
16c
COSY
1
14
10
8
9
11
3
3
4
4
16
13
17
f2 (ppm)
f1 (ppm)

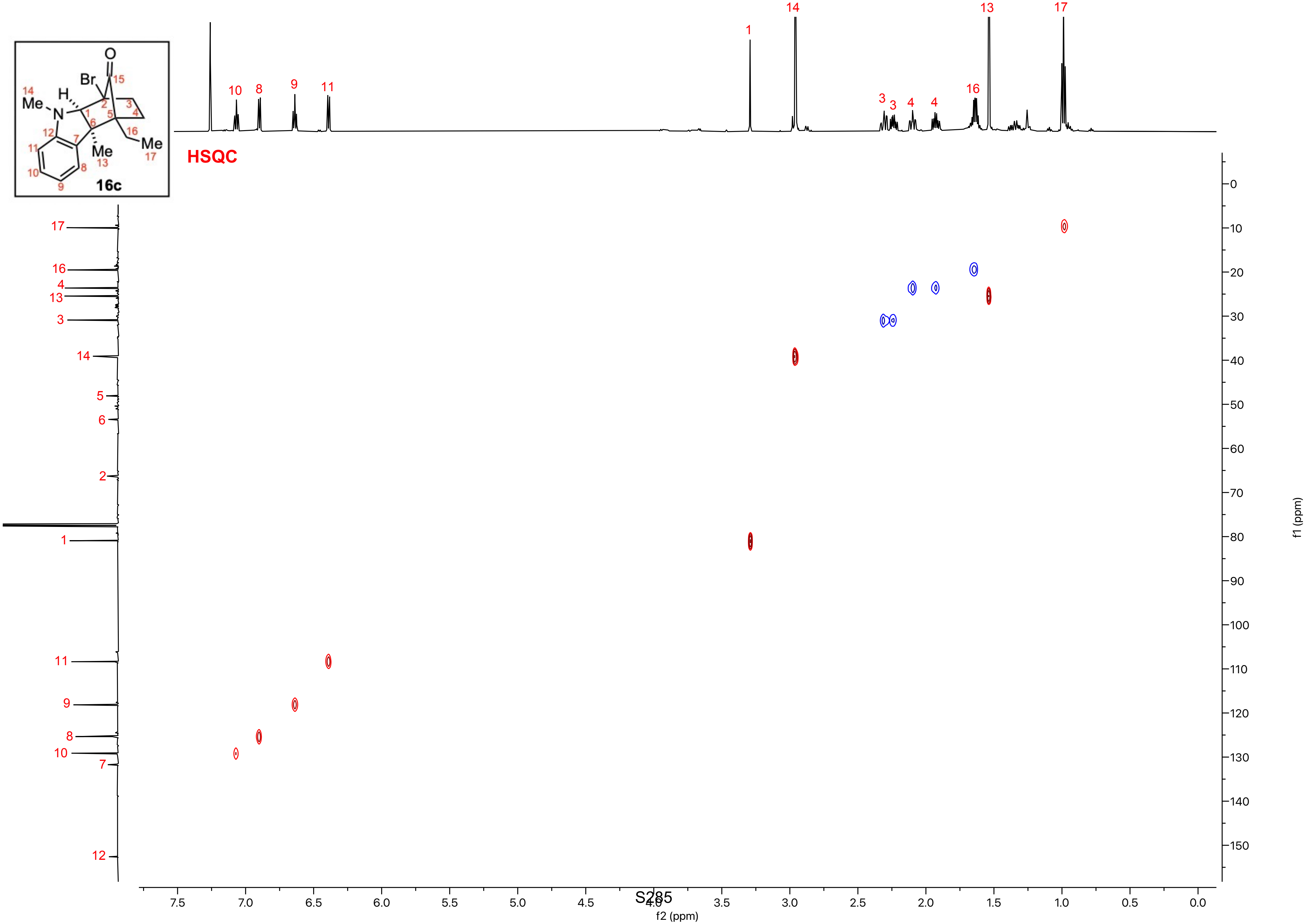

16c
HSQC
f2 (ppm)
f1 (ppm)

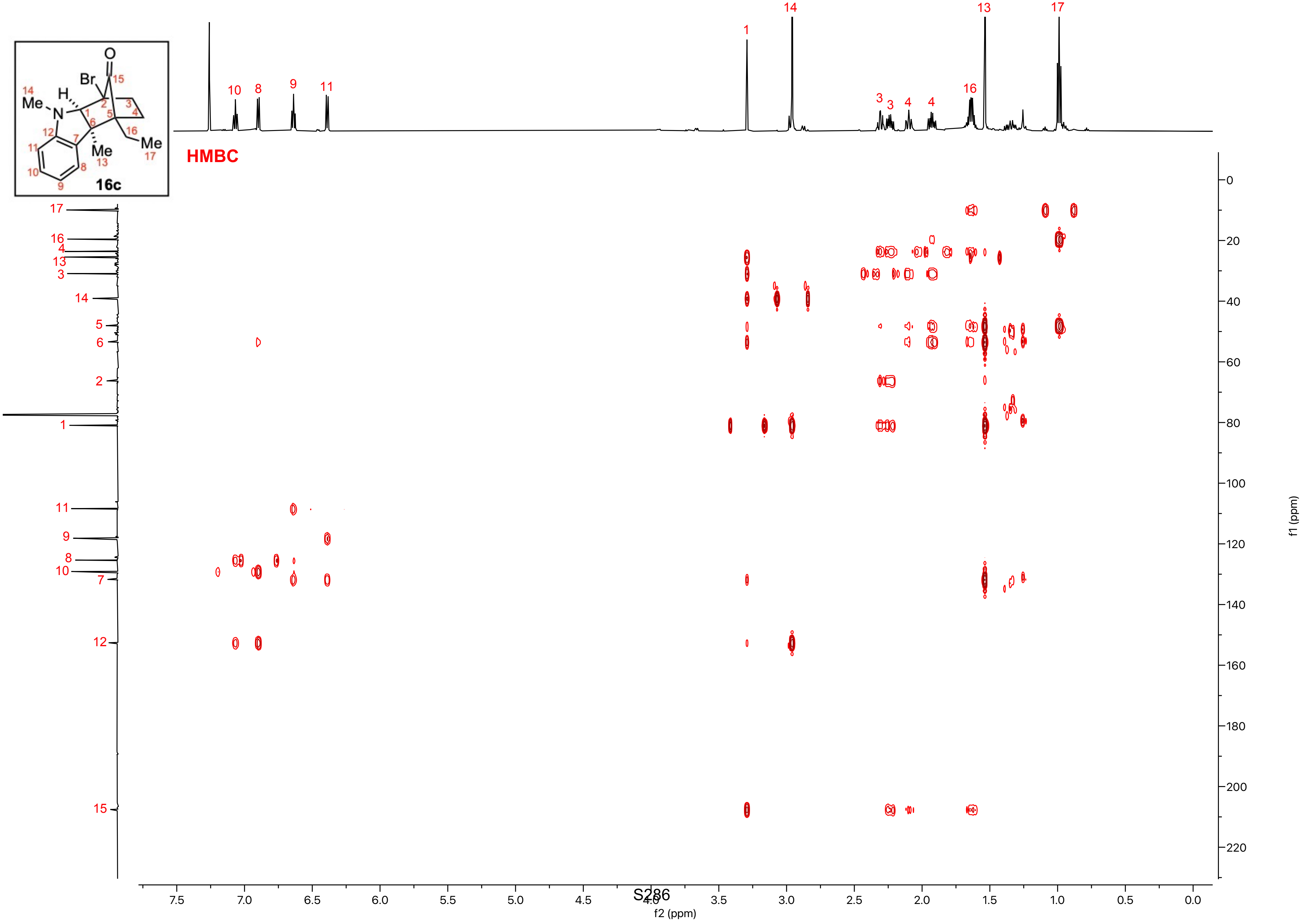

HMBC
16c
f2 (ppm)
f1 (ppm)

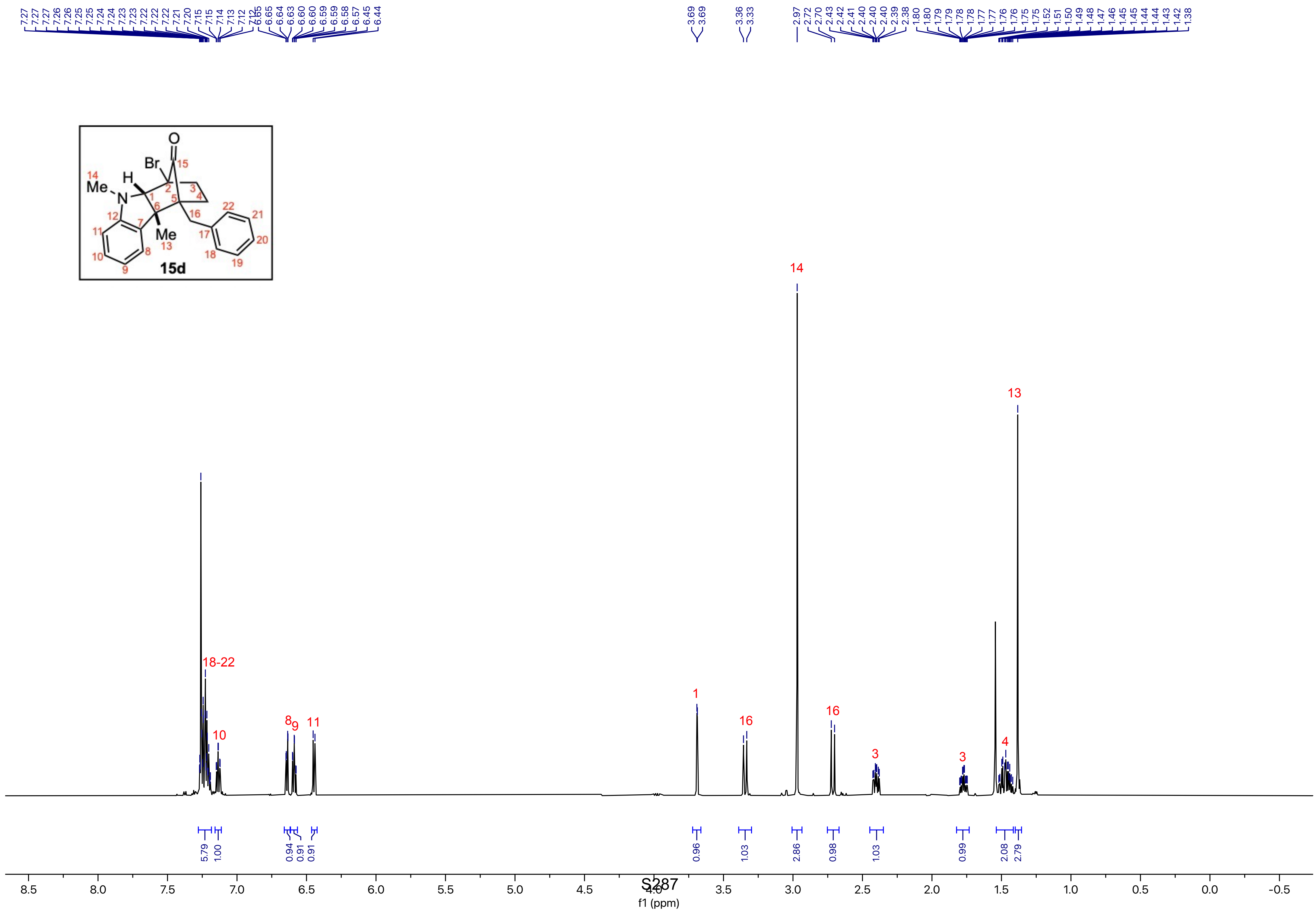
15d
18-22
10
8
9
11
1
16
14
16
3
3
4
13
f1 (ppm)

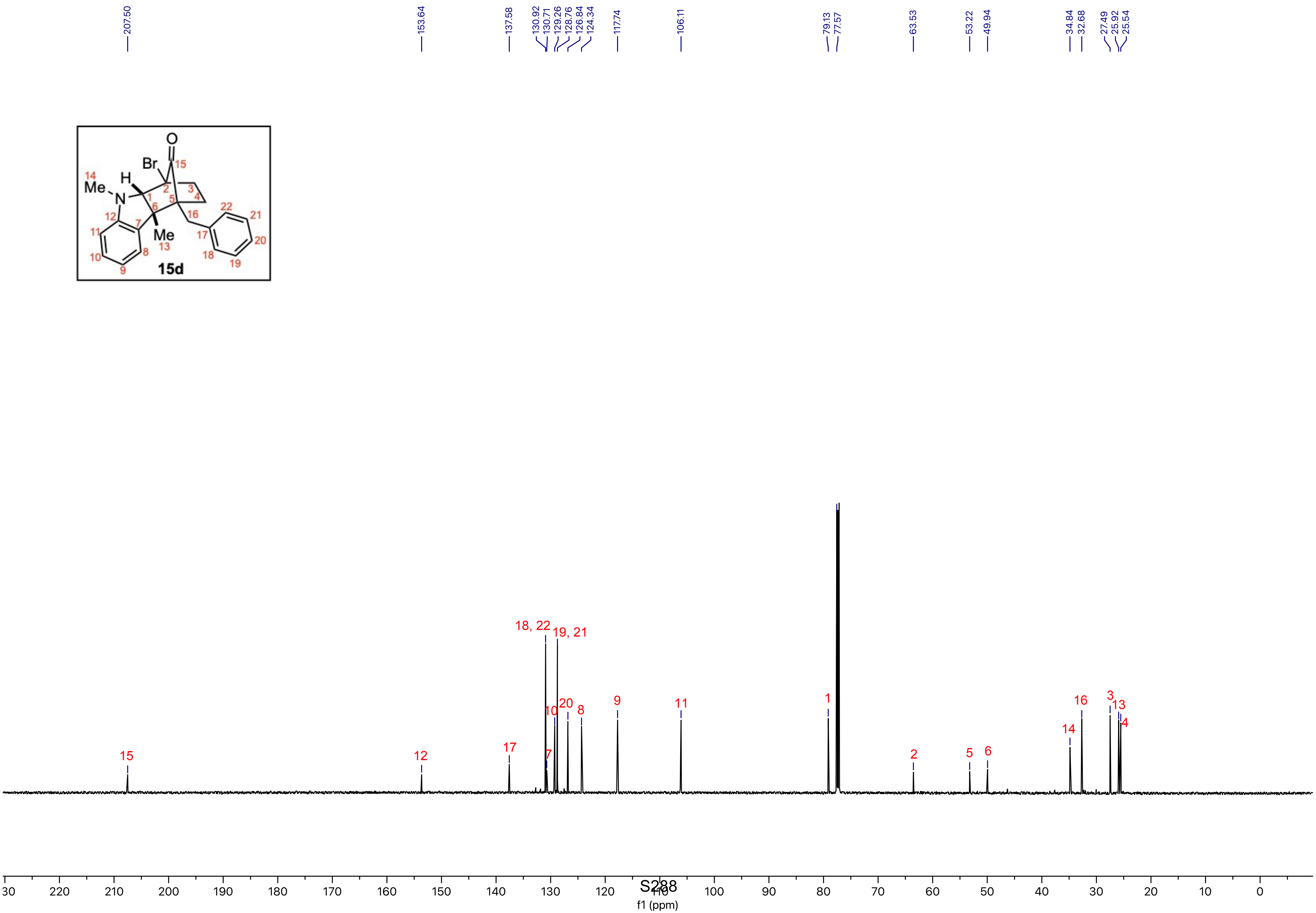
207.50
153.64
137.58
130.92
130.71
129.26
128.76
126.84
124.34
117.74
106.11
79.13
77.57
63.53
53.22
49.94
34.84
32.68
27.49
25.92
25.54
15d
f1 (ppm)

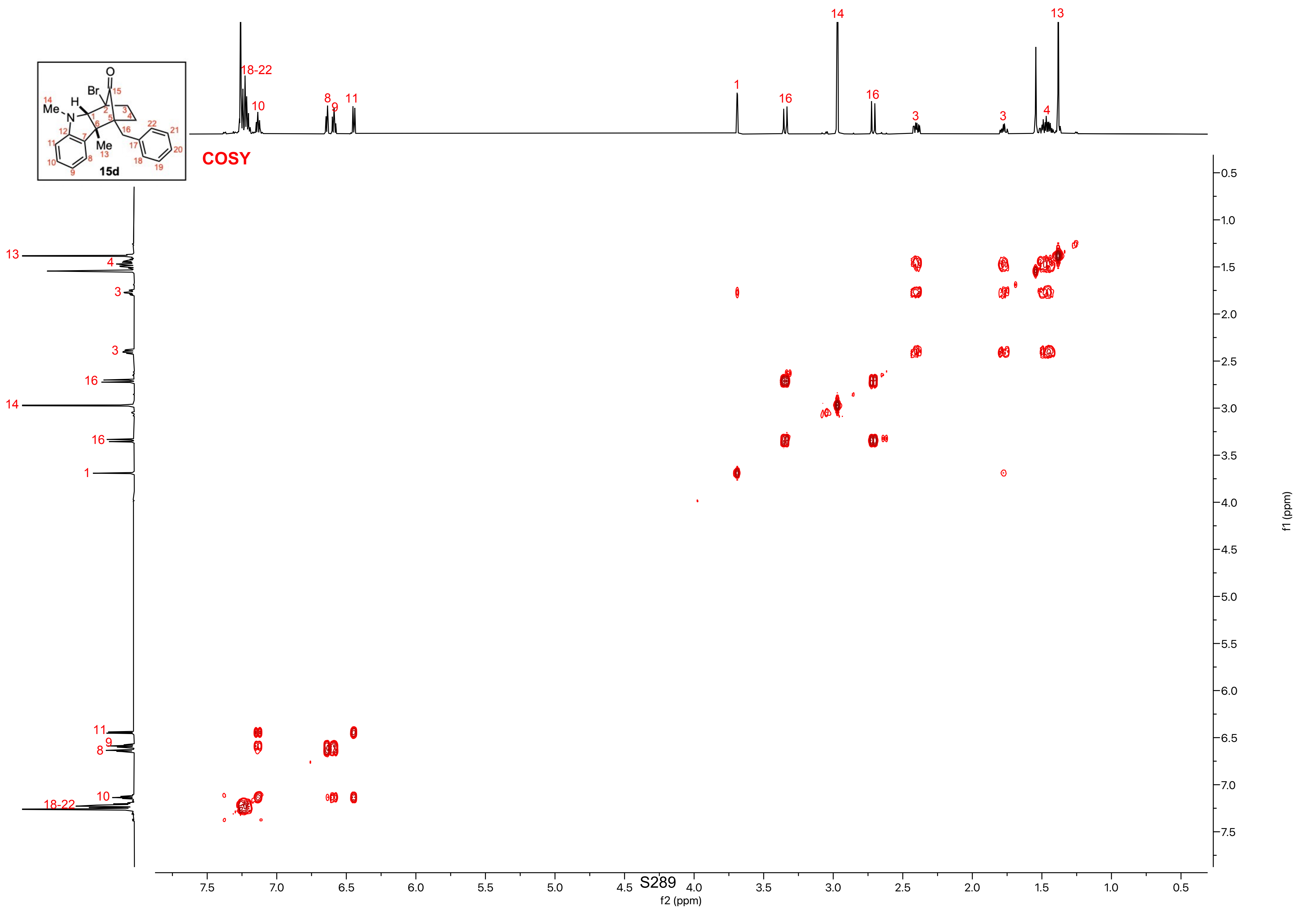
15d
COSY
18-22
10
8
9
11
1
16
14
16
3
3
4
13
f2 (ppm)
f1 (ppm)

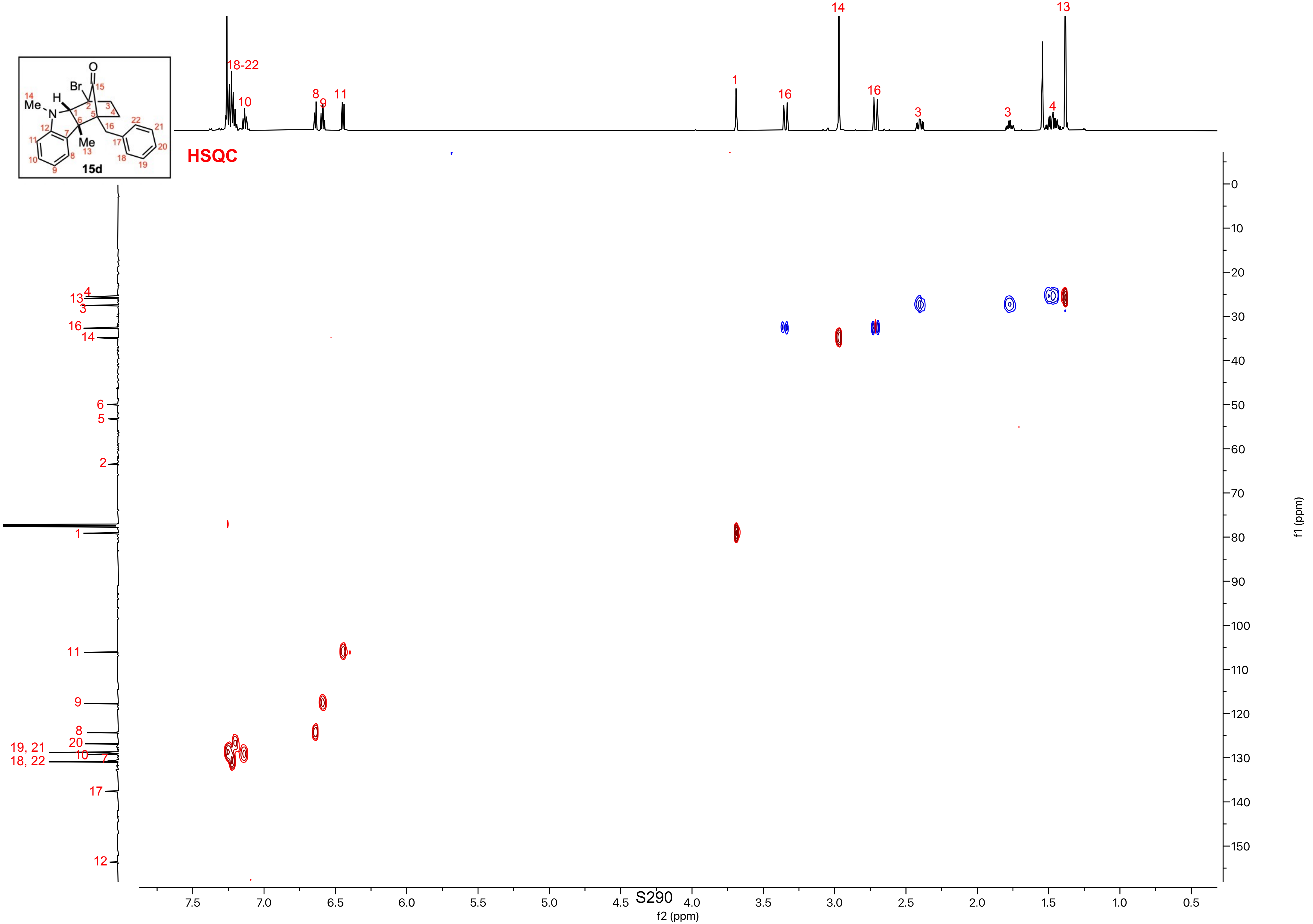

15d
HSQC
18-22
10
8
9
11
1
16
14
16
3
3
4
13
f2 (ppm)
f1 (ppm)

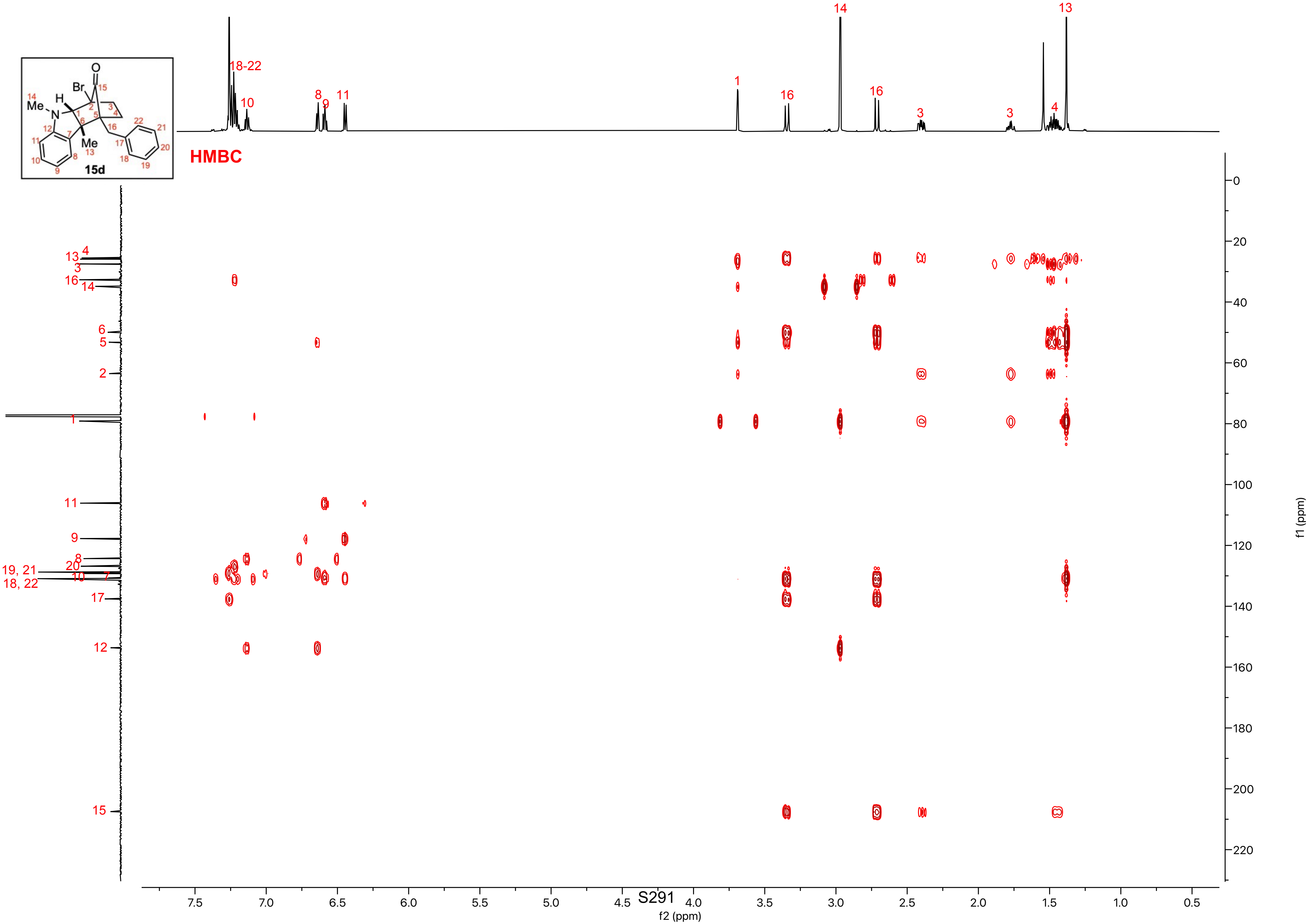

HMBC
15d
18-22
10
8
9
11
1
16
14
16
3
3
4
13
f2 (ppm)
f1 (ppm)

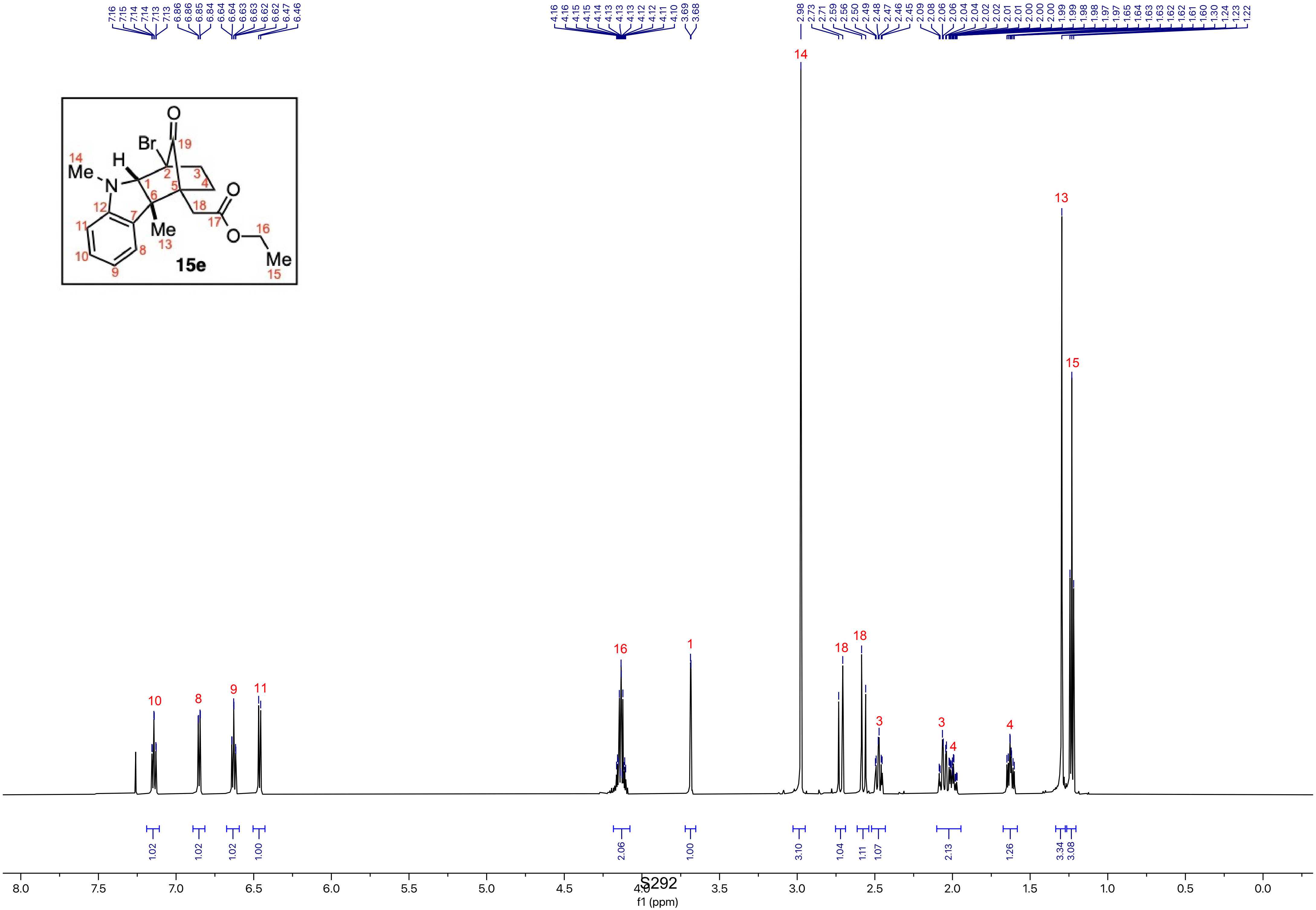
7.16
7.15
7.14
7.14
7.13
7.13
6.86
6.86
6.85
6.84
6.64
6.64
6.63
6.63
6.62
6.62
6.47
6.46
4.16
4.16
4.15
4.15
4.14
4.13
4.13
4.13
4.12
4.12
4.11
4.10
3.69
3.68
2.98
2.73
2.71
2.59
2.56
2.50
2.49
2.48
2.47
2.46
2.45
2.09
2.08
2.06
2.06
2.04
2.04
2.02
2.02
2.01
2.01
2.00
2.00
2.00
1.99
1.99
1.98
1.98
1.97
1.97
1.65
1.64
1.63
1.63
1.62
1.62
1.61
1.60
1.30
1.24
1.23
1.22
O
Br
19
H
14
Me
N
1
2
3
5
4
O
6
18
12
7
11
Me
13
17
O
16
10
8
15e
Me
9
15
14
13
15
10
8
9
11
16
1
18
18
3
3
4
4
1.02
1.02
1.02
1.00
2.06
1.00
3.10
1.04
1.11
1.07
2.13
1.26
3.34
3.08
8.0
7.5
7.0
6.5
6.0
5.5
5.0
4.5
4.0
3.5
3.0
2.5
2.0
1.5
1.0
0.5
0.0
f1 (ppm)

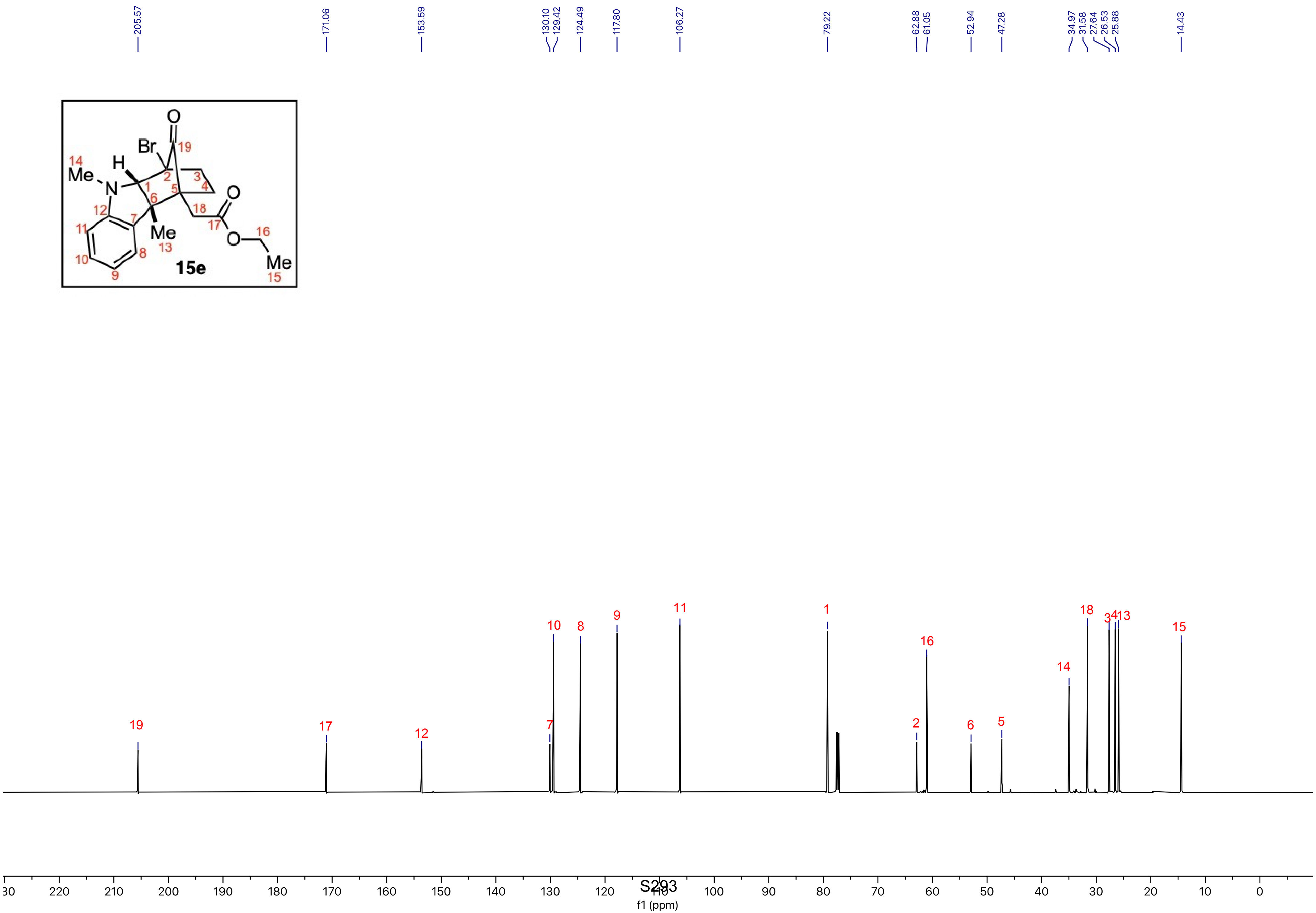
205.57
171.06
153.59
130.10
129.42
124.49
117.80
106.27
79.22
62.88
61.05
52.94
47.28
34.97
31.58
27.64
26.53
25.88
14.43
15e
Br
Me
N
H
O
19
17
12
7
10
8
9
11
1
2
16
6
5
14
18
3
4
13
15
30
220
210
200
190
180
170
160
150
140
130
120
110
100
90
80
70
60
50
40
30
20
10
0
f1 (ppm)

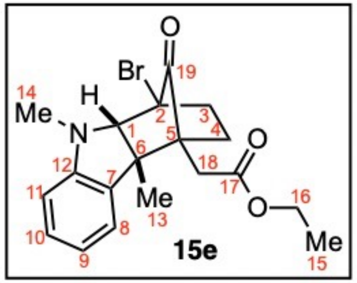
O
Br
19
H
14
Me
N
1
2
3
4
5
6
O
18
17
O
16
Me
15
Me
13
12
7
11
10
9
8

15e

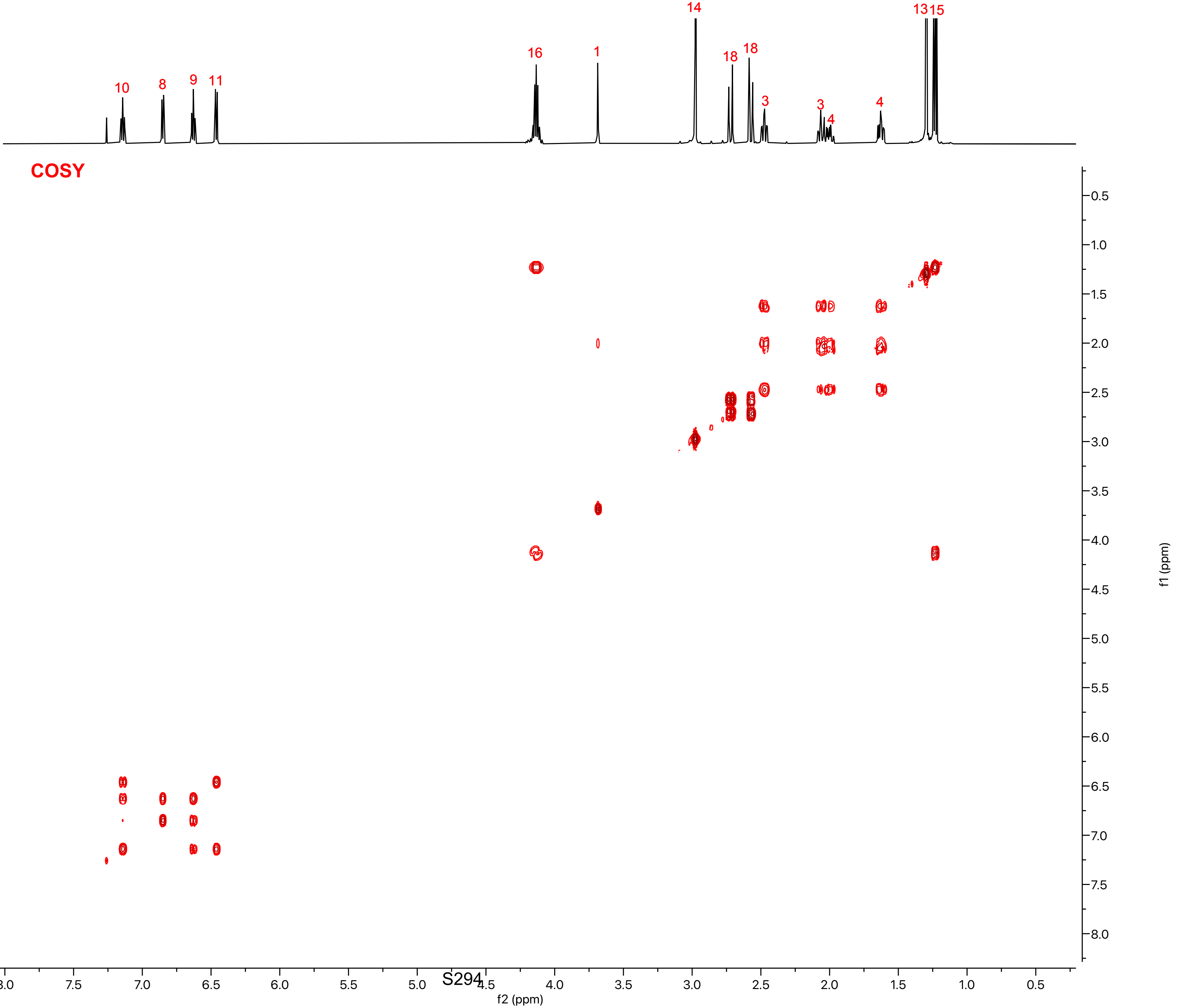
COSY
10
8
9
11
16
1
14
18
18
3
3
4
4
13
15
f2 (ppm)
f1 (ppm)
8.0
7.5
7.0
6.5
6.0
5.5
5.0
4.5
4.0
3.5
3.0
2.5
2.0
1.5
1.0
0.5

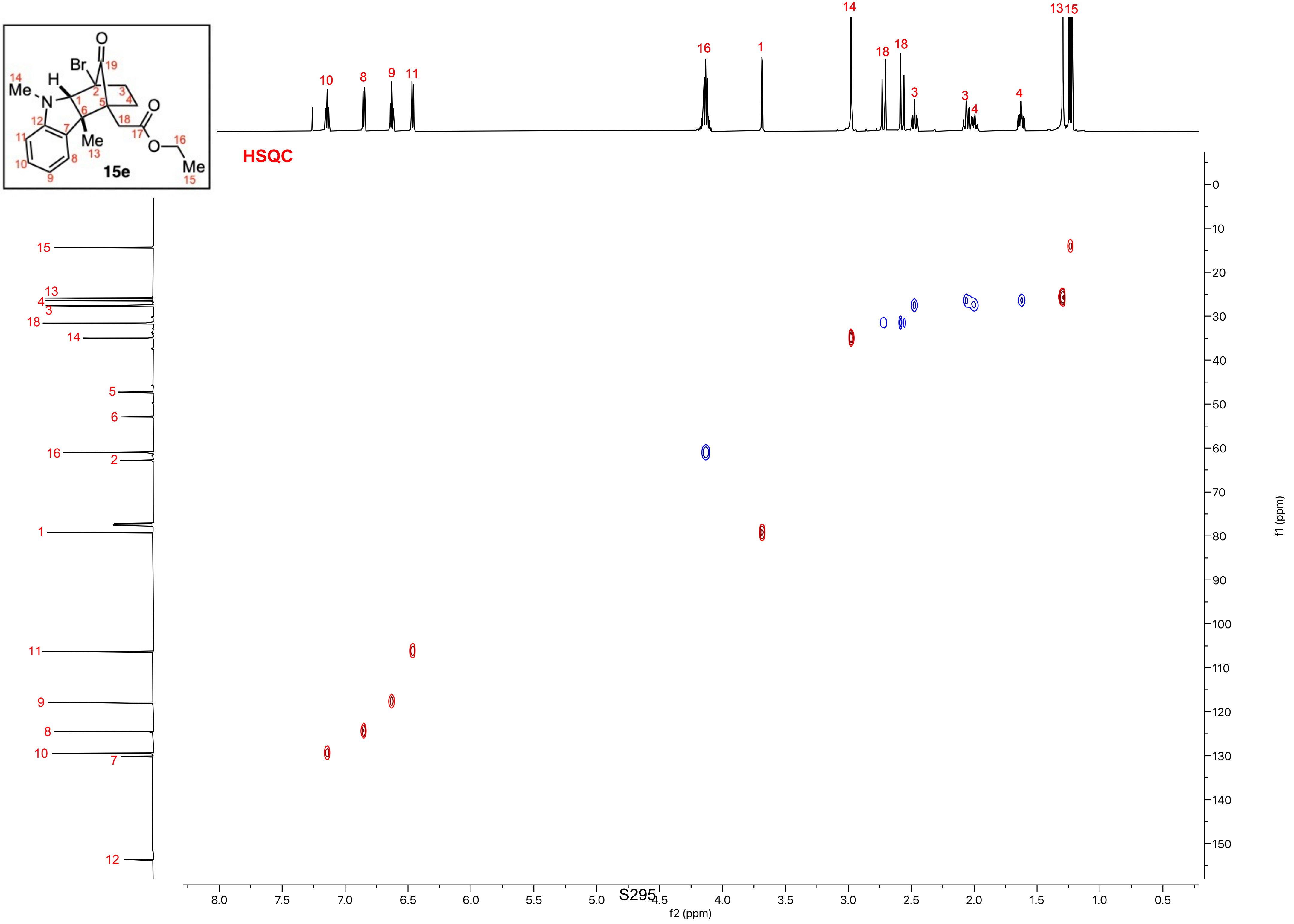
15e
HSQC
f2 (ppm)
f1 (ppm)

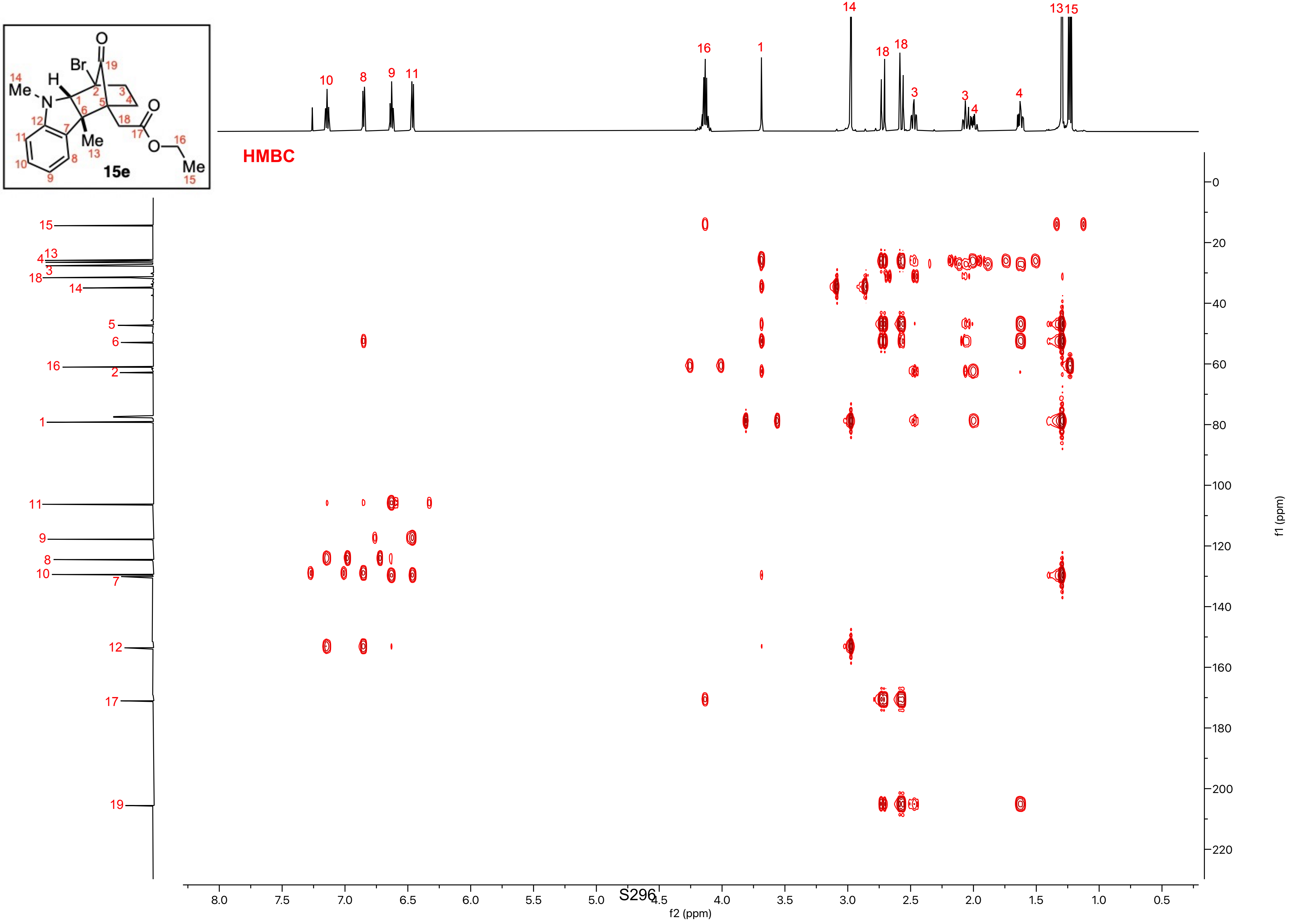

15e
HMBC
f2 (ppm)
f1 (ppm)

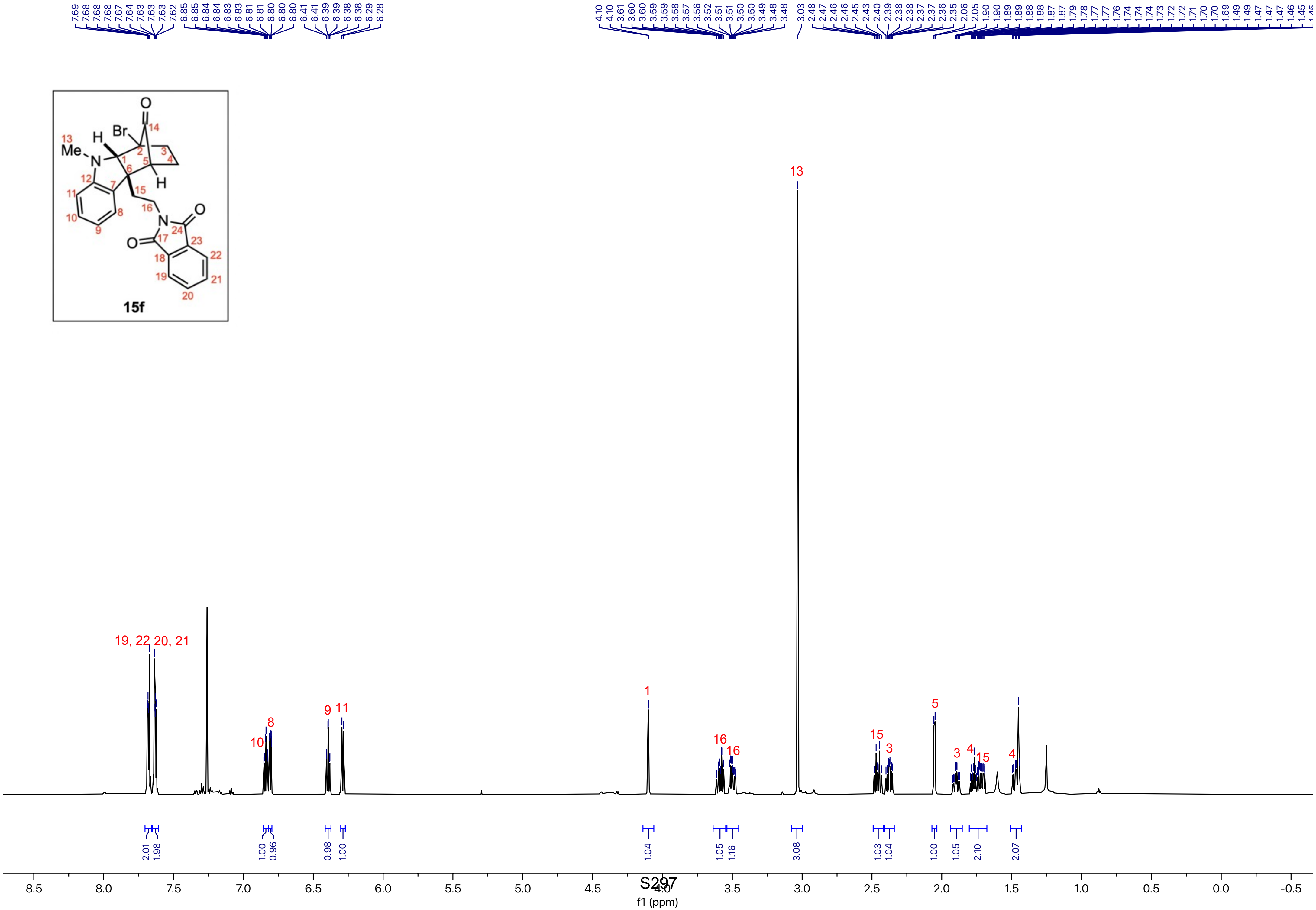
7.69 7.68 7.68 7.67 7.64 7.63 7.63 7.62 6.85 6.84 6.84 6.83 6.83 6.81 6.81 6.80 6.80 6.80 6.41 6.41 6.39 6.39 6.38 6.38 6.29 6.28
4.10 4.10 3.61 3.60 3.60 3.59 3.59 3.58 3.57 3.56 3.52 3.51 3.51 3.50 3.50 3.49 3.48 3.48 3.03 2.48 2.47 2.46 2.46 2.45 2.43 2.40 2.39 2.38 2.38 2.37 2.36 2.35 2.06 2.05 1.90 1.90 1.89 1.89 1.88 1.88 1.87 1.87 1.79 1.78 1.77 1.77 1.76 1.74 1.74 1.74 1.73 1.72 1.72 1.71 1.70 1.70 1.69 1.49 1.49 1.47 1.47 1.47 1.46 1.45
15f
19, 22
20, 21
10
8
9
11
1
16
16
13
15
3
5
3
4
15
4
2.01 1.98 1.00 0.96 0.98 1.00 1.04 1.05 1.16 3.08 1.03 1.04 1.00 1.05 2.10 2.07
f1 (ppm)

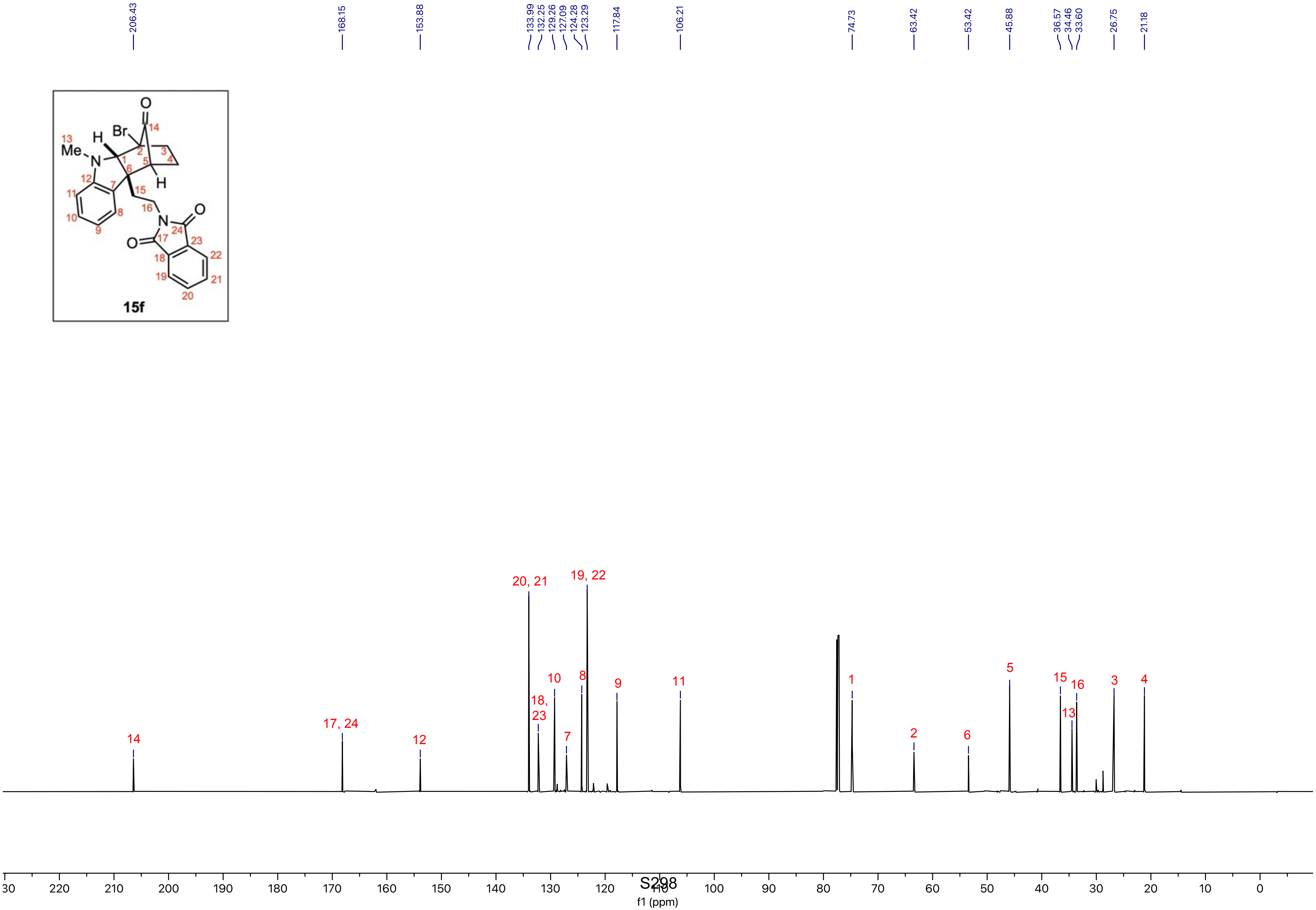
206.43
168.15
153.88
133.99
132.25
129.26
127.09
124.28
123.29
117.84
106.21
74.73
63.42
53.42
45.88
36.57
34.46
33.60
26.75
21.18
15f
14
17, 24
12
20, 21
18, 23
10
7
8
19, 22
9
11
1
2
6
5
15
13
16
3
4
f1 (ppm)

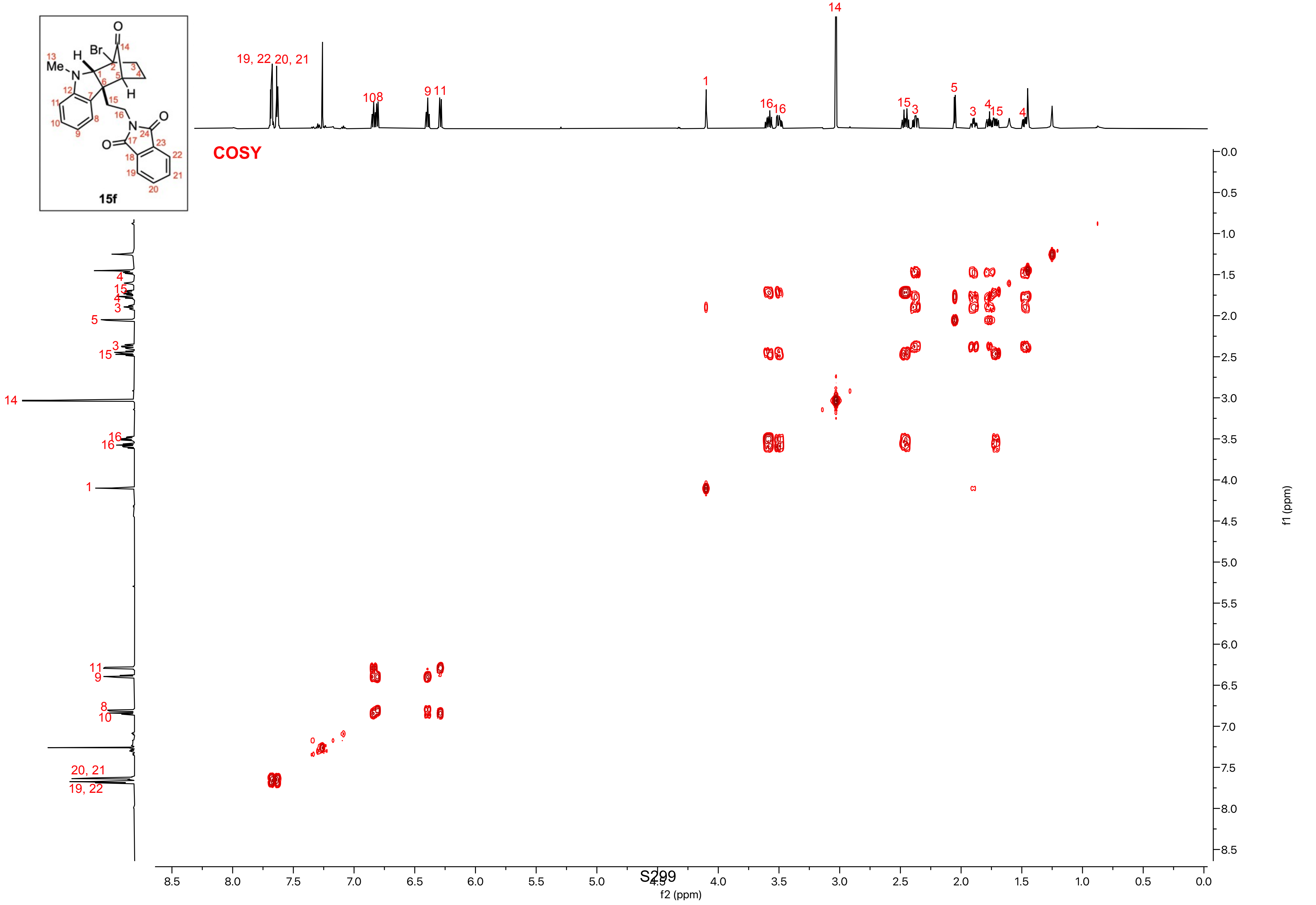

15f
COSY
19, 22 20, 21
108
9 11
1
16 16
14
15 3
5
3 4 15
4
f2 (ppm)
f1 (ppm)

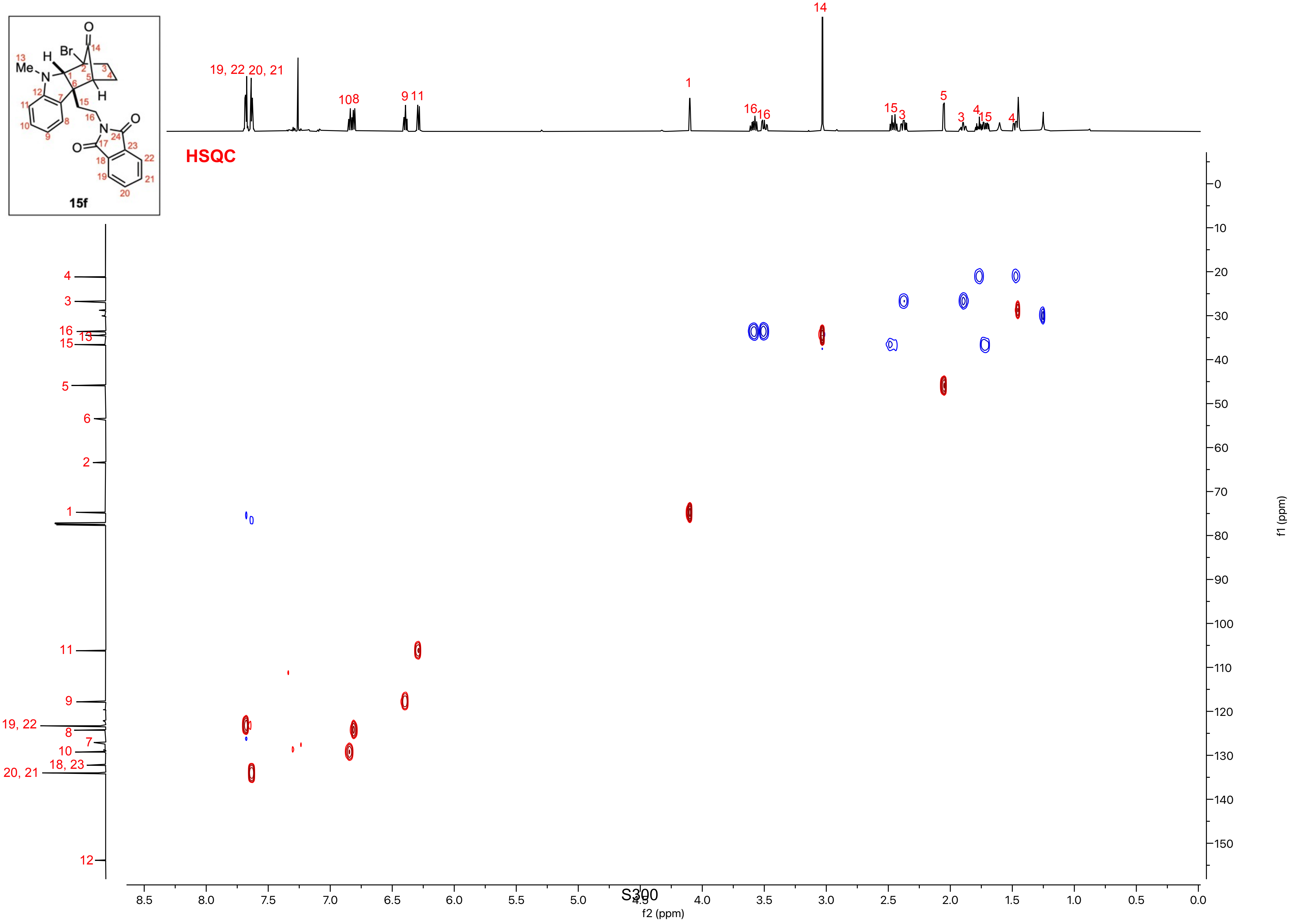

HSQC
15f
19, 22
20, 21
14
1
108
9
11
16
16
15
3
5
3
4
15
4
4
3
16
13
15
5
6
2
1
11
9
19, 22
8
7
10
18, 23
20, 21
12
f2 (ppm)
f1 (ppm)

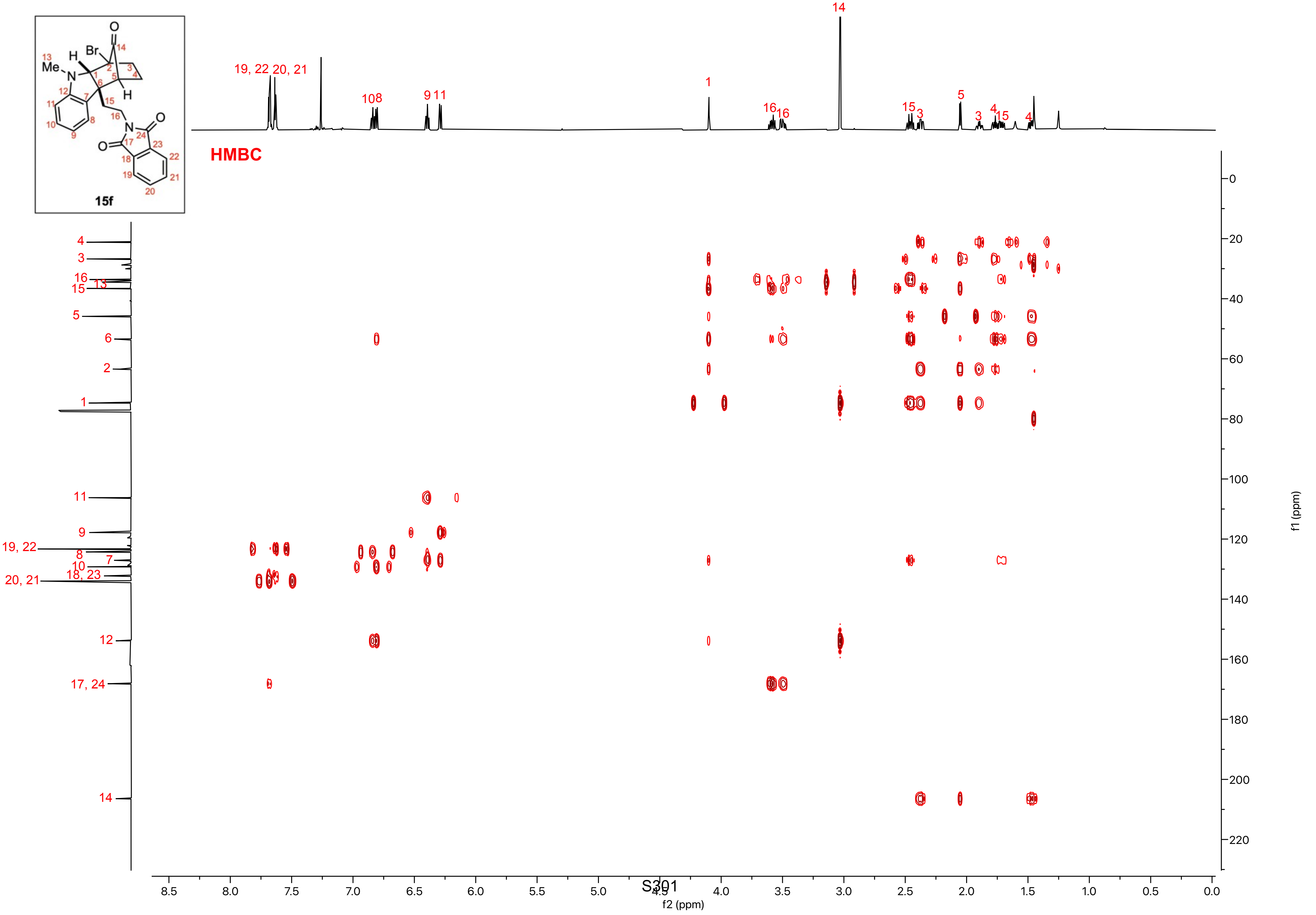
HMBC
15f
f2 (ppm)
f1 (ppm)

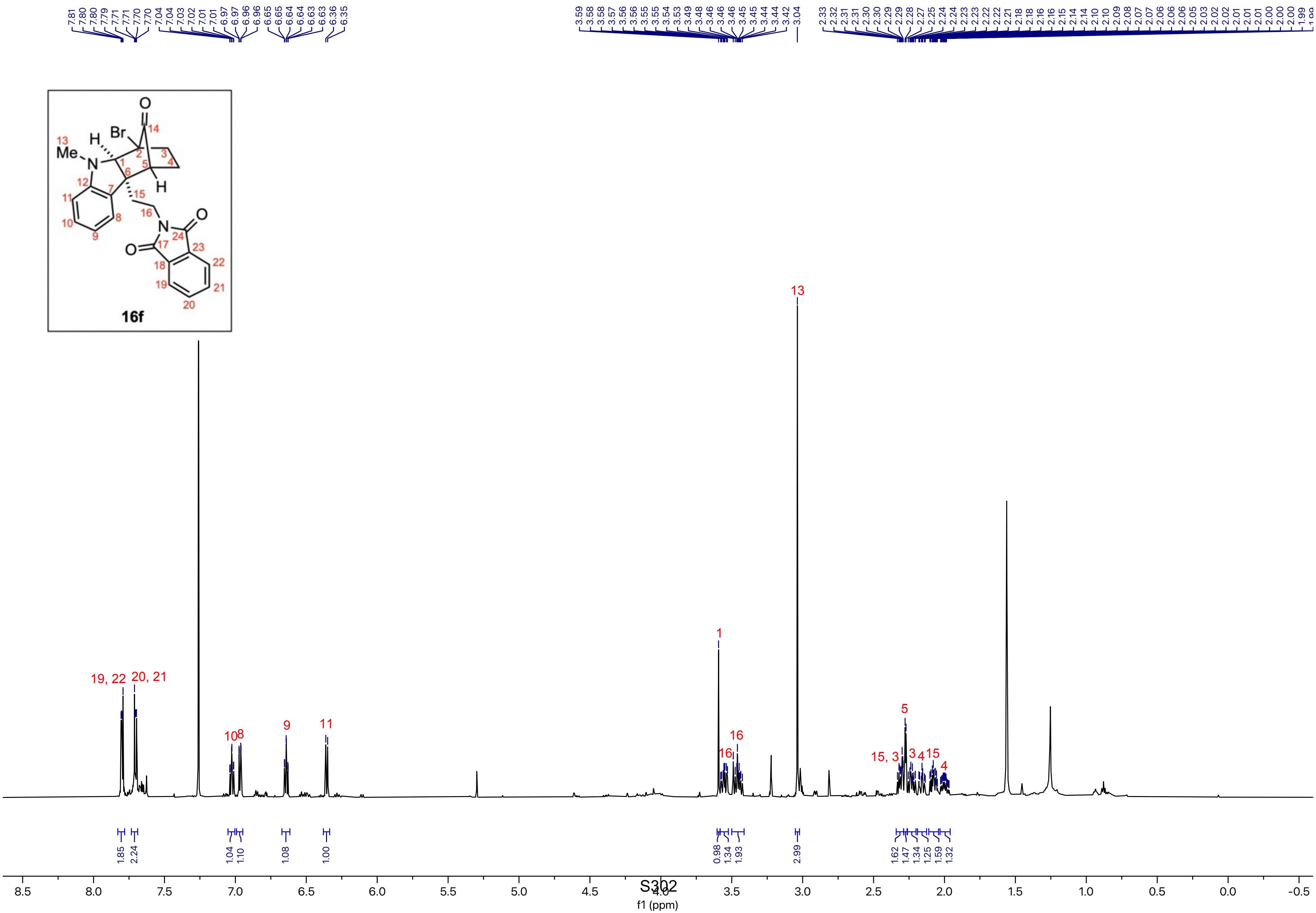

7.81 7.80 7.80 7.79 7.71 7.71 7.70 7.70 7.04 7.04 7.03 7.02 7.01 7.01 6.97 6.97 6.96 6.96 6.65 6.65 6.64 6.64 6.63 6.63 6.36 6.35
3.59 3.58 3.58 3.57 3.56 3.56 3.55 3.55 3.54 3.53 3.49 3.48 3.46 3.46 3.46 3.45 3.44 3.44 3.42 3.04
2.33 2.32 2.31 2.31 2.30 2.30 2.29 2.29 2.28 2.27 2.25 2.24 2.24 2.23 2.23 2.22 2.22 2.21 2.18 2.18 2.16 2.16 2.15 2.14 2.10 2.10 2.09 2.08 2.07 2.06 2.06 2.05 2.03 2.02 2.02 2.01 2.01 2.00 2.00 1.99
16f
19, 22
20, 21
10
8
9
11
1
16
16
13
15, 3
5
3
4
15
4
1.85 2.24 1.04 1.10 1.08 1.00 0.98 1.34 1.93 2.99 1.62 1.47 1.34 1.25 1.59 1.32
8.5 8.0 7.5 7.0 6.5 6.0 5.5 5.0 4.5 4.0 3.5 3.0 2.5 2.0 1.5 1.0 0.5 0.0 -0.5
f1 (ppm)

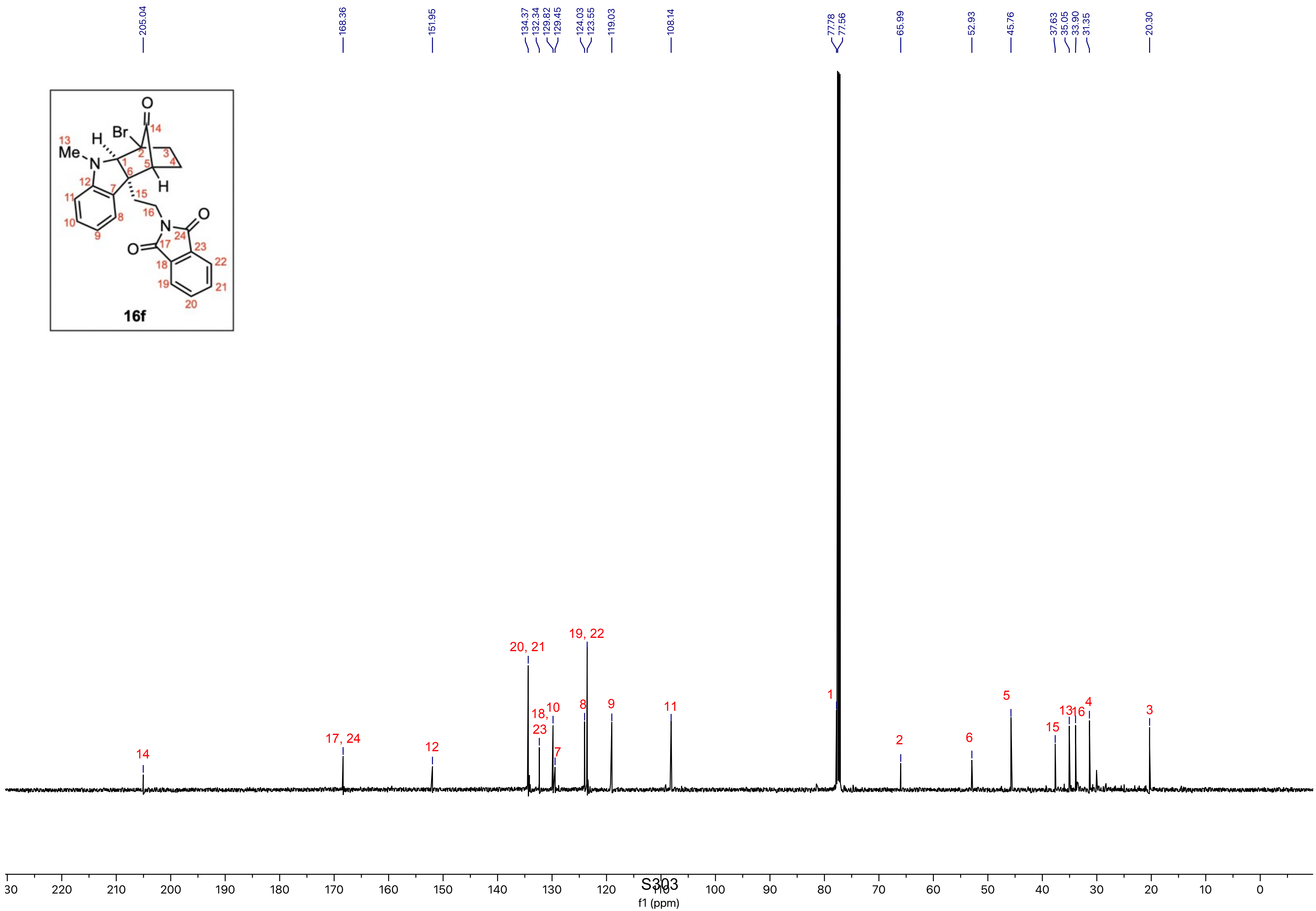
205.04
168.36
151.95
134.37
132.34
129.82
129.45
124.03
123.55
119.03
108.14
77.78
77.56
65.99
52.93
45.76
37.63
35.05
33.90
31.35
20.30
16f
14
17, 24
12
20, 21
18, 23
10
7
19, 22
8
9
11
1
2
6
5
15
13
16
4
3
f1 (ppm)

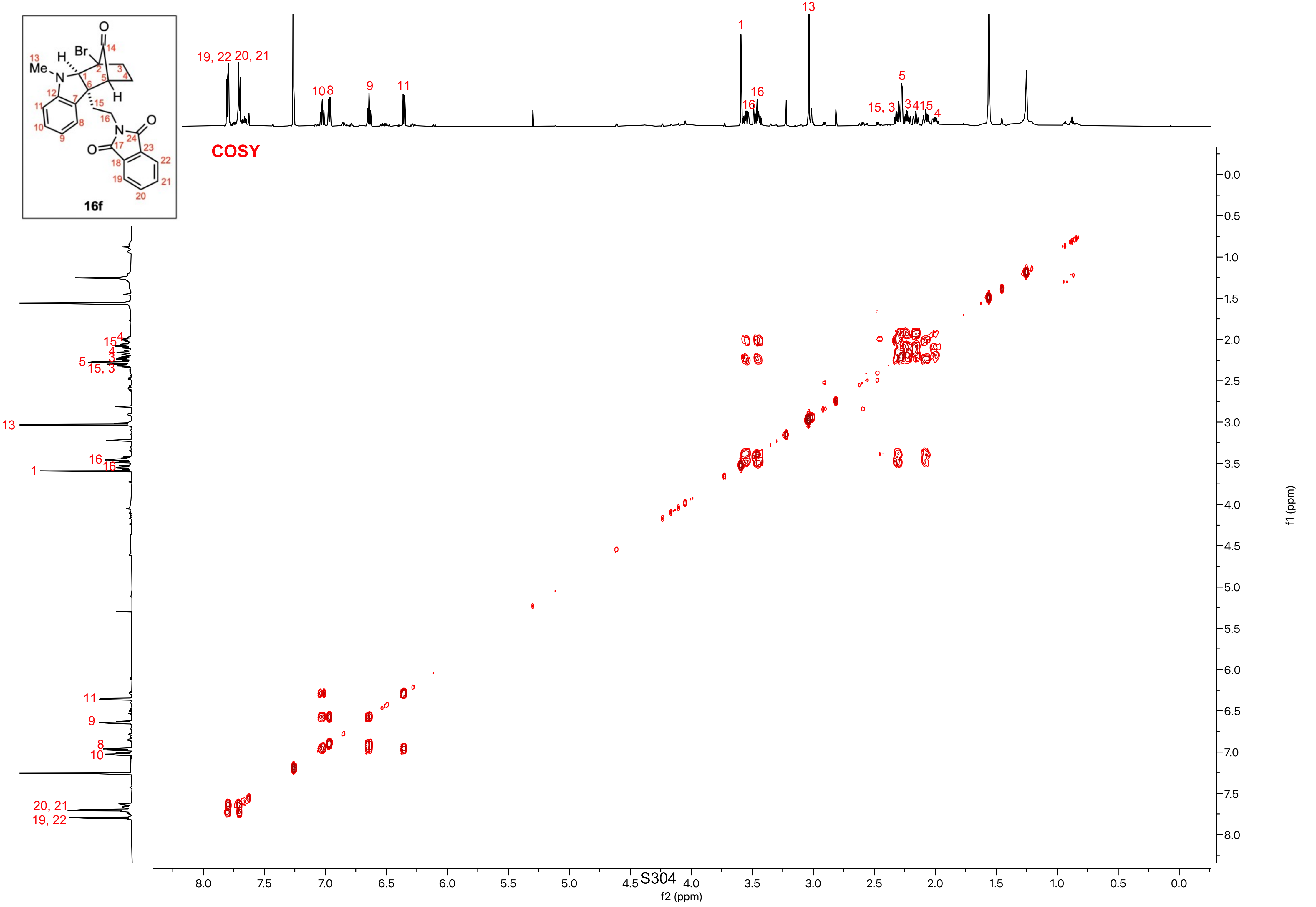

16f
COSY
f2 (ppm)
f1 (ppm)

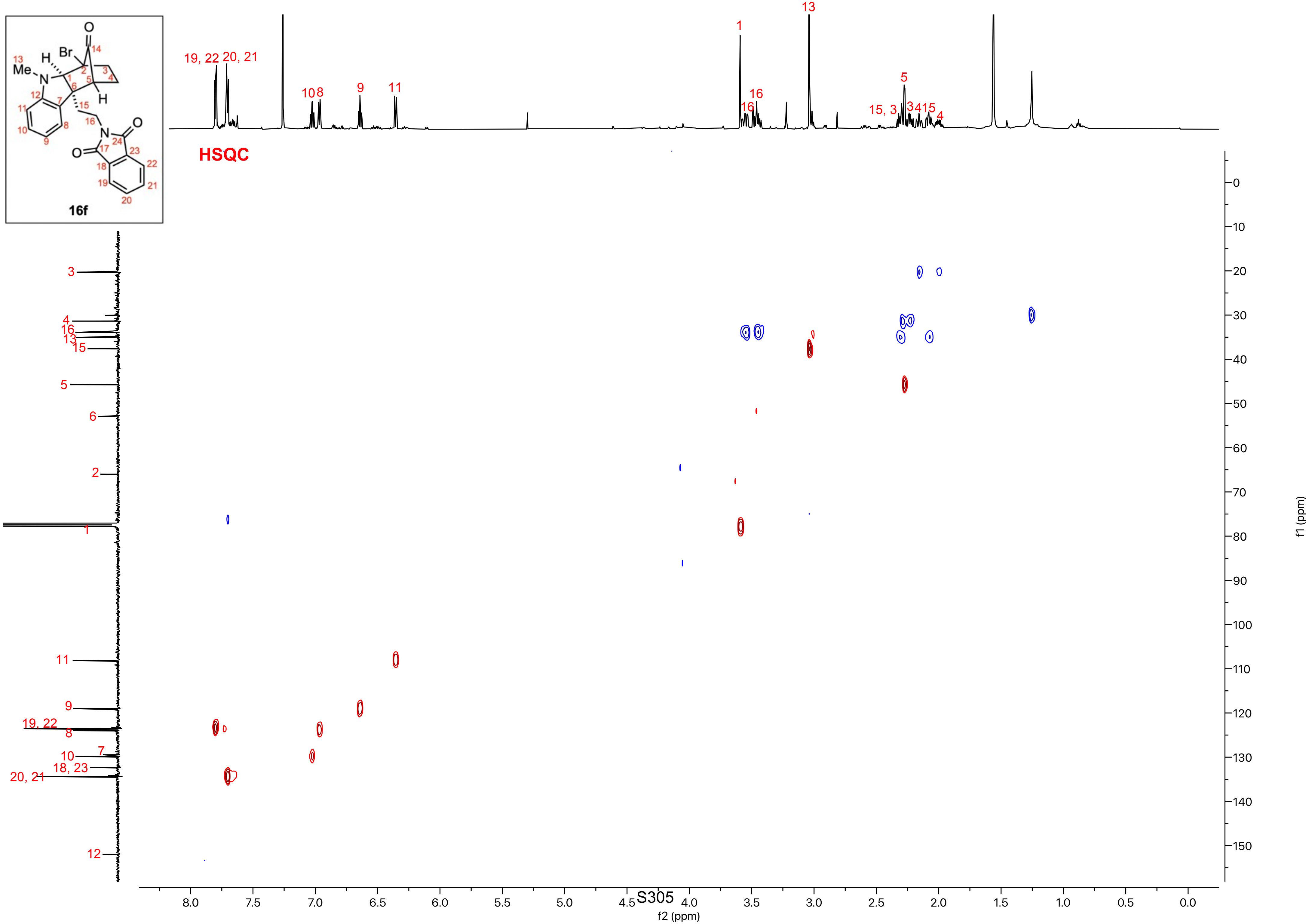
HSQC
16f
19, 22
20, 21
10
8
9
11
1
13
16
5
15, 3
3
4
15
4
3
4
16
13
15
5
6
2
1
11
9
19, 22
8
7
10
18, 23
20, 21
12
f2 (ppm)
f1 (ppm)

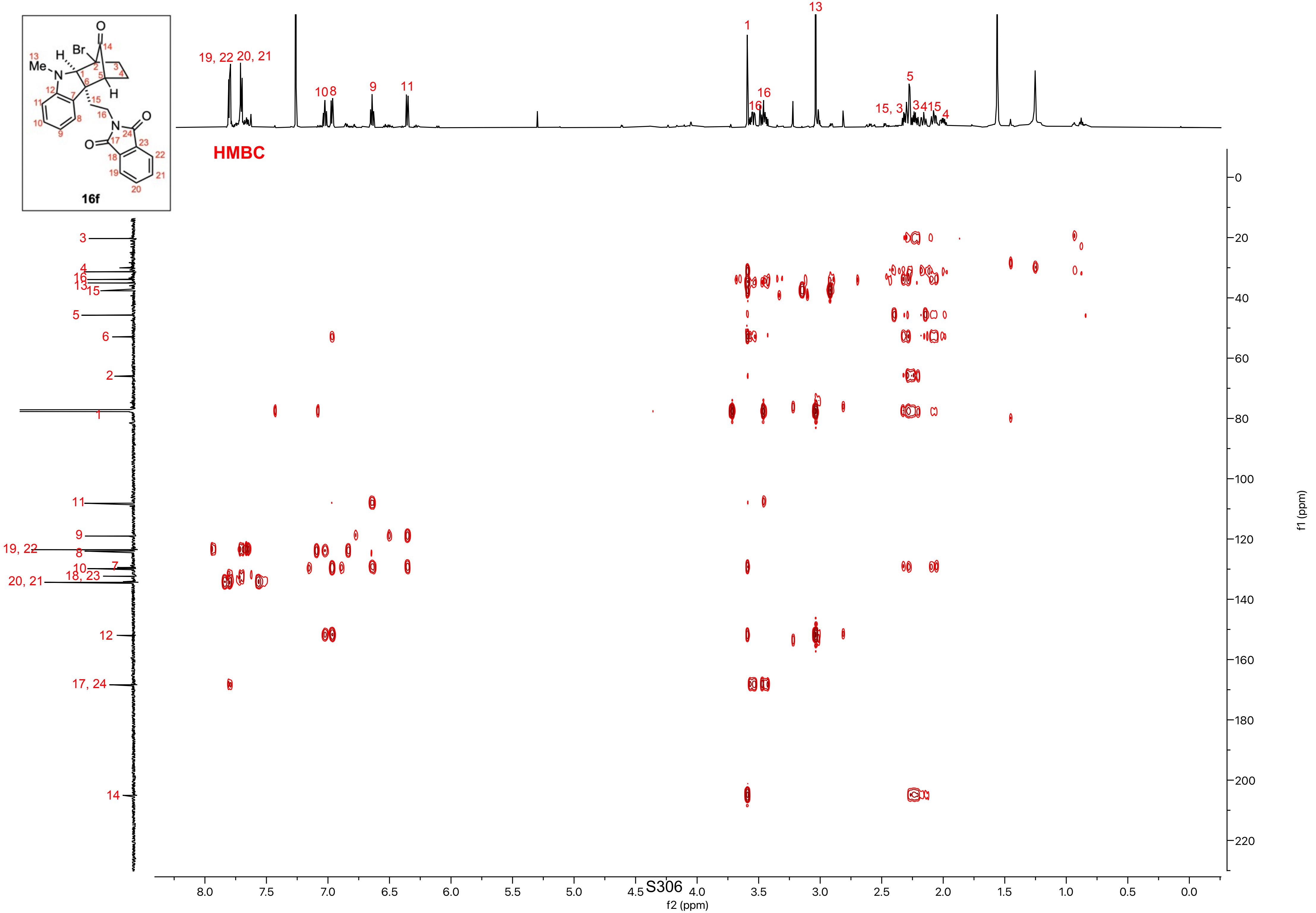

16f
HMBC
19, 22
20, 21
10
8
9
11
1
13
16
5
15, 3
3
4
15
f2 (ppm)
f1 (ppm)
6
2
12
17, 24
14
18, 23
7

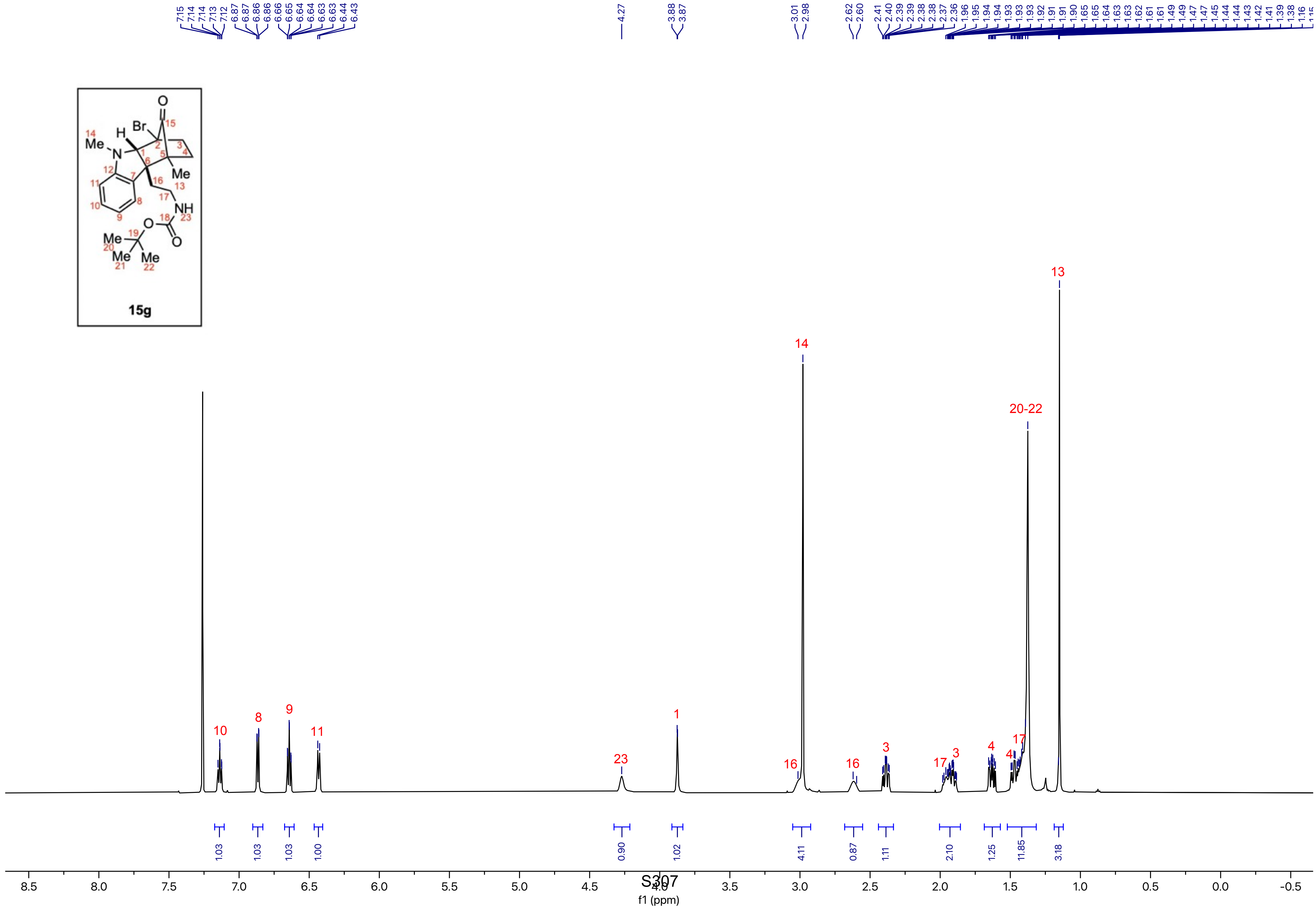

7.15
7.14
7.14
7.13
7.12
6.87
6.87
6.86
6.86
6.66
6.65
6.64
6.64
6.63
6.63
6.44
6.43
4.27
3.88
3.87
3.01
2.98
2.62
2.60
2.41
2.40
2.39
2.39
2.38
2.38
2.37
2.36
1.96
1.95
1.94
1.94
1.93
1.93
1.93
1.92
1.91
1.91
1.90
1.65
1.65
1.64
1.63
1.63
1.62
1.61
1.61
1.49
1.49
1.47
1.47
1.45
1.44
1.44
1.43
1.42
1.41
1.39
1.38
1.16
15g
13
14
20-22
10
8
9
11
23
1
16
16
3
17
3
4
4
17
1.03
1.03
1.03
1.00
0.90
1.02
4.11
0.87
1.11
2.10
1.25
11.85
3.18
8.5
8.0
7.5
7.0
6.5
6.0
5.5
5.0
4.5
4.0
3.5
3.0
2.5
2.0
1.5
1.0
0.5
0.0
-0.5
f1 (ppm)

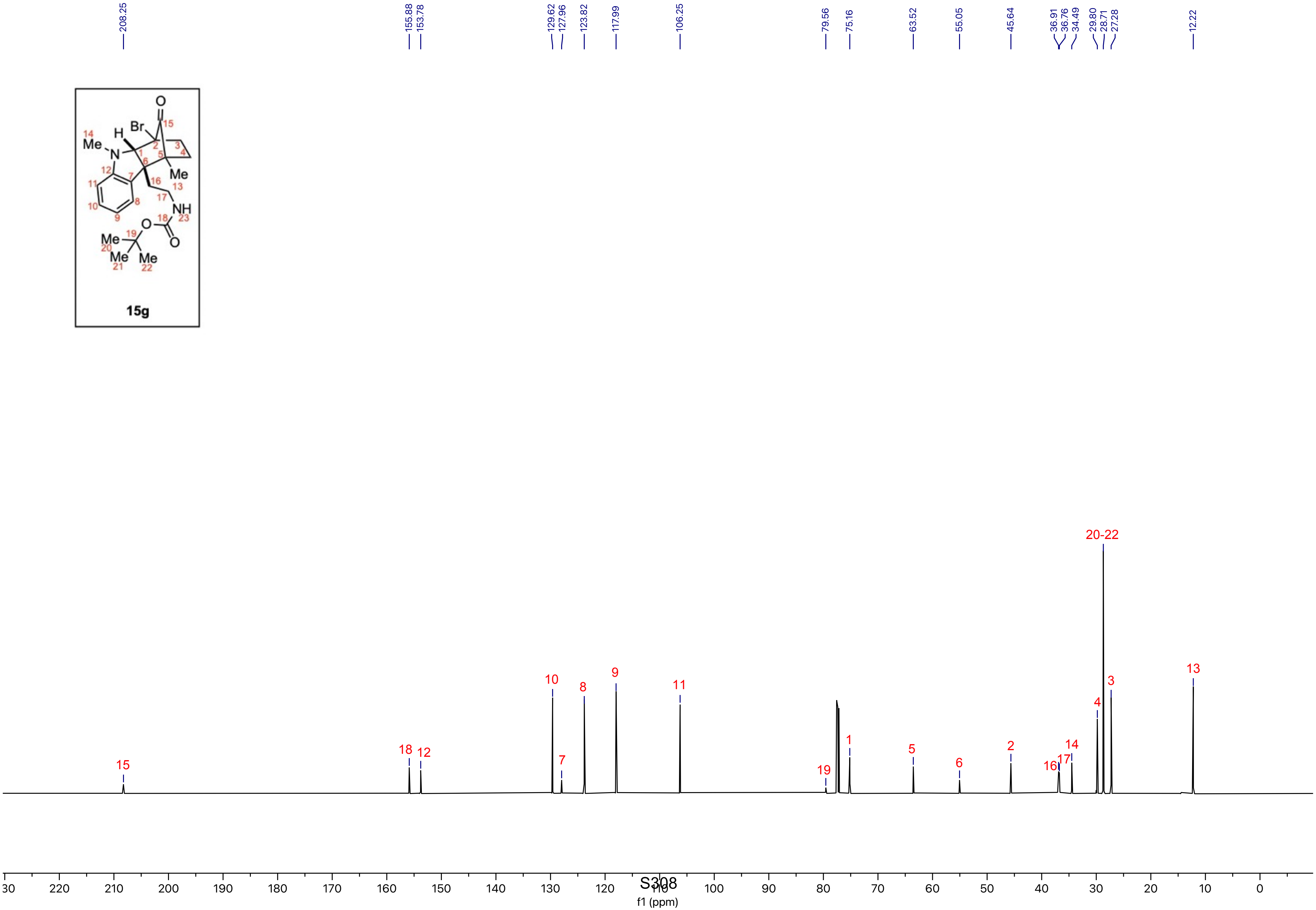
208.25
155.88
153.78
129.62
127.96
123.82
117.99
106.25
79.56
75.16
63.52
55.05
45.64
36.91
36.76
34.49
29.80
28.71
27.28
12.22
15g
f1 (ppm)

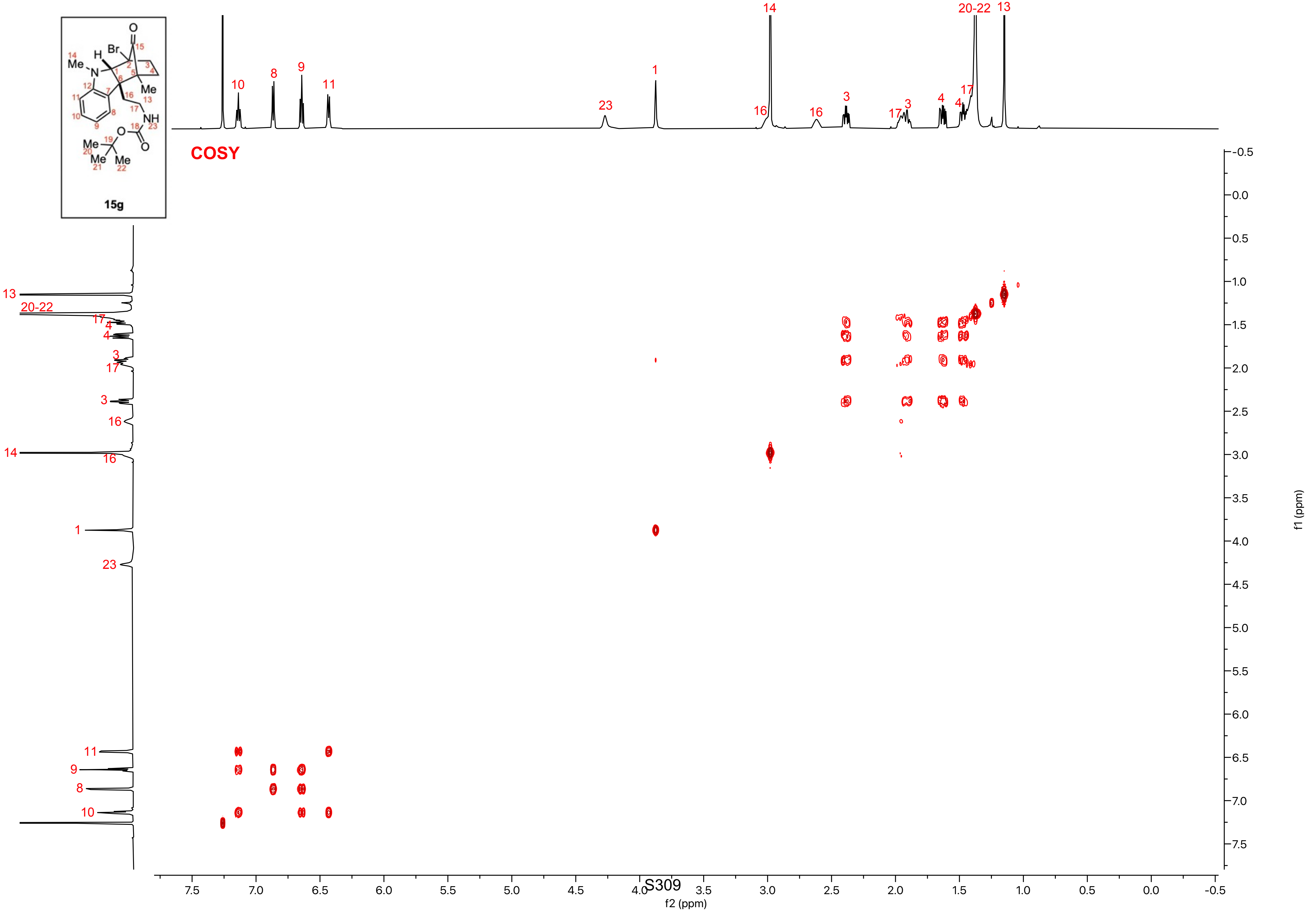
15g
COSY
14
20-22
13
10
8
9
11
23
1
16
3
17
4
f2 (ppm)
f1 (ppm)

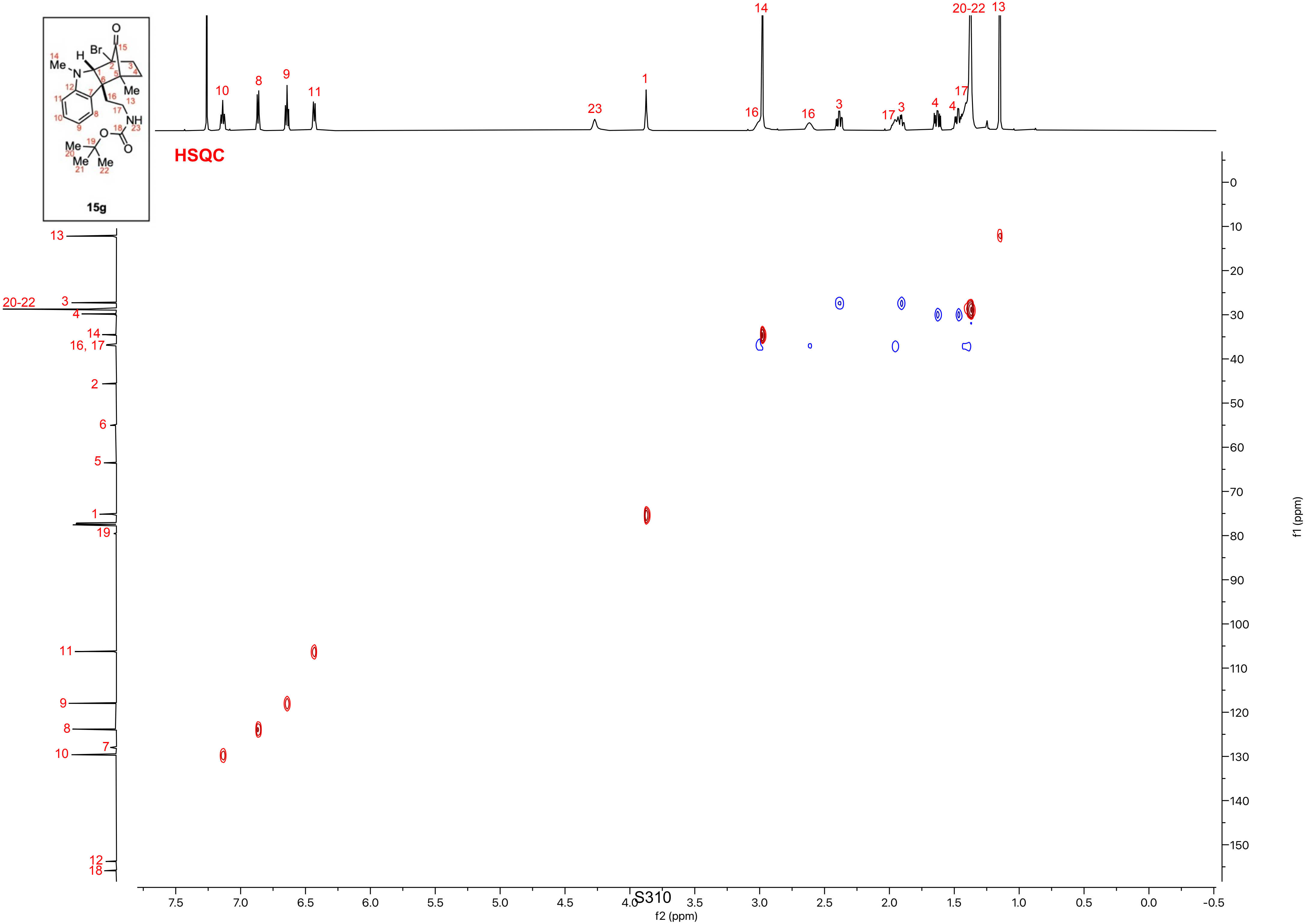

15g
HSQC
f2 (ppm)
f1 (ppm)

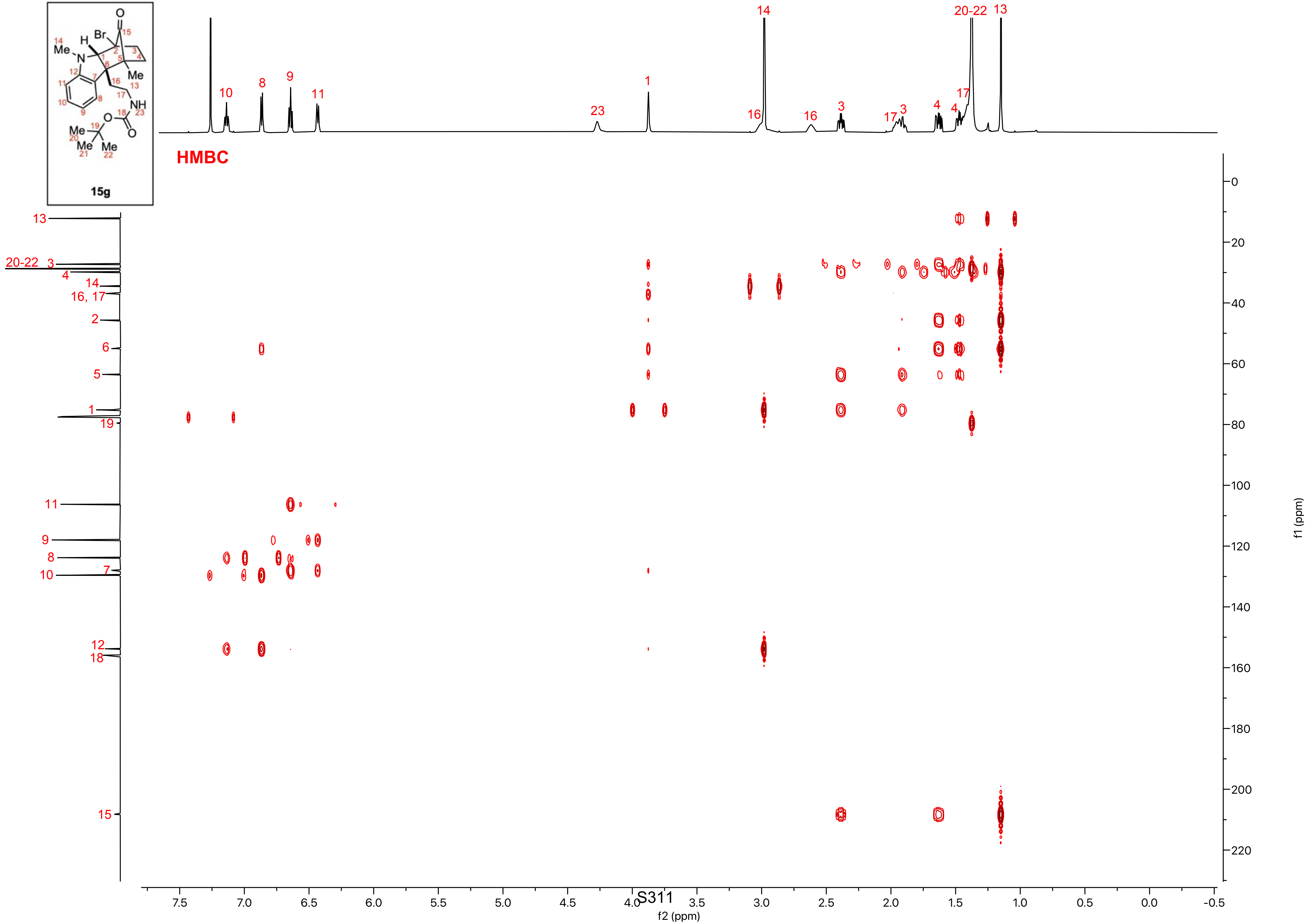

15g
HMBC
f2 (ppm)
f1 (ppm)

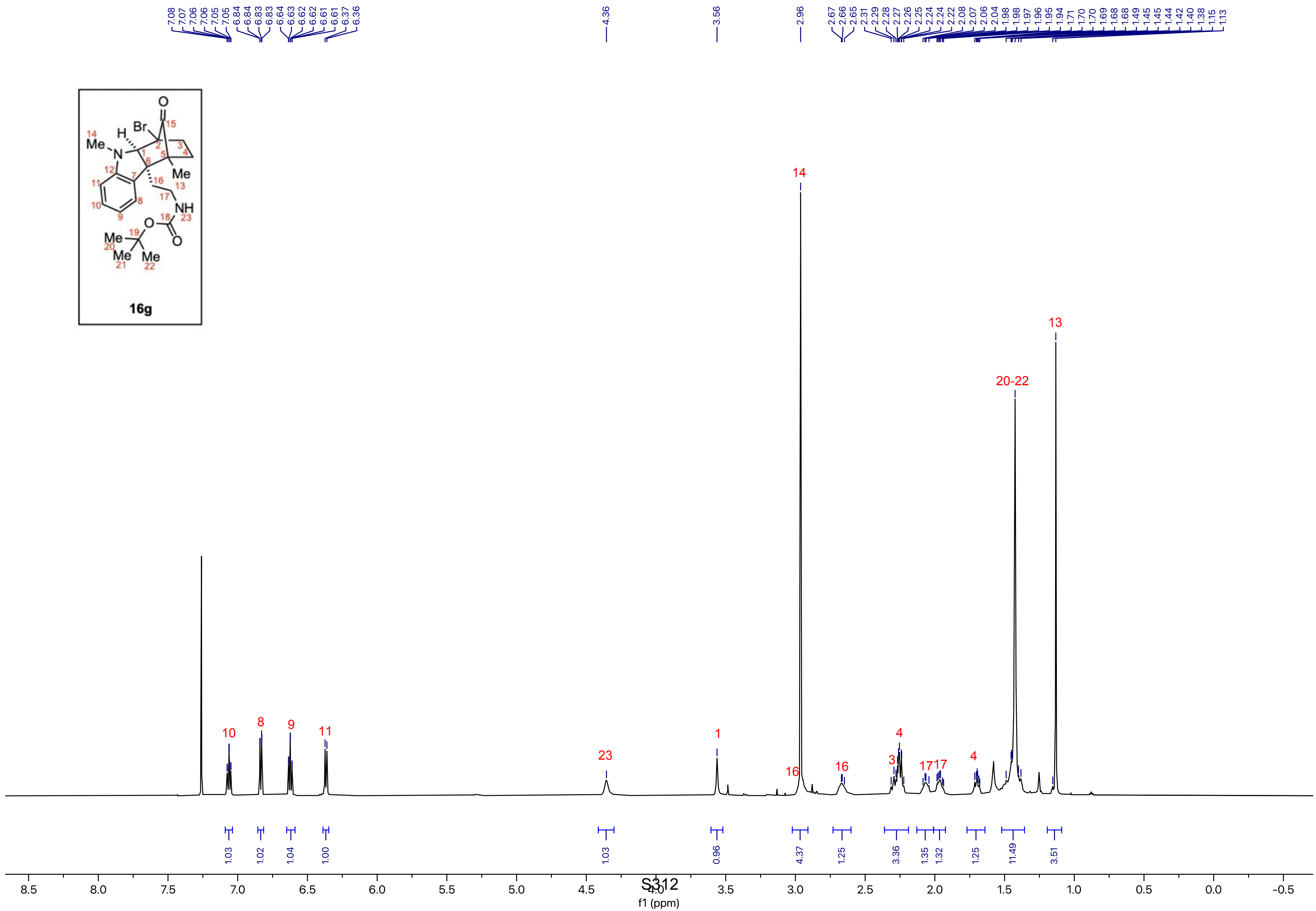

16g
f1 (ppm)

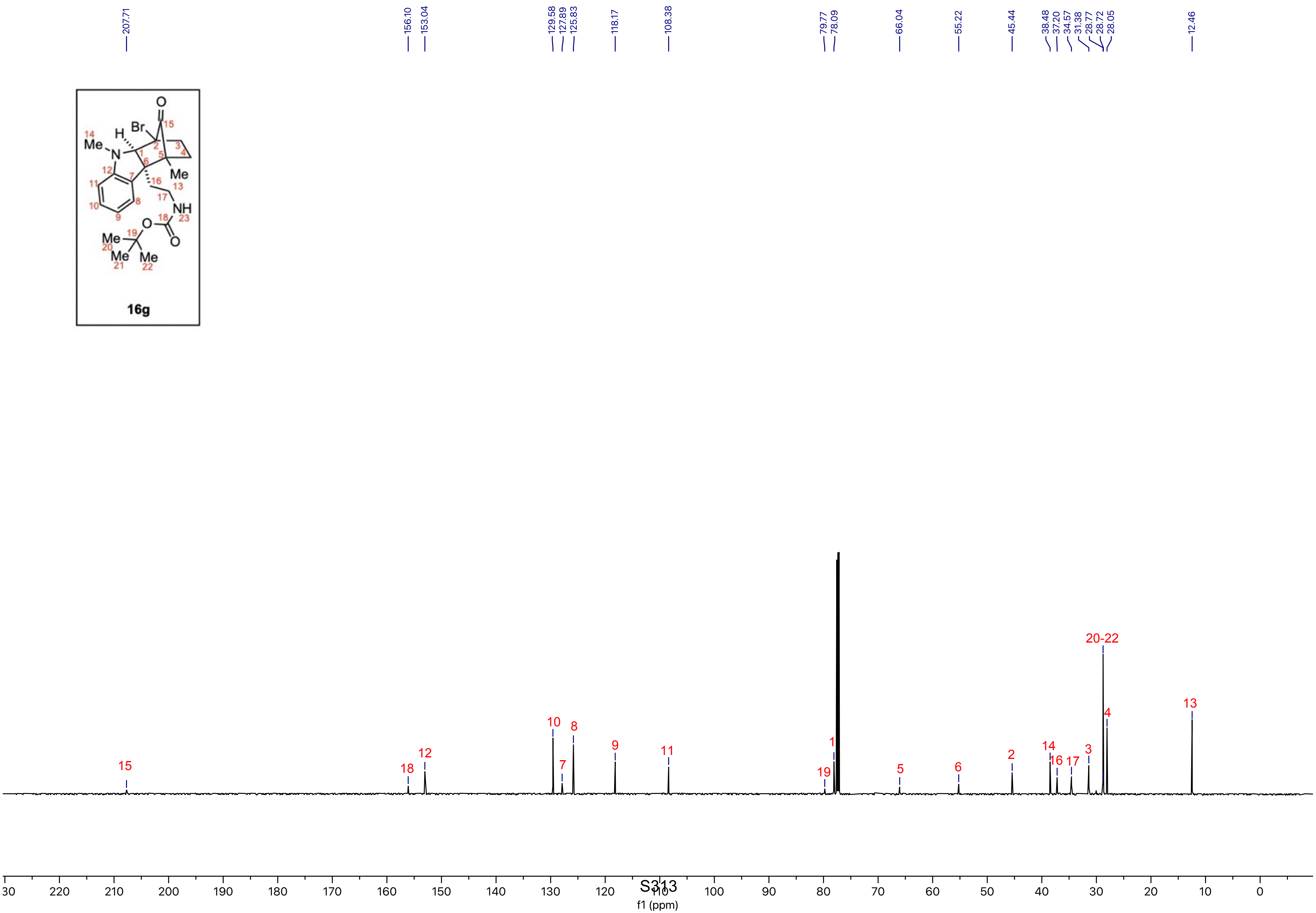
207.71
156.10
153.04
129.58
127.89
125.83
118.17
108.38
79.77
78.09
66.04
55.22
45.44
38.48
37.20
34.57
31.38
28.77
28.72
28.05
12.46
16g
15
18
12
10
7
8
9
11
19
1
5
6
2
14
16
17
3
20-22
4
13
f1 (ppm)

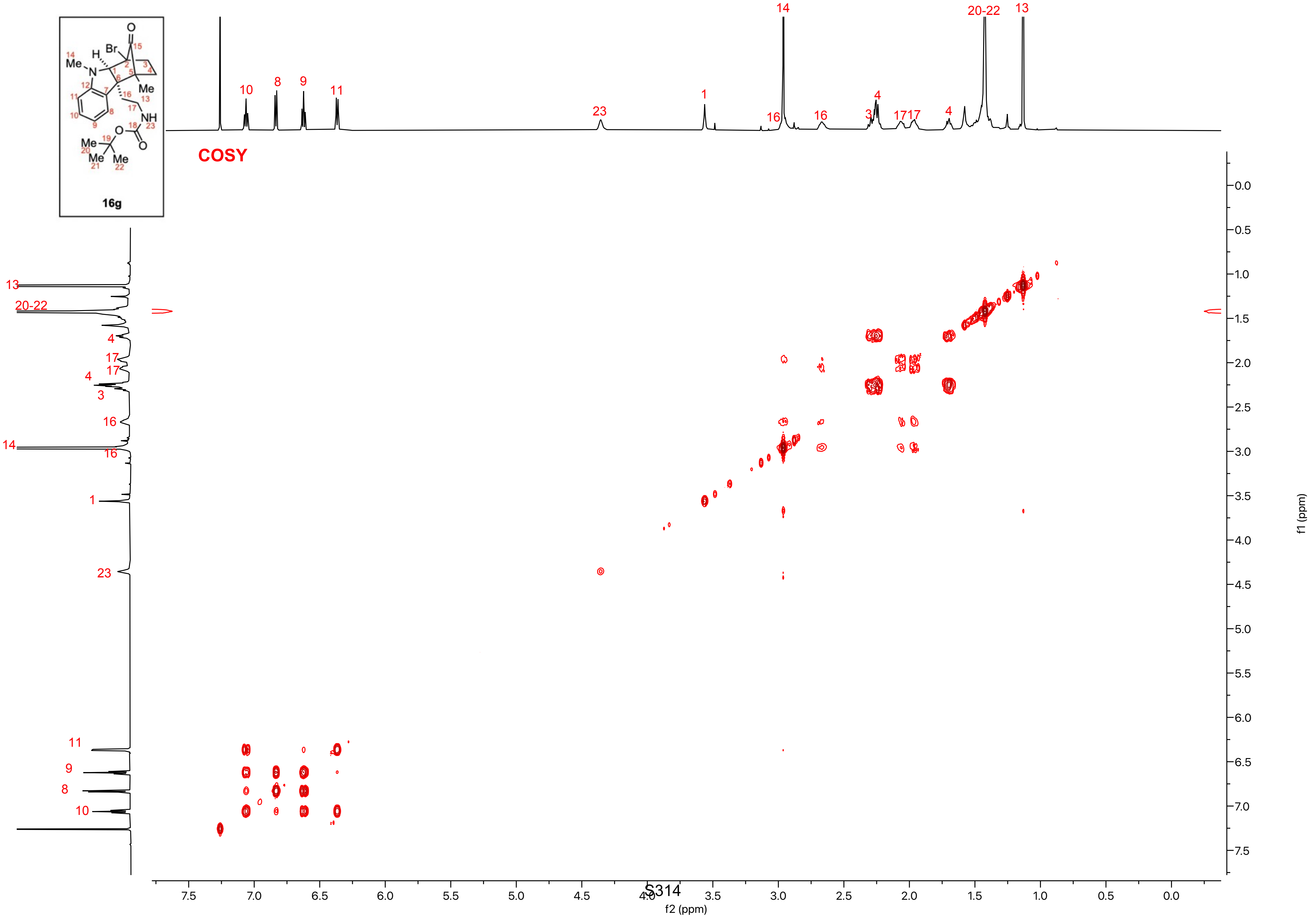
COSY
16g
14
20-22
13
10
8
9
11
23
1
16
16
3
4
17
17
4
f2 (ppm)
f1 (ppm)

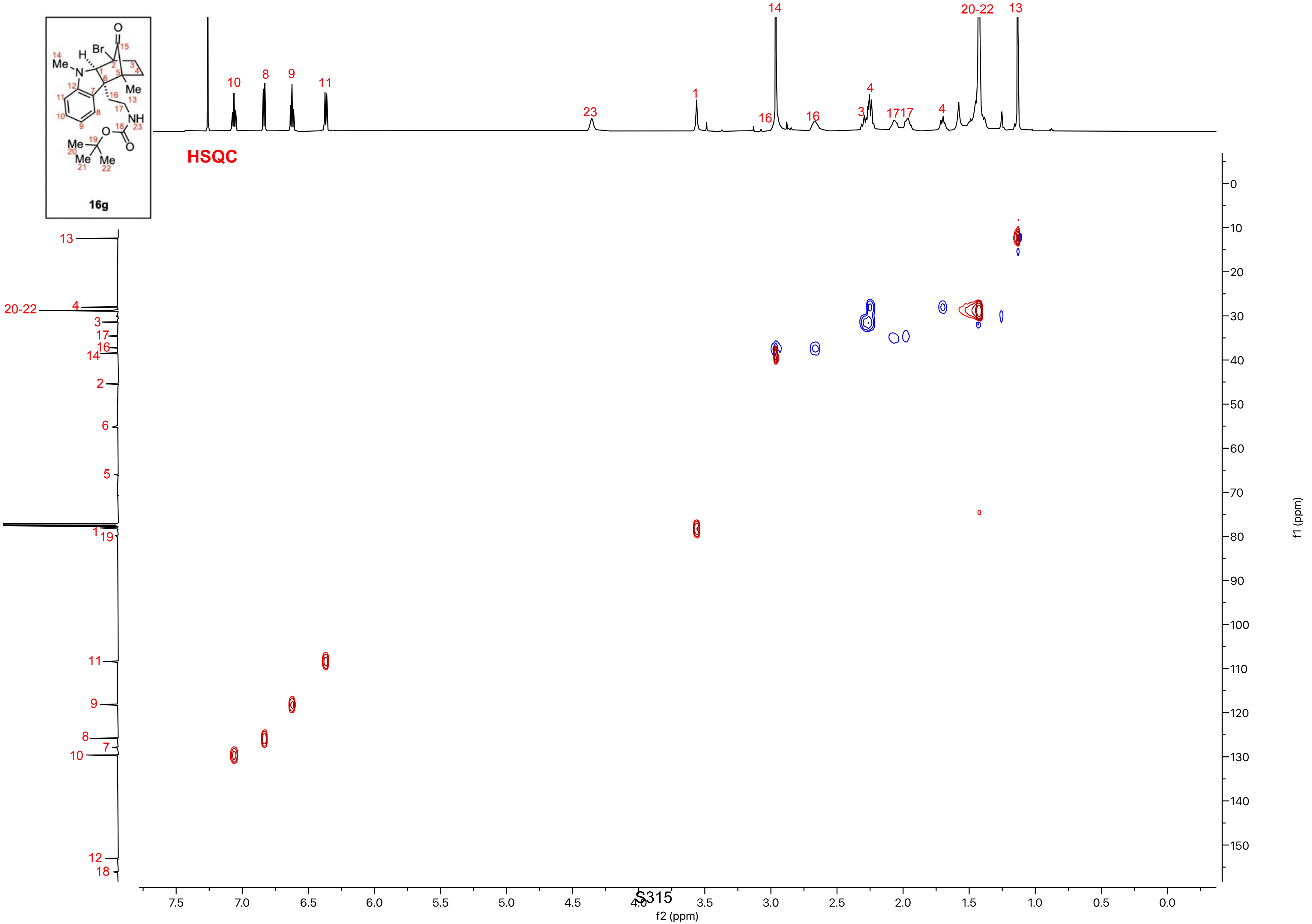

16g
HSQC
f2 (ppm)
f1 (ppm)

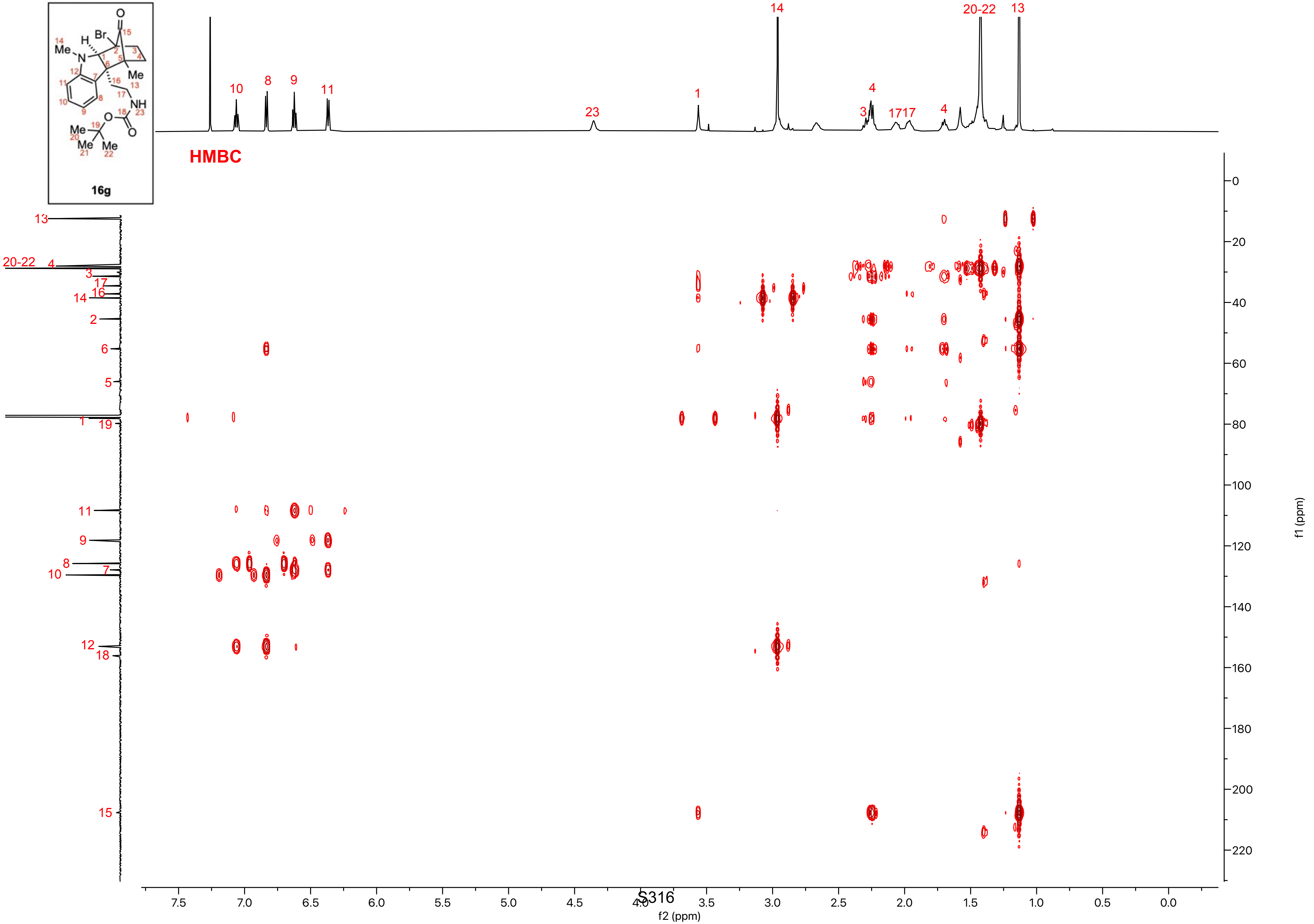
16g
HMBC
f2 (ppm)
f1 (ppm)

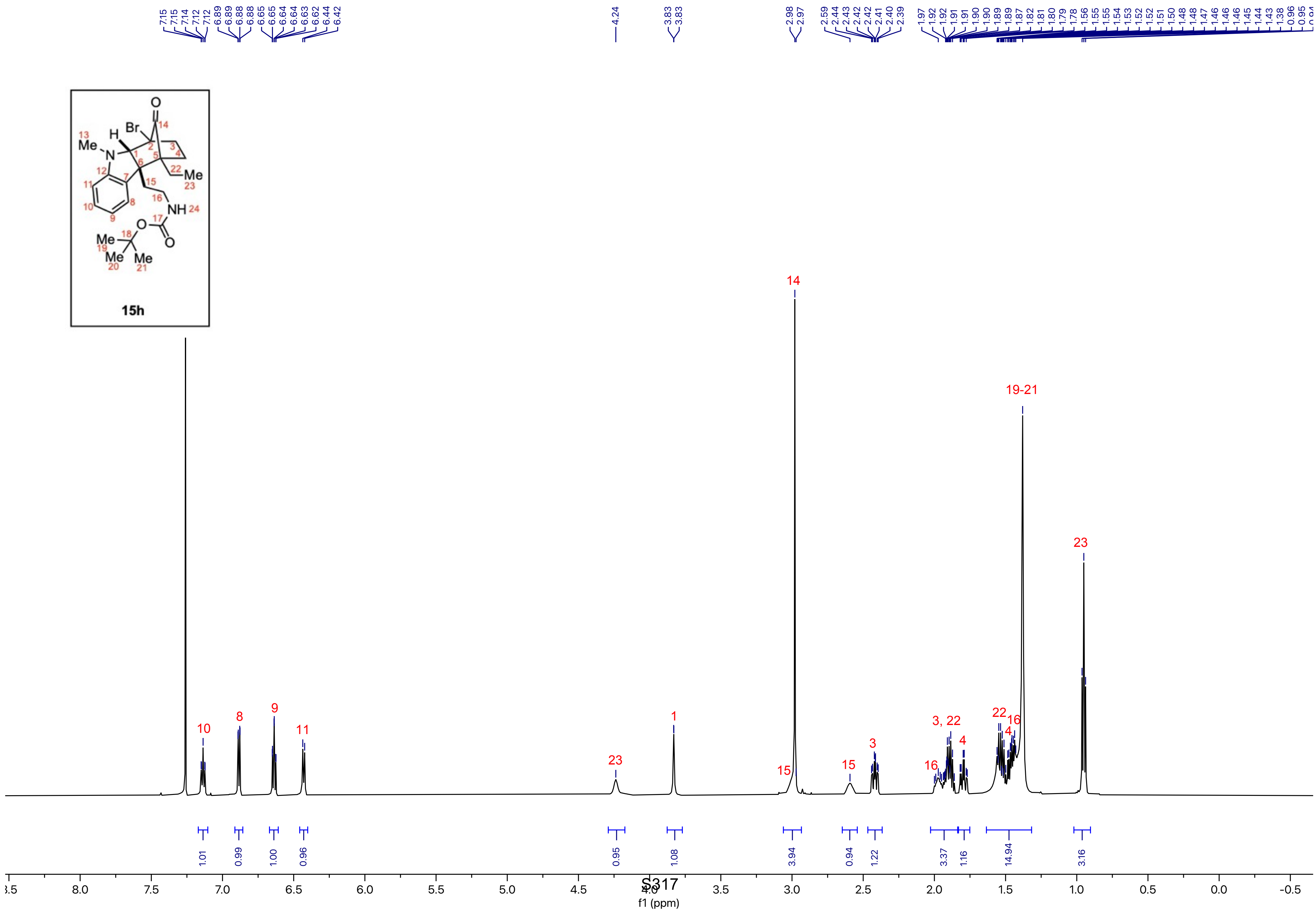

7.15
7.15
7.14
7.12
7.12
6.89
6.89
6.88
6.88
6.65
6.65
6.64
6.64
6.63
6.62
6.44
6.42
4.24
3.83
3.83
2.98
2.97
2.59
2.44
2.43
2.42
2.42
2.41
2.40
2.39
1.97
1.92
1.92
1.91
1.91
1.90
1.90
1.89
1.89
1.87
1.82
1.81
1.80
1.79
1.78
1.56
1.55
1.55
1.54
1.53
1.52
1.52
1.51
1.50
1.48
1.48
1.47
1.46
1.46
1.45
1.44
1.43
1.38
0.96
O
Br
H
Me
N
Me
NH
O
O
Me
Me
Me
15h
14
19-21
23
10
8
9
11
23
1
15
15
3
16
3, 22
4
22
16
4
1.01
0.99
1.00
0.96
0.95
1.08
3.94
0.94
1.22
3.37
1.16
14.94
3.16
8.0
7.5
7.0
6.5
6.0
5.5
5.0
4.5
4.0
3.5
3.0
2.5
2.0
1.5
1.0
0.5
0.0
-0.5
f1 (ppm)

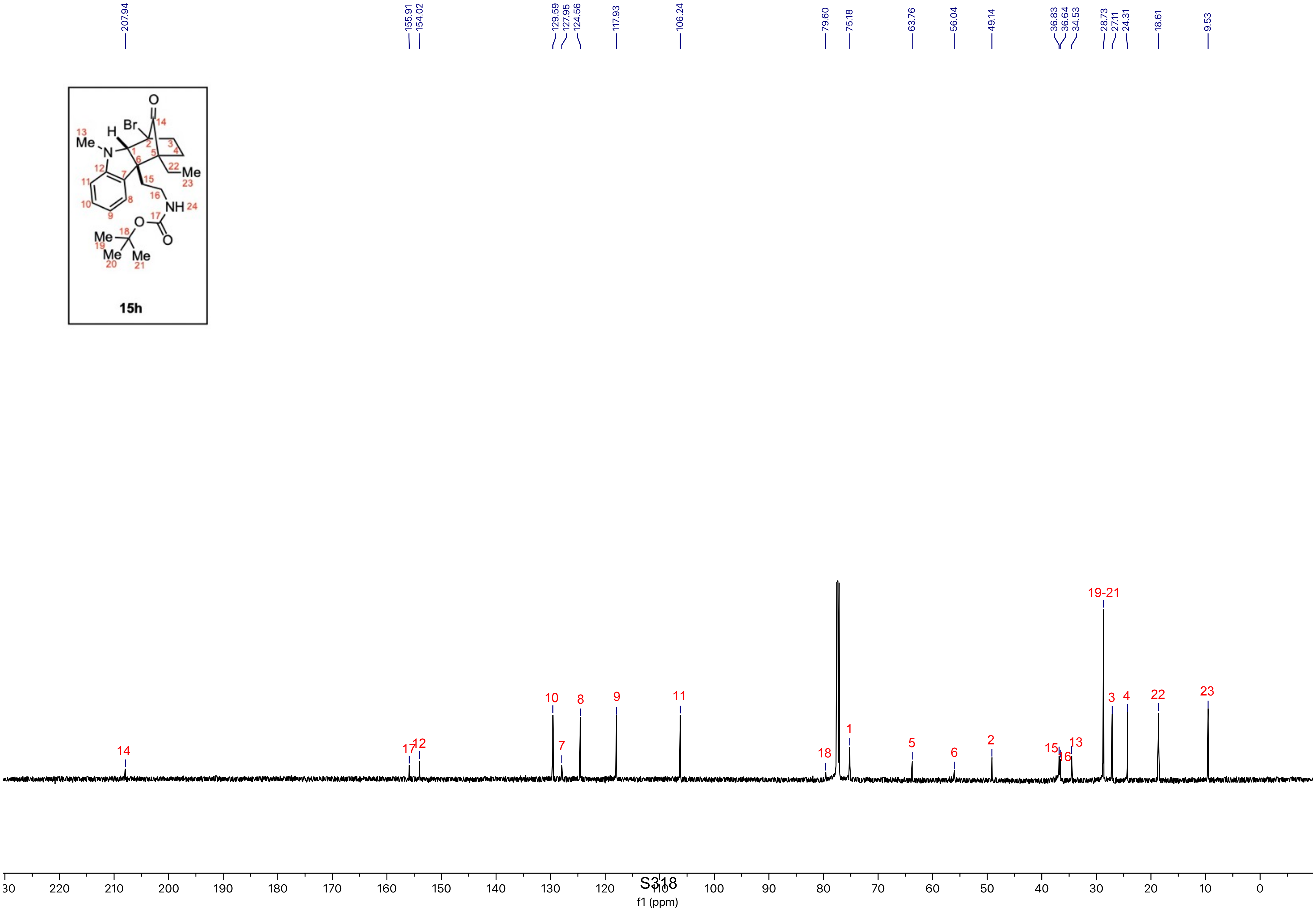

207.94
155.91
154.02
129.59
127.95
124.56
117.93
106.24
79.60
75.18
63.76
56.04
49.14
36.83
36.64
34.53
28.73
27.11
24.31
18.61
9.53
15h
f1 (ppm)

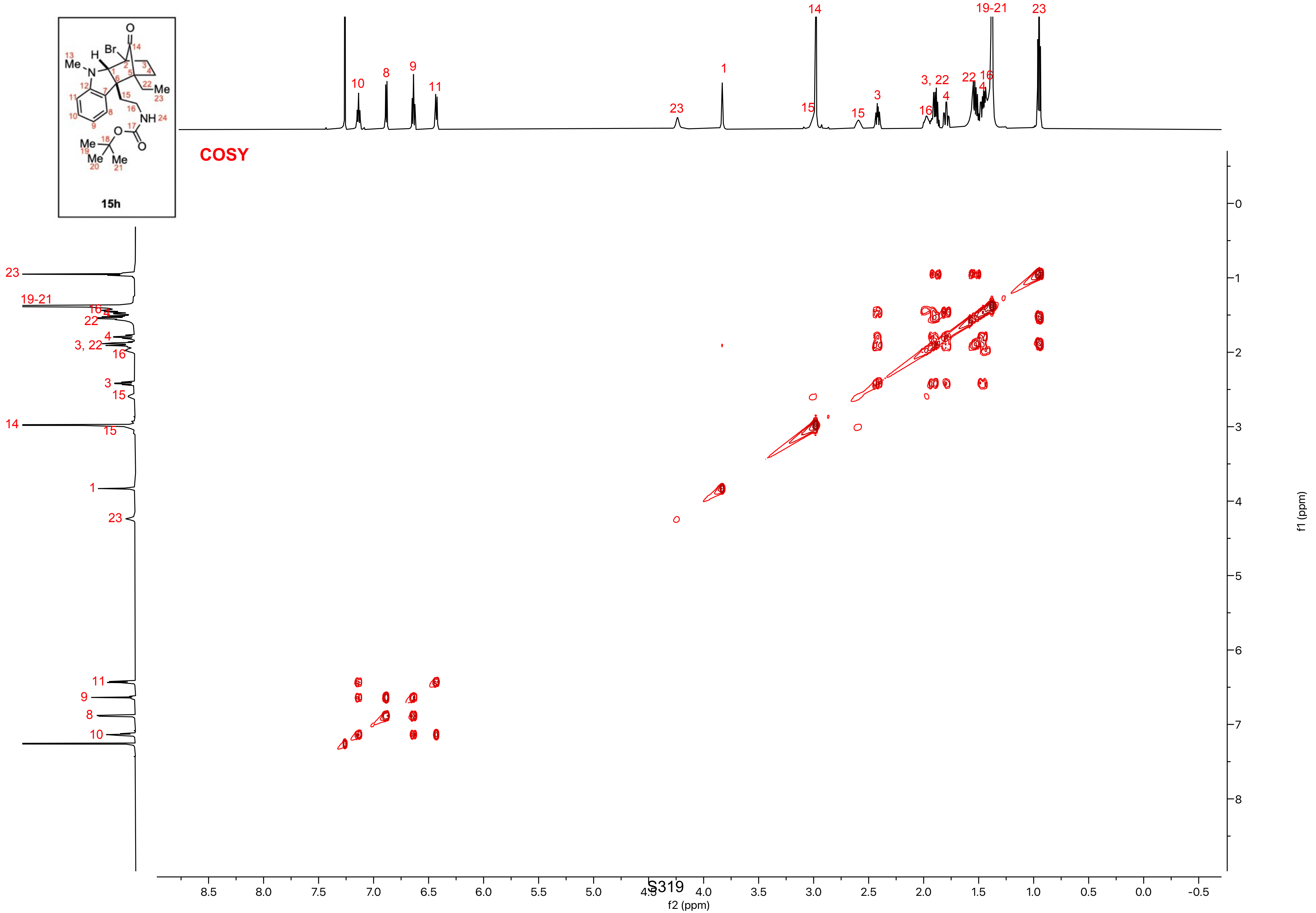

15h
COSY
f2 (ppm)
f1 (ppm)

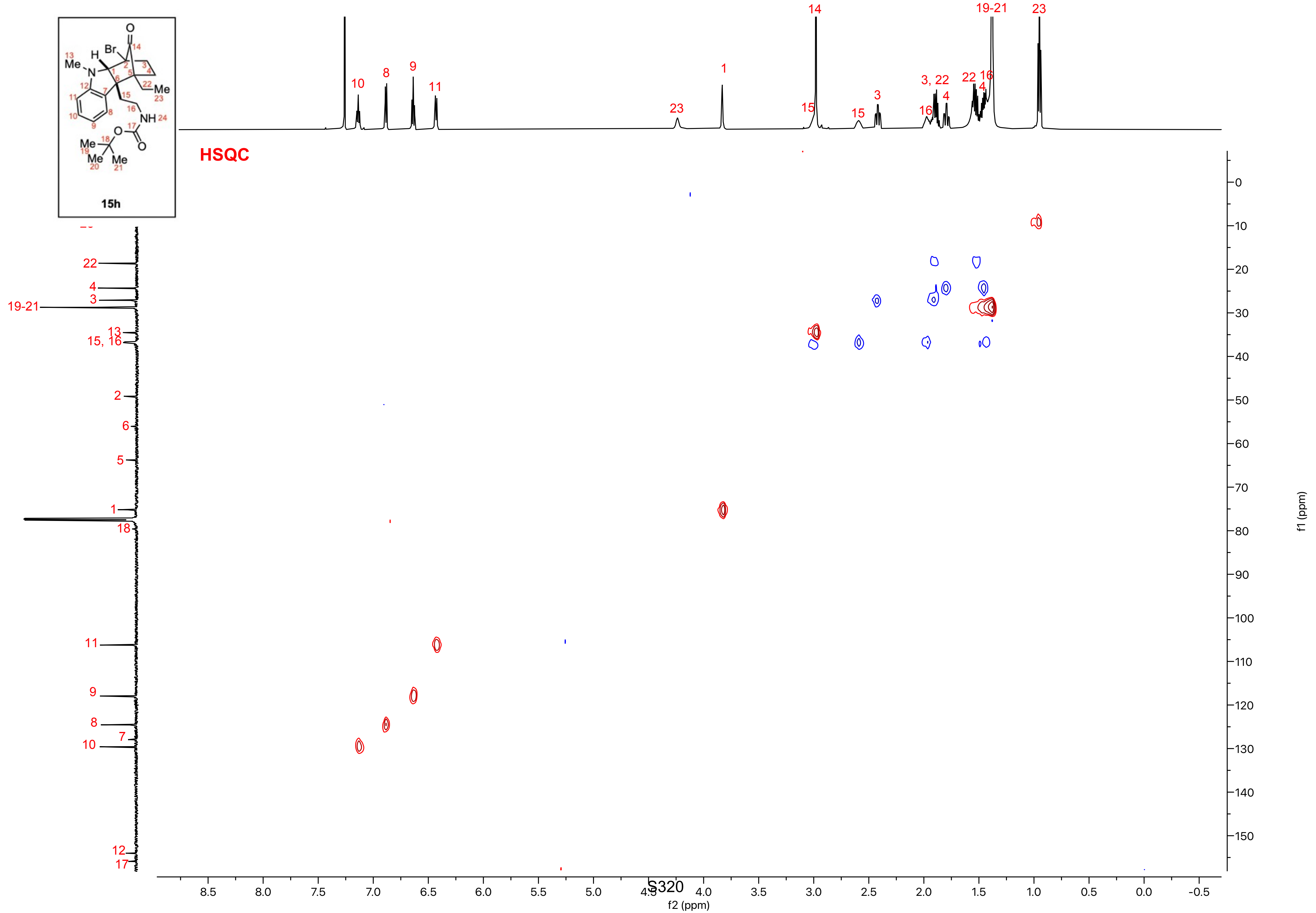

HSQC
15h
10
8
9
11
23
1
14
15
15
3
3, 22
4
16
22
16
4
19-21
23
22
4
3
19-21
13
15, 16
2
6
5
1
18
11
9
8
7
10
12
17
f2 (ppm)
f1 (ppm)

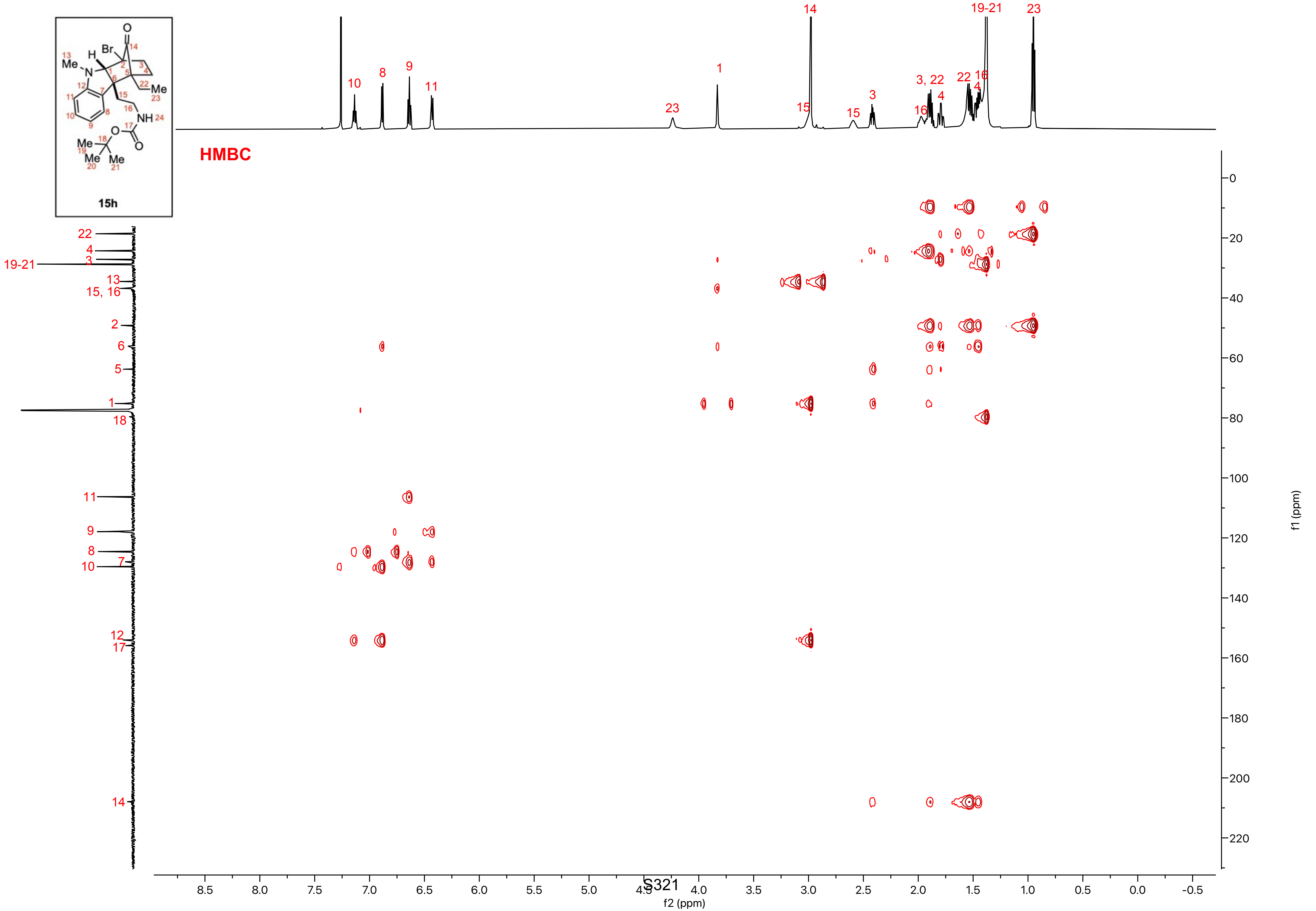
HMBC
f2 (ppm)
f1 (ppm)
15h

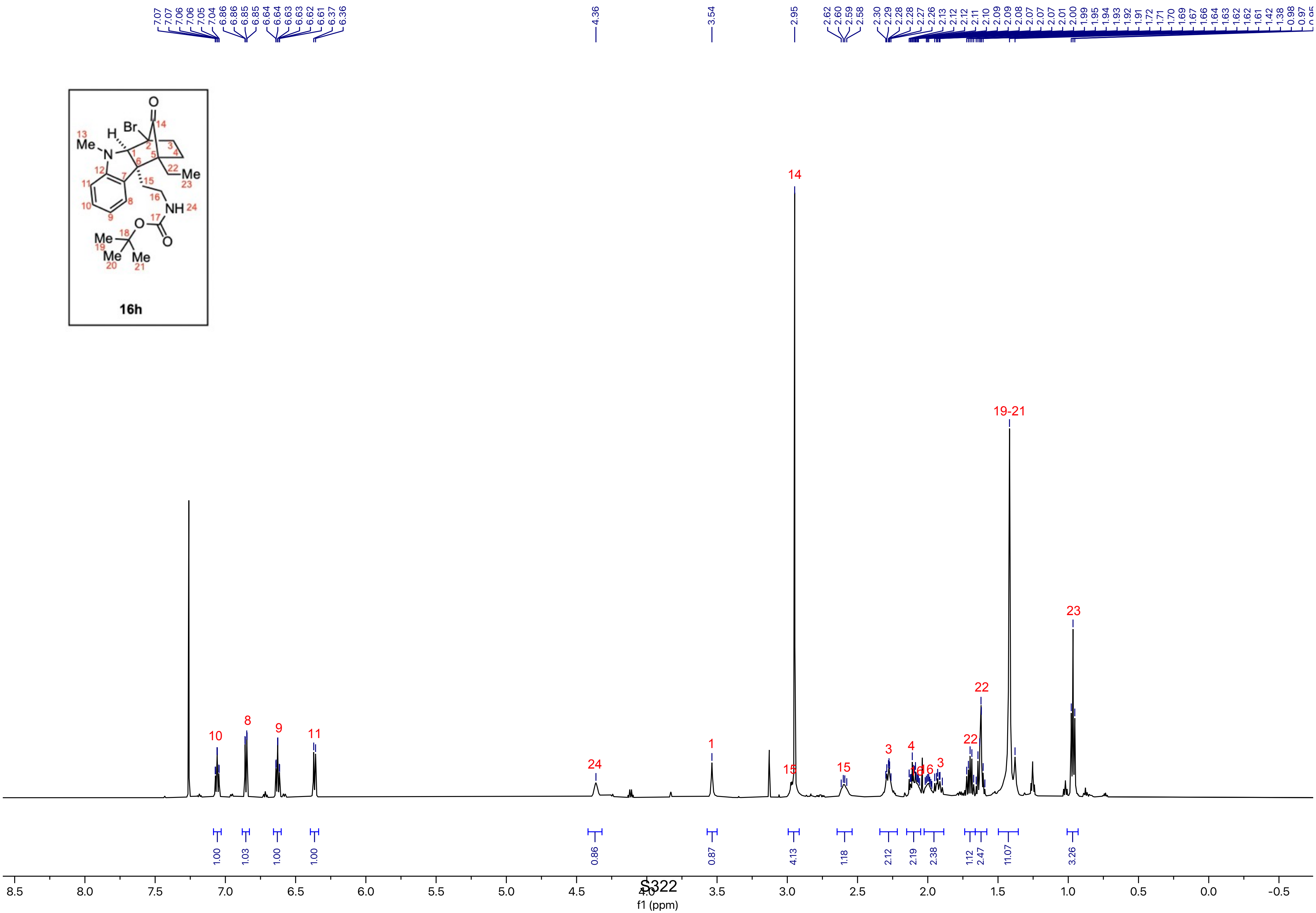
16h
f1 (ppm)

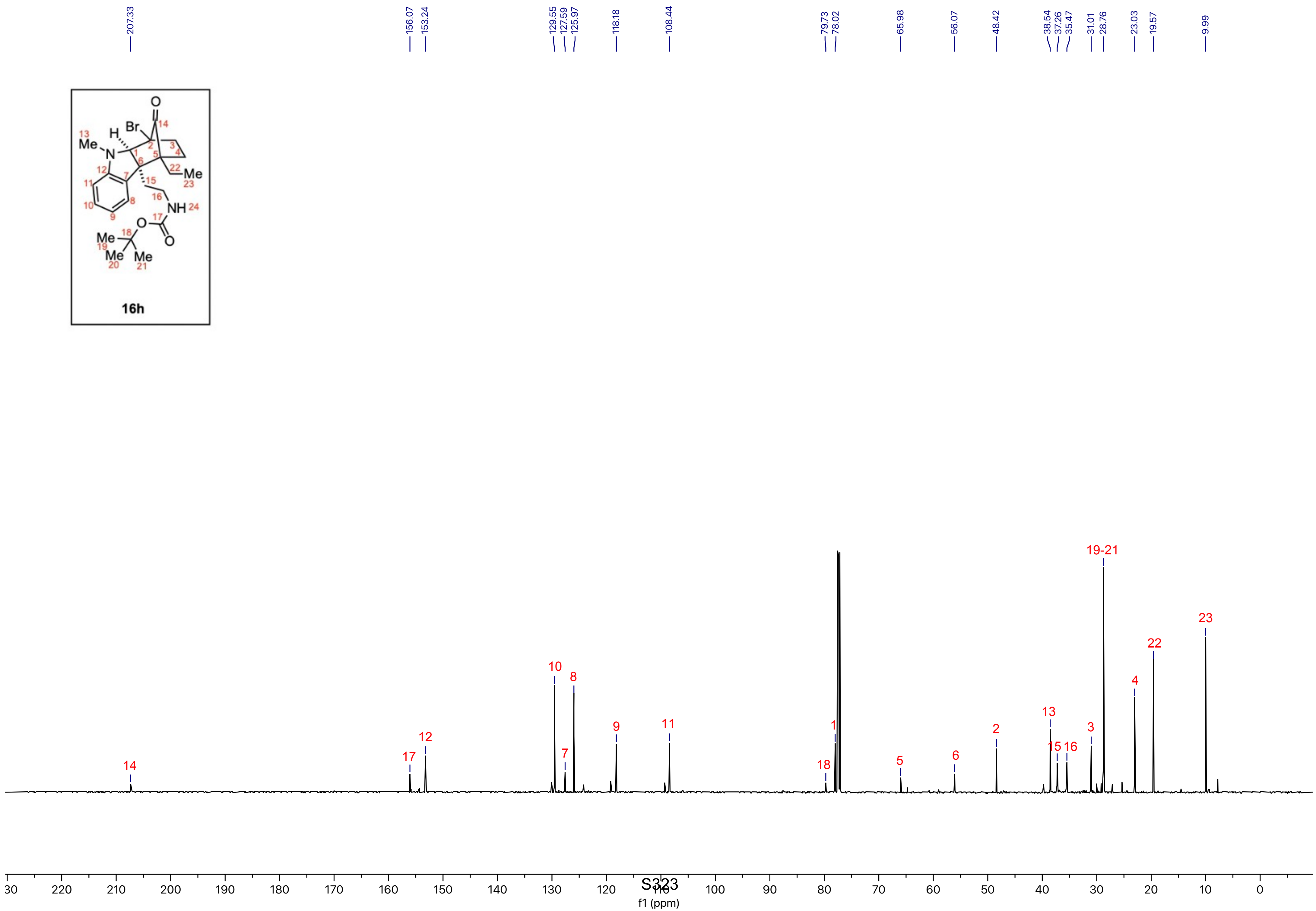
207.33
156.07
153.24
129.55
127.59
125.97
118.18
108.44
79.73
78.02
65.98
56.07
48.42
38.54
37.26
35.47
31.01
28.76
23.03
19.57
9.99
16h
14
17
12
10
7
8
9
11
18
1
5
6
2
13
15
16
3
19-21
4
22
23
f1 (ppm)

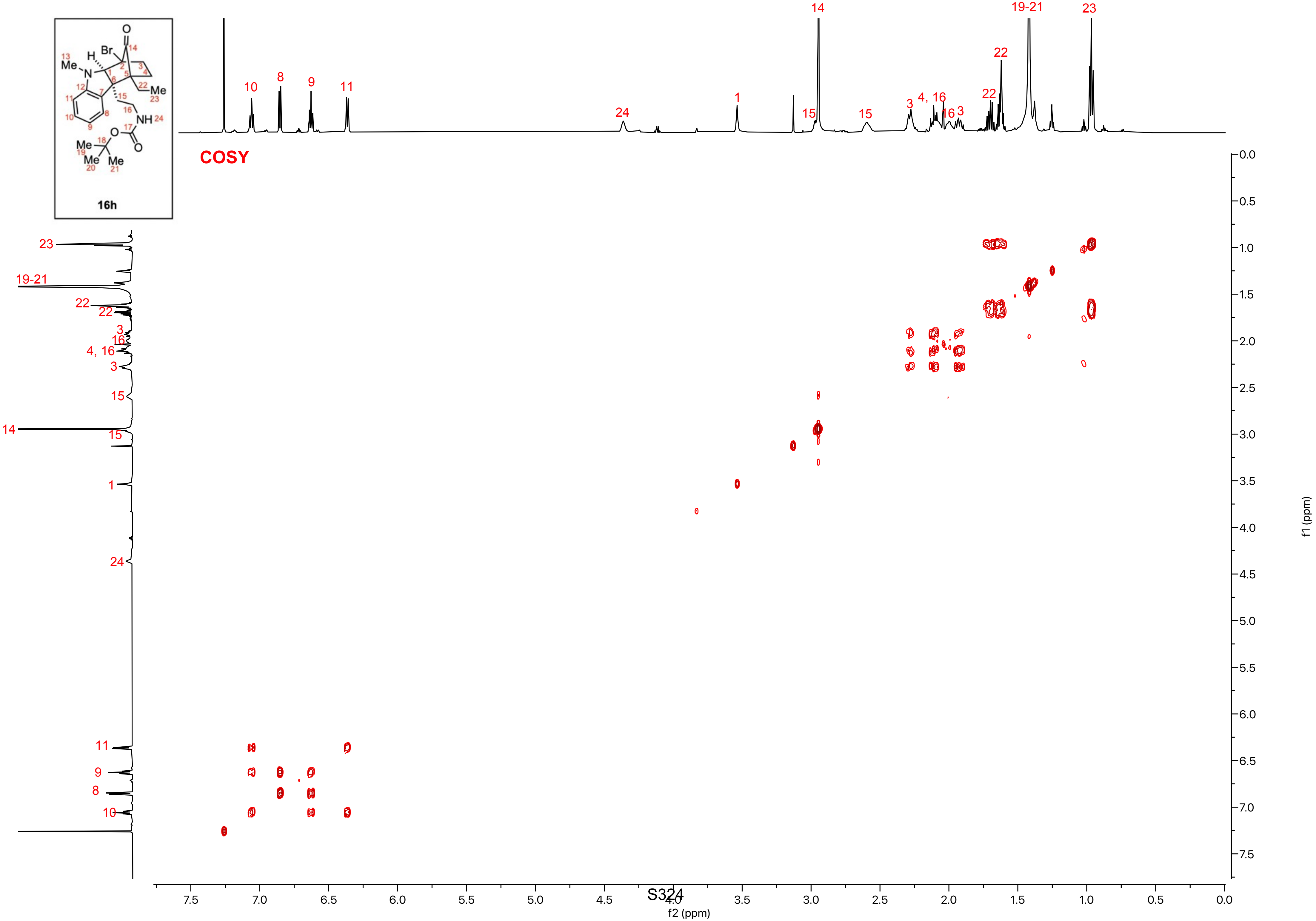

16h
COSY
14
19-21
23
22
10
8
9
11
24
1
15
15
3
4, 16
16
3
22
f2 (ppm)
f1 (ppm)

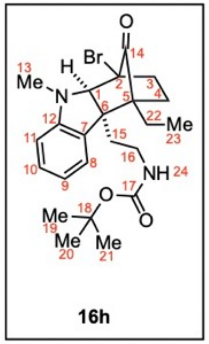
Br
H
Me
N
O
Me
NH
O
O
Me
Me
Me
16h

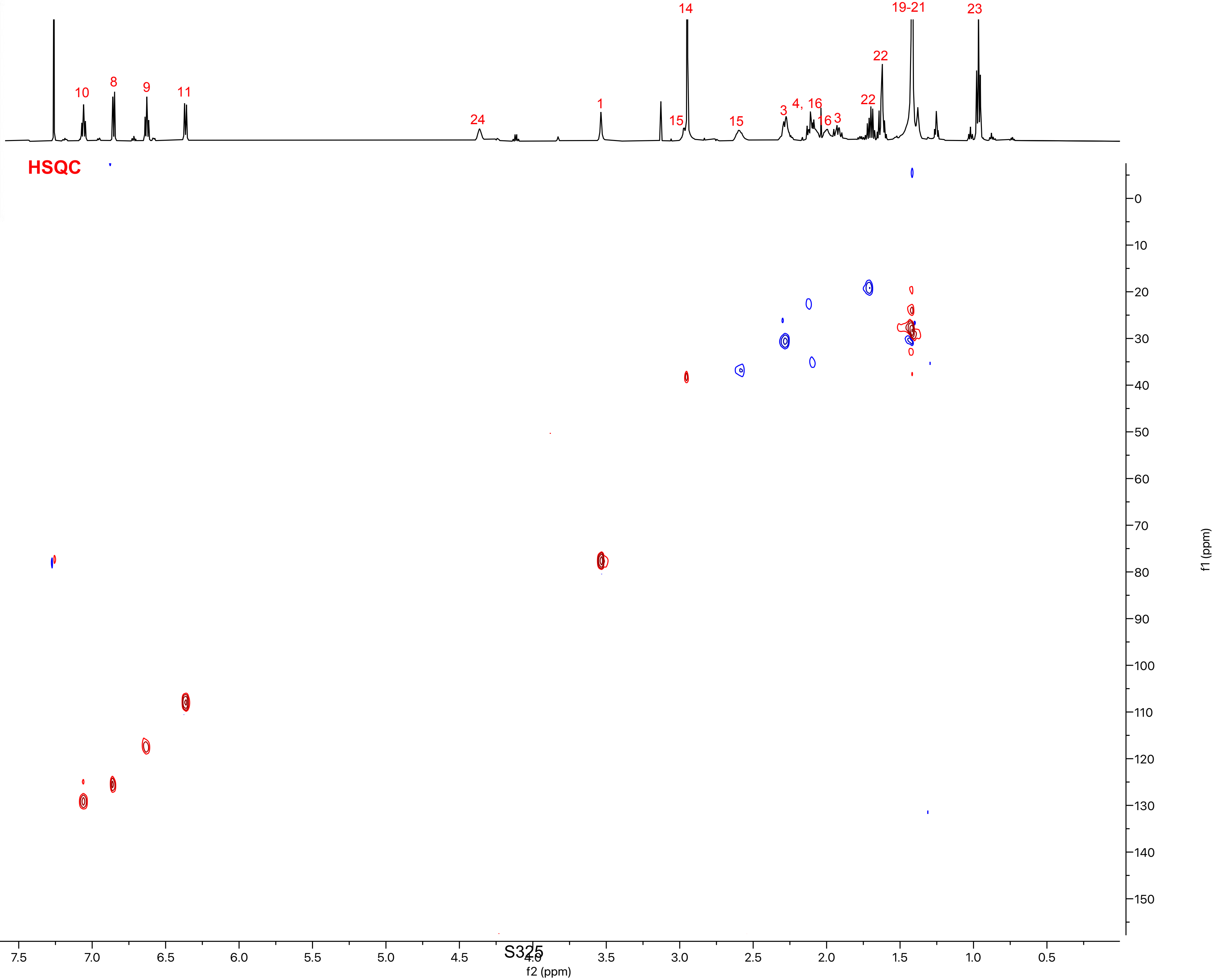
HSQC
f2 (ppm)
f1 (ppm)

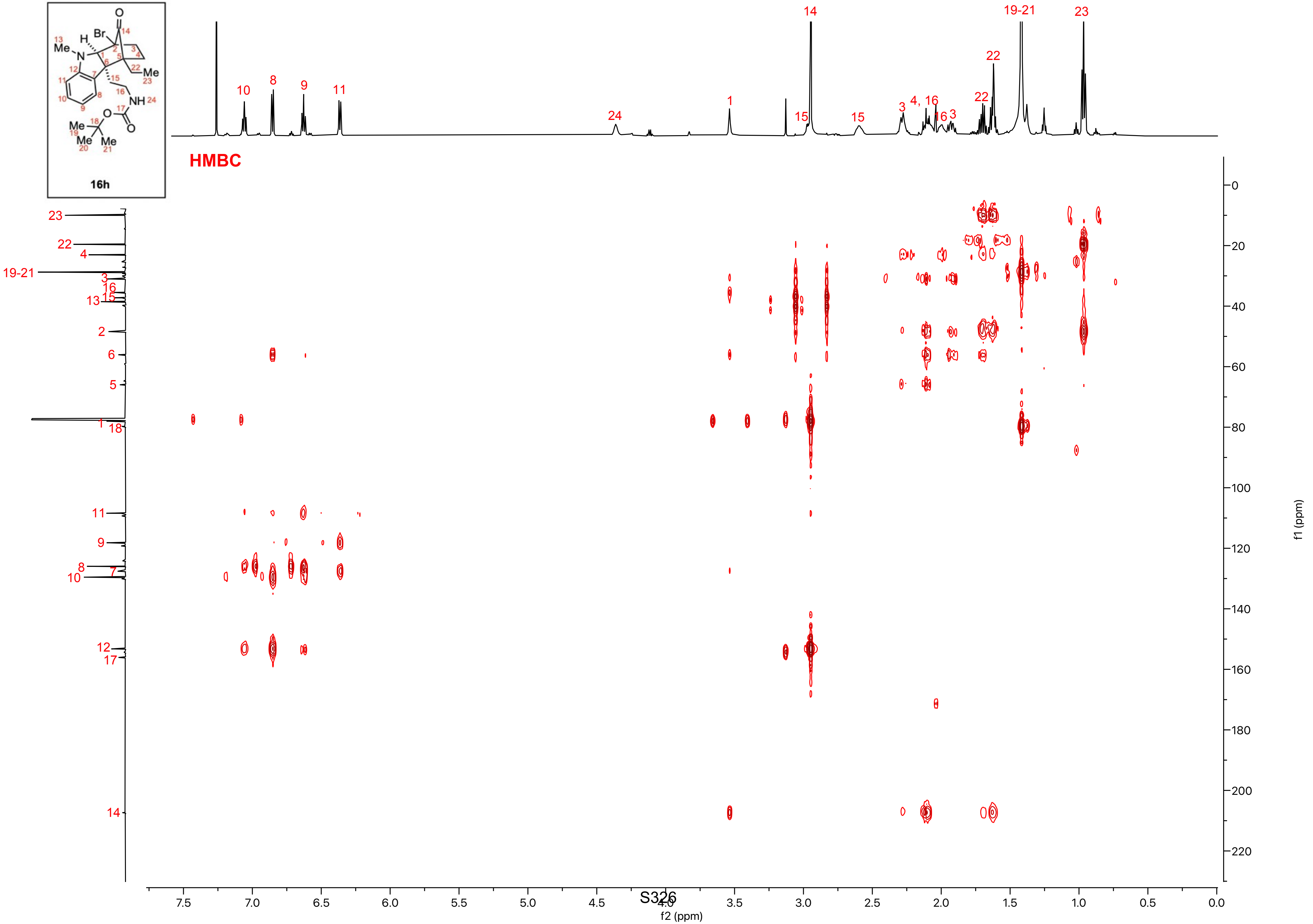
16h
HMBC
f2 (ppm)
f1 (ppm)

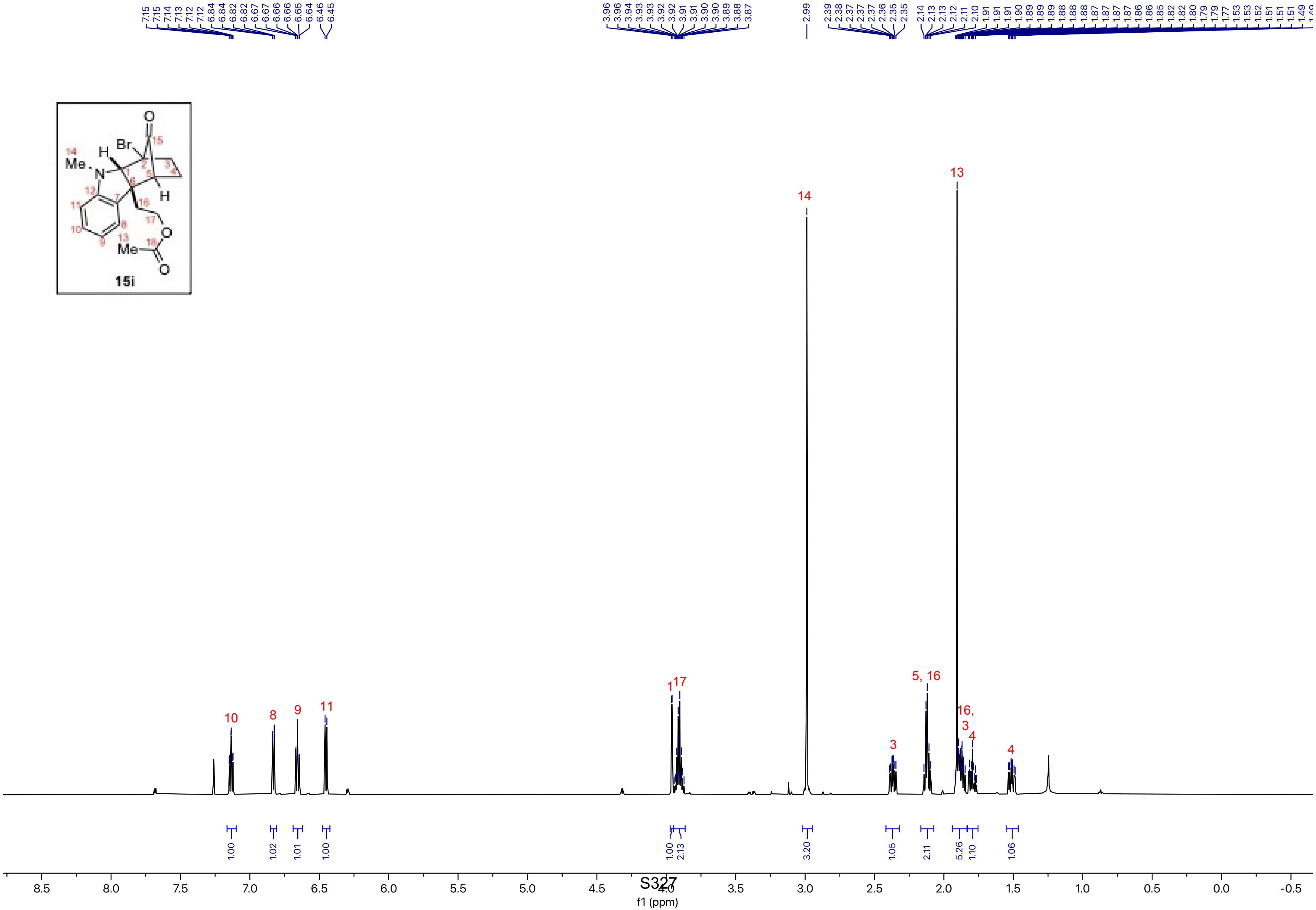
15i
14
13
10
8
9
11
1
17
5, 16
16,
3
4
3
4
f1 (ppm)

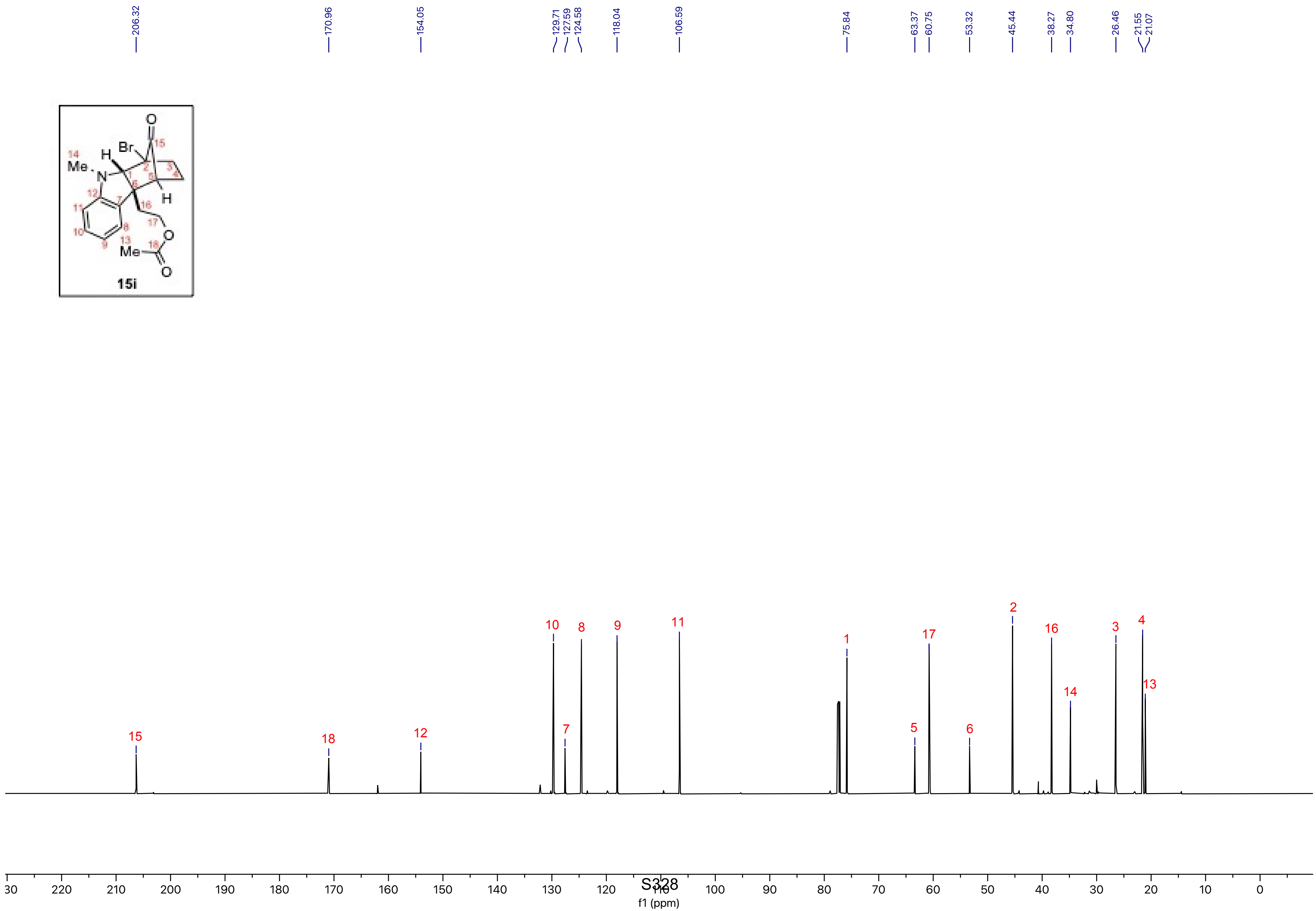
206.32
170.96
154.05
129.71
127.59
124.58
118.04
106.59
75.84
63.37
60.75
53.32
45.44
38.27
34.80
26.46
21.55
21.07
15i
f1 (ppm)

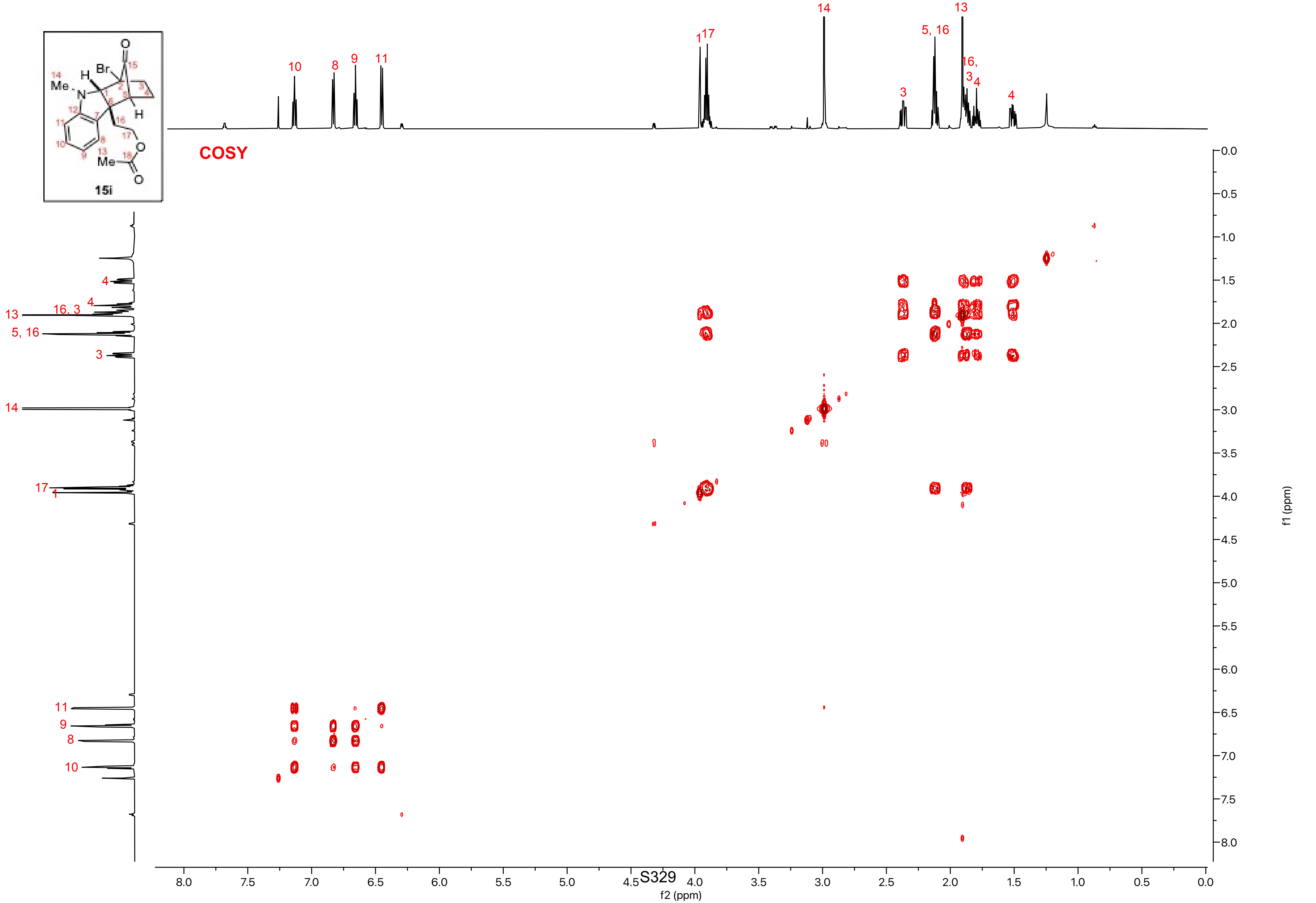

15i
COSY
14
13
5, 16
16,
3
4
1
17
10
8
9
11
f2 (ppm)
f1 (ppm)

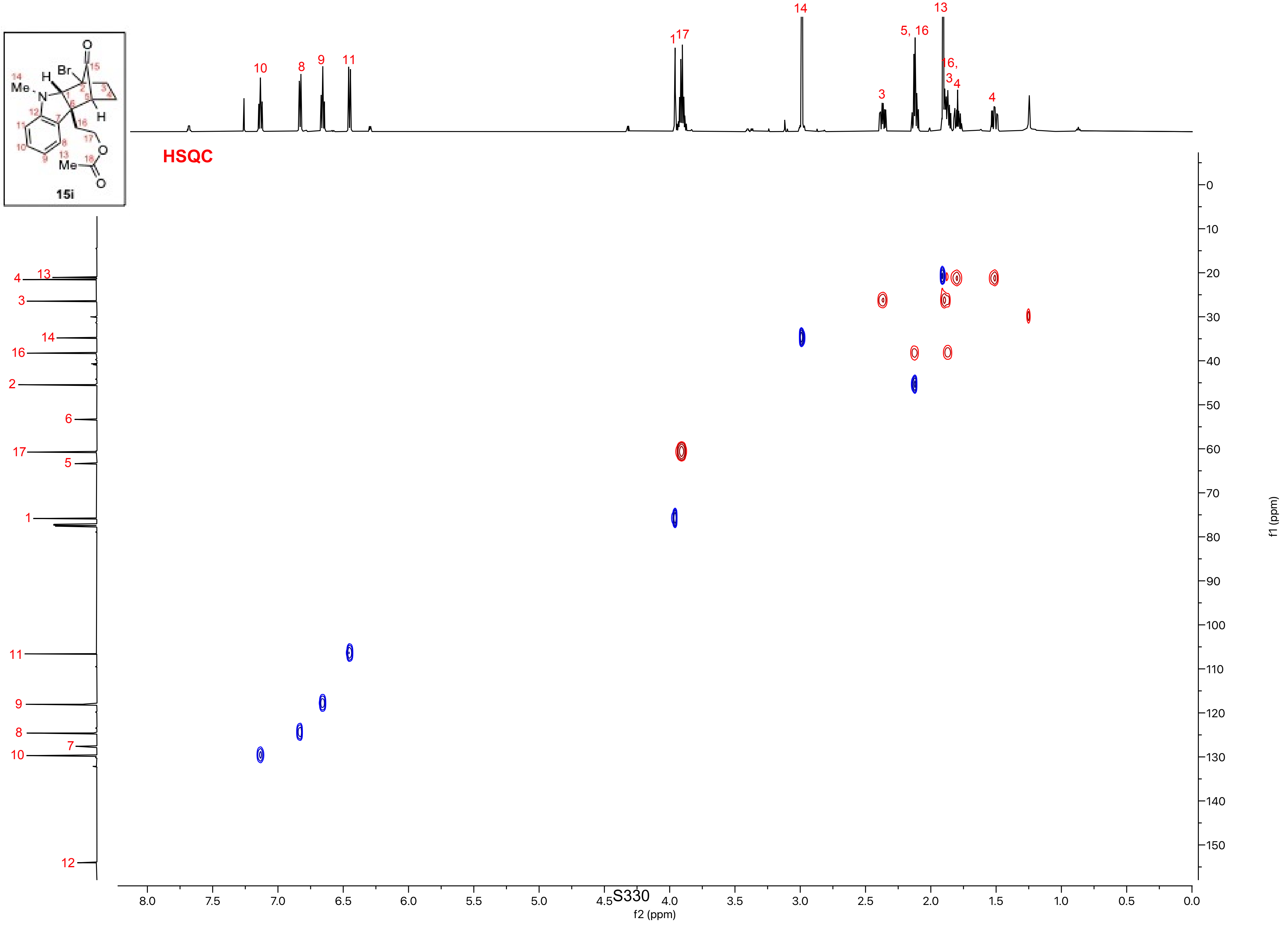
HSQC
15i
f2 (ppm)
f1 (ppm)

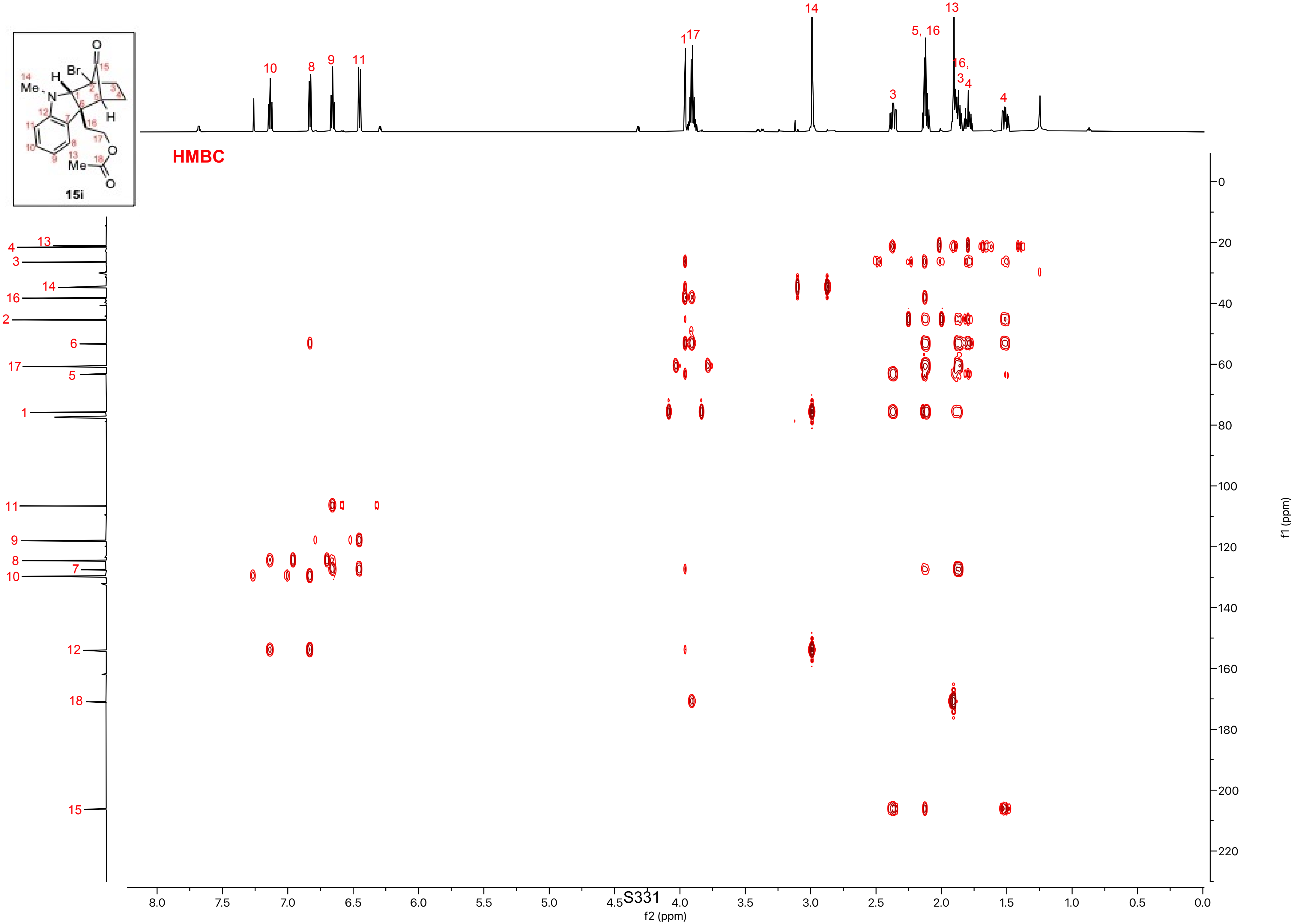
HMBC
15i
f2 (ppm)
f1 (ppm)

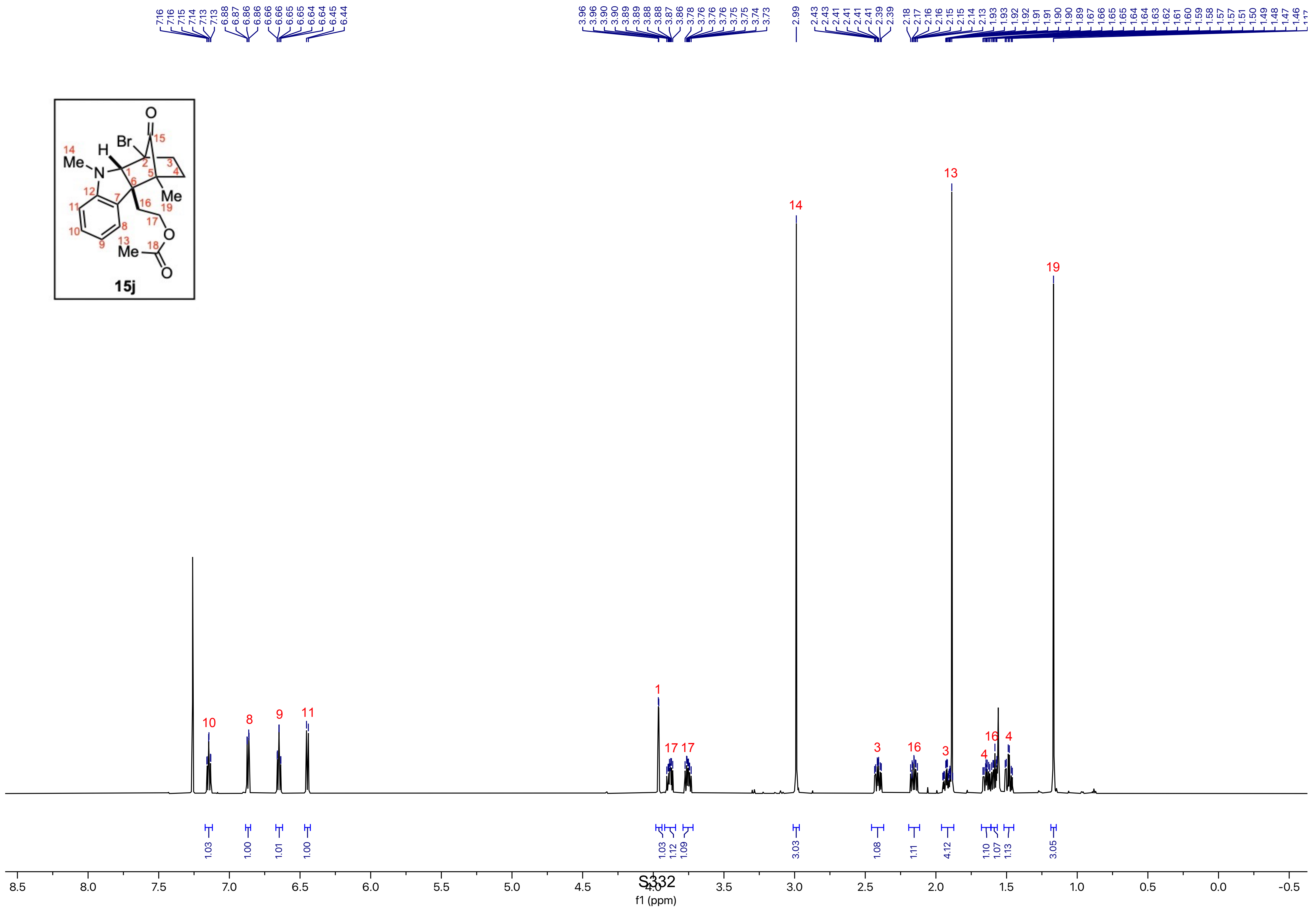
15j
Br
H
Me
N
O
Me
Me
O
O
7.16
7.16
7.15
7.14
7.13
7.13
6.88
6.87
6.86
6.86
6.66
6.66
6.65
6.65
6.64
6.64
6.45
6.44
3.96
3.96
3.90
3.89
3.89
3.88
3.88
3.87
3.86
3.78
3.76
3.76
3.76
3.75
3.74
3.73
2.99
2.43
2.43
2.41
2.41
2.41
2.39
2.39
2.18
2.17
2.16
2.16
2.15
2.15
2.14
2.13
1.93
1.93
1.92
1.91
1.91
1.90
1.90
1.89
1.67
1.66
1.65
1.65
1.64
1.64
1.63
1.62
1.61
1.60
1.59
1.58
1.57
1.57
1.51
1.50
1.49
1.48
1.47
1.46
1.17
f1 (ppm)

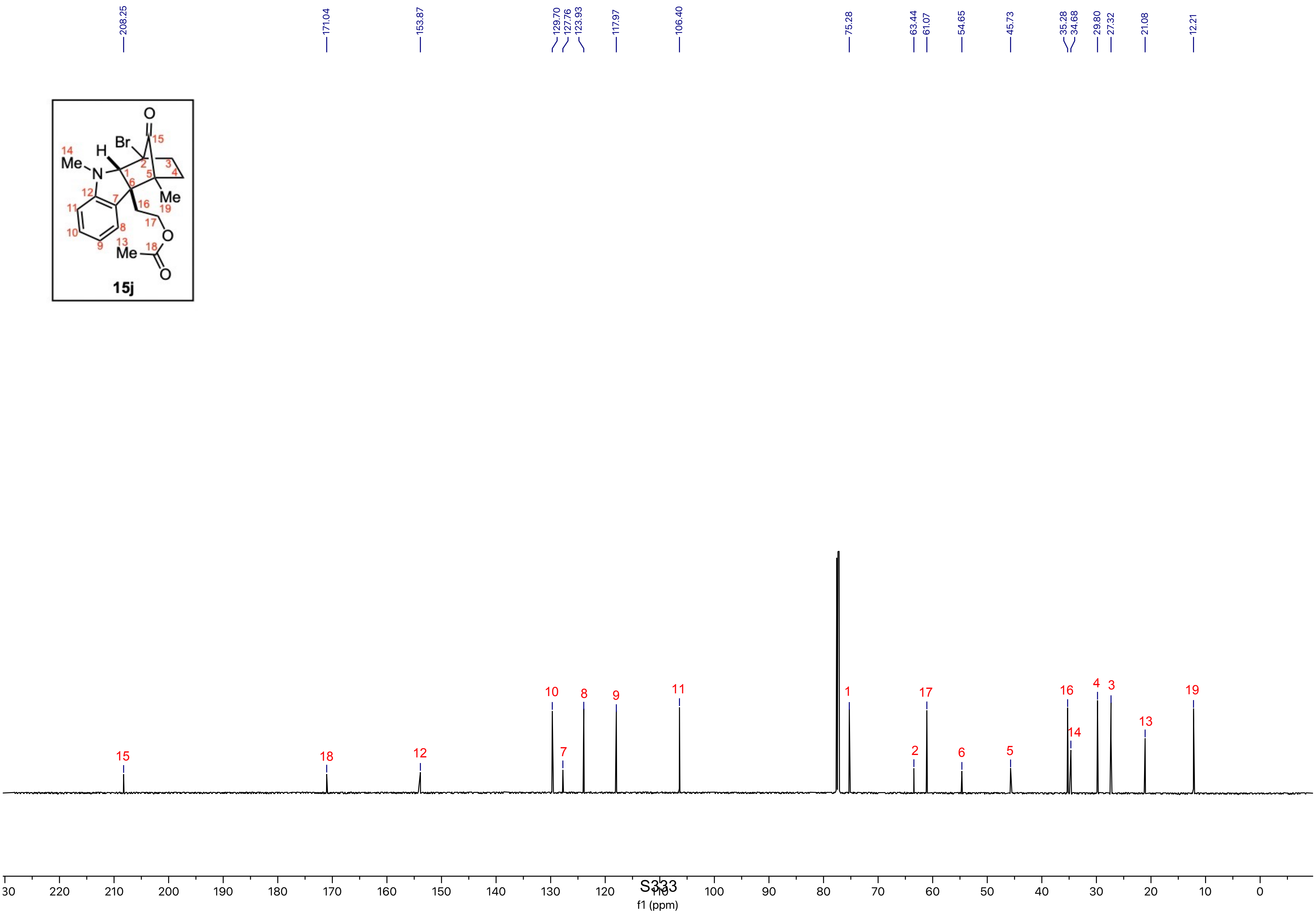

208.25
171.04
153.87
129.70
127.76
123.93
117.97
106.40
75.28
63.44
61.07
54.65
45.73
35.28
34.68
29.80
27.32
21.08
12.21
O
Br
H
Me
N
Me
Me
O
O
15j
f1 (ppm)

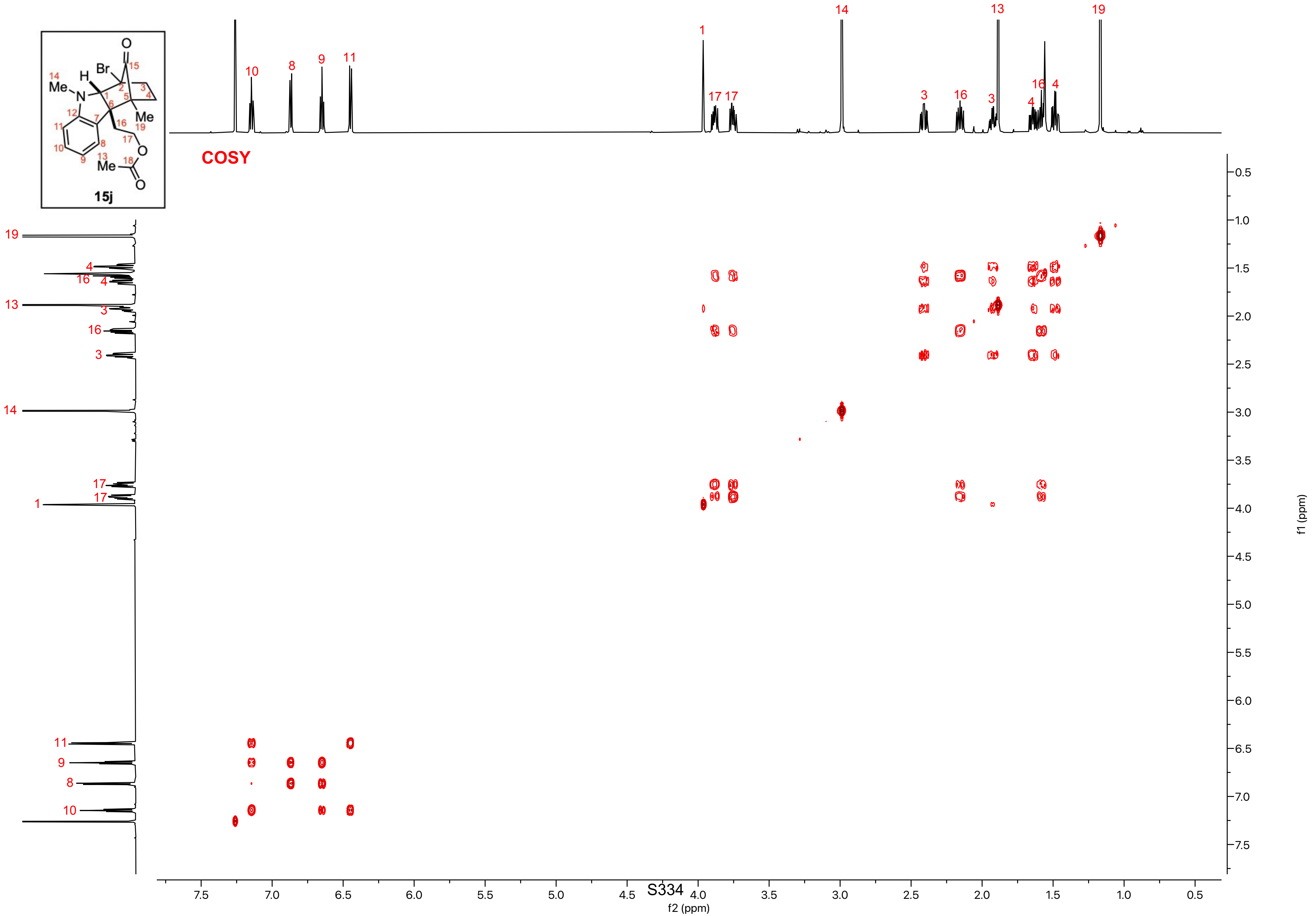
15j
COSY
f2 (ppm)
f1 (ppm)
7.5
7.0
6.5
6.0
5.5
5.0
4.5
4.0
3.5
3.0
2.5
2.0
1.5
1.0
0.5

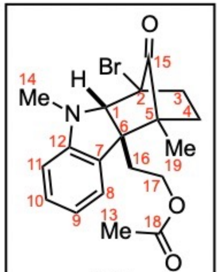

15j

HSQC

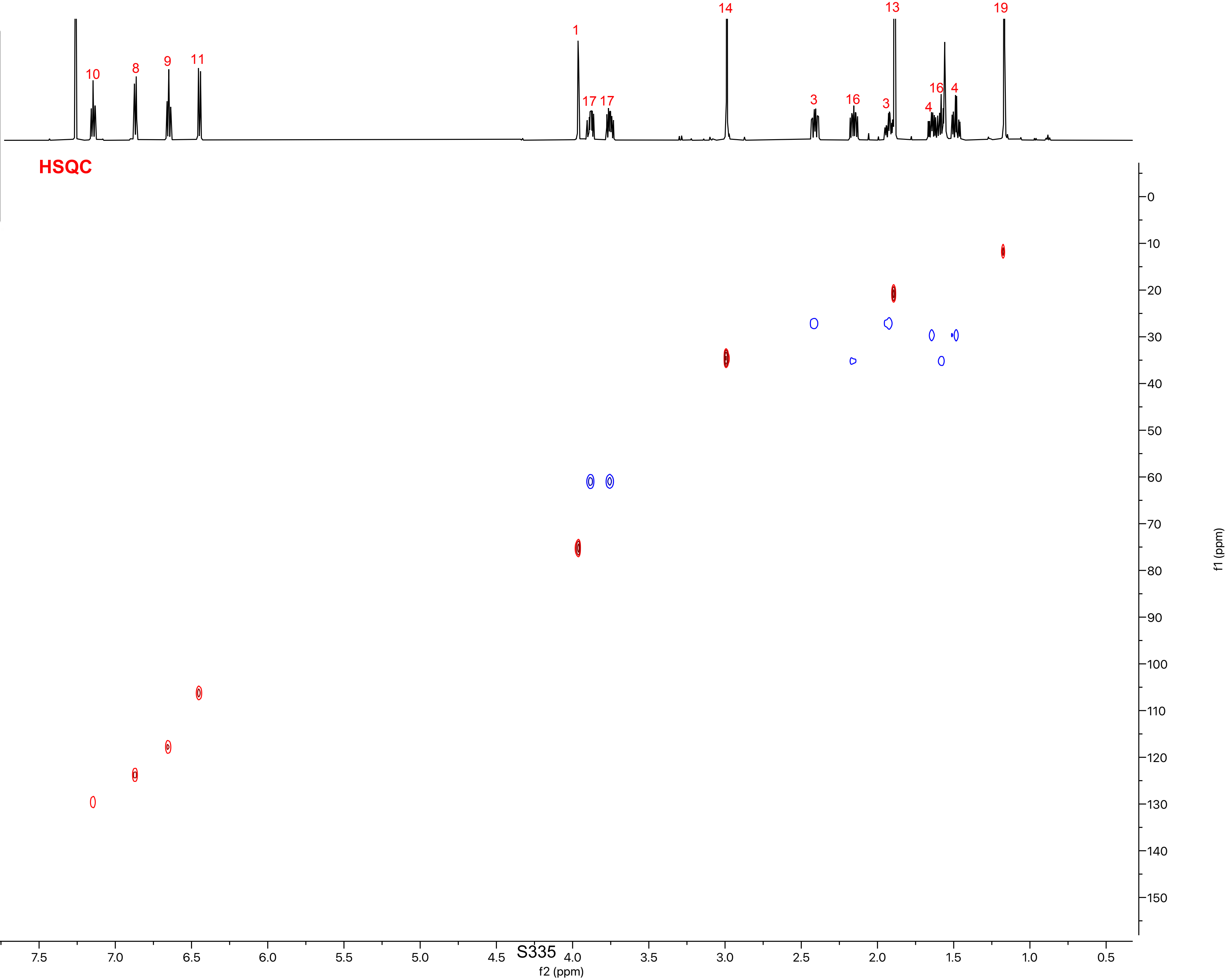

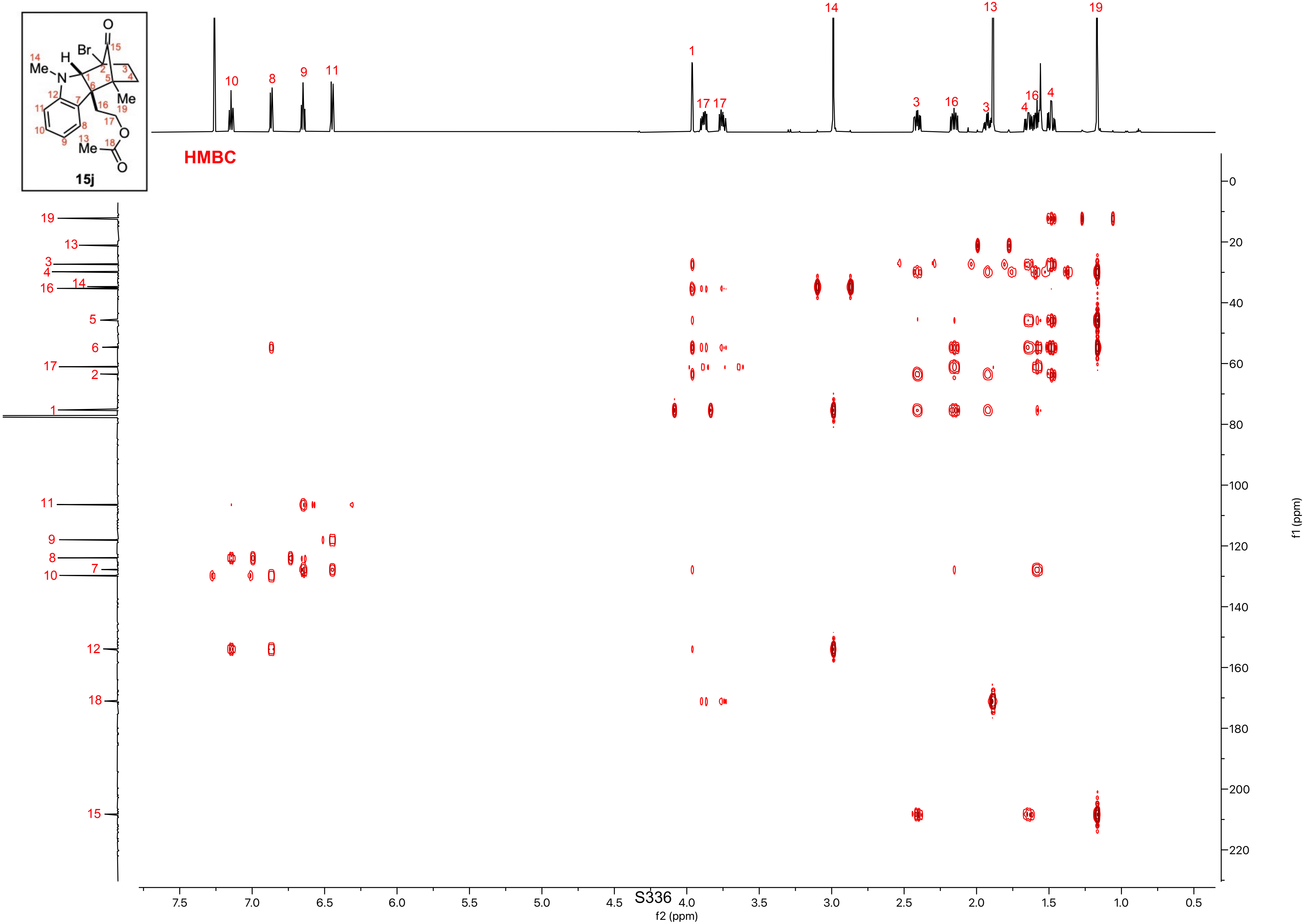

15j
HMBC
f2 (ppm)
f1 (ppm)

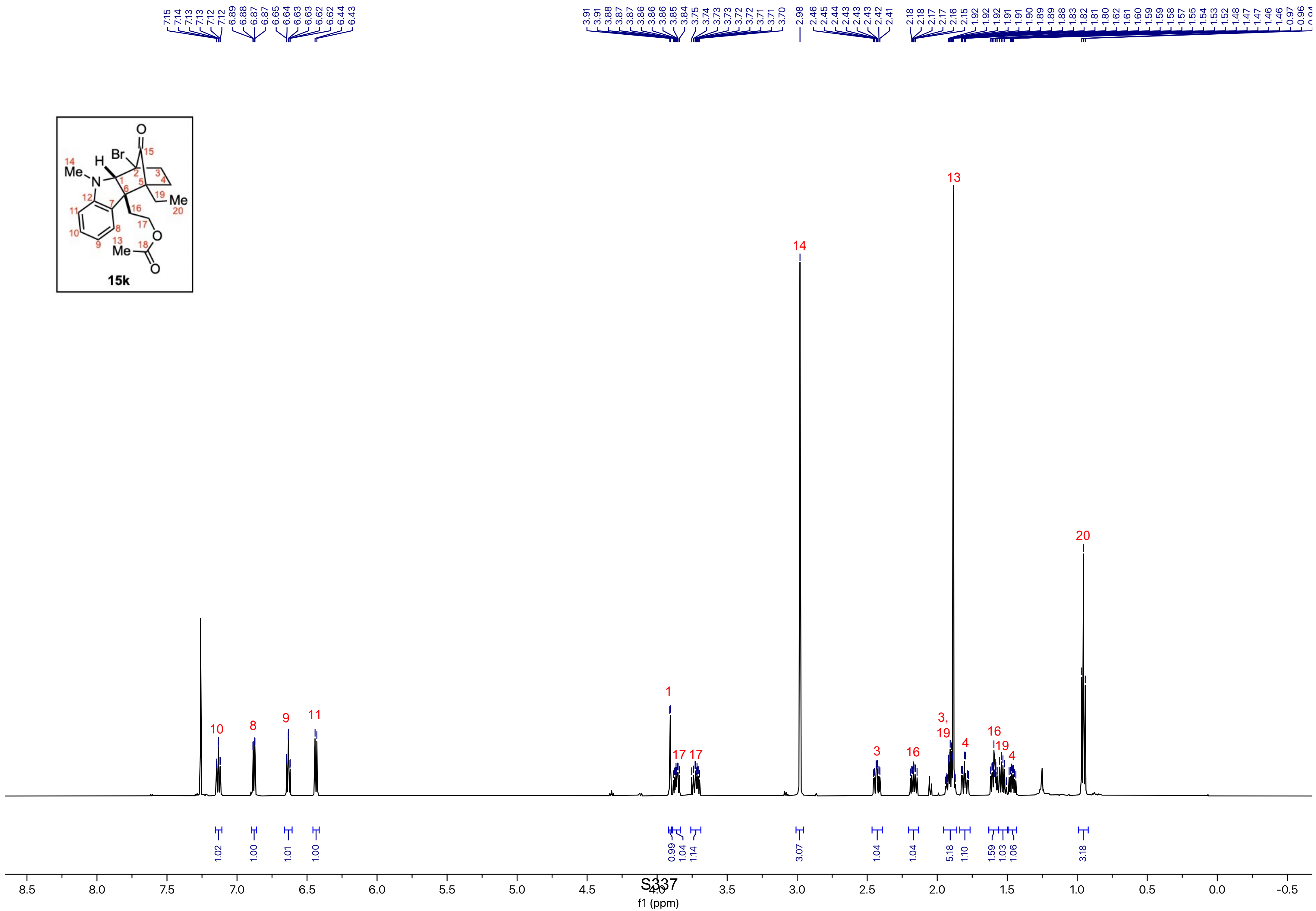
7.15 7.14 7.13 7.13 7.12 7.12 6.89 6.88 6.87 6.87 6.65 6.64 6.63 6.63 6.62 6.62 6.44 6.43
3.91 3.91 3.88 3.87 3.87 3.86 3.86 3.86 3.85 3.84 3.75 3.74 3.73 3.73 3.72 3.71 3.71 3.70
2.98
2.46 2.45 2.44 2.43 2.43 2.43 2.42 2.41
2.18 2.18 2.17 2.17 2.16 2.15 1.92 1.92 1.92 1.91 1.91 1.90 1.89 1.89 1.88 1.83 1.82 1.81 1.80 1.62 1.61 1.60 1.59 1.59 1.58 1.57 1.55 1.54 1.53 1.52 1.48 1.47 1.47 1.46 1.46 0.97 0.96
O
Br
H
Me
N
14
15
1
2
3
4
5
6
7
8
9
10
11
12
13
16
17
18
19
20
Me
O
Me
O
15k
10
8
9
11
1
17
17
14
3
16
3,
19
13
4
16
19
4
20
1.02
1.00
1.01
1.00
0.99
1.04
1.14
3.07
1.04
1.04
5.18
1.10
1.59
1.03
1.06
3.18
8.5 8.0 7.5 7.0 6.5 6.0 5.5 5.0 4.5 4.0 3.5 3.0 2.5 2.0 1.5 1.0 0.5 0.0 -0.5
f1 (ppm)

207.87
171.01
154.05
129.63
127.69
124.63
117.88
106.34
75.25
63.65
61.13
55.57
49.18
34.96
34.67
27.13
24.23
21.07
18.55
9.50

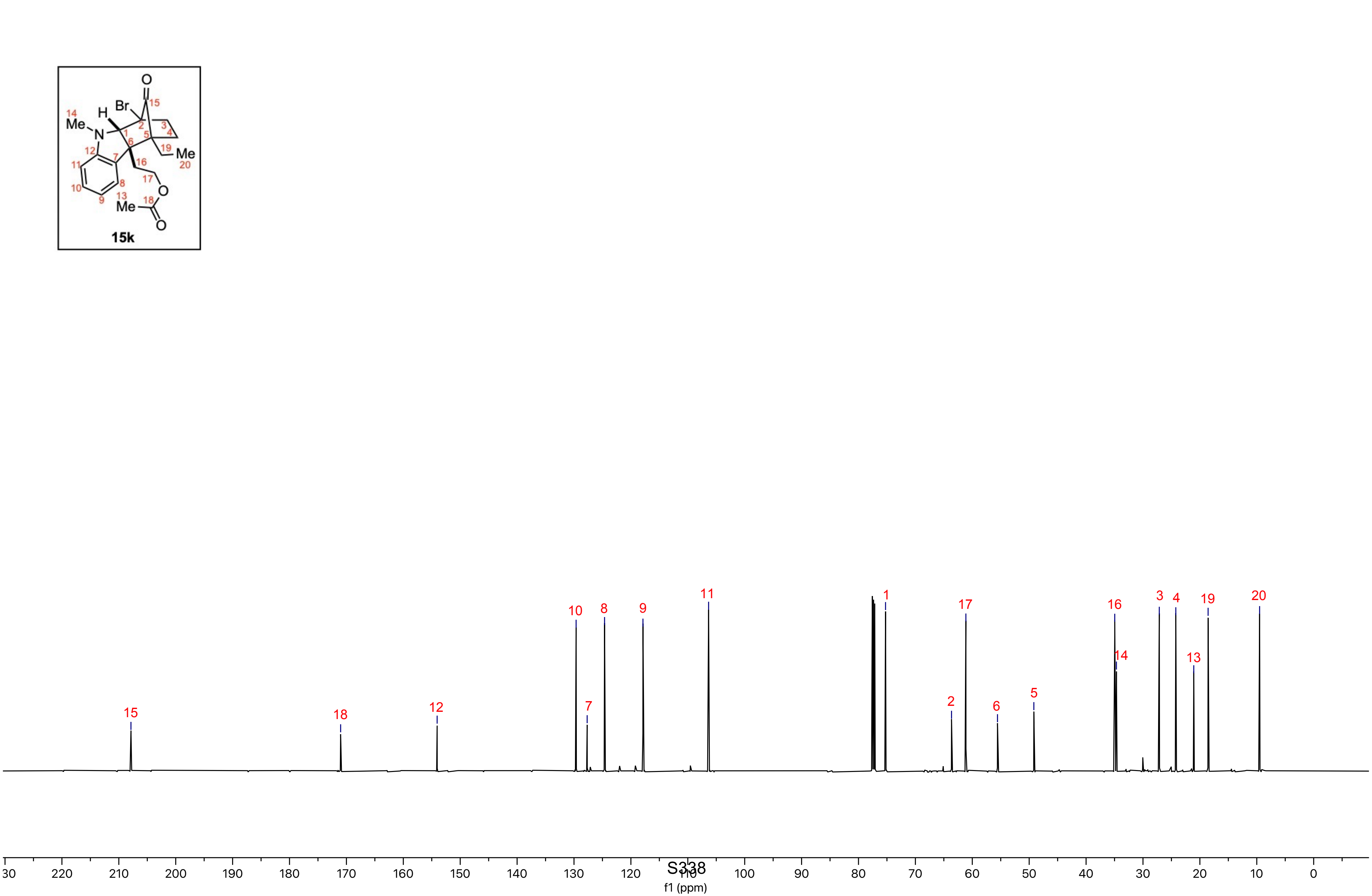

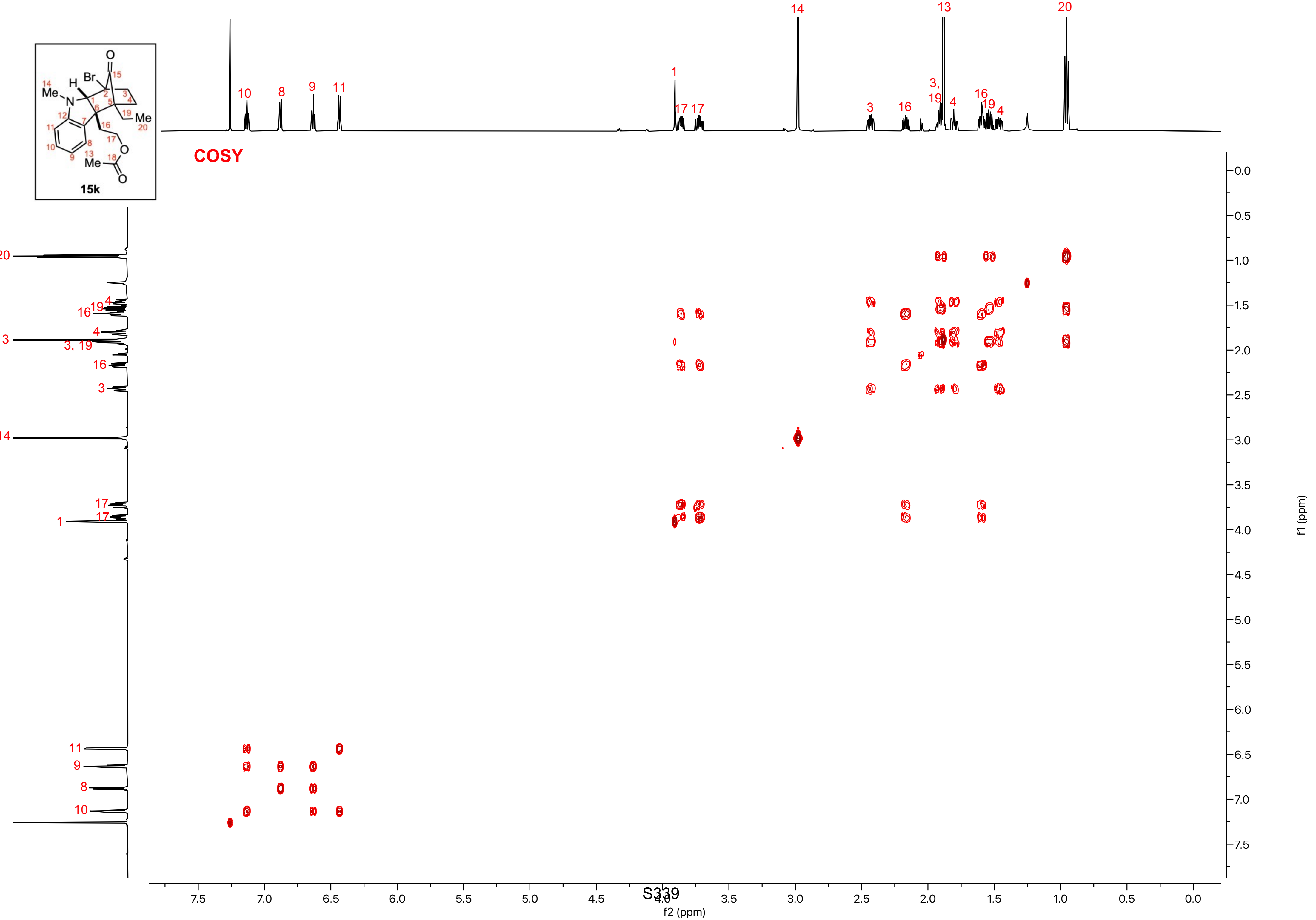

15k
COSY
14
13
20
1
17 17
10
8
9
11
3
16
3, 19
4
19
f2 (ppm)
f1 (ppm)

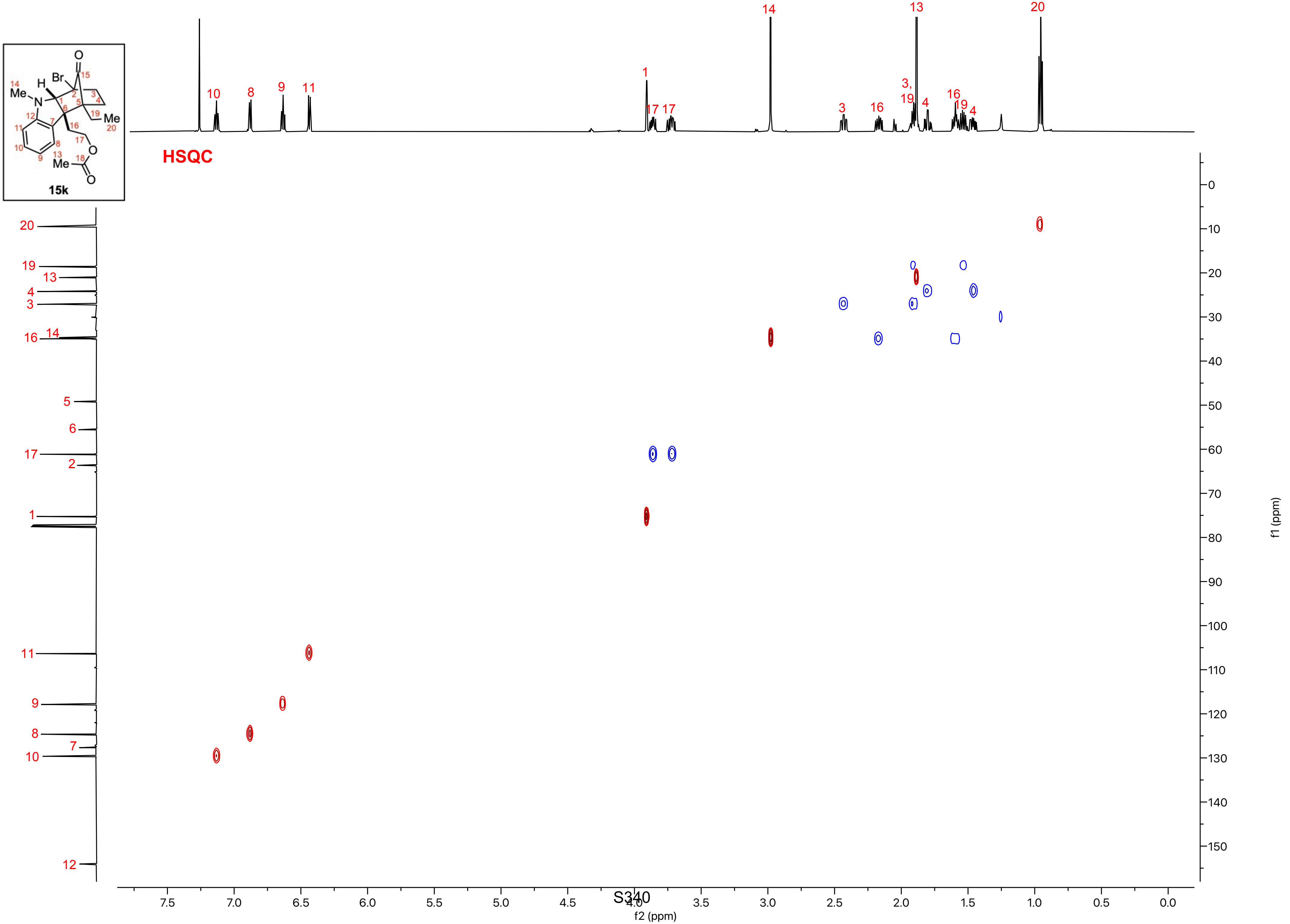

HSQC
15k
Me
N
H
Br
O
Me
O
Me
O
f2 (ppm)
f1 (ppm)

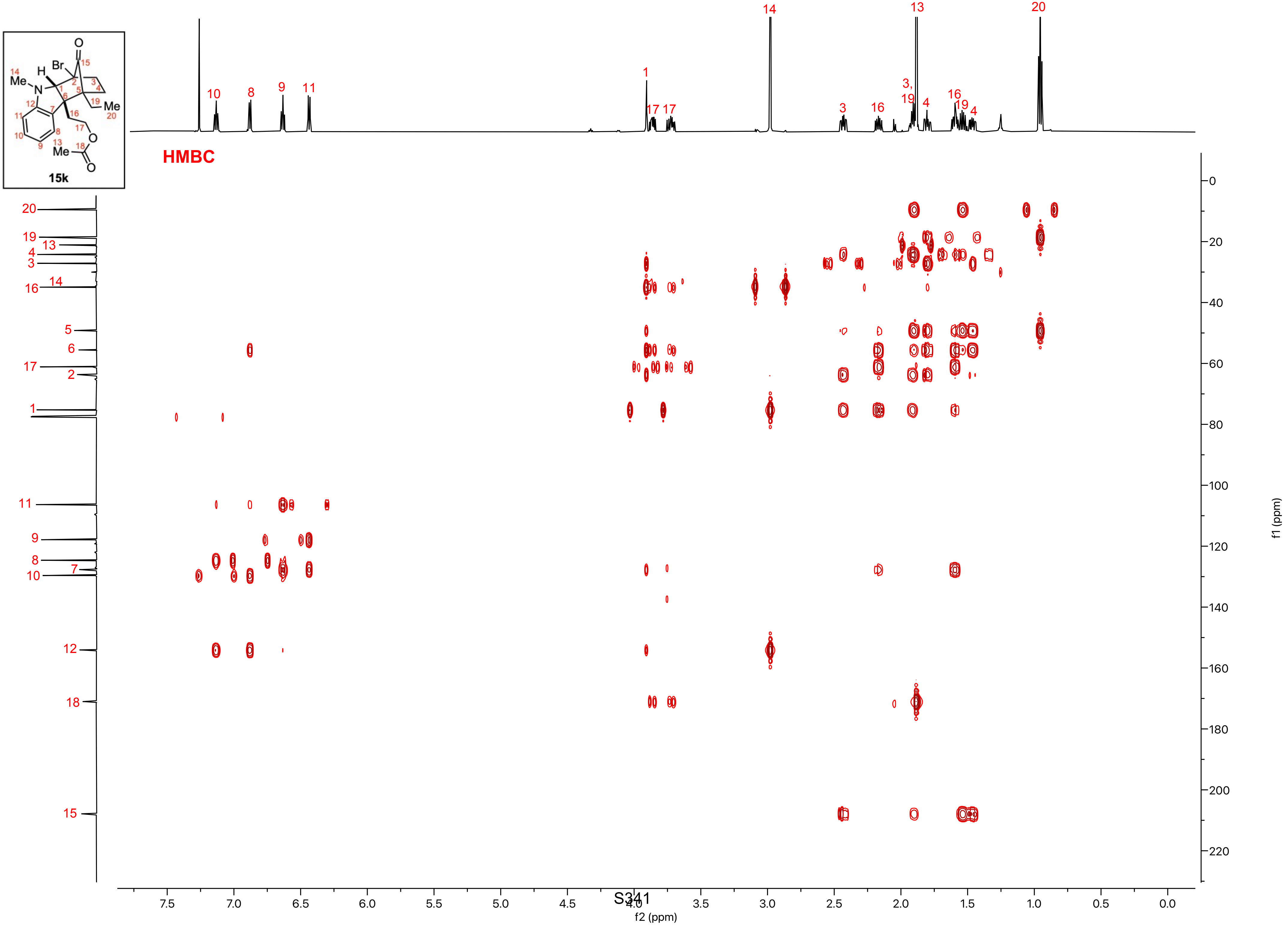
HMBC
15k
f2 (ppm)
f1 (ppm)

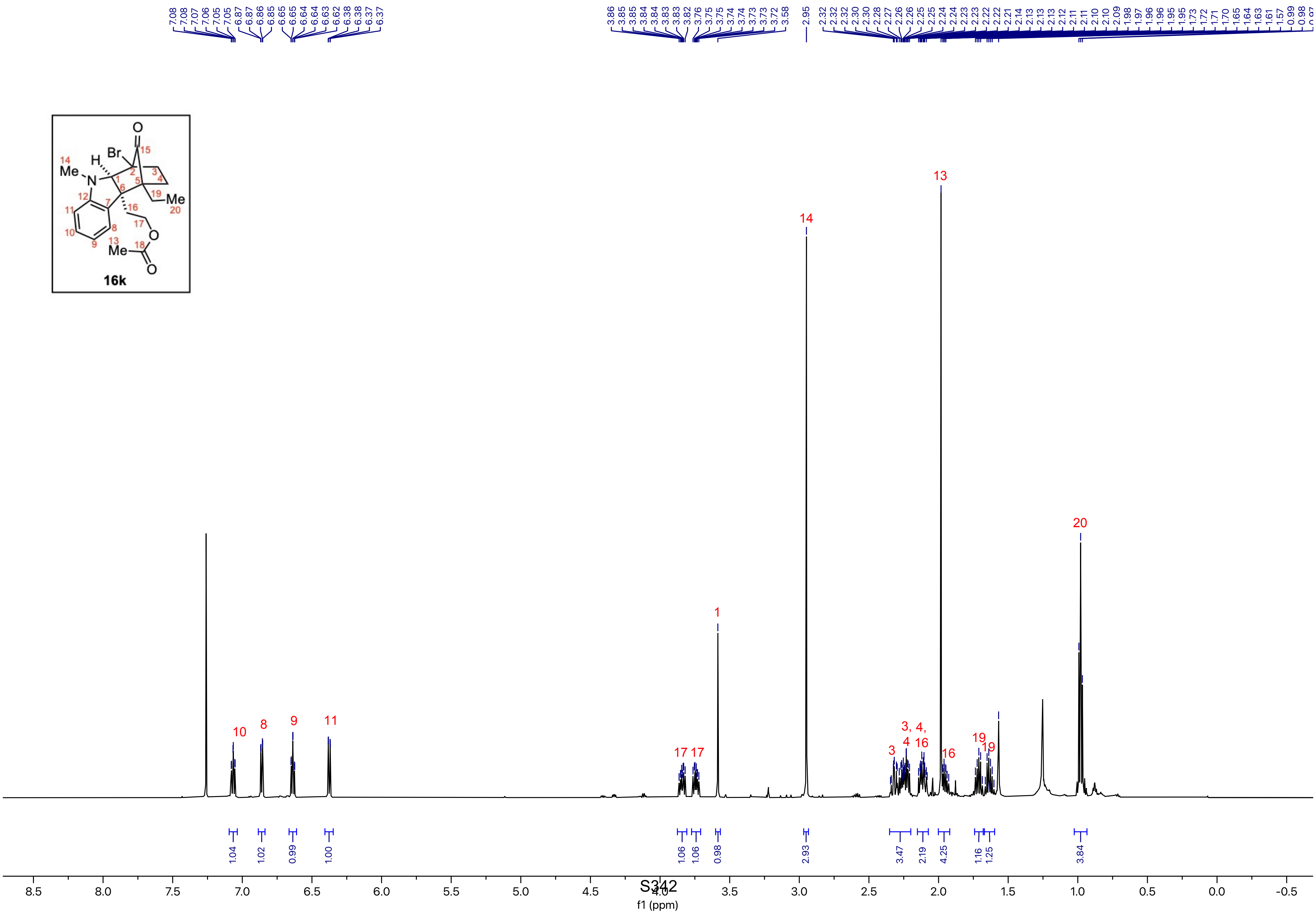
16k
f1 (ppm)

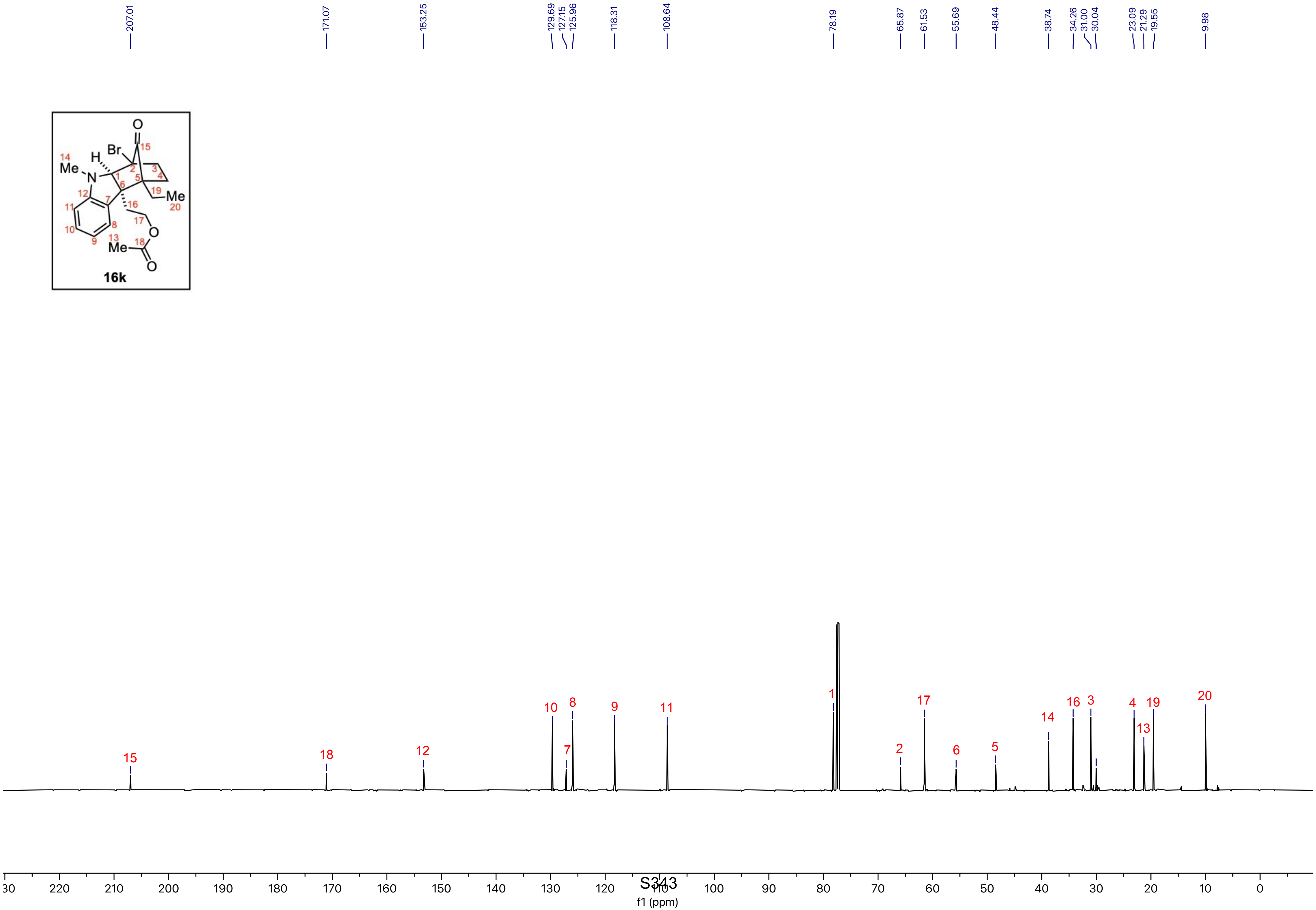
207.01
171.07
153.25
129.69
127.15
125.96
118.31
108.64
78.19
65.87
61.53
55.69
48.44
38.74
34.26
31.00
30.04
23.09
21.29
19.55
9.98
Br
H
Me
N
O
Me
Me
O
O
16k
f1 (ppm)

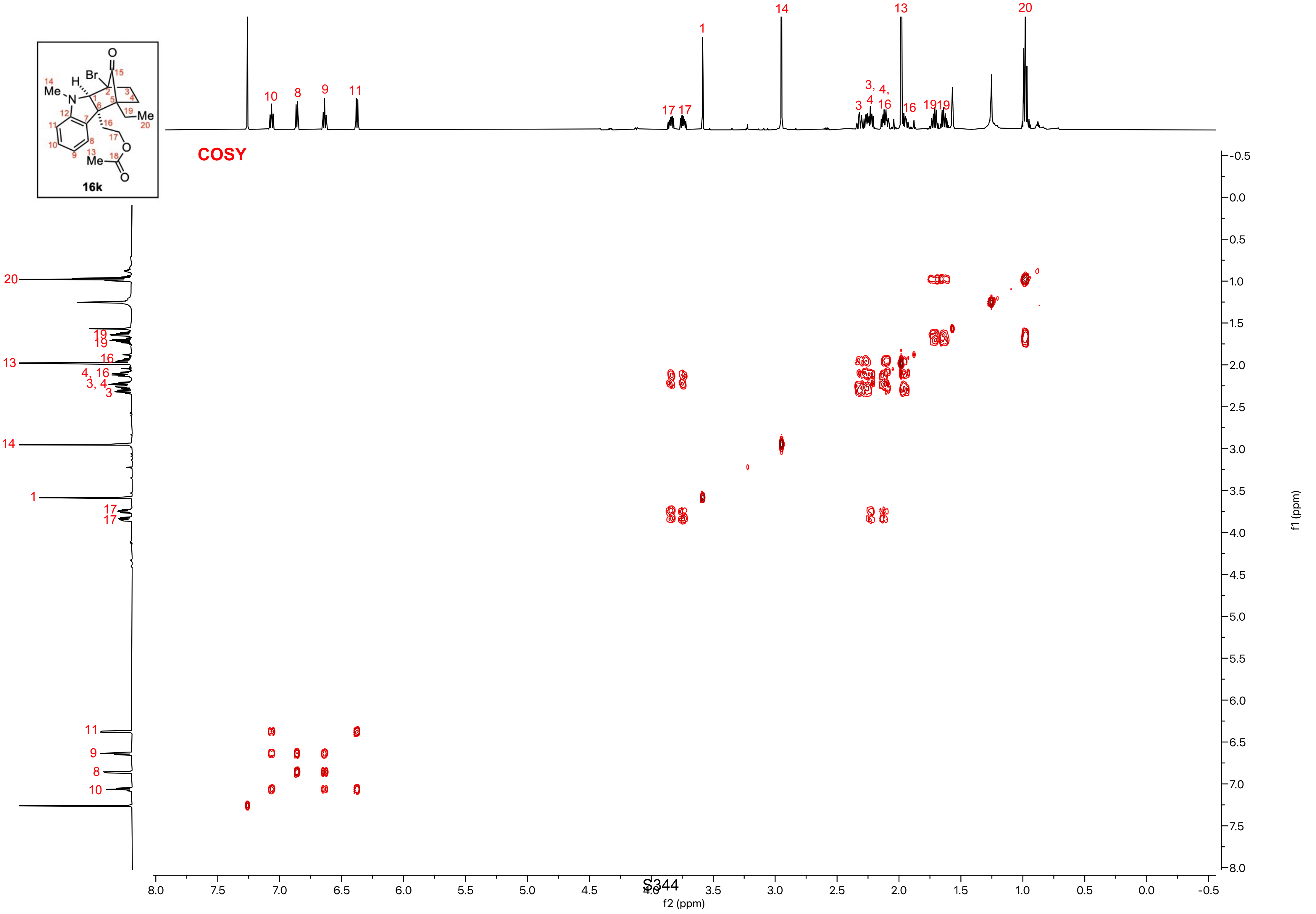

Br
H
Me
N
O
Me
Me
O
O
16k
COSY
f2 (ppm)
f1 (ppm)

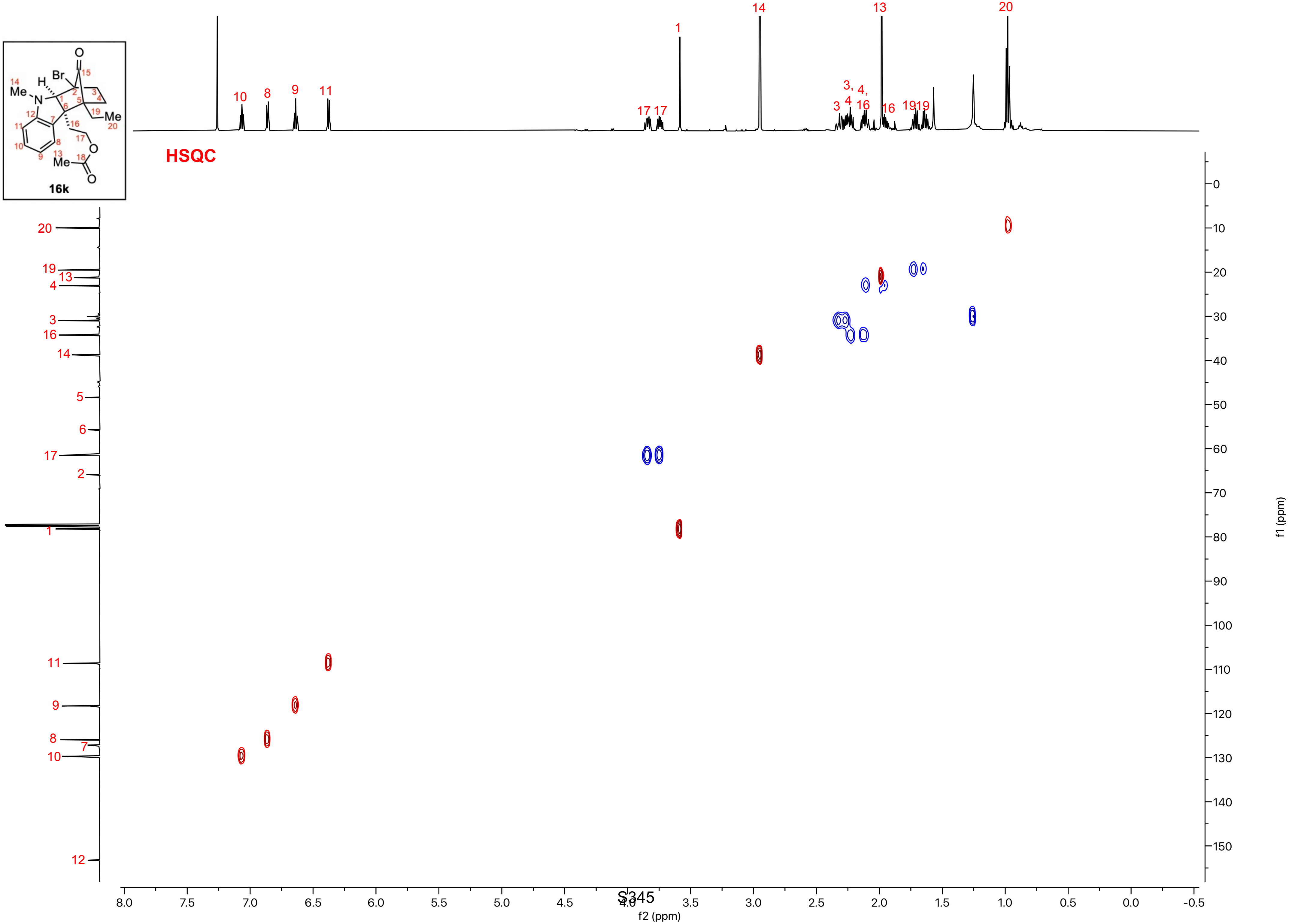
HSQC
16k
f2 (ppm)
f1 (ppm)

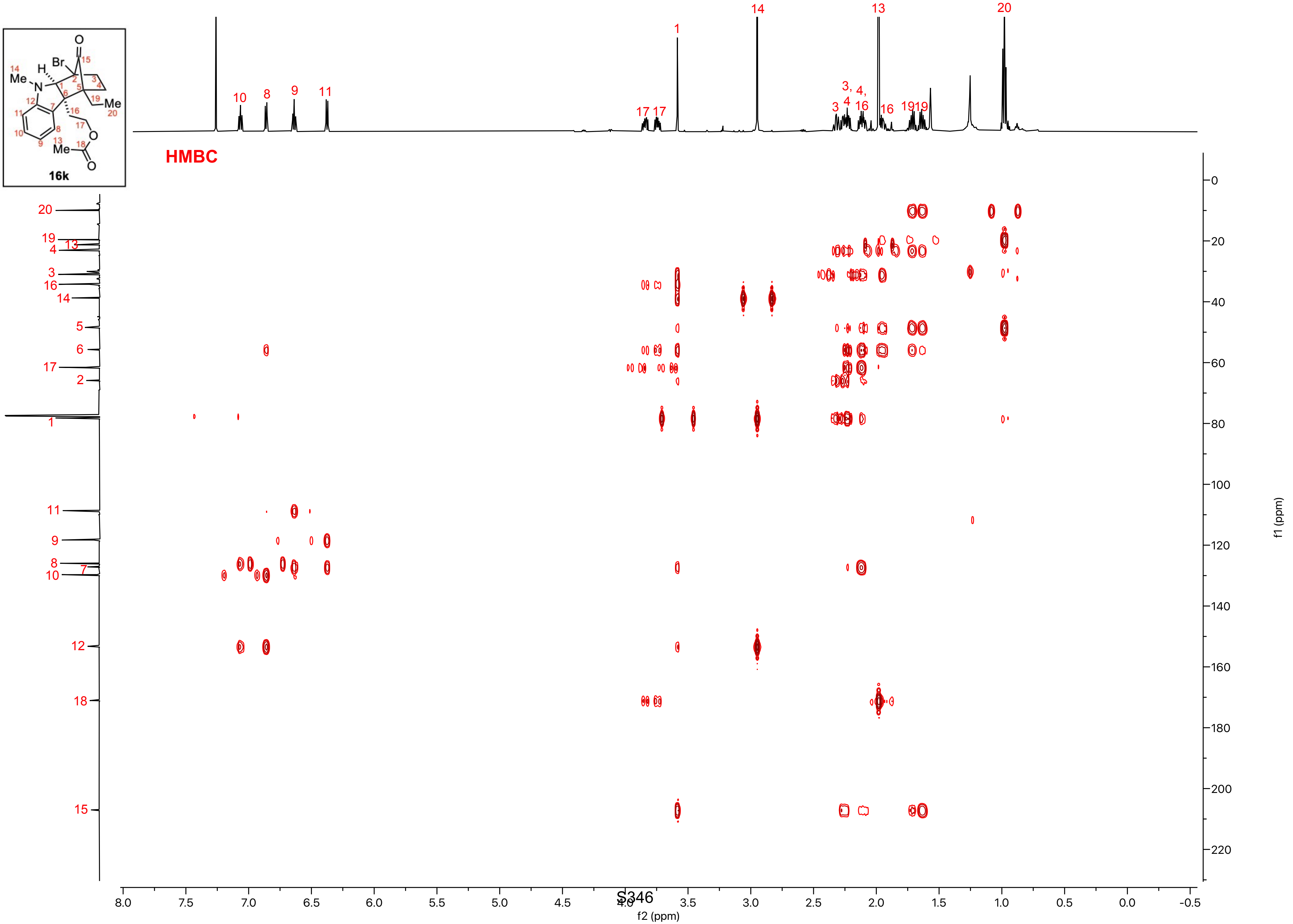

16k
HMBC
f2 (ppm)
f1 (ppm)

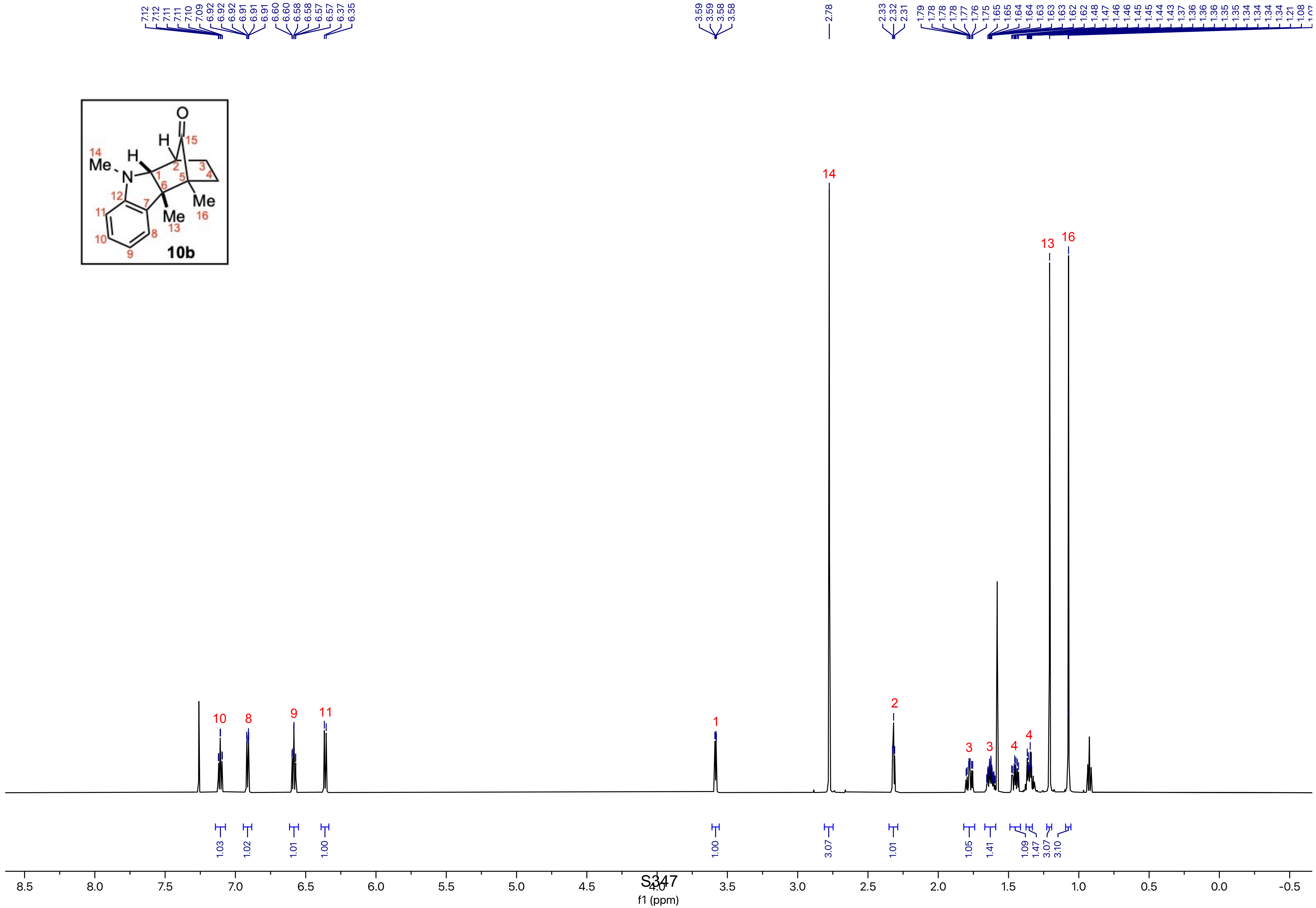
10b
f1 (ppm)

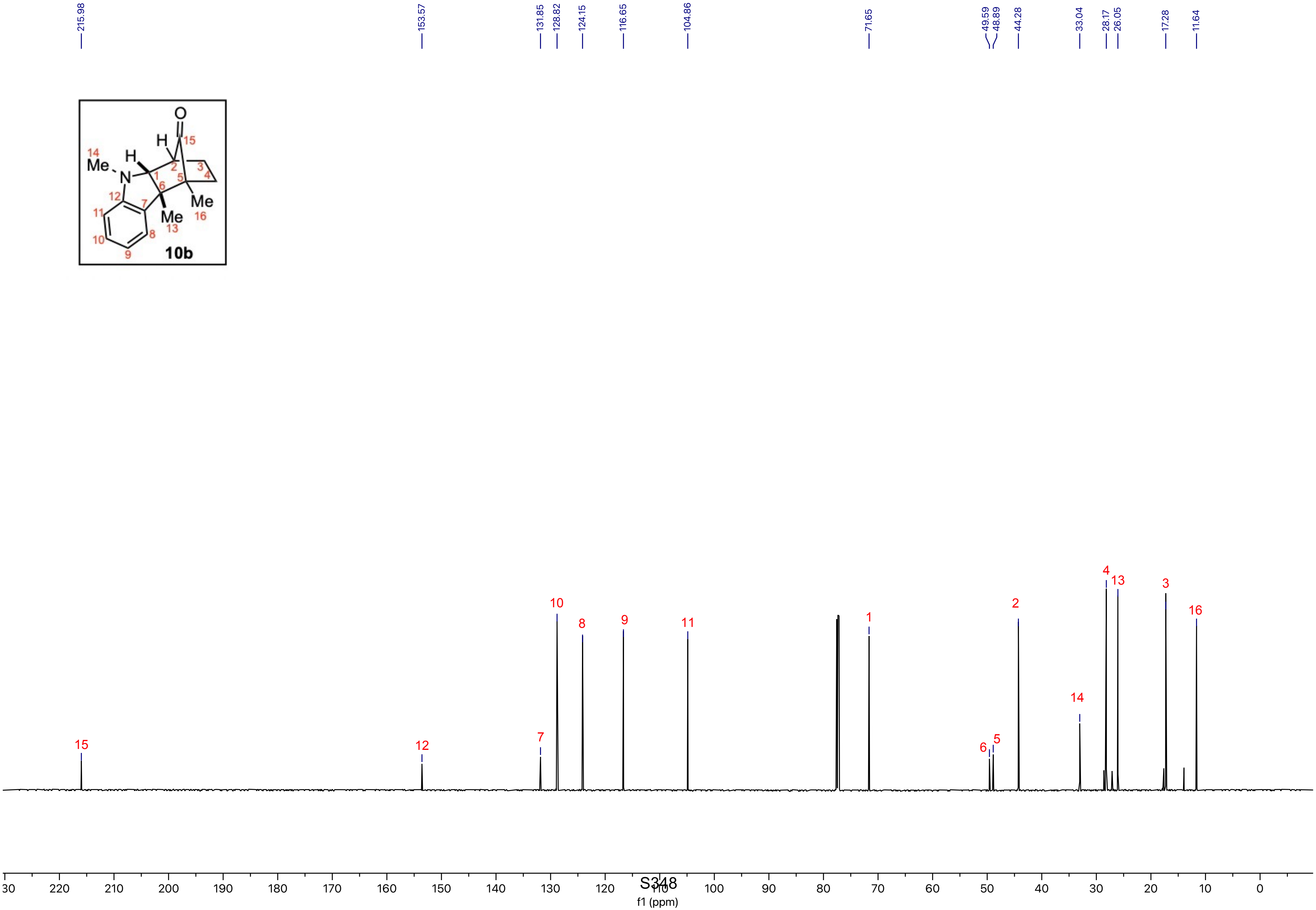
215.98
153.57
131.85
128.82
124.15
116.65
104.86
71.65
49.59
48.89
44.28
33.04
28.17
26.05
17.28
11.64
O
H
H
Me
N
Me
Me
10b
f1 (ppm)

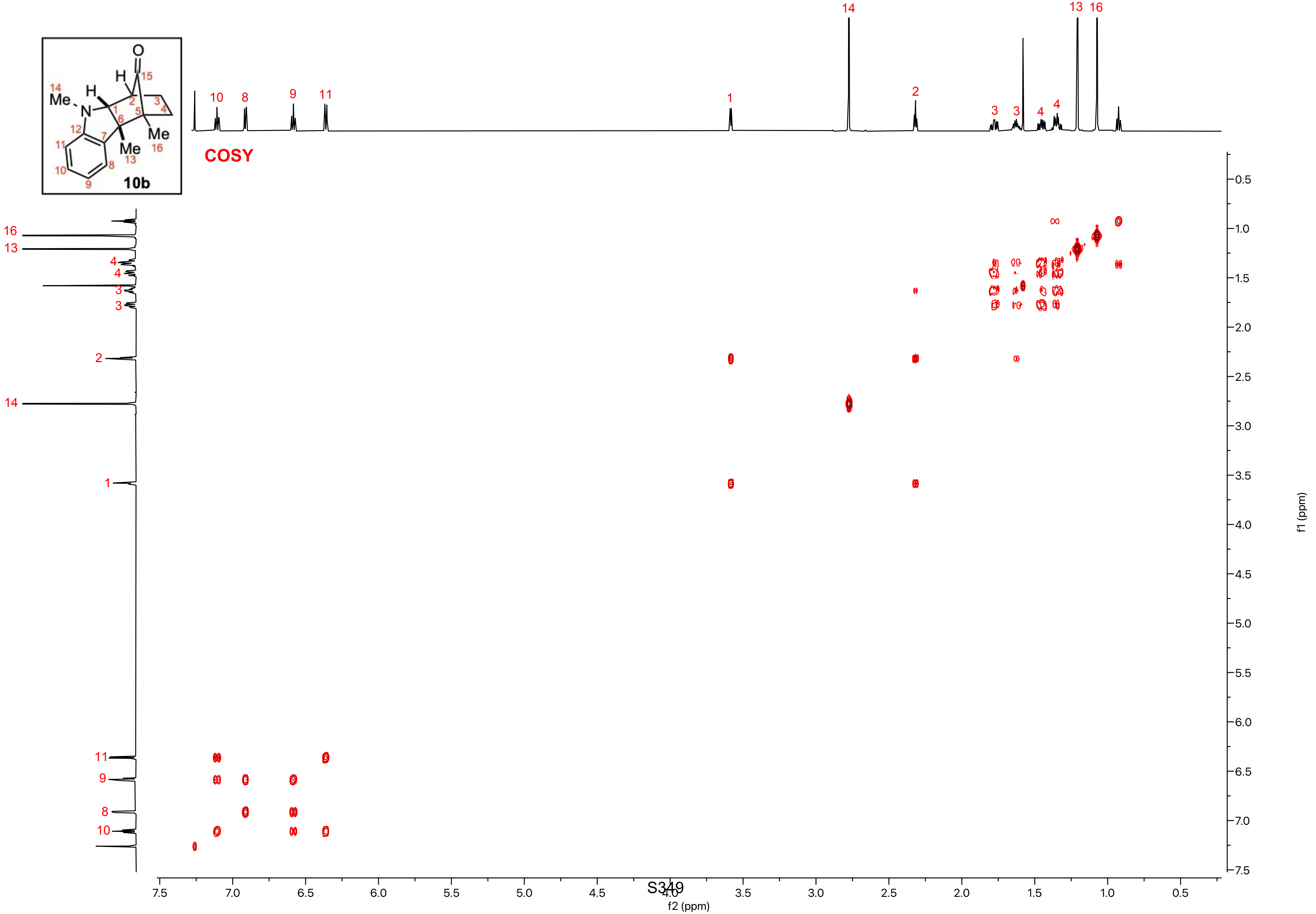
O
H
15
14
Me
H
N
1
2
3
4
6
5
Me
16
12
7
11
Me
13
10
8
9
10b
COSY
10
8
9
11
1
14
2
3
3
4
4
13
16
f2 (ppm)
f1 (ppm)

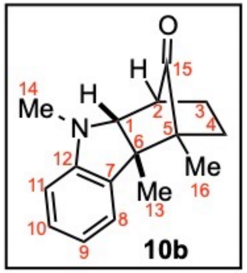
O
H
15
14
Me
H
N
1
2
3
4
5
6
12
7
Me
16
11
Me
13
10
8
9
10b

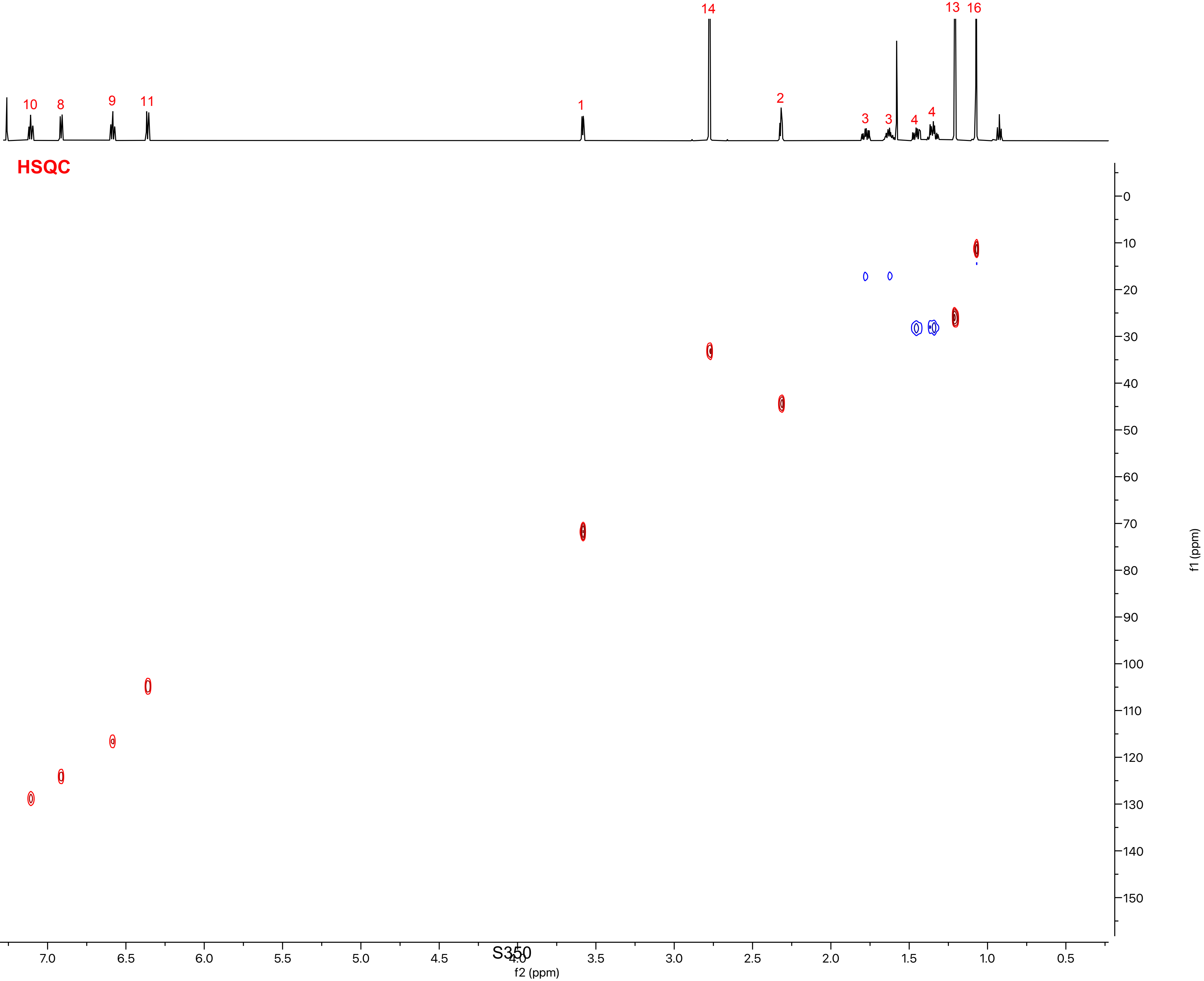
HSQC
10
8
9
11
1
14
2
3
3
4
4
13
16
f2 (ppm)
f1 (ppm)

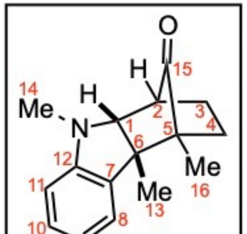

O
H
15
14
Me
N
H
1
2
3
4
5
6
Me
16
12
7
11
Me
13
10
8
9

10b

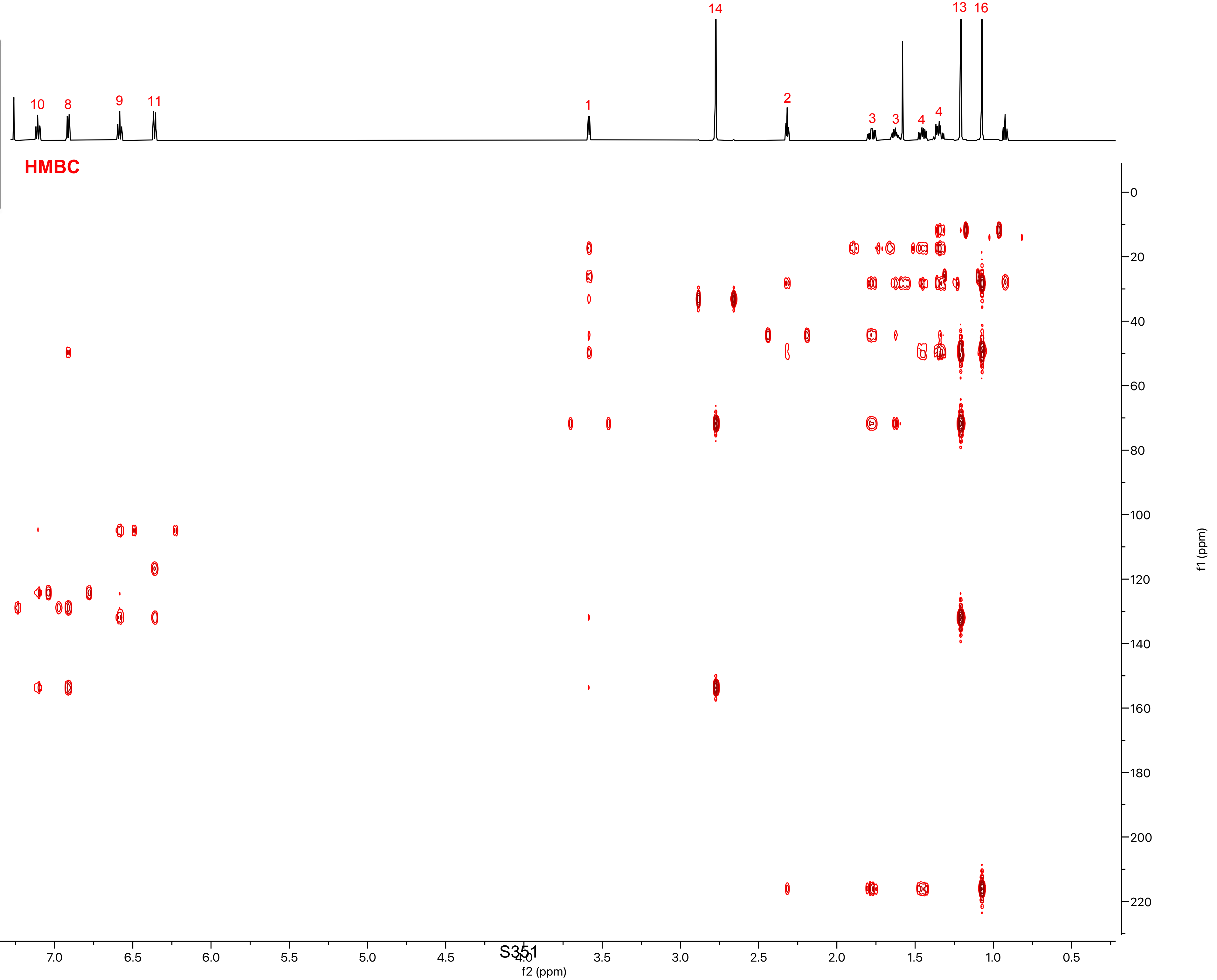

HMBC
10
8
9
11
1
14
2
3
3
4
4
13
16
16
3
13
4
14
2
5
6
1
11
9
8
10
7
12
15
0
20
40
60
80
100
120
140
160
180
200
220
f1 (ppm)
7.5
7.0
6.5
6.0
5.5
5.0
4.5
4.0
3.5
3.0
2.5
2.0
1.5
1.0
0.5
f2 (ppm)
